# Neotropical Bats: Estimating Species Diversity with DNA Barcodes

**DOI:** 10.1371/journal.pone.0022648

**Published:** 2011-07-26

**Authors:** Elizabeth L. Clare, Burton K. Lim, M. Brock Fenton, Paul D. N. Hebert

**Affiliations:** 1 Department of Integrative Biology, University of Guelph, Guelph, Ontario, Canada; 2 Department of Natural History, Royal Ontario Museum, Toronto, Ontario, Canada; 3 Department of Biology, University of Western Ontario, London, Ontario, Canada; Biodiversity Insitute of Ontario - University of Guelph, Canada

## Abstract

DNA barcoding using the cytochrome *c* oxidase subunit 1 gene (COI) is frequently employed as an efficient method of species identification in animal life and may also be used to estimate species richness, particularly in understudied faunas. Despite numerous past demonstrations of the efficiency of this technique, few studies have attempted to employ DNA barcoding methodologies on a large geographic scale, particularly within tropical regions. In this study we survey current and potential species diversity using DNA barcodes with a collection of more than 9000 individuals from 163 species of Neotropical bats (order Chiroptera). This represents one of the largest surveys to employ this strategy on any animal group and is certainly the largest to date for land vertebrates. Our analysis documents the utility of this tool over great geographic distances and across extraordinarily diverse habitats. Among the 163 included species 98.8% possessed distinct sets of COI haplotypes making them easily recognizable at this locus. We detected only a single case of shared haplotypes. Intraspecific diversity in the region was high among currently recognized species (mean of 1.38%, range 0–11.79%) with respect to birds, though comparable to other bat assemblages. In 44 of 163 cases, well-supported, distinct intraspecific lineages were identified which may suggest the presence of cryptic species though mean and maximum intraspecific divergence were not good predictors of their presence. In all cases, intraspecific lineages require additional investigation using complementary molecular techniques and additional characters such as morphology and acoustic data. Our analysis provides strong support for the continued assembly of DNA barcoding libraries and ongoing taxonomic investigation of bats.

## Introduction

DNA barcoding studies employ the mitochondrial cytochrome *c* oxidase subunit 1 gene (COI) as a tool for species identification and discovery through the comparison of inter- and intraspecific sequence divergences [1]. The effectiveness of this technique has been validated in various animal groups, where most species are characterized by highly similar haplotypes with low intraspecific variation and substantial divergence from closely related taxa [1]–[5]. In a few cases incomplete lineage sorting or shared barcode haplotypes exist between hybridizing or closely related taxa [5], [6] limiting identifications for several groups of species (invariably within a genus). Conversely, most prior barcode studies have generated hypotheses about the existence of cryptic species based on unusually high genetic divergence between intraspecific lineages, some of which have subsequently been recognized as having morphological or ecological differences e.g. [7], supporting the use of barcoding for species discovery.

Assembling a reference database of DNA barcode sequences for mammals represents an obvious target for the global DNA barcode of life campaign. Mammals are a large, charismatic and relatively well-studied group of animals, but a modest objective with just over 5400 species recognized in 2007 [8] making the assembly of a DNA barcoding reference library a readily attainable goal. Despite the popular assumption that most mammals have been described, the rate of species discovery has actually accelerated recently [8] particularly with the aid of new molecular technologies. Bats (order Chiroptera) represented approximately 20% (1116 of 5416) of all mammal species indexed in 2005 [9] but the incidence of overlooked taxa is likely to be particularly high within this group due to their cryptic nocturnal, volant behaviour and often subtle morphological differences between species.

Most past DNA barcode studies of mammals have concentrated on local faunas or have had a taxonomically limited scope and include two studies of primates [10], [11], one survey of bats [4], one survey of small mammals [12], a methodological study [3] and a taxonomic revision of the bat *Myotis phanluongi* [13]. Molecular taxonomic surveys of bats using mitochondrial genes other than COI have been conducted in Europe [14] using ND1 and in Central and South America [15] using cytochrome b. In both cases, numerous hypotheses regarding cryptic speciation were advanced. The largest study of bats to date [16] included 1896 specimens representing 157 bat species in South East Asia and speculated that taxonomic richness in this area may be underestimated by more than 50%. Francis et al. [16] also speculate that rates of endemism are much higher than previously recognized by classical morphology, a conclusion which has great conservation implications for the region.

Bradley and Baker [17] derived a set of criteria for evaluating the taxonomic implications of genetic diversity at mitochondrial loci (particularly cytochrome b): values <2% were indicative of intraspecific variation, values between 2 and 11% were often indicative of variation between species (thus species with intraspecific values in this range require additional taxonomic scrutiny) and values >11% invariably indicated the presence of other congeneric species. Baker and Bradley [15] defined a theoretical framework for a genetic species concept for mammals and, using criteria similar to Bradley and Baker [17], evaluated cytochrome b sequences from 718 specimens representing 61 Neotropical mammal species (29 of which were bats). In total, Baker and Bradley [15] identified 32 cases (11 in bats) where a currently recognized species contained “phylogroups” with substantial DNA sequence variation (>5%) suggesting the presence of cryptic species and concluded that the species richness of mammals in Neotropical regions may be significantly under diagnosed. While similar to the conclusion of Francis et al. [16], it is somewhat surprising because, although the Neotropics contain some of the highest bat species diversity in the world [18], they have also received considerable taxonomic scrutiny e.g. [19]–[24]. Given the increasing evidence suggesting that cryptic diversity is prevalent in this region [4], [12], [15] a comprehensive survey of potential diversity is needed on a scale which is taxonomically diverse, geographically broad, and includes many representatives per species.

Here we examine patterns of COI sequence divergence in 9076 vouchered specimens from 163 bat species spanning collections from 13 countries across the continental Neotropics. To the best of our knowledge, it is one of the largest molecular surveys of biodiversity ever conducted and certainly the largest for land vertebrates. We evaluate these species with the following goals: 1) to assess genetic variation, 2) to estimate the number of distinct intraspecific mitochondrial lineages and 3) to evaluate the distance-based criteria used by Bradley and Baker [17] to categorize mitochondrial diversity. We use these data to estimate the potential taxonomic richness of the area and to provide a framework for further taxonomic investigation.

## Methods

### Sample Acquisition

We sampled preserved tissue from 9076 vouchered specimens held at the Royal Ontario Museum, representing 163 species from 65 genera including representatives from all nine bat families present within Central and South America. We followed the taxonomic designations of Simmons [9] with the following exceptions: we retained *Artibeus intermedius* as distinct from *A. lituratus* (R.J. Baker, pers comm.), *A. planirostris* as distinct from *A. jamicensis* following Lim et al. [23], *A. bogotensis* as distinct from *A. glaucus* [24], a species of *Choeroniscus* in the western Amazon distinct from *C. minor* due to a taxonomic revision in progress, and *Molossus sp.* as an undescribed species in Guyana following Lim and Engstrom [21] and Clare et al. [4]. Details on all specimens (sampling location, GPS co-ordinates of collection, voucher number etc.) are available within the “Bats of the Neotropics” project in the Barcode of Life Data Systems (BOLD, www.barcodinglife.org). Records from previously published data used here are contained on BOLD within the projects “Bats of Guyana” [4], “BMC *Sturnira*” [3] and “Small mammal survey in Bakhuis, Suriname” [12]. Our protocols for DNA extraction, amplification and sequencing follow Clare et al. [4], Ivanova et al. [25], [26] and Borisenko et al. [12]. Genbank, BOLD and Museum accessions for all sequences are located in Table S1.

### Data analysis

We aligned sequences using SeqScape v.2.1.1 (Applied Biosystems) and edited them manually. Sequences and original trace files are available in the BOLD projects described earlier. We calculated sequence divergences using the Kimura-two-parameter (K2P) model of base substitution [27] and generated a neighbor-joining (NJ) tree of K2P distances showing intra- and interspecific variation in BOLD (Figure S1). We generated all other trees in MEGA [28] as NJ trees of K2P sequence variation. Given the number of sequences and that phylogeny/branch arrangements were not a goal of this analysis, branch support was calculated on subsets of species for simplicity using 1000 bootstrap replications.

## Results

### Molecular Taxonomic Identification

Our analysis included a mean of 56 individuals per species (range 1–1013, median = 11) with 147 species represented by multiple samples. The NJ tree of COI sequence divergence for all individuals (Figure S1) demonstrates that only two species, *Artibeus lituratus* and *A. intermedius*, are not differentiated by COI sequences. In both species, levels of intraspecific variation are similar to other species in the genus (*A. lituratus* mean = 0.69% and *A. intermedius* mean = 0.79%) but form a single reciprocally monophyletic cluster with many common haplotypes. Mean intraspecific sequence variation in all species represented by ≥3 sequences was 1.38% (equal weighting regardless of sample size), but varied from 0–11.79%. Mean intraspecific variation was not correlated to sample size (one tailed test, *r* = 0.03, p = 0.74 for all species with n≥3).

**Figure 1:**
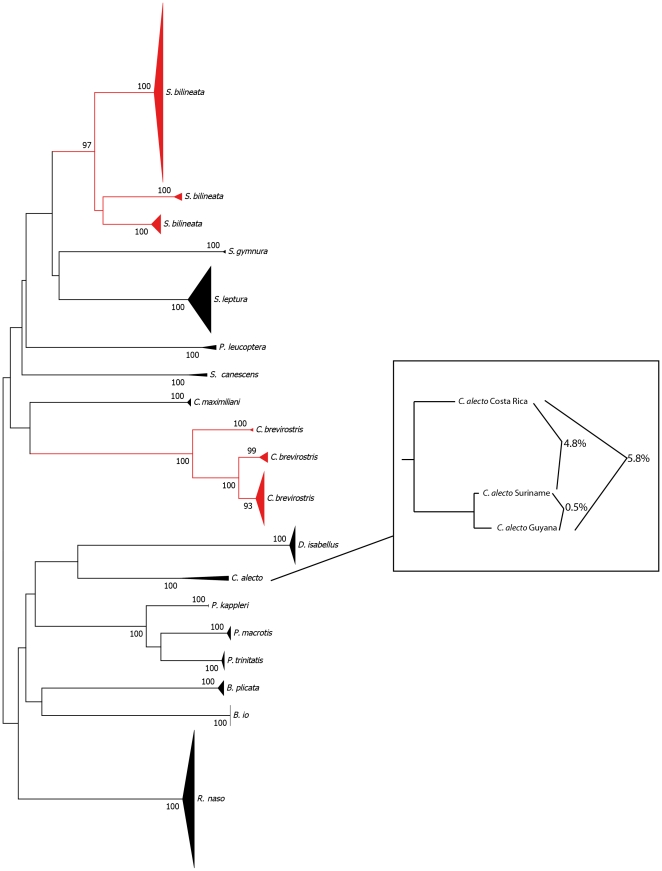
A neighbour-joining tree of COI sequence divergence (K2P) in surveyed species in the family Emballonuridae. All currently recognized species are supported by bootstrap values ≥97 (1000 replications). Triangles indicate the relative number of individuals sampled (height) and sequence divergence (width). In two cases, *Saccopteryx bilineata* and *Cormura brevirostris* (highlighted in red) deep intraspecific mitochondrial lineages are present which are strongly supported indicating the need for additional taxonomic scrutiny. The identification of intraspecific lineages can be hindered by small sample sizes from large geographic areas (e.g. *Cyttarops alecto*) where divergent sequences may represent independent lineages or poorly sampled intraspecific variation.

**Figure 2:**
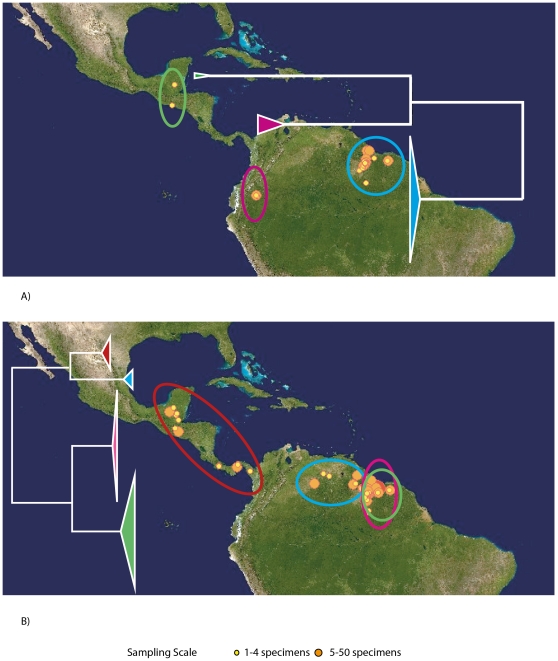
Allopatric and sympatric divergences of COI. Intraspecific clusters within *Saccopteryx bilineata* (A) are allopatric. One cluster in *Pteronotus parnellii* (B) exists in Central America, while the other three have potential zones of sympatry in Guyana.

Using the criteria established by Bradley and Baker [17] we observed 107 species with <2% mean sequence divergence which would be classified as intraspecific variation whereas 29 had between 2 and 11% mean sequence divergence and would be classified as potentially containing cryptic species requiring additional taxonomic scrutiny, and one species contained >11% mean sequence divergence. A visual inspection of the structure of the NJ trees (Figure S1, Figure S2) suggests that at least 44 of the species surveyed may contain distinct intraspecific mitochondrial lineages (e.g. Figure 1) with substantial divergence from other conspecifics, most supported by bootstrap values ≥90 (Table 1, 2, 3, 4, 5, 6, 7, 8). In some cases, these lineages represent a single divergent haplotype in the dataset which may reflect rare mutations within a geographic area (e.g. *Pteronotus personatus* Figure S1, Figure S2) rather than distinct lineages. In other cases, small sample sizes from large geographic areas (e.g. *Cyttarops alecto* Figure 1) hinder the interpretation of mitochondrial sequence variation because divergent sequences may represent independent lineages or panmictic intraspecific variation that is poorly sampled. Divergent intraspecific lineages are found with both allopatric (e.g. Figure 2a) and sympatric (e.g. Figure 2b) distributional patterns.

**Table 1:** Emballonuridae, Furipteridae, Mormoopidae Natalidae.

Case	Family	Genus	Species	n	Mean intraspecific distance (%)	Maximum intraspecific distance (%)	Number of lineages observed	Number of lineages reported±	Reference
1	Emballonuridae	*Balantiopteryx*	*Balantiopteryx io*	14	0	0	1		
2			*Balantiopteryx plicata*	10	0.37	0.77	1		
3		*Centronycteris*	*Centronycteris maximiliani*	5	0.41	0.73	1		
4		*Cormura*	*Cormura brevirostris*	45	1.81	8.47	3		
5		*Cyttarops*	*Cyttarops alecto*	3	3.69	5.82	1		
6		*Diclidurus*	*Diclidurus isabellus*	25	0.32	0.63	1		
7		*Peropteryx*	*Peropteryx kappleri*	2	N/A	N/A	1		
8			*Peropteryx leucoptera*	3	1.06	1.6	1		
9			*Peropteryx macrotis*	9	0.23	0.46	1		
10			*Peropteryx trinitatis*	12	0.22	0.47	1		
11		*Rhynchonycteris*	*Rhynchonycteris naso*	93	0.88	2.23	1		
12		*Saccopteryx*	*Saccopteryx bilineata*	139	2.10	9.99	3	3	[29]
13			*Saccopteryx canescens*	2	N/A	N/A	1		
14			*Saccopteryx gymnura*	2	N/A	N/A	1		
15			*Saccopteryx leptura*	45	0.92	3.72	1		
16	Furipteridae	*Furipterus*	*Furipterus horrens*	4	2.48	4.64	1		
17	Mormoopidae	*Mormoops*	*Mormoops megalophylla*	5	0	0	1		
18		*Pteronotus*	*Pteronotus davyi*	10	0.09	0.31	1	3	[15], [51]
19			*Pteronotus gymnonotus*	11	0.22	0.62	1		
20			*Pteronotus parnellii*	355	5.0	12.55	4	2,4	[12], [15], [51]
21			*Pteronotus personatus*	48	2.20	10.40	5	2	[12]
22	Natalidae	*Natalus*	*Natalus stramineus*	11	0.8	2.03	1		
23			*Natalus tumidirostris*	1	N/A	N/A	N/A		

^±^Reported previously: includes both hypothesized cryptic taxa and those identified as geographic variants without species hypotheses.

Species of bats examined for DNA barcoding analysis with sample size and number of lineages previously reported. For species represented by >2 specimens, mean and maximum intraspecific sequence divergences (K2P) are reported. For species represented by >1 specimen the number of potential mitochondrial lineages is indicated.

**Table 2 pone-0022648-t002:** Noctilionidae, Molossidae.

Case	Family	Genus	Species	n	Mean intraspecific distance (%)	Maximum intraspecific distance (%)	Number of lineages observed	Number of lineages reported±	Reference
24	Noctilionidae	*Noctilio*	*Noctilio albiventris*	48	6.41	7.03	2	2,3	[4], [15], [51]
25			*Noctilio leporinus*	33	0.74	2.90	1		
26	Molossidae	*Cynomops*	*Cynomops paranus*	14	0.75	3.81	2		
27			*Cynomops planirostris*	1	N/A	N/A	N/A		
28		*Eumops*	*Eumops auripendulus*	7	0.25	0.77	1		
29			*Eumops hansae*	10	1.04	4.65	2		
30			*Eumops maurus*	1	N/A	N/A	N/A		
31		*Molossops*	*Molossops neglectus*	9	0.16	0.47	1		
32			*Molossops temminckii*	5	0.18	0.31	1		
33		*Molossus*	*Molossus coibensis*	7	0.13	0.31	1		
34			*Molossus molossus* *	138	0.51	2.22	1		
35			*Molossus rufus*	48	0.80	1.72	1		
36			*Molossus sp.*	1	N/A	N/A	N/A		
37		*Nyctinomops*	*Nyctinomops laticaudatus*	17	0.13	0.47	1		
38			*Nyctinomops macrotis*	1	N/A	N/A	N/A		
39		*Promops*	*Promops centralis*	3	0.62	0.94	1		
40		*Tadarida*	*Tadarida brasiliensis*	6	0.56	0.77	1		

^±^Reported previously: includes both hypothesized cryptic taxa and those identified as geographic variants without species hypotheses.

*bootstrap support for at least one lineage below 90.

Species of bats examined for DNA barcoding analysis with sample size and number of lineages previously reported. For species represented by >2 specimens, mean and maximum intraspecific sequence divergences (K2P) are reported. For species represented by >1 specimen the number of potential mitochondrial lineages is indicated.

**Table 3 pone-0022648-t003:** Phyllostomidae Part 1.

Case	Family	Genus	Species	n	Mean intraspecific distance (%)	Maximum intraspecific distance (%)	Number of lineages observed	Number of lineages reported±	Reference
41	Phyllostomidae	*Ametrida*	*Ametrida centurio*	137	1.21	2.57	1		
42		*Anoura*	*Anoura caudifer*	55	2.56	16.51	2		
43			*Anoura cultrate*	1	N/A	N/A	N/A		
44			*Anoura geoffroyi*	77	1.56	7.75	2		
45			*Anoura latidens*	6	0.09	0.17	1		
46		*Artibeus*	*Artibeus amplus*	32	0.46	1.08	1		
47			*Artibeus anderseni*	14	0.31	0.62	1		
48			*Artibeus aztecus*	11	0.27	0.47	1		
49			*Artibeus bogotensis*	69	0.87	2.21	1		
50			*Artibeus cinereus*	159	0.30	1.24	1		
51			*Artibeus concolor*	85	1.40	3.15	1		
52			*Artibeus fimbriatus*	3	1.03	1.40	1		
53			*Artibeus gnomus*	154	1.13	3.15	1		
54			*Artibeus intermedius* *	111	0.79	2.95	1		
55			*Artibeus jamaicensis* *	91	1.14	3.47	2	3	[53]
56			*Artibeus lituratus*	619	0.69	2.35	1		
57			*Artibeus obscurus*	531	0.60	2.36	2		
58			*Artibeus phaeotis*	60	0.20	0.79	1		
59			*Artibeus planirostris*	510	1.31	3.24	1		
60			*Artibeus toltecus*	27	0.26	1.40	1		
61			*Artibeus watsoni*	25	5.31	10.63	2		

^±^Reported previously: includes both hypothesized cryptic taxa and those identified as geographic variants without species hypotheses.

*bootstrap support for at least one lineage below 90.

Species of bats examined for DNA barcoding analysis with sample size and number of lineages previously reported. For species represented by >2 specimens, mean and maximum intraspecific sequence divergences (K2P) are reported. For species represented by >1 specimen the number of potential mitochondrial lineages is indicated.

**Table 4 pone-0022648-t004:** Phyllostomidae Part 2.

Case	Family	Genus	Species	n	Mean intraspecific distance (%)	Maximum intraspecific distance (%)	Number of lineages observed	Number of lineages reported±	Reference
62	Phyllostomidae	*Carollia*	*Carollia brevicauda* *	266	1.48	3.70	3	2	[4], [15], [17], [54]
63			*Carollia castanea*	59	3.45	6.84	3	4,3	[15], [17], [44], [54,]
64			*Carollia perspicillata*	1013	0.71	2.83	1	2	[40]
65			*Carollia sowelli*	68	0.73	3.47	2		
66			*Carollia subrufa*	23	0.23	0.93	1		
67		*Centurio*	*Centurio senex*	44	0.91	2.20	1		
68		*Chiroderma*	*Chiroderma doriae*	4	0.23	0.46	1		
69			*Chiroderma salvini*	1	N/A	N/A	N/A		
70			*Chiroderma trinitatum*	44	0.82	1.87	1		
71			*Chiroderma villosum*	55	0.94	2.19	1		
72		*Choeroniscus*	*Choeroniscus godmani*	1	N/A	N/A	N/A		
73			*Choeroniscus minor*	7	0.07	0.16	1		
74			*Choeroniscus sp.*	4	1.11	1.71	1		
75		*Chrotopterus*	*Chrotopterus auritus*	64	3.39	15.98	3	3	[29]
76		*Desmodus*	*Desmodus rotundus*	107	2.96	6.58	6	5,6	[29], [41], [42]
77		*Diaemus*	*Diaemus youngi*	4	0.33	0.46	1		
78		*Diphylla*	*Diphylla ecaudata*	3	4.32	6.48	2		
79		*Ectophylla*	*Ectophylla alba*	1	N/A	N/A	N/A		
80		*Enchisthenes*	*Enchisthenes hartii*	3	2.12	2.51	1		
81		*Glossophaga*	*Glossophaga commissarisi*	36	1.59	3.80	2		
82			*Glossophaga leachii*	9	0.03	0.15	1		
83			*Glossophaga longirostris*	38	0.57	1.08	1		
84			*Glossophaga soricina* *	196	2.67	5.95	3	2,2,3	[15], [17], [29], [40]

^±^Reported previously: includes both hypothesized cryptic taxa and those identified as geographic variants without species hypotheses.

*bootstrap support for at least one lineage below 90.

Species of bats examined for DNA barcoding analysis with sample size and number of lineages previously reported. For species represented by >2 specimens, mean and maximum intraspecific sequence divergences (K2P) are reported. For species represented by >1 specimen the number of potential mitochondrial lineages is indicated.

**Table 5 pone-0022648-t005:** Phyllostomidae Part 3 Species of bats examined for DNA barcoding analysis with sample size and number of lineages previously reported.

Case	Family	Genus	Species	n	Mean intraspecific distance (%)	Maximum intraspecific distance (%)	Number of lineages observed	Number of lineages reported±	Reference
85	Phyllostomidae	*Glyphonycteris*	*Glyphonycteris daviesi*	9	1.24	2.19	1		
86			*Glyphonycteris sylvestris*	4	1.43	2.02	1		
87		*Hylonycteris*	*Hylonycteris underwoodi*	4	4.72	9.46	2		
88		*Lampronycteris*	*Lampronycteris brachyotis*	3	0.31	0.307	1		
89		*Lichonycteris*	*Lichonycteris obscura*	2	N/A	N/A	1		
90		*Lionycteris*	*Lionycteris spurrelli*	61	1.00	2.67	1		
91		*Lonchophylla*	*Lonchophylla chocoana*	1	N/A	N/A	N/A		
92			*Lonchophylla mordax*	1	N/A	N/A	N/A		
93			*Lonchophylla robusta*	1	N/A	N/A	N/A		
94			*Lonchophylla thomasi*	152	2.57	8.16	3		
95		*Lonchorhina*	*Lonchorhina aurita*	2	N/A	N/A	1		
96			*Lonchorhina inusitata*	5	0.32	0.53	1		
97			*Lonchorhina orinocensis*	10	0.47	1.40	1		
98		*Lophostoma*	*Lophostoma brasiliense*	15	1.48	7.73	2		
99			*Lophostoma carrikeri*	11	0.67	1.24	1		
100			*Lophostoma evotis*	3	0.20	0.31	1		
101			*Lophostoma schulzi*	7	0.44	0.93	1		
102			*Lophostoma silvicolum*	152	1.67	5.48	2		
103		*Macrophyllum*	*Macrophyllum macrophyllum* *	18	2.54	4.31	4		
104		*Mesophylla*	*Mesophylla macconnelli*	38	0.72	1.57	1	2	[55]

^±^Reported previously: includes both hypothesized cryptic taxa and those identified as geographic variants without species hypotheses.

*bootstrap support for at least one lineage below 90.

For species represented by >2 specimens, mean and maximum intraspecific sequence divergences (K2P) are reported. For species represented by >1 specimen the number of potential mitochondrial lineages is indicated.

**Table 6 pone-0022648-t006:** Phyllostomidae Part 4.

Case	Family	Genus	Species	n	Mean intraspecific distance (%)	Maximum intraspecific distance (%)	Number of lineages observed	Number of lineages reported±	Reference
105	Phyllostomidae	*Micronycteris*	*Micronycteris brosseti*	4	0.15	0.31	1		
106			*Micronycteris hirsuta*	9	1.94	7.03	3		
107			*Micronycteris megalotis*	53	4.18	7.70	6	5,9	[15], [29]
108			*Micronycteris microtis*	2	N/A	N/A	1		
109			*Micronycteris minuta*	23	2.40	4.63	3	2	[15]
110		*Mimon*	*Mimon bennettii*	3	0	0	1		
111			*Mimon cozumelae*	7	0.17	0.33	1		
112			*Mimon crenulatum*	76	1.82	4.95	1		
113		*Phylloderma*	*Phylloderma stenops*	25	2.12	4.66	4	2	[4]
114		*Phyllostomus*	*Phyllostomus discolor*	75	0.46	1.43	1		
115			*Phyllostomus elongatus*	179	0.26	1.08	1		
116			*Phyllostomus hastatus*	55	1.02	4.35	2		
117			*Phyllostomus latifolius*	7	1.07	1.87	2		
118		*Platyrrhinus*	*Platyrrhinus aurarius*	45	0.35	0.99	1		
119			*Platyrrhinus brachycephalus*	3	0.93	1.40	1		
120			*Platyrrhinus helleri* *	179	2.55	5.83	4	3,3,4	[4], [12], [29]
121			*Platyrrhinus infuscus*	29	0.39	0.93	1		
122			*Platyrrhinus lineatus*	2	N/A	N/A	1		
123			*Platyrrhinus recifinus*	3	0.20	0.31	1		
124			*Platyrrhinus vittatus*	1	N/A	N/A	N/A		
125		*Rhinophylla*	*Rhinophylla alethina*	3	1.56	2.19	1		
126			*Rhinophylla fischerae*	39	0.88	2.05	1		
127			*Rhinophylla pumilio*	366	0.73	2.66	1		

^±^Reported previously: includes both hypothesized cryptic taxa and those identified as geographic variants without species hypotheses.

*bootstrap support for at least one lineage below 90.

Species of bats examined for DNA barcoding analysis with sample size and number of lineages previously reported. For species represented by >2 specimens, mean and maximum intraspecific sequence divergences (K2P) are reported. For species represented by >1 specimen the number of potential mitochondrial lineages is indicated.

**Table 7 pone-0022648-t007:** Phyllostomidae Part 5, Thyropteridae.

Case	Family	Genus	Species	n	Mean intraspecific distance (%)	Maximum intraspecific distance (%)	Number of lineages observed	Number of lineages reported±	Reference
128	Phyllostomidae	*Sturnira*	*Sturnira lilium*	245	2.91	8.87	3	3,4	[3], [40]
129			*Sturnira ludovici*	28	2.09	5.98	2		
130			*Sturnira magna*	37	0.67	1.91	1		
131			*Sturnira tildae*	162	0.39	1.71	1		
132		*Tonatia*	*Tonatia saurophila*	64	1.78	5.49	2		
133		*Trachops*	*Trachops cirrhosus* *	158	3.85	8.43	9	3,9	[4], [29]
134		*Trinycteris*	*Trinycteris nicefori*	35	2.95	7.70	2		
135		*Uroderma*	*Uroderma bilobatum* *	135	1.13	4.19	2	3,2	[29], [38]
136			*Uroderma magnirostrum*	1	N/A	N/A	N/A		
137		*Vampyressa*	*Vampyressa bidens*	138	0.80	3.31	1		
138			*Vampyressa brocki*	9	0.54	1.35	1		
139			*Vampyressa nymphaea*	8	0.52	1.24	1		
140			*Vampyressa pusilla*	7	0.57	1.08	1	2	[15], [55]
141			*Vampyressa thyone*	52	0.50	1.27	1	2	[55]
142		*Vampyrodes*	*Vampyrodes caraccioli* *	58	1.36	4.64	3		
143		*Vampyrum*	*Vampyrum spectrum*	5	0.80	1.39	1		
144	Thyropteridae	*Thyroptera*	*Thyroptera lavali*	3	1.35	1.71	1		
145			*Thyroptera tricolor*	26	6.95	14.97	3		

^±^Reported previously: includes both hypothesized cryptic taxa and those identified as geographic variants without species hypotheses.

*bootstrap support for at least one lineage below 90.

Species of bats examined for DNA barcoding analysis with sample size and number of lineages previously reported. For species represented by >2 specimens, mean and maximum intraspecific sequence divergences (K2P) are reported. For species represented by >1 specimen the number of potential mitochondrial lineages is indicated.

**Table 8:** Vespertilionidae. Species of bats examined for DNA barcoding analysis with sample size and number of lineages previously reported.

Case	Family	Genus	Species	n	Mean intraspecific distance (%)	Maximum intraspecific distance (%)	Number of lineages observed	Number of lineages reported±	Reference
146	Vespertilionidae	*Bauerus*	*Bauerus dubiaquercus*	2	N/A	N/A	1		
147		*Eptesicus*	*Eptesicus brasiliensis*	1	N/A	N/A	N/A		
148			*Eptesicus chiriquinus*	22	0.58	1.71	1		
149			*Eptesicus furinalis* *	31	3.49	6.69	3		
150		*Euderma*	*Euderma maculatum*	1	N/A	N/A	N/A		
151		*Lasiurus*	*Lasiurus atratus*	4	0.49	0.51	1		
152			*Lasiurus blossevillii*	7	8.20	13.96	2		
153			*Lasiurus egregius*	4	0.46	0.93	1		
154		*Myotis*	*Myotis albescens*	26	0.93	2.04	1		
155			*Myotis elegans*	5	0.52	0.93	1		
156			*Myotis keaysi*	27	4.36	10.12	2		
157			*Myotis nigricans*	3	0.31	0.72	1		
158			*Myotis riparius*	24	11.79	14.39	3	3	[4]
159			*Myotis ruber*	3	0	0	1		
160			*Myotis velifer*	2	N/A	N/A	1		
161		*Rhogeessa*	*Rhogeessa aeneus*	18	0.69	1.71	1		
162			*Rhogeessa io*	6	0	0	1		
163			*Rhogeessa tumida*	2	N/A	N/A	1		
Totals for all surveyed bat species (Tables 1–8)			Current species richness = 163				Total = 232		

^±^Reported previously: includes both hypothesized cryptic taxa and those identified as geographic variants without species hypotheses.

*bootstrap support for at least one lineage below 90.

For species represented by >2 specimens, mean and maximum intraspecific sequence divergences (K2P) are reported. For species represented by >1 specimen the number of potential mitochondrial lineages is indicated.

**Figure 3:**
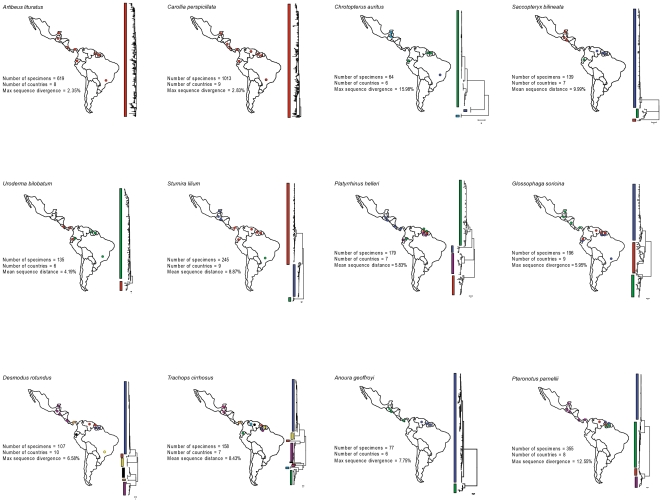
Neighbour joining trees of COI sequences demonstrating the diversity in the twelve most widely sampled bat species (n>60) in the DNA barcode dataset from Central and South America.

**Figure 4:**
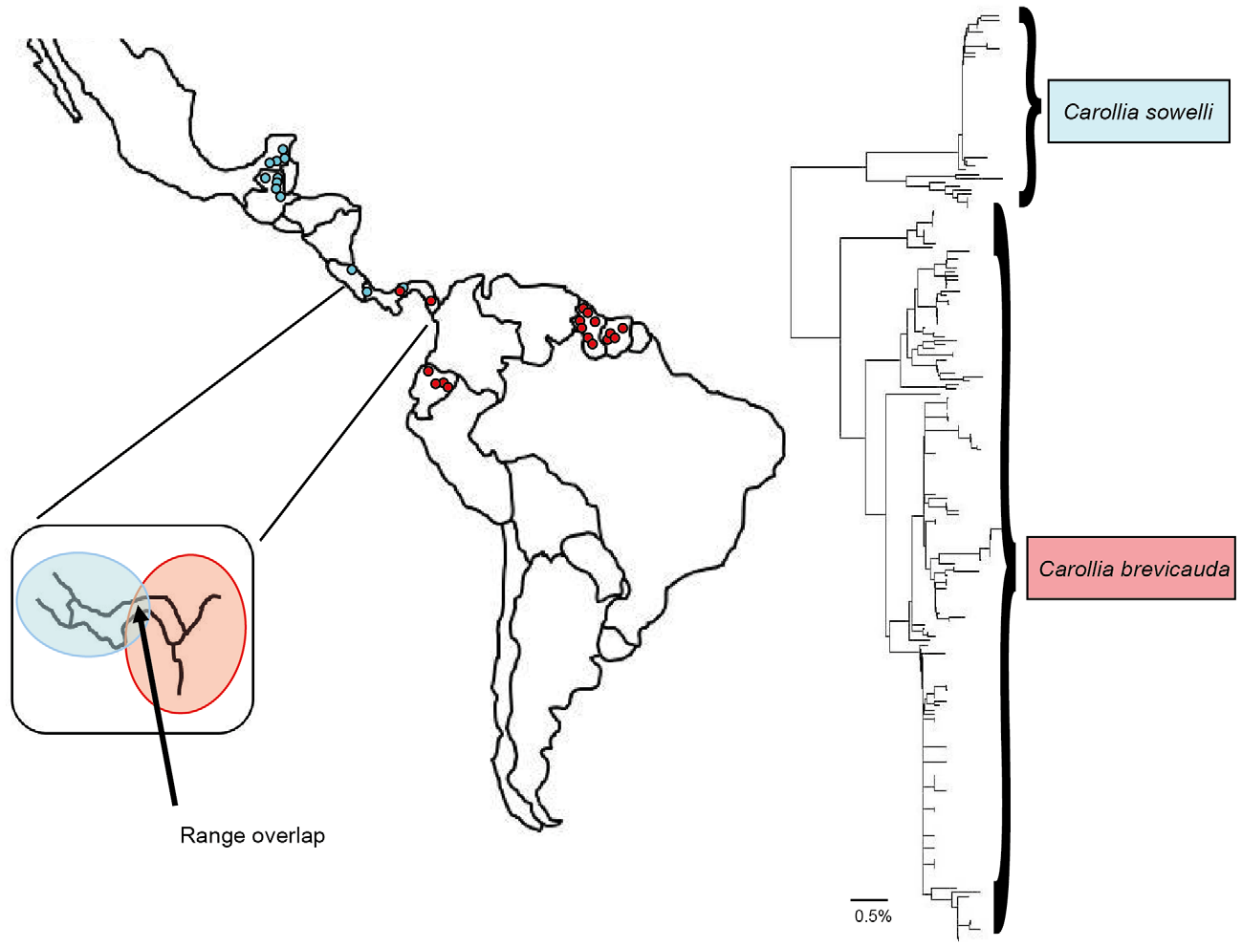
Geographic distribution and genetic diversity of COI for *Carollia brevicauda* and *C. sowelli.* *C. brevicauda* is found in eastern Panama and South America whereas *C. sowelli* occurs in other parts of Central America with a potential sympatric zone in central Panama. Intraspecific variation exists within both species including additional divergent clusters.

For twelve species our sampling was extensive with 64–1013 sequences acquired per species from 5–10 countries in both Central and South America (Figure 3). In two of these cases (*Artibeus lituratus* and *Carollia perspicillata*) no geographic structuring is evident despite sequence divergences of up to 2.35% and 2.83% respectively. In the remaining ten species substantial mitochondrial structuring was observed. In four cases (*Chrotopterus auritus*, *Saccopteryx bilineata*, *Anoura geoffroyi*, and *Sturnira lilium*) distinct mitochondrial lineages within each species appear to have allopatric distributions. In *Uroderma bilobatum*, Central and South American groups are similarly evident except for one sample from Ecuador that groups with Central America (though see [29] for a discussion of *U. bilobatum*). Within each of the remaining five species (*Platyrrhinus helleri*, *Glossophaga soricina*, *Desmodus rotundus*, *Trachops cirrhosus*, and *Pteronotus parnellii*) distinct lineages are found with both allopatric and sympatric (either in whole or in part) distributional patterns. Similarly, *C. brevicauda* and *C. sowelli*, (formerly included in *C. brevicauda* but restricted to Central America) have a potential sympatric zone in central Panama (Figure 4). In seven species (*C. auritus*, *S. bilineata*, *S. lilium*, *P. helleri*, *G. soricina, A. geoffroyi* and *P. parnellii*) the Central American specimens form a single group that is distinct from South American groups (Figure 3).

## Discussion

To our knowledge, the present study is the largest survey ever conducted of land vertebrate mtDNA diversity. Our results provide further confirmation that DNA barcoding is a powerful tool for species identification in Neotropical bats regardless of geographic scale or sample size. Only two of the 163 species examined in this study (*Artibeus intermedius* and *A. lituratus)* share haplotypes and cannot be distinguished via DNA barcoding. The remaining species are distinguishable at this locus and the resulting library of molecular data will be a powerful tool for guiding systematic research and furthering phylogeographic studies. As our sequences are all derived from vouchered specimens the reference database will also be a valuable tool for validating field collections e.g. [12] when vouchering is impractical and the discrimination of some species requires examination of morphological characters which cannot be evaluated on live specimens (e.g. cranial or dental characters). In addition, molecular tools can help to identify partial remains or trace materials from guano when capture, morphological assessment or tissue acquisition are not possible [30], [31].

### Cryptic Taxa and Estimates of Diversity

DNA barcoding campaigns seek to simplify and aid in the identification of species, and to advance species discovery by using deep intraspecific sequence divergence between mitochondrial lineages as an indication of potential new species. Methods of identifying cryptic lineages are diverse. Distance-based methods are common, particularly using strict thresholds [15], [17]. However, thresholds will not necessarily reveal recently diverged species and may inflate or deflate the species count within some genera if not accompanied by analyses of morphological, behavioral and ecological characteristics. Rate heterogeneity and variation in selective pressure on protein evolution in mitochondrial DNA likely contribute to levels of genetic divergence [32] but they also make character-based approaches [33]–[35], the 10x threshold rule [36] and other distance approaches [37], [6] unlikely to provide more accurate estimates of cryptic species.

We estimate potential taxonomic richness by visual inspection of trees for distinct lineages that are well supported (most bootstrap values ≥90) and compared these to the criteria described by Bradley and Baker [17] and Baker and Bradley [15]. Only 30 of 137 taxa represented by 3 or more samples contained >2% mean sequence divergence and would be flagged by the Bradley and Baker [17] criteria. In contrast, by visually inspecting the trees for deep, intraspecific, mitochondrial structure we found 44 cases of potential cryptic speciation. In three cases, *Furipterus horrens* (2.48% mean sequence divergence), *Enchisthenes hartii* (2.12% mean sequence divergence) and *Cyttarops alecto* (3.69% mean sequence divergence), species had divergence >2% but no distinct mitochondrial lineages or “phylogroups” as defined in Baker and Bradley [15], though in all three cases determining the pattern of intraspecific divergence is complicated by a small sample size. Maximum sequence divergence was a similarly poor predictor of mitochondrial lineages. It is also interesting to note that one of the best examples to date of cryptic diversity and the genetic species concept in bats, *Uroderma bilobatum* [38], would not have been flagged for taxonomic reassessment as it had 1.13% mean sequence divergence though internal mitochondrial structuring was obvious by visual inspection of the tree. It should however be noted, that cytochrome b evolves at a faster rate than COI [39] so the criteria developed by Bradley and Baker [17] might need to be lowered for COI, though explicit tests of rate heterogeneity have not been made here and variation in selection pressure may alter this pattern. It remains to be seen how many cases of distinct mitochondrial lineages are associated with a cessation of gene flow – an assessment that will require the analysis of nuclear loci.

Even in this relatively well-studied group, our estimates of species richness suggest as much as a 42% increase in species diversity compared to current estimates (Table 1, 2, 3, 4, 5, 6, 7, 8). Though these are rough estimates, and can change depending on how “intraspecific mitochondrial lineages” is defined, they provide a guide for future systematic research and the number of cases is likely to increase with more complete geographic sampling, particularly with the addition of specimens from the Antilles due to the influence of island isolation [40]. In particular, the monotypic genera *Desmodus* and *Trachops* may contain as many as 15 intraspecific lineages, any of which may represent cryptic species (Figure 3, Table 4, Table 7) and this observation is in accordance with the high diversity in *Desmodus* observed by Martins et al. [41], [42]. Of the 12 species with extensive geographic and individual sampling (Figure 3) six appear to contain multiple divergent lineages located within the same countries (particularly Ecuador, Guyana, and Suriname) suggesting at least partially sympatric ranges for these lineages and raising questions about modes of reproductive isolation, the role of male-mediated gene flow, and the frequency of hybridization.

Allopatric lineages can be difficult to define as they may appear allopatric due to incomplete sampling. In *Saccopteryx bilineata* (Figure 2a) our sampling suggests three distinct lineages that are strongly geographically isolated. However, no known break in the distribution of *S. bilineata* is currently recognized making it impossible to predict whether these lineages would become one hyperdiverse cluster if sampling through Central America and northern South America were increased, or whether the lineages are maintained with allopatric or symptatric distributions. The genus *Carollia* contains newly described species which were recognized genetically [43], [44]. *Carollia brevicauda* was thought to be distributed in both Central and South America until the Central American lineage was identified as distinct and revised as *C. sowelli* (Figure 4) by Baker et al. [43]. These species were reported as occupying allopatric distributions [9], but our data (Figure 4) suggests a sympatric zone in central Panama though it cannot be determined from these data whether these species hybridize or live in reproductive isolation at this location.

Previous regional assessments of bat diversity using COI [4], [12] identified a number of species which may represent complexes of undescribed taxa though these were only investigated in small geographic areas. In the continental survey conducted here, lineages proposed by Clare et al. [4] and Borisenko et al. [12] were supported by increased sampling over broader geographic areas.

### Future Research Directions

Mean sequence divergence in bats (1.38%) is substantially higher than that observed in birds (0.23%), the only other vertebrate group to have been surveyed across a continent [5]. However the birds were of North American origin so the effect of locality cannot be separated from that of taxonomy. Similarly, the proportion of distinct lineages reported here is high compared to birds [5], but not dissimilar to estimates provided for mammals by Baker and Bradley [15] and for South East Asian bats by Francis et al. [16]. Several clear research priorities exist to understand the biodiversity of Neotropical bats. First, the nature and extent of intraspecific sequence divergence must be quantified to provide an accurate measure of diversity, and this must be done in the context of selection, rates of mutation, protein evolution and the role of selective sweeps [45], [46], particularly in hyperdiverse taxa. For taxonomic assessments, additional gene regions/markers, particularly of nuclear origin, will be required to understand evolutionary patterns e.g. [29]. Directed morphological analysis of species in potential areas of diversity will also help to clarify species boundaries.

Because many bats do not rely on vision as a primary means for conspecific identification, they likely use other sensory modalities for mate recognition. Acoustic analysis of echolocation may identify the basis for intra- and interspecific recognition and potential modes of speciation [47]. Alternately, olfaction also plays a large role in habitat choice (particularly for food) and may also be utilized in intra- and interspecific recognition. For example, many of the “whispering bats” (family Phyllostomidae, widely represented in our dataset) use lower intensity echolocation calls (although see [48], [49]) but tend to be frugivorous or nectivorous species which may rely heavily on olfactory cues for both food acquisition and mate recognition. Some insectivores, such as some sac-winged bats (Emballonuridae) also rely heavily on olfaction to attract mates [50]. Alternative isolating cues in these different sensory modalities may evolve faster in species where selection drives non-visual means of inter- and intraspecific recognition. While these traits cannot be evaluated in museum specimens, they may provide a wealth of research opportunities and a method of identifying cryptic modes of assortative mating and prezygotic reproductive isolation.

## Acknowledgments

This research would not have been possible without support provided by the Biodiversity Institute of Ontario and access to the collections held by the Royal Ontario Museum (Toronto Ontario, Canada). We particularly thank Dr. Judith Eger and Dr. Mark Engstrom at the Royal Ontario Museum for facilitating collection access and Dr. Alex Borisenko, Dr. Natalia Ivanova, Agata Pawlowski and Miranda Elliott at the Biodiversity Institute of Ontario for assistance with informatics and molecular analysis. Dr. Robin Floyd and two anonymous reviewers provided excellent feedback on this manuscript.

## Supporting Information

Figure S1 A neighbour-joining tree of COI sequence divergence (K2P) in surveyed species.
(PDF)

Figure S2 Neighbour-joining trees of COI sequence divergence (K2P) in surveyed species simplified to show current species designations and cases of deeply divergent intraspecific lineages (coloured red) in need of further systematic study. For clarity, trees were generated on subsets of the total dataset. All branch supports represent boostrap values (1000 replications).
(PDF)

Table S1 GenBank and BOLD accessions for all COI sequences. Museum accessions for all vouchered specimens.
(XLS)
