# Supplementary material for: Neotropical Bats: Estimating Species Diversity with DNA Barcodes
Source: PLoS One. 2011 Jul 26;6(7):e22648. doi: 10.1371/journal.pone.0022648 (PMC3144236; doi:10.1371/journal.pone.0022648)
Supplement: Figure S1 — A neighbour-joining tree of COI sequence divergence (K2P) in surveyed species. (PDF) [file pone.0022648.s001.pdf]

BOLD TaxonID Tree

Project	:	BOLD PROCESSDS[NO CODE]
Date	:	6-June-2011
Data Type	:	Nucleotide
Distance Model	:	Kimura 2 Parameter
Codon Positions	:	1st, 2nd, 3rd
Labels	:	Country & Province, SampleID, ProcessID,
Filters	:	Length > 300
Colorization	:	[blue]=Stop Codons [red]=Contamination or misidentification

Sequence Count	:	9076
Species count	:	176
Genus count	:	65
Family count	:	9
Unidentified	:	0

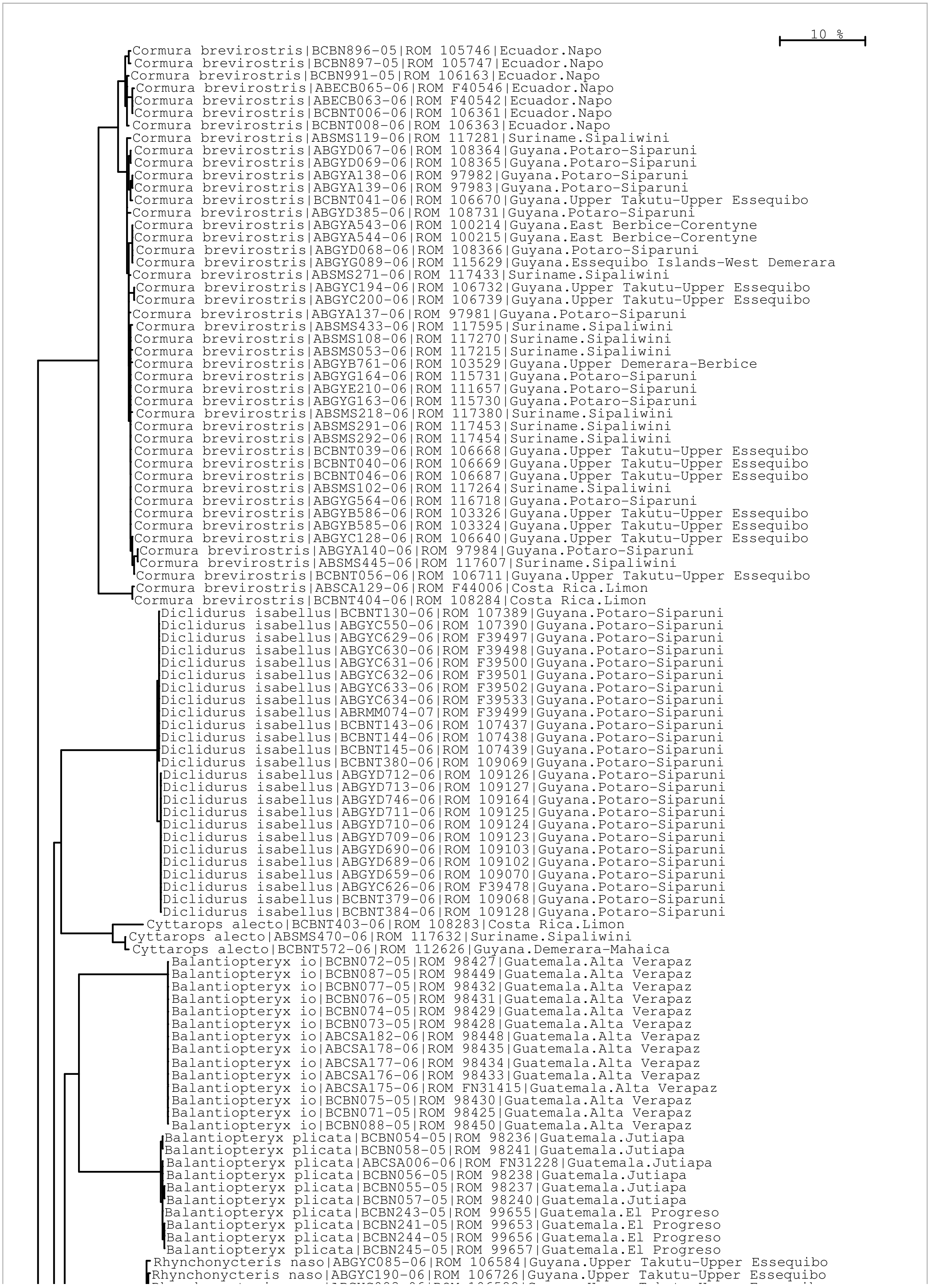
10 %
Cormura brevirostris|BCBN896-05|ROM 105746|Ecuador.Napo
Cormura brevirostris|BCBN897-05|ROM 105747|Ecuador.Napo
Cormura brevirostris|BCBN991-05|ROM 106163|Ecuador.Napo
Cormura brevirostris|ABECB065-06|ROM F40546|Ecuador.Napo
Cormura brevirostris|ABECB063-06|ROM F40542|Ecuador.Napo
Cormura brevirostris|BCBNT006-06|ROM 106361|Ecuador.Napo
Cormura brevirostris|BCBNT008-06|ROM 106363|Ecuador.Napo
Cormura brevirostris|ABSMS119-06|ROM 117281|Suriname.Sipaliwini
Cormura brevirostris|ABGYD067-06|ROM 108364|Guyana.Potaro-Siparuni
Cormura brevirostris|ABGYD069-06|ROM 108365|Guyana.Potaro-Siparuni
Cormura brevirostris|ABGYA138-06|ROM 97982|Guyana.Potaro-Siparuni
Cormura brevirostris|ABGYA139-06|ROM 97983|Guyana.Potaro-Siparuni
Cormura brevirostris|BCBNT041-06|ROM 106670|Guyana.Upper Takutu-Upper Essequibo
Cormura brevirostris|ABGYD385-06|ROM 108731|Guyana.Potaro-Siparuni
Cormura brevirostris|ABGYA543-06|ROM 100214|Guyana.East Berbice-Corentyne
Cormura brevirostris|ABGYA544-06|ROM 100215|Guyana.East Berbice-Corentyne
Cormura brevirostris|ABGYD068-06|ROM 108366|Guyana.Potaro-Siparuni
Cormura brevirostris|ABGYG089-06|ROM 115629|Guyana.Essequibo Islands-West Demerara
Cormura brevirostris|ABSMS271-06|ROM 117433|Suriname.Sipaliwini
Cormura brevirostris|ABGYC194-06|ROM 106732|Guyana.Upper Takutu-Upper Essequibo
Cormura brevirostris|ABGYC200-06|ROM 106739|Guyana.Upper Takutu-Upper Essequibo
Cormura brevirostris|ABGYA137-06|ROM 97981|Guyana.Potaro-Siparuni
Cormura brevirostris|ABSMS433-06|ROM 117595|Suriname.Sipaliwini
Cormura brevirostris|ABSMS108-06|ROM 117270|Suriname.Sipaliwini
Cormura brevirostris|ABSMS053-06|ROM 117215|Suriname.Sipaliwini
Cormura brevirostris|ABGYB761-06|ROM 103529|Guyana.Upper Demerara-Berbice
Cormura brevirostris|ABGYG164-06|ROM 115731|Guyana.Potaro-Siparuni
Cormura brevirostris|ABGYE210-06|ROM 111657|Guyana.Potaro-Siparuni
Cormura brevirostris|ABGYG163-06|ROM 115730|Guyana.Potaro-Siparuni
Cormura brevirostris|ABSMS218-06|ROM 117380|Suriname.Sipaliwini
Cormura brevirostris|ABSMS291-06|ROM 117453|Suriname.Sipaliwini
Cormura brevirostris|ABSMS292-06|ROM 117454|Suriname.Sipaliwini
Cormura brevirostris|BCBNT039-06|ROM 106668|Guyana.Upper Takutu-Upper Essequibo
Cormura brevirostris|BCBNT040-06|ROM 106669|Guyana.Upper Takutu-Upper Essequibo
Cormura brevirostris|BCBNT046-06|ROM 106687|Guyana.Upper Takutu-Upper Essequibo
Cormura brevirostris|ABSMS102-06|ROM 117264|Suriname.Sipaliwini
Cormura brevirostris|ABGYG564-06|ROM 116718|Guyana.Potaro-Siparuni
Cormura brevirostris|ABGYB586-06|ROM 103326|Guyana.Upper Takutu-Upper Essequibo
Cormura brevirostris|ABGYB585-06|ROM 103324|Guyana.Upper Takutu-Upper Essequibo
Cormura brevirostris|ABGYC128-06|ROM 106640|Guyana.Upper Takutu-Upper Essequibo
Cormura brevirostris|ABGYA140-06|ROM 97984|Guyana.Potaro-Siparuni
Cormura brevirostris|ABSMS445-06|ROM 117607|Suriname.Sipaliwini
Cormura brevirostris|BCBNT056-06|ROM 106711|Guyana.Upper Takutu-Upper Essequibo
Cormura brevirostris|ABSCA129-06|ROM F44006|Costa Rica.Limon
Cormura brevirostris|BCBNT404-06|ROM 108284|Costa Rica.Limon
Diclidurus isabellus|BCBNT130-06|ROM 107389|Guyana.Potaro-Siparuni
Diclidurus isabellus|ABGYC550-06|ROM 107390|Guyana.Potaro-Siparuni
Diclidurus isabellus|ABGYC629-06|ROM F39497|Guyana.Potaro-Siparuni
Diclidurus isabellus|ABGYC630-06|ROM F39498|Guyana.Potaro-Siparuni
Diclidurus isabellus|ABGYC631-06|ROM F39500|Guyana.Potaro-Siparuni
Diclidurus isabellus|ABGYC632-06|ROM F39501|Guyana.Potaro-Siparuni
Diclidurus isabellus|ABGYC633-06|ROM F39502|Guyana.Potaro-Siparuni
Diclidurus isabellus|ABGYC634-06|ROM F39533|Guyana.Potaro-Siparuni
Diclidurus isabellus|ABRMM074-07|ROM F39499|Guyana.Potaro-Siparuni
Diclidurus isabellus|BCBNT143-06|ROM 107437|Guyana.Potaro-Siparuni
Diclidurus isabellus|BCBNT144-06|ROM 107438|Guyana.Potaro-Siparuni
Diclidurus isabellus|BCBNT145-06|ROM 107439|Guyana.Potaro-Siparuni
Diclidurus isabellus|BCBNT380-06|ROM 109069|Guyana.Potaro-Siparuni
Diclidurus isabellus|ABGYD712-06|ROM 109126|Guyana.Potaro-Siparuni
Diclidurus isabellus|ABGYD713-06|ROM 109127|Guyana.Potaro-Siparuni
Diclidurus isabellus|ABGYD746-06|ROM 109164|Guyana.Potaro-Siparuni
Diclidurus isabellus|ABGYD711-06|ROM 109125|Guyana.Potaro-Siparuni
Diclidurus isabellus|ABGYD710-06|ROM 109124|Guyana.Potaro-Siparuni
Diclidurus isabellus|ABGYD709-06|ROM 109123|Guyana.Potaro-Siparuni
Diclidurus isabellus|ABGYD690-06|ROM 109103|Guyana.Potaro-Siparuni
Diclidurus isabellus|ABGYD689-06|ROM 109102|Guyana.Potaro-Siparuni
Diclidurus isabellus|ABGYD659-06|ROM 109070|Guyana.Potaro-Siparuni
Diclidurus isabellus|ABGYC626-06|ROM F39478|Guyana.Potaro-Siparuni
Diclidurus isabellus|BCBNT379-06|ROM 109068|Guyana.Potaro-Siparuni
Diclidurus isabellus|BCBNT384-06|ROM 109128|Guyana.Potaro-Siparuni
Cyttarops alecto|BCBNT403-06|ROM 108283|Costa Rica.Limon
Cyttarops alecto|ABSMS470-06|ROM 117632|Suriname.Sipaliwini
Cyttarops alecto|BCBNT572-06|ROM 112626|Guyana.Demerara-Mahaica
Balantiopteryx io|BCBN072-05|ROM 98427|Guatemala.Alta Verapaz
Balantiopteryx io|BCBN087-05|ROM 98449|Guatemala.Alta Verapaz
Balantiopteryx io|BCBN077-05|ROM 98432|Guatemala.Alta Verapaz
Balantiopteryx io|BCBN076-05|ROM 98431|Guatemala.Alta Verapaz
Balantiopteryx io|BCBN074-05|ROM 98429|Guatemala.Alta Verapaz
Balantiopteryx io|BCBN073-05|ROM 98428|Guatemala.Alta Verapaz
Balantiopteryx io|ABCSA182-06|ROM 98448|Guatemala.Alta Verapaz
Balantiopteryx io|ABCSA178-06|ROM 98435|Guatemala.Alta Verapaz
Balantiopteryx io|ABCSA177-06|ROM 98434|Guatemala.Alta Verapaz
Balantiopteryx io|ABCSA176-06|ROM 98433|Guatemala.Alta Verapaz
Balantiopteryx io|ABCSA175-06|ROM FN31415|Guatemala.Alta Verapaz
Balantiopteryx io|BCBN075-05|ROM 98430|Guatemala.Alta Verapaz
Balantiopteryx io|BCBN071-05|ROM 98425|Guatemala.Alta Verapaz
Balantiopteryx io|BCBN088-05|ROM 98450|Guatemala.Alta Verapaz
Balantiopteryx plicata|BCBN054-05|ROM 98236|Guatemala.Jutiapa
Balantiopteryx plicata|BCBN058-05|ROM 98241|Guatemala.Jutiapa
Balantiopteryx plicata|ABCSA006-06|ROM FN31228|Guatemala.Jutiapa
Balantiopteryx plicata|BCBN056-05|ROM 98238|Guatemala.Jutiapa
Balantiopteryx plicata|BCBN055-05|ROM 98237|Guatemala.Jutiapa
Balantiopteryx plicata|BCBN057-05|ROM 98240|Guatemala.Jutiapa
Balantiopteryx plicata|BCBN243-05|ROM 99655|Guatemala.El Progreso
Balantiopteryx plicata|BCBN241-05|ROM 99653|Guatemala.El Progreso
Balantiopteryx plicata|BCBN244-05|ROM 99656|Guatemala.El Progreso
Balantiopteryx plicata|BCBN245-05|ROM 99657|Guatemala.El Progreso
Rhynchonycteris naso|ABGYC085-06|ROM 106584|Guyana.Upper Takutu-Upper Essequibo
Rhynchonycteris naso|ABGYC190-06|ROM 106726|Guyana.Upper Takutu-Upper Essequibo

Balantiopteryx plicata|BCBN245-05|ROM 99657|Guatemala.El Progreso
Rhynchonycteris naso|ABGYC085-06|ROM 106584|Guyana.Upper Takutu-Upper Essequibo
Rhynchonycteris naso|ABGYC190-06|ROM 106726|Guyana.Upper Takutu-Upper Essequibo
Rhynchonycteris naso|ABGYC083-06|ROM 106582|Guyana.Upper Takutu-Upper Essequibo
Rhynchonycteris naso|ABGYA739-06|ROM 100444|Guyana.East Berbice-Corentyne
Rhynchonycteris naso|ABECB163-08|ROM 118841|Ecuador
Rhynchonycteris naso|ABGYE260-06|ROM 111709|Guyana.Potaro-Siparuni
Rhynchonycteris naso|ABGYG647-08|ROM 119075|Guyana
Rhynchonycteris naso|BCBN849-05|ROM 105537|Ecuador.Napo
Rhynchonycteris naso|BCBNT191-06|ROM 107891|Venezuela.Amazonas
Rhynchonycteris naso|ABGYD730-06|ROM 109146|Guyana.Potaro-Siparuni
Rhynchonycteris naso|ABGYD784-06|ROM 109205|Guyana.Potaro-Siparuni
Rhynchonycteris naso|ABGYC125-06|ROM 106637|Guyana.Upper Takutu-Upper Essequibo
Rhynchonycteris naso|ABGYA224-06|ROM 98095|Guyana.Upper Takutu-Upper Essequibo
Rhynchonycteris naso|ABGYC100-06|ROM 106607|Guyana.Upper Takutu-Upper Essequibo
Rhynchonycteris naso|ABGYC178-06|ROM 106712|Guyana.Upper Takutu-Upper Essequibo
Rhynchonycteris naso|ABGYE167-06|ROM 111610|Guyana.Potaro-Siparuni
Rhynchonycteris naso|ABGYE694-06|ROM 112624|Guyana.Demerara-Mahaica
Rhynchonycteris naso|ABGYE710-06|ROM 112645|Guyana.Demerara-Mahaica
Rhynchonycteris naso|ABGYC192-06|ROM 106730|Guyana.Upper Takutu-Upper Essequibo
Rhynchonycteris naso|ABGYC193-06|ROM 106731|Guyana.Upper Takutu-Upper Essequibo
Rhynchonycteris naso|ABSRA505-06|ROM 117095|Suriname.Nickerie
Rhynchonycteris naso|ABGYA160-06|ROM 98016|Guyana.Potaro-Siparuni
Rhynchonycteris naso|ABGYC560-06|ROM 107404|Guyana.Potaro-Siparuni
Rhynchonycteris naso|ABGYA222-06|ROM 98093|Guyana.Upper Takutu-Upper Essequibo
Rhynchonycteris naso|ABGYD785-06|ROM 109206|Guyana.Potaro-Siparuni
Rhynchonycteris naso|ABSMS523-06|ROM 117685|Suriname.Sipaliwini
Rhynchonycteris naso|ABGYC084-06|ROM 106583|Guyana.Upper Takutu-Upper Essequibo
Rhynchonycteris naso|ABGYG611-08|ROM 119039|Guyana
Rhynchonycteris naso|ABSRA516-06|ROM 117106|Suriname.Nickerie
Rhynchonycteris naso|ABGYC065-06|ROM 106552|Guyana.Upper Takutu-Upper Essequibo
Rhynchonycteris naso|ABGYG701-08|ROM 119129|Guyana
Rhynchonycteris naso|ABGYC126-06|ROM 106638|Guyana.Upper Takutu-Upper Essequibo
Rhynchonycteris naso|ABGYC531-06|ROM 107370|Guyana.Potaro-Siparuni
Rhynchonycteris naso|ABGYG120-06|ROM 115672|Guyana.Potaro-Siparuni
Rhynchonycteris naso|ABGYG610-08|ROM 119038|Guyana
Rhynchonycteris naso|ABGYE125-06|ROM 111568|Guyana.Potaro-Siparuni
Rhynchonycteris naso|ABGYD798-06|ROM 109220|Guyana.Potaro-Siparuni
Rhynchonycteris naso|ABGYD657-06|ROM 109066|Guyana.Potaro-Siparuni
Rhynchonycteris naso|ABGYA103-06|ROM 97939|Guyana.Upper Takutu-Upper Essequibo
Rhynchonycteris naso|ABGYF209-06|ROM 113779|Guyana.Demerara-Mahaica
Rhynchonycteris naso|ABGYC164-06|ROM 106688|Guyana.Upper Takutu-Upper Essequibo
Rhynchonycteris naso|ABGYE688-06|ROM 112618|Guyana.Demerara-Mahaica
Rhynchonycteris naso|ABSMS001-06|ROM 117163|Suriname.Sipaliwini
Rhynchonycteris naso|ABSMS432-06|ROM 117594|Suriname.Sipaliwini
Rhynchonycteris naso|BCBNT353-06|ROM 108965|Guyana.Potaro-Siparuni
Rhynchonycteris naso|BCBNT356-06|ROM 108977|Guyana.Potaro-Siparuni
Rhynchonycteris naso|ABGYG166-06|ROM 115733|Guyana.Potaro-Siparuni
Rhynchonycteris naso|BCBNT347-06|ROM 108952|Guyana.Potaro-Siparuni
Rhynchonycteris naso|ABGYD736-06|ROM 109152|Guyana.Potaro-Siparuni
Rhynchonycteris naso|ABGYA031-06|ROM 97821|Guyana.Upper Takutu-Upper Essequibo
Rhynchonycteris naso|ABGYC703-06|ROM 108102|Guyana.Cuyuni-Mazaruni
Rhynchonycteris naso|ABGYC705-06|ROM 108104|Guyana.Cuyuni-Mazaruni
Rhynchonycteris naso|ABGYC314-06|ROM 107125|Guyana.Potaro-Siparuni
Rhynchonycteris naso|ABGYC441-06|ROM 107265|Guyana.Potaro-Siparuni
Rhynchonycteris naso|ABGYA392-06|ROM 98796|Guyana.Barima-Waini
Rhynchonycteris naso|ABGYD610-06|ROM 108978|Guyana.Potaro-Siparuni
Rhynchonycteris naso|ABGYD789-06|ROM 109211|Guyana.Potaro-Siparuni
Rhynchonycteris naso|BCBNT355-06|ROM 108976|Guyana.Potaro-Siparuni
Rhynchonycteris naso|ABGYE692-06|ROM 112622|Guyana.Demerara-Mahaica
Rhynchonycteris naso|ABGYE661-06|ROM 112581|Guyana.Demerara-Mahaica
Rhynchonycteris naso|ABGYE693-06|ROM 112623|Guyana.Demerara-Mahaica
Rhynchonycteris naso|ABGYA368-06|ROM 98769|Guyana.Barima-Waini
Rhynchonycteris naso|ABGYA369-06|ROM 98770|Guyana.Barima-Waini
Rhynchonycteris naso|ABGYC191-06|ROM 106729|Guyana.Upper Takutu-Upper Essequibo
Rhynchonycteris naso|ABSMS002-06|ROM 117164|Suriname.Sipaliwini
Rhynchonycteris naso|ABGYD601-06|ROM 108966|Guyana.Potaro-Siparuni
Rhynchonycteris naso|ABGYD611-06|ROM 108979|Guyana.Potaro-Siparuni
Rhynchonycteris naso|ABGYE152-06|ROM 111595|Guyana.Potaro-Siparuni
Rhynchonycteris naso|ABSRA330-06|ROM 116919|Suriname.Nickerie
Rhynchonycteris naso|ABGYF208-06|ROM 113778|Guyana.Demerara-Mahaica
Rhynchonycteris naso|ABSMS438-06|ROM 117600|Suriname.Sipaliwini
Rhynchonycteris naso|ABSMS516-06|ROM 117678|Suriname.Sipaliwini
Rhynchonycteris naso|ABSMS003-06|ROM 117165|Suriname.Sipaliwini
Rhynchonycteris naso|ABSMS004-06|ROM 117166|Suriname.Sipaliwini
Rhynchonycteris naso|ABGYE662-06|ROM 112582|Guyana.Demerara-Mahaica
Rhynchonycteris naso|ABGYG165-06|ROM 115732|Guyana.Potaro-Siparuni
Rhynchonycteris naso|ABGYE284-06|ROM 111733|Guyana.Potaro-Siparuni
Rhynchonycteris naso|ABGYE124-06|ROM 111567|Guyana.Potaro-Siparuni
Rhynchonycteris naso|ABGYD763-06|ROM 109183|Guyana.Potaro-Siparuni
Rhynchonycteris naso|ABGYC704-06|ROM 108103|Guyana.Cuyuni-Mazaruni
Rhynchonycteris naso|ABGYC702-06|ROM F39757|Guyana.Cuyuni-Mazaruni
Rhynchonycteris naso|ABGYC700-06|ROM 108100|Guyana.Cuyuni-Mazaruni
Rhynchonycteris naso|ABGYC699-06|ROM 108099|Guyana.Cuyuni-Mazaruni
Rhynchonycteris naso|ABGYC470-06|ROM 107302|Guyana.Potaro-Siparuni
Rhynchonycteris naso|ABGYA740-06|ROM 100445|Guyana.East Berbice-Corentyne
Rhynchonycteris naso|ABGYA223-06|ROM 98094|Guyana.Upper Takutu-Upper Essequibo
Rhynchonycteris naso|ABGYG665-08|ROM 119093|Guyana
Rhynchonycteris naso|ABGYC440-06|ROM 107264|Guyana.Potaro-Siparuni
Rhynchonycteris naso|ABSRA298-06|ROM 116887|Suriname.Nickerie
Rhynchonycteris naso|ABSMS422-06|ROM 117584|Suriname.Sipaliwini
Rhynchonycteris naso|ABSRA329-06|ROM 116918|Suriname.Nickerie
Rhynchonycteris naso|ABSRA299-06|ROM 116888|Suriname.Nickerie
Rhynchonycteris naso|BCBNT357-06|ROM 108980|Guyana.Potaro-Siparuni
Peropteryx kappleri|BCBN332-05|ROM 100910|Guyana.Barima-Waini
Peropteryx kappleri|BCBN365-05|ROM 101123|Guyana.Barima-Waini
Peropteryx macrotis|ABMXC087-06|ROM 95945|Mexico.Yucatan
Peropteryx macrotis|ABMXC089-06|ROM FN29738|Mexico.Yucatan
Peropteryx macrotis|BCBN039-05|ROM 96443|Mexico.Yucatan
Peropteryx macrotis|ABMXA934-06|ROM FN33843|Mexico.Quintana Roo
Peropteryx macrotis|ABMXC088-06|ROM 95946|Mexico.Yucatan
Peropteryx macrotis|BCBN038-05|ROM 96442|Mexico.Yucatan
Peropteryx macrotis|BCBN041-05|ROM 96445|Mexico.Yucatan

Peropteryx macrotis|ABMXC088-06|ROM 95946|Mexico.Yucatan
Peropteryx macrotis|BCBN038-05|ROM 96442|Mexico.Yucatan
Peropteryx macrotis|BCBN041-05|ROM 96445|Mexico.Yucatan
Peropteryx macrotis|BCBN040-05|ROM 96444|Mexico.Yucatan
Peropteryx macrotis|BCBN042-05|ROM 96446|Mexico.Yucatan
Peropteryx trinitatis|BCBNT212-06|ROM 107922|Venezuela.Bolivar
Peropteryx trinitatis|ABSA034-06|ROM 107916|Venezuela.Bolivar
Peropteryx trinitatis|BCBNT209-06|ROM 107918|Venezuela.Bolivar
Peropteryx trinitatis|ABSA002-06|ROM 107830|Venezuela
Peropteryx trinitatis|BCBNT159-06|ROM 107822|Venezuela.Amazonas
Peropteryx trinitatis|BCBNT162-06|ROM 107825|Venezuela.Amazonas
Peropteryx trinitatis|BCBNT163-06|ROM 107831|Venezuela.Amazonas
Peropteryx trinitatis|BCBNT160-06|ROM 107823|Venezuela.Amazonas
Peropteryx trinitatis|BCBNT161-06|ROM 107824|Venezuela.Amazonas
Peropteryx trinitatis|BCBNT210-06|ROM 107920|Venezuela.Bolivar
Peropteryx trinitatis|BCBNT211-06|ROM 107921|Venezuela.Bolivar
Peropteryx trinitatis|BCBNT213-06|ROM 107923|Venezuela.Bolivar
Peropteryx leucoptera|BCBNT551-06|ROM 112531|Guyana.Demerara-Mahaica
Peropteryx leucoptera|BCBNT550-06|ROM 112530|Guyana.Demerara-Mahaica
Peropteryx leucoptera|BCBNT646-06|ROM 113611|Guyana.Demerara-Mahaica
Centronycteris maximiliani|ABSMS076-06|ROM 117238|Suriname.Sipaliwini
Centronycteris maximiliani|ABSMS381-06|ROM 117543|Suriname.Sipaliwini
Centronycteris maximiliani|ABGYC624-06|ROM F39343|Guyana.Potaro-Siparuni
Centronycteris maximiliani|ABSMS134-06|ROM 117296|Suriname.Sipaliwini
Centronycteris maximiliani|ABSMS484-06|ROM 117646|Suriname.Sipaliwini
Saccopteryx canescens|BCBN315-05|ROM 100446|Guyana.East Berbice-Corentyne
Saccopteryx canescens|BCBNT190-06|ROM 107890|Venezuela.Amazonas
Saccopteryx gymnura|ABSMS401-06|ROM 117563|Suriname.Sipaliwini
Saccopteryx gymnura|BCBN523-05|ROM 102952|Guyana.Upper Takutu-Upper Essequibo
Saccopteryx bilineata|BCBN195-05|ROM 99322|Guatemala.Peten
Saccopteryx bilineata|BCBN196-05|ROM 99323|Guatemala.Peten
Saccopteryx bilineata|BCBN421-05|ROM 101336|El Salvador.Ahuachapan
Saccopteryx bilineata|BCBN422-05|ROM 101337|El Salvador.Ahuachapan
Saccopteryx bilineata|BCBNC222-06|ROM 96376|Mexico.Campeche
Saccopteryx bilineata|BCBN871-05|ROM 105672|Ecuador.Napo
Saccopteryx bilineata|ABECB061-06|ROM F40539|Ecuador.Napo
Saccopteryx bilineata|ABECA453-06|ROM 105363|Ecuador.Napo
Saccopteryx bilineata|ABECA653-06|ROM 105708|Ecuador.Napo
Saccopteryx bilineata|ABECA498-06|ROM 105523|Ecuador.Napo
Saccopteryx bilineata|BCBN795-05|ROM 105206|Ecuador.Napo
Saccopteryx bilineata|ABECA452-06|ROM 105362|Ecuador.Napo
Saccopteryx bilineata|ABECB062-06|ROM 106360|Ecuador.Napo
Saccopteryx bilineata|ABECB060-06|ROM F40538|Ecuador.Napo
Saccopteryx bilineata|BCBN972-05|ROM 106016|Ecuador.Napo
Saccopteryx bilineata|BCBN840-05|ROM 105522|Ecuador.Napo
Saccopteryx bilineata|ABECA412-06|ROM F37639|Ecuador.Napo
Saccopteryx bilineata|BCBNT005-06|ROM 106359|Ecuador.Napo
Saccopteryx bilineata|ABGYB719-06|ROM 103485|Guyana.Upper Demerara-Berbice
Saccopteryx bilineata|BCBNT566-06|ROM 112597|Guyana.Demerara-Mahaica
Saccopteryx bilineata|ABSMS054-06|ROM 117216|Suriname.Sipaliwini
Saccopteryx bilineata|ABSMS359-06|ROM 117521|Suriname.Sipaliwini
Saccopteryx bilineata|ABSMS404-06|ROM 117566|Suriname.Sipaliwini
Saccopteryx bilineata|ABGYG044-06|ROM 115554|Guyana.Essequibo Islands-West Demerara
Saccopteryx bilineata|ABSMS082-06|ROM 117244|Suriname.Sipaliwini
Saccopteryx bilineata|ABSMS283-06|ROM 117445|Suriname.Sipaliwini
Saccopteryx bilineata|ABSMS515-06|ROM 117677|Suriname.Sipaliwini
Saccopteryx bilineata|BCBNT768-06|ROM 113980|Suriname.Brokopondo
Saccopteryx bilineata|ABGYC105-06|ROM 106613|Guyana.Upper Takutu-Upper Essequibo
Saccopteryx bilineata|ABGYE706-06|ROM 112640|Guyana.Demerara-Mahaica
Saccopteryx bilineata|ABGYA030-06|ROM 97820|Guyana.Upper Takutu-Upper Essequibo
Saccopteryx bilineata|ABGYA104-06|ROM 97940|Guyana.Upper Takutu-Upper Essequibo
Saccopteryx bilineata|BCBNT208-06|ROM 107914|Venezuela.Bolivar
Saccopteryx bilineata|ABGYE673-06|ROM 112598|Guyana.Demerara-Mahaica
Saccopteryx bilineata|ABGYA618-06|ROM 100308|Guyana.East Berbice-Corentyne
Saccopteryx bilineata|ABGYB704-06|ROM 103468|Guyana.Upper Demerara-Berbice
Saccopteryx bilineata|ABGYA602-06|ROM 100289|Guyana.East Berbice-Corentyne
Saccopteryx bilineata|ABGYB481-06|ROM 103216|Guyana.Upper Takutu-Upper Essequibo
Saccopteryx bilineata|ABGYE590-06|ROM 112069|Guyana.Potaro-Siparuni
Saccopteryx bilineata|ABGYE721-06|ROM 112656|Guyana.Demerara-Mahaica
Saccopteryx bilineata|ABGYF151-06|ROM 113704|Guyana.Demerara-Mahaica
Saccopteryx bilineata|ABGYF183-06|ROM 113744|Guyana.Demerara-Mahaica
Saccopteryx bilineata|ABGYG052-06|ROM 115562|Guyana.Essequibo Islands-West Demerara
Saccopteryx bilineata|ABGYG145-06|ROM 115702|Guyana.Potaro-Siparuni
Saccopteryx bilineata|ABSMS118-06|ROM 117280|Suriname.Sipaliwini
Saccopteryx bilineata|ABSMS174-06|ROM 117336|Suriname.Sipaliwini
Saccopteryx bilineata|BCBNT565-06|ROM 112596|Guyana.Demerara-Mahaica
Saccopteryx bilineata|ABGYB521-06|ROM 103256|Guyana.Upper Takutu-Upper Essequibo
Saccopteryx bilineata|ABSMS080-06|ROM 117242|Suriname.Sipaliwini
Saccopteryx bilineata|ABGYD231-06|ROM 108563|Guyana.Potaro-Siparuni
Saccopteryx bilineata|ABSMS424-06|ROM 117586|Suriname.Sipaliwini
Saccopteryx bilineata|ABSMS483-06|ROM 117645|Suriname.Sipaliwini
Saccopteryx bilineata|ABGYB642-06|ROM 103398|Guyana.Upper Demerara-Berbice
Saccopteryx bilineata|ABGYD195-06|ROM 108522|Guyana.Potaro-Siparuni
Saccopteryx bilineata|ABGYG043-06|ROM 115553|Guyana.Essequibo Islands-West Demerara
Saccopteryx bilineata|ABGYG070-06|ROM 115595|Guyana.Essequibo Islands-West Demerara
Saccopteryx bilineata|ABGYG074-06|ROM 115600|Guyana.Essequibo Islands-West Demerara
Saccopteryx bilineata|BCBNT708-06|ROM 113877|Suriname.Brokopondo
Saccopteryx bilineata|ABSMS521-06|ROM 117683|Suriname.Sipaliwini
Saccopteryx bilineata|ABGYC469-06|ROM 107301|Guyana.Potaro-Siparuni
Saccopteryx bilineata|ABGYC602-06|ROM 107459|Guyana.Potaro-Siparuni
Saccopteryx bilineata|ABSMS183-06|ROM 117345|Suriname.Sipaliwini
Saccopteryx bilineata|ABSMS360-06|ROM 117522|Suriname.Sipaliwini
Saccopteryx bilineata|ABSMS489-06|ROM 117651|Suriname.Sipaliwini
Saccopteryx bilineata|ABGYG863-08|ROM 119291|Guyana
Saccopteryx bilineata|ABGYG902-08|ROM 119330|Guyana
Saccopteryx bilineata|ABSMS457-06|ROM 117619|Suriname.Sipaliwini
Saccopteryx bilineata|ABGYF197-06|ROM 113763|Guyana.Demerara-Mahaica
Saccopteryx bilineata|ABGYG107-06|ROM 115656|Guyana.Potaro-Siparuni
Saccopteryx bilineata|ABGYA052-06|ROM 97855|Guyana.Upper Takutu-Upper Essequibo
Saccopteryx bilineata|ABGYB641-06|ROM 103397|Guyana.Upper Demerara-Berbice
Saccopteryx bilineata|ABGYB643-06|ROM 103399|Guyana.Upper Demerara-Berbice
Saccopteryx bilineata|ABSMS273-06|ROM 117435|Suriname.Sipaliwini
Saccopteryx bilineata|ABGYB716-06|ROM 103481|Guyana.Upper Demerara-Berbice

Saccopteryx bilineata|ABGYB643-06|ROM 103399|Guyana.Upper Demerara-Berbice
Saccopteryx bilineata|ABSMS273-06|ROM 117435|Suriname.Sipaliwini
Saccopteryx bilineata|ABGYB716-06|ROM 103481|Guyana.Upper Demerara-Berbice
Saccopteryx bilineata|ABSMS366-06|ROM 117528|Suriname.Sipaliwini
Saccopteryx bilineata|ABGYE674-06|ROM 112599|Guyana.Demerara-Mahaica
Saccopteryx bilineata|ABGYD838-06|ROM 109264|Guyana.Potaro-Siparuni
Saccopteryx bilineata|ABGYB748-06|ROM 103515|Guyana.Upper Demerara-Berbice
Saccopteryx bilineata|ABGYD423-06|ROM 108773|Guyana.Potaro-Siparuni
Saccopteryx bilineata|ABGYE669-06|ROM 112591|Guyana.Demerara-Mahaica
Saccopteryx bilineata|ABGYD095-06|ROM 108390|Guyana.Potaro-Siparuni
Saccopteryx bilineata|ABGYE505-06|ROM 111977|Guyana.Potaro-Siparuni
Saccopteryx bilineata|ABSMS104-06|ROM 117266|Suriname.Sipaliwini
Saccopteryx bilineata|ABSMS368-06|ROM 117530|Suriname.Sipaliwini
Saccopteryx bilineata|ABSMS157-06|ROM 117319|Suriname.Sipaliwini
Saccopteryx bilineata|BCBNT722-06|ROM 113893|Suriname.Brokopondo
Saccopteryx bilineata|ABGYE567-06|ROM 112042|Guyana.Potaro-Siparuni
Saccopteryx bilineata|ABGYF091-06|ROM 113623|Guyana.Demerara-Mahaica
Saccopteryx bilineata|ABGYG062-06|ROM 115582|Guyana.Essequibo Islands-West Demerara
Saccopteryx bilineata|ABGYG063-06|ROM 115583|Guyana.Essequibo Islands-West Demerara
Saccopteryx bilineata|ABGYG069-06|ROM 115594|Guyana.Essequibo Islands-West Demerara
Saccopteryx bilineata|ABGYG073-06|ROM 115599|Guyana.Essequibo Islands-West Demerara
Saccopteryx bilineata|ABGYG080-06|ROM 115613|Guyana.Essequibo Islands-West Demerara
Saccopteryx bilineata|ABGYG081-06|ROM 115614|Guyana.Essequibo Islands-West Demerara
Saccopteryx bilineata|ABGYG090-06|ROM 115630|Guyana.Essequibo Islands-West Demerara
Saccopteryx bilineata|ABGYG092-06|ROM 115633|Guyana.Essequibo Islands-West Demerara
Saccopteryx bilineata|ABGYG861-08|ROM 119289|Guyana
Saccopteryx bilineata|ABGYG862-08|ROM 119290|Guyana
Saccopteryx bilineata|ABGYG864-08|ROM 119292|Guyana
Saccopteryx bilineata|ABGYG901-08|ROM 119329|Guyana
Saccopteryx bilineata|ABSMS012-06|ROM 117174|Suriname.Sipaliwini
Saccopteryx bilineata|ABSMS077-06|ROM 117239|Suriname.Sipaliwini
Saccopteryx bilineata|ABSMS081-06|ROM 117243|Suriname.Sipaliwini
Saccopteryx bilineata|ABSMS111-06|ROM 117273|Suriname.Sipaliwini
Saccopteryx bilineata|ABSMS124-06|ROM 117286|Suriname.Sipaliwini
Saccopteryx bilineata|ABSMS133-06|ROM 117295|Suriname.Sipaliwini
Saccopteryx bilineata|ABSMS153-06|ROM 117315|Suriname.Sipaliwini
Saccopteryx bilineata|ABSMS154-06|ROM 117316|Suriname.Sipaliwini
Saccopteryx bilineata|ABSMS155-06|ROM 117317|Suriname.Sipaliwini
Saccopteryx bilineata|ABSMS158-06|ROM 117320|Suriname.Sipaliwini
Saccopteryx bilineata|ABSMS284-06|ROM 117446|Suriname.Sipaliwini
Saccopteryx bilineata|ABSMS367-06|ROM 117529|Suriname.Sipaliwini
Saccopteryx bilineata|BCBNT577-06|ROM 112662|Guyana.Demerara-Mahaica
Saccopteryx bilineata|BCBNT578-06|ROM 112663|Guyana.Demerara-Mahaica
Saccopteryx bilineata|ABGYE504-06|ROM 111976|Guyana.Potaro-Siparuni
Saccopteryx bilineata|ABGYE524-06|ROM 111996|Guyana.Potaro-Siparuni
Saccopteryx bilineata|ABGYD230-06|ROM 108562|Guyana.Potaro-Siparuni
Saccopteryx bilineata|ABGYD232-06|ROM 108564|Guyana.Potaro-Siparuni
Saccopteryx bilineata|ABGYD094-06|ROM 108392|Guyana.Potaro-Siparuni
Saccopteryx bilineata|ABGYD120-06|ROM 108418|Guyana.Potaro-Siparuni
Saccopteryx bilineata|ABSMS437-06|ROM 117599|Suriname.Sipaliwini
Saccopteryx bilineata|ABSMS458-06|ROM 117620|Suriname.Sipaliwini
Saccopteryx bilineata|ABSMS248-06|ROM 117410|Suriname.Sipaliwini
Saccopteryx bilineata|ABSMS274-06|ROM 117436|Suriname.Sipaliwini
Saccopteryx bilineata|ABGYD093-06|ROM 108391|Guyana.Potaro-Siparuni
Saccopteryx bilineata|ABGYC452-06|ROM 107277|Guyana.Potaro-Siparuni
Saccopteryx bilineata|ABGYC401-06|ROM 107220|Guyana.Potaro-Siparuni
Saccopteryx bilineata|ABGYC127-06|ROM 106639|Guyana.Upper Takutu-Upper Essequibo
Saccopteryx bilineata|ABGYB898-06|ROM 104743|Guyana.Potaro-Siparuni
Saccopteryx bilineata|ABGYB778-06|ROM 103546|Guyana.Upper Demerara-Berbice
Saccopteryx bilineata|ABGYB598-06|ROM 103344|Guyana.Upper Takutu-Upper Essequibo
Saccopteryx bilineata|ABGYB567-06|ROM 103303|Guyana.Upper Takutu-Upper Essequibo
Saccopteryx bilineata|ABGYB520-06|ROM 103255|Guyana.Upper Takutu-Upper Essequibo
Saccopteryx bilineata|ABGYA189-06|ROM 98057|Guyana.Potaro-Siparuni
Saccopteryx bilineata|ABGYE720-06|ROM 112655|Guyana.Demerara-Mahaica
Saccopteryx bilineata|ABGYA088-06|ROM 97921|Guyana.Upper Takutu-Upper Essequibo
Saccopteryx bilineata|ABSMS397-06|ROM 117559|Suriname.Sipaliwini
Saccopteryx bilineata|BCBNT821-06|ROM 114138|Suriname.Brokopondo
Saccopteryx bilineata|BCBNT822-06|ROM 114141|Suriname.Brokopondo
Saccopteryx leptura|BCBN612-05|ROM 104208|Panama.Canal Zone
Saccopteryx leptura|ABSMS249-06|ROM 117411|Suriname.Sipaliwini
Saccopteryx leptura|ABSMS399-06|ROM 117561|Suriname.Sipaliwini
Saccopteryx leptura|BCBNT579-06|ROM 112664|Guyana.Demerara-Mahaica
Saccopteryx leptura|ABSMS078-06|ROM 117240|Suriname.Sipaliwini
Saccopteryx leptura|ABSMS079-06|ROM 117241|Suriname.Sipaliwini
Saccopteryx leptura|ABSMS294-06|ROM 117456|Suriname.Sipaliwini
Saccopteryx leptura|ABGYG072-06|ROM 115598|Guyana.Essequibo Islands-West Demerara
Saccopteryx leptura|BCBN285-05|ROM 100227|Guyana.East Berbice-Corentyne
Saccopteryx leptura|ABSMS250-06|ROM 117412|Suriname.Sipaliwini
Saccopteryx leptura|ABGYA087-06|ROM 97920|Guyana.Upper Takutu-Upper Essequibo
Saccopteryx leptura|ABSMS456-06|ROM 117618|Suriname.Sipaliwini
Saccopteryx leptura|ABSMS400-06|ROM 117562|Suriname.Sipaliwini
Saccopteryx leptura|ABSMS513-06|ROM 117675|Suriname.Sipaliwini
Saccopteryx leptura|ABSMS103-06|ROM 117265|Suriname.Sipaliwini
Saccopteryx leptura|ABSMS207-06|ROM 117369|Suriname.Sipaliwini
Saccopteryx leptura|ABSMS110-06|ROM 117272|Suriname.Sipaliwini
Saccopteryx leptura|ABSMS329-06|ROM 117491|Suriname.Sipaliwini
Saccopteryx leptura|ABSMS403-06|ROM 117565|Suriname.Sipaliwini
Saccopteryx leptura|ABSMS159-06|ROM 117321|Suriname.Sipaliwini
Saccopteryx leptura|ABGYC077-06|ROM 106574|Guyana.Upper Takutu-Upper Essequibo
Saccopteryx leptura|ABGYG082-06|ROM 115615|Guyana.Essequibo Islands-West Demerara
Saccopteryx leptura|ABSMS013-06|ROM 117175|Suriname.Sipaliwini
Saccopteryx leptura|ABSMS055-06|ROM 117217|Suriname.Sipaliwini
Saccopteryx leptura|ABSMS056-06|ROM 117218|Suriname.Sipaliwini
Saccopteryx leptura|ABSMS165-06|ROM 117327|Suriname.Sipaliwini
Saccopteryx leptura|ABSMS326-06|ROM 117488|Suriname.Sipaliwini
Saccopteryx leptura|ABSMS490-06|ROM 117652|Suriname.Sipaliwini
Saccopteryx leptura|ABSMS109-06|ROM 117271|Suriname.Sipaliwini
Saccopteryx leptura|BCBNT650-06|ROM 113624|Guyana.Demerara-Mahaica
Saccopteryx leptura|ABSMS173-06|ROM 117335|Suriname.Sipaliwini
Saccopteryx leptura|ABSMS512-06|ROM 117674|Suriname.Sipaliwini
Saccopteryx leptura|ABSMS206-06|ROM 117368|Suriname.Sipaliwini
Saccopteryx leptura|BCBNT852-06|ROM 114223|Suriname.Brokopondo
Saccopteryx leptura|ABSMS514-06|ROM 117676|Suriname.Sipaliwini

Saccopteryx leptura|[illegible]|Suriname.Sipaliwini
Saccopteryx leptura|ABSMS206-06|ROM 117368|Suriname.Sipaliwini
Saccopteryx leptura|BCBNT852-06|ROM 114223|Suriname.Brokopondo
Saccopteryx leptura|ABSMS514-06|ROM 117676|Suriname.Sipaliwini
Saccopteryx leptura|ABSMS117-06|ROM 117279|Suriname.Sipaliwini
Saccopteryx leptura|ABSMS164-06|ROM 117326|Suriname.Sipaliwini
Saccopteryx leptura|ABSMS272-06|ROM 117434|Suriname.Sipaliwini
Saccopteryx leptura|ABSMS522-06|ROM 117684|Suriname.Sipaliwini
Saccopteryx leptura|BCBN845-05|ROM 105530|Ecuador.Napo
Saccopteryx leptura|BCBN846-05|ROM 105531|Ecuador.Napo
Saccopteryx leptura|ABGYE572-06|ROM 112047|Guyana.Potaro-Siparuni
Saccopteryx leptura|ABSMS182-06|ROM 117344|Suriname.Sipaliwini
Saccopteryx leptura|ABSMS423-06|ROM 117585|Suriname.Sipaliwini
Saccopteryx leptura|BCBNT860-06|ROM 114240|Suriname.Brokopondo
Lasiurus egregius|ABSMS071-06|ROM 117233|Suriname.Sipaliwini
Lasiurus egregius|ABSMS072-06|ROM 117234|Suriname.Sipaliwini
Lasiurus egregius|ABSMS362-06|ROM 117524|Suriname.Sipaliwini
Lasiurus egregius|ABSMS379-06|ROM 117541|Suriname.Sipaliwini
Tadarida brasiliensis|BCBN061-05|ROM 98387|Guatemala.El Progreso
Tadarida brasiliensis|BCBN060-05|ROM 98386|Guatemala.El Progreso
Tadarida brasiliensis|BCBN063-05|ROM 98390|Guatemala.El Progreso
Tadarida brasiliensis|BCBN062-05|ROM 98388|Guatemala.El Progreso
Tadarida brasiliensis|ABCSA147-06|ROM FN31378|Guatemala.El Progreso
Tadarida brasiliensis|BCBN064-05|ROM 98391|Guatemala.El Progreso
Eumops maurus|BCBN995-05|ROM 106326|Ecuador.Napo
Eumops auripendulus|BCBN843-05|ROM 105526|Ecuador.Napo
Eumops auripendulus|BCBNT337-06|ROM 108917|Guyana.Potaro-Siparuni
Eumops auripendulus|BCBNT338-06|ROM 108918|Guyana.Potaro-Siparuni
Eumops auripendulus|ABGYA890-07|ROM 103342|Guyana.Upper Takutu-Upper Essequibo
Eumops auripendulus|BCBN532-05|ROM 103160|Guyana.Upper Takutu-Upper Essequibo
Eumops auripendulus|BCBN535-05|ROM 103340|Guyana.Upper Takutu-Upper Essequibo
Eumops auripendulus|BCBNT339-06|ROM 108919|Guyana.Potaro-Siparuni
Molossops temminckii|BCBN820-05|ROM 105305|Ecuador.Napo
Molossops temminckii|BCBN841-05|ROM 105524|Ecuador.Napo
Molossops temminckii|BCBN829-05|ROM 105357|Ecuador.Napo
Molossops temminckii|BCBN842-05|ROM 105525|Ecuador.Napo
Molossops temminckii|BCBN943-05|ROM 105876|Ecuador.Napo
Molossops neglectus|ABSRA446-06|ROM 117036|Suriname.Nickerie
Molossops neglectus|BCBNT276-06|ROM 108446|Guyana.Potaro-Siparuni
Molossops neglectus|BCBNT286-06|ROM 108481|Guyana.Potaro-Siparuni
Molossops neglectus|ABSRA517-06|ROM 117107|Suriname.Nickerie
Molossops neglectus|ABSMS303-06|ROM 117465|Suriname.Sipaliwini
Molossops neglectus|BCBNT277-06|ROM 108447|Guyana.Potaro-Siparuni
Molossops neglectus|BCBNT287-06|ROM 108482|Guyana.Potaro-Siparuni
Molossops neglectus|BCBNT288-06|ROM 108483|Guyana.Potaro-Siparuni
Molossops neglectus|BCBNT289-06|ROM 108484|Guyana.Potaro-Siparuni
Cynomops planirostris|BCBN120-05|ROM 97854|Guyana.Upper Takutu-Upper Essequibo
Cynomops paranus|BCBN838-05|ROM 105504|Ecuador.Napo
Cynomops paranus|BCBNT389-06|ROM 109178|Guyana.Potaro-Siparuni
Cynomops paranus|BCBNT894-06|ROM 115525|Guyana.Essequibo Islands-West Demerara
Cynomops paranus|BCBNT543-06|ROM 112066|Guyana.Potaro-Siparuni
Cynomops paranus|BCBNT892-06|ROM 115523|Guyana.Essequibo Islands-West Demerara
Cynomops paranus|BCBNT895-06|ROM 115526|Guyana.Essequibo Islands-West Demerara
Cynomops paranus|BCBNT891-06|ROM 115522|Guyana.Essequibo Islands-West Demerara
Cynomops paranus|BCBNT893-06|ROM 115524|Guyana.Essequibo Islands-West Demerara
Cynomops paranus|BCBNT542-06|ROM 112065|Guyana.Potaro-Siparuni
Cynomops paranus|ABSRA507-06|ROM 117097|Suriname.Nickerie
Cynomops paranus|ABSRA420-06|ROM 117009|Suriname.Nickerie
Cynomops paranus|ABSRA419-06|ROM 117008|Suriname.Nickerie
Cynomops paranus|BCBNT896-06|ROM 115527|Guyana.Essequibo Islands-West Demerara
Cynomops paranus|BCBNT913-06|ROM 115579|Guyana.Essequibo Islands-West Demerara
Nyctinomops laticaudatus|BCBN017-05|ROM 95941|Mexico.Yucatan
Nyctinomops laticaudatus|BCBN034-05|ROM 96418|Mexico.Yucatan
Nyctinomops laticaudatus|BCBN019-05|ROM 95944|Mexico.Yucatan
Nyctinomops laticaudatus|BCBN018-05|ROM 95943|Mexico.Yucatan
Nyctinomops laticaudatus|BCBN016-05|ROM 95940|Mexico.Yucatan
Nyctinomops laticaudatus|ABMXC657-06|ROM 96422|Mexico.Yucatan
Nyctinomops laticaudatus|ABMXC656-06|ROM 96420|Mexico.Yucatan
Nyctinomops laticaudatus|ABMXC298-06|ROM 96423|Mexico.Yucatan
Nyctinomops laticaudatus|ABMXC297-06|ROM 96421|Mexico.Yucatan
Nyctinomops laticaudatus|ABMXC295-06|ROM FN30234|Mexico.Yucatan
Nyctinomops laticaudatus|BCBN033-05|ROM 96416|Mexico.Yucatan
Nyctinomops laticaudatus|ABMXC296-06|ROM 96419|Mexico.Yucatan
Nyctinomops laticaudatus|ABMXC086-06|ROM FN29733|Mexico.Yucatan
Nyctinomops laticaudatus|BCBN036-05|ROM 96425|Mexico.Yucatan
Nyctinomops laticaudatus|BCBN037-05|ROM 96426|Mexico.Yucatan
Nyctinomops laticaudatus|BCBN032-05|ROM 96414|Mexico.Yucatan
Nyctinomops laticaudatus|BCBNC223-06|ROM 96415|Mexico.Yucatan
Nyctinomops macrotis|BCBNT290-06|ROM 108485|Guyana.Potaro-Siparuni
Eumops hansae|ABRMM297-07|ROM 115464|Belize
Eumops hansae|BCBNT386-06|ROM 109153|Guyana.Potaro-Siparuni
Eumops hansae|BCBN864-05|ROM 105642|Ecuador.Napo
Eumops hansae|BCBNT270-06|ROM 108361|Guyana.Potaro-Siparuni
Eumops hansae|BCBNT303-06|ROM 108561|Guyana.Potaro-Siparuni
Eumops hansae|BCBNT354-06|ROM 108969|Guyana.Potaro-Siparuni
Eumops hansae|BCBN533-05|ROM 103327|Guyana.Upper Takutu-Upper Essequibo
Eumops hansae|BCBN525-05|ROM 103125|Guyana.Upper Takutu-Upper Essequibo
Eumops hansae|BCBNT150-06|ROM 107472|Guyana.Potaro-Siparuni
Eumops hansae|BCBNT400-06|ROM 109310|Guyana.Potaro-Siparuni
Natalus tumidirostris|ABGYG715-08|ROM 119143|Guyana
Natalus stramineus|BCBN483-05|ROM 97440|Mexico.Quintana Roo
Natalus stramineus|BCBN484-05|ROM 97441|Mexico.Quintana Roo
Natalus stramineus|BCBN487-05|ROM 97444|Mexico.Quintana Roo
Natalus stramineus|BCBN508-05|ROM 97518|Mexico.Quintana Roo
Natalus stramineus|BCBN485-05|ROM 97442|Mexico.Quintana Roo
Natalus stramineus|BCBN486-05|ROM 97443|Mexico.Quintana Roo
Natalus stramineus|BCBN009-05|ROM 95708|Mexico.Campeche
Natalus stramineus|BCBN488-05|ROM 97445|Mexico.Quintana Roo
Natalus stramineus|BCBN509-05|ROM 97519|Mexico.Quintana Roo
Natalus stramineus|BCBN240-05|ROM 99652|Guatemala.El Progreso
Natalus stramineus|BCBNT548-06|ROM 112172|Nicaragua.Rivas
Promops centralis|ABRMM107-07|ROM F41839|Ecuador
Promops centralis|ABRMM103-07|ROM F41835|Ecuador
Promops centralis|[illegible]|Ecuador.Napo

Natalus stramineus|BCBN1548-06|ROM 112172|Nicaragua.Rivas
Promops centralis|ABRMM107-07|ROM F41839|Ecuador
Promops centralis|ABRMM103-07|ROM F41835|Ecuador
Promops centralis|BCBN973-05|ROM 106020|Ecuador.Napo
Molossus sp.|BCBNT388-06|ROM 109176|Guyana.Potaro-Siparuni
Molossus rufus|ABCSA718-06|ROM 101285|El Salvador.Ahuachapan
Molossus rufus|ABSMS315-06|ROM 117477|Suriname.Sipaliwini
Molossus rufus|BCBN396-05|ROM 101280|El Salvador.Ahuachapan
Molossus rufus|ABCSA715-06|ROM 101278|El Salvador.Ahuachapan
Molossus rufus|ABCSA716-06|ROM 101282|El Salvador.Ahuachapan
Molossus rufus|ABCSA717-06|ROM 101283|El Salvador.Ahuachapan
Molossus rufus|BCBN398-05|ROM 101284|El Salvador.Ahuachapan
Molossus rufus|ABCSA719-06|ROM 101287|El Salvador.Ahuachapan
Molossus rufus|BCBN395-05|ROM 101279|El Salvador.Ahuachapan
Molossus rufus|BCBN020-05|ROM 96186|Mexico.Tabasco
Molossus rufus|BCBN397-05|ROM 101281|El Salvador.Ahuachapan
Molossus rufus|BCBN399-05|ROM 101286|El Salvador.Ahuachapan
Molossus rufus|BCBN827-05|ROM 105355|Ecuador.Napo
Molossus rufus|ABSMS386-06|ROM 117548|Suriname.Sipaliwini
Molossus rufus|ABSMS312-06|ROM 117474|Suriname.Sipaliwini
Molossus rufus|ABSMS316-06|ROM 117478|Suriname.Sipaliwini
Molossus rufus|ABSRA418-06|ROM 117007|Suriname.Nickerie
Molossus rufus|ABSMS313-06|ROM 117475|Suriname.Sipaliwini
Molossus rufus|BCBN855-05|ROM 105602|Ecuador.Napo
Molossus rufus|ABGYC428-06|ROM 107250|Guyana.Potaro-Siparuni
Molossus rufus|BCBNT272-06|ROM 108420|Guyana.Potaro-Siparuni
Molossus rufus|ABGYD165-06|ROM 108479|Guyana.Potaro-Siparuni
Molossus rufus|ABSMS311-06|ROM 117473|Suriname.Sipaliwini
Molossus rufus|ABSRA410-06|ROM 116999|Suriname.Nickerie
Molossus rufus|BCBNT285-06|ROM 108478|Guyana.Potaro-Siparuni
Molossus rufus|ABRMM064-07|ROM F39071|Guyana.Potaro-Siparuni
Molossus rufus|ABECA635-06|ROM 105682|Ecuador.Napo
Molossus rufus|BCBN875-05|ROM 105681|Ecuador.Napo
Molossus rufus|ABGYD166-06|ROM 108480|Guyana.Potaro-Siparuni
Molossus rufus|ABGYC611-06|ROM F39068|Guyana.Potaro-Siparuni
Molossus rufus|BCBNT105-06|ROM 107167|Guyana.Potaro-Siparuni
Molossus rufus|ABSRA411-06|ROM 117000|Suriname.Nickerie
Molossus rufus|ABECA405-06|ROM 105304|Ecuador.Napo
Molossus rufus|BCBNT107-06|ROM 107177|Guyana.Potaro-Siparuni
Molossus rufus|BCBNT271-06|ROM 108419|Guyana.Potaro-Siparuni
Molossus rufus|ABSMS304-06|ROM 117466|Suriname.Sipaliwini
Molossus rufus|ABRMM101-07|ROM F41833|Ecuador
Molossus rufus|ABGYD758-06|ROM 109177|Guyana.Potaro-Siparuni
Molossus rufus|ABGYD737-06|ROM 109154|Guyana.Potaro-Siparuni
Molossus rufus|ABSMS314-06|ROM 117476|Suriname.Sipaliwini
Molossus rufus|ABGYD121-06|ROM 108421|Guyana.Potaro-Siparuni
Molossus rufus|ABGYD122-06|ROM 108422|Guyana.Potaro-Siparuni
Molossus rufus|ABSMS317-06|ROM 117479|Suriname.Sipaliwini
Molossus rufus|ABSMS319-06|ROM 117481|Suriname.Sipaliwini
Molossus rufus|ABSMS387-06|ROM 117549|Suriname.Sipaliwini
Molossus rufus|ABSMS388-06|ROM 117550|Suriname.Sipaliwini
Molossus rufus|BCBN878-05|ROM 105686|Ecuador.Napo
Molossus rufus|BCBNT732-06|ROM 113903|Suriname.Brokopondo
Molossus coibensis|BCBN861-05|ROM 105637|Ecuador.Napo
Molossus coibensis|BCBN880-05|ROM 105688|Ecuador.Napo
Molossus coibensis|BCBN862-05|ROM 105638|Ecuador.Napo
Molossus coibensis|ABRMM098-07|ROM F41830|Ecuador
Molossus coibensis|BCBN819-05|ROM 105303|Ecuador.Napo
Molossus coibensis|BCBN879-05|ROM 105687|Ecuador.Napo
Molossus coibensis|BCBN881-05|ROM 105690|Ecuador.Napo
Molossus molossus|BCBNT181-06|ROM 107869|Venezuela.Amazonas
Molossus molossus|BCBNT195-06|ROM 107900|Venezuela.Amazonas
Molossus molossus|BCBNT196-06|ROM 107901|Venezuela.Amazonas
Molossus molossus|ABGYA026-06|ROM 97808|Guyana.Upper Takutu-Upper Essequibo
Molossus molossus|ABGYA318-06|ROM 98716|Guyana.Barima-Waini
Molossus molossus|ABECA508-06|ROM 105543|Ecuador.Napo
Molossus molossus|BCBN828-05|ROM 105356|Ecuador.Napo
Molossus molossus|ABECA491-06|ROM 105514|Ecuador.Napo
Molossus molossus|ABGYD641-06|ROM 109049|Guyana.Potaro-Siparuni
Molossus molossus|ABGYD650-06|ROM 109058|Guyana.Potaro-Siparuni
Molossus molossus|ABGYA005-06|ROM 97773|Guyana.Upper Takutu-Upper Essequibo
Molossus molossus|BCBNT947-06|ROM 115673|Guyana.Potaro-Siparuni
Molossus molossus|ABGYE517-06|ROM 111989|Guyana.Potaro-Siparuni
Molossus molossus|ABECB123-08|ROM 118785|Ecuador
Molossus molossus|BCBNT959-06|ROM 115725|Guyana.Potaro-Siparuni
Molossus molossus|ABECA507-06|ROM 105542|Ecuador.Napo
Molossus molossus|ABGYA023-06|ROM 97805|Guyana.Upper Takutu-Upper Essequibo
Molossus molossus|ABGYA025-06|ROM 97807|Guyana.Upper Takutu-Upper Essequibo
Molossus molossus|ABSMS498-06|ROM 117660|Suriname.Sipaliwini
Molossus molossus|BCBNT555-06|ROM 112546|Guyana.Demerara-Mahaica
Molossus molossus|ABGYA322-06|ROM 98720|Guyana.Barima-Waini
Molossus molossus|ABECA541-06|ROM 105568|Ecuador.Napo
Molossus molossus|ABGYE514-06|ROM 111986|Guyana.Potaro-Siparuni
Molossus molossus|ABSMS496-06|ROM 117658|Suriname.Sipaliwini
Molossus molossus|BCBNT948-06|ROM 115674|Guyana.Potaro-Siparuni
Molossus molossus|ABSMS520-06|ROM 117682|Suriname.Sipaliwini
Molossus molossus|ABGYB806-06|ROM 103574|Guyana.Upper Demerara-Berbice
Molossus molossus|ABGYG510-06|ROM 116652|Guyana.Potaro-Siparuni
Molossus molossus|ABGYB801-06|ROM 103569|Guyana.Upper Demerara-Berbice
Molossus molossus|ABGYB803-06|ROM 103571|Guyana.Upper Demerara-Berbice
Molossus molossus|BCBNT951-06|ROM 115689|Guyana.Potaro-Siparuni
Molossus molossus|ABECB007-06|ROM 106324|Ecuador.Napo
Molossus molossus|ABGYB794-06|ROM 103562|Guyana.Upper Demerara-Berbice
Molossus molossus|ABSMS494-06|ROM 117656|Suriname.Sipaliwini
Molossus molossus|ABGYB795-06|ROM 103563|Guyana.Upper Demerara-Berbice
Molossus molossus|ABGYF189-06|ROM 113750|Guyana.Demerara-Mahaica
Molossus molossus|ABGYG397-06|ROM 116525|Guyana.Potaro-Siparuni
Molossus molossus|ABGYD642-06|ROM 109050|Guyana.Potaro-Siparuni
Molossus molossus|ABGYE562-06|ROM 112035|Guyana.Potaro-Siparuni
Molossus molossus|ABGYB808-06|ROM 103576|Guyana.Upper Demerara-Berbice
Molossus molossus|ABGYB807-06|ROM 103575|Guyana.Upper Demerara-Berbice
Molossus molossus|ABGYB805-06|ROM 103573|Guyana.Upper Demerara-Berbice
Molossus molossus|ABGYB797-06|ROM 103565|Guyana.Upper Demerara-Berbice

Molossus molossus|ABGYB807-06|ROM 103575|Guyana.Upper Demerara-Berbice
Molossus molossus|ABGYB805-06|ROM 103573|Guyana.Upper Demerara-Berbice
Molossus molossus|ABGYB797-06|ROM 103565|Guyana.Upper Demerara-Berbice
Molossus molossus|ABGYB793-06|ROM 103561|Guyana.Upper Demerara-Berbice
Molossus molossus|ABGYB791-06|ROM 103559|Guyana.Upper Demerara-Berbice
Molossus molossus|ABGYB790-06|ROM 103558|Guyana.Upper Demerara-Berbice
Molossus molossus|ABGYB789-06|ROM 103557|Guyana.Upper Demerara-Berbice
Molossus molossus|ABGYA307-06|ROM 98703|Guyana.Barima-Waini
Molossus molossus|ABECA450-06|ROM 105360|Ecuador.Napo
Molossus molossus|ABECB006-06|ROM F40478|Ecuador.Napo
Molossus molossus|ABGYA020-06|ROM 97802|Guyana.Upper Takutu-Upper Essequibo
Molossus molossus|ABGYA022-06|ROM 97804|Guyana.Upper Takutu-Upper Essequibo
Molossus molossus|ABGYB802-06|ROM 103570|Guyana.Upper Demerara-Berbice
Molossus molossus|ABGYB796-06|ROM 103564|Guyana.Upper Demerara-Berbice
Molossus molossus|ABGYB809-06|ROM 103577|Guyana.Upper Demerara-Berbice
Molossus molossus|ABGYC730-06|ROM F39787|Guyana.Cuyuni-Mazaruni
Molossus molossus|ABGYG493-06|ROM 116634|Guyana.Potaro-Siparuni
Molossus molossus|ABGYA324-06|ROM 98722|Guyana.Barima-Waini
Molossus molossus|BCBNT755-06|ROM 113950|Suriname.Brokopondo
Molossus molossus|BCBNT961-06|ROM 115727|Guyana.Potaro-Siparuni
Molossus molossus|ABECA542-06|ROM 105569|Ecuador.Napo
Molossus molossus|BCBN601-05|ROM 104194|Panama.Canal Zone
Molossus molossus|BCBN944-05|ROM 105878|Ecuador.Napo
Molossus molossus|BCBN954-05|ROM 105926|Ecuador.Napo
Molossus molossus|ABECA036-06|ROM 104018|Ecuador.Napo
Molossus molossus|BCBN977-05|ROM 106053|Ecuador.Napo
Molossus molossus|ABECA040-06|ROM 104022|Ecuador.Napo
Molossus molossus|ABECB008-06|ROM 106325|Ecuador.Napo
Molossus molossus|BCBN978-05|ROM 106054|Ecuador.Napo
Molossus molossus|ABGYA027-06|ROM 97809|Guyana.Upper Takutu-Upper Essequibo
Molossus molossus|ABGYC726-06|ROM 108126|Guyana.Cuyuni-Mazaruni
Molossus molossus|ABSMS495-06|ROM 117657|Suriname.Sipaliwini
Molossus molossus|ABGYA319-06|ROM 98717|Guyana.Barima-Waini
Molossus molossus|ABGYA323-06|ROM 98721|Guyana.Barima-Waini
Molossus molossus|ABGYB788-06|ROM 103556|Guyana.Upper Demerara-Berbice
Molossus molossus|ABGYC729-06|ROM F39786|Guyana.Cuyuni-Mazaruni
Molossus molossus|ABGYE520-06|ROM 111992|Guyana.Potaro-Siparuni
Molossus molossus|BCBNT376-06|ROM 109044|Guyana.Potaro-Siparuni
Molossus molossus|BCBNT943-06|ROM 115644|Guyana.Potaro-Siparuni
Molossus molossus|ABGYE519-06|ROM 111991|Guyana.Potaro-Siparuni
Molossus molossus|ABSMS499-06|ROM 117661|Suriname.Sipaliwini
Molossus molossus|BCBNT377-06|ROM 109045|Guyana.Potaro-Siparuni
Molossus molossus|BCBNT730-06|ROM 113901|Suriname.Brokopondo
Molossus molossus|ABGYD644-06|ROM 109052|Guyana.Potaro-Siparuni
Molossus molossus|ABGYD649-06|ROM 109057|Guyana.Potaro-Siparuni
Molossus molossus|BCBNT952-06|ROM 115690|Guyana.Potaro-Siparuni
Molossus molossus|ABGYA024-06|ROM 97806|Guyana.Upper Takutu-Upper Essequibo
Molossus molossus|ABGYD638-06|ROM 109046|Guyana.Potaro-Siparuni
Molossus molossus|ABRMM106-07|ROM F41838|Ecuador
Molossus molossus|ABRMM105-07|ROM F41837|Ecuador
Molossus molossus|ABGYF235-06|ROM 113813|Guyana.Demerara-Mahaica
Molossus molossus|ABGYC727-06|ROM 108127|Guyana.Cuyuni-Mazaruni
Molossus molossus|ABGYB800-06|ROM 103568|Guyana.Upper Demerara-Berbice
Molossus molossus|ABGYB792-06|ROM 103560|Guyana.Upper Demerara-Berbice
Molossus molossus|ABECA509-06|ROM 105544|Ecuador.Napo
Molossus molossus|BCBNT960-06|ROM 115726|Guyana.Potaro-Siparuni
Molossus molossus|BCBNT373-06|ROM 109041|Guyana.Potaro-Siparuni
Molossus molossus|ABECA561-06|ROM 105590|Ecuador.Napo
Molossus molossus|ABECA637-06|ROM 105689|Ecuador.Napo
Molossus molossus|ABGYE516-06|ROM 111988|Guyana.Potaro-Siparuni
Molossus molossus|ABGYE521-06|ROM 111993|Guyana.Potaro-Siparuni
Molossus molossus|ABECA457-06|ROM 105367|Ecuador.Napo
Molossus molossus|ABECA510-06|ROM 105545|Ecuador.Napo
Molossus molossus|ABGYE513-06|ROM 111985|Guyana.Potaro-Siparuni
Molossus molossus|ABGYD646-06|ROM 109054|Guyana.Potaro-Siparuni
Molossus molossus|ABGYD647-06|ROM 109055|Guyana.Potaro-Siparuni
Molossus molossus|ABGYD648-06|ROM 109056|Guyana.Potaro-Siparuni
Molossus molossus|ABGYD651-06|ROM 109059|Guyana.Potaro-Siparuni
Molossus molossus|ABGYD652-06|ROM 109060|Guyana.Potaro-Siparuni
Molossus molossus|ABGYE508-06|ROM 111980|Guyana.Potaro-Siparuni
Molossus molossus|ABGYE745-06|ROM 112688|Guyana.Demerara-Mahaica
Molossus molossus|ABGYE746-06|ROM 112689|Guyana.Demerara-Mahaica
Molossus molossus|ABGYE515-06|ROM 111987|Guyana.Potaro-Siparuni
Molossus molossus|ABGYE518-06|ROM 111990|Guyana.Potaro-Siparuni
Molossus molossus|ABGYE754-06|ROM 112700|Guyana.Demerara-Mahaica
Molossus molossus|ABGYF236-06|ROM 113814|Guyana.Demerara-Mahaica
Molossus molossus|ABRMM104-07|ROM F41836|Ecuador
Molossus molossus|ABSMS497-06|ROM 117659|Suriname.Sipaliwini
Molossus molossus|BCBNT374-06|ROM 109042|Guyana.Potaro-Siparuni
Molossus molossus|BCBNT375-06|ROM 109043|Guyana.Potaro-Siparuni
Molossus molossus|BCBNT728-06|ROM 113899|Suriname.Brokopondo
Molossus molossus|BCBNT729-06|ROM 113900|Suriname.Brokopondo
Molossus molossus|BCBNT731-06|ROM 113902|Suriname.Brokopondo
Molossus molossus|BCBNT958-06|ROM 115724|Guyana.Potaro-Siparuni
Molossus molossus|ABGYD645-06|ROM 109053|Guyana.Potaro-Siparuni
Molossus molossus|ABGYD640-06|ROM 109048|Guyana.Potaro-Siparuni
Molossus molossus|ABGYD639-06|ROM 109047|Guyana.Potaro-Siparuni
Molossus molossus|ABGYC067-06|ROM 106554|Guyana.Upper Takutu-Upper Essequibo
Molossus molossus|ABGYB804-06|ROM 103572|Guyana.Upper Demerara-Berbice
Molossus molossus|ABGYB799-06|ROM 103567|Guyana.Upper Demerara-Berbice
Molossus molossus|ABGYB798-06|ROM 103566|Guyana.Upper Demerara-Berbice
Molossus molossus|ABGYA320-06|ROM 98718|Guyana.Barima-Waini
Molossus molossus|ABGYA021-06|ROM 97803|Guyana.Upper Takutu-Upper Essequibo
Molossus molossus|ABECA137-06|ROM 104435|Ecuador.Napo
Molossus molossus|ABGYA007-06|ROM 97775|Guyana.Upper Takutu-Upper Essequibo
Molossus molossus|ABGYD643-06|ROM 109051|Guyana.Potaro-Siparuni
Molossus molossus|ABGYG700-08|ROM 119128|Guyana
Molossus molossus|BCBNT962-06|ROM 115728|Guyana.Potaro-Siparuni
Lasiurus atratus|ABSMS346-06|ROM 117508|Suriname.Sipaliwini
Lasiurus atratus|BCBNT112-06|ROM 107228|Guyana.Potaro-Siparuni
Lasiurus atratus|ABSRA447-06|ROM 117037|Suriname.Nickerie
Lasiurus atratus|BCBNT391-06|ROM 109207|Guyana.Potaro-Siparuni
Lasiurus blossevillii|BCBN267-05|ROM 99782|Guatemala.Solola

Lasiurus atratus|ABSRA447-06|ROM 117037|Suriname.Nickerie
Lasiurus atratus|BCBNT391-06|ROM 109207|Guyana.Potaro-Siparuni
Lasiurus blossevillii|BCBN267-05|ROM 99782|Guatemala.Solola
Lasiurus blossevillii|BCBN268-05|ROM 99783|Guatemala.Solola
Lasiurus blossevillii|BCBN269-05|ROM 99784|Guatemala.Solola
Lasiurus blossevillii|BCBN664-05|ROM 104285|Panama.Chiriqui
Lasiurus blossevillii|ABSMS295-06|ROM 117457|Suriname.Sipaliwini
Lasiurus blossevillii|BCBNT455-06|ROM 111049|Brazil.Sao Paulo
Lasiurus blossevillii|BCBNT459-06|ROM 111055|Brazil.Sao Paulo
Rhogeessa io|ABGYG664-08|ROM 119092|Guyana
Rhogeessa io|ABGYG693-08|ROM 119121|Guyana
Rhogeessa io|ABGYG722-08|ROM 119150|Guyana
Rhogeessa io|ABGYG893-08|ROM 119321|Guyana
Rhogeessa io|ABGYG910-08|ROM 119338|Guyana
Rhogeessa io|ABGYG948-08|ROM 119376|Guyana
Rhogeessa aeneus|ABMXA906-06|ROM FN30678|Mexico.Campeche
Rhogeessa aeneus|BCBN053-05|ROM 98229|Mexico.Quintana Roo
Rhogeessa aeneus|BCBN030-05|ROM 96408|Mexico.Campeche
Rhogeessa aeneus|ABMXA780-06|ROM FN30463|Mexico.Yucatan
Rhogeessa aeneus|ABMXA905-06|ROM FN30677|Mexico.Campeche
Rhogeessa aeneus|ABMXA944-06|ROM MDE6002|Mexico.Quintana Roo
Rhogeessa aeneus|ABMXA945-06|ROM MDE6003|Mexico.Quintana Roo
Rhogeessa aeneus|BCBN014-05|ROM 95784|Mexico.Campeche
Rhogeessa aeneus|BCBN031-05|ROM 96409|Mexico.Campeche
Rhogeessa aeneus|BCBN052-05|ROM 98228|Mexico.Quintana Roo
Rhogeessa aeneus|BCBN050-05|ROM 98226|Mexico.Quintana Roo
Rhogeessa aeneus|BCBN235-05|ROM 99531|Guatemala.Peten
Rhogeessa aeneus|ABMXA781-06|ROM FN30464|Mexico.Yucatan
Rhogeessa aeneus|ABMXC291-06|ROM 96406|Mexico.Campeche
Rhogeessa aeneus|ABMXC292-06|ROM 96407|Mexico.Campeche
Rhogeessa aeneus|ABMXA779-06|ROM FN30462|Mexico.Yucatan
Rhogeessa aeneus|BCBN236-05|ROM 99532|Guatemala.Peten
Rhogeessa aeneus|BCBN237-05|ROM 99533|Guatemala.Peten
Rhogeessa tumida|BCBN415-05|ROM 101320|El Salvador.Ahuachapan
Rhogeessa tumida|BCBN416-05|ROM 101321|El Salvador.Ahuachapan
Bauerus dubiaquercus|BCBN234-05|ROM 99530|Guatemala.Peten
Bauerus dubiaquercus|BCBN521-05|ROM 97719|Mexico.Campeche
Euderma maculatum|ABMXA458-06|ROM ASK692|Mexico.Queretaro
Eptesicus brasiliensis|BCBN663-05|ROM 104284|Panama.Chiriqui
Eptesicus furinalis|ABMXA938-06|ROM FN33847|Mexico.Quintana Roo
Eptesicus furinalis|ABMXA937-06|ROM FN33846|Mexico.Quintana Roo
Eptesicus furinalis|ABMXA935-06|ROM FN33844|Mexico.Quintana Roo
Eptesicus furinalis|ABMXA936-06|ROM FN33845|Mexico.Quintana Roo
Eptesicus furinalis|ABMXA939-06|ROM FN33848|Mexico.Quintana Roo
Eptesicus furinalis|ABMXA940-06|ROM FN33849|Mexico.Quintana Roo
Eptesicus furinalis|ABMXA941-06|ROM FN33850|Mexico.Quintana Roo
Eptesicus furinalis|ABMXA942-06|ROM MDE6000|Mexico.Quintana Roo
Eptesicus furinalis|BCBN197-05|ROM 99324|Guatemala.Peten
Eptesicus chiriquinus|BCBN318-05|ROM 100811|Guyana.Barima-Waini
Eptesicus chiriquinus|ABSMS074-06|ROM 117236|Suriname.Sipaliwini
Eptesicus chiriquinus|ABSMS208-06|ROM 117370|Suriname.Sipaliwini
Eptesicus chiriquinus|ABSMS305-06|ROM 117467|Suriname.Sipaliwini
Eptesicus chiriquinus|ABSMS107-06|ROM 117269|Suriname.Sipaliwini
Eptesicus chiriquinus|ABSMS163-06|ROM 117325|Suriname.Sipaliwini
Eptesicus chiriquinus|ABSMS246-06|ROM 117408|Suriname.Sipaliwini
Eptesicus chiriquinus|ABSMS347-06|ROM 117509|Suriname.Sipaliwini
Eptesicus chiriquinus|ABSMS348-06|ROM 117510|Suriname.Sipaliwini
Eptesicus chiriquinus|ABSMS345-06|ROM 117507|Suriname.Sipaliwini
Eptesicus chiriquinus|ABSMS524-06|ROM 117686|Suriname.Sipaliwini
Eptesicus chiriquinus|ABSMS276-06|ROM 117438|Suriname.Sipaliwini
Eptesicus chiriquinus|ABSMS140-06|ROM 117302|Suriname.Sipaliwini
Eptesicus chiriquinus|ABSMS275-06|ROM 117437|Suriname.Sipaliwini
Eptesicus chiriquinus|ABSMS270-06|ROM 117432|Suriname.Sipaliwini
Eptesicus chiriquinus|ABSMS073-06|ROM 117235|Suriname.Sipaliwini
Eptesicus chiriquinus|ABSMS132-06|ROM 117294|Suriname.Sipaliwini
Eptesicus chiriquinus|ABSMS152-06|ROM 117314|Suriname.Sipaliwini
Eptesicus chiriquinus|ABSMS378-06|ROM 117540|Suriname.Sipaliwini
Eptesicus chiriquinus|ABSMS488-06|ROM 117650|Suriname.Sipaliwini
Eptesicus chiriquinus|ABSMS518-06|ROM 117680|Suriname.Sipaliwini
Eptesicus chiriquinus|BCBNT721-06|ROM 113891|Suriname.Brokopondo
Eptesicus furinalis|BCBN824-05|ROM 105343|Ecuador.Napo
Eptesicus furinalis|BCBN830-05|ROM 105359|Ecuador.Napo
Eptesicus furinalis|BCBN850-05|ROM 105538|Ecuador.Napo
Eptesicus furinalis|BCBN338-05|ROM 100922|Guyana.Barima-Waini
Eptesicus furinalis|ABSMS508-06|ROM 117670|Suriname.Sipaliwini
Eptesicus furinalis|BCBNT575-06|ROM 112641|Guyana.Demerara-Mahaica
Eptesicus furinalis|ABSMS506-06|ROM 117668|Suriname.Sipaliwini
Eptesicus furinalis|BCBNT583-06|ROM 112691|Guyana.Demerara-Mahaica
Eptesicus furinalis|ABSRA393-06|ROM 116982|Suriname.Nickerie
Eptesicus furinalis|ABSMS517-06|ROM 117679|Suriname.Sipaliwini
Eptesicus furinalis|BCBN337-05|ROM 100921|Guyana.Barima-Waini
Eptesicus furinalis|ABSMS511-06|ROM 117673|Suriname.Sipaliwini
Eptesicus furinalis|ABSMS509-06|ROM 117671|Suriname.Sipaliwini
Eptesicus furinalis|BCBNT694-06|ROM 113815|Guyana.Demerara-Mahaica
Eptesicus furinalis|BCBNT711-06|ROM 113880|Suriname.Brokopondo
Eptesicus furinalis|BCBN526-05|ROM 103132|Guyana.Upper Takutu-Upper Essequibo
Eptesicus furinalis|ABSMS452-06|ROM 117614|Suriname.Sipaliwini
Eptesicus furinalis|ABSMS507-06|ROM 117669|Suriname.Sipaliwini
Eptesicus furinalis|ABSMS510-06|ROM 117672|Suriname.Sipaliwini
Eptesicus furinalis|BCBN112-05|ROM 97826|Guyana.Upper Takutu-Upper Essequibo
Eptesicus furinalis|BCBNT897-06|ROM 115528|Guyana.Essequibo Islands-West Demerara
Eptesicus furinalis|BCBNT898-06|ROM 115529|Guyana.Essequibo Islands-West Demerara
Myotis nigricans PS1|ABSMS269-06|ROM 117431|Suriname.Sipaliwini
Myotis nigricans PS1|BCBN137-05|ROM 98018|Guyana.Potaro-Siparuni
Myotis nigricans PS1|BCBN307-05|ROM 100348|Guyana.East Berbice-Corentyne
Myotis albescens|BCBNT036-06|ROM 106655|Guyana.Upper Takutu-Upper Essequibo
Myotis albescens|ABGYG595-08|ROM 119023|Guyana
Myotis albescens|BCBN131-05|ROM 97922|Guyana.Upper Takutu-Upper Essequibo
Myotis albescens|ABGYG596-08|ROM 119024|Guyana
Myotis albescens|ABGYG620-08|ROM 119048|Guyana
Myotis albescens|ABGYG621-08|ROM 119049|Guyana
Myotis albescens|ABGYG622-08|ROM 119050|Guyana
Myotis albescens|ABGYG624-08|ROM 119052|Guyana

[illegible]
Myotis albescens|ABGYG621-08|ROM 119049|Guyana
Myotis albescens|ABGYG622-08|ROM 119050|Guyana
Myotis albescens|ABGYG624-08|ROM 119052|Guyana
Myotis albescens|BCBN286-05|ROM 100228|Guyana.East Berbice-Corentyne
Myotis albescens|BCBNT103-06|ROM 107115|Guyana.Potaro-Siparuni
Myotis albescens|ABGYA161-06|ROM 98019|Guyana.Potaro-Siparuni
Myotis albescens|ABGYC242-06|ROM 106795|Guyana.Upper Takutu-Upper Essequibo
Myotis albescens|ABSRA392-06|ROM 116981|Suriname.Nickerie
Myotis albescens|BCBNT539-06|ROM 112041|Guyana.Potaro-Siparuni
Myotis albescens|ABSMS037-06|ROM 117199|Suriname.Sipaliwini
Myotis albescens|ABSRA412-06|ROM 117001|Suriname.Nickerie
Myotis albescens|BCBNT540-06|ROM 112048|Guyana.Potaro-Siparuni
Myotis albescens|ABGYG619-08|ROM 119047|Guyana
Myotis albescens|ABGYG623-08|ROM 119051|Guyana
Myotis albescens|ABGYG683-08|ROM 119111|Guyana
Myotis albescens|ABGYE211-06|ROM 111658|Guyana.Potaro-Siparuni
Myotis albescens|ABGYE212-06|ROM 111659|Guyana.Potaro-Siparuni
Myotis albescens|BCBN136-05|ROM 98017|Guyana.Potaro-Siparuni
Myotis albescens|BCBNT537-06|ROM 111997|Guyana.Potaro-Siparuni
Myotis albescens|ABGYC261-06|ROM 107047|Guyana.Potaro-Siparuni
Myotis albescens|BCBNT571-06|ROM 112625|Guyana.Demerara-Mahaica
Myotis velifer|BCBN433-05|ROM 101358|El Salvador.Santa Ana
Myotis velifer|BCBN434-05|ROM 101359|El Salvador.Santa Ana
Myotis riparius PS1|BCBNC043-06|ROM 114789|Guyana.Potaro-Siparuni
Myotis riparius PS1|BCBNC187-06|ROM 116560|Guyana.Potaro-Siparuni
Myotis riparius PS1|BCBNC192-06|ROM 116613|Guyana.Potaro-Siparuni
Myotis riparius PS2|BCBNC181-06|ROM 116523|Guyana.Potaro-Siparuni
Myotis riparius PS2|BCBNT560-06|ROM 112572|Guyana.Demerara-Mahaica
Myotis riparius PS2|BCBNT569-06|ROM 112605|Guyana.Demerara-Mahaica
Myotis riparius PS2|BCBNT580-06|ROM 112665|Guyana.Demerara-Mahaica
Myotis riparius PS2|BCBNT677-06|ROM 113743|Guyana.Demerara-Mahaica
Myotis riparius PS2|BCBNT552-06|ROM 112532|Guyana.Demerara-Mahaica
Myotis riparius PS2|BCBNT559-06|ROM 112571|Guyana.Demerara-Mahaica
Myotis riparius PS2|BCBNT693-06|ROM 113810|Guyana.Demerara-Mahaica
Myotis keaysi|ABMXA932-06|ROM FN33841|Mexico.Quintana Roo
Myotis keaysi|ABMXA933-06|ROM FN33842|Mexico.Quintana Roo
Myotis keaysi|ABMXA760-06|ROM FN30435|Mexico.Yucatan
Myotis keaysi|ABMXA951-06|ROM MDE6009|Mexico.Quintana Roo
Myotis keaysi|BCBN043-05|ROM 96463|Mexico.Yucatan
Myotis keaysi|ABMXA950-06|ROM MDE6008|Mexico.Quintana Roo
Myotis keaysi|BCBN044-05|ROM 96464|Mexico.Yucatan
Myotis keaysi|BCBN177-05|ROM 99232|Guatemala.Peten
Myotis keaysi|BCBN178-05|ROM 99233|Guatemala.Peten
Myotis keaysi|ABCSA841-06|ROM 101467|El Salvador.Santa Ana
Myotis keaysi|BCBN276-05|ROM 99833|Guatemala.Zacapa
Myotis keaysi|ABCSA840-06|ROM 101466|El Salvador.Santa Ana
Myotis keaysi|ABCSA754-06|ROM 101355|El Salvador.Santa Ana
Myotis keaysi|ABCSA812-06|ROM 101431|El Salvador.Santa Ana
Myotis keaysi|ABCSA814-06|ROM 101433|El Salvador.Santa Ana
Myotis keaysi|BCBN432-05|ROM 101353|El Salvador.Santa Ana
Myotis keaysi|ABCSA753-06|ROM 101354|El Salvador.Santa Ana
Myotis keaysi|ABCSA813-06|ROM 101432|El Salvador.Santa Ana
Myotis keaysi|ABCSA839-06|ROM 101465|El Salvador.Santa Ana
Myotis keaysi|ABCSA891-06|ROM 101525|El Salvador.Santa Ana
Myotis keaysi|BCBN394-05|ROM 101273|El Salvador.Ahuachapan
Myotis keaysi|BCBN431-05|ROM 101352|El Salvador.Santa Ana
Myotis keaysi|ABCSA756-06|ROM 101357|El Salvador.Santa Ana
Myotis keaysi|BCBN450-05|ROM 101430|El Salvador.Santa Ana
Myotis keaysi|ABCSA755-06|ROM 101356|El Salvador.Santa Ana
Myotis keaysi|BCBN464-05|ROM 101524|El Salvador.Santa Ana
Myotis keaysi|BCBN677-05|ROM 104302|Panama.Chiriqui
Myotis ruber|BCBNT489-06|ROM 111110|Brazil.Sao Paulo
Myotis ruber|BCBNT490-06|ROM 111111|Brazil.Sao Paulo
Myotis ruber|BCBNT498-06|ROM 111137|Brazil.Sao Paulo
Myotis elegans|BCBN209-05|ROM 99433|Guatemala.Peten
Myotis elegans|BCBN231-05|ROM 99515|Guatemala.Peten
Myotis elegans|BCBN414-05|ROM 101319|El Salvador.Ahuachapan
Myotis elegans|BCBN402-05|ROM 101293|El Salvador.Ahuachapan
Myotis elegans|BCBN430-05|ROM 101349|El Salvador.Ahuachapan
Myotis riparius PS3|BCBNC060-06|ROM 115113|Guyana.Cuyuni-Mazaruni
Myotis riparius PS3|BCBNT600-06|ROM 113462|Guyana.Upper Takutu-Upper Essequibo
Myotis riparius PS3|BCBNC142-06|ROM 115825|Guyana.Potaro-Siparuni
Myotis riparius PS3|BCBNC002-06|ROM 114689|Guyana.Potaro-Siparuni
Myotis riparius PS3|BCBNC143-06|ROM 115852|Guyana.Potaro-Siparuni
Myotis riparius PS3|BCBNT541-06|ROM 112049|Guyana.Potaro-Siparuni
Myotis riparius PS3|BCBNT604-06|ROM 113494|Guyana.Upper Takutu-Upper Essequibo
Myotis riparius PS3|BCBNT068-06|ROM 106773|Guyana.Upper Takutu-Upper Essequibo
Myotis riparius PS3|BCBNC056-06|ROM 115087|Guyana.Cuyuni-Mazaruni
Myotis riparius PS3|BCBNT063-06|ROM 106751|Guyana.Upper Takutu-Upper Essequibo
Myotis riparius PS3|BCBN530-05|ROM 103136|Guyana.Upper Takutu-Upper Essequibo
Myotis riparius PS3|BCBNC122-06|ROM 115777|Guyana.Potaro-Siparuni
Myotis riparius PS3|BCBNT971-06|ROM 114620|Guyana.Potaro-Siparuni
Artibeus lituratus|BCBNT714-06|ROM 113883|Suriname.Brokopondo
Artibeus lituratus|ABGYA202-06|ROM 98070|Guyana.Potaro-Siparuni
Artibeus lituratus|BCBNT485-06|ROM 111102|Brazil.Sao Paulo
Artibeus lituratus|BCBNT092-06|ROM 107092|Guyana.Potaro-Siparuni
Artibeus intermedius|BCBN469-05|ROM 96960|Mexico.Campeche
Artibeus lituratus|BCBN117-05|ROM 97848|Guyana.Upper Takutu-Upper Essequibo
Artibeus lituratus|ABSRA372-06|ROM 116961|Suriname.Nickerie
Artibeus lituratus|ABSA103-06|ROM 111143|Brazil.Sao Paulo
Artibeus lituratus|ABSA099-06|ROM 111136|Brazil.Sao Paulo
Artibeus lituratus|ABRMM078-07|ROM F39672|Guyana.Potaro-Siparuni
Artibeus lituratus|ABGYG1031-08|ROM 118955|Guyana
Artibeus lituratus|ABGYG085-06|ROM 115622|Guyana.Essequibo Islands-West Demerara
Artibeus lituratus|ABGYG076-06|ROM 115606|Guyana.Essequibo Islands-West Demerara
Artibeus lituratus|ABGYF157-06|ROM 113710|Guyana.Demerara-Mahaica
Artibeus lituratus|ABGYE864-06|ROM 113480|Guyana.Upper Takutu-Upper Essequibo
Artibeus lituratus|ABGYE817-06|ROM 113423|Guyana.Upper Demerara-Berbice
Artibeus lituratus|ABGYE797-06|ROM 113399|Guyana.Upper Demerara-Berbice
Artibeus lituratus|ABGYE441-06|ROM 111902|Guyana.Potaro-Siparuni
Artibeus lituratus|ABGYE311-06|ROM 111761|Guyana.Potaro-Siparuni
Artibeus lituratus|ABGYE019-06|ROM 109303|Guyana.Potaro-Siparuni
[illegible]

Artibeus lituratus|ABGYE441-06|ROM 111902|Guyana.Potaro-Siparuni
Artibeus lituratus|ABGYE311-06|ROM 111761|Guyana.Potaro-Siparuni
Artibeus lituratus|ABGYE019-06|ROM 109303|Guyana.Potaro-Siparuni
Artibeus lituratus|ABGYE018-06|ROM 109302|Guyana.Potaro-Siparuni
Artibeus lituratus|ABGYD803-06|ROM 109226|Guyana.Potaro-Siparuni
Artibeus lituratus|ABGYD584-06|ROM 109004|Guyana.Potaro-Siparuni
Artibeus lituratus|ABGYD583-06|ROM 109003|Guyana.Potaro-Siparuni
Artibeus lituratus|ABGYD543-06|ROM 108903|Guyana.Potaro-Siparuni
Artibeus lituratus|ABGYD498-06|ROM 108856|Guyana.Potaro-Siparuni
Artibeus lituratus|ABGYD429-06|ROM 108779|Guyana.Potaro-Siparuni
Artibeus lituratus|ABGYD083-06|ROM 108380|Guyana.Potaro-Siparuni
Artibeus lituratus|ABGYC654-06|ROM 107473|Guyana.Potaro-Siparuni
Artibeus lituratus|ABGYC493-06|ROM 107326|Guyana.Potaro-Siparuni
Artibeus lituratus|ABGYC492-06|ROM 107325|Guyana.Potaro-Siparuni
Artibeus lituratus|ABGYC338-06|ROM 107150|Guyana.Potaro-Siparuni
Artibeus lituratus|ABGYB899-06|ROM 104744|Guyana.Potaro-Siparuni
Artibeus lituratus|ABGYB873-06|ROM 104717|Guyana.Potaro-Siparuni
Artibeus lituratus|ABGYB677-06|ROM 103441|Guyana.Upper Demerara-Berbice
Artibeus lituratus|ABGYB675-06|ROM 103438|Guyana.Upper Demerara-Berbice
Artibeus lituratus|ABGYB168-06|ROM 101159|Guyana.Barima-Waini
Artibeus lituratus|ABGYB157-06|ROM 101146|Guyana.Barima-Waini
Artibeus lituratus|ABGYB133-06|ROM 101115|Guyana.Barima-Waini
Artibeus lituratus|ABGYB060-06|ROM 101036|Guyana.Barima-Waini
Artibeus lituratus|ABGYC108-06|ROM 106618|Guyana.Upper Takutu-Upper Essequibo
Artibeus lituratus|ABGYA880-06|ROM 100958|Guyana.Barima-Waini
Artibeus lituratus|ABGYA877-06|ROM 100953|Guyana.Barima-Waini
Artibeus lituratus|ABGYA867-06|ROM 100940|Guyana.Barima-Waini
Artibeus lituratus|ABGYB229-06|ROM 102931|Guyana.Upper Takutu-Upper Essequibo
Artibeus lituratus|ABGYA866-06|ROM 100939|Guyana.Barima-Waini
Artibeus lituratus|ABGYB682-06|ROM 103446|Guyana.Upper Demerara-Berbice
Artibeus lituratus|ABMXA857-06|ROM FN30604|Mexico.Campeche
Artibeus lituratus|ABCSA467-06|ROM 99626|Guatemala.Peten
Artibeus lituratus|ABGYD272-06|ROM 108608|Guyana.Potaro-Siparuni
Artibeus lituratus|ABSA118-06|ROM 111166|Brazil.Sao Paulo
Artibeus lituratus|BCBN727-05|ROM 104408|Ecuador.Napo
Artibeus lituratus|ABECB073-08|ROM 118735|Ecuador
Artibeus lituratus|ABGYA784-06|ROM 100836|Guyana.Barima-Waini
Artibeus lituratus|ABGYB173-06|ROM 101164|Guyana.Barima-Waini
Artibeus lituratus|ABGYA738-06|ROM 100443|Guyana.East Berbice-Corentyne
Artibeus lituratus|ABGYG771-08|ROM 119199|Guyana
Artibeus lituratus|ABGYD343-06|ROM 108684|Guyana.Potaro-Siparuni
Artibeus lituratus|ABSMS286-06|ROM 117448|Suriname.Sipaliwini
Artibeus lituratus|ABECA484-06|ROM 105508|Ecuador.Napo
Artibeus lituratus|ABGYD275-06|ROM 108611|Guyana.Potaro-Siparuni
Artibeus lituratus|ABGYC819-06|ROM 108224|Guyana.Cuyuni-Mazaruni
Artibeus lituratus|ABGYB071-06|ROM 101047|Guyana.Barima-Waini
Artibeus lituratus|ABGYA746-06|ROM 100452|Guyana.East Berbice-Corentyne
Artibeus lituratus|ABGYD700-06|ROM 109113|Guyana.Potaro-Siparuni
Artibeus lituratus|ABECA329-06|ROM 105212|Ecuador.Napo
Artibeus lituratus|ABGYE580-06|ROM 112057|Guyana.Potaro-Siparuni
Artibeus lituratus|ABSA071-06|ROM 111089|Brazil.Sao Paulo
Artibeus lituratus|ABGYB025-06|ROM 100996|Guyana.Barima-Waini
Artibeus lituratus|ABGYB330-06|ROM 103037|Guyana.Upper Takutu-Upper Essequibo
Artibeus lituratus|ABSA094-06|ROM 111126|Brazil.Sao Paulo
Artibeus lituratus|ABGYB725-06|ROM 103491|Guyana.Upper Demerara-Berbice
Artibeus lituratus|ABGYE041-06|ROM 109327|Guyana.Potaro-Siparuni
Artibeus lituratus|ABSRA335-06|ROM 116924|Suriname.Nickerie
Artibeus lituratus|BCBNT359-06|ROM 108986|Guyana.Potaro-Siparuni
Artibeus lituratus|ABGYC494-06|ROM 107327|Guyana.Potaro-Siparuni
Artibeus lituratus|ABGYC776-06|ROM F43208|Guyana.Cuyuni-Mazaruni
Artibeus lituratus|ABGYC651-06|ROM 107469|Guyana.Potaro-Siparuni
Artibeus lituratus|ABGYE551-06|ROM 112024|Guyana.Potaro-Siparuni
Artibeus lituratus|ABSA085-06|ROM 111117|Brazil.Sao Paulo
Artibeus lituratus|ABSA119-06|ROM 111167|Brazil.Sao Paulo
Artibeus lituratus|ABGYB416-06|ROM 103123|Guyana.Upper Takutu-Upper Essequibo
Artibeus lituratus|ABMXB751-06|ROM 95689|Mexico.Campeche
Artibeus lituratus|ABMXC550-06|ROM 97478|Mexico.Quintana Roo
Artibeus lituratus|ABGYA488-06|ROM 98898|Guyana.Barima-Waini
Artibeus lituratus|ABGYB106-06|ROM 101084|Guyana.Barima-Waini
Artibeus lituratus|ABGYA535-06|ROM 100205|Guyana.East Berbice-Corentyne
Artibeus lituratus|BCBNT522-06|ROM 111874|Guyana.Potaro-Siparuni
Artibeus lituratus|BCBNT088-06|ROM 107080|Guyana.Potaro-Siparuni
Artibeus lituratus|ABSRA522-06|ROM 117112|Suriname.Nickerie
Artibeus lituratus|ABGYD631-06|ROM 109029|Guyana.Potaro-Siparuni
Artibeus lituratus|ABGYD305-06|ROM 108644|Guyana.Potaro-Siparuni
Artibeus lituratus|ABGYG1021-08|ROM 118945|Guyana
Artibeus lituratus|ABGYD271-06|ROM 108607|Guyana.Potaro-Siparuni
Artibeus lituratus|ABGYD114-06|ROM 108412|Guyana.Potaro-Siparuni
Artibeus lituratus|ABGYC480-06|ROM 107313|Guyana.Potaro-Siparuni
Artibeus lituratus|ABGYC501-06|ROM 107336|Guyana.Potaro-Siparuni
Artibeus lituratus|ABGYC731-06|ROM 108129|Guyana.Cuyuni-Mazaruni
Artibeus lituratus|ABGYB684-06|ROM 103448|Guyana.Upper Demerara-Berbice
Artibeus lituratus|ABGYD760-06|ROM 109180|Guyana.Potaro-Siparuni
Artibeus lituratus|ABGYA834-06|ROM 100897|Guyana.Barima-Waini
Artibeus lituratus|ABGYB179-06|ROM 101172|Guyana.Barima-Waini
Artibeus lituratus|BCBNT366-06|ROM 109002|Guyana.Potaro-Siparuni
Artibeus lituratus|BCBNT638-06|ROM 113583|Guyana.Upper Takutu-Upper Essequibo
Artibeus lituratus|ABSRA457-06|ROM 117047|Suriname.Nickerie
Artibeus lituratus|ABGYD482-06|ROM 108835|Guyana.Potaro-Siparuni
Artibeus lituratus|ABGYE782-06|ROM 113382|Guyana.Upper Demerara-Berbice
Artibeus intermedius|ABMXB709-06|ROM 95483|Mexico.Campeche
Artibeus intermedius|ABMXC316-06|ROM 96513|Mexico.Yucatan
Artibeus intermedius|ABMXC447-06|ROM 96935|Mexico.Quintana Roo
Artibeus lituratus|ABMXA858-06|ROM FN30605|Mexico.Campeche
Artibeus intermedius|ABMXC462-06|ROM 96990|Mexico.Campeche
Artibeus lituratus|ABMXA809-06|ROM FN30519|Mexico.Campeche
Artibeus lituratus|ABMXA811-06|ROM FN30521|Mexico.Campeche
Artibeus intermedius|ABMXC542-06|ROM 97470|Mexico.Quintana Roo
Artibeus lituratus|ABGYD694-06|ROM 109107|Guyana.Potaro-Siparuni
Artibeus lituratus|ABMXA885-06|ROM FN30648|Mexico.Campeche
Artibeus lituratus|ABECA133-06|ROM 104431|Ecuador.Napo
Artibeus lituratus|ABGYE779-06|ROM 113380|Guyana.Upper Demerara-Berbice
Artibeus lituratus|ABSRA472-06|ROM 117062|Suriname.Nickerie

Artibeus lituratus|ABECA133-06|ROM 104431|Ecuador.Napo
Artibeus lituratus|ABGYE779-06|ROM 113380|Guyana.Upper Demerara-Berbice
Artibeus lituratus|ABSRA472-06|ROM 117062|Suriname.Nickerie
Artibeus lituratus|ABGYB875-06|ROM 104719|Guyana.Potaro-Siparuni
Artibeus lituratus|BCBN498-05|ROM 97488|Mexico.Quintana Roo
Artibeus intermedius|ABMXB736-06|ROM 95644|Mexico.Campeche
Artibeus intermedius|ABMXC461-06|ROM 96989|Mexico.Campeche
Artibeus intermedius|ABMXC625-06|ROM 97724|Mexico.Campeche
Artibeus intermedius|BCBN467-05|ROM 96958|Mexico.Campeche
Artibeus intermedius|BCBN259-05|ROM 99561|Guatemala.Peten
Artibeus intermedius|BCBN211-05|ROM 99576|Guatemala.Peten
Artibeus lituratus|ABSCA061-06|ROM F38092|Panama
Artibeus lituratus|ABMXC548-06|ROM 97476|Mexico.Quintana Roo
Artibeus intermedius|ABMXC545-06|ROM 97473|Mexico.Quintana Roo
Artibeus intermedius|ABMXC469-06|ROM 97000|Mexico.Campeche
Artibeus intermedius|ABMXC452-06|ROM 96962|Mexico.Campeche
Artibeus intermedius|ABMXC451-06|ROM 96961|Mexico.Campeche
Artibeus intermedius|ABMXC315-06|ROM 96512|Mexico.Yucatan
Artibeus intermedius|ABMXC266-06|ROM 96365|Mexico.Campeche
Artibeus intermedius|ABMXB771-06|ROM 95733|Mexico.Campeche
Artibeus intermedius|ABMXB735-06|ROM 95619|Mexico.Campeche
Artibeus intermedius|ABMXB706-06|ROM 95476|Mexico.Campeche
Artibeus lituratus|ABMXB648-06|ROM 95277|Mexico.Campeche
Artibeus lituratus|ABMXA886-06|ROM FN30649|Mexico.Campeche
Artibeus lituratus|ABMXA837-06|ROM FN30571|Mexico.Campeche
Artibeus lituratus|ABMXA810-06|ROM FN30520|Mexico.Campeche
Artibeus lituratus|ABMXA747-06|ROM FN30419|Mexico.Yucatan
Artibeus lituratus|ABMXA746-06|ROM FN30418|Mexico.Yucatan
Artibeus lituratus|ABGYF124-06|ROM 113670|Guyana.Demerara-Mahaica
Artibeus intermedius|ABMXB653-06|ROM 95342|Mexico.Campeche
Artibeus lituratus|ABMXB654-06|ROM 95343|Mexico.Campeche
Artibeus lituratus|ABGYD778-06|ROM 109199|Guyana.Potaro-Siparuni
Artibeus lituratus|ABGYB635-06|ROM 103390|Guyana.Upper Demerara-Berbice
Artibeus intermedius|ABMXB769-06|ROM 95731|Mexico.Campeche
Artibeus intermedius|BCBN260-05|ROM 99562|Guatemala.Peten
Artibeus intermedius|BCBN468-05|ROM 96959|Mexico.Campeche
Artibeus lituratus|ABCSA583-06|ROM 99798|Guatemala.Solola
Artibeus intermedius|ABCSA431-06|ROM 99569|Guatemala.Peten
Artibeus lituratus|ABCSA430-06|ROM 99568|Guatemala.Peten
Artibeus intermedius|ABMXC507-06|ROM 97423|Mexico.Quintana Roo
Artibeus lituratus|ABCSA225-06|ROM 98501|Guatemala.El Progreso
Artibeus lituratus|ABMXA861-06|ROM FN30608|Mexico.Campeche
Artibeus lituratus|ABCSA300-06|ROM 99276|Guatemala.Peten
Artibeus lituratus|ABMXB655-06|ROM 95344|Mexico.Campeche
Artibeus intermedius|ABMXB711-06|ROM 95485|Mexico.Campeche
Artibeus intermedius|ABMXC220-06|ROM 96243|Mexico.Tabasco
Artibeus intermedius|ABMXC481-06|ROM 97044|Mexico.Campeche
Artibeus lituratus|ABCSA227-06|ROM 98503|Guatemala.El Progreso
Artibeus lituratus|ABGYC482-06|ROM 107315|Guyana.Potaro-Siparuni
Artibeus intermedius|ABMXC623-06|ROM 97722|Mexico.Campeche
Artibeus lituratus|ABCSA437-06|ROM 99578|Guatemala.Peten
Artibeus lituratus|BCBN499-05|ROM 97489|Mexico.Quintana Roo
Artibeus lituratus|BCBN641-05|ROM 104253|Panama
Artibeus lituratus|BCBN642-05|ROM 104254|Panama
Artibeus lituratus|ABGYB674-06|ROM 103437|Guyana.Upper Demerara-Berbice
Artibeus lituratus|BCBNT645-06|ROM 113604|Guyana.Upper Takutu-Upper Essequibo
Artibeus lituratus|ABGYC775-06|ROM F43207|Guyana.Cuyuni-Mazaruni
Artibeus lituratus|ABGYB129-06|ROM 101111|Guyana.Barima-Waini
Artibeus lituratus|ABGYD307-06|ROM 108646|Guyana.Potaro-Siparuni
Artibeus lituratus|ABGYE017-06|ROM 109301|Guyana.Potaro-Siparuni
Artibeus lituratus|ABGYE838-06|ROM 113450|Guyana.Upper Demerara-Berbice
Artibeus lituratus|ABGYD407-06|ROM 108753|Guyana.Potaro-Siparuni
Artibeus lituratus|ABGYD529-06|ROM 108887|Guyana.Potaro-Siparuni
Artibeus lituratus|ABGYD615-06|ROM 108985|Guyana.Potaro-Siparuni
Artibeus lituratus|ABGYE193-06|ROM 111639|Guyana.Potaro-Siparuni
Artibeus lituratus|ABGYE182-06|ROM 111625|Guyana.Potaro-Siparuni
Artibeus lituratus|ABGYD569-06|ROM 108945|Guyana.Potaro-Siparuni
Artibeus lituratus|ABGYC774-06|ROM 108179|Guyana.Cuyuni-Mazaruni
Artibeus lituratus|ABGYB062-06|ROM 101038|Guyana.Barima-Waini
Artibeus lituratus|ABGYD787-06|ROM 109209|Guyana.Potaro-Siparuni
Artibeus lituratus|ABGYC490-06|ROM 107323|Guyana.Potaro-Siparuni
Artibeus lituratus|ABGYA813-06|ROM 100872|Guyana.Barima-Waini
Artibeus lituratus|ABGYB180-06|ROM 101173|Guyana.Barima-Waini
Artibeus lituratus|ABGYC406-06|ROM 107225|Guyana.Potaro-Siparuni
Artibeus lituratus|ABGYA590-06|ROM 100275|Guyana.East Berbice-Corentyne
Artibeus lituratus|ABGYD567-06|ROM 108943|Guyana.Potaro-Siparuni
Artibeus lituratus|ABECB077-08|ROM 118739|Ecuador
Artibeus lituratus|ABGYG1015-08|ROM 118939|Guyana
Artibeus lituratus|BCBNT641-06|ROM 113591|Guyana.Upper Takutu-Upper Essequibo
Artibeus intermedius|BCBN212-05|ROM 99577|Guatemala.Peten
Artibeus lituratus|ABGYE810-06|ROM 113413|Guyana.Upper Demerara-Berbice
Artibeus lituratus|ABGYG773-08|ROM 119201|Guyana
Artibeus lituratus|ABGYE192-06|ROM 111638|Guyana.Potaro-Siparuni
Artibeus lituratus|ABGYE429-06|ROM 111888|Guyana.Potaro-Siparuni
Artibeus lituratus|ABGYE048-06|ROM 109334|Guyana.Potaro-Siparuni
Artibeus lituratus|ABGYD695-06|ROM 109108|Guyana.Potaro-Siparuni
Artibeus lituratus|ABGYD320-06|ROM 108660|Guyana.Potaro-Siparuni
Artibeus lituratus|ABGYC483-06|ROM 107316|Guyana.Potaro-Siparuni
Artibeus lituratus|ABGYD084-06|ROM 108381|Guyana.Potaro-Siparuni
Artibeus lituratus|ABGYC803-06|ROM F43240|Guyana.Cuyuni-Mazaruni
Artibeus lituratus|ABGYE432-06|ROM 111891|Guyana.Potaro-Siparuni
Artibeus lituratus|ABGYD274-06|ROM 108610|Guyana.Potaro-Siparuni
Artibeus lituratus|ABGYC136-06|ROM 106648|Guyana.Upper Takutu-Upper Essequibo
Artibeus lituratus|ABCSA434-06|ROM 99572|Guatemala.Peten
Artibeus lituratus|ABGYB678-06|ROM 103442|Guyana.Upper Demerara-Berbice
Artibeus lituratus|ABGYD614-06|ROM 108984|Guyana.Potaro-Siparuni
Artibeus lituratus|ABGYD720-06|ROM 109135|Guyana.Potaro-Siparuni
Artibeus lituratus|ABSRA337-06|ROM 116926|Suriname.Nickerie
Artibeus lituratus|ABGYB167-06|ROM 101158|Guyana.Barima-Waini
Artibeus lituratus|ABGYA565-06|ROM 100241|Guyana.East Berbice-Corentyne
Artibeus lituratus|ABGYC481-06|ROM 107314|Guyana.Potaro-Siparuni
Artibeus lituratus|ABGYB103-06|ROM 101081|Guyana.Barima-Waini
Artibeus lituratus|ABECA033-06|ROM 104014|Ecuador.Napo

Artibeus lituratus|ABGYC481-06|ROM 107314|Guyana.Potaro-Siparuni
Artibeus lituratus|ABGYB103-06|ROM 101081|Guyana.Barima-Waini
Artibeus lituratus|ABECA033-06|ROM 104014|Ecuador.Napo
Artibeus lituratus|BCBN970-05|ROM 106013|Ecuador.Napo
Artibeus lituratus|BCBNT396-06|ROM 109267|Guyana.Potaro-Siparuni
Artibeus lituratus|ABGYD306-06|ROM 108645|Guyana.Potaro-Siparuni
Artibeus lituratus|ABGYD719-06|ROM 109134|Guyana.Potaro-Siparuni
Artibeus lituratus|ABCSA346-06|ROM 99328|Guatemala.Peten
Artibeus lituratus|ABGYE439-06|ROM 111900|Guyana.Potaro-Siparuni
Artibeus lituratus|ABGYE347-06|ROM 111797|Guyana.Potaro-Siparuni
Artibeus intermedius|ABMXC440-06|ROM 97383|Mexico.Quintana Roo
Artibeus lituratus|BCBNT093-06|ROM 107093|Guyana.Potaro-Siparuni
Artibeus lituratus|ABGYB722-06|ROM 103488|Guyana.Upper Demerara-Berbice
Artibeus lituratus|ABGYE043-06|ROM 109329|Guyana.Potaro-Siparuni
Artibeus lituratus|ABGYB182-06|ROM 101175|Guyana.Barima-Waini
Artibeus lituratus|ABSMS285-06|ROM 117447|Suriname.Sipaliwini
Artibeus lituratus|ABGYB496-06|ROM 103231|Guyana.Upper Takutu-Upper Essequibo
Artibeus lituratus|ABGYE821-06|ROM 113427|Guyana.Upper Demerara-Berbice
Artibeus lituratus|ABSRA381-06|ROM 116970|Suriname.Nickerie
Artibeus intermedius|ABMXC498-06|ROM 97410|Mexico.Quintana Roo
Artibeus lituratus|ABSRA380-06|ROM 116969|Suriname.Nickerie
Artibeus lituratus|ABMXB712-06|ROM 95486|Mexico.Campeche
Artibeus lituratus|ABCSA513-06|ROM 99525|Guatemala.Peten
Artibeus lituratus|ABMXC552-06|ROM 97480|Mexico.Quintana Roo
Artibeus lituratus|ABMXB710-06|ROM 95484|Mexico.Campeche
Artibeus lituratus|BCBNT414-06|ROM 108294|Costa Rica.Limon
Artibeus lituratus|ABGYB711-06|ROM 103476|Guyana.Upper Demerara-Berbice
Artibeus lituratus|ABCSA435-06|ROM 99573|Guatemala.Peten
Artibeus lituratus|ABCSA510-06|ROM 99521|Guatemala.Peten
Artibeus lituratus|ABMXA867-06|ROM FN30614|Mexico.Campeche
Artibeus intermedius|ABMXC463-06|ROM 96991|Mexico.Campeche
Artibeus lituratus|BCBN472-05|ROM 96999|Mexico.Campeche
Artibeus lituratus|BCBN121-05|ROM 97857|Guyana.Upper Takutu-Upper Essequibo
Artibeus lituratus|ABSRA336-06|ROM 116925|Suriname.Nickerie
Artibeus lituratus|ABSRA312-06|ROM 116901|Suriname.Nickerie
Artibeus lituratus|ABSMS356-06|ROM 117518|Suriname.Sipaliwini
Artibeus intermedius|ABMXC543-06|ROM 97471|Mexico.Quintana Roo
Artibeus lituratus|ABMXB704-06|ROM 95474|Mexico.Campeche
Artibeus lituratus|ABGYF148-06|ROM 113701|Guyana.Demerara-Mahaica
Artibeus lituratus|ABGYE369-06|ROM 111819|Guyana.Potaro-Siparuni
Artibeus lituratus|ABGYD742-06|ROM 109160|Guyana.Potaro-Siparuni
Artibeus lituratus|ABGYD687-06|ROM 109099|Guyana.Potaro-Siparuni
Artibeus lituratus|ABGYD590-06|ROM 109010|Guyana.Potaro-Siparuni
Artibeus lituratus|ABGYD481-06|ROM 108834|Guyana.Potaro-Siparuni
Artibeus lituratus|ABGYD427-06|ROM 108777|Guyana.Potaro-Siparuni
Artibeus lituratus|ABGYD289-06|ROM 108628|Guyana.Potaro-Siparuni
Artibeus lituratus|ABGYC491-06|ROM 107324|Guyana.Potaro-Siparuni
Artibeus lituratus|ABGYB908-06|ROM 104753|Guyana.Potaro-Siparuni
Artibeus lituratus|ABGYB646-06|ROM 103403|Guyana.Upper Demerara-Berbice
Artibeus lituratus|ABGYE550-06|ROM 112023|Guyana.Potaro-Siparuni
Artibeus lituratus|ABGYB676-06|ROM 103439|Guyana.Upper Demerara-Berbice
Artibeus lituratus|ABGYB544-06|ROM 103279|Guyana.Upper Takutu-Upper Essequibo
Artibeus lituratus|ABGYD443-06|ROM 108794|Guyana.Potaro-Siparuni
Artibeus lituratus|ABGYA622-06|ROM 100316|Guyana.East Berbice-Corentyne
Artibeus lituratus|ABGYB155-06|ROM 101144|Guyana.Barima-Waini
Artibeus lituratus|ABGYB636-06|ROM 103391|Guyana.Upper Demerara-Berbice
Artibeus lituratus|ABGYA819-06|ROM 100878|Guyana.Barima-Waini
Artibeus lituratus|ABGYB681-06|ROM 103445|Guyana.Upper Demerara-Berbice
Artibeus lituratus|ABGYA878-06|ROM 100954|Guyana.Barima-Waini
Artibeus lituratus|ABGYC502-06|ROM 107337|Guyana.Potaro-Siparuni
Artibeus intermedius|ABCSA436-06|ROM 99574|Guatemala.Peten
Artibeus lituratus|ABCSA345-06|ROM 99327|Guatemala.Peten
Artibeus lituratus|ABGYB181-06|ROM 101174|Guyana.Barima-Waini
Artibeus lituratus|ABMXA786-06|ROM FN30475|Mexico.Campeche
Artibeus lituratus|ABECA322-06|ROM 105202|Ecuador.Napo
Artibeus intermedius|ABMXB707-06|ROM 95481|Mexico.Campeche
Artibeus lituratus|ABCSA432-06|ROM 99570|Guatemala.Peten
Artibeus intermedius|ABMXC621-06|ROM 97720|Mexico.Campeche
Artibeus lituratus|ABGYC404-06|ROM 107223|Guyana.Potaro-Siparuni
Artibeus lituratus|ABGYB633-06|ROM 103388|Guyana.Upper Demerara-Berbice
Artibeus lituratus|BCBNT735-06|ROM 113910|Suriname.Brokopondo
Artibeus intermedius|ABMXC554-06|ROM 97485|Mexico.Quintana Roo
Artibeus lituratus|ABSA107-06|ROM 111147|Brazil.Sao Paulo
Artibeus lituratus|ABSA114-06|ROM 111161|Brazil.Sao Paulo
Artibeus lituratus|ABMXA748-06|ROM FN30420|Mexico.Yucatan
Artibeus intermedius|ABMXB671-06|ROM FN29168|Mexico.Campeche
Artibeus intermedius|ABMXC624-06|ROM 97723|Mexico.Campeche
Artibeus intermedius|ABMXC455-06|ROM 96965|Mexico.Campeche
Artibeus intermedius|ABMXC506-06|ROM 97422|Mexico.Quintana Roo
Artibeus intermedius|ABMXB647-06|ROM 95276|Mexico.Campeche
Artibeus lituratus|ABMXA888-06|ROM FN30651|Mexico.Campeche
Artibeus intermedius|ABMXB713-06|ROM 95487|Mexico.Campeche
Artibeus intermedius|BCBN381-05|ROM 101235|El Salvador.Ahuachapan
Artibeus lituratus|ABCSA224-06|ROM FN31489|Guatemala.El Progreso
Artibeus intermedius|ABMXB774-06|ROM 95736|Mexico.Campeche
Artibeus lituratus|ABMXC649-06|ROM 97680|Mexico.Chiapas
Artibeus lituratus|ABMXC648-06|ROM 97679|Mexico.Chiapas
Artibeus intermedius|ABMXB673-06|ROM 95379|Mexico.Campeche
Artibeus intermedius|ABMXC446-06|ROM 96934|Mexico.Quintana Roo
Artibeus intermedius|ABMXC546-06|ROM 97474|Mexico.Quintana Roo
Artibeus intermedius|ABMXC646-06|ROM 97677|Mexico.Chiapas
Artibeus lituratus|ABMXC647-06|ROM 97678|Mexico.Chiapas
Artibeus intermedius|ABMXC547-06|ROM 97475|Mexico.Quintana Roo
Artibeus lituratus|ABMXA834-06|ROM FN30568|Mexico.Campeche
Artibeus intermedius|ABMXC260-06|ROM FN30173|Mexico.Campeche
Artibeus intermedius|ABMXC479-06|ROM 97042|Mexico.Campeche
Artibeus intermedius|ABMXC544-06|ROM 97472|Mexico.Quintana Roo
Artibeus intermedius|ABMXC270-06|ROM 96369|Mexico.Campeche
Artibeus lituratus|ABMXA859-06|ROM FN30606|Mexico.Campeche
Artibeus lituratus|ABMXA828-06|ROM FN30559|Mexico.Campeche
Artibeus lituratus|ABMXA758-06|ROM FN30429|Mexico.Yucatan
Artibeus intermedius|ABMXC318-06|ROM 96515|Mexico.Yucatan
Artibeus intermedius|ABMXC449-06|ROM 96937|Mexico.Quintana Roo

[illegible]
Artibeus lituratus|ABMXA758-06|ROM FN30429|Mexico.Yucatan
Artibeus intermedius|ABMXC318-06|ROM 96515|Mexico.Yucatan
Artibeus intermedius|ABMXC449-06|ROM 96937|Mexico.Quintana Roo
Artibeus intermedius|ABMXC268-06|ROM 96367|Mexico.Campeche
Artibeus lituratus|ABMXA829-06|ROM FN30560|Mexico.Campeche
Artibeus intermedius|BCBN210-05|ROM 99575|Guatemala.Peten
Artibeus intermedius|BCBN391-05|ROM 101262|El Salvador.Ahuachapan
Artibeus intermedius|ABMXB669-06|ROM 95371|Mexico.Campeche
Artibeus lituratus|ABMXA832-06|ROM FN30566|Mexico.Campeche
Artibeus intermedius|ABMXB705-06|ROM 95475|Mexico.Campeche
Artibeus intermedius|ABMXC314-06|ROM 96511|Mexico.Yucatan
Artibeus lituratus|ABMXA826-06|ROM FN30557|Mexico.Campeche
Artibeus lituratus|ABMXA860-06|ROM FN30607|Mexico.Campeche
Artibeus intermedius|ABMXC219-06|ROM 96242|Mexico.Tabasco
Artibeus lituratus|ABMXA814-06|ROM FN30524|Mexico.Campeche
Artibeus lituratus|ABMXA827-06|ROM FN30558|Mexico.Campeche
Artibeus intermedius|ABMXB773-06|ROM 95735|Mexico.Campeche
Artibeus intermedius|ABMXB767-06|ROM 95729|Mexico.Campeche
Artibeus lituratus|ABCSA226-06|ROM 98502|Guatemala.El Progreso
Artibeus intermedius|ABMXC555-06|ROM 97487|Mexico.Quintana Roo
Artibeus lituratus|ABMXA889-06|ROM FN30652|Mexico.Campeche
Artibeus intermedius|ABMXC263-06|ROM 96360|Mexico.Campeche
Artibeus intermedius|ABMXB749-06|ROM 95687|Mexico.Campeche
Artibeus intermedius|ABMXC474-06|ROM 97037|Mexico.Campeche
Artibeus intermedius|ABMXC448-06|ROM 96936|Mexico.Quintana Roo
Artibeus intermedius|BCBN392-05|ROM 101263|El Salvador.Ahuachapan
Artibeus lituratus|ABMXA833-06|ROM FN30567|Mexico.Campeche
Artibeus lituratus|ABMXA883-06|ROM FN30646|Mexico.Campeche
Artibeus intermedius|ABMXC269-06|ROM 96368|Mexico.Campeche
Artibeus intermedius|ABMXB770-06|ROM 95732|Mexico.Campeche
Artibeus intermedius|ABMXC645-06|ROM 97676|Mexico.Chiapas
Artibeus intermedius|BCBN471-05|ROM 96988|Mexico.Campeche
Artibeus lituratus|ABGYE049-06|ROM 109335|Guyana.Potaro-Siparuni
Artibeus lituratus|ABECA684-06|ROM 105744|Ecuador.Napo
Artibeus lituratus|ABCSA263-06|ROM 99224|Guatemala.Peten
Artibeus lituratus|ABGYE057-06|ROM 109343|Guyana.Potaro-Siparuni
Artibeus lituratus|ABGYB645-06|ROM 103402|Guyana.Upper Demerara-Berbice
Artibeus lituratus|ABSRA360-06|ROM 116949|Suriname.Nickerie
Artibeus lituratus|ABGYA837-06|ROM 100900|Guyana.Barima-Waini
Artibeus lituratus|ABGYB318-06|ROM 103023|Guyana.Upper Takutu-Upper Essequibo
Artibeus lituratus|ABGYE418-06|ROM 111875|Guyana.Potaro-Siparuni
Artibeus lituratus|ABGYE341-06|ROM 111791|Guyana.Potaro-Siparuni
Artibeus lituratus|ABGYE300-06|ROM 111750|Guyana.Potaro-Siparuni
Artibeus lituratus|ABGYE132-06|ROM 111575|Guyana.Potaro-Siparuni
Artibeus lituratus|ABGYD595-06|ROM 109017|Guyana.Potaro-Siparuni
Artibeus lituratus|ABGYC467-06|ROM 107297|Guyana.Potaro-Siparuni
Artibeus lituratus|ABGYE118-06|ROM 111561|Guyana.Potaro-Siparuni
Artibeus lituratus|ABGYC466-06|ROM 107296|Guyana.Potaro-Siparuni
Artibeus lituratus|ABGYB059-06|ROM 101035|Guyana.Barima-Waini
Artibeus lituratus|ABGYD304-06|ROM 108643|Guyana.Potaro-Siparuni
Artibeus lituratus|ABGYD409-06|ROM 108755|Guyana.Potaro-Siparuni
Artibeus lituratus|ABGYB626-06|ROM 103381|Guyana.Upper Demerara-Berbice
Artibeus lituratus|ABSMS231-06|ROM 117393|Suriname.Sipaliwini
Artibeus lituratus|ABGYD630-06|ROM 109028|Guyana.Potaro-Siparuni
Artibeus lituratus|ABSA090-06|ROM 111122|Brazil.Sao Paulo
Artibeus lituratus|ABGYE611-06|ROM 112090|Guyana.Potaro-Siparuni
Artibeus lituratus|BCBN258-05|ROM 99560|Guatemala.Peten
Artibeus lituratus|ABGYE342-06|ROM 111792|Guyana.Potaro-Siparuni
Artibeus lituratus|ABGYD143-06|ROM 108449|Guyana.Potaro-Siparuni
Artibeus lituratus|ABGYD276-06|ROM 108612|Guyana.Potaro-Siparuni
Artibeus lituratus|ABGYE798-06|ROM 113400|Guyana.Upper Demerara-Berbice
Artibeus lituratus|ABGYC465-06|ROM 107295|Guyana.Potaro-Siparuni
Artibeus lituratus|ABGYB930-06|ROM 104775|Guyana.Potaro-Siparuni
Artibeus lituratus|ABGYB624-06|ROM 103379|Guyana.Upper Demerara-Berbice
Artibeus lituratus|ABGYD168-06|ROM 108488|Guyana.Potaro-Siparuni
Artibeus lituratus|ABGYB072-06|ROM 101048|Guyana.Barima-Waini
Artibeus lituratus|ABSRA362-06|ROM 116951|Suriname.Nickerie
Artibeus lituratus|ABGYB935-07|ROM 103440|Guyana.Upper Demerara-Berbice
Artibeus lituratus|BCBN263-05|ROM 99567|Guatemala.Peten
Artibeus lituratus|ABGYD414-06|ROM 108761|Guyana.Potaro-Siparuni
Artibeus lituratus|ABGYE040-06|ROM 109326|Guyana.Potaro-Siparuni
Artibeus lituratus|ABMXB716-06|ROM 95490|Mexico.Campeche
Artibeus lituratus|ABMXA743-06|ROM FN30400|Mexico.Yucatan
Artibeus lituratus|ABGYG838-08|ROM 119266|Guyana
Artibeus lituratus|ABGYG057-06|ROM 115570|Guyana.Essequibo Islands-West Demerara
Artibeus lituratus|ABGYE713-06|ROM 112648|Guyana.Demerara-Mahaica
Artibeus lituratus|ABGYE204-06|ROM 111650|Guyana.Potaro-Siparuni
Artibeus lituratus|ABGYD773-06|ROM 109193|Guyana.Potaro-Siparuni
Artibeus lituratus|ABGYD629-06|ROM 109027|Guyana.Potaro-Siparuni
Artibeus lituratus|ABGYD594-06|ROM 109016|Guyana.Potaro-Siparuni
Artibeus lituratus|ABGYD308-06|ROM 108647|Guyana.Potaro-Siparuni
Artibeus lituratus|ABGYA783-06|ROM 100835|Guyana.Barima-Waini
Artibeus lituratus|ABECA500-06|ROM 105528|Ecuador.Napo
Artibeus lituratus|ABECA486-06|ROM 105509|Ecuador.Napo
Artibeus lituratus|ABECA321-06|ROM 105201|Ecuador.Napo
Artibeus lituratus|ABCSA469-06|ROM 99628|Guatemala.Peten
Artibeus lituratus|ABECA483-06|ROM 105507|Ecuador.Napo
Artibeus lituratus|ABGYG1044-08|ROM 118968|Guyana
Artibeus lituratus|ABGYA558-06|ROM 100233|Guyana.East Berbice-Corentyne
Artibeus lituratus|ABSMS210-06|ROM 117372|Suriname.Sipaliwini
Artibeus lituratus|ABSMS353-06|ROM 117515|Suriname.Sipaliwini
Artibeus lituratus|ABGYD224-06|ROM 108555|Guyana.Potaro-Siparuni
Artibeus lituratus|ABGYA820-06|ROM 100879|Guyana.Barima-Waini
Artibeus lituratus|ABGYE762-06|ROM 113361|Guyana.Upper Demerara-Berbice
Artibeus lituratus|ABMXA863-06|ROM FN30610|Mexico.Campeche
Artibeus lituratus|ABGYB628-06|ROM 103383|Guyana.Upper Demerara-Berbice
Artibeus lituratus|ABGYE276-06|ROM 111725|Guyana.Potaro-Siparuni
Artibeus lituratus|ABGYA879-06|ROM 100957|Guyana.Barima-Waini
Artibeus lituratus|ABGYC035-06|ROM 104812|Guyana.Potaro-Siparuni
Artibeus lituratus|ABGYE370-06|ROM 111820|Guyana.Potaro-Siparuni
Artibeus lituratus|ABGYB625-06|ROM 103380|Guyana.Upper Demerara-Berbice
Artibeus lituratus|ABGYD287-06|ROM 108626|Guyana.Potaro-Siparuni
[illegible]

Artibeus lituratus|[illegible]|ROM [illegible]|Guyana.Potaro-Siparuni
Artibeus lituratus|ABGYB625-06|ROM 103380|Guyana.Upper Demerara-Berbice
Artibeus lituratus|ABGYD287-06|ROM 108626|Guyana.Potaro-Siparuni
Artibeus lituratus|ABGYC368-06|ROM 107184|Guyana.Potaro-Siparuni
Artibeus lituratus|ABGYF185-06|ROM 113746|Guyana.Demerara-Mahaica
Artibeus lituratus|ABSRA455-06|ROM 117045|Suriname.Nickerie
Artibeus lituratus|ABGYB159-06|ROM 101148|Guyana.Barima-Waini
Artibeus lituratus|ABSA075-06|ROM 111093|Brazil.Sao Paulo
Artibeus lituratus|ABSA108-06|ROM 111151|Brazil.Sao Paulo
Artibeus lituratus|BCBN787-05|ROM 105183|Ecuador.Napo
Artibeus lituratus|ABGYB648-06|ROM 103405|Guyana.Upper Demerara-Berbice
Artibeus lituratus|ABGYD564-06|ROM 108935|Guyana.Potaro-Siparuni
Artibeus lituratus|ABECA323-06|ROM 105203|Ecuador.Napo
Artibeus lituratus|ABSRA363-06|ROM 116952|Suriname.Nickerie
Artibeus lituratus|BCBNT394-06|ROM 109261|Guyana.Potaro-Siparuni
Artibeus lituratus|ABGYD568-06|ROM 108944|Guyana.Potaro-Siparuni
Artibeus lituratus|ABGYG067-06|ROM 115589|Guyana.Essequibo Islands-West Demerara
Artibeus lituratus|ABMXB715-06|ROM 95489|Mexico.Campeche
Artibeus lituratus|ABMXC549-06|ROM 97477|Mexico.Quintana Roo
Artibeus lituratus|ABGYB158-06|ROM 101147|Guyana.Barima-Waini
Artibeus intermedius|ABMXB714-06|ROM 95488|Mexico.Campeche
Artibeus lituratus|BCBNT778-06|ROM 114005|Suriname.Brokopondo
Artibeus lituratus|ABGYD444-06|ROM 108795|Guyana.Potaro-Siparuni
Artibeus lituratus|ABGYB139-06|ROM 101125|Guyana.Barima-Waini
Artibeus lituratus|ABGYB140-06|ROM 101126|Guyana.Barima-Waini
Artibeus lituratus|ABSRA382-06|ROM 116971|Suriname.Nickerie
Artibeus intermedius|ABMXC262-06|ROM 96359|Mexico.Campeche
Artibeus lituratus|ABECA683-06|ROM F37999|Ecuador.Napo
Artibeus lituratus|ABGYD225-06|ROM 108556|Guyana.Potaro-Siparuni
Artibeus intermedius|ABMXC454-06|ROM 96964|Mexico.Campeche
Artibeus lituratus|ABGYB061-06|ROM 101037|Guyana.Barima-Waini
Artibeus lituratus|ABGYB169-06|ROM 101160|Guyana.Barima-Waini
Artibeus lituratus|ABGYC760-06|ROM 108163|Guyana.Cuyuni-Mazaruni
Artibeus lituratus|ABMXC232-06|ROM 96300|Mexico.Campeche
Artibeus lituratus|ABGYD493-06|ROM 108851|Guyana.Potaro-Siparuni
Artibeus lituratus|ABCSA344-06|ROM 99326|Guatemala.Peten
Artibeus lituratus|ABGYA876-06|ROM 100952|Guyana.Barima-Waini
Artibeus lituratus|ABGYB888-06|ROM 104733|Guyana.Potaro-Siparuni
Artibeus lituratus|ABGYB615-06|ROM 103367|Guyana.Upper Demerara-Berbice
Artibeus lituratus|ABECA477-06|ROM 105500|Ecuador.Napo
Artibeus lituratus|ABGYD428-06|ROM 108778|Guyana.Potaro-Siparuni
Artibeus lituratus|ABGYA559-06|ROM 100234|Guyana.East Berbice-Corentyne
Artibeus lituratus|ABGYA859-06|ROM 100930|Guyana.Barima-Waini
Artibeus lituratus|ABCSA438-06|ROM 99579|Guatemala.Peten
Artibeus lituratus|ABECA744-06|ROM 105891|Ecuador.Napo
Artibeus lituratus|ABGYB726-06|ROM 103492|Guyana.Upper Demerara-Berbice
Artibeus lituratus|ABGYE440-06|ROM 111901|Guyana.Potaro-Siparuni
Artibeus intermedius|ABMXC317-06|ROM 96514|Mexico.Yucatan
Artibeus lituratus|ABSMS384-06|ROM 117546|Suriname.Sipaliwini
Artibeus lituratus|ABGYE442-06|ROM 111903|Guyana.Potaro-Siparuni
Artibeus lituratus|ABGYB398-06|ROM 103105|Guyana.Upper Takutu-Upper Essequibo
Artibeus lituratus|ABSMS089-06|ROM 117251|Suriname.Sipaliwini
Artibeus lituratus|BCBN510-05|ROM 97523|Mexico.Quintana Roo
Artibeus lituratus|ABGYD021-06|ROM 108242|Guyana.Cuyuni-Mazaruni
Artibeus lituratus|BCBN571-05|ROM 104015|Ecuador.Napo
Artibeus lituratus|ABGYB156-06|ROM 101145|Guyana.Barima-Waini
Artibeus lituratus|ABGYD115-06|ROM 108413|Guyana.Potaro-Siparuni
Artibeus lituratus|ABGYB170-06|ROM 101161|Guyana.Barima-Waini
Artibeus lituratus|ABGYC652-06|ROM 107470|Guyana.Potaro-Siparuni
Artibeus lituratus|ABGYG128-06|ROM 115682|Guyana.Potaro-Siparuni
Artibeus lituratus|ABGYB679-06|ROM 103443|Guyana.Upper Demerara-Berbice
Artibeus lituratus|ABGYE340-06|ROM 111790|Guyana.Potaro-Siparuni
Artibeus lituratus|ABGYA264-06|ROM 98164|Guyana.Potaro-Siparuni
Artibeus lituratus|ABGYE724-06|ROM 112659|Guyana.Demerara-Mahaica
Artibeus lituratus|ABSMS245-06|ROM 117407|Suriname.Sipaliwini
Artibeus lituratus|ABGYA887-06|ROM 100967|Guyana.Barima-Waini
Artibeus lituratus|ABGYD430-06|ROM 108780|Guyana.Potaro-Siparuni
Artibeus lituratus|BCBNT629-06|ROM 113566|Guyana.Upper Takutu-Upper Essequibo
Artibeus lituratus|ABMXC551-06|ROM 97479|Mexico.Quintana Roo
Artibeus lituratus|ABGYB634-06|ROM 103389|Guyana.Upper Demerara-Berbice
Artibeus lituratus|ABGYC405-06|ROM 107224|Guyana.Potaro-Siparuni
Artibeus lituratus|ABGYB928-06|ROM 104773|Guyana.Potaro-Siparuni
Artibeus lituratus|ABGYG023-06|ROM 115514|Guyana.Essequibo Islands-West Demerara
Artibeus lituratus|ABMXB750-06|ROM 95688|Mexico.Campeche
Artibeus intermedius|ABMXB768-06|ROM 95730|Mexico.Campeche
Artibeus intermedius|ABMXC267-06|ROM 96366|Mexico.Campeche
Artibeus lituratus|ABMXC472-06|ROM 97008|Mexico.Campeche
Artibeus lituratus|ABMXA830-06|ROM FN30563|Mexico.Campeche
Artibeus lituratus|ABGYB545-06|ROM 103280|Guyana.Upper Takutu-Upper Essequibo
Artibeus lituratus|ABGYE612-06|ROM 112091|Guyana.Potaro-Siparuni
Artibeus lituratus|BCBN693-05|ROM 104336|Panama.Darien
Artibeus lituratus|ABGYE781-06|ROM 113381|Guyana.Upper Demerara-Berbice
Artibeus lituratus|ABMXC264-06|ROM 96361|Mexico.Campeche
Artibeus lituratus|BCBN257-05|ROM 99559|Guatemala.Peten
Artibeus intermedius|ABMXC441-06|ROM 97385|Mexico.Quintana Roo
Artibeus lituratus|ABGYB673-06|ROM 103436|Guyana.Upper Demerara-Berbice
Artibeus lituratus|BCBNT360-06|ROM 108987|Guyana.Potaro-Siparuni
Artibeus lituratus|ABGYA747-06|ROM 100453|Guyana.East Berbice-Corentyne
Artibeus lituratus|ABGYB494-06|ROM 103229|Guyana.Upper Takutu-Upper Essequibo
Artibeus lituratus|ABGYC326-06|ROM 107138|Guyana.Potaro-Siparuni
Artibeus lituratus|ABGYE020-06|ROM 109304|Guyana.Potaro-Siparuni
Artibeus lituratus|ABGYE154-06|ROM 111597|Guyana.Potaro-Siparuni
Artibeus lituratus|ABGYE822-06|ROM 113428|Guyana.Upper Demerara-Berbice
Artibeus lituratus|ABGYG787-08|ROM 119215|Guyana
Artibeus lituratus|ABSMS237-06|ROM 117399|Suriname.Sipaliwini
Artibeus lituratus|ABSRA383-06|ROM 116972|Suriname.Nickerie
Artibeus lituratus|BCBNT640-06|ROM 113590|Guyana.Upper Takutu-Upper Essequibo
Artibeus lituratus|ABCSA468-06|ROM 99627|Guatemala.Peten
Artibeus lituratus|ABCSA433-06|ROM 99571|Guatemala.Peten
Artibeus lituratus|ABGYD517-06|ROM 108875|Guyana.Potaro-Siparuni
Artibeus lituratus|ABGYA802-06|ROM 100860|Guyana.Barima-Waini
Artibeus lituratus|BCBNT084-06|ROM 107072|Guyana.Potaro-Siparuni
Artibeus lituratus|ABGYD772-06|ROM 109192|Guyana.Potaro-Siparuni
[illegible]

Artibeus lituratus|ABGYA802-06|ROM 100860|Guyana.Barima-Waini
Artibeus lituratus|BCBNT084-06|ROM 107072|Guyana.Potaro-Siparuni
Artibeus lituratus|ABGYD772-06|ROM 109192|Guyana.Potaro-Siparuni
Artibeus lituratus|ABGYD609-06|ROM 108975|Guyana.Potaro-Siparuni
Artibeus intermedius|ABMXB656-06|ROM 95345|Mexico.Campeche
Artibeus intermedius|ABMXB748-06|ROM 95686|Mexico.Campeche
Artibeus intermedius|ABMXC480-06|ROM 97043|Mexico.Campeche
Artibeus intermedius|ABMXC445-06|ROM 96929|Mexico.Quintana Roo
Artibeus intermedius|ABMXC626-06|ROM 97729|Mexico.Campeche
Artibeus lituratus|ABGYE155-06|ROM 111598|Guyana.Potaro-Siparuni
Artibeus lituratus|ABMXA862-06|ROM FN30609|Mexico.Campeche
Artibeus intermedius|ABMXC622-06|ROM 97721|Mexico.Campeche
Artibeus lituratus|ABGYG1085-08|ROM 119009|Guyana
Artibeus intermedius|ABMXB731-06|ROM 95571|Mexico.Campeche
Artibeus intermedius|ABMXC502-06|ROM 97418|Mexico.Quintana Roo
Artibeus intermedius|ABMXB734-06|ROM 95618|Mexico.Campeche
Artibeus intermedius|ABMXC439-06|ROM 97382|Mexico.Quintana Roo
Artibeus intermedius|ABMXC476-06|ROM 97039|Mexico.Campeche
Artibeus intermedius|ABMXC478-06|ROM 97041|Mexico.Campeche
Artibeus lituratus|ABGYF011-06|ROM 113501|Guyana.Upper Takutu-Upper Essequibo
Artibeus lituratus|ABGYA882-06|ROM 100960|Guyana.Barima-Waini
Artibeus lituratus|ABGYE042-06|ROM 109328|Guyana.Potaro-Siparuni
Artibeus lituratus|ABMXA836-06|ROM FN30570|Mexico.Campeche
Artibeus intermedius|ABMXC505-06|ROM 97421|Mexico.Quintana Roo
Artibeus lituratus|ABGYD290-06|ROM 108629|Guyana.Potaro-Siparuni
Artibeus lituratus|ABECA328-06|ROM F37530|Ecuador.Napo
Artibeus lituratus|ABGYB172-06|ROM 101163|Guyana.Barima-Waini
Artibeus lituratus|ABSMS371-06|ROM 117533|Suriname.Sipaliwini
Artibeus lituratus|ABECA134-06|ROM 104432|Ecuador.Napo
Artibeus intermedius|ABMXB672-06|ROM FN29169|Mexico.Campeche
Artibeus lituratus|BCBNT398-06|ROM 109288|Guyana.Potaro-Siparuni
Artibeus intermedius|ABMXB674-06|ROM 95380|Mexico.Campeche
Artibeus lituratus|BCBNT361-06|ROM 108988|Guyana.Potaro-Siparuni
Artibeus lituratus|ABSRA510-06|ROM 117100|Suriname.Nickerie
Artibeus lituratus|ABMXC576-06|ROM 97524|Mexico.Quintana Roo
Artibeus lituratus|ABSRA364-06|ROM 116953|Suriname.Nickerie
Artibeus lituratus|ABGYC489-06|ROM 107322|Guyana.Potaro-Siparuni
Artibeus lituratus|BCBN705-05|ROM 104354|Panama.Darien
Artibeus lituratus|ABGYA803-06|ROM 100861|Guyana.Barima-Waini
Artibeus lituratus|ABGYC427-06|ROM 107249|Guyana.Potaro-Siparuni
Artibeus lituratus|ABGYB463-06|ROM 103197|Guyana.Upper Takutu-Upper Essequibo
Artibeus lituratus|ABGYE431-06|ROM 111890|Guyana.Potaro-Siparuni
Artibeus lituratus|BCBNT397-06|ROM 109287|Guyana.Potaro-Siparuni
Artibeus lituratus|BCBN737-05|ROM 104430|Ecuador.Napo
Artibeus lituratus|ABSRA365-06|ROM 116954|Suriname.Nickerie
Artibeus lituratus|ABSRA310-06|ROM 116899|Suriname.Nickerie
Artibeus lituratus|ABSMS391-06|ROM 117553|Suriname.Sipaliwini
Artibeus lituratus|ABSMS230-06|ROM 117392|Suriname.Sipaliwini
Artibeus lituratus|ABSA112-06|ROM 111158|Brazil.Sao Paulo
Artibeus lituratus|ABSA087-06|ROM 111119|Brazil.Sao Paulo
Artibeus intermedius|ABMXC475-06|ROM 97038|Mexico.Campeche
Artibeus lituratus|ABMXA866-06|ROM FN30613|Mexico.Campeche
Artibeus lituratus|ABMXA835-06|ROM FN30569|Mexico.Campeche
Artibeus lituratus|ABGYC820-06|ROM F43266|Guyana.Cuyuni-Mazaruni
Artibeus lituratus|ABGYD793-06|ROM 109215|Guyana.Potaro-Siparuni
Artibeus lituratus|ABGYD445-06|ROM 108796|Guyana.Potaro-Siparuni
Artibeus lituratus|ABGYD142-06|ROM 108445|Guyana.Potaro-Siparuni
Artibeus lituratus|ABGYB909-06|ROM 104754|Guyana.Potaro-Siparuni
Artibeus lituratus|ABGYA856-06|ROM 100927|Guyana.Barima-Waini
Artibeus lituratus|ABSMS302-06|ROM 117464|Suriname.Sipaliwini
Artibeus lituratus|ABGYB495-06|ROM 103230|Guyana.Upper Takutu-Upper Essequibo
Artibeus lituratus|ABGYE430-06|ROM 111889|Guyana.Potaro-Siparuni
Artibeus lituratus|ABSMS344-06|ROM 117506|Suriname.Sipaliwini
Artibeus lituratus|ABSMS224-06|ROM 117386|Suriname.Sipaliwini
Artibeus lituratus|ABSMS062-06|ROM 117224|Suriname.Sipaliwini
Artibeus lituratus|ABMXB730-06|ROM 95570|Mexico.Campeche
Artibeus lituratus|ABGYG066-06|ROM 115588|Guyana.Essequibo Islands-West Demerara
Artibeus lituratus|ABGYF077-06|ROM 113605|Guyana.Upper Takutu-Upper Essequibo
Artibeus lituratus|ABGYD288-06|ROM 108627|Guyana.Potaro-Siparuni
Artibeus lituratus|ABGYC369-06|ROM 107185|Guyana.Potaro-Siparuni
Artibeus lituratus|ABGYB623-06|ROM 103378|Guyana.Upper Demerara-Berbice
Artibeus lituratus|ABGYA858-06|ROM 100929|Guyana.Barima-Waini
Artibeus lituratus|ABGYA263-06|ROM 98163|Guyana.Potaro-Siparuni
Artibeus lituratus|ABECA131-06|ROM F37265|Ecuador.Napo
Artibeus lituratus|ABGYB134-06|ROM 101116|Guyana.Barima-Waini
Artibeus lituratus|ABGYB183-06|ROM 101176|Guyana.Barima-Waini
Artibeus lituratus|ABGYF238-06|ROM 113817|Guyana.Demerara-Mahaica
Artibeus lituratus|ABGYC802-06|ROM 108201|Guyana.Cuyuni-Mazaruni
Artibeus lituratus|ABGYF184-06|ROM 113745|Guyana.Demerara-Mahaica
Artibeus lituratus|ABGYB105-06|ROM 101083|Guyana.Barima-Waini
Artibeus lituratus|ABSRA379-06|ROM 116968|Suriname.Nickerie
Artibeus lituratus|ABSRA492-06|ROM 117082|Suriname.Nickerie
Artibeus lituratus|BCBNT362-06|ROM 108989|Guyana.Potaro-Siparuni
Artibeus lituratus|ABGYB627-06|ROM 103382|Guyana.Upper Demerara-Berbice
Artibeus lituratus|ABGYA838-06|ROM 100901|Guyana.Barima-Waini
Artibeus intermedius|BCBN470-05|ROM 96987|Mexico.Campeche
Artibeus lituratus|ABGYA843-06|ROM 100906|Guyana.Barima-Waini
Artibeus lituratus|ABGYE738-06|ROM 112681|Guyana.Demerara-Mahaica
Artibeus lituratus|ABGYB152-06|ROM 101140|Guyana.Barima-Waini
Artibeus lituratus|ABGYA661-06|ROM 100360|Guyana.East Berbice-Corentyne
Artibeus lituratus|ABMXB708-06|ROM 95482|Mexico.Campeche
Artibeus lituratus|ABMXA887-06|ROM FN30650|Mexico.Campeche
Artibeus lituratus|ABMXA787-06|ROM FN30476|Mexico.Campeche
Artibeus intermedius|ABMXB772-06|ROM 95734|Mexico.Campeche
Artibeus lituratus|BCBN473-05|ROM 97007|Mexico.Campeche
Artibeus lituratus|ABSA113-06|ROM 111159|Brazil.Sao Paulo
Artibeus lituratus|ABSA084-06|ROM 111116|Brazil.Sao Paulo
Artibeus lituratus|BCBNT483-06|ROM 111100|Brazil.Sao Paulo
Artibeus lituratus|ABGYB417-06|ROM 103124|Guyana.Upper Takutu-Upper Essequibo
Artibeus lituratus|ABGYB929-06|ROM 104774|Guyana.Potaro-Siparuni
Artibeus lituratus|ABGYA752-06|ROM 100458|Guyana.East Berbice-Corentyne
Artibeus lituratus|ABSA063-06|ROM 111077|Brazil.Sao Paulo
Artibeus lituratus|ABGYB683-06|ROM 103447|Guyana.Upper Demerara-Berbice

Artibeus lituratus|ABGYA752-06|ROM 100458|Guyana.East Berbice-Corentyne
Artibeus lituratus|ABSA063-06|ROM 111077|Brazil.Sao Paulo
Artibeus lituratus|ABGYB683-06|ROM 103447|Guyana.Upper Demerara-Berbice
Artibeus lituratus|ABGYD303-06|ROM 108642|Guyana.Potaro-Siparuni
Artibeus lituratus|ABSCA143-06|ROM F44067|Costa Rica.Limon
Artibeus lituratus|ABGYD286-06|ROM 108625|Guyana.Potaro-Siparuni
Artibeus lituratus|ABSA081-06|ROM 111109|Brazil.Sao Paulo
Artibeus lituratus|ABSA086-06|ROM 111118|Brazil.Sao Paulo
Artibeus lituratus|ABGYE791-06|ROM 113392|Guyana.Upper Demerara-Berbice
Artibeus lituratus|ABGYD518-06|ROM 108876|Guyana.Potaro-Siparuni
Artibeus lituratus|ABGYD141-06|ROM 108444|Guyana.Potaro-Siparuni
Artibeus lituratus|ABGYA842-06|ROM 100905|Guyana.Barima-Waini
Artibeus lituratus|ABGYA881-06|ROM 100959|Guyana.Barima-Waini
Artibeus lituratus|ABGYA844-06|ROM 100907|Guyana.Barima-Waini
Artibeus lituratus|ABGYA745-06|ROM 100451|Guyana.East Berbice-Corentyne
Artibeus lituratus|ABGYD741-06|ROM 109159|Guyana.Potaro-Siparuni
Artibeus lituratus|ABSA089-06|ROM 111121|Brazil.Sao Paulo
Artibeus lituratus|ABSA073-06|ROM 111091|Brazil.Sao Paulo
Artibeus lituratus|ABSRA338-06|ROM 116927|Suriname.Nickerie
Artibeus lituratus|ABSRA456-06|ROM 117046|Suriname.Nickerie
Artibeus lituratus|ABSA096-06|ROM 111128|Brazil.Sao Paulo
Artibeus lituratus|ABSA079-06|ROM 111107|Brazil.Sao Paulo
Artibeus lituratus|ABGYB104-06|ROM 101082|Guyana.Barima-Waini
Artibeus lituratus|ABSRA384-06|ROM 116973|Suriname.Nickerie
Artibeus lituratus|ABSRA489-06|ROM 117079|Suriname.Nickerie
Artibeus lituratus|ABSRA521-06|ROM 117111|Suriname.Nickerie
Artibeus lituratus|BCBNT842-06|ROM 114198|Suriname.Brokopondo
Artibeus lituratus|ABSA072-06|ROM 111090|Brazil.Sao Paulo
Artibeus lituratus|ABSA104-06|ROM 111144|Brazil.Sao Paulo
Artibeus lituratus|ABSA098-06|ROM 111135|Brazil.Sao Paulo
Artibeus lituratus|ABSA092-06|ROM 111124|Brazil.Sao Paulo
Artibeus intermedius|ABMXB754-06|ROM 95692|Mexico.Campeche
Artibeus lituratus|ABMXA816-06|ROM FN30526|Mexico.Campeche
Artibeus lituratus|ABGYD830-06|ROM 109254|Guyana.Potaro-Siparuni
Artibeus lituratus|ABGYC135-06|ROM 106647|Guyana.Upper Takutu-Upper Essequibo
Artibeus lituratus|ABGYC117-06|ROM 106629|Guyana.Upper Takutu-Upper Essequibo
Artibeus intermedius|ABMXB766-06|ROM 95728|Mexico.Campeche
Artibeus intermedius|ABMXC477-06|ROM 97040|Mexico.Campeche
Artibeus intermedius|ABMXB670-06|ROM 95376|Mexico.Campeche
Artibeus lituratus|BCBN261-05|ROM 99563|Guatemala.Peten
Artibeus lituratus|ABSA105-06|ROM 111145|Brazil.Sao Paulo
Artibeus lituratus|ABMXA812-06|ROM FN30522|Mexico.Campeche
Artibeus lituratus|ABMXA884-06|ROM FN30647|Mexico.Campeche
Artibeus lituratus|ABMXB753-06|ROM 95691|Mexico.Campeche
Artibeus intermedius|ABMXC453-06|ROM 96963|Mexico.Campeche
Artibeus lituratus|ABMXA865-06|ROM FN30612|Mexico.Campeche
Artibeus lituratus|BCBN262-05|ROM 99566|Guatemala.Peten
Artibeus lituratus|ABCSA512-06|ROM 99524|Guatemala.Peten
Artibeus lituratus|ABCSA301-06|ROM 99277|Guatemala.Peten
Artibeus lituratus|ABGYB141-06|ROM 101127|Guyana.Barima-Waini
Artibeus lituratus|ABCSA509-06|ROM 99520|Guatemala.Peten
Artibeus lituratus|ABSA080-06|ROM 111108|Brazil.Sao Paulo
Artibeus lituratus|BCBNT395-06|ROM 109262|Guyana.Potaro-Siparuni
Artibeus lituratus|BCBNT099-06|ROM 107109|Guyana.Potaro-Siparuni
Artibeus lituratus|ABSA110-06|ROM 111155|Brazil.Sao Paulo
Artibeus lituratus|ABGYB171-06|ROM 101162|Guyana.Barima-Waini
Artibeus lituratus|ABGYE811-06|ROM 113414|Guyana.Upper Demerara-Berbice
Artibeus lituratus|ABGYC463-06|ROM 107293|Guyana.Potaro-Siparuni
Artibeus lituratus|ABGYB151-06|ROM 101139|Guyana.Barima-Waini
Artibeus lituratus|ABGYB076-06|ROM 101052|Guyana.Barima-Waini
Artibeus lituratus|ABSA088-06|ROM 111120|Brazil.Sao Paulo
Artibeus lituratus|BCBNT481-06|ROM 111098|Brazil.Sao Paulo
Artibeus lituratus|BCBNT487-06|ROM 111104|Brazil.Sao Paulo
Artibeus lituratus|ABECA485-06|ROM F37740|Ecuador.Napo
Artibeus lituratus|ABGYD792-06|ROM 109214|Guyana.Potaro-Siparuni
Artibeus lituratus|ABSMS339-06|ROM 117501|Suriname.Sipaliwini
Artibeus lituratus|ABSRA356-06|ROM 116945|Suriname.Nickerie
Artibeus intermedius|ABMXB752-06|ROM 95690|Mexico.Campeche
Artibeus lituratus|ABSMS324-06|ROM 117486|Suriname.Sipaliwini
Artibeus intermedius|ABMXB652-06|ROM 95341|Mexico.Campeche
Artibeus lituratus|ABGYE591-06|ROM 112070|Guyana.Potaro-Siparuni
Artibeus lituratus|ABGYD542-06|ROM 108902|Guyana.Potaro-Siparuni
Artibeus lituratus|ABGYC737-06|ROM 108138|Guyana.Cuyuni-Mazaruni
Artibeus lituratus|ABGYC433-06|ROM 107257|Guyana.Potaro-Siparuni
Artibeus lituratus|ABGYB710-06|ROM 103475|Guyana.Upper Demerara-Berbice
Artibeus lituratus|ABGYC464-06|ROM 107294|Guyana.Potaro-Siparuni
Artibeus lituratus|ABGYB721-06|ROM 103487|Guyana.Upper Demerara-Berbice
Artibeus lituratus|ABGYA875-06|ROM 100951|Guyana.Barima-Waini
Artibeus lituratus|ABGYB647-06|ROM 103404|Guyana.Upper Demerara-Berbice
Artibeus lituratus|ABGYB462-06|ROM 103196|Guyana.Upper Takutu-Upper Essequibo
Artibeus lituratus|ABGYE078-06|ROM 111521|Guyana.Potaro-Siparuni
Artibeus lituratus|ABGYD794-06|ROM 109216|Guyana.Potaro-Siparuni
Artibeus lituratus|ABGYG786-08|ROM 119214|Guyana
Artibeus lituratus|ABCSA347-06|ROM 99329|Guatemala.Peten
Artibeus lituratus|ABGYB397-06|ROM 103104|Guyana.Upper Takutu-Upper Essequibo
Artibeus lituratus|ABGYB358-06|ROM 103065|Guyana.Upper Takutu-Upper Essequibo
Artibeus lituratus|ABGYD483-06|ROM 108836|Guyana.Potaro-Siparuni
Artibeus lituratus|ABSRA361-06|ROM 116950|Suriname.Nickerie
Artibeus lituratus|ABGYF012-06|ROM 113502|Guyana.Upper Takutu-Upper Essequibo
Artibeus lituratus|BCBNT827-06|ROM 114168|Suriname.Brokopondo
Artibeus lituratus|ABSA117-06|ROM 111165|Brazil.Sao Paulo
Artibeus lituratus|ABGYE712-06|ROM 112647|Guyana.Demerara-Mahaica
Artibeus lituratus|ABGYD494-06|ROM 108852|Guyana.Potaro-Siparuni
Artibeus lituratus|ABGYE058-06|ROM 109344|Guyana.Potaro-Siparuni
Artibeus lituratus|ABGYG837-08|ROM 119265|Guyana
Artibeus lituratus|BCBNT484-06|ROM 111101|Brazil.Sao Paulo
Artibeus lituratus|ABECA330-06|ROM 105213|Ecuador.Napo
Artibeus lituratus|ABSA091-06|ROM 111123|Brazil.Sao Paulo
Artibeus lituratus|ABSA109-06|ROM 111153|Brazil.Sao Paulo
Artibeus lituratus|ABSMS100-06|ROM 117262|Suriname.Sipaliwini
Artibeus lituratus|ABGYD530-06|ROM 108888|Guyana.Potaro-Siparuni
Artibeus lituratus|ABGYA371-06|ROM 98772|Guyana.Barima-Waini
Artibeus lituratus|ABGYB178-06|ROM 101171|Guyana.Barima-Waini

Artibeus lituratus|ABGYD530-06|ROM 108888|Guyana.Potaro-Siparuni
Artibeus lituratus|ABGYA371-06|ROM 98772|Guyana.Barima-Waini
Artibeus lituratus|ABGYB178-06|ROM 101171|Guyana.Barima-Waini
Artibeus lituratus|ABGYA857-06|ROM 100928|Guyana.Barima-Waini
Artibeus lituratus|ABGYD244-06|ROM 108576|Guyana.Potaro-Siparuni
Artibeus lituratus|ABGYG036-06|ROM 115543|Guyana.Essequibo Islands-West Demerara
Artibeus lituratus|ABGYB597-06|ROM 103338|Guyana.Upper Takutu-Upper Essequibo
Artibeus lituratus|ABSMS041-06|ROM 117203|Suriname.Sipaliwini
Artibeus lituratus|ABECA132-06|ROM 104429|Ecuador.Napo
Artibeus lituratus|ABGYC773-06|ROM 108178|Guyana.Cuyuni-Mazaruni
Artibeus lituratus|ABGYD701-06|ROM 109114|Guyana.Potaro-Siparuni
Artibeus lituratus|ABGYD408-06|ROM 108754|Guyana.Potaro-Siparuni
Artibeus lituratus|ABGYG1030-08|ROM 118954|Guyana
Artibeus intermedius|ABMXC464-06|ROM 96992|Mexico.Campeche
Artibeus amplus|ABGYG533-06|ROM 116677|Guyana.Potaro-Siparuni
Artibeus amplus|ABGYG534-06|ROM 116678|Guyana.Potaro-Siparuni
Artibeus amplus|ABGYG573-06|ROM 116735|Guyana.Potaro-Siparuni
Artibeus amplus|BCBNT058-06|ROM 106722|Guyana.Upper Takutu-Upper Essequibo
Artibeus amplus|ABGYG532-06|ROM 116676|Guyana.Potaro-Siparuni
Artibeus amplus|ABGYG537-06|ROM 116681|Guyana.Potaro-Siparuni
Artibeus amplus|ABGYG540-06|ROM 116684|Guyana.Potaro-Siparuni
Artibeus amplus|ABGYG530-06|ROM 116674|Guyana.Potaro-Siparuni
Artibeus amplus|ABGYG531-06|ROM 116675|Guyana.Potaro-Siparuni
Artibeus amplus|ABGYG548-06|ROM 116694|Guyana.Potaro-Siparuni
Artibeus amplus|BCBNT062-06|ROM 106748|Guyana.Upper Takutu-Upper Essequibo
Artibeus amplus|ABGYG518-06|ROM 116660|Guyana.Potaro-Siparuni
Artibeus amplus|BCBNT045-06|ROM 106679|Guyana.Upper Takutu-Upper Essequibo
Artibeus amplus|ABGYG568-06|ROM 116722|Guyana.Potaro-Siparuni
Artibeus amplus|BCBNT065-06|ROM 106761|Guyana.Upper Takutu-Upper Essequibo
Artibeus amplus|ABGYG453-06|ROM 116590|Guyana.Potaro-Siparuni
Artibeus amplus|ABGYG539-06|ROM 116683|Guyana.Potaro-Siparuni
Artibeus amplus|ABGYG542-06|ROM 116686|Guyana.Potaro-Siparuni
Artibeus amplus|ABGYG567-06|ROM 116721|Guyana.Potaro-Siparuni
Artibeus amplus|ABGYG543-06|ROM 116687|Guyana.Potaro-Siparuni
Artibeus amplus|ABGYG517-06|ROM 116659|Guyana.Potaro-Siparuni
Artibeus amplus|ABGYG536-06|ROM 116680|Guyana.Potaro-Siparuni
Artibeus amplus|ABGYG581-06|ROM 116744|Guyana.Potaro-Siparuni
Artibeus amplus|BCBNT051-06|ROM 106697|Guyana.Upper Takutu-Upper Essequibo
Artibeus amplus|BCBNC063-06|ROM 115137|Guyana.Cuyuni-Mazaruni
Artibeus amplus|ABGYG538-06|ROM 116682|Guyana.Potaro-Siparuni
Artibeus amplus|ABGYG541-06|ROM 116685|Guyana.Potaro-Siparuni
Artibeus amplus|ABGYG574-06|ROM 116736|Guyana.Potaro-Siparuni
Artibeus amplus|BCBNT173-06|ROM 107847|Venezuela.Amazonas
Artibeus amplus|ABGYG519-06|ROM 116661|Guyana.Potaro-Siparuni
Artibeus amplus|ABGYG535-06|ROM 116679|Guyana.Potaro-Siparuni
Artibeus amplus|BCBNT198-06|ROM 107904|Venezuela.Amazonas
Artibeus planirostris|ABGYA207-06|ROM 98075|Guyana.Potaro-Siparuni
Artibeus planirostris|ABSRA373-06|ROM 116962|Suriname.Nickerie
Artibeus planirostris|ABSMS226-06|ROM 117388|Suriname.Sipaliwini
Artibeus planirostris|ABSMS190-06|ROM 117352|Suriname.Sipaliwini
Artibeus planirostris|ABGYG769-08|ROM 119197|Guyana
Artibeus planirostris|ABGYC823-06|ROM 108227|Guyana.Cuyuni-Mazaruni
Artibeus planirostris|ABGYA751-06|ROM 100457|Guyana.East Berbice-Corentyne
Artibeus planirostris|ABGYE288-06|ROM 111737|Guyana.Potaro-Siparuni
Artibeus planirostris|ABGYB320-06|ROM 103025|Guyana.Upper Takutu-Upper Essequibo
Artibeus planirostris|ABGYB319-06|ROM 103024|Guyana.Upper Takutu-Upper Essequibo
Artibeus planirostris|ABGYA840-06|ROM 100903|Guyana.Barima-Waini
Artibeus planirostris|ABGYA709-06|ROM 100414|Guyana.East Berbice-Corentyne
Artibeus planirostris|ABGYF014-06|ROM 113504|Guyana.Upper Takutu-Upper Essequibo
Artibeus planirostris|ABGYF263-06|ROM 113851|Guyana.Demerara-Mahaica
Artibeus planirostris|BCBNT095-06|ROM 107095|Guyana.Potaro-Siparuni
Artibeus planirostris|ABGYD161-06|ROM 108473|Guyana.Potaro-Siparuni
Artibeus planirostris|ABGYD007-06|ROM 108229|Guyana.Cuyuni-Mazaruni
Artibeus planirostris|ABGYE682-06|ROM 112610|Guyana.Demerara-Mahaica
Artibeus planirostris|ABGYF137-06|ROM 113688|Guyana.Demerara-Mahaica
Artibeus planirostris|ABGYF180-06|ROM 113737|Guyana.Demerara-Mahaica
Artibeus planirostris|ABGYA588-06|ROM 100273|Guyana.East Berbice-Corentyne
Artibeus planirostris|ABGYA674-06|ROM 100379|Guyana.East Berbice-Corentyne
Artibeus planirostris|ABGYA677-06|ROM 100382|Guyana.East Berbice-Corentyne
Artibeus planirostris|ABGYA705-06|ROM 100410|Guyana.East Berbice-Corentyne
Artibeus planirostris|ABGYA727-06|ROM 100432|Guyana.East Berbice-Corentyne
Artibeus planirostris|ABSRA491-06|ROM 117081|Suriname.Nickerie
Artibeus planirostris|ABGYD254-06|ROM 108590|Guyana.Potaro-Siparuni
Artibeus planirostris|ABSMS287-06|ROM 117449|Suriname.Sipaliwini
Artibeus planirostris|ABGYB340-06|ROM 103047|Guyana.Upper Takutu-Upper Essequibo
Artibeus planirostris|ABGYA575-06|ROM 100255|Guyana.East Berbice-Corentyne
Artibeus planirostris|ABGYE237-06|ROM 111685|Guyana.Potaro-Siparuni
Artibeus planirostris|ABGYF259-06|ROM 113847|Guyana.Demerara-Mahaica
Artibeus planirostris|ABGYF170-06|ROM 113727|Guyana.Demerara-Mahaica
Artibeus planirostris|ABGYF035-06|ROM 113542|Guyana.Upper Takutu-Upper Essequibo
Artibeus planirostris|ABGYE789-06|ROM 113390|Guyana.Upper Demerara-Berbice
Artibeus planirostris|ABGYE579-06|ROM 112056|Guyana.Potaro-Siparuni
Artibeus planirostris|ABGYE236-06|ROM 111684|Guyana.Potaro-Siparuni
Artibeus planirostris|ABGYE217-06|ROM 111665|Guyana.Potaro-Siparuni
Artibeus planirostris|ABGYD783-06|ROM 109204|Guyana.Potaro-Siparuni
Artibeus planirostris|ABGYD270-06|ROM 108606|Guyana.Potaro-Siparuni
Artibeus planirostris|ABGYD246-06|ROM 108578|Guyana.Potaro-Siparuni
Artibeus planirostris|ABGYD148-06|ROM 108454|Guyana.Potaro-Siparuni
Artibeus planirostris|ABGYD048-06|ROM 108271|Guyana.Cuyuni-Mazaruni
Artibeus planirostris|ABGYD043-06|ROM 108266|Guyana.Cuyuni-Mazaruni
Artibeus planirostris|ABGYB595-06|ROM 103336|Guyana.Upper Takutu-Upper Essequibo
Artibeus planirostris|ABGYA855-06|ROM 100925|Guyana.Barima-Waini
Artibeus planirostris|ABGYA671-06|ROM 100375|Guyana.East Berbice-Corentyne
Artibeus planirostris|ABGYA650-06|ROM 100346|Guyana.East Berbice-Corentyne
Artibeus planirostris|ABGYA633-06|ROM 100327|Guyana.East Berbice-Corentyne
Artibeus planirostris|ABGYA630-06|ROM 100324|Guyana.East Berbice-Corentyne
Artibeus planirostris|ABGYA574-06|ROM 100254|Guyana.East Berbice-Corentyne
Artibeus planirostris|ABGYA560-06|ROM 100235|Guyana.East Berbice-Corentyne
Artibeus planirostris|ABGYA121-06|ROM 97961|Guyana.Upper Takutu-Upper Essequibo
Artibeus planirostris|ABGYA002-06|ROM 97770|Guyana.Upper Takutu-Upper Essequibo
Artibeus planirostris|ABGYG896-08|ROM 119324|Guyana
Artibeus planirostris|ABECB067-06|ROM 97768|Guyana.Upper Takutu-Upper Essequibo
Artibeus planirostris|ABGYA201-06|ROM 98069|Guyana.Potaro-Siparuni

[illegible]
Artibeus planirostris|ABGYG896-08|ROM 119324|Guyana
Artibeus planirostris|ABECB067-06|ROM 97768|Guyana.Upper Takutu-Upper Essequibo
Artibeus planirostris|ABGYA201-06|ROM 98069|Guyana.Potaro-Siparuni
Artibeus planirostris|ABGYG1003-08|ROM 118927|Guyana
Artibeus planirostris|ABGYG923-08|ROM 119351|Guyana
Artibeus planirostris|ABSMS228-06|ROM 117390|Suriname.Sipaliwini
Artibeus planirostris|ABSMS354-06|ROM 117516|Suriname.Sipaliwini
Artibeus planirostris|ABSRA321-06|ROM 116910|Suriname.Nickerie
Artibeus planirostris|ABGYD188-06|ROM 108515|Guyana.Potaro-Siparuni
Artibeus planirostris|ABGYF031-06|ROM 113529|Guyana.Upper Takutu-Upper Essequibo
Artibeus planirostris|ABGYG900-08|ROM 119328|Guyana
Artibeus planirostris|ABGYG714-08|ROM 119142|Guyana
Artibeus planirostris|ABGYD185-06|ROM 108512|Guyana.Potaro-Siparuni
Artibeus planirostris|ABGYD140-06|ROM 108443|Guyana.Potaro-Siparuni
Artibeus planirostris|ABGYA165-06|ROM 98024|Guyana.Potaro-Siparuni
Artibeus planirostris|ABSMS309-06|ROM 117471|Suriname.Sipaliwini
Artibeus planirostris|ABSRA320-06|ROM 116909|Suriname.Nickerie
Artibeus planirostris|BCBNT623-06|ROM 113540|Guyana.Upper Takutu-Upper Essequibo
Artibeus planirostris|ABGYD159-06|ROM 108471|Guyana.Potaro-Siparuni
Artibeus planirostris|BCBNT738-06|ROM 113915|Suriname.Brokopondo
Artibeus planirostris|ABSRA319-06|ROM 116908|Suriname.Nickerie
Artibeus planirostris|ABGYB222-06|ROM 102924|Guyana.Upper Takutu-Upper Essequibo
Artibeus planirostris|ABGYD118-06|ROM 108416|Guyana.Potaro-Siparuni
Artibeus planirostris|ABGYG920-08|ROM 119348|Guyana
Artibeus planirostris|ABGYF134-06|ROM 113685|Guyana.Demerara-Mahaica
Artibeus planirostris|ABGYG713-08|ROM 119141|Guyana
Artibeus planirostris|ABGYB364-06|ROM 103071|Guyana.Upper Takutu-Upper Essequibo
Artibeus planirostris|ABGYD208-06|ROM 108539|Guyana.Potaro-Siparuni
Artibeus planirostris|ABGYF001-06|ROM 113485|Guyana.Upper Takutu-Upper Essequibo
Artibeus planirostris|ABGYG1058-08|ROM 118982|Guyana
Artibeus planirostris|ABGYG922-08|ROM 119350|Guyana
Artibeus planirostris|ABSRA357-06|ROM 116946|Suriname.Nickerie
Artibeus planirostris|ABGYE752-06|ROM 112698|Guyana.Demerara-Mahaica
Artibeus planirostris|ABGYG897-08|ROM 119325|Guyana
Artibeus planirostris|ABGYG919-08|ROM 119347|Guyana
Artibeus planirostris|ABGYC138-06|ROM 106650|Guyana.Upper Takutu-Upper Essequibo
Artibeus planirostris|ABGYA675-06|ROM 100380|Guyana.East Berbice-Corentyne
Artibeus planirostris|ABGYB204-06|ROM 102906|Guyana.Upper Takutu-Upper Essequibo
Artibeus planirostris|ABGYA737-06|ROM 100442|Guyana.East Berbice-Corentyne
Artibeus planirostris|ABGYB363-06|ROM 103070|Guyana.Upper Takutu-Upper Essequibo
Artibeus planirostris|ABSRA395-06|ROM 116984|Suriname.Nickerie
Artibeus planirostris|BCBN345-05|ROM 100956|Guyana.Barima-Waini
Artibeus planirostris|ABSRA309-06|ROM 116898|Suriname.Nickerie
Artibeus planirostris|ABSRA322-06|ROM 116911|Suriname.Nickerie
Artibeus planirostris|ABGYG927-08|ROM 119355|Guyana
Artibeus planirostris|ABSMS227-06|ROM 117389|Suriname.Sipaliwini
Artibeus planirostris|ABGYG799-08|ROM 119227|Guyana
Artibeus planirostris|ABGYG894-08|ROM 119322|Guyana
Artibeus planirostris|ABGYG1057-08|ROM 118981|Guyana
Artibeus planirostris|ABGYG788-08|ROM 119216|Guyana
Artibeus planirostris|ABGYE714-06|ROM 112649|Guyana.Demerara-Mahaica
Artibeus planirostris|ABGYF055-06|ROM 113568|Guyana.Upper Takutu-Upper Essequibo
Artibeus planirostris|ABGYD769-06|ROM 109189|Guyana.Potaro-Siparuni
Artibeus planirostris|ABGYE382-06|ROM 111834|Guyana.Potaro-Siparuni
Artibeus planirostris|ABGYD298-06|ROM 108637|Guyana.Potaro-Siparuni
Artibeus planirostris|ABGYD479-06|ROM 108832|Guyana.Potaro-Siparuni
Artibeus planirostris|ABGYD152-06|ROM 108458|Guyana.Potaro-Siparuni
Artibeus planirostris|ABGYD265-06|ROM 108601|Guyana.Potaro-Siparuni
Artibeus planirostris|ABGYC666-06|ROM 107486|Guyana.Potaro-Siparuni
Artibeus planirostris|ABGYD085-06|ROM 108382|Guyana.Potaro-Siparuni
Artibeus planirostris|ABGYC186-06|ROM 106721|Guyana.Upper Takutu-Upper Essequibo
Artibeus planirostris|ABGYC281-06|ROM 107074|Guyana.Potaro-Siparuni
Artibeus planirostris|ABGYB338-06|ROM 103045|Guyana.Upper Takutu-Upper Essequibo
Artibeus planirostris|ABGYB203-06|ROM 102905|Guyana.Upper Takutu-Upper Essequibo
Artibeus planirostris|ABGYB186-06|ROM 102888|Guyana.Upper Takutu-Upper Essequibo
Artibeus planirostris|ABGYA839-06|ROM 100902|Guyana.Barima-Waini
Artibeus planirostris|ABGYA836-06|ROM 100899|Guyana.Barima-Waini
Artibeus planirostris|ABGYA647-06|ROM 100343|Guyana.East Berbice-Corentyne
Artibeus planirostris|ABGYA635-06|ROM 100329|Guyana.East Berbice-Corentyne
Artibeus planirostris|ABGYC137-06|ROM 106649|Guyana.Upper Takutu-Upper Essequibo
Artibeus planirostris|ABGYA586-06|ROM 100271|Guyana.East Berbice-Corentyne
Artibeus planirostris|ABGYA660-06|ROM 100359|Guyana.East Berbice-Corentyne
Artibeus planirostris|ABGYA841-06|ROM 100904|Guyana.Barima-Waini
Artibeus planirostris|ABGYB251-06|ROM 102955|Guyana.Upper Takutu-Upper Essequibo
Artibeus planirostris|ABSMS019-06|ROM 117181|Suriname.Sipaliwini
Artibeus planirostris|ABGYA567-06|ROM 100243|Guyana.East Berbice-Corentyne
Artibeus planirostris|ABGYC324-06|ROM 107136|Guyana.Potaro-Siparuni
Artibeus planirostris|ABGYD413-06|ROM 108760|Guyana.Potaro-Siparuni
Artibeus planirostris|ABSMS263-06|ROM 117425|Suriname.Sipaliwini
Artibeus planirostris|ABGYA209-06|ROM 98077|Guyana.Potaro-Siparuni
Artibeus planirostris|ABGYD469-06|ROM 108820|Guyana.Potaro-Siparuni
Artibeus planirostris|ABGYE362-06|ROM 111812|Guyana.Potaro-Siparuni
Artibeus planirostris|ABGYE363-06|ROM 111813|Guyana.Potaro-Siparuni
Artibeus planirostris|ABGYD450-06|ROM 108801|Guyana.Potaro-Siparuni
Artibeus planirostris|ABGYB837-06|ROM 104678|Guyana.Potaro-Siparuni
Artibeus planirostris|ABGYA208-06|ROM 98076|Guyana.Potaro-Siparuni
Artibeus planirostris|ABGYE861-06|ROM 113477|Guyana.Upper Takutu-Upper Essequibo
Artibeus planirostris|ABSRA523-06|ROM 117113|Suriname.Nickerie
Artibeus planirostris|BCBNT094-06|ROM 107094|Guyana.Potaro-Siparuni
Artibeus planirostris|ABGYA643-06|ROM 100338|Guyana.East Berbice-Corentyne
Artibeus planirostris|ABGYC780-06|ROM F43212|Guyana.Cuyuni-Mazaruni
Artibeus planirostris|ABGYD184-06|ROM 108511|Guyana.Potaro-Siparuni
Artibeus planirostris|ABGYD294-06|ROM 108633|Guyana.Potaro-Siparuni
Artibeus planirostris|ABGYD759-06|ROM 109179|Guyana.Potaro-Siparuni
Artibeus planirostris|ABGYE467-06|ROM 111934|Guyana.Potaro-Siparuni
Artibeus planirostris|ABGYF125-06|ROM 113671|Guyana.Demerara-Mahaica
Artibeus planirostris|ABGYF138-06|ROM 113689|Guyana.Demerara-Mahaica
Artibeus planirostris|ABSMS189-06|ROM 117351|Suriname.Sipaliwini
Artibeus planirostris|ABSMS098-06|ROM 117260|Suriname.Sipaliwini
Artibeus planirostris|ABSMS068-06|ROM 117230|Suriname.Sipaliwini
Artibeus planirostris|ABGYG926-08|ROM 119354|Guyana
Artibeus planirostris|ABGYG1084-08|ROM 119008|Guyana
[illegible]

Artibeus planirostris|ABSMS068-06|ROM 117230|Suriname.Sipaliwini
Artibeus planirostris|ABGYG926-08|ROM 119354|Guyana
Artibeus planirostris|ABGYG1084-08|ROM 119008|Guyana
Artibeus planirostris|ABGYG1004-08|ROM 118928|Guyana
Artibeus planirostris|ABGYF262-06|ROM 113850|Guyana.Demerara-Mahaica
Artibeus planirostris|ABGYF037-06|ROM 113544|Guyana.Upper Takutu-Upper Essequibo
Artibeus planirostris|ABGYE456-06|ROM 111920|Guyana.Potaro-Siparuni
Artibeus planirostris|ABGYE322-06|ROM 111772|Guyana.Potaro-Siparuni
Artibeus planirostris|ABGYD779-06|ROM 109200|Guyana.Potaro-Siparuni
Artibeus planirostris|ABGYD269-06|ROM 108605|Guyana.Potaro-Siparuni
Artibeus planirostris|ABGYC527-06|ROM 107365|Guyana.Potaro-Siparuni
Artibeus planirostris|ABGYC495-06|ROM 107328|Guyana.Potaro-Siparuni
Artibeus planirostris|ABGYB323-06|ROM 103028|Guyana.Upper Takutu-Upper Essequibo
Artibeus planirostris|ABGYA822-06|ROM 100881|Guyana.Barima-Waini
Artibeus planirostris|ABGYA710-06|ROM 100415|Guyana.East Berbice-Corentyne
Artibeus planirostris|ABGYA634-06|ROM 100328|Guyana.East Berbice-Corentyne
Artibeus planirostris|ABGYA628-06|ROM 100322|Guyana.East Berbice-Corentyne
Artibeus planirostris|ABGYA587-06|ROM 100272|Guyana.East Berbice-Corentyne
Artibeus planirostris|ABGYA203-06|ROM 98071|Guyana.Potaro-Siparuni
Artibeus planirostris|ABGYA199-06|ROM 98067|Guyana.Potaro-Siparuni
Artibeus planirostris|ABGYF057-06|ROM 113570|Guyana.Upper Takutu-Upper Essequibo
Artibeus planirostris|ABGYE750-06|ROM 112696|Guyana.Demerara-Mahaica
Artibeus planirostris|ABGYA729-06|ROM 100434|Guyana.East Berbice-Corentyne
Artibeus planirostris|ABGYA636-06|ROM 100330|Guyana.East Berbice-Corentyne
Artibeus planirostris|ABGYE705-06|ROM 112639|Guyana.Demerara-Mahaica
Artibeus planirostris|ABSMS373-06|ROM 117535|Suriname.Sipaliwini
Artibeus planirostris|ABSRA481-06|ROM 117071|Suriname.Nickerie
Artibeus planirostris|BCBNC131-06|ROM 115799|Guyana.Potaro-Siparuni
Artibeus planirostris|BCBNT363-06|ROM 108991|Guyana.Potaro-Siparuni
Artibeus planirostris|ABGYA454-06|ROM 98864|Guyana.Barima-Waini
Artibeus planirostris|ABGYA456-06|ROM 98866|Guyana.Barima-Waini
Artibeus planirostris|ABGYC119-06|ROM 106631|Guyana.Upper Takutu-Upper Essequibo
Artibeus planirostris|ABGYA629-06|ROM 100323|Guyana.East Berbice-Corentyne
Artibeus planirostris|ABGYA860-06|ROM 100931|Guyana.Barima-Waini
Artibeus planirostris|BCBNT819-06|ROM 114134|Suriname.Brokopondo
Artibeus planirostris|ABGYD147-06|ROM 108453|Guyana.Potaro-Siparuni
Artibeus planirostris|ABGYD236-06|ROM 108568|Guyana.Potaro-Siparuni
Artibeus planirostris|ABGYE663-06|ROM 112584|Guyana.Demerara-Mahaica
Artibeus planirostris|ABGYE715-06|ROM 112650|Guyana.Demerara-Mahaica
Artibeus planirostris|ABGYE748-06|ROM 112694|Guyana.Demerara-Mahaica
Artibeus planirostris|ABGYE753-06|ROM 112699|Guyana.Demerara-Mahaica
Artibeus planirostris|ABGYE790-06|ROM 113391|Guyana.Upper Demerara-Berbice
Artibeus planirostris|ABGYF036-06|ROM 113543|Guyana.Upper Takutu-Upper Essequibo
Artibeus planirostris|ABGYF056-06|ROM 113569|Guyana.Upper Takutu-Upper Essequibo
Artibeus planirostris|ABGYF126-06|ROM 113672|Guyana.Demerara-Mahaica
Artibeus planirostris|ABGYF136-06|ROM 113687|Guyana.Demerara-Mahaica
Artibeus planirostris|ABGYF147-06|ROM 113700|Guyana.Demerara-Mahaica
Artibeus planirostris|ABGYF264-06|ROM 113852|Guyana.Demerara-Mahaica
Artibeus planirostris|ABGYF267-06|ROM 113855|Guyana.Demerara-Mahaica
Artibeus planirostris|ABGYG712-08|ROM 119140|Guyana
Artibeus planirostris|ABGYG749-08|ROM 119177|Guyana
Artibeus planirostris|ABGYG797-08|ROM 119225|Guyana
Artibeus planirostris|ABGYG921-08|ROM 119349|Guyana
Artibeus planirostris|ABGYG924-08|ROM 119352|Guyana
Artibeus planirostris|ABGYG945-08|ROM 119373|Guyana
Artibeus planirostris|BCBNT081-06|ROM 107052|Guyana.Potaro-Siparuni
Artibeus planirostris|BCBNT174-06|ROM 107848|Venezuela.Amazonas
Artibeus planirostris|ABGYE381-06|ROM 111833|Guyana.Potaro-Siparuni
Artibeus planirostris|ABGYE466-06|ROM 111933|Guyana.Potaro-Siparuni
Artibeus planirostris|ABGYD296-06|ROM 108635|Guyana.Potaro-Siparuni
Artibeus planirostris|ABGYD528-06|ROM 108886|Guyana.Potaro-Siparuni
Artibeus planirostris|ABGYD268-06|ROM 108604|Guyana.Potaro-Siparuni
Artibeus planirostris|ABGYD291-06|ROM 108630|Guyana.Potaro-Siparuni
Artibeus planirostris|ABGYD040-06|ROM 108262|Guyana.Cuyuni-Mazaruni
Artibeus planirostris|ABGYD132-06|ROM 108435|Guyana.Potaro-Siparuni
Artibeus planirostris|BCBNT619-06|ROM 113534|Guyana.Upper Takutu-Upper Essequibo
Artibeus planirostris|BCBNT624-06|ROM 113541|Guyana.Upper Takutu-Upper Essequibo
Artibeus planirostris|BCBNT331-06|ROM 108905|Guyana.Potaro-Siparuni
Artibeus planirostris|BCBNT529-06|ROM 111915|Guyana.Potaro-Siparuni
Artibeus planirostris|ABGYG1073-08|ROM 118997|Guyana
Artibeus planirostris|ABGYG663-08|ROM 119091|Guyana
Artibeus planirostris|ABGYF171-06|ROM 113728|Guyana.Demerara-Mahaica
Artibeus planirostris|ABGYF260-06|ROM 113848|Guyana.Demerara-Mahaica
Artibeus planirostris|BCBNT630-06|ROM 113567|Guyana.Upper Takutu-Upper Essequibo
Artibeus planirostris|ABGYD008-06|ROM 108230|Guyana.Cuyuni-Mazaruni
Artibeus planirostris|ABGYC763-06|ROM 108165|Guyana.Cuyuni-Mazaruni
Artibeus planirostris|ABGYC089-06|ROM 106593|Guyana.Upper Takutu-Upper Essequibo
Artibeus planirostris|ABGYB669-06|ROM 103432|Guyana.Upper Demerara-Berbice
Artibeus planirostris|ABGYA817-06|ROM 100876|Guyana.Barima-Waini
Artibeus planirostris|ABGYA697-06|ROM 100402|Guyana.East Berbice-Corentyne
Artibeus planirostris|ABGYA589-06|ROM 100274|Guyana.East Berbice-Corentyne
Artibeus planirostris|ABGYA210-06|ROM 98078|Guyana.Potaro-Siparuni
Artibeus planirostris|ABGYD449-06|ROM 108800|Guyana.Potaro-Siparuni
Artibeus planirostris|ABGYG1023-08|ROM 118947|Guyana
Artibeus planirostris|ABGYD293-06|ROM 108632|Guyana.Potaro-Siparuni
Artibeus planirostris|ABGYD295-06|ROM 108634|Guyana.Potaro-Siparuni
Artibeus planirostris|BCBNT535-06|ROM 111942|Guyana.Potaro-Siparuni
Artibeus planirostris|BCBNT770-06|ROM 113988|Suriname.Brokopondo
Artibeus planirostris|ABGYA510-06|ROM 98921|Guyana.Barima-Waini
Artibeus planirostris|ABSRA344-06|ROM 116933|Suriname.Nickerie
Artibeus planirostris|ABGYA206-06|ROM 98074|Guyana.Potaro-Siparuni
Artibeus planirostris|ABGYE172-06|ROM 111615|Guyana.Potaro-Siparuni
Artibeus planirostris|ABGYD370-06|ROM 108713|Guyana.Potaro-Siparuni
Artibeus planirostris|ABGYE339-06|ROM 111789|Guyana.Potaro-Siparuni
Artibeus planirostris|ABGYE818-06|ROM 113424|Guyana.Upper Demerara-Berbice
Artibeus planirostris|ABGYE676-06|ROM 112603|Guyana.Demerara-Mahaica
Artibeus planirostris|ABGYG436-06|ROM 116571|Guyana.Potaro-Siparuni
Artibeus planirostris|ABGYA863-06|ROM 100934|Guyana.Barima-Waini
Artibeus planirostris|ABGYC764-06|ROM 108166|Guyana.Cuyuni-Mazaruni
Artibeus planirostris|ABGYA639-06|ROM 100333|Guyana.East Berbice-Corentyne
Artibeus planirostris|ABGYC762-06|ROM 108164|Guyana.Cuyuni-Mazaruni
Artibeus planirostris|ABGYD001-06|ROM 108228|Guyana.Cuyuni-Mazaruni
Artibeus planirostris|ABGYD004-06|ROM F43273|Guyana.Cuyuni-Mazaruni

Artibeus planirostris|ABGYC762-06|ROM 108164|Guyana.Cuyuni-Mazaruni
Artibeus planirostris|ABGYD001-06|ROM 108228|Guyana.Cuyuni-Mazaruni
Artibeus planirostris|ABGYD004-06|ROM F43273|Guyana.Cuyuni-Mazaruni
Artibeus planirostris|ABGYD047-06|ROM 108270|Guyana.Cuyuni-Mazaruni
Artibeus planirostris|ABGYD470-06|ROM 108821|Guyana.Potaro-Siparuni
Artibeus planirostris|ABGYE749-06|ROM 112695|Guyana.Demerara-Mahaica
Artibeus planirostris|ABGYE173-06|ROM 111616|Guyana.Potaro-Siparuni
Artibeus planirostris|ABGYG1036-08|ROM 118960|Guyana
Artibeus planirostris|ABGYE552-06|ROM 112025|Guyana.Potaro-Siparuni
Artibeus planirostris|ABSMS099-06|ROM 117261|Suriname.Sipaliwini
Artibeus planirostris|ABSMS225-06|ROM 117387|Suriname.Sipaliwini
Artibeus planirostris|ABGYG768-08|ROM 119196|Guyana
Artibeus planirostris|ABSMS088-06|ROM 117250|Suriname.Sipaliwini
Artibeus planirostris|ABSMS338-06|ROM 117500|Suriname.Sipaliwini
Artibeus planirostris|ABSMS355-06|ROM 117517|Suriname.Sipaliwini
Artibeus planirostris|ABGYE801-06|ROM 113403|Guyana.Upper Demerara-Berbice
Artibeus planirostris|ABGYF013-06|ROM 113503|Guyana.Upper Takutu-Upper Essequibo
Artibeus planirostris|ABGYD266-06|ROM 108602|Guyana.Potaro-Siparuni
Artibeus planirostris|ABGYD133-06|ROM 108436|Guyana.Potaro-Siparuni
Artibeus planirostris|ABGYA704-06|ROM 100409|Guyana.East Berbice-Corentyne
Artibeus planirostris|ABGYA200-06|ROM 98068|Guyana.Potaro-Siparuni
Artibeus planirostris|ABGYA573-06|ROM 100253|Guyana.East Berbice-Corentyne
Artibeus planirostris|ABGYA728-06|ROM 100433|Guyana.East Berbice-Corentyne
Artibeus planirostris|ABGYD447-06|ROM 108798|Guyana.Potaro-Siparuni
Artibeus planirostris|ABGYD448-06|ROM 108799|Guyana.Potaro-Siparuni
Artibeus planirostris|ABGYE361-06|ROM 111811|Guyana.Potaro-Siparuni
Artibeus planirostris|ABGYE553-06|ROM 112026|Guyana.Potaro-Siparuni
Artibeus planirostris|ABGYE799-06|ROM 113401|Guyana.Upper Demerara-Berbice
Artibeus planirostris|BCBNC133-06|ROM 115802|Guyana.Potaro-Siparuni
Artibeus planirostris|ABGYA455-06|ROM 98865|Guyana.Barima-Waini
Artibeus planirostris|BCBNT885-06|ROM 115513|Guyana.Essequibo Islands-West Demerara
Artibeus planirostris|BCBNT931-06|ROM 115621|Guyana.Essequibo Islands-West Demerara
Artibeus planirostris|BCBN587-05|ROM 104045|Ecuador.Napo
Artibeus planirostris|ABGYD405-06|ROM 108751|Guyana.Potaro-Siparuni
Artibeus planirostris|ABECA948-06|ROM F40396|Ecuador.Napo
Artibeus planirostris|BCBN738-05|ROM 104433|Ecuador.Napo
Artibeus planirostris|ABECB104-08|ROM 118766|Ecuador
Artibeus planirostris|ABGYA736-06|ROM 100441|Guyana.East Berbice-Corentyne
Artibeus planirostris|ABGYB365-06|ROM 103072|Guyana.Upper Takutu-Upper Essequibo
Artibeus planirostris|BCBNT085-06|ROM 107073|Guyana.Potaro-Siparuni
Artibeus planirostris|ABECA268-06|ROM 105136|Ecuador.Napo
Artibeus planirostris|ABECA639-06|ROM 105692|Ecuador.Napo
Artibeus planirostris|ABECA640-06|ROM 105693|Ecuador.Napo
Artibeus planirostris|ABECB058-06|ROM 106358|Ecuador.Napo
Artibeus planirostris|BCBN588-05|ROM 104046|Ecuador.Napo
Artibeus planirostris|ABECA178-06|ROM 104491|Ecuador.Napo
Artibeus planirostris|ABECA076-06|ROM 104071|Ecuador.Napo
Artibeus planirostris|ABECA478-06|ROM F37730|Ecuador.Napo
Artibeus planirostris|ABECA776-06|ROM 105939|Ecuador.Napo
Artibeus planirostris|BCBN728-05|ROM 104411|Ecuador.Napo
Artibeus planirostris|BCBN598-05|ROM 104070|Ecuador.Napo
Artibeus planirostris|ABECA116-06|ROM 104409|Ecuador.Napo
Artibeus planirostris|BCBN591-05|ROM 104051|Ecuador.Napo
Artibeus planirostris|ABECA734-06|ROM 105880|Ecuador.Napo
Artibeus planirostris|BCBN751-05|ROM 104469|Ecuador.Napo
Artibeus planirostris|ABECA254-06|ROM 105123|Ecuador.Napo
Artibeus planirostris|BCBNT199-06|ROM 107905|Venezuela.Amazonas
Artibeus planirostris|ABGYA141-06|ROM 97985|Guyana.Potaro-Siparuni
Artibeus planirostris|ABGYF090-06|ROM 113621|Guyana.Demerara-Mahaica
Artibeus planirostris|ABGYF168-06|ROM 113725|Guyana.Demerara-Mahaica
Artibeus planirostris|ABGYF169-06|ROM 113726|Guyana.Demerara-Mahaica
Artibeus planirostris|BCBNT812-06|ROM 114120|Suriname.Brokopondo
Artibeus planirostris|ABECA536-06|ROM 105565|Ecuador.Napo
Artibeus planirostris|ABECA269-06|ROM 105137|Ecuador.Napo
Artibeus planirostris|ABECA682-06|ROM F37998|Ecuador.Napo
Artibeus planirostris|BCBN586-05|ROM 104044|Ecuador.Napo
Artibeus planirostris|ABECA947-06|ROM 106138|Ecuador.Napo
Artibeus planirostris|ABGYF015-06|ROM 113505|Guyana.Upper Takutu-Upper Essequibo
Artibeus planirostris|ABGYD044-06|ROM 108267|Guyana.Cuyuni-Mazaruni
Artibeus planirostris|ABGYC024-06|ROM 104801|Guyana.Potaro-Siparuni
Artibeus planirostris|ABGYC738-06|ROM 108139|Guyana.Cuyuni-Mazaruni
Artibeus planirostris|ABGYA001-06|ROM 97769|Guyana.Upper Takutu-Upper Essequibo
Artibeus planirostris|ABECA525-06|ROM 105555|Ecuador.Napo
Artibeus planirostris|ABECA487-06|ROM 105510|Ecuador.Napo
Artibeus planirostris|ABECA277-06|ROM F37456|Ecuador.Napo
Artibeus planirostris|ABECA117-06|ROM 104410|Ecuador.Napo
Artibeus planirostris|ABECA077-06|ROM 104072|Ecuador.Napo
Artibeus planirostris|ABECA259-06|ROM F37437|Ecuador.Napo
Artibeus planirostris|ABECA946-06|ROM 106137|Ecuador.Napo
Artibeus planirostris|ABECA535-06|ROM 105564|Ecuador.Napo
Artibeus planirostris|ABSMS229-06|ROM 117391|Suriname.Sipaliwini
Artibeus planirostris|ABECA686-06|ROM 105745|Ecuador.Napo
Artibeus planirostris|ABECA021-06|ROM 104003|Ecuador.Napo
Artibeus planirostris|BCBN720-05|ROM 104387|Ecuador.Napo
Artibeus planirostris|BCBN749-05|ROM 104467|Ecuador.Napo
Artibeus planirostris|ABGYD209-06|ROM 108540|Guyana.Potaro-Siparuni
Artibeus planirostris|ABGYB230-06|ROM 102932|Guyana.Upper Takutu-Upper Essequibo
Artibeus planirostris|ABGYE465-06|ROM 111932|Guyana.Potaro-Siparuni
Artibeus planirostris|ABECA164-06|ROM 104478|Ecuador.Napo
Artibeus planirostris|ABECA526-06|ROM 105556|Ecuador.Napo
Artibeus planirostris|BCBN750-05|ROM 104468|Ecuador.Napo
Artibeus planirostris|ABGYC444-06|ROM 107268|Guyana.Potaro-Siparuni
Artibeus planirostris|ABECA930-06|ROM F40371|Ecuador.Napo
Artibeus planirostris|ABECA795-06|ROM 105960|Ecuador.Napo
Artibeus planirostris|ABECA253-06|ROM 105121|Ecuador.Napo
Artibeus planirostris|ABSMS390-06|ROM 117552|Suriname.Sipaliwini
Artibeus planirostris|ABSRA318-06|ROM 116907|Suriname.Nickerie
Artibeus planirostris|ABSRA428-06|ROM 117017|Suriname.Nickerie
Artibeus planirostris|ABECA748-06|ROM 105897|Ecuador.Napo
Artibeus planirostris|ABGYC118-06|ROM 106630|Guyana.Upper Takutu-Upper Essequibo
Artibeus planirostris|ABGYD086-06|ROM 108383|Guyana.Potaro-Siparuni
Artibeus planirostris|ABGYF186-06|ROM 113747|Guyana.Demerara-Mahaica
Artibeus planirostris|ABGYG944-08|ROM 119372|Guyana

Artibeus planirostris|ABGYD086-06|ROM 108383|Guyana.Potaro-Siparuni
Artibeus planirostris|ABGYF186-06|ROM 113747|Guyana.Demerara-Mahaica
Artibeus planirostris|ABGYG944-08|ROM 119372|Guyana
Artibeus planirostris|ABECA800-06|ROM 105966|Ecuador.Napo
Artibeus planirostris|ABECA939-06|ROM 106122|Ecuador.Napo
Artibeus planirostris|ABECA794-06|ROM 105959|Ecuador.Napo
Artibeus planirostris|BCBN729-05|ROM 104412|Ecuador.Napo
Artibeus planirostris|BCBNT330-06|ROM 108904|Guyana.Potaro-Siparuni
Artibeus planirostris|ABGYC777-06|ROM 108180|Guyana.Cuyuni-Mazaruni
Artibeus planirostris|ABGYE671-06|ROM 112593|Guyana.Demerara-Mahaica
Artibeus planirostris|ABGYA786-06|ROM 100839|Guyana.Barima-Waini
Artibeus planirostris|ABGYA762-06|ROM 100812|Guyana.Barima-Waini
Artibeus planirostris|ABGYA815-06|ROM 100874|Guyana.Barima-Waini
Artibeus planirostris|ABGYA805-06|ROM 100863|Guyana.Barima-Waini
Artibeus planirostris|ABGYC496-06|ROM 107329|Guyana.Potaro-Siparuni
Artibeus planirostris|ABGYC034-06|ROM 104811|Guyana.Potaro-Siparuni
Artibeus planirostris|ABGYB680-06|ROM 103444|Guyana.Upper Demerara-Berbice
Artibeus planirostris|ABGYA869-06|ROM 100944|Guyana.Barima-Waini
Artibeus planirostris|ABGYA646-06|ROM 100342|Guyana.East Berbice-Corentyne
Artibeus planirostris|ABGYA474-06|ROM 98884|Guyana.Barima-Waini
Artibeus planirostris|ABGYC739-06|ROM 108140|Guyana.Cuyuni-Mazaruni
Artibeus planirostris|ABGYC804-06|ROM 108202|Guyana.Cuyuni-Mazaruni
Artibeus planirostris|ABGYD009-06|ROM 108231|Guyana.Cuyuni-Mazaruni
Artibeus planirostris|ABGYD046-06|ROM 108269|Guyana.Cuyuni-Mazaruni
Artibeus planirostris|ABGYD078-06|ROM 108375|Guyana.Potaro-Siparuni
Artibeus planirostris|ABGYD160-06|ROM 108472|Guyana.Potaro-Siparuni
Artibeus planirostris|ABGYE751-06|ROM 112697|Guyana.Demerara-Mahaica
Artibeus planirostris|ABGYF123-06|ROM 113669|Guyana.Demerara-Mahaica
Artibeus planirostris|ABGYF172-06|ROM 113729|Guyana.Demerara-Mahaica
Artibeus planirostris|ABGYF266-06|ROM 113854|Guyana.Demerara-Mahaica
Artibeus planirostris|ABGYG1005-08|ROM 118929|Guyana
Artibeus planirostris|ABGYG1059-08|ROM 118983|Guyana
Artibeus planirostris|BCBN357-05|ROM 101053|Guyana.Barima-Waini
Artibeus planirostris|BCBNT341-06|ROM 108936|Guyana.Potaro-Siparuni
Artibeus planirostris|ABGYA672-06|ROM 100376|Guyana.East Berbice-Corentyne
Artibeus planirostris|ABGYB362-06|ROM 103069|Guyana.Upper Takutu-Upper Essequibo
Artibeus planirostris|BCBNT532-06|ROM 111927|Guyana.Potaro-Siparuni
Artibeus planirostris|ABGYA142-06|ROM 97986|Guyana.Potaro-Siparuni
Artibeus planirostris|ABGYA731-06|ROM 100436|Guyana.East Berbice-Corentyne
Artibeus planirostris|ABGYB224-06|ROM 102926|Guyana.Upper Takutu-Upper Essequibo
Artibeus planirostris|ABGYD169-06|ROM 108489|Guyana.Potaro-Siparuni
Artibeus planirostris|ABSMS254-06|ROM 117416|Suriname.Sipaliwini
Artibeus planirostris|ABSRA468-06|ROM 117058|Suriname.Nickerie
Artibeus planirostris|ABGYB933-07|ROM 103036|Guyana.Upper Takutu-Upper Essequibo
Artibeus planirostris|ABGYF167-06|ROM 113724|Guyana.Demerara-Mahaica
Artibeus planirostris|ABGYF265-06|ROM 113853|Guyana.Demerara-Mahaica
Artibeus planirostris|ABGYD006-06|ROM F43275|Guyana.Cuyuni-Mazaruni
Artibeus planirostris|ABGYF130-06|ROM 113678|Guyana.Demerara-Mahaica
Artibeus planirostris|ABGYC795-06|ROM 108196|Guyana.Cuyuni-Mazaruni
Artibeus planirostris|ABGYC050-06|ROM 104827|Guyana.Potaro-Siparuni
Artibeus planirostris|ABGYB322-06|ROM 103027|Guyana.Upper Takutu-Upper Essequibo
Artibeus planirostris|ABGYB250-06|ROM 102954|Guyana.Upper Takutu-Upper Essequibo
Artibeus planirostris|ABGYA676-06|ROM 100381|Guyana.East Berbice-Corentyne
Artibeus planirostris|ABGYA648-06|ROM 100344|Guyana.East Berbice-Corentyne
Artibeus planirostris|ABGYA638-06|ROM 100332|Guyana.East Berbice-Corentyne
Artibeus planirostris|ABGYA509-06|ROM 98920|Guyana.Barima-Waini
Artibeus planirostris|ABGYA730-06|ROM 100435|Guyana.East Berbice-Corentyne
Artibeus planirostris|ABSMS244-06|ROM 117406|Suriname.Sipaliwini
Artibeus planirostris|ABSMS253-06|ROM 117415|Suriname.Sipaliwini
Artibeus planirostris|BCBN356-05|ROM 101034|Guyana.Barima-Waini
Artibeus planirostris|ABGYA120-06|ROM 97960|Guyana.Upper Takutu-Upper Essequibo
Artibeus planirostris|ABGYB202-06|ROM 102904|Guyana.Upper Takutu-Upper Essequibo
Artibeus planirostris|ABGYG796-08|ROM 119224|Guyana
Artibeus planirostris|ABGYB339-06|ROM 103046|Guyana.Upper Takutu-Upper Essequibo
Artibeus planirostris|ABSMS112-06|ROM 117274|Suriname.Sipaliwini
Artibeus planirostris|ABGYA143-06|ROM 97987|Guyana.Potaro-Siparuni
Artibeus planirostris|ABGYD139-06|ROM 108442|Guyana.Potaro-Siparuni
Artibeus planirostris|ABGYD378-06|ROM 108723|Guyana.Potaro-Siparuni
Artibeus planirostris|ABGYC511-06|ROM 107347|Guyana.Potaro-Siparuni
Artibeus planirostris|ABGYG1024-08|ROM 118948|Guyana
Artibeus planirostris|BCBNT188-06|ROM 107888|Venezuela.Amazonas
Artibeus planirostris|ABGYG798-08|ROM 119226|Guyana
Artibeus planirostris|BCBNT192-06|ROM 107893|Venezuela.Amazonas
Artibeus planirostris|ABSRA323-06|ROM 116912|Suriname.Nickerie
Artibeus planirostris|ABGYC109-06|ROM 106619|Guyana.Upper Takutu-Upper Essequibo
Artibeus planirostris|ABGYG612-08|ROM 119040|Guyana
Artibeus planirostris|ABSMS061-06|ROM 117223|Suriname.Sipaliwini
Artibeus planirostris|ABSRA359-06|ROM 116948|Suriname.Nickerie
Artibeus planirostris|ABGYG1025-08|ROM 118949|Guyana
Artibeus planirostris|ABGYG1035-08|ROM 118959|Guyana
Artibeus planirostris|ABGYF135-06|ROM 113686|Guyana.Demerara-Mahaica
Artibeus planirostris|ABGYF261-06|ROM 113849|Guyana.Demerara-Mahaica
Artibeus planirostris|ABGYE696-06|ROM 112629|Guyana.Demerara-Mahaica
Artibeus planirostris|ABGYF122-06|ROM 113668|Guyana.Demerara-Mahaica
Artibeus planirostris|ABGYA868-06|ROM 100941|Guyana.Barima-Waini
Artibeus planirostris|ABGYB154-06|ROM 101143|Guyana.Barima-Waini
Artibeus planirostris|ABGYA818-06|ROM 100877|Guyana.Barima-Waini
Artibeus planirostris|ABGYA835-06|ROM 100898|Guyana.Barima-Waini
Artibeus planirostris|ABGYA763-06|ROM 100813|Guyana.Barima-Waini
Artibeus planirostris|ABGYA814-06|ROM 100873|Guyana.Barima-Waini
Artibeus planirostris|ABGYA703-06|ROM 100408|Guyana.East Berbice-Corentyne
Artibeus planirostris|ABGYA649-06|ROM 100345|Guyana.East Berbice-Corentyne
Artibeus planirostris|ABGYA637-06|ROM 100331|Guyana.East Berbice-Corentyne
Artibeus planirostris|ABGYA623-06|ROM 100317|Guyana.East Berbice-Corentyne
Artibeus planirostris|ABGYA442-06|ROM 98851|Guyana.Barima-Waini
Artibeus planirostris|ABGYA854-06|ROM 100924|Guyana.Barima-Waini
Artibeus planirostris|ABGYA406-06|ROM 98814|Guyana.Barima-Waini
Artibeus planirostris|ABGYB166-06|ROM 101157|Guyana.Barima-Waini
Artibeus planirostris|ABGYA566-06|ROM 100242|Guyana.East Berbice-Corentyne
Artibeus planirostris|ABSMS211-06|ROM 117373|Suriname.Sipaliwini
Artibeus planirostris|ABSRA396-06|ROM 116985|Suriname.Nickerie
Artibeus planirostris|BCBN369-05|ROM 101142|Guyana.Barima-Waini
Artibeus planirostris|BCBNT616-06|ROM 113530|Guyana.Upper Takutu-Upper Essequibo

[illegible]|Suriname.Sipaliwini
Artibeus planirostris|ABSRA396-06|ROM 116985|Suriname.Nickerie
Artibeus planirostris|BCBN369-05|ROM 101142|Guyana.Barima-Waini
Artibeus planirostris|BCBNT616-06|ROM 113530|Guyana.Upper Takutu-Upper Essequibo
Artibeus planirostris|BCBNT534-06|ROM 111941|Guyana.Potaro-Siparuni
Artibeus planirostris|ABSMS385-06|ROM 117547|Suriname.Sipaliwini
Artibeus planirostris|ABRMM082-07|ROM F39696|Guyana.Potaro-Siparuni
Artibeus planirostris|ABGYG925-08|ROM 119353|Guyana
Artibeus planirostris|ABGYG770-08|ROM 119198|Guyana
Artibeus planirostris|ABGYE443-06|ROM 111904|Guyana.Potaro-Siparuni
Artibeus planirostris|ABGYC779-06|ROM 108182|Guyana.Cuyuni-Mazaruni
Artibeus planirostris|ABGYG898-08|ROM 119326|Guyana
Artibeus planirostris|ABGYC778-06|ROM 108181|Guyana.Cuyuni-Mazaruni
Artibeus planirostris|BCBNT723-06|ROM 113894|Suriname.Brokopondo
Artibeus planirostris|ABGYD045-06|ROM 108268|Guyana.Cuyuni-Mazaruni
Artibeus planirostris|ABGYE099-06|ROM 111542|Guyana.Potaro-Siparuni
Artibeus planirostris|ABGYG853-08|ROM 119281|Guyana
Artibeus planirostris|ABGYG946-08|ROM 119374|Guyana
Artibeus planirostris|ABGYC169-06|ROM 106698|Guyana.Upper Takutu-Upper Essequibo
Artibeus planirostris|ABGYG895-08|ROM 119323|Guyana
Artibeus planirostris|ABSRA334-06|ROM 116923|Suriname.Nickerie
Artibeus planirostris|ABGYF239-06|ROM 113818|Guyana.Demerara-Mahaica
Artibeus planirostris|ABGYE800-06|ROM 113402|Guyana.Upper Demerara-Berbice
Artibeus planirostris|ABGYE060-06|ROM 109346|Guyana.Potaro-Siparuni
Artibeus planirostris|ABGYD480-06|ROM 108833|Guyana.Potaro-Siparuni
Artibeus planirostris|ABGYD446-06|ROM 108797|Guyana.Potaro-Siparuni
Artibeus planirostris|ABGYD406-06|ROM 108752|Guyana.Potaro-Siparuni
Artibeus planirostris|ABGYC484-06|ROM 107317|Guyana.Potaro-Siparuni
Artibeus planirostris|ABGYG795-08|ROM 119223|Guyana
Artibeus planirostris|ABGYD267-06|ROM 108603|Guyana.Potaro-Siparuni
Artibeus planirostris|ABGYB321-06|ROM 103026|Guyana.Upper Takutu-Upper Essequibo
Artibeus planirostris|ABGYC503-06|ROM 107338|Guyana.Potaro-Siparuni
Artibeus planirostris|ABGYB596-06|ROM 103337|Guyana.Upper Takutu-Upper Essequibo
Artibeus planirostris|ABGYB223-06|ROM 102925|Guyana.Upper Takutu-Upper Essequibo
Artibeus planirostris|ABGYG845-08|ROM 119273|Guyana
Artibeus planirostris|ABSMS372-06|ROM 117534|Suriname.Sipaliwini
Artibeus planirostris|ABGYA640-06|ROM 100334|Guyana.East Berbice-Corentyne
Artibeus planirostris|ABGYD153-06|ROM 108459|Guyana.Potaro-Siparuni
Artibeus planirostris|ABGYD297-06|ROM 108636|Guyana.Potaro-Siparuni
Artibeus planirostris|ABGYD496-06|ROM 108854|Guyana.Potaro-Siparuni
Artibeus planirostris|ABGYD002-06|ROM F43271|Guyana.Cuyuni-Mazaruni
Artibeus planirostris|ABGYC761-06|ROM F43189|Guyana.Cuyuni-Mazaruni
Artibeus planirostris|ABGYD003-06|ROM F43272|Guyana.Cuyuni-Mazaruni
Artibeus planirostris|ABGYD005-06|ROM F43274|Guyana.Cuyuni-Mazaruni
Artibeus planirostris|ABGYD226-06|ROM 108557|Guyana.Potaro-Siparuni
Artibeus planirostris|ABGYD292-06|ROM 108631|Guyana.Potaro-Siparuni
Artibeus planirostris|ABGYE389-06|ROM 111841|Guyana.Potaro-Siparuni
Artibeus planirostris|ABSMS325-06|ROM 117487|Suriname.Sipaliwini
Artibeus planirostris|ABSRA502-06|ROM 117092|Suriname.Nickerie
Artibeus planirostris|ABGYG582-06|ROM 116745|Guyana.Potaro-Siparuni
Artibeus planirostris|ABGYG646-08|ROM 119074|Guyana
Artibeus planirostris|ABGYG854-08|ROM 119282|Guyana
Artibeus planirostris|ABSMS123-06|ROM 117285|Suriname.Sipaliwini
Artibeus planirostris|BCBNT073-06|ROM 107028|Guyana.Potaro-Siparuni
Artibeus planirostris|BCBNT531-06|ROM 111926|Guyana.Potaro-Siparuni
Artibeus planirostris|ABGYF268-06|ROM 113856|Guyana.Demerara-Mahaica
Artibeus planirostris|ABGYG580-06|ROM 116743|Guyana.Potaro-Siparuni
Artibeus planirostris|BCBNT997-06|ROM 114685|Guyana.Potaro-Siparuni
Artibeus obscurus|ABGYG1047-08|ROM 118971|Guyana
Artibeus obscurus|ABGYG1046-08|ROM 118970|Guyana
Artibeus obscurus|ABGYC185-06|ROM 106720|Guyana.Upper Takutu-Upper Essequibo
Artibeus obscurus|ABGYC033-06|ROM 104810|Guyana.Potaro-Siparuni
Artibeus obscurus|ABGYC168-06|ROM 106696|Guyana.Upper Takutu-Upper Essequibo
Artibeus obscurus|ABGYC184-06|ROM 106719|Guyana.Upper Takutu-Upper Essequibo
Artibeus obscurus|ABGYD571-06|ROM 108947|Guyana.Potaro-Siparuni
Artibeus obscurus|BCBNT333-06|ROM 108907|Guyana.Potaro-Siparuni
Artibeus obscurus|ABSMS374-06|ROM 117536|Suriname.Sipaliwini
Artibeus obscurus|BCBN124-05|ROM 97860|Guyana.Upper Takutu-Upper Essequibo
Artibeus obscurus|ABSMS060-06|ROM 117222|Suriname.Sipaliwini
Artibeus obscurus|ABSMS097-06|ROM 117259|Suriname.Sipaliwini
Artibeus obscurus|ABGYG126-06|ROM 115680|Guyana.Potaro-Siparuni
Artibeus obscurus|ABSMS018-06|ROM 117180|Suriname.Sipaliwini
Artibeus obscurus|ABGYF048-06|ROM 113557|Guyana.Upper Takutu-Upper Essequibo
Artibeus obscurus|ABGYF050-06|ROM 113559|Guyana.Upper Takutu-Upper Essequibo
Artibeus obscurus|ABGYF033-06|ROM 113536|Guyana.Upper Takutu-Upper Essequibo
Artibeus obscurus|ABGYF038-06|ROM 113545|Guyana.Upper Takutu-Upper Essequibo
Artibeus obscurus|ABGYE856-06|ROM 113472|Guyana.Upper Takutu-Upper Essequibo
Artibeus obscurus|ABGYF002-06|ROM 113486|Guyana.Upper Takutu-Upper Essequibo
Artibeus obscurus|ABGYE486-06|ROM 111958|Guyana.Potaro-Siparuni
Artibeus obscurus|ABGYE851-06|ROM 113467|Guyana.Upper Takutu-Upper Essequibo
Artibeus obscurus|ABGYD724-06|ROM 109140|Guyana.Potaro-Siparuni
Artibeus obscurus|ABGYD162-06|ROM 108474|Guyana.Potaro-Siparuni
Artibeus obscurus|ABGYC426-06|ROM 107248|Guyana.Potaro-Siparuni
Artibeus obscurus|ABGYC398-06|ROM 107217|Guyana.Potaro-Siparuni
Artibeus obscurus|ABGYB272-06|ROM 102976|Guyana.Upper Takutu-Upper Essequibo
Artibeus obscurus|ABGYA262-06|ROM 98162|Guyana.Potaro-Siparuni
Artibeus obscurus|ABGYD770-06|ROM 109190|Guyana.Potaro-Siparuni
Artibeus obscurus|ABGYG836-08|ROM 119264|Guyana
Artibeus obscurus|ABGYD761-06|ROM 109181|Guyana.Potaro-Siparuni
Artibeus obscurus|ABGYD781-06|ROM 109202|Guyana.Potaro-Siparuni
Artibeus obscurus|ABGYE259-06|ROM 111708|Guyana.Potaro-Siparuni
Artibeus obscurus|ABGYC659-06|ROM 107479|Guyana.Potaro-Siparuni
Artibeus obscurus|ABGYD581-06|ROM 108961|Guyana.Potaro-Siparuni
Artibeus obscurus|ABGYD587-06|ROM 109007|Guyana.Potaro-Siparuni
Artibeus obscurus|ABGYE477-06|ROM 111949|Guyana.Potaro-Siparuni
Artibeus obscurus|ABGYE599-06|ROM 112078|Guyana.Potaro-Siparuni
Artibeus obscurus|ABGYE066-06|ROM 111509|Guyana.Potaro-Siparuni
Artibeus obscurus|ABGYF051-06|ROM 113560|Guyana.Upper Takutu-Upper Essequibo
Artibeus obscurus|ABSMS042-06|ROM 117204|Suriname.Sipaliwini
Artibeus obscurus|ABSRA279-06|ROM 116868|Suriname.Nickerie
Artibeus obscurus|BCBNT350-06|ROM 109012|Guyana.Potaro-Siparuni
Artibeus obscurus|BCBNT613-06|ROM 113523|Guyana.Upper Takutu-Upper Essequibo
Artibeus obscurus|BCBNT736-06|ROM 113911|Suriname.Brokopondo
[illegible]

Artibeus obscurus|BCBNT350-06|ROM 109012|Guyana.Potaro-Siparuni
Artibeus obscurus|BCBNT613-06|ROM 113523|Guyana.Upper Takutu-Upper Essequibo
Artibeus obscurus|BCBNT736-06|ROM 113911|Suriname.Brokopondo
Artibeus obscurus|BCBNT746-06|ROM 113927|Suriname.Brokopondo
Artibeus obscurus|ABECA932-06|ROM 106115|Ecuador.Napo
Artibeus obscurus|ABECA940-06|ROM F40381|Ecuador.Napo
Artibeus obscurus|BCBN745-05|ROM 104458|Ecuador.Napo
Artibeus obscurus|BCBN904-05|ROM 105764|Ecuador.Napo
Artibeus obscurus|ABECA933-06|ROM 106116|Ecuador.Napo
Artibeus obscurus|BCBN597-05|ROM 104069|Ecuador.Napo
Artibeus obscurus|ABECA685-06|ROM F40031|Ecuador.Napo
Artibeus obscurus|ABECA805-06|ROM 105971|Ecuador.Napo
Artibeus obscurus|ABECA559-06|ROM 105588|Ecuador.Napo
Artibeus obscurus|ABECA560-06|ROM 105589|Ecuador.Napo
Artibeus obscurus|ABECA527-06|ROM 105557|Ecuador.Napo
Artibeus obscurus|ABECA316-06|ROM 105196|Ecuador.Napo
Artibeus obscurus|ABECA306-06|ROM 105180|Ecuador.Napo
Artibeus obscurus|ABECA282-06|ROM 105151|Ecuador.Napo
Artibeus obscurus|ABECA230-06|ROM F37407|Ecuador.Napo
Artibeus obscurus|ABECA203-06|ROM 104524|Ecuador.Napo
Artibeus obscurus|ABECA202-06|ROM 104523|Ecuador.Napo
Artibeus obscurus|ABECA035-06|ROM 104017|Ecuador.Napo
Artibeus obscurus|ABECA034-06|ROM 104016|Ecuador.Napo
Artibeus obscurus|ABECA311-06|ROM 105188|Ecuador.Napo
Artibeus obscurus|ABECA823-06|ROM F40245|Ecuador.Napo
Artibeus obscurus|ABECA195-06|ROM 104513|Ecuador.Napo
Artibeus obscurus|ABECA528-06|ROM 105558|Ecuador.Napo
Artibeus obscurus|ABECA260-06|ROM F37438|Ecuador.Napo
Artibeus obscurus|ABECA763-06|ROM 105916|Ecuador.Napo
Artibeus obscurus|ABECA765-06|ROM 105918|Ecuador.Napo
Artibeus obscurus|BCBN776-05|ROM 105146|Ecuador.Napo
Artibeus obscurus|ABECA058-06|ROM F37149|Ecuador.Napo
Artibeus obscurus|ABECA143-06|ROM 104442|Ecuador.Napo
Artibeus obscurus|ABECA286-06|ROM 105157|Ecuador.Napo
Artibeus obscurus|ABECA489-06|ROM F37744|Ecuador.Napo
Artibeus obscurus|ABECA764-06|ROM 105917|Ecuador.Napo
Artibeus obscurus|ABECA204-06|ROM 104525|Ecuador.Napo
Artibeus obscurus|ABECA327-06|ROM 105209|Ecuador.Napo
Artibeus obscurus|ABECA099-06|ROM 104386|Ecuador.Napo
Artibeus obscurus|BCBN961-05|ROM 105938|Ecuador.Napo
Artibeus obscurus|BCBNT172-06|ROM 107846|Venezuela.Amazonas
Artibeus obscurus|BCBNT222-06|ROM 107937|Venezuela.Bolivar
Artibeus obscurus|ABGYC011-06|ROM 104788|Guyana.Potaro-Siparuni
Artibeus obscurus|ABGYC028-06|ROM 104805|Guyana.Potaro-Siparuni
Artibeus obscurus|ABGYC049-06|ROM 104826|Guyana.Potaro-Siparuni
Artibeus obscurus|ABGYG1041-08|ROM 118965|Guyana
Artibeus obscurus|ABGYA888-07|ROM 103035|Guyana.Upper Takutu-Upper Essequibo
Artibeus obscurus|ABGYC009-06|ROM 104786|Guyana.Potaro-Siparuni
Artibeus obscurus|BCBNT524-06|ROM 111887|Guyana.Potaro-Siparuni
Artibeus obscurus|ABGYC055-06|ROM 104832|Guyana.Potaro-Siparuni
Artibeus obscurus|ABGYE266-06|ROM 111715|Guyana.Potaro-Siparuni
Artibeus obscurus|ABGYE771-06|ROM 113372|Guyana.Upper Demerara-Berbice
Artibeus obscurus|ABGYE787-06|ROM 113388|Guyana.Upper Demerara-Berbice
Artibeus obscurus|ABGYE256-06|ROM 111705|Guyana.Potaro-Siparuni
Artibeus obscurus|ABGYE494-06|ROM 111966|Guyana.Potaro-Siparuni
Artibeus obscurus|ABGYD591-06|ROM 109011|Guyana.Potaro-Siparuni
Artibeus obscurus|ABGYE146-06|ROM 111589|Guyana.Potaro-Siparuni
Artibeus obscurus|ABGYD579-06|ROM 108959|Guyana.Potaro-Siparuni
Artibeus obscurus|ABGYD545-06|ROM 108910|Guyana.Potaro-Siparuni
Artibeus obscurus|ABGYD544-06|ROM 108909|Guyana.Potaro-Siparuni
Artibeus obscurus|ABGYD228-06|ROM 108559|Guyana.Potaro-Siparuni
Artibeus obscurus|ABGYD145-06|ROM 108451|Guyana.Potaro-Siparuni
Artibeus obscurus|ABGYB931-06|ROM 104776|Guyana.Potaro-Siparuni
Artibeus obscurus|ABGYB782-06|ROM 103550|Guyana.Upper Demerara-Berbice
Artibeus obscurus|ABGYB779-06|ROM 103547|Guyana.Upper Demerara-Berbice
Artibeus obscurus|ABGYB622-06|ROM 103377|Guyana.Upper Demerara-Berbice
Artibeus obscurus|ABGYB385-06|ROM 103092|Guyana.Upper Takutu-Upper Essequibo
Artibeus obscurus|ABGYB359-06|ROM 103066|Guyana.Upper Takutu-Upper Essequibo
Artibeus obscurus|ABGYB327-06|ROM 103032|Guyana.Upper Takutu-Upper Essequibo
Artibeus obscurus|ABGYB270-06|ROM 102974|Guyana.Upper Takutu-Upper Essequibo
Artibeus obscurus|ABGYB329-06|ROM 103034|Guyana.Upper Takutu-Upper Essequibo
Artibeus obscurus|ABGYD339-06|ROM 108679|Guyana.Potaro-Siparuni
Artibeus obscurus|ABGYD616-06|ROM 108990|Guyana.Potaro-Siparuni
Artibeus obscurus|ABGYE808-06|ROM 113411|Guyana.Upper Demerara-Berbice
Artibeus obscurus|ABGYC207-06|ROM 106746|Guyana.Upper Takutu-Upper Essequibo
Artibeus obscurus|ABGYE855-06|ROM 113471|Guyana.Upper Takutu-Upper Essequibo
Artibeus obscurus|ABGYG1060-08|ROM 118984|Guyana
Artibeus obscurus|ABGYA372-06|ROM 98773|Guyana.Barima-Waini
Artibeus obscurus|ABGYA435-06|ROM 98843|Guyana.Barima-Waini
Artibeus obscurus|ABGYA484-06|ROM 98894|Guyana.Barima-Waini
Artibeus obscurus|ABSRA480-06|ROM 117070|Suriname.Nickerie
Artibeus obscurus|ABGYG116-06|ROM 115666|Guyana.Potaro-Siparuni
Artibeus obscurus|ABGYE809-06|ROM 113412|Guyana.Upper Demerara-Berbice
Artibeus obscurus|ABGYE289-06|ROM 111738|Guyana.Potaro-Siparuni
Artibeus obscurus|ABGYE080-06|ROM 111523|Guyana.Potaro-Siparuni
Artibeus obscurus|ABGYD555-06|ROM 108925|Guyana.Potaro-Siparuni
Artibeus obscurus|ABGYD358-06|ROM 108701|Guyana.Potaro-Siparuni
Artibeus obscurus|ABGYC487-06|ROM 107320|Guyana.Potaro-Siparuni
Artibeus obscurus|ABGYC486-06|ROM 107319|Guyana.Potaro-Siparuni
Artibeus obscurus|ABGYB780-06|ROM 103548|Guyana.Upper Demerara-Berbice
Artibeus obscurus|ABGYB164-06|ROM 101155|Guyana.Barima-Waini
Artibeus obscurus|ABGYB153-06|ROM 101141|Guyana.Barima-Waini
Artibeus obscurus|ABGYA823-06|ROM 100882|Guyana.Barima-Waini
Artibeus obscurus|ABGYA787-06|ROM 100840|Guyana.Barima-Waini
Artibeus obscurus|ABGYA487-06|ROM 98897|Guyana.Barima-Waini
Artibeus obscurus|ABGYA486-06|ROM 98896|Guyana.Barima-Waini
Artibeus obscurus|ABGYA471-06|ROM 98881|Guyana.Barima-Waini
Artibeus obscurus|ABGYA470-06|ROM 98880|Guyana.Barima-Waini
Artibeus obscurus|ABGYA468-06|ROM 98878|Guyana.Barima-Waini
Artibeus obscurus|ABGYA467-06|ROM 98877|Guyana.Barima-Waini
Artibeus obscurus|ABGYA465-06|ROM 98875|Guyana.Barima-Waini
Artibeus obscurus|ABGYA443-06|ROM 98852|Guyana.Barima-Waini
Artibeus obscurus|ABGYA436-06|ROM 98844|Guyana.Barima-Waini
[illegible]

Artibeus obscurus|ABGYA465-06|ROM 98875|Guyana.Barima-Waini
Artibeus obscurus|ABGYA443-06|ROM 98852|Guyana.Barima-Waini
Artibeus obscurus|ABGYA436-06|ROM 98844|Guyana.Barima-Waini
Artibeus obscurus|ABGYA432-06|ROM 98840|Guyana.Barima-Waini
Artibeus obscurus|ABGYA428-06|ROM 98836|Guyana.Barima-Waini
Artibeus obscurus|ABGYA412-06|ROM 98820|Guyana.Barima-Waini
Artibeus obscurus|ABGYA411-06|ROM 98819|Guyana.Barima-Waini
Artibeus obscurus|ABGYA409-06|ROM 98817|Guyana.Barima-Waini
Artibeus obscurus|ABGYA408-06|ROM 98816|Guyana.Barima-Waini
Artibeus obscurus|ABGYA407-06|ROM 98815|Guyana.Barima-Waini
Artibeus obscurus|ABGYA401-06|ROM 98809|Guyana.Barima-Waini
Artibeus obscurus|ABGYA398-06|ROM 98806|Guyana.Barima-Waini
Artibeus obscurus|ABGYA361-06|ROM 98762|Guyana.Barima-Waini
Artibeus obscurus|ABGYA466-06|ROM 98876|Guyana.Barima-Waini
Artibeus obscurus|ABGYA806-06|ROM 100864|Guyana.Barima-Waini
Artibeus obscurus|ABGYA346-06|ROM 98746|Guyana.Barima-Waini
Artibeus obscurus|ABGYA404-06|ROM 98812|Guyana.Barima-Waini
Artibeus obscurus|ABGYA469-06|ROM 98879|Guyana.Barima-Waini
Artibeus obscurus|ABGYA473-06|ROM 98883|Guyana.Barima-Waini
Artibeus obscurus|ABGYA402-06|ROM 98810|Guyana.Barima-Waini
Artibeus obscurus|ABGYA403-06|ROM 98811|Guyana.Barima-Waini
Artibeus obscurus|ABGYA414-06|ROM 98822|Guyana.Barima-Waini
Artibeus obscurus|ABGYA433-06|ROM 98841|Guyana.Barima-Waini
Artibeus obscurus|ABGYA434-06|ROM 98842|Guyana.Barima-Waini
Artibeus obscurus|BCBN339-05|ROM 100926|Guyana.Barima-Waini
Artibeus obscurus|BCBN344-05|ROM 100955|Guyana.Barima-Waini
Artibeus obscurus|BCBNT334-06|ROM 108908|Guyana.Potaro-Siparuni
Artibeus obscurus|BCBNT884-06|ROM 115512|Guyana.Essequibo Islands-West Demerara
Artibeus obscurus|ABGYE258-06|ROM 111707|Guyana.Potaro-Siparuni
Artibeus obscurus|ABGYB632-06|ROM 103387|Guyana.Upper Demerara-Berbice
Artibeus obscurus|ABSMS138-06|ROM 117300|Suriname.Sipaliwini
Artibeus obscurus|ABGYE853-06|ROM 113469|Guyana.Upper Takutu-Upper Essequibo
Artibeus obscurus|ABGYG1042-08|ROM 118966|Guyana
Artibeus obscurus|ABGYG1013-08|ROM 118937|Guyana
Artibeus obscurus|ABGYC208-06|ROM 106747|Guyana.Upper Takutu-Upper Essequibo
Artibeus obscurus|ABGYC145-06|ROM 106661|Guyana.Upper Takutu-Upper Essequibo
Artibeus obscurus|ABGYG1064-08|ROM 118988|Guyana
Artibeus obscurus|ABGYC821-06|ROM 108225|Guyana.Cuyuni-Mazaruni
Artibeus obscurus|ABGYD010-06|ROM 108232|Guyana.Cuyuni-Mazaruni
Artibeus obscurus|ABGYC797-06|ROM F43234|Guyana.Cuyuni-Mazaruni
Artibeus obscurus|ABGYC798-06|ROM F43235|Guyana.Cuyuni-Mazaruni
Artibeus obscurus|ABGYC782-06|ROM 108183|Guyana.Cuyuni-Mazaruni
Artibeus obscurus|ABGYC766-06|ROM 108168|Guyana.Cuyuni-Mazaruni
Artibeus obscurus|ABGYC765-06|ROM 108167|Guyana.Cuyuni-Mazaruni
Artibeus obscurus|ABGYC716-06|ROM 108116|Guyana.Cuyuni-Mazaruni
Artibeus obscurus|ABGYC707-06|ROM 108106|Guyana.Cuyuni-Mazaruni
Artibeus obscurus|ABGYB668-06|ROM 103431|Guyana.Upper Demerara-Berbice
Artibeus obscurus|ABGYA749-06|ROM 100455|Guyana.East Berbice-Corentyne
Artibeus obscurus|ABGYA579-06|ROM 100264|Guyana.East Berbice-Corentyne
Artibeus obscurus|ABGYA548-06|ROM 100220|Guyana.East Berbice-Corentyne
Artibeus obscurus|ABGYA399-06|ROM 98807|Guyana.Barima-Waini
Artibeus obscurus|ABGYC781-06|ROM F43213|Guyana.Cuyuni-Mazaruni
Artibeus obscurus|ABGYC796-06|ROM 108197|Guyana.Cuyuni-Mazaruni
Artibeus obscurus|ABGYC799-06|ROM 108198|Guyana.Cuyuni-Mazaruni
Artibeus obscurus|ABGYD011-06|ROM 108233|Guyana.Cuyuni-Mazaruni
Artibeus obscurus|ABGYA605-06|ROM 100292|Guyana.East Berbice-Corentyne
Artibeus obscurus|ABGYG557-06|ROM 116706|Guyana.Potaro-Siparuni
Artibeus obscurus|ABGYA485-06|ROM 98895|Guyana.Barima-Waini
Artibeus obscurus|ABGYA785-06|ROM 100838|Guyana.Barima-Waini
Artibeus obscurus|ABGYC691-06|ROM 108090|Guyana.Cuyuni-Mazaruni
Artibeus obscurus|ABGYC692-06|ROM 108091|Guyana.Cuyuni-Mazaruni
Artibeus obscurus|ABGYC717-06|ROM 108117|Guyana.Cuyuni-Mazaruni
Artibeus obscurus|ABSRA427-06|ROM 117016|Suriname.Nickerie
Artibeus obscurus|ABGYE419-06|ROM 111876|Guyana.Potaro-Siparuni
Artibeus obscurus|ABGYD229-06|ROM 108560|Guyana.Potaro-Siparuni
Artibeus obscurus|ABGYD227-06|ROM 108558|Guyana.Potaro-Siparuni
Artibeus obscurus|ABGYD111-06|ROM 108409|Guyana.Potaro-Siparuni
Artibeus obscurus|ABGYC458-06|ROM 107288|Guyana.Potaro-Siparuni
Artibeus obscurus|ABGYE498-06|ROM 111970|Guyana.Potaro-Siparuni
Artibeus obscurus|ABGYG555-06|ROM 116702|Guyana.Potaro-Siparuni
Artibeus obscurus|ABGYD743-06|ROM 109161|Guyana.Potaro-Siparuni
Artibeus obscurus|ABGYG117-06|ROM 115667|Guyana.Potaro-Siparuni
Artibeus obscurus|ABGYD580-06|ROM 108960|Guyana.Potaro-Siparuni
Artibeus obscurus|ABGYC658-06|ROM 107478|Guyana.Potaro-Siparuni
Artibeus obscurus|ABGYB403-06|ROM 103110|Guyana.Upper Takutu-Upper Essequibo
Artibeus obscurus|ABGYB395-06|ROM 103102|Guyana.Upper Takutu-Upper Essequibo
Artibeus obscurus|ABGYB394-06|ROM 103101|Guyana.Upper Takutu-Upper Essequibo
Artibeus obscurus|ABGYB392-06|ROM 103099|Guyana.Upper Takutu-Upper Essequibo
Artibeus obscurus|ABGYB390-06|ROM 103097|Guyana.Upper Takutu-Upper Essequibo
Artibeus obscurus|ABGYB386-06|ROM 103093|Guyana.Upper Takutu-Upper Essequibo
Artibeus obscurus|ABGYB361-06|ROM 103068|Guyana.Upper Takutu-Upper Essequibo
Artibeus obscurus|ABGYB325-06|ROM 103030|Guyana.Upper Takutu-Upper Essequibo
Artibeus obscurus|ABGYB324-06|ROM 103029|Guyana.Upper Takutu-Upper Essequibo
Artibeus obscurus|ABGYB396-06|ROM 103103|Guyana.Upper Takutu-Upper Essequibo
Artibeus obscurus|BCBNC108-06|ROM 115744|Guyana.Potaro-Siparuni
Artibeus obscurus|ABGYC340-06|ROM 107152|Guyana.Potaro-Siparuni
Artibeus obscurus|ABGYG1000-08|ROM 118924|Guyana
Artibeus obscurus|BCBNT523-06|ROM 111877|Guyana.Potaro-Siparuni
Artibeus obscurus|ABGYE777-06|ROM 113378|Guyana.Upper Demerara-Berbice
Artibeus obscurus|ABGYE778-06|ROM 113379|Guyana.Upper Demerara-Berbice
Artibeus obscurus|ABGYE082-06|ROM 111525|Guyana.Potaro-Siparuni
Artibeus obscurus|ABGYE267-06|ROM 111716|Guyana.Potaro-Siparuni
Artibeus obscurus|ABGYD277-06|ROM 108613|Guyana.Potaro-Siparuni
Artibeus obscurus|ABGYD585-06|ROM 109005|Guyana.Potaro-Siparuni
Artibeus obscurus|ABGYE010-06|ROM 109293|Guyana.Potaro-Siparuni
Artibeus obscurus|ABGYE070-06|ROM 111513|Guyana.Potaro-Siparuni
Artibeus obscurus|ABGYC488-06|ROM 107321|Guyana.Potaro-Siparuni
Artibeus obscurus|ABGYC263-06|ROM 107049|Guyana.Potaro-Siparuni
Artibeus obscurus|ABGYC012-06|ROM 104789|Guyana.Potaro-Siparuni
Artibeus obscurus|ABGYB917-06|ROM 104762|Guyana.Potaro-Siparuni
Artibeus obscurus|ABGYB874-06|ROM 104718|Guyana.Potaro-Siparuni
Artibeus obscurus|ABGYB872-06|ROM 104716|Guyana.Potaro-Siparuni
Artibeus obscurus|ABGYB688-06|ROM 103452|Guyana.Upper Demerara-Berbice

Artibeus obscurus|ABGYB874-06|ROM 104718|Guyana.Potaro-Siparuni
Artibeus obscurus|ABGYB872-06|ROM 104716|Guyana.Potaro-Siparuni
Artibeus obscurus|ABGYB688-06|ROM 103452|Guyana.Upper Demerara-Berbice
Artibeus obscurus|ABGYB653-06|ROM 103410|Guyana.Upper Demerara-Berbice
Artibeus obscurus|ABGYE271-06|ROM 111720|Guyana.Potaro-Siparuni
Artibeus obscurus|ABGYB887-06|ROM 104732|Guyana.Potaro-Siparuni
Artibeus obscurus|BCBNT086-06|ROM 107075|Guyana.Potaro-Siparuni
Artibeus obscurus|BCBNT526-06|ROM 111898|Guyana.Potaro-Siparuni
Artibeus obscurus|BCBNT536-06|ROM 111943|Guyana.Potaro-Siparuni
Artibeus obscurus|ABGYA700-06|ROM 100405|Guyana.East Berbice-Corentyne
Artibeus obscurus|BCBNT737-06|ROM 113912|Suriname.Brokopondo
Artibeus obscurus|BCBNT100-06|ROM 107110|Guyana.Potaro-Siparuni
Artibeus obscurus|BCBNT332-06|ROM 108906|Guyana.Potaro-Siparuni
Artibeus obscurus|BCBNT080-06|ROM 107051|Guyana.Potaro-Siparuni
Artibeus obscurus|BCBNT096-06|ROM 107096|Guyana.Potaro-Siparuni
Artibeus obscurus|BCBN321-05|ROM 100837|Guyana.Barima-Waini
Artibeus obscurus|BCBNT079-06|ROM 107050|Guyana.Potaro-Siparuni
Artibeus obscurus|ABSRA469-06|ROM 117059|Suriname.Nickerie
Artibeus obscurus|ABSRA479-06|ROM 117069|Suriname.Nickerie
Artibeus obscurus|ABGYF162-06|ROM 113718|Guyana.Demerara-Mahaica
Artibeus obscurus|ABGYG094-06|ROM 115637|Guyana.Essequibo Islands-West Demerara
Artibeus obscurus|ABGYC706-06|ROM 108105|Guyana.Cuyuni-Mazaruni
Artibeus obscurus|ABGYD056-06|ROM 108352|Guyana.Potaro-Siparuni
Artibeus obscurus|ABGYD187-06|ROM 108514|Guyana.Potaro-Siparuni
Artibeus obscurus|ABGYD210-06|ROM 108541|Guyana.Potaro-Siparuni
Artibeus obscurus|ABGYD247-06|ROM 108579|Guyana.Potaro-Siparuni
Artibeus obscurus|ABGYD248-06|ROM 108580|Guyana.Potaro-Siparuni
Artibeus obscurus|ABGYD279-06|ROM 108615|Guyana.Potaro-Siparuni
Artibeus obscurus|ABGYD280-06|ROM 108616|Guyana.Potaro-Siparuni
Artibeus obscurus|ABGYD299-06|ROM 108638|Guyana.Potaro-Siparuni
Artibeus obscurus|ABGYD309-06|ROM 108648|Guyana.Potaro-Siparuni
Artibeus obscurus|ABGYD359-06|ROM 108702|Guyana.Potaro-Siparuni
Artibeus obscurus|ABGYD468-06|ROM 108819|Guyana.Potaro-Siparuni
Artibeus obscurus|ABGYD572-06|ROM 108948|Guyana.Potaro-Siparuni
Artibeus obscurus|ABGYD586-06|ROM 109006|Guyana.Potaro-Siparuni
Artibeus obscurus|ABGYD588-06|ROM 109008|Guyana.Potaro-Siparuni
Artibeus obscurus|ABGYD702-06|ROM 109115|Guyana.Potaro-Siparuni
Artibeus obscurus|ABGYD725-06|ROM 109141|Guyana.Potaro-Siparuni
Artibeus obscurus|ABGYD739-06|ROM 109157|Guyana.Potaro-Siparuni
Artibeus obscurus|ABGYA542-06|ROM 100213|Guyana.East Berbice-Corentyne
Artibeus obscurus|ABGYA582-06|ROM 100267|Guyana.East Berbice-Corentyne
Artibeus obscurus|ABGYD755-06|ROM 109173|Guyana.Potaro-Siparuni
Artibeus obscurus|ABGYD776-06|ROM 109197|Guyana.Potaro-Siparuni
Artibeus obscurus|ABGYD780-06|ROM 109201|Guyana.Potaro-Siparuni
Artibeus obscurus|ABGYE006-06|ROM 109289|Guyana.Potaro-Siparuni
Artibeus obscurus|ABGYD554-06|ROM 108924|Guyana.Potaro-Siparuni
Artibeus obscurus|ABGYD570-06|ROM 108946|Guyana.Potaro-Siparuni
Artibeus obscurus|ABGYC462-06|ROM 107292|Guyana.Potaro-Siparuni
Artibeus obscurus|ABGYC497-06|ROM 107330|Guyana.Potaro-Siparuni
Artibeus obscurus|ABGYC561-06|ROM 107405|Guyana.Potaro-Siparuni
Artibeus obscurus|ABGYC653-06|ROM 107471|Guyana.Potaro-Siparuni
Artibeus obscurus|ABGYD079-06|ROM 108376|Guyana.Potaro-Siparuni
Artibeus obscurus|ABGYD110-06|ROM 108408|Guyana.Potaro-Siparuni
Artibeus obscurus|ABGYD117-06|ROM 108415|Guyana.Potaro-Siparuni
Artibeus obscurus|ABGYD146-06|ROM 108452|Guyana.Potaro-Siparuni
Artibeus obscurus|ABGYA583-06|ROM 100268|Guyana.East Berbice-Corentyne
Artibeus obscurus|ABGYA584-06|ROM 100269|Guyana.East Berbice-Corentyne
Artibeus obscurus|ABGYA603-06|ROM 100290|Guyana.East Berbice-Corentyne
Artibeus obscurus|ABGYA625-06|ROM 100319|Guyana.East Berbice-Corentyne
Artibeus obscurus|ABGYA626-06|ROM 100320|Guyana.East Berbice-Corentyne
Artibeus obscurus|ABGYA631-06|ROM 100325|Guyana.East Berbice-Corentyne
Artibeus obscurus|ABGYA632-06|ROM 100326|Guyana.East Berbice-Corentyne
Artibeus obscurus|ABGYA641-06|ROM 100336|Guyana.East Berbice-Corentyne
Artibeus obscurus|ABGYA698-06|ROM 100403|Guyana.East Berbice-Corentyne
Artibeus obscurus|ABGYA702-06|ROM 100407|Guyana.East Berbice-Corentyne
Artibeus obscurus|ABGYA711-06|ROM 100416|Guyana.East Berbice-Corentyne
Artibeus obscurus|ABGYA732-06|ROM 100437|Guyana.East Berbice-Corentyne
Artibeus obscurus|ABGYB165-06|ROM 101156|Guyana.Barima-Waini
Artibeus obscurus|ABGYB258-06|ROM 102962|Guyana.Upper Takutu-Upper Essequibo
Artibeus obscurus|ABGYB326-06|ROM 103031|Guyana.Upper Takutu-Upper Essequibo
Artibeus obscurus|ABGYB328-06|ROM 103033|Guyana.Upper Takutu-Upper Essequibo
Artibeus obscurus|ABGYB341-06|ROM 103048|Guyana.Upper Takutu-Upper Essequibo
Artibeus obscurus|ABGYB360-06|ROM 103067|Guyana.Upper Takutu-Upper Essequibo
Artibeus obscurus|ABGYB540-06|ROM 103275|Guyana.Upper Takutu-Upper Essequibo
Artibeus obscurus|ABGYB629-06|ROM 103384|Guyana.Upper Demerara-Berbice
Artibeus obscurus|ABGYB631-06|ROM 103386|Guyana.Upper Demerara-Berbice
Artibeus obscurus|ABGYB650-06|ROM 103407|Guyana.Upper Demerara-Berbice
Artibeus obscurus|ABGYB651-06|ROM 103408|Guyana.Upper Demerara-Berbice
Artibeus obscurus|ABGYB652-06|ROM 103409|Guyana.Upper Demerara-Berbice
Artibeus obscurus|ABGYB686-06|ROM 103450|Guyana.Upper Demerara-Berbice
Artibeus obscurus|ABGYB687-06|ROM 103451|Guyana.Upper Demerara-Berbice
Artibeus obscurus|ABGYB746-06|ROM 103513|Guyana.Upper Demerara-Berbice
Artibeus obscurus|ABGYB747-06|ROM 103514|Guyana.Upper Demerara-Berbice
Artibeus obscurus|ABGYB763-06|ROM 103531|Guyana.Upper Demerara-Berbice
Artibeus obscurus|ABGYB772-06|ROM 103540|Guyana.Upper Demerara-Berbice
Artibeus obscurus|ABGYB781-06|ROM 103549|Guyana.Upper Demerara-Berbice
Artibeus obscurus|ABGYB783-06|ROM 103551|Guyana.Upper Demerara-Berbice
Artibeus obscurus|ABGYB836-06|ROM 104677|Guyana.Potaro-Siparuni
Artibeus obscurus|ABGYB871-06|ROM 104715|Guyana.Potaro-Siparuni
Artibeus obscurus|ABGYB886-06|ROM 104731|Guyana.Potaro-Siparuni
Artibeus obscurus|ABGYB924-06|ROM 104769|Guyana.Potaro-Siparuni
Artibeus obscurus|ABGYC010-06|ROM 104787|Guyana.Potaro-Siparuni
Artibeus obscurus|ABGYC013-06|ROM 104790|Guyana.Potaro-Siparuni
Artibeus obscurus|ABGYC029-06|ROM 104806|Guyana.Potaro-Siparuni
Artibeus obscurus|ABGYC030-06|ROM 104807|Guyana.Potaro-Siparuni
Artibeus obscurus|ABGYC366-06|ROM 107182|Guyana.Potaro-Siparuni
Artibeus obscurus|ABGYC397-06|ROM 107216|Guyana.Potaro-Siparuni
Artibeus obscurus|ABGYC425-06|ROM 107247|Guyana.Potaro-Siparuni
Artibeus obscurus|ABGYC434-06|ROM 107258|Guyana.Potaro-Siparuni
Artibeus obscurus|ABGYC435-06|ROM 107259|Guyana.Potaro-Siparuni
Artibeus obscurus|ABGYC459-06|ROM 107289|Guyana.Potaro-Siparuni
Artibeus obscurus|ABGYC460-06|ROM 107290|Guyana.Potaro-Siparuni
Artibeus obscurus|ABGYC461-06|ROM 107291|Guyana.Potaro-Siparuni

[illegible]
Artibeus obscurus|ABGYC459-06|ROM 107289|Guyana.Potaro-Siparuni
Artibeus obscurus|ABGYC460-06|ROM 107290|Guyana.Potaro-Siparuni
Artibeus obscurus|ABGYC461-06|ROM 107291|Guyana.Potaro-Siparuni
Artibeus obscurus|ABGYC206-06|ROM 106745|Guyana.Upper Takutu-Upper Essequibo
Artibeus obscurus|ABGYC214-06|ROM 106755|Guyana.Upper Takutu-Upper Essequibo
Artibeus obscurus|ABGYC262-06|ROM 107048|Guyana.Potaro-Siparuni
Artibeus obscurus|ABGYC325-06|ROM 107137|Guyana.Potaro-Siparuni
Artibeus obscurus|ABGYC031-06|ROM 104808|Guyana.Potaro-Siparuni
Artibeus obscurus|ABGYC032-06|ROM 104809|Guyana.Potaro-Siparuni
Artibeus obscurus|ABGYC053-06|ROM 104830|Guyana.Potaro-Siparuni
Artibeus obscurus|ABGYC120-06|ROM 106632|Guyana.Upper Takutu-Upper Essequibo
Artibeus obscurus|ABGYA429-06|ROM 98837|Guyana.Barima-Waini
Artibeus obscurus|ABGYA472-06|ROM 98882|Guyana.Barima-Waini
Artibeus obscurus|ABGYA415-06|ROM 98823|Guyana.Barima-Waini
Artibeus obscurus|ABGYA427-06|ROM 98835|Guyana.Barima-Waini
Artibeus obscurus|ABGYE030-06|ROM 109315|Guyana.Potaro-Siparuni
Artibeus obscurus|ABGYE044-06|ROM 109330|Guyana.Potaro-Siparuni
Artibeus obscurus|ABGYE050-06|ROM 109336|Guyana.Potaro-Siparuni
Artibeus obscurus|ABGYE059-06|ROM 109345|Guyana.Potaro-Siparuni
Artibeus obscurus|ABGYE067-06|ROM 111510|Guyana.Potaro-Siparuni
Artibeus obscurus|ABGYE068-06|ROM 111511|Guyana.Potaro-Siparuni
Artibeus obscurus|ABGYE272-06|ROM 111721|Guyana.Potaro-Siparuni
Artibeus obscurus|ABGYE273-06|ROM 111722|Guyana.Potaro-Siparuni
Artibeus obscurus|ABGYE438-06|ROM 111899|Guyana.Potaro-Siparuni
Artibeus obscurus|ABGYE535-06|ROM 112008|Guyana.Potaro-Siparuni
Artibeus obscurus|ABGYE763-06|ROM 113362|Guyana.Upper Demerara-Berbice
Artibeus obscurus|ABGYE854-06|ROM 113470|Guyana.Upper Takutu-Upper Essequibo
Artibeus obscurus|ABGYE862-06|ROM 113478|Guyana.Upper Takutu-Upper Essequibo
Artibeus obscurus|ABGYF121-06|ROM 113667|Guyana.Demerara-Mahaica
Artibeus obscurus|ABGYG095-06|ROM 115641|Guyana.Potaro-Siparuni
Artibeus obscurus|ABGYG1012-08|ROM 118936|Guyana
Artibeus obscurus|ABGYG1014-08|ROM 118938|Guyana
Artibeus obscurus|ABGYG1022-08|ROM 118946|Guyana
Artibeus obscurus|ABGYG1045-08|ROM 118969|Guyana
Artibeus obscurus|ABGYG1083-08|ROM 119007|Guyana
Artibeus obscurus|ABGYG114-06|ROM 115664|Guyana.Potaro-Siparuni
Artibeus obscurus|ABGYG127-06|ROM 115681|Guyana.Potaro-Siparuni
Artibeus obscurus|ABGYG129-06|ROM 115683|Guyana.Potaro-Siparuni
Artibeus obscurus|ABGYG160-06|ROM 115720|Guyana.Potaro-Siparuni
Artibeus obscurus|ABSMS180-06|ROM 117342|Suriname.Sipaliwini
Artibeus obscurus|ABSMS238-06|ROM 117400|Suriname.Sipaliwini
Artibeus obscurus|ABSMS417-06|ROM 117579|Suriname.Sipaliwini
Artibeus obscurus|ABSMS526-06|ROM 117688|Suriname.Sipaliwini
Artibeus obscurus|ABSRA280-06|ROM 116869|Suriname.Nickerie
Artibeus obscurus|ABSRA394-06|ROM 116983|Suriname.Nickerie
Artibeus obscurus|ABSRA444-06|ROM 117034|Suriname.Nickerie
Artibeus obscurus|ABSRA454-06|ROM 117044|Suriname.Nickerie
Artibeus obscurus|ABSRA488-06|ROM 117078|Suriname.Nickerie
Artibeus obscurus|ABSRA501-06|ROM 117091|Suriname.Nickerie
Artibeus obscurus|BCBNT520-06|ROM 111867|Guyana.Potaro-Siparuni
Artibeus obscurus|BCBNT612-06|ROM 113516|Guyana.Upper Takutu-Upper Essequibo
Artibeus obscurus|BCBNT615-06|ROM 113528|Guyana.Upper Takutu-Upper Essequibo
Artibeus obscurus|BCBNT620-06|ROM 113535|Guyana.Upper Takutu-Upper Essequibo
Artibeus obscurus|BCBNT715-06|ROM 113884|Suriname.Brokopondo
Artibeus obscurus|BCBNT771-06|ROM 113990|Suriname.Brokopondo
Artibeus obscurus|ABGYC014-06|ROM 104791|Guyana.Potaro-Siparuni
Artibeus obscurus|ABGYC025-06|ROM 104802|Guyana.Potaro-Siparuni
Artibeus obscurus|ABGYB387-06|ROM 103094|Guyana.Upper Takutu-Upper Essequibo
Artibeus obscurus|ABGYB389-06|ROM 103096|Guyana.Upper Takutu-Upper Essequibo
Artibeus obscurus|ABGYA642-06|ROM 100337|Guyana.East Berbice-Corentyne
Artibeus obscurus|ABGYA651-06|ROM 100347|Guyana.East Berbice-Corentyne
Artibeus obscurus|BCBNT925-06|ROM 115607|Guyana.Essequibo Islands-West Demerara
Artibeus obscurus|ABGYA413-06|ROM 98821|Guyana.Barima-Waini
Artibeus obscurus|ABGYA410-06|ROM 98818|Guyana.Barima-Waini
Artibeus obscurus|ABGYA405-06|ROM 98813|Guyana.Barima-Waini
Artibeus obscurus|ABGYA400-06|ROM 98808|Guyana.Barima-Waini
Artibeus obscurus|ABGYA580-06|ROM 100265|Guyana.East Berbice-Corentyne
Artibeus obscurus|ABGYA604-06|ROM 100291|Guyana.East Berbice-Corentyne
Artibeus obscurus|ABGYA204-06|ROM 98072|Guyana.Potaro-Siparuni
Artibeus obscurus|ABGYA163-06|ROM 98022|Guyana.Potaro-Siparuni
Artibeus obscurus|ABGYA148-06|ROM 97993|Guyana.Potaro-Siparuni
Artibeus obscurus|ABGYC339-06|ROM 107151|Guyana.Potaro-Siparuni
Artibeus obscurus|ABGYB257-06|ROM 102961|Guyana.Upper Takutu-Upper Essequibo
Artibeus obscurus|ABGYD831-06|ROM 109255|Guyana.Potaro-Siparuni
Artibeus obscurus|ABGYE536-06|ROM 112009|Guyana.Potaro-Siparuni
Artibeus obscurus|ABGYB723-06|ROM 103489|Guyana.Upper Demerara-Berbice
Artibeus obscurus|ABGYA390-06|ROM 98794|Guyana.Barima-Waini
Artibeus obscurus|ABSMS442-06|ROM 117604|Suriname.Sipaliwini
Artibeus obscurus|ABGYB201-06|ROM 102903|Guyana.Upper Takutu-Upper Essequibo
Artibeus obscurus|ABGYD116-06|ROM 108414|Guyana.Potaro-Siparuni
Artibeus obscurus|ABGYD144-06|ROM 108450|Guyana.Potaro-Siparuni
Artibeus obscurus|ABGYD154-06|ROM 108461|Guyana.Potaro-Siparuni
Artibeus obscurus|ABGYD186-06|ROM 108513|Guyana.Potaro-Siparuni
Artibeus obscurus|ABGYD281-06|ROM 108617|Guyana.Potaro-Siparuni
Artibeus obscurus|ABGYA581-06|ROM 100266|Guyana.East Berbice-Corentyne
Artibeus obscurus|ABGYB033-06|ROM 101005|Guyana.Barima-Waini
Artibeus obscurus|ABGYA353-06|ROM 98753|Guyana.Barima-Waini
Artibeus obscurus|ABGYA354-06|ROM 98754|Guyana.Barima-Waini
Artibeus obscurus|ABGYA508-06|ROM 98919|Guyana.Barima-Waini
Artibeus obscurus|ABGYB393-06|ROM 103100|Guyana.Upper Takutu-Upper Essequibo
Artibeus obscurus|ABGYD832-06|ROM 109256|Guyana.Potaro-Siparuni
Artibeus obscurus|ABGYG1082-08|ROM 119006|Guyana
Artibeus obscurus|ABGYA699-06|ROM 100404|Guyana.East Berbice-Corentyne
Artibeus obscurus|BCBN342-05|ROM 100942|Guyana.Barima-Waini
Artibeus obscurus|BCBN343-05|ROM 100943|Guyana.Barima-Waini
Artibeus obscurus|ABGYD726-06|ROM 109142|Guyana.Potaro-Siparuni
Artibeus obscurus|ABGYE079-06|ROM 111522|Guyana.Potaro-Siparuni
Artibeus obscurus|ABGYD369-06|ROM 108712|Guyana.Potaro-Siparuni
Artibeus obscurus|ABGYD497-06|ROM 108855|Guyana.Potaro-Siparuni
Artibeus obscurus|ABGYD155-06|ROM 108462|Guyana.Potaro-Siparuni
Artibeus obscurus|ABGYD368-06|ROM 108711|Guyana.Potaro-Siparuni
Artibeus obscurus|ABGYE493-06|ROM 111965|Guyana.Potaro-Siparuni
[illegible]

Artibeus obscurus|ABGYD[illegible]-06|ROM [illegible]|Guyana.Potaro-Siparuni
Artibeus obscurus|ABGYD368-06|ROM 108711|Guyana.Potaro-Siparuni
Artibeus obscurus|ABGYE493-06|ROM 111965|Guyana.Potaro-Siparuni
Artibeus obscurus|ABGYE497-06|ROM 111969|Guyana.Potaro-Siparuni
Artibeus obscurus|ABGYD522-06|ROM 108880|Guyana.Potaro-Siparuni
Artibeus obscurus|ABGYE069-06|ROM 111512|Guyana.Potaro-Siparuni
Artibeus obscurus|ABGYB932-06|ROM 104777|Guyana.Potaro-Siparuni
Artibeus obscurus|ABGYB769-06|ROM 103537|Guyana.Upper Demerara-Berbice
Artibeus obscurus|ABGYA748-06|ROM 100454|Guyana.East Berbice-Corentyne
Artibeus obscurus|ABGYA627-06|ROM 100321|Guyana.East Berbice-Corentyne
Artibeus obscurus|ABGYA585-06|ROM 100270|Guyana.East Berbice-Corentyne
Artibeus obscurus|ABGYA578-06|ROM 100263|Guyana.East Berbice-Corentyne
Artibeus obscurus|ABGYA296-06|ROM 98215|Guyana.Demerara-Mahaica
Artibeus obscurus|ABGYA295-06|ROM 98214|Guyana.Demerara-Mahaica
Artibeus obscurus|ABGYA624-06|ROM 100318|Guyana.East Berbice-Corentyne
Artibeus obscurus|ABGYC027-06|ROM 104804|Guyana.Potaro-Siparuni
Artibeus obscurus|ABGYB271-06|ROM 102975|Guyana.Upper Takutu-Upper Essequibo
Artibeus obscurus|ABGYC054-06|ROM 104831|Guyana.Potaro-Siparuni
Artibeus obscurus|ABGYE004-06|ROM 109285|Guyana.Potaro-Siparuni
Artibeus obscurus|ABGYE802-06|ROM 113404|Guyana.Upper Demerara-Berbice
Artibeus obscurus|ABGYG1066-08|ROM 118990|Guyana
Artibeus obscurus|ABGYG113-06|ROM 115663|Guyana.Potaro-Siparuni
Artibeus obscurus|ABGYG159-06|ROM 115719|Guyana.Potaro-Siparuni
Artibeus obscurus|ABGYE495-06|ROM 111967|Guyana.Potaro-Siparuni
Artibeus obscurus|ABGYF444-06|ROM 115074|Guyana.Cuyuni-Mazaruni
Artibeus obscurus|ABGYD775-06|ROM 109196|Guyana.Potaro-Siparuni
Artibeus obscurus|ABGYB391-06|ROM 103098|Guyana.Upper Takutu-Upper Essequibo
Artibeus obscurus|ABGYA205-06|ROM 98073|Guyana.Potaro-Siparuni
Artibeus obscurus|ABGYA673-06|ROM 100377|Guyana.East Berbice-Corentyne
Artibeus obscurus|ABGYA733-06|ROM 100438|Guyana.East Berbice-Corentyne
Artibeus obscurus|ABGYC657-06|ROM 107477|Guyana.Potaro-Siparuni
Artibeus obscurus|ABGYA735-06|ROM 100440|Guyana.East Berbice-Corentyne
Artibeus obscurus|ABGYG149-06|ROM 115709|Guyana.Potaro-Siparuni
Artibeus obscurus|ABGYC485-06|ROM 107318|Guyana.Potaro-Siparuni
Artibeus obscurus|ABGYA164-06|ROM 98023|Guyana.Potaro-Siparuni
Artibeus obscurus|ABGYG115-06|ROM 115665|Guyana.Potaro-Siparuni
Artibeus obscurus|ABECA002-06|ROM 103980|Ecuador.Napo
Artibeus obscurus|ABECA179-06|ROM 104492|Ecuador.Napo
Artibeus obscurus|ABECA255-06|ROM 105124|Ecuador.Napo
Artibeus obscurus|ABECA256-06|ROM 105125|Ecuador.Napo
Artibeus obscurus|ABECA270-06|ROM 105138|Ecuador.Napo
Artibeus obscurus|ABECA278-06|ROM 105145|Ecuador.Napo
Artibeus obscurus|ABECA488-06|ROM 105511|Ecuador.Napo
Artibeus obscurus|ABECB079-08|ROM 118741|Ecuador
Artibeus obscurus|ABGYA734-06|ROM 100439|Guyana.East Berbice-Corentyne
Artibeus obscurus|ABGYE235-06|ROM 111683|Guyana.Potaro-Siparuni
Artibeus obscurus|ABGYF016-06|ROM 113506|Guyana.Upper Takutu-Upper Essequibo
Artibeus obscurus|ABGYB388-06|ROM 103095|Guyana.Upper Takutu-Upper Essequibo
Artibeus obscurus|ABSRA478-06|ROM 117068|Suriname.Nickerie
Artibeus obscurus|ABSRA512-06|ROM 117102|Suriname.Nickerie
Artibeus obscurus|ABGYD608-06|ROM 108974|Guyana.Potaro-Siparuni
Artibeus obscurus|ABGYG1001-08|ROM 118925|Guyana
Artibeus obscurus|ABGYF049-06|ROM 113558|Guyana.Upper Takutu-Upper Essequibo
Artibeus obscurus|ABSRA524-06|ROM 117114|Suriname.Nickerie
Artibeus obscurus|ABSMS375-06|ROM 117537|Suriname.Sipaliwini
Artibeus obscurus|ABGYC218-06|ROM 106760|Guyana.Upper Takutu-Upper Essequibo
Artibeus obscurus|ABGYF039-06|ROM 113546|Guyana.Upper Takutu-Upper Essequibo
Artibeus obscurus|ABGYG800-08|ROM 119228|Guyana
Artibeus obscurus|ABGYG801-08|ROM 119229|Guyana
Artibeus obscurus|ABGYG843-08|ROM 119271|Guyana
Artibeus obscurus|ABGYG844-08|ROM 119272|Guyana
Artibeus obscurus|ABGYG1065-08|ROM 118989|Guyana
Artibeus obscurus|ABRMM076-07|ROM F39664|Guyana.Potaro-Siparuni
Artibeus obscurus|ABGYA701-06|ROM 100406|Guyana.East Berbice-Corentyne
Artibeus obscurus|ABGYB752-06|ROM 103519|Guyana.Upper Demerara-Berbice
Artibeus obscurus|ABGYC026-06|ROM 104803|Guyana.Potaro-Siparuni
Artibeus obscurus|ABGYC367-06|ROM 107183|Guyana.Potaro-Siparuni
Artibeus obscurus|ABGYC647-06|ROM 107465|Guyana.Potaro-Siparuni
Artibeus obscurus|ABGYE065-06|ROM 111508|Guyana.Potaro-Siparuni
Artibeus obscurus|ABGYE081-06|ROM 111524|Guyana.Potaro-Siparuni
Artibeus obscurus|ABGYE842-06|ROM 113455|Guyana.Upper Takutu-Upper Essequibo
Artibeus obscurus|ABGYE850-06|ROM 113466|Guyana.Upper Takutu-Upper Essequibo
Artibeus obscurus|ABGYE852-06|ROM 113468|Guyana.Upper Takutu-Upper Essequibo
Artibeus obscurus|ABGYE863-06|ROM 113479|Guyana.Upper Takutu-Upper Essequibo
Artibeus obscurus|BCBNT626-06|ROM 113556|Guyana.Upper Takutu-Upper Essequibo
Artibeus obscurus|ABGYB774-06|ROM 103542|Guyana.Upper Demerara-Berbice
Artibeus obscurus|ABGYB773-06|ROM 103541|Guyana.Upper Demerara-Berbice
Artibeus obscurus|ABGYB689-06|ROM 103453|Guyana.Upper Demerara-Berbice
Artibeus obscurus|ABGYB685-06|ROM 103449|Guyana.Upper Demerara-Berbice
Artibeus obscurus|ABGYB630-06|ROM 103385|Guyana.Upper Demerara-Berbice
Artibeus obscurus|ABGYD278-06|ROM 108614|Guyana.Potaro-Siparuni
Artibeus obscurus|ABGYD373-06|ROM 108718|Guyana.Potaro-Siparuni
Artibeus obscurus|ABGYD740-06|ROM 109158|Guyana.Potaro-Siparuni
Artibeus obscurus|ABGYE257-06|ROM 111706|Guyana.Potaro-Siparuni
Artibeus obscurus|ABGYE788-06|ROM 113389|Guyana.Upper Demerara-Berbice
Artibeus obscurus|ABGYG1002-08|ROM 118926|Guyana
Artibeus obscurus|BCBNT352-06|ROM 109018|Guyana.Potaro-Siparuni
Artibeus obscurus|BCBNT908-06|ROM 115571|Guyana.Essequibo Islands-West Demerara
Artibeus obscurus|ABGYA389-06|ROM 98793|Guyana.Barima-Waini
Artibeus obscurus|BCBNT937-06|ROM 115636|Guyana.Essequibo Islands-West Demerara
Artibeus jamaicensis|BCBN620-05|ROM 104220|Panama.Canal Zone
Artibeus jamaicensis|ABSCA060-06|ROM 104252|Panama
Artibeus jamaicensis|BCBN639-05|ROM 104250|Panama
Artibeus jamaicensis|ABSCA122-06|ROM 104363|Panama.Darien
Artibeus jamaicensis|ABSCA121-06|ROM 104362|Panama.Darien
Artibeus jamaicensis|ABSCA105-06|ROM F38189|Panama.Darien
Artibeus jamaicensis|ABSCA036-06|ROM 104206|Panama.Canal Zone
Artibeus jamaicensis|BCBN640-05|ROM 104251|Panama
Artibeus jamaicensis|ABSCA100-06|ROM 104327|Panama.Chiriqui
Artibeus jamaicensis|BCBN684-05|ROM 104317|Panama.Chiriqui
Artibeus jamaicensis|BCBNT438-06|ROM 108316|Costa Rica.Limon
Artibeus jamaicensis|ABMXA782-06|ROM FN30470|Mexico.Yucatan
Artibeus jamaicensis|ABCSA479-06|ROM 99462|Guatemala.Peten

Artibeus jamaicensis|BCBNT438-06|ROM 108316|Costa Rica.Limon
Artibeus jamaicensis|ABMXA782-06|ROM FN30470|Mexico.Yucatan
Artibeus jamaicensis|ABCSA479-06|ROM 99462|Guatemala.Peten
Artibeus jamaicensis|ABMXA789-06|ROM FN30478|Mexico.Campeche
Artibeus jamaicensis|ABMXA794-06|ROM FN30483|Mexico.Campeche
Artibeus jamaicensis|ABMXC437-06|ROM 97380|Mexico.Quintana Roo
Artibeus jamaicensis|ABMXC508-06|ROM 97424|Mexico.Quintana Roo
Artibeus jamaicensis|ABMXC541-06|ROM 97469|Mexico.Quintana Roo
Artibeus jamaicensis|BCBN495-05|ROM 97482|Mexico.Quintana Roo
Artibeus jamaicensis|ABMXA795-06|ROM FN30484|Mexico.Campeche
Artibeus jamaicensis|ABMXC438-06|ROM 97381|Mexico.Quintana Roo
Artibeus jamaicensis|ABMXC504-06|ROM 97420|Mexico.Quintana Roo
Artibeus jamaicensis|BCBN497-05|ROM 97486|Mexico.Quintana Roo
Artibeus jamaicensis|ABMXC539-06|ROM 97467|Mexico.Quintana Roo
Artibeus jamaicensis|BCBN380-05|ROM 101234|El Salvador.Ahuachapan
Artibeus jamaicensis|ABCSA477-06|ROM 99460|Guatemala.Peten
Artibeus jamaicensis|ABCSA724-06|ROM 101298|El Salvador.Ahuachapan
Artibeus jamaicensis|ABCSA302-06|ROM 99278|Guatemala.Peten
Artibeus jamaicensis|BCBN188-05|ROM 99260|Guatemala.Peten
Artibeus jamaicensis|ABMXC256-06|ROM 96353|Mexico.Campeche
Artibeus jamaicensis|ABMXB733-06|ROM 95617|Mexico.Campeche
Artibeus jamaicensis|ABMXA788-06|ROM FN30477|Mexico.Campeche
Artibeus jamaicensis|ABCSA749-06|ROM 101346|El Salvador.Ahuachapan
Artibeus jamaicensis|ABCSA741-06|ROM 101326|El Salvador.Ahuachapan
Artibeus jamaicensis|ABCSA740-06|ROM 101325|El Salvador.Ahuachapan
Artibeus jamaicensis|ABCSA739-06|ROM 101324|El Salvador.Ahuachapan
Artibeus jamaicensis|ABCSA737-06|ROM 101322|El Salvador.Ahuachapan
Artibeus jamaicensis|ABCSA541-06|ROM 99555|Guatemala.Peten
Artibeus jamaicensis|ABCSA539-06|ROM 99551|Guatemala.Peten
Artibeus jamaicensis|ABCSA476-06|ROM 99459|Guatemala.Peten
Artibeus jamaicensis|ABCSA289-06|ROM 99264|Guatemala.Peten
Artibeus jamaicensis|ABMXA873-06|ROM FN30620|Mexico.Campeche
Artibeus jamaicensis|ABCSA223-06|ROM 98499|Guatemala.El Progreso
Artibeus jamaicensis|ABCSA288-06|ROM 99263|Guatemala.Peten
Artibeus jamaicensis|ABMXC538-06|ROM 97466|Mexico.Quintana Roo
Artibeus jamaicensis|BCBN189-05|ROM 99261|Guatemala.Peten
Artibeus jamaicensis|ABMXA792-06|ROM FN30481|Mexico.Campeche
Artibeus jamaicensis|BCBN403-05|ROM 101295|El Salvador.Ahuachapan
Artibeus jamaicensis|ABMXA875-06|ROM FN30622|Mexico.Campeche
Artibeus jamaicensis|ABCSA478-06|ROM 99461|Guatemala.Peten
Artibeus jamaicensis|BCBN405-05|ROM 101297|El Salvador.Ahuachapan
Artibeus jamaicensis|BCBN496-05|ROM 97483|Mexico.Quintana Roo
Artibeus jamaicensis|ABSCA142-06|ROM F44062|Costa Rica.Limon
Artibeus jamaicensis|BCBNT450-06|ROM 108328|Costa Rica.Limon
Artibeus jamaicensis|ABMXC255-06|ROM 96352|Mexico.Campeche
Artibeus jamaicensis|ABCSA348-06|ROM 99330|Guatemala.Peten
Artibeus jamaicensis|ABCSA540-06|ROM 99554|Guatemala.Peten
Artibeus jamaicensis|ABMXC257-06|ROM 96354|Mexico.Campeche
Artibeus jamaicensis|ABMXC258-06|ROM 96355|Mexico.Campeche
Artibeus jamaicensis|ABCSA222-06|ROM 98498|Guatemala.El Progreso
Artibeus jamaicensis|ABMXA793-06|ROM FN30482|Mexico.Campeche
Artibeus jamaicensis|ABCSA221-06|ROM 98497|Guatemala.El Progreso
Artibeus jamaicensis|ABMXC509-06|ROM 97425|Mexico.Quintana Roo
Artibeus jamaicensis|ABCSA303-06|ROM 99279|Guatemala.Peten
Artibeus jamaicensis|ABMXA868-06|ROM FN30615|Mexico.Campeche
Artibeus jamaicensis|ABMXA874-06|ROM FN30621|Mexico.Campeche
Artibeus jamaicensis|ABMXA864-06|ROM FN30611|Mexico.Campeche
Artibeus jamaicensis|ABMXA229-06|ROM ASK379|Mexico.Campeche
Artibeus jamaicensis|ABCSA304-06|ROM 99280|Guatemala.Peten
Artibeus jamaicensis|ABMXA817-06|ROM FN30527|Mexico.Campeche
Artibeus jamaicensis|ABMXC231-06|ROM 96299|Mexico.Campeche
Artibeus jamaicensis|ABMXC259-06|ROM 96356|Mexico.Campeche
Artibeus jamaicensis|ABMXC501-06|ROM 97417|Mexico.Quintana Roo
Artibeus jamaicensis|ABMXC537-06|ROM 97465|Mexico.Quintana Roo
Artibeus jamaicensis|ABMXC540-06|ROM 97468|Mexico.Quintana Roo
Artibeus jamaicensis|BCBN190-05|ROM 99262|Guatemala.Peten
Artibeus jamaicensis|BCBN256-05|ROM 99553|Guatemala.Peten
Artibeus jamaicensis|ABCSA220-06|ROM FN31485|Guatemala.El Progreso
Artibeus jamaicensis|ABCSA154-06|ROM 98401|Guatemala.El Progreso
Artibeus jamaicensis|ABCSA738-06|ROM 101323|El Salvador.Ahuachapan
Artibeus jamaicensis|BCBN428-05|ROM 101345|El Salvador.Ahuachapan
Artibeus jamaicensis|BCBN255-05|ROM 99552|Guatemala.Peten
Artibeus jamaicensis|ABMXA969-06|ROM FN32775|Mexico.Campeche
Artibeus jamaicensis|ABMXA872-06|ROM FN30619|Mexico.Campeche
Artibeus jamaicensis|ABMXA870-06|ROM FN30617|Mexico.Campeche
Artibeus jamaicensis|ABMXA869-06|ROM FN30616|Mexico.Campeche
Artibeus jamaicensis|ABCSA475-06|ROM 99458|Guatemala.Peten
Artibeus jamaicensis|ABCSA373-06|ROM 99385|Guatemala.Peten
Artibeus jamaicensis|ABCSA480-06|ROM 99463|Guatemala.Peten
Artibeus jamaicensis|BCBN494-05|ROM 97481|Mexico.Quintana Roo
Artibeus jamaicensis|BCBNT451-06|ROM 108329|Costa Rica.Limon
Artibeus fimbriatus|BCBNT504-06|ROM 111152|Brazil.Sao Paulo
Artibeus fimbriatus|BCBNT480-06|ROM 111097|Brazil.Sao Paulo
Artibeus fimbriatus|BCBNT507-06|ROM 111160|Brazil.Sao Paulo
Artibeus concolor|ABGYB539-06|ROM 103274|Guyana.Upper Takutu-Upper Essequibo
Artibeus concolor|ABGYG577-06|ROM 116739|Guyana.Potaro-Siparuni
Artibeus concolor|ABGYG379-06|ROM 116502|Guyana.Potaro-Siparuni
Artibeus concolor|ABGYC094-06|ROM 106600|Guyana.Upper Takutu-Upper Essequibo
Artibeus concolor|ABGYE703-06|ROM 112637|Guyana.Demerara-Mahaica
Artibeus concolor|ABGYG452-06|ROM 116589|Guyana.Potaro-Siparuni
Artibeus concolor|ABGYC134-06|ROM 106646|Guyana.Upper Takutu-Upper Essequibo
Artibeus concolor|ABGYB538-06|ROM 103273|Guyana.Upper Takutu-Upper Essequibo
Artibeus concolor|ABGYB724-06|ROM 103490|Guyana.Upper Demerara-Berbice
Artibeus concolor|ABGYC170-06|ROM 106699|Guyana.Upper Takutu-Upper Essequibo
Artibeus concolor|ABGYC586-06|ROM 107441|Guyana.Potaro-Siparuni
Artibeus concolor|ABGYA438-06|ROM 98846|Guyana.Barima-Waini
Artibeus concolor|ABGYC378-06|ROM 107195|Guyana.Potaro-Siparuni
Artibeus concolor|ABGYB499-06|ROM 103234|Guyana.Upper Takutu-Upper Essequibo
Artibeus concolor|ABGYB273-06|ROM 102977|Guyana.Upper Takutu-Upper Essequibo
Artibeus concolor|ABGYB592-06|ROM 103333|Guyana.Upper Takutu-Upper Essequibo
Artibeus concolor|ABGYB821-06|ROM 104662|Guyana.Potaro-Siparuni
Artibeus concolor|ABRMM083-07|ROM F39705|Guyana.Potaro-Siparuni
Artibeus concolor|ABGYB555-06|ROM 103291|Guyana.Upper Takutu-Upper Essequibo

Artibeus concolor|ABGYB821-06|ROM 104662|Guyana.Potaro-Siparuni
Artibeus concolor|ABRMM083-07|ROM F39705|Guyana.Potaro-Siparuni
Artibeus concolor|ABGYB555-06|ROM 103291|Guyana.Upper Takutu-Upper Essequibo
Artibeus concolor|ABGYB556-06|ROM 103292|Guyana.Upper Takutu-Upper Essequibo
Artibeus concolor|BCBNT125-06|ROM 107335|Guyana.Potaro-Siparuni
Artibeus concolor|ABGYD495-06|ROM 108853|Guyana.Potaro-Siparuni
Artibeus concolor|ABGYB830-06|ROM 104671|Guyana.Potaro-Siparuni
Artibeus concolor|ABGYC573-06|ROM 107421|Guyana.Potaro-Siparuni
Artibeus concolor|ABGYA614-06|ROM 100302|Guyana.East Berbice-Corentyne
Artibeus concolor|ABGYB195-06|ROM 102897|Guyana.Upper Takutu-Upper Essequibo
Artibeus concolor|ABGYB308-06|ROM 103013|Guyana.Upper Takutu-Upper Essequibo
Artibeus concolor|ABGYB500-06|ROM 103235|Guyana.Upper Takutu-Upper Essequibo
Artibeus concolor|ABGYB750-06|ROM 103517|Guyana.Upper Demerara-Berbice
Artibeus concolor|ABGYF221-06|ROM 113792|Guyana.Demerara-Mahaica
Artibeus concolor|BCBNT135-06|ROM 107408|Guyana.Potaro-Siparuni
Artibeus concolor|ABGYB498-06|ROM 103233|Guyana.Upper Takutu-Upper Essequibo
Artibeus concolor|ABSMS337-06|ROM 117499|Suriname.Sipaliwini
Artibeus concolor|BCBNT279-06|ROM 108460|Guyana.Potaro-Siparuni
Artibeus concolor|ABGYB501-06|ROM 103236|Guyana.Upper Takutu-Upper Essequibo
Artibeus concolor|ABGYG471-06|ROM 116609|Guyana.Potaro-Siparuni
Artibeus concolor|ABGYC660-06|ROM 107480|Guyana.Potaro-Siparuni
Artibeus concolor|ABGYE390-06|ROM 111842|Guyana.Potaro-Siparuni
Artibeus concolor|ABGYB649-06|ROM 103406|Guyana.Upper Demerara-Berbice
Artibeus concolor|ABGYC323-06|ROM 107135|Guyana.Potaro-Siparuni
Artibeus concolor|BCBNT179-06|ROM 107867|Venezuela.Amazonas
Artibeus concolor|BCBNT530-06|ROM 111925|Guyana.Potaro-Siparuni
Artibeus concolor|ABGYB535-06|ROM 103270|Guyana.Upper Takutu-Upper Essequibo
Artibeus concolor|ABGYB536-06|ROM 103271|Guyana.Upper Takutu-Upper Essequibo
Artibeus concolor|ABGYE681-06|ROM 112609|Guyana.Demerara-Mahaica
Artibeus concolor|ABGYC071-06|ROM 106568|Guyana.Upper Takutu-Upper Essequibo
Artibeus concolor|ABGYB502-06|ROM 103237|Guyana.Upper Takutu-Upper Essequibo
Artibeus concolor|ABGYB503-06|ROM 103238|Guyana.Upper Takutu-Upper Essequibo
Artibeus concolor|ABGYB664-06|ROM 103424|Guyana.Upper Demerara-Berbice
Artibeus concolor|ABGYC095-06|ROM 106601|Guyana.Upper Takutu-Upper Essequibo
Artibeus concolor|BCBN544-05|ROM 103392|Guyana.Upper Demerara-Berbice
Artibeus concolor|ABGYB196-06|ROM 102898|Guyana.Upper Takutu-Upper Essequibo
Artibeus concolor|ABGYC315-06|ROM 107127|Guyana.Potaro-Siparuni
Artibeus concolor|BCBNT122-06|ROM 107298|Guyana.Potaro-Siparuni
Artibeus concolor|ABGYC093-06|ROM 106599|Guyana.Upper Takutu-Upper Essequibo
Artibeus concolor|ABGYB907-06|ROM 104752|Guyana.Potaro-Siparuni
Artibeus concolor|ABGYG789-08|ROM 119217|Guyana
Artibeus concolor|BCBNT754-06|ROM 113949|Suriname.Brokopondo
Artibeus concolor|BCBNT782-06|ROM 114018|Suriname.Brokopondo
Artibeus concolor|ABGYA437-06|ROM 98845|Guyana.Barima-Waini
Artibeus concolor|ABGYG846-08|ROM 119274|Guyana
Artibeus concolor|ABGYE670-06|ROM 112592|Guyana.Demerara-Mahaica
Artibeus concolor|BCBNT116-06|ROM 107269|Guyana.Potaro-Siparuni
Artibeus concolor|ABGYD489-06|ROM 108842|Guyana.Potaro-Siparuni
Artibeus concolor|ABGYB537-06|ROM 103272|Guyana.Upper Takutu-Upper Essequibo
Artibeus concolor|ABGYG380-06|ROM 116503|Guyana.Potaro-Siparuni
Artibeus concolor|BCBNT136-06|ROM 107413|Guyana.Potaro-Siparuni
Artibeus concolor|BCBNT401-06|ROM 109316|Guyana.Potaro-Siparuni
Artibeus concolor|BCBNT365-06|ROM 108999|Guyana.Potaro-Siparuni
Artibeus concolor|ABSRA346-06|ROM 116935|Suriname.Nickerie
Artibeus concolor|ABGYE704-06|ROM 112638|Guyana.Demerara-Mahaica
Artibeus concolor|ABGYD751-06|ROM 109169|Guyana.Potaro-Siparuni
Artibeus concolor|ABGYD519-06|ROM 108877|Guyana.Potaro-Siparuni
Artibeus concolor|ABGYC407-06|ROM 107226|Guyana.Potaro-Siparuni
Artibeus concolor|ABGYB591-06|ROM 103332|Guyana.Upper Takutu-Upper Essequibo
Artibeus concolor|BCBNT798-06|ROM 114069|Suriname.Brokopondo
Artibeus concolor|ABGYB754-06|ROM 103522|Guyana.Upper Demerara-Berbice
Artibeus concolor|ABGYB749-06|ROM 103516|Guyana.Upper Demerara-Berbice
Artibeus concolor|ABGYC667-06|ROM 107487|Guyana.Potaro-Siparuni
Artibeus concolor|ABGYG472-06|ROM 116610|Guyana.Potaro-Siparuni
Artibeus concolor|ABGYC568-06|ROM 107414|Guyana.Potaro-Siparuni
Artibeus concolor|ABGYG835-08|ROM 119263|Guyana
Artibeus concolor|ABGYB497-06|ROM 103232|Guyana.Upper Takutu-Upper Essequibo
Artibeus concolor|ABGYG552-06|ROM 116698|Guyana.Potaro-Siparuni
Artibeus concolor|BCBNT907-06|ROM 115569|Guyana.Essequibo Islands-West Demerara
Artibeus watsoni|BCBN927-05|ROM 105802|Ecuador.Esmeraldas
Artibeus watsoni|BCBN911-05|ROM 105783|Ecuador.Esmeraldas
Artibeus watsoni|ABECA717-06|ROM 105799|Ecuador.Esmeraldas
Artibeus watsoni|ABECA719-06|ROM 105805|Ecuador.Esmeraldas
Artibeus watsoni|ABECA718-06|ROM 105804|Ecuador.Esmeraldas
Artibeus watsoni|BCBN922-05|ROM 105796|Ecuador.Esmeraldas
Artibeus watsoni|BCBN923-05|ROM 105797|Ecuador.Esmeraldas
Artibeus watsoni|ABECA721-06|ROM 105817|Ecuador.Esmeraldas
Artibeus watsoni|BCBN928-05|ROM 105803|Ecuador.Esmeraldas
Artibeus watsoni|ABSCA093-06|ROM 104319|Panama.Chiriqui
Artibeus watsoni|BCBN685-05|ROM 104318|Panama.Chiriqui
Artibeus watsoni|BCBN686-05|ROM 104320|Panama.Chiriqui
Artibeus watsoni|ABSCA069-06|ROM F38109|Panama
Artibeus watsoni|BCBN650-05|ROM 104267|Panama
Artibeus watsoni|BCBN635-05|ROM 104245|Panama
Artibeus watsoni|BCBN659-05|ROM 104278|Panama
Artibeus watsoni|BCBNT409-06|ROM 108289|Costa Rica.Limon
Artibeus watsoni|ABSCA132-06|ROM F44014|Costa Rica.Limon
Artibeus watsoni|BCBN208-05|ROM 99427|Guatemala.Peten
Artibeus watsoni|BCBNT439-06|ROM 108317|Costa Rica.Limon
Artibeus watsoni|BCBNT428-06|ROM 108308|Costa Rica.Limon
Artibeus watsoni|BCBNT427-06|ROM 108307|Costa Rica.Limon
Artibeus watsoni|BCBNT410-06|ROM 108290|Costa Rica.Limon
Artibeus watsoni|BCBNT426-06|ROM 108306|Costa Rica.Limon
Artibeus watsoni|BCBNT445-06|ROM 108323|Costa Rica.Limon
Artibeus phaeotis|ABCSA499-06|ROM 99499|Guatemala.Peten
Artibeus phaeotis|ABMXC503-06|ROM 97419|Mexico.Quintana Roo
Artibeus phaeotis|BCBN378-05|ROM 101232|El Salvador.Ahuachapan
Artibeus phaeotis|ABCSA448-06|ROM 99593|Guatemala.Peten
Artibeus phaeotis|ABCSA408-06|ROM 99428|Guatemala.Peten
Artibeus phaeotis|ABMXC510-06|ROM 97426|Mexico.Quintana Roo
Artibeus phaeotis|ABMXC530-06|ROM 97455|Mexico.Quintana Roo
Artibeus phaeotis|ABMXC528-06|ROM 97453|Mexico.Quintana Roo
Artibeus phaeotis|ABMXC492-06|ROM 97404|Mexico.Quintana Roo

[illegible]
Artibeus phaeotis|ABMXC530-06|ROM 97455|Mexico.Quintana Roo
Artibeus phaeotis|ABMXC528-06|ROM 97453|Mexico.Quintana Roo
Artibeus phaeotis|ABMXC492-06|ROM 97404|Mexico.Quintana Roo
Artibeus phaeotis|ABMXA926-06|ROM FN33835|Mexico.Quintana Roo
Artibeus phaeotis|ABMXA799-06|ROM FN30488|Mexico.Campeche
Artibeus phaeotis|ABCSA372-06|ROM 99384|Guatemala.Peten
Artibeus phaeotis|ABMXA759-06|ROM FN30434|Mexico.Yucatan
Artibeus phaeotis|ABMXC531-06|ROM 97456|Mexico.Quintana Roo
Artibeus phaeotis|BCBN193-05|ROM 99282|Guatemala.Peten
Artibeus phaeotis|BCBN200-05|ROM 99382|Guatemala.Peten
Artibeus phaeotis|BCBN379-05|ROM 101233|El Salvador.Ahuachapan
Artibeus phaeotis|BCBN475-05|ROM 97399|Mexico.Quintana Roo
Artibeus phaeotis|ABCSA349-06|ROM 99332|Guatemala.Peten
Artibeus phaeotis|ABCSA483-06|ROM 99466|Guatemala.Peten
Artibeus phaeotis|ABMXA797-06|ROM FN30486|Mexico.Campeche
Artibeus phaeotis|BCBN192-05|ROM 99281|Guatemala.Peten
Artibeus phaeotis|ABCSA450-06|ROM 99595|Guatemala.Peten
Artibeus phaeotis|ABMXC511-06|ROM 97427|Mexico.Quintana Roo
Artibeus phaeotis|ABMXC529-06|ROM 97454|Mexico.Quintana Roo
Artibeus phaeotis|ABCSA503-06|ROM 99503|Guatemala.Peten
Artibeus phaeotis|ABMXA798-06|ROM FN30487|Mexico.Campeche
Artibeus phaeotis|ABMXA800-06|ROM FN30489|Mexico.Campeche
Artibeus phaeotis|ABMXC494-06|ROM 97406|Mexico.Quintana Roo
Artibeus phaeotis|ABMXC496-06|ROM 97408|Mexico.Quintana Roo
Artibeus phaeotis|ABMXC512-06|ROM 97428|Mexico.Quintana Roo
Artibeus phaeotis|ABMXC532-06|ROM 97457|Mexico.Quintana Roo
Artibeus phaeotis|ABMXC533-06|ROM 97458|Mexico.Quintana Roo
Artibeus phaeotis|ABMXC534-06|ROM 97459|Mexico.Quintana Roo
Artibeus phaeotis|ABMXC535-06|ROM 97460|Mexico.Quintana Roo
Artibeus phaeotis|BCBN191-05|ROM 99265|Guatemala.Peten
Artibeus phaeotis|ABMXC497-06|ROM 97409|Mexico.Quintana Roo
Artibeus phaeotis|ABMXC500-06|ROM 97416|Mexico.Quintana Roo
Artibeus phaeotis|ABMXC450-06|ROM 96938|Mexico.Quintana Roo
Artibeus phaeotis|ABMXC493-06|ROM 97405|Mexico.Quintana Roo
Artibeus phaeotis|BCBN198-05|ROM 99331|Guatemala.Peten
Artibeus phaeotis|BCBN199-05|ROM 99381|Guatemala.Peten
Artibeus phaeotis|ABMXA796-06|ROM FN30485|Mexico.Campeche
Artibeus phaeotis|ABMXA761-06|ROM FN30436|Mexico.Yucatan
Artibeus phaeotis|ABCSA502-06|ROM 99502|Guatemala.Peten
Artibeus phaeotis|ABCSA501-06|ROM 99501|Guatemala.Peten
Artibeus phaeotis|ABCSA500-06|ROM 99500|Guatemala.Peten
Artibeus phaeotis|ABCSA449-06|ROM 99594|Guatemala.Peten
Artibeus phaeotis|ABCSA447-06|ROM 99592|Guatemala.Peten
Artibeus phaeotis|ABCSA446-06|ROM 99591|Guatemala.Peten
Artibeus phaeotis|ABCSA409-06|ROM 99429|Guatemala.Peten
Artibeus phaeotis|ABCSA371-06|ROM 99383|Guatemala.Peten
Artibeus phaeotis|ABCSA350-06|ROM 99333|Guatemala.Peten
Artibeus phaeotis|ABCSA305-06|ROM 99283|Guatemala.Peten
Artibeus phaeotis|ABMXC495-06|ROM 97407|Mexico.Quintana Roo
Artibeus phaeotis|BCBN474-05|ROM 97398|Mexico.Quintana Roo
Artibeus phaeotis|ABMXC491-06|ROM 97403|Mexico.Quintana Roo
Artibeus phaeotis|BCBN476-05|ROM 97400|Mexico.Quintana Roo
Artibeus phaeotis|BCBN477-05|ROM 97401|Mexico.Quintana Roo
Artibeus phaeotis|BCBN478-05|ROM 97402|Mexico.Quintana Roo
Artibeus toltecus|ABCSA685-06|ROM 101228|El Salvador.Ahuachapan
Artibeus toltecus|ABSCA079-06|ROM F38137|Panama.Chiriqui
Artibeus toltecus|ABCSA696-06|ROM 101249|El Salvador.Ahuachapan
Artibeus toltecus|ABCSA697-06|ROM 101250|El Salvador.Ahuachapan
Artibeus toltecus|BCBN382-05|ROM 101236|El Salvador.Ahuachapan
Artibeus toltecus|ABCSA838-06|ROM 101458|El Salvador.Santa Ana
Artibeus toltecus|BCBN089-05|ROM 98465|Guatemala.Alta Verapaz
Artibeus toltecus|ABSCA080-06|ROM 104288|Panama.Chiriqui
Artibeus toltecus|BCBN638-05|ROM 104249|Panama
Artibeus toltecus|BCBN637-05|ROM 104248|Panama
Artibeus toltecus|BCBN667-05|ROM 104289|Panama.Chiriqui
Artibeus toltecus|BCBN515-05|ROM 97629|Mexico.Chiapas
Artibeus toltecus|BCBN636-05|ROM 104246|Panama
Artibeus toltecus|BCBN385-05|ROM 101247|El Salvador.Ahuachapan
Artibeus toltecus|BCBN426-05|ROM 101341|El Salvador.Ahuachapan
Artibeus toltecus|BCBN375-05|ROM 101227|El Salvador.Ahuachapan
Artibeus toltecus|BCBN277-05|ROM 99838|Guatemala.Zacapa
Artibeus toltecus|ABSCA059-06|ROM 104247|Panama
Artibeus toltecus|ABSCA058-06|ROM F38085|Panama
Artibeus toltecus|ABCSA837-06|ROM 101457|El Salvador.Santa Ana
Artibeus toltecus|ABCSA695-06|ROM 101248|El Salvador.Ahuachapan
Artibeus toltecus|ABCSA694-06|ROM 101246|El Salvador.Ahuachapan
Artibeus toltecus|ABCSA693-06|ROM 101244|El Salvador.Ahuachapan
Artibeus toltecus|ABCSA692-06|ROM 101243|El Salvador.Ahuachapan
Artibeus toltecus|ABCSA691-06|ROM 101242|El Salvador.Ahuachapan
Artibeus toltecus|BCBN417-05|ROM 101327|El Salvador.Ahuachapan
Artibeus toltecus|BCBN687-05|ROM 104321|Panama.Chiriqui
Artibeus aztecus|BCBN441-05|ROM 101407|El Salvador.Santa Ana
Artibeus aztecus|BCBN440-05|ROM 101406|El Salvador.Santa Ana
Artibeus aztecus|BCBN443-05|ROM 101423|El Salvador.Santa Ana
Artibeus aztecus|BCBN448-05|ROM 101428|El Salvador.Santa Ana
Artibeus aztecus|BCBN449-05|ROM 101429|El Salvador.Santa Ana
Artibeus aztecus|ABCSA797-06|ROM 101405|El Salvador.Santa Ana
Artibeus aztecus|BCBN451-05|ROM 101435|El Salvador.Santa Ana
Artibeus aztecus|BCBN436-05|ROM 101365|El Salvador.Santa Ana
Artibeus aztecus|BCBN439-05|ROM 101404|El Salvador.Santa Ana
Artibeus aztecus|BCBN435-05|ROM 101364|El Salvador.Santa Ana
Artibeus aztecus|BCBN462-05|ROM 101475|El Salvador.Santa Ana
Artibeus anderseni|ABRMM096-07|ROM F41828|Ecuador
Artibeus anderseni|ABECA053-06|ROM 104037|Ecuador.Napo
Artibeus anderseni|BCBN823-05|ROM 105341|Ecuador.Napo
Artibeus anderseni|BCBN863-05|ROM 105641|Ecuador.Napo
Artibeus anderseni|BCBN765-05|ROM 104543|Ecuador.Napo
Artibeus anderseni|BCBN567-05|ROM 104007|Ecuador.Napo
Artibeus anderseni|BCBN566-05|ROM 104006|Ecuador.Napo
Artibeus anderseni|ABECA085-06|ROM F37193|Ecuador.Napo
Artibeus anderseni|BCBN785-05|ROM 105176|Ecuador.Napo
Artibeus anderseni|ABECA638-06|ROM 105691|Ecuador.Napo
[illegible]

Artibeus anderseni|ABECA[illegible]-06|ROM [illegible]|Ecuador.Napo
Artibeus anderseni|BCBN785-05|ROM 105176|Ecuador.Napo
Artibeus anderseni|ABECA638-06|ROM 105691|Ecuador.Napo
Artibeus anderseni|BCBN825-05|ROM 105351|Ecuador.Napo
Artibeus anderseni|BCBN847-05|ROM 105533|Ecuador.Napo
Artibeus anderseni|BCBN866-05|ROM 105654|Ecuador.Napo
Artibeus anderseni|BCBN892-05|ROM 105717|Ecuador.Napo
Artibeus cinereus|ABGYA440-06|ROM 98848|Guyana.Barima-Waini
Artibeus cinereus|ABGYA541-06|ROM 100212|Guyana.East Berbice-Corentyne
Artibeus cinereus|ABGYA072-06|ROM 97894|Guyana.Upper Takutu-Upper Essequibo
Artibeus cinereus|ABGYE678-06|ROM 112606|Guyana.Demerara-Mahaica
Artibeus cinereus|ABGYA536-06|ROM 100206|Guyana.East Berbice-Corentyne
Artibeus cinereus|ABGYB261-06|ROM 102965|Guyana.Upper Takutu-Upper Essequibo
Artibeus cinereus|ABGYF203-06|ROM 113773|Guyana.Demerara-Mahaica
Artibeus cinereus|ABGYG889-08|ROM 119317|Guyana
Artibeus cinereus|ABGYA620-06|ROM 100312|Guyana.East Berbice-Corentyne
Artibeus cinereus|ABGYE701-06|ROM 112635|Guyana.Demerara-Mahaica
Artibeus cinereus|ABGYE702-06|ROM 112636|Guyana.Demerara-Mahaica
Artibeus cinereus|ABGYG782-08|ROM 119210|Guyana
Artibeus cinereus|ABGYF176-06|ROM 113732|Guyana.Demerara-Mahaica
Artibeus cinereus|ABGYB315-06|ROM 103020|Guyana.Upper Takutu-Upper Essequibo
Artibeus cinereus|ABGYB295-06|ROM 102999|Guyana.Upper Takutu-Upper Essequibo
Artibeus cinereus|ABGYB513-06|ROM 103248|Guyana.Upper Takutu-Upper Essequibo
Artibeus cinereus|ABGYB559-06|ROM 103295|Guyana.Upper Takutu-Upper Essequibo
Artibeus cinereus|ABGYF139-06|ROM 113690|Guyana.Demerara-Mahaica
Artibeus cinereus|ABGYB401-06|ROM 103108|Guyana.Upper Takutu-Upper Essequibo
Artibeus cinereus|ABGYB442-06|ROM 103156|Guyana.Upper Takutu-Upper Essequibo
Artibeus cinereus|ABGYA078-06|ROM 97901|Guyana.Upper Takutu-Upper Essequibo
Artibeus cinereus|ABGYB262-06|ROM 102966|Guyana.Upper Takutu-Upper Essequibo
Artibeus cinereus|ABGYG727-08|ROM 119155|Guyana
Artibeus cinereus|BCBNT687-06|ROM 113796|Guyana.Demerara-Mahaica
Artibeus cinereus|ABGYG780-08|ROM 119208|Guyana
Artibeus cinereus|ABGYG988-08|ROM 119416|Guyana
Artibeus cinereus|ABGYA397-06|ROM 98805|Guyana.Barima-Waini
Artibeus cinereus|ABGYA439-06|ROM 98847|Guyana.Barima-Waini
Artibeus cinereus|ABGYE717-06|ROM 112652|Guyana.Demerara-Mahaica
Artibeus cinereus|BCBNT695-06|ROM 113819|Guyana.Demerara-Mahaica
Artibeus cinereus|ABGYF142-06|ROM 113693|Guyana.Demerara-Mahaica
Artibeus cinereus|ABGYF223-06|ROM 113794|Guyana.Demerara-Mahaica
Artibeus cinereus|BCBN118-05|ROM 97849|Guyana.Upper Takutu-Upper Essequibo
Artibeus cinereus|ABGYE730-06|ROM 112670|Guyana.Demerara-Mahaica
Artibeus cinereus|ABGYB200-06|ROM 102902|Guyana.Upper Takutu-Upper Essequibo
Artibeus cinereus|ABGYF229-06|ROM 113804|Guyana.Demerara-Mahaica
Artibeus cinereus|ABGYA556-06|ROM 100231|Guyana.East Berbice-Corentyne
Artibeus cinereus|ABGYA425-06|ROM 98833|Guyana.Barima-Waini
Artibeus cinereus|BCBNT700-06|ROM 113833|Guyana.Demerara-Mahaica
Artibeus cinereus|BCBN168-05|ROM 98803|Guyana.Barima-Waini
Artibeus cinereus|ABGYG954-08|ROM 119382|Guyana
Artibeus cinereus|ABGYG952-08|ROM 119380|Guyana
Artibeus cinereus|ABGYG865-08|ROM 119293|Guyana
Artibeus cinereus|ABGYG828-08|ROM 119256|Guyana
Artibeus cinereus|ABGYG781-08|ROM 119209|Guyana
Artibeus cinereus|ABGYG708-08|ROM 119136|Guyana
Artibeus cinereus|ABGYF224-06|ROM 113795|Guyana.Demerara-Mahaica
Artibeus cinereus|ABGYF217-06|ROM 113788|Guyana.Demerara-Mahaica
Artibeus cinereus|ABGYF201-06|ROM 113771|Guyana.Demerara-Mahaica
Artibeus cinereus|ABGYF177-06|ROM 113734|Guyana.Demerara-Mahaica
Artibeus cinereus|ABGYF175-06|ROM 113731|Guyana.Demerara-Mahaica
Artibeus cinereus|ABGYF174-06|ROM 113733|Guyana.Demerara-Mahaica
Artibeus cinereus|ABGYF173-06|ROM 113730|Guyana.Demerara-Mahaica
Artibeus cinereus|ABGYF141-06|ROM 113692|Guyana.Demerara-Mahaica
Artibeus cinereus|ABGYF140-06|ROM 113691|Guyana.Demerara-Mahaica
Artibeus cinereus|ABGYF114-06|ROM 113653|Guyana.Demerara-Mahaica
Artibeus cinereus|ABGYF113-06|ROM 113652|Guyana.Demerara-Mahaica
Artibeus cinereus|ABGYF111-06|ROM 113650|Guyana.Demerara-Mahaica
Artibeus cinereus|ABGYE737-06|ROM 112680|Guyana.Demerara-Mahaica
Artibeus cinereus|ABGYE729-06|ROM 112669|Guyana.Demerara-Mahaica
Artibeus cinereus|ABGYE716-06|ROM 112651|Guyana.Demerara-Mahaica
Artibeus cinereus|ABGYE700-06|ROM 112634|Guyana.Demerara-Mahaica
Artibeus cinereus|ABGYE679-06|ROM 112607|Guyana.Demerara-Mahaica
Artibeus cinereus|ABGYE672-06|ROM 112595|Guyana.Demerara-Mahaica
Artibeus cinereus|ABGYE667-06|ROM 112588|Guyana.Demerara-Mahaica
Artibeus cinereus|ABGYE666-06|ROM 112587|Guyana.Demerara-Mahaica
Artibeus cinereus|ABGYE664-06|ROM 112585|Guyana.Demerara-Mahaica
Artibeus cinereus|ABGYE653-06|ROM 112570|Guyana.Demerara-Mahaica
Artibeus cinereus|ABGYB316-06|ROM 103021|Guyana.Upper Takutu-Upper Essequibo
Artibeus cinereus|ABGYB263-06|ROM 102967|Guyana.Upper Takutu-Upper Essequibo
Artibeus cinereus|ABGYB199-06|ROM 102901|Guyana.Upper Takutu-Upper Essequibo
Artibeus cinereus|ABGYA619-06|ROM 100310|Guyana.East Berbice-Corentyne
Artibeus cinereus|ABGYA550-06|ROM 100222|Guyana.East Berbice-Corentyne
Artibeus cinereus|ABGYA538-06|ROM 100209|Guyana.East Berbice-Corentyne
Artibeus cinereus|ABGYA426-06|ROM 98834|Guyana.Barima-Waini
Artibeus cinereus|ABGYA424-06|ROM 98832|Guyana.Barima-Waini
Artibeus cinereus|ABGYA298-06|ROM 98694|Guyana.Barima-Waini
Artibeus cinereus|ABGYA292-06|ROM 98211|Guyana.Demerara-Mahaica
Artibeus cinereus|ABGYA291-06|ROM 98210|Guyana.Demerara-Mahaica
Artibeus cinereus|ABGYA290-06|ROM 98209|Guyana.Demerara-Mahaica
Artibeus cinereus|ABGYA067-06|ROM 97889|Guyana.Upper Takutu-Upper Essequibo
Artibeus cinereus|ABGYB310-06|ROM 103015|Guyana.Upper Takutu-Upper Essequibo
Artibeus cinereus|ABGYA444-06|ROM 98853|Guyana.Barima-Waini
Artibeus cinereus|ABGYA373-06|ROM 98774|Guyana.Barima-Waini
Artibeus cinereus|ABGYA294-06|ROM 98213|Guyana.Demerara-Mahaica
Artibeus cinereus|ABGYF228-06|ROM 113803|Guyana.Demerara-Mahaica
Artibeus cinereus|BCBN169-05|ROM 98804|Guyana.Barima-Waini
Artibeus cinereus|BCBNT888-06|ROM 115519|Guyana.Essequibo Islands-West Demerara
Artibeus cinereus|ABGYG918-08|ROM 119346|Guyana
Artibeus cinereus|ABGYB275-06|ROM 102979|Guyana.Upper Takutu-Upper Essequibo
Artibeus cinereus|ABGYG916-08|ROM 119344|Guyana
Artibeus cinereus|ABGYG914-08|ROM 119342|Guyana
Artibeus cinereus|ABGYG890-08|ROM 119318|Guyana
Artibeus cinereus|ABGYG857-08|ROM 119285|Guyana
Artibeus cinereus|ABGYG699-08|ROM 119127|Guyana
Artibeus cinereus|ABGYG641-08|ROM 119069|Guyana

Artibeus cinereus|ABGYG857-08|ROM 119285|Guyana
Artibeus cinereus|ABGYG699-08|ROM 119127|Guyana
Artibeus cinereus|ABGYG641-08|ROM 119069|Guyana
Artibeus cinereus|ABGYB357-06|ROM 103064|Guyana.Upper Takutu-Upper Essequibo
Artibeus cinereus|ABGYB309-06|ROM 103014|Guyana.Upper Takutu-Upper Essequibo
Artibeus cinereus|ABGYA079-06|ROM 97902|Guyana.Upper Takutu-Upper Essequibo
Artibeus cinereus|BCBN119-05|ROM 97850|Guyana.Upper Takutu-Upper Essequibo
Artibeus cinereus|ABGYB558-06|ROM 103294|Guyana.Upper Takutu-Upper Essequibo
Artibeus cinereus|ABGYE731-06|ROM 112671|Guyana.Demerara-Mahaica
Artibeus cinereus|ABGYB314-06|ROM 103019|Guyana.Upper Takutu-Upper Essequibo
Artibeus cinereus|ABGYB366-06|ROM 103073|Guyana.Upper Takutu-Upper Essequibo
Artibeus cinereus|ABGYB367-06|ROM 103074|Guyana.Upper Takutu-Upper Essequibo
Artibeus cinereus|ABGYB368-06|ROM 103075|Guyana.Upper Takutu-Upper Essequibo
Artibeus cinereus|ABGYF158-06|ROM 113711|Guyana.Demerara-Mahaica
Artibeus cinereus|ABGYF163-06|ROM 113719|Guyana.Demerara-Mahaica
Artibeus cinereus|ABGYF200-06|ROM 113770|Guyana.Demerara-Mahaica
Artibeus cinereus|ABGYG593-08|ROM 119021|Guyana
Artibeus cinereus|ABGYG640-08|ROM 119068|Guyana
Artibeus cinereus|ABGYG698-08|ROM 119126|Guyana
Artibeus cinereus|ABGYG725-08|ROM 119153|Guyana
Artibeus cinereus|ABGYG760-08|ROM 119188|Guyana
Artibeus cinereus|ABGYG806-08|ROM 119234|Guyana
Artibeus cinereus|ABGYG829-08|ROM 119257|Guyana
Artibeus cinereus|ABGYG858-08|ROM 119286|Guyana
Artibeus cinereus|ABGYG891-08|ROM 119319|Guyana
Artibeus cinereus|ABGYG915-08|ROM 119343|Guyana
Artibeus cinereus|ABGYG917-08|ROM 119345|Guyana
Artibeus cinereus|ABGYG953-08|ROM 119381|Guyana
Artibeus cinereus|ABGYG955-08|ROM 119383|Guyana
Artibeus cinereus|ABGYG956-08|ROM 119384|Guyana
Artibeus cinereus|ABGYG987-08|ROM 119415|Guyana
Artibeus cinereus|ABGYB312-06|ROM 103017|Guyana.Upper Takutu-Upper Essequibo
Artibeus cinereus|ABGYB311-06|ROM 103016|Guyana.Upper Takutu-Upper Essequibo
Artibeus cinereus|ABGYB259-06|ROM 102963|Guyana.Upper Takutu-Upper Essequibo
Artibeus cinereus|ABGYA557-06|ROM 100232|Guyana.East Berbice-Corentyne
Artibeus cinereus|ABGYA549-06|ROM 100221|Guyana.East Berbice-Corentyne
Artibeus cinereus|ABGYA540-06|ROM 100211|Guyana.East Berbice-Corentyne
Artibeus cinereus|ABGYA299-06|ROM 98695|Guyana.Barima-Waini
Artibeus cinereus|ABGYA068-06|ROM 97890|Guyana.Upper Takutu-Upper Essequibo
Artibeus cinereus|ABGYA077-06|ROM 97900|Guyana.Upper Takutu-Upper Essequibo
Artibeus cinereus|ABGYA071-06|ROM 97893|Guyana.Upper Takutu-Upper Essequibo
Artibeus cinereus|ABGYA049-06|ROM 97851|Guyana.Upper Takutu-Upper Essequibo
Artibeus cinereus|ABGYE680-06|ROM 112608|Guyana.Demerara-Mahaica
Artibeus cinereus|ABGYG728-08|ROM 119156|Guyana
Artibeus cinereus|BCBN122-05|ROM 97858|Guyana.Upper Takutu-Upper Essequibo
Artibeus cinereus|ABGYF112-06|ROM 113651|Guyana.Demerara-Mahaica
Artibeus cinereus|ABGYF196-06|ROM 113760|Guyana.Demerara-Mahaica
Artibeus cinereus|BCBNT699-06|ROM 113832|Guyana.Demerara-Mahaica
Artibeus cinereus|ABGYA289-06|ROM 98208|Guyana.Demerara-Mahaica
Artibeus cinereus|ABGYB237-06|ROM 102939|Guyana.Upper Takutu-Upper Essequibo
Artibeus cinereus|ABGYA050-06|ROM 97852|Guyana.Upper Takutu-Upper Essequibo
Artibeus cinereus|ABGYA621-06|ROM 100314|Guyana.East Berbice-Corentyne
Artibeus cinereus|ABGYB236-06|ROM 102938|Guyana.Upper Takutu-Upper Essequibo
Artibeus cinereus|ABGYB541-06|ROM 103276|Guyana.Upper Takutu-Upper Essequibo
Artibeus cinereus|ABGYB543-06|ROM 103278|Guyana.Upper Takutu-Upper Essequibo
Artibeus cinereus|ABGYF131-06|ROM 113679|Guyana.Demerara-Mahaica
Artibeus cinereus|ABGYA889-07|ROM 100313|Guyana.East Berbice-Corentyne
Artibeus cinereus|BCBNT869-06|ROM 115474|Guyana.Essequibo Islands-West Demerara
Artibeus cinereus|ABGYA539-06|ROM 100210|Guyana.East Berbice-Corentyne
Artibeus cinereus|ABGYB198-06|ROM 102900|Guyana.Upper Takutu-Upper Essequibo
Artibeus cinereus|ABGYA645-06|ROM 100341|Guyana.East Berbice-Corentyne
Artibeus cinereus|ABGYA070-06|ROM 97892|Guyana.Upper Takutu-Upper Essequibo
Artibeus cinereus|ABGYB504-06|ROM 103239|Guyana.Upper Takutu-Upper Essequibo
Artibeus cinereus|ABGYD109-06|ROM 108407|Guyana.Potaro-Siparuni
Artibeus cinereus|ABGYF202-06|ROM 113772|Guyana.Demerara-Mahaica
Artibeus cinereus|BCBNT889-06|ROM 115520|Guyana.Essequibo Islands-West Demerara
Artibeus bogotensis|ABGYG443-06|ROM 116579|Guyana.Potaro-Siparuni
Artibeus bogotensis|ABGYG451-06|ROM 116588|Guyana.Potaro-Siparuni
Artibeus bogotensis|ABGYG830-08|ROM 119258|Guyana
Artibeus bogotensis|ABGYG576-06|ROM 116738|Guyana.Potaro-Siparuni
Artibeus bogotensis|ABGYG398-06|ROM 116526|Guyana.Potaro-Siparuni
Artibeus bogotensis|ABGYD484-06|ROM 108837|Guyana.Potaro-Siparuni
Artibeus bogotensis|ABGYB916-06|ROM 104761|Guyana.Potaro-Siparuni
Artibeus bogotensis|BCBNC008-06|ROM 114695|Guyana.Potaro-Siparuni
Artibeus bogotensis|BCBNC147-06|ROM 115866|Guyana.Potaro-Siparuni
Artibeus bogotensis|BCBNT248-06|ROM 108177|Guyana.Cuyuni-Mazaruni
Artibeus bogotensis|ABGYE366-06|ROM 111816|Guyana.Potaro-Siparuni
Artibeus bogotensis|ABGYG479-06|ROM 116619|Guyana.Potaro-Siparuni
Artibeus bogotensis|BCBNT261-06|ROM 108222|Guyana.Cuyuni-Mazaruni
Artibeus bogotensis|ABGYG425-06|ROM 116558|Guyana.Potaro-Siparuni
Artibeus bogotensis|ABGYG478-06|ROM 116618|Guyana.Potaro-Siparuni
Artibeus bogotensis|ABGYG515-06|ROM 116657|Guyana.Potaro-Siparuni
Artibeus bogotensis|BCBNT811-06|ROM 114118|Suriname.Brokopondo
Artibeus bogotensis|ABGYD699-06|ROM 109112|Guyana.Potaro-Siparuni
Artibeus bogotensis|ABGYB313-06|ROM 103018|Guyana.Upper Takutu-Upper Essequibo
Artibeus bogotensis|BCBNT527-06|ROM 111905|Guyana.Potaro-Siparuni
Artibeus bogotensis|ABGYD338-06|ROM 108678|Guyana.Potaro-Siparuni
Artibeus bogotensis|BCBNT247-06|ROM 108176|Guyana.Cuyuni-Mazaruni
Artibeus bogotensis|BCBNC012-06|ROM 114703|Guyana.Potaro-Siparuni
Artibeus bogotensis|BCBNC032-06|ROM 114757|Guyana.Potaro-Siparuni
Artibeus bogotensis|ABGYG783-08|ROM 119211|Guyana
Artibeus bogotensis|ABGYG761-08|ROM 119189|Guyana
Artibeus bogotensis|ABGYG729-08|ROM 119157|Guyana
Artibeus bogotensis|ABGYD520-06|ROM 108878|Guyana.Potaro-Siparuni
Artibeus bogotensis|ABGYB274-06|ROM 102978|Guyana.Upper Takutu-Upper Essequibo
Artibeus bogotensis|BCBNT262-06|ROM 108223|Guyana.Cuyuni-Mazaruni
Artibeus bogotensis|ABGYG516-06|ROM 116658|Guyana.Potaro-Siparuni
Artibeus bogotensis|BCBNC112-06|ROM 115755|Guyana.Potaro-Siparuni
Artibeus bogotensis|ABGYE380-06|ROM 111832|Guyana.Potaro-Siparuni
Artibeus bogotensis|ABGYG429-06|ROM 116564|Guyana.Potaro-Siparuni
Artibeus bogotensis|ABGYD285-06|ROM 108623|Guyana.Potaro-Siparuni
Artibeus bogotensis|ABGYD319-06|ROM 108659|Guyana.Potaro-Siparuni
Artibeus bogotensis|ABGYD344-06|ROM 108685|Guyana.Potaro-Siparuni

Artibeus bogotensis|ABGYD285-06|ROM 108623|Guyana.Potaro-Siparuni
Artibeus bogotensis|ABGYD319-06|ROM 108659|Guyana.Potaro-Siparuni
Artibeus bogotensis|ABGYD344-06|ROM 108685|Guyana.Potaro-Siparuni
Artibeus bogotensis|ABGYE368-06|ROM 111818|Guyana.Potaro-Siparuni
Artibeus bogotensis|BCBNC155-06|ROM 115917|Guyana.Potaro-Siparuni
Artibeus bogotensis|BCBNT521-06|ROM 111872|Guyana.Potaro-Siparuni
Artibeus bogotensis|ABGYG785-08|ROM 119213|Guyana
Artibeus bogotensis|ABGYB400-06|ROM 103107|Guyana.Upper Takutu-Upper Essequibo
Artibeus bogotensis|BCBNT636-06|ROM 113581|Guyana.Upper Takutu-Upper Essequibo
Artibeus bogotensis|ABGYD310-06|ROM 108649|Guyana.Potaro-Siparuni
Artibeus bogotensis|ABGYB331-06|ROM 103038|Guyana.Upper Takutu-Upper Essequibo
Artibeus bogotensis|BCBNT783-06|ROM 114019|Suriname.Brokopondo
Artibeus bogotensis|ABGYD273-06|ROM 108609|Guyana.Potaro-Siparuni
Artibeus bogotensis|ABGYG489-06|ROM 116629|Guyana.Potaro-Siparuni
Artibeus bogotensis|BCBNT224-06|ROM 107942|Venezuela.Bolivar
Artibeus bogotensis|ABGYG463-06|ROM 116601|Guyana.Potaro-Siparuni
Artibeus bogotensis|ABGYB514-06|ROM 103249|Guyana.Upper Takutu-Upper Essequibo
Artibeus bogotensis|ABGYD318-06|ROM 108658|Guyana.Potaro-Siparuni
Artibeus bogotensis|BCBNT201-06|ROM 107907|Venezuela.Bolivar
Artibeus bogotensis|BCBNC154-06|ROM 115916|Guyana.Potaro-Siparuni
Artibeus bogotensis|ABGYD189-06|ROM 108516|Guyana.Potaro-Siparuni
Artibeus bogotensis|ABGYC718-06|ROM 108118|Guyana.Cuyuni-Mazaruni
Artibeus bogotensis|ABGYA145-06|ROM 97989|Guyana.Potaro-Siparuni
Artibeus bogotensis|ABGYE376-06|ROM 111828|Guyana.Potaro-Siparuni
Artibeus bogotensis|ABGYB402-06|ROM 103109|Guyana.Upper Takutu-Upper Essequibo
Artibeus bogotensis|ABGYG424-06|ROM 116557|Guyana.Potaro-Siparuni
Artibeus bogotensis|ABGYG438-06|ROM 116573|Guyana.Potaro-Siparuni
Artibeus bogotensis|ABSRA511-06|ROM 117101|Suriname.Nickerie
Artibeus bogotensis|BCBNC033-06|ROM 114758|Guyana.Potaro-Siparuni
Artibeus bogotensis|BCBNC121-06|ROM 115774|Guyana.Potaro-Siparuni
Artibeus bogotensis|BCBNT525-06|ROM 111893|Guyana.Potaro-Siparuni
Artibeus bogotensis|BCBNT841-06|ROM 114197|Suriname.Brokopondo
Artibeus bogotensis|BCBNT742-06|ROM 113921|Suriname.Brokopondo
Artibeus bogotensis|BCBNT840-06|ROM 114196|Suriname.Brokopondo
Artibeus bogotensis|BCBNT944-06|ROM 115662|Guyana.Potaro-Siparuni
Artibeus gnomus|ABGYE219-06|ROM 111667|Guyana.Potaro-Siparuni
Artibeus gnomus|ABGYC575-06|ROM 107423|Guyana.Potaro-Siparuni
Artibeus gnomus|ABGYD088-06|ROM 108385|Guyana.Potaro-Siparuni
Artibeus gnomus|ABSRA407-06|ROM 116996|Suriname.Nickerie
Artibeus gnomus|ABGYE274-06|ROM 111723|Guyana.Potaro-Siparuni
Artibeus gnomus|ABGYE268-06|ROM 111717|Guyana.Potaro-Siparuni
Artibeus gnomus|ABGYE230-06|ROM 111678|Guyana.Potaro-Siparuni
Artibeus gnomus|ABGYE157-06|ROM 111600|Guyana.Potaro-Siparuni
Artibeus gnomus|ABGYC594-06|ROM 107450|Guyana.Potaro-Siparuni
Artibeus gnomus|ABGYC352-06|ROM 107164|Guyana.Potaro-Siparuni
Artibeus gnomus|ABGYC015-06|ROM 104792|Guyana.Potaro-Siparuni
Artibeus gnomus|ABGYB915-06|ROM 104760|Guyana.Potaro-Siparuni
Artibeus gnomus|ABGYB260-06|ROM 102964|Guyana.Upper Takutu-Upper Essequibo
Artibeus gnomus|ABGYB771-06|ROM 103539|Guyana.Upper Demerara-Berbice
Artibeus gnomus|ABGYB276-06|ROM 102980|Guyana.Upper Takutu-Upper Essequibo
Artibeus gnomus|BCBNC022-06|ROM 114730|Guyana.Potaro-Siparuni
Artibeus gnomus|BCBNT870-06|ROM 115475|Guyana.Essequibo Islands-West Demerara
Artibeus gnomus|BCBNT902-06|ROM 115544|Guyana.Essequibo Islands-West Demerara
Artibeus gnomus|ABECA634-06|ROM 105680|Ecuador.Napo
Artibeus gnomus|ABECA022-06|ROM 104004|Ecuador.Napo
Artibeus gnomus|ABECA701-06|ROM 105761|Ecuador.Napo
Artibeus gnomus|BCBN837-05|ROM 105502|Ecuador.Napo
Artibeus gnomus|ABECA312-06|ROM 105189|Ecuador.Napo
Artibeus gnomus|ABECA851-06|ROM 106026|Ecuador.Napo
Artibeus gnomus|ABECA139-06|ROM 104437|Ecuador.Napo
Artibeus gnomus|ABECA529-06|ROM 105559|Ecuador.Napo
Artibeus gnomus|ABECA140-06|ROM 104438|Ecuador.Napo
Artibeus gnomus|ABECA850-06|ROM 106025|Ecuador.Napo
Artibeus gnomus|ABECA439-06|ROM 105340|Ecuador.Napo
Artibeus gnomus|ABECA938-06|ROM 106121|Ecuador.Napo
Artibeus gnomus|BCBN774-05|ROM 105126|Ecuador.Napo
Artibeus gnomus|ABECA052-06|ROM 104036|Ecuador.Napo
Artibeus gnomus|ABECA173-06|ROM 104486|Ecuador.Napo
Artibeus gnomus|ABECA271-06|ROM 105139|Ecuador.Napo
Artibeus gnomus|BCBN836-05|ROM 105501|Ecuador.Napo
Artibeus gnomus|BCBN947-05|ROM 105896|Ecuador.Napo
Artibeus gnomus|ABECA303-06|ROM 105175|Ecuador.Napo
Artibeus gnomus|ABECA446-06|ROM 105350|Ecuador.Napo
Artibeus gnomus|ABECA261-06|ROM 105129|Ecuador.Napo
Artibeus gnomus|ABECA023-06|ROM 104005|Ecuador.Napo
Artibeus gnomus|BCBN952-05|ROM 105919|Ecuador.Napo
Artibeus gnomus|BCBNT193-06|ROM 107894|Venezuela.Amazonas
Artibeus gnomus|ABGYE183-06|ROM 111626|Guyana.Potaro-Siparuni
Artibeus gnomus|ABGYD665-06|ROM 109076|Guyana.Potaro-Siparuni
Artibeus gnomus|ABGYC361-06|ROM 107176|Guyana.Potaro-Siparuni
Artibeus gnomus|ABGYC110-06|ROM 106620|Guyana.Upper Takutu-Upper Essequibo
Artibeus gnomus|ABGYE321-06|ROM 111771|Guyana.Potaro-Siparuni
Artibeus gnomus|ABGYD235-06|ROM 108567|Guyana.Potaro-Siparuni
Artibeus gnomus|ABGYE290-06|ROM 111739|Guyana.Potaro-Siparuni
Artibeus gnomus|ABGYE454-06|ROM 111918|Guyana.Potaro-Siparuni
Artibeus gnomus|ABSMS308-06|ROM 117470|Suriname.Sipaliwini
Artibeus gnomus|BCBNT926-06|ROM 115608|Guyana.Essequibo Islands-West Demerara
Artibeus gnomus|BCBNT351-06|ROM 109013|Guyana.Potaro-Siparuni
Artibeus gnomus|ABGYB765-06|ROM 103533|Guyana.Upper Demerara-Berbice
Artibeus gnomus|ABGYE675-06|ROM 112602|Guyana.Demerara-Mahaica
Artibeus gnomus|BCBNT909-06|ROM 115572|Guyana.Essequibo Islands-West Demerara
Artibeus gnomus|ABGYE255-06|ROM 111704|Guyana.Potaro-Siparuni
Artibeus gnomus|ABGYE194-06|ROM 111640|Guyana.Potaro-Siparuni
Artibeus gnomus|ABGYE220-06|ROM 111668|Guyana.Potaro-Siparuni
Artibeus gnomus|ABGYG506-06|ROM 116648|Guyana.Potaro-Siparuni
Artibeus gnomus|ABGYE527-06|ROM 112000|Guyana.Potaro-Siparuni
Artibeus gnomus|ABGYE528-06|ROM 112001|Guyana.Potaro-Siparuni
Artibeus gnomus|ABGYC360-06|ROM 107175|Guyana.Potaro-Siparuni
Artibeus gnomus|ABGYE417-06|ROM 111873|Guyana.Potaro-Siparuni
Artibeus gnomus|BCBN304-05|ROM 100315|Guyana.East Berbice-Corentyne
Artibeus gnomus|ABGYB197-06|ROM 102899|Guyana.Upper Takutu-Upper Essequibo
Artibeus gnomus|ABGYC370-06|ROM 107186|Guyana.Potaro-Siparuni
Artibeus gnomus|ABGYE108-06|ROM 111551|Guyana.Potaro-Siparuni

[illegible]
Artibeus gnomus|ABGYB197-06|ROM 102899|Guyana.Upper Takutu-Upper Essequibo
Artibeus gnomus|ABGYC370-06|ROM 107186|Guyana.Potaro-Siparuni
Artibeus gnomus|ABGYE108-06|ROM 111551|Guyana.Potaro-Siparuni
Artibeus gnomus|ABGYE803-06|ROM 113405|Guyana.Upper Demerara-Berbice
Artibeus gnomus|ABGYE174-06|ROM 111617|Guyana.Potaro-Siparuni
Artibeus gnomus|ABGYE665-06|ROM 112586|Guyana.Demerara-Mahaica
Artibeus gnomus|ABGYA507-06|ROM 98918|Guyana.Barima-Waini
Artibeus gnomus|ABGYC505-06|ROM 107340|Guyana.Potaro-Siparuni
Artibeus gnomus|ABGYF222-06|ROM 113793|Guyana.Demerara-Mahaica
Artibeus gnomus|ABGYG437-06|ROM 116572|Guyana.Potaro-Siparuni
Artibeus gnomus|ABGYC818-06|ROM 108221|Guyana.Cuyuni-Mazaruni
Artibeus gnomus|ABGYA349-06|ROM 98749|Guyana.Barima-Waini
Artibeus gnomus|ABGYG426-06|ROM 116559|Guyana.Potaro-Siparuni
Artibeus gnomus|ABGYE391-06|ROM 111843|Guyana.Potaro-Siparuni
Artibeus gnomus|ABGYE453-06|ROM 111917|Guyana.Potaro-Siparuni
Artibeus gnomus|ABGYE460-06|ROM 111924|Guyana.Potaro-Siparuni
Artibeus gnomus|ABGYE378-06|ROM 111830|Guyana.Potaro-Siparuni
Artibeus gnomus|ABGYE452-06|ROM 111916|Guyana.Potaro-Siparuni
Artibeus gnomus|ABGYE275-06|ROM 111724|Guyana.Potaro-Siparuni
Artibeus gnomus|ABGYD360-06|ROM 108703|Guyana.Potaro-Siparuni
Artibeus gnomus|ABGYD233-06|ROM 108565|Guyana.Potaro-Siparuni
Artibeus gnomus|ABGYD089-06|ROM 108386|Guyana.Potaro-Siparuni
Artibeus gnomus|ABGYC740-06|ROM 108141|Guyana.Cuyuni-Mazaruni
Artibeus gnomus|ABGYB767-06|ROM 103535|Guyana.Upper Demerara-Berbice
Artibeus gnomus|ABGYB135-06|ROM 101117|Guyana.Barima-Waini
Artibeus gnomus|ABGYA847-06|ROM 100911|Guyana.Barima-Waini
Artibeus gnomus|ABGYA464-06|ROM 98874|Guyana.Barima-Waini
Artibeus gnomus|ABGYA453-06|ROM 98863|Guyana.Barima-Waini
Artibeus gnomus|ABGYB102-06|ROM 101080|Guyana.Barima-Waini
Artibeus gnomus|ABGYB369-06|ROM 103076|Guyana.Upper Takutu-Upper Essequibo
Artibeus gnomus|ABGYD211-06|ROM 108542|Guyana.Potaro-Siparuni
Artibeus gnomus|ABGYF164-06|ROM 113720|Guyana.Demerara-Mahaica
Artibeus gnomus|ABGYG460-06|ROM 116597|Guyana.Potaro-Siparuni
Artibeus gnomus|ABGYG566-06|ROM 116720|Guyana.Potaro-Siparuni
Artibeus gnomus|ABSMS139-06|ROM 117301|Suriname.Sipaliwini
Artibeus gnomus|BCBN302-05|ROM 100309|Guyana.East Berbice-Corentyne
Artibeus gnomus|BCBN306-05|ROM 100340|Guyana.East Berbice-Corentyne
Artibeus gnomus|ABGYC574-06|ROM 107422|Guyana.Potaro-Siparuni
Artibeus gnomus|BCBNT310-06|ROM 108657|Guyana.Potaro-Siparuni
Artibeus gnomus|ABGYB889-06|ROM 104734|Guyana.Potaro-Siparuni
Artibeus gnomus|ABGYE238-06|ROM 111686|Guyana.Potaro-Siparuni
Artibeus gnomus|BCBNT364-06|ROM 108992|Guyana.Potaro-Siparuni
Artibeus gnomus|ABGYC693-06|ROM 108092|Guyana.Cuyuni-Mazaruni
Artibeus gnomus|ABGYG430-06|ROM 116565|Guyana.Potaro-Siparuni
Artibeus gnomus|BCBNT637-06|ROM 113582|Guyana.Upper Takutu-Upper Essequibo
Artibeus gnomus|ABGYD212-06|ROM 108543|Guyana.Potaro-Siparuni
Artibeus gnomus|ABGYE156-06|ROM 111599|Guyana.Potaro-Siparuni
Artibeus gnomus|ABGYC583-06|ROM 107432|Guyana.Potaro-Siparuni
Artibeus gnomus|ABGYC592-06|ROM 107448|Guyana.Potaro-Siparuni
Artibeus gnomus|ABGYC504-06|ROM 107339|Guyana.Potaro-Siparuni
Artibeus gnomus|ABGYD138-06|ROM 108441|Guyana.Potaro-Siparuni
Artibeus gnomus|ABGYE239-06|ROM 111687|Guyana.Potaro-Siparuni
Artibeus gnomus|ABGYD087-06|ROM 108384|Guyana.Potaro-Siparuni
Artibeus gnomus|ABGYE433-06|ROM 111892|Guyana.Potaro-Siparuni
Artibeus gnomus|ABGYE310-06|ROM 111760|Guyana.Potaro-Siparuni
Artibeus gnomus|ABGYE586-06|ROM 112063|Guyana.Potaro-Siparuni
Artibeus gnomus|ABGYD728-06|ROM 109144|Guyana.Potaro-Siparuni
Artibeus gnomus|ABGYE083-06|ROM 111526|Guyana.Potaro-Siparuni
Artibeus gnomus|ABGYA187-06|ROM 98055|Guyana.Potaro-Siparuni
Artibeus gnomus|ABGYB906-06|ROM 104751|Guyana.Potaro-Siparuni
Artibeus gnomus|ABGYD234-06|ROM 108566|Guyana.Potaro-Siparuni
Artibeus gnomus|ABGYD521-06|ROM 108879|Guyana.Potaro-Siparuni
Artibeus gnomus|ABGYE860-06|ROM 113476|Guyana.Upper Takutu-Upper Essequibo
Artibeus gnomus|ABSMS416-06|ROM 117578|Suriname.Sipaliwini
Artibeus gnomus|ABGYG507-06|ROM 116649|Guyana.Potaro-Siparuni
Artibeus gnomus|ABGYE377-06|ROM 111829|Guyana.Potaro-Siparuni
Artibeus gnomus|ABGYE379-06|ROM 111831|Guyana.Potaro-Siparuni
Artibeus gnomus|ABGYC544-06|ROM 107383|Guyana.Potaro-Siparuni
Artibeus gnomus|ABGYE240-06|ROM 111688|Guyana.Potaro-Siparuni
Artibeus gnomus|ABGYB870-06|ROM 104714|Guyana.Potaro-Siparuni
Artibeus gnomus|ABGYC479-06|ROM 107312|Guyana.Potaro-Siparuni
Artibeus gnomus|ABSMS402-06|ROM 117564|Suriname.Sipaliwini
Artibeus gnomus|BCBN303-05|ROM 100311|Guyana.East Berbice-Corentyne
Artibeus gnomus|ABGYE820-06|ROM 113426|Guyana.Upper Demerara-Berbice
Artibeus gnomus|ABGYF231-06|ROM 113806|Guyana.Demerara-Mahaica
Artibeus gnomus|BCBNT514-06|ROM 111651|Guyana.Potaro-Siparuni
Artibeus gnomus|ABGYC380-06|ROM 107197|Guyana.Potaro-Siparuni
Artibeus gnomus|BCBNT733-06|ROM 113904|Suriname.Brokopondo
Artibeus gnomus|BCBNT734-06|ROM 113905|Suriname.Brokopondo
Artibeus gnomus|ABGYC528-06|ROM 107366|Guyana.Potaro-Siparuni
Artibeus gnomus|ABGYE301-06|ROM 111751|Guyana.Potaro-Siparuni
Artibeus gnomus|BCBN296-05|ROM 100261|Guyana.East Berbice-Corentyne
Artibeus gnomus|BCBNT890-06|ROM 115521|Guyana.Essequibo Islands-West Demerara
Artibeus gnomus|ABGYA670-06|ROM 100374|Guyana.East Berbice-Corentyne
Artibeus gnomus|ABGYC240-06|ROM 106793|Guyana.Upper Takutu-Upper Essequibo
Artibeus gnomus|ABGYC403-06|ROM 107222|Guyana.Potaro-Siparuni
Artibeus gnomus|ABGYA293-06|ROM 98212|Guyana.Demerara-Mahaica
Artibeus gnomus|ABGYE455-06|ROM 111919|Guyana.Potaro-Siparuni
Artibeus gnomus|BCBNT993-06|ROM 114664|Guyana.Potaro-Siparuni
Centurio senex|BCBN389-05|ROM 101257|El Salvador.Ahuachapan
Centurio senex|ABMXA808-06|ROM FN30518|Mexico.Campeche
Centurio senex|BCBN001-05|ROM 95288|Mexico.Campeche
Centurio senex|ABCSA211-06|ROM 98482|Guatemala.El Progreso
Centurio senex|ABMXB695-06|ROM 95446|Mexico.Campeche
Centurio senex|BCBN004-05|ROM 95382|Mexico.Campeche
Centurio senex|ABMXB777-06|ROM 95739|Mexico.Campeche
Centurio senex|ABMXB698-06|ROM 95449|Mexico.Campeche
Centurio senex|ABMXB724-06|ROM 95564|Mexico.Campeche
Centurio senex|ABMXB721-06|ROM 95561|Mexico.Campeche
Centurio senex|ABMXB726-06|ROM 95566|Mexico.Campeche
Centurio senex|ABMXB727-06|ROM 95567|Mexico.Campeche
Centurio senex|BCBN387-05|ROM 101252|El Salvador.Ahuachapan
Centurio senex|ABMXB703-06|ROM 95454|Mexico.Campeche

Centurio senex|ABMXB726-06|ROM 95566|Mexico.Campeche
Centurio senex|ABMXB727-06|ROM 95567|Mexico.Campeche
Centurio senex|BCBN387-05|ROM 101252|El Salvador.Ahuachapan
Centurio senex|ABMXB703-06|ROM 95454|Mexico.Campeche
Centurio senex|ABMXB729-06|ROM 95569|Mexico.Campeche
Centurio senex|ABMXB697-06|ROM FN29239|Mexico.Campeche
Centurio senex|ABMXB699-06|ROM 95450|Mexico.Campeche
Centurio senex|ABMXB725-06|ROM 95565|Mexico.Campeche
Centurio senex|ABMXB728-06|ROM 95568|Mexico.Campeche
Centurio senex|BCBN002-05|ROM 95289|Mexico.Campeche
Centurio senex|BCBN216-05|ROM 99584|Guatemala.Peten
Centurio senex|BCBN251-05|ROM 99673|Guatemala.El Progreso
Centurio senex|ABMXB694-06|ROM FN29236|Mexico.Campeche
Centurio senex|ABMXB776-06|ROM 95738|Mexico.Campeche
Centurio senex|ABMXB696-06|ROM 95447|Mexico.Campeche
Centurio senex|ABCSA743-06|ROM 101330|El Salvador.Ahuachapan
Centurio senex|BCBN091-05|ROM 98481|Guatemala.El Progreso
Centurio senex|ABMXB700-06|ROM 95451|Mexico.Campeche
Centurio senex|ABMXB775-06|ROM 95737|Mexico.Campeche
Centurio senex|BCBN003-05|ROM 95381|Mexico.Campeche
Centurio senex|BCBN250-05|ROM 99672|Guatemala.El Progreso
Centurio senex|BCBN383-05|ROM 101239|El Salvador.Ahuachapan
Centurio senex|BCBN418-05|ROM 101329|El Salvador.Ahuachapan
Centurio senex|BCBN407-05|ROM 101300|El Salvador.Ahuachapan
Centurio senex|ABMXB702-06|ROM 95453|Mexico.Campeche
Centurio senex|ABCSA688-06|ROM 101238|El Salvador.Ahuachapan
Centurio senex|ABCSA698-06|ROM 101255|El Salvador.Ahuachapan
Centurio senex|ABMXB701-06|ROM 95452|Mexico.Campeche
Centurio senex|ABMXB720-06|ROM 95560|Mexico.Campeche
Centurio senex|ABMXB722-06|ROM 95562|Mexico.Campeche
Centurio senex|ABMXB723-06|ROM 95563|Mexico.Campeche
Centurio senex|ABCSA699-06|ROM 101256|El Salvador.Ahuachapan
Centurio senex|BCBN215-05|ROM 99583|Guatemala.Peten
Centurio senex|BCBN482-05|ROM 97430|Mexico.Quintana Roo
Ametrida centurio|ABGYB565-06|ROM 103301|Guyana.Upper Takutu-Upper Essequibo
Ametrida centurio|ABGYC500-06|ROM 107334|Guyana.Potaro-Siparuni
Ametrida centurio|ABGYC557-06|ROM 107401|Guyana.Potaro-Siparuni
Ametrida centurio|ABGYD705-06|ROM 109119|Guyana.Potaro-Siparuni
Ametrida centurio|ABGYB508-06|ROM 103243|Guyana.Upper Takutu-Upper Essequibo
Ametrida centurio|ABGYB511-06|ROM 103246|Guyana.Upper Takutu-Upper Essequibo
Ametrida centurio|ABGYD817-06|ROM 109240|Guyana.Potaro-Siparuni
Ametrida centurio|ABGYG726-08|ROM 119154|Guyana
Ametrida centurio|ABSRA484-06|ROM 117074|Suriname.Nickerie
Ametrida centurio|ABSRA482-06|ROM 117072|Suriname.Nickerie
Ametrida centurio|ABSRA498-06|ROM 117088|Suriname.Nickerie
Ametrida centurio|ABGYD827-06|ROM 109250|Guyana.Potaro-Siparuni
Ametrida centurio|ABGYC708-06|ROM 108107|Guyana.Cuyuni-Mazaruni
Ametrida centurio|ABGYE002-06|ROM 109283|Guyana.Potaro-Siparuni
Ametrida centurio|ABGYG376-06|ROM 116499|Guyana.Potaro-Siparuni
Ametrida centurio|ABSMS005-06|ROM 117167|Suriname.Sipaliwini
Ametrida centurio|ABGYD809-06|ROM 109232|Guyana.Potaro-Siparuni
Ametrida centurio|ABSRA370-06|ROM 116959|Suriname.Nickerie
Ametrida centurio|ABGYD825-06|ROM 109248|Guyana.Potaro-Siparuni
Ametrida centurio|ABGYC513-06|ROM 107349|Guyana.Potaro-Siparuni
Ametrida centurio|ABGYB467-06|ROM 103201|Guyana.Upper Takutu-Upper Essequibo
Ametrida centurio|BCBN167-05|ROM 98798|Guyana.Barima-Waini
Ametrida centurio|ABGYE420-06|ROM 111878|Guyana.Potaro-Siparuni
Ametrida centurio|ABGYG551-06|ROM 116697|Guyana.Potaro-Siparuni
Ametrida centurio|ABGYD854-06|ROM 109281|Guyana.Potaro-Siparuni
Ametrida centurio|ABGYD808-06|ROM 109231|Guyana.Potaro-Siparuni
Ametrida centurio|ABGYD806-06|ROM 109229|Guyana.Potaro-Siparuni
Ametrida centurio|ABSRA278-06|ROM 116867|Suriname.Nickerie
Ametrida centurio|ABGYD532-06|ROM 108891|Guyana.Potaro-Siparuni
Ametrida centurio|ABSRA471-06|ROM 117061|Suriname.Nickerie
Ametrida centurio|ABGYD732-06|ROM 109148|Guyana.Potaro-Siparuni
Ametrida centurio|ABGYC554-06|ROM 107398|Guyana.Potaro-Siparuni
Ametrida centurio|ABGYC337-06|ROM 107149|Guyana.Potaro-Siparuni
Ametrida centurio|ABGYD733-06|ROM 109149|Guyana.Potaro-Siparuni
Ametrida centurio|ABGYD801-06|ROM 109223|Guyana.Potaro-Siparuni
Ametrida centurio|ABGYD816-06|ROM 109239|Guyana.Potaro-Siparuni
Ametrida centurio|ABGYB078-06|ROM 101055|Guyana.Barima-Waini
Ametrida centurio|ABGYC670-06|ROM 108076|Guyana.Cuyuni-Mazaruni
Ametrida centurio|ABGYG814-08|ROM 119242|Guyana
Ametrida centurio|ABSRA499-06|ROM 117089|Suriname.Nickerie
Ametrida centurio|ABGYG790-08|ROM 119218|Guyana
Ametrida centurio|ABGYG832-08|ROM 119260|Guyana
Ametrida centurio|ABGYD777-06|ROM 109198|Guyana.Potaro-Siparuni
Ametrida centurio|ABGYC311-06|ROM 107122|Guyana.Potaro-Siparuni
Ametrida centurio|ABGYC646-06|ROM 107464|Guyana.Potaro-Siparuni
Ametrida centurio|ABGYD807-06|ROM 109230|Guyana.Potaro-Siparuni
Ametrida centurio|ABGYD822-06|ROM 109245|Guyana.Potaro-Siparuni
Ametrida centurio|ABGYG550-06|ROM 116696|Guyana.Potaro-Siparuni
Ametrida centurio|ABGYD731-06|ROM 109147|Guyana.Potaro-Siparuni
Ametrida centurio|ABSRA277-06|ROM 116866|Suriname.Nickerie
Ametrida centurio|ABGYB303-06|ROM 103008|Guyana.Upper Takutu-Upper Essequibo
Ametrida centurio|ABSRA307-06|ROM 116896|Suriname.Nickerie
Ametrida centurio|ABGYB289-06|ROM 102993|Guyana.Upper Takutu-Upper Essequibo
Ametrida centurio|ABGYD824-06|ROM 109247|Guyana.Potaro-Siparuni
Ametrida centurio|ABGYE444-06|ROM 111906|Guyana.Potaro-Siparuni
Ametrida centurio|ABGYE360-06|ROM 111810|Guyana.Potaro-Siparuni
Ametrida centurio|ABGYE001-06|ROM 109282|Guyana.Potaro-Siparuni
Ametrida centurio|ABGYB077-06|ROM 101054|Guyana.Barima-Waini
Ametrida centurio|ABGYG449-06|ROM 116586|Guyana.Potaro-Siparuni
Ametrida centurio|ABGYG957-08|ROM 119385|Guyana
Ametrida centurio|ABGYB334-06|ROM 103041|Guyana.Upper Takutu-Upper Essequibo
Ametrida centurio|ABSRA371-06|ROM 116960|Suriname.Nickerie
Ametrida centurio|ABSRA405-06|ROM 116994|Suriname.Nickerie
Ametrida centurio|ABGYC363-06|ROM 107179|Guyana.Potaro-Siparuni
Ametrida centurio|ABGYE459-06|ROM 111923|Guyana.Potaro-Siparuni
Ametrida centurio|ABRMM077-07|ROM F39670|Guyana.Potaro-Siparuni
Ametrida centurio|ABSRA483-06|ROM 117073|Suriname.Nickerie
Ametrida centurio|ABGYC555-06|ROM 107399|Guyana.Potaro-Siparuni
Ametrida centurio|ABSRA304-06|ROM 116893|Suriname.Nickerie
[illegible]

Ametrida centurio|ABSRA483-06|ROM 117073|Suriname.Nickerie
Ametrida centurio|ABGYC555-06|ROM 107399|Guyana.Potaro-Siparuni
Ametrida centurio|ABSRA304-06|ROM 116893|Suriname.Nickerie
Ametrida centurio|ABGYA833-06|ROM 100893|Guyana.Barima-Waini
Ametrida centurio|ABGYD818-06|ROM 109241|Guyana.Potaro-Siparuni
Ametrida centurio|BCBN328-05|ROM 100892|Guyana.Barima-Waini
Ametrida centurio|ABGYD823-06|ROM 109246|Guyana.Potaro-Siparuni
Ametrida centurio|BCBNT311-06|ROM 108680|Guyana.Potaro-Siparuni
Ametrida centurio|ABGYB302-06|ROM 103007|Guyana.Upper Takutu-Upper Essequibo
Ametrida centurio|ABGYC566-06|ROM 107411|Guyana.Potaro-Siparuni
Ametrida centurio|ABGYD474-06|ROM 108825|Guyana.Potaro-Siparuni
Ametrida centurio|ABGYD765-06|ROM 109185|Guyana.Potaro-Siparuni
Ametrida centurio|BCBNT313-06|ROM 108700|Guyana.Potaro-Siparuni
Ametrida centurio|BCBN319-05|ROM 100832|Guyana.Barima-Waini
Ametrida centurio|BCBNT098-06|ROM 107108|Guyana.Potaro-Siparuni
Ametrida centurio|ABGYG441-06|ROM 116576|Guyana.Potaro-Siparuni
Ametrida centurio|ABGYD819-06|ROM 109242|Guyana.Potaro-Siparuni
Ametrida centurio|ABGYD821-06|ROM 109244|Guyana.Potaro-Siparuni
Ametrida centurio|ABGYE458-06|ROM 111922|Guyana.Potaro-Siparuni
Ametrida centurio|ABSRA378-06|ROM 116967|Suriname.Nickerie
Ametrida centurio|ABGYC556-06|ROM 107400|Guyana.Potaro-Siparuni
Ametrida centurio|ABSRA513-06|ROM 117103|Suriname.Nickerie
Ametrida centurio|ABSRA514-06|ROM 117104|Suriname.Nickerie
Ametrida centurio|ABGYD853-06|ROM 109280|Guyana.Potaro-Siparuni
Ametrida centurio|ABGYC569-06|ROM 107415|Guyana.Potaro-Siparuni
Ametrida centurio|ABGYC235-06|ROM 106785|Guyana.Upper Takutu-Upper Essequibo
Ametrida centurio|ABGYD149-06|ROM 108455|Guyana.Potaro-Siparuni
Ametrida centurio|ABGYE117-06|ROM 111560|Guyana.Potaro-Siparuni
Ametrida centurio|ABSRA345-06|ROM 116934|Suriname.Nickerie
Ametrida centurio|BCBN361-05|ROM 101098|Guyana.Barima-Waini
Ametrida centurio|BCBNT309-06|ROM 108624|Guyana.Potaro-Siparuni
Ametrida centurio|ABGYC087-06|ROM 106591|Guyana.Upper Takutu-Upper Essequibo
Ametrida centurio|ABGYA135-06|ROM 97977|Guyana.Potaro-Siparuni
Ametrida centurio|BCBNT316-06|ROM 108730|Guyana.Potaro-Siparuni
Ametrida centurio|ABGYD533-06|ROM 108892|Guyana.Potaro-Siparuni
Ametrida centurio|ABGYG767-08|ROM 119195|Guyana
Ametrida centurio|ABGYE315-06|ROM 111765|Guyana.Potaro-Siparuni
Ametrida centurio|ABGYB097-06|ROM 101074|Guyana.Barima-Waini
Ametrida centurio|ABGYE421-06|ROM 111879|Guyana.Potaro-Siparuni
Ametrida centurio|ABGYG579-06|ROM 116741|Guyana.Potaro-Siparuni
Ametrida centurio|ABGYD653-06|ROM 109061|Guyana.Potaro-Siparuni
Ametrida centurio|ABGYD403-06|ROM 108749|Guyana.Potaro-Siparuni
Ametrida centurio|ABGYE005-06|ROM 109286|Guyana.Potaro-Siparuni
Ametrida centurio|ABGYD722-06|ROM 109137|Guyana.Potaro-Siparuni
Ametrida centurio|ABGYB439-06|ROM 103153|Guyana.Upper Takutu-Upper Essequibo
Ametrida centurio|ABGYC655-06|ROM 107474|Guyana.Potaro-Siparuni
Ametrida centurio|ABGYD534-06|ROM 108893|Guyana.Potaro-Siparuni
Ametrida centurio|ABGYB304-06|ROM 103009|Guyana.Upper Takutu-Upper Essequibo
Ametrida centurio|ABGYB566-06|ROM 103302|Guyana.Upper Takutu-Upper Essequibo
Ametrida centurio|ABGYA146-06|ROM 97991|Guyana.Potaro-Siparuni
Ametrida centurio|ABGYA853-06|ROM 100923|Guyana.Barima-Waini
Ametrida centurio|ABGYE427-06|ROM 111885|Guyana.Potaro-Siparuni
Ametrida centurio|ABGYG377-06|ROM 116500|Guyana.Potaro-Siparuni
Ametrida centurio|ABGYD402-06|ROM 108748|Guyana.Potaro-Siparuni
Ametrida centurio|ABGYD820-06|ROM 109243|Guyana.Potaro-Siparuni
Ametrida centurio|ABGYE116-06|ROM 111559|Guyana.Potaro-Siparuni
Ametrida centurio|ABGYG559-06|ROM 116709|Guyana.Potaro-Siparuni
Ametrida centurio|ABSRA497-06|ROM 117087|Suriname.Nickerie
Ametrida centurio|ABGYE205-06|ROM 111652|Guyana.Potaro-Siparuni
Ametrida centurio|ABGYD404-06|ROM 108750|Guyana.Potaro-Siparuni
Ametrida centurio|ABGYG454-06|ROM 116591|Guyana.Potaro-Siparuni
Ametrida centurio|ABSRA520-06|ROM 117110|Suriname.Nickerie
Ametrida centurio|ABSRA515-06|ROM 117105|Suriname.Nickerie
Ametrida centurio|ABGYD826-06|ROM 109249|Guyana.Potaro-Siparuni
Ametrida centurio|ABGYD828-06|ROM 109251|Guyana.Potaro-Siparuni
Ametrida centurio|ABGYD654-06|ROM 109062|Guyana.Potaro-Siparuni
Ametrida centurio|ABGYD829-06|ROM 109252|Guyana.Potaro-Siparuni
Ametrida centurio|ABGYD852-06|ROM 109279|Guyana.Potaro-Siparuni
Ametrida centurio|BCBN170-05|ROM 98849|Guyana.Barima-Waini
Ametrida centurio|ABGYD401-06|ROM 108747|Guyana.Potaro-Siparuni
Ametrida centurio|BCBNT793-06|ROM 114056|Suriname.Brokopondo
Ectophylla alba|BCBNT416-06|ROM 108296|Costa Rica.Limon
Enchisthenes hartii|ABSCA078-06|ROM F38136|Panama.Chiriqui
Enchisthenes hartii|BCBN665-05|ROM 104286|Panama.Chiriqui
Enchisthenes hartii|BCBN666-05|ROM 104287|Panama.Chiriqui
Tonatia saurophila|ABECA198-06|ROM 104519|Ecuador.Napo
Tonatia saurophila|ABECA792-06|ROM 105956|Ecuador.Napo
Tonatia saurophila|ABECA804-06|ROM 105970|Ecuador.Napo
Tonatia saurophila|BCBN747-05|ROM 104461|Ecuador.Napo
Tonatia saurophila|BCBN559-05|ROM 103986|Ecuador.Napo
Tonatia saurophila|ABECA004-06|ROM 103985|Ecuador.Napo
Tonatia saurophila|ABECA010-06|ROM F37074|Ecuador.Napo
Tonatia saurophila|ABECA154-06|ROM F37297|Ecuador.Napo
Tonatia saurophila|ABECA155-06|ROM 104460|Ecuador.Napo
Tonatia saurophila|ABECA335-06|ROM 105220|Ecuador.Napo
Tonatia saurophila|ABECB074-08|ROM 118736|Ecuador
Tonatia saurophila|BCBN746-05|ROM 104459|Ecuador.Napo
Tonatia saurophila|BCBN816-05|ROM 105299|Ecuador.Napo
Tonatia saurophila|BCBN950-05|ROM 105914|Ecuador.Napo
Tonatia saurophila|BCBNT562-06|ROM 112583|Guyana.Demerara-Mahaica
Tonatia saurophila|ABGYC472-06|ROM 107305|Guyana.Potaro-Siparuni
Tonatia saurophila|ABSRA424-06|ROM 117013|Suriname.Nickerie
Tonatia saurophila|ABSMS177-06|ROM 117339|Suriname.Sipaliwini
Tonatia saurophila|ABSMS181-06|ROM 117343|Suriname.Sipaliwini
Tonatia saurophila|ABSRA487-06|ROM 117077|Suriname.Nickerie
Tonatia saurophila|ABGYC244-06|ROM 107024|Guyana.Potaro-Siparuni
Tonatia saurophila|ABGYC291-06|ROM 107090|Guyana.Potaro-Siparuni
Tonatia saurophila|ABGYD802-06|ROM 109225|Guyana.Potaro-Siparuni
Tonatia saurophila|ABSMS289-06|ROM 117451|Suriname.Sipaliwini
Tonatia saurophila|BCBNT747-06|ROM 113930|Suriname.Brokopondo
Tonatia saurophila|BCBNT724-06|ROM 113895|Suriname.Brokopondo
Tonatia saurophila|BCBNT121-06|ROM 107287|Guyana.Potaro-Siparuni
Tonatia saurophila|BCBNT120-06|ROM 107286|Guyana.Potaro-Siparuni

Tonatia saurophila|BCBNT724-06|ROM 113895|Suriname.Brokopondo
Tonatia saurophila|BCBNT121-06|ROM 107287|Guyana.Potaro-Siparuni
Tonatia saurophila|BCBNT120-06|ROM 107286|Guyana.Potaro-Siparuni
Tonatia saurophila|BCBNT119-06|ROM 107285|Guyana.Potaro-Siparuni
Tonatia saurophila|BCBNT118-06|ROM 107284|Guyana.Potaro-Siparuni
Tonatia saurophila|ABSMS336-06|ROM 117498|Suriname.Sipaliwini
Tonatia saurophila|ABSMS137-06|ROM 117299|Suriname.Sipaliwini
Tonatia saurophila|ABSMS031-06|ROM 117193|Suriname.Sipaliwini
Tonatia saurophila|ABRMM085-07|ROM F39711|Guyana.Potaro-Siparuni
Tonatia saurophila|ABGYG1043-08|ROM 118967|Guyana
Tonatia saurophila|ABGYE785-06|ROM 113385|Guyana.Upper Demerara-Berbice
Tonatia saurophila|ABGYE400-06|ROM 111852|Guyana.Potaro-Siparuni
Tonatia saurophila|ABGYE149-06|ROM 111592|Guyana.Potaro-Siparuni
Tonatia saurophila|ABGYD578-06|ROM 108957|Guyana.Potaro-Siparuni
Tonatia saurophila|ABGYD113-06|ROM 108411|Guyana.Potaro-Siparuni
Tonatia saurophila|ABGYD081-06|ROM 108378|Guyana.Potaro-Siparuni
Tonatia saurophila|ABGYC601-06|ROM 107457|Guyana.Potaro-Siparuni
Tonatia saurophila|ABGYC589-06|ROM 107444|Guyana.Potaro-Siparuni
Tonatia saurophila|ABGYC546-06|ROM 107385|Guyana.Potaro-Siparuni
Tonatia saurophila|ABGYC529-06|ROM 107367|Guyana.Potaro-Siparuni
Tonatia saurophila|ABGYC514-06|ROM 107350|Guyana.Potaro-Siparuni
Tonatia saurophila|ABGYC447-06|ROM 107272|Guyana.Potaro-Siparuni
Tonatia saurophila|ABGYC417-06|ROM 107239|Guyana.Potaro-Siparuni
Tonatia saurophila|ABGYC413-06|ROM 107235|Guyana.Potaro-Siparuni
Tonatia saurophila|ABGYC402-06|ROM 107221|Guyana.Potaro-Siparuni
Tonatia saurophila|ABGYC383-06|ROM 107200|Guyana.Potaro-Siparuni
Tonatia saurophila|ABGYC358-06|ROM 107173|Guyana.Potaro-Siparuni
Tonatia saurophila|ABGYC322-06|ROM 107134|Guyana.Potaro-Siparuni
Tonatia saurophila|ABGYC045-06|ROM 104822|Guyana.Potaro-Siparuni
Tonatia saurophila|ABGYB657-06|ROM 103414|Guyana.Upper Demerara-Berbice
Tonatia saurophila|ABGYB644-06|ROM 103401|Guyana.Upper Demerara-Berbice
Tonatia saurophila|ABGYB475-06|ROM 103210|Guyana.Upper Takutu-Upper Essequibo
Tonatia saurophila|ABSMS146-06|ROM 117308|Suriname.Sipaliwini
Tonatia saurophila|ABGYC600-06|ROM 107456|Guyana.Potaro-Siparuni
Tonatia saurophila|ABGYC565-06|ROM 107410|Guyana.Potaro-Siparuni
Tonatia saurophila|ABGYG179-06|ROM 115743|Guyana.Potaro-Siparuni
Tonatia saurophila|ABGYD082-06|ROM 108379|Guyana.Potaro-Siparuni
Tonatia saurophila|BCBNT791-06|ROM 114051|Suriname.Brokopondo
Tonatia saurophila|BCBNT795-06|ROM 114062|Suriname.Brokopondo
Uroderma magnirostrum|ABGYG763-08|ROM 119191|Guyana
Uroderma bilobatum|ABSCA112-06|ROM 104350|Panama.Darien
Uroderma bilobatum|ABSCA113-06|ROM 104351|Panama.Darien
Uroderma bilobatum|BCBN590-05|ROM 104050|Ecuador.Napo
Uroderma bilobatum|BCBN629-05|ROM 104234|Panama
Uroderma bilobatum|BCBN711-05|ROM 104368|Panama.Darien
Uroderma bilobatum|ABSCA136-06|ROM F44037|Costa Rica.Limon
Uroderma bilobatum|ABSCA035-06|ROM F38032|Panama.Canal Zone
Uroderma bilobatum|BCBN608-05|ROM 104203|Panama.Canal Zone
Uroderma bilobatum|BCBNT431-06|ROM 108309|Costa Rica.Limon
Uroderma bilobatum|BCBN628-05|ROM 104233|Panama
Uroderma bilobatum|ABSCA051-06|ROM 104235|Panama
Uroderma bilobatum|BCBNT441-06|ROM 108319|Costa Rica.Limon
Uroderma bilobatum|BCBNT442-06|ROM 108320|Costa Rica.Limon
Uroderma bilobatum|ABGYC133-06|ROM 106645|Guyana.Upper Takutu-Upper Essequibo
Uroderma bilobatum|ABECA360-06|ROM 105252|Ecuador.Napo
Uroderma bilobatum|ABGYG549-06|ROM 116695|Guyana.Potaro-Siparuni
Uroderma bilobatum|ABGYB175-06|ROM 101166|Guyana.Barima-Waini
Uroderma bilobatum|ABGYB380-06|ROM 103087|Guyana.Upper Takutu-Upper Essequibo
Uroderma bilobatum|ABGYD317-06|ROM 108656|Guyana.Potaro-Siparuni
Uroderma bilobatum|ABGYD491-06|ROM 108845|Guyana.Potaro-Siparuni
Uroderma bilobatum|ABGYA191-06|ROM 98059|Guyana.Potaro-Siparuni
Uroderma bilobatum|ABGYD137-06|ROM 108440|Guyana.Potaro-Siparuni
Uroderma bilobatum|ABGYG764-08|ROM 119192|Guyana
Uroderma bilobatum|ABECB023-06|ROM 106338|Ecuador.Napo
Uroderma bilobatum|ABECA361-06|ROM F37574|Ecuador.Napo
Uroderma bilobatum|ABECB024-06|ROM 106339|Ecuador.Napo
Uroderma bilobatum|ABGYG848-08|ROM 119276|Guyana
Uroderma bilobatum|ABGYB411-06|ROM 103118|Guyana.Upper Takutu-Upper Essequibo
Uroderma bilobatum|ABGYA715-06|ROM 100420|Guyana.East Berbice-Corentyne
Uroderma bilobatum|ABGYC741-06|ROM 108142|Guyana.Cuyuni-Mazaruni
Uroderma bilobatum|ABGYC365-06|ROM 107181|Guyana.Potaro-Siparuni
Uroderma bilobatum|ABGYF297-06|ROM 114665|Guyana.Potaro-Siparuni
Uroderma bilobatum|ABGYA491-06|ROM 98901|Guyana.Barima-Waini
Uroderma bilobatum|ABGYB291-06|ROM 102995|Guyana.Upper Takutu-Upper Essequibo
Uroderma bilobatum|ABGYD049-06|ROM 108272|Guyana.Cuyuni-Mazaruni
Uroderma bilobatum|ABGYB143-06|ROM 101130|Guyana.Barima-Waini
Uroderma bilobatum|ABGYB144-06|ROM 101131|Guyana.Barima-Waini
Uroderma bilobatum|BCBN354-05|ROM 101030|Guyana.Barima-Waini
Uroderma bilobatum|ABECA574-06|ROM 105604|Ecuador.Napo
Uroderma bilobatum|ABGYB148-06|ROM 101135|Guyana.Barima-Waini
Uroderma bilobatum|ABGYB161-06|ROM 101150|Guyana.Barima-Waini
Uroderma bilobatum|ABGYA333-06|ROM 98731|Guyana.Barima-Waini
Uroderma bilobatum|ABGYB162-06|ROM 101151|Guyana.Barima-Waini
Uroderma bilobatum|ABGYF120-06|ROM 113666|Guyana.Demerara-Mahaica
Uroderma bilobatum|ABGYD372-06|ROM 108716|Guyana.Potaro-Siparuni
Uroderma bilobatum|ABGYA852-06|ROM 100920|Guyana.Barima-Waini
Uroderma bilobatum|ABGYB412-06|ROM 103119|Guyana.Upper Takutu-Upper Essequibo
Uroderma bilobatum|ABGYB593-06|ROM 103334|Guyana.Upper Takutu-Upper Essequibo
Uroderma bilobatum|BCBN359-05|ROM 101094|Guyana.Barima-Waini
Uroderma bilobatum|ABGYB294-06|ROM 102998|Guyana.Upper Takutu-Upper Essequibo
Uroderma bilobatum|ABGYB375-06|ROM 103082|Guyana.Upper Takutu-Upper Essequibo
Uroderma bilobatum|ABECA700-06|ROM 105760|Ecuador.Napo
Uroderma bilobatum|ABGYB489-06|ROM 103224|Guyana.Upper Takutu-Upper Essequibo
Uroderma bilobatum|ABGYF199-06|ROM 113769|Guyana.Demerara-Mahaica
Uroderma bilobatum|ABGYB031-06|ROM 101003|Guyana.Barima-Waini
Uroderma bilobatum|ABGYA190-06|ROM 98058|Guyana.Potaro-Siparuni
Uroderma bilobatum|ABGYB492-06|ROM 103227|Guyana.Upper Takutu-Upper Essequibo
Uroderma bilobatum|ABGYA048-06|ROM 97847|Guyana.Upper Takutu-Upper Essequibo
Uroderma bilobatum|ABGYC107-06|ROM 106617|Guyana.Upper Takutu-Upper Essequibo
Uroderma bilobatum|ABGYD194-06|ROM 108521|Guyana.Potaro-Siparuni
Uroderma bilobatum|ABGYB490-06|ROM 103225|Guyana.Upper Takutu-Upper Essequibo
Uroderma bilobatum|ABGYB226-06|ROM 102928|Guyana.Upper Takutu-Upper Essequibo
Uroderma bilobatum|ABGYE269-06|ROM 111718|Guyana.Potaro-Siparuni

[illegible]
Uroderma bilobatum|ABGYB490-06|ROM 103225|Guyana.Upper Takutu-Upper Essequibo
Uroderma bilobatum|ABGYB226-06|ROM 102928|Guyana.Upper Takutu-Upper Essequibo
Uroderma bilobatum|ABGYE269-06|ROM 111718|Guyana.Potaro-Siparuni
Uroderma bilobatum|ABGYC599-06|ROM 107455|Guyana.Potaro-Siparuni
Uroderma bilobatum|ABGYB332-06|ROM 103039|Guyana.Upper Takutu-Upper Essequibo
Uroderma bilobatum|ABSMS485-06|ROM 117647|Suriname.Sipaliwini
Uroderma bilobatum|ABSMS482-06|ROM 117644|Suriname.Sipaliwini
Uroderma bilobatum|ABSMS466-06|ROM 117628|Suriname.Sipaliwini
Uroderma bilobatum|ABGYG572-06|ROM 116733|Guyana.Potaro-Siparuni
Uroderma bilobatum|ABGYG500-06|ROM 116642|Guyana.Potaro-Siparuni
Uroderma bilobatum|ABGYC757-06|ROM 108160|Guyana.Cuyuni-Mazaruni
Uroderma bilobatum|ABGYB292-06|ROM 102996|Guyana.Upper Takutu-Upper Essequibo
Uroderma bilobatum|ABGYA492-06|ROM 98902|Guyana.Barima-Waini
Uroderma bilobatum|ABGYD440-06|ROM 108791|Guyana.Potaro-Siparuni
Uroderma bilobatum|ABSMS262-06|ROM 117424|Suriname.Sipaliwini
Uroderma bilobatum|BCBN340-05|ROM 100936|Guyana.Barima-Waini
Uroderma bilobatum|ABGYE314-06|ROM 111764|Guyana.Potaro-Siparuni
Uroderma bilobatum|ABSMS288-06|ROM 117450|Suriname.Sipaliwini
Uroderma bilobatum|ABGYB491-06|ROM 103226|Guyana.Upper Takutu-Upper Essequibo
Uroderma bilobatum|ABECA511-06|ROM F37780|Ecuador.Napo
Uroderma bilobatum|ABGYF067-06|ROM 113589|Guyana.Upper Takutu-Upper Essequibo
Uroderma bilobatum|BCBN346-05|ROM 100965|Guyana.Barima-Waini
Uroderma bilobatum|ABGYB493-06|ROM 103228|Guyana.Upper Takutu-Upper Essequibo
Uroderma bilobatum|ABGYD473-06|ROM 108824|Guyana.Potaro-Siparuni
Uroderma bilobatum|ABGYE313-06|ROM 111763|Guyana.Potaro-Siparuni
Uroderma bilobatum|ABGYA462-06|ROM 98872|Guyana.Barima-Waini
Uroderma bilobatum|ABGYC001-06|ROM 104778|Guyana.Potaro-Siparuni
Uroderma bilobatum|ABECA935-06|ROM 106118|Ecuador.Napo
Uroderma bilobatum|ABECA054-06|ROM F37141|Ecuador.Napo
Uroderma bilobatum|ABECA772-06|ROM 105930|Ecuador.Napo
Uroderma bilobatum|ABECA358-06|ROM 105250|Ecuador.Napo
Uroderma bilobatum|ABECA506-06|ROM 105541|Ecuador.Napo
Uroderma bilobatum|ABECA334-06|ROM 105219|Ecuador.Napo
Uroderma bilobatum|ABECA169-06|ROM 104482|Ecuador.Napo
Uroderma bilobatum|ABECA636-06|ROM 105684|Ecuador.Napo
Uroderma bilobatum|ABECA803-06|ROM 105969|Ecuador.Napo
Uroderma bilobatum|ABECA934-06|ROM 106117|Ecuador.Napo
Uroderma bilobatum|ABECB022-06|ROM F40498|Ecuador.Napo
Uroderma bilobatum|ABECB026-06|ROM 106341|Ecuador.Napo
Uroderma bilobatum|ABGYE461-06|ROM 111928|Guyana.Potaro-Siparuni
Uroderma bilobatum|ABECB004-06|ROM F40476|Ecuador.Napo
Uroderma bilobatum|ABGYA306-06|ROM 98702|Guyana.Barima-Waini
Uroderma bilobatum|ABGYF240-06|ROM 113820|Guyana.Demerara-Mahaica
Uroderma bilobatum|ABSRA426-06|ROM 117015|Suriname.Nickerie
Uroderma bilobatum|BCBN575-05|ROM 104027|Ecuador.Napo
Uroderma bilobatum|ABGYB518-06|ROM 103253|Guyana.Upper Takutu-Upper Essequibo
Uroderma bilobatum|ABECA359-06|ROM 105251|Ecuador.Napo
Uroderma bilobatum|ABECA324-06|ROM 105205|Ecuador.Napo
Uroderma bilobatum|ABECB025-06|ROM 106340|Ecuador.Napo
Uroderma bilobatum|ABGYB163-06|ROM 101152|Guyana.Barima-Waini
Uroderma bilobatum|ABGYB189-06|ROM 102891|Guyana.Upper Takutu-Upper Essequibo
Uroderma bilobatum|ABGYB468-06|ROM 103202|Guyana.Upper Takutu-Upper Essequibo
Uroderma bilobatum|BCBN794-05|ROM 105204|Ecuador.Napo
Uroderma bilobatum|ABGYF119-06|ROM 113665|Guyana.Demerara-Mahaica
Uroderma bilobatum|ABECA055-06|ROM F37142|Ecuador.Napo
Uroderma bilobatum|ABECA936-06|ROM 106119|Ecuador.Napo
Uroderma bilobatum|ABECA937-06|ROM 106120|Ecuador.Napo
Uroderma bilobatum|ABECA616-06|ROM 105655|Ecuador.Napo
Uroderma bilobatum|ABECA643-06|ROM 105698|Ecuador.Napo
Uroderma bilobatum|ABECB021-06|ROM F40497|Ecuador.Napo
Uroderma bilobatum|BCBN576-05|ROM 104028|Ecuador.Napo
Uroderma bilobatum|ABECA605-06|ROM 105640|Ecuador.Napo
Uroderma bilobatum|ABECA654-06|ROM 105712|Ecuador.Napo
Uroderma bilobatum|ABECA041-06|ROM 104023|Ecuador.Napo
Uroderma bilobatum|BCBN979-05|ROM 106073|Ecuador.Napo
Uroderma bilobatum|ABSMS441-06|ROM 117603|Suriname.Sipaliwini
Uroderma bilobatum|ABGYD171-06|ROM 108491|Guyana.Potaro-Siparuni
Uroderma bilobatum|ABGYD431-06|ROM 108782|Guyana.Potaro-Siparuni
Uroderma bilobatum|BCBNT741-06|ROM 113920|Suriname.Brokopondo
Uroderma bilobatum|ABGYB703-06|ROM 103467|Guyana.Upper Demerara-Berbice
Uroderma bilobatum|ABGYD134-06|ROM 108437|Guyana.Potaro-Siparuni
Uroderma bilobatum|ABGYD316-06|ROM 108655|Guyana.Potaro-Siparuni
Uroderma bilobatum|ABGYA167-06|ROM 98026|Guyana.Potaro-Siparuni
Uroderma bilobatum|ABGYB563-06|ROM 103299|Guyana.Upper Takutu-Upper Essequibo
Uroderma bilobatum|BCBNT769-06|ROM 113987|Suriname.Brokopondo
Vampyressa thyone|ABSCA094-06|ROM 104322|Panama.Chiriqui
Vampyressa thyone|BCBN680-05|ROM 104306|Panama.Chiriqui
Vampyressa thyone|BCBN692-05|ROM 104332|Panama.Chiriqui
Vampyressa thyone|ABECA313-06|ROM 105190|Ecuador.Napo
Vampyressa thyone|ABECA336-06|ROM 105222|Ecuador.Napo
Vampyressa thyone|ABECB014-06|ROM F40488|Ecuador.Napo
Vampyressa thyone|ABECA102-06|ROM 104391|Ecuador.Napo
Vampyressa thyone|ABECA218-06|ROM 104544|Ecuador.Napo
Vampyressa thyone|ABECA291-06|ROM F37475|Ecuador.Napo
Vampyressa thyone|ABECA341-06|ROM 105228|Ecuador.Napo
Vampyressa thyone|ABECA555-06|ROM F37827|Ecuador.Napo
Vampyressa thyone|ABECA736-06|ROM 105882|Ecuador.Napo
Vampyressa thyone|ABECB121-08|ROM 118783|Ecuador
Vampyressa thyone|ABECA292-06|ROM 105161|Ecuador.Napo
Vampyressa thyone|ABECA339-06|ROM 105226|Ecuador.Napo
Vampyressa thyone|BCBN762-05|ROM 104538|Ecuador.Napo
Vampyressa thyone|ABECA333-06|ROM 105218|Ecuador.Napo
Vampyressa thyone|BCBN890-05|ROM 105714|Ecuador.Napo
Vampyressa thyone|ABECA180-06|ROM 104493|Ecuador.Napo
Vampyressa thyone|ABECA655-06|ROM 105716|Ecuador.Napo
Vampyressa thyone|ABECA227-06|ROM 104550|Ecuador.Napo
Vampyressa thyone|ABECA340-06|ROM 105227|Ecuador.Napo
Vampyressa thyone|ABECA121-06|ROM 104414|Ecuador.Napo
Vampyressa thyone|ABECA221-06|ROM 104546|Ecuador.Napo
Vampyressa thyone|ABECA827-06|ROM 105996|Ecuador.Napo
Vampyressa thyone|ABECA229-06|ROM 104554|Ecuador.Napo
Vampyressa thyone|ABECA826-06|ROM 105995|Ecuador.Napo
Vampyressa thyone|ABECA213-06|ROM 104535|Ecuador.Napo

[illegible]
Vampyressa thyone|ABECA229-06|ROM 104554|Ecuador.Napo
Vampyressa thyone|ABECA826-06|ROM 105995|Ecuador.Napo
Vampyressa thyone|ABECA213-06|ROM 104535|Ecuador.Napo
Vampyressa thyone|ABECA214-06|ROM 104536|Ecuador.Napo
Vampyressa thyone|BCBN891-05|ROM 105715|Ecuador.Napo
Vampyressa thyone|ABECA222-06|ROM 104547|Ecuador.Napo
Vampyressa thyone|BCBN631-05|ROM 104238|Panama
Vampyressa thyone|BCBN889-05|ROM 105713|Ecuador.Napo
Vampyressa thyone|ABECB015-06|ROM F40489|Ecuador.Napo
Vampyressa thyone|ABSCA124-06|ROM 104367|Panama.Darien
Vampyressa thyone|BCBN632-05|ROM 104239|Panama
Vampyressa thyone|ABSCA123-06|ROM 104366|Panama.Darien
Vampyressa thyone|BCBN929-05|ROM 105806|Ecuador.Esmeraldas
Vampyressa thyone|ABSCA126-06|ROM 104370|Panama.Darien
Vampyressa thyone|ABECA896-06|ROM 106076|Ecuador.Napo
Vampyressa thyone|ABECB016-06|ROM 106333|Ecuador.Napo
Vampyressa thyone|ABSCA054-06|ROM 104237|Panama
Vampyressa thyone|ABECA346-06|ROM 105233|Ecuador.Napo
Vampyressa thyone|ABSCA053-06|ROM F38074|Panama
Vampyressa thyone|BCBN699-05|ROM 104345|Panama.Darien
Vampyressa thyone|BCBNT001-06|ROM 106332|Ecuador.Napo
Vampyressa thyone|ABECA149-06|ROM 104453|Ecuador.Napo
Vampyressa thyone|ABECA216-06|ROM 104539|Ecuador.Napo
Vampyressa thyone|ABECA220-06|ROM F37394|Ecuador.Napo
Vampyressa thyone|ABECB057-06|ROM 106357|Ecuador.Napo
Vampyressa thyone|BCBNT237-06|ROM 108145|Guyana.Cuyuni-Mazaruni
Vampyressa thyone|BCBNT308-06|ROM 108622|Guyana.Potaro-Siparuni
Vampyressa pusilla|ABSA062-06|ROM 111076|Brazil.Sao Paulo
Vampyressa pusilla|BCBNT479-06|ROM 111095|Brazil.Sao Paulo
Vampyressa pusilla|BCBNT470-06|ROM 111071|Brazil.Sao Paulo
Vampyressa pusilla|ABSA069-06|ROM 111087|Brazil.Sao Paulo
Vampyressa pusilla|ABSA106-06|ROM 111146|Brazil.Sao Paulo
Vampyressa pusilla|BCBNT476-06|ROM 111083|Brazil.Sao Paulo
Vampyressa pusilla|BCBNT494-06|ROM 111130|Brazil.Sao Paulo
Vampyressa nymphaea|BCBNT407-06|ROM 108287|Costa Rica.Limon
Vampyressa nymphaea|BCBNT408-06|ROM 108288|Costa Rica.Limon
Vampyressa nymphaea|ABSCA052-06|ROM F38073|Panama
Vampyressa nymphaea|BCBN630-05|ROM 104236|Panama
Vampyressa nymphaea|BCBN653-05|ROM 104270|Panama
Vampyressa nymphaea|ABSCA131-06|ROM F44012|Costa Rica.Limon
Vampyressa nymphaea|BCBN697-05|ROM 104343|Panama.Darien
Vampyressa nymphaea|BCBNT444-06|ROM 108322|Costa Rica.Limon
Vampyressa brocki|ABSMS202-06|ROM 117364|Suriname.Sipaliwini
Vampyressa brocki|BCBNT545-06|ROM 112095|Guyana.Potaro-Siparuni
Vampyressa brocki|BCBNT544-06|ROM 112094|Guyana.Potaro-Siparuni
Vampyressa brocki|BCBNT328-06|ROM 108850|Guyana.Potaro-Siparuni
Vampyressa brocki|ABGYC618-06|ROM F39219|Guyana.Potaro-Siparuni
Vampyressa brocki|BCBNT533-06|ROM 111935|Guyana.Potaro-Siparuni
Vampyressa brocki|BCBNT648-06|ROM 113614|Guyana.Demerara-Mahaica
Vampyressa brocki|BCBNT858-06|ROM 114235|Suriname.Brokopondo
Vampyressa brocki|BCBNT933-06|ROM 115624|Guyana.Essequibo Islands-West Demerara
Vampyressa bidens|ABECA185-06|ROM 104503|Ecuador.Napo
Vampyressa bidens|ABGYD835-06|ROM 109259|Guyana.Potaro-Siparuni
Vampyressa bidens|ABGYE221-06|ROM 111669|Guyana.Potaro-Siparuni
Vampyressa bidens|ABECA186-06|ROM F37349|Ecuador.Napo
Vampyressa bidens|ABECA014-06|ROM 103995|Ecuador.Napo
Vampyressa bidens|ABECA223-06|ROM F37398|Ecuador.Napo
Vampyressa bidens|ABECA013-06|ROM 103994|Ecuador.Napo
Vampyressa bidens|ABECA771-06|ROM 105929|Ecuador.Napo
Vampyressa bidens|ABECA071-06|ROM 104065|Ecuador.Napo
Vampyressa bidens|ABECA894-06|ROM 106074|Ecuador.Napo
Vampyressa bidens|ABECB019-06|ROM F40495|Ecuador.Napo
Vampyressa bidens|BCBN766-05|ROM 104548|Ecuador.Napo
Vampyressa bidens|BCBNT003-06|ROM 106335|Ecuador.Napo
Vampyressa bidens|ABECA265-06|ROM 105133|Ecuador.Napo
Vampyressa bidens|ABECA165-06|ROM 104479|Ecuador.Napo
Vampyressa bidens|ABECA212-06|ROM 104534|Ecuador.Napo
Vampyressa bidens|ABECA275-06|ROM F37454|Ecuador.Napo
Vampyressa bidens|ABECA480-06|ROM 105505|Ecuador.Napo
Vampyressa bidens|ABGYB739-06|ROM 103506|Guyana.Upper Demerara-Berbice
Vampyressa bidens|BCBN893-05|ROM 105722|Ecuador.Napo
Vampyressa bidens|ABECA661-06|ROM 105724|Ecuador.Napo
Vampyressa bidens|ABECA699-06|ROM 105759|Ecuador.Napo
Vampyressa bidens|ABECA267-06|ROM 105135|Ecuador.Napo
Vampyressa bidens|ABECA796-06|ROM 105961|Ecuador.Napo
Vampyressa bidens|ABECA293-06|ROM 105162|Ecuador.Napo
Vampyressa bidens|ABECA580-06|ROM 105612|Ecuador.Napo
Vampyressa bidens|ABECA849-06|ROM 106024|Ecuador.Napo
Vampyressa bidens|BCBN767-05|ROM 104551|Ecuador.Napo
Vampyressa bidens|ABECB018-06|ROM 106336|Ecuador.Napo
Vampyressa bidens|ABECA258-06|ROM 105128|Ecuador.Napo
Vampyressa bidens|ABECB040-06|ROM F40517|Ecuador.Napo
Vampyressa bidens|BCBN894-05|ROM 105723|Ecuador.Napo
Vampyressa bidens|ABECA266-06|ROM 105134|Ecuador.Napo
Vampyressa bidens|ABECA352-06|ROM 105242|Ecuador.Napo
Vampyressa bidens|ABGYE468-06|ROM 111936|Guyana.Potaro-Siparuni
Vampyressa bidens|ABGYE423-06|ROM 111881|Guyana.Potaro-Siparuni
Vampyressa bidens|ABGYG554-06|ROM 116700|Guyana.Potaro-Siparuni
Vampyressa bidens|ABGYG034-06|ROM 115540|Guyana.Essequibo Islands-West Demerara
Vampyressa bidens|BCBNT932-06|ROM 115623|Guyana.Essequibo Islands-West Demerara
Vampyressa bidens|ABGYC545-06|ROM 107384|Guyana.Potaro-Siparuni
Vampyressa bidens|ABGYC597-06|ROM 107453|Guyana.Potaro-Siparuni
Vampyressa bidens|ABGYD191-06|ROM 108518|Guyana.Potaro-Siparuni
Vampyressa bidens|ABGYD361-06|ROM 108704|Guyana.Potaro-Siparuni
Vampyressa bidens|ABGYD846-06|ROM 109273|Guyana.Potaro-Siparuni
Vampyressa bidens|ABGYE412-06|ROM 111866|Guyana.Potaro-Siparuni
Vampyressa bidens|ABRMM158-07|ROM F41904|Ecuador
Vampyressa bidens|ABGYC424-06|ROM 107246|Guyana.Potaro-Siparuni
Vampyressa bidens|ABGYD356-06|ROM 108698|Guyana.Potaro-Siparuni
Vampyressa bidens|ABGYG514-06|ROM 116656|Guyana.Potaro-Siparuni
Vampyressa bidens|ABGYD442-06|ROM 108793|Guyana.Potaro-Siparuni
Vampyressa bidens|ABECA298-06|ROM 105169|Ecuador.Napo
[illegible]

Vampyressa bidens|ABGYG514-06|ROM 116656|Guyana.Potaro-Siparuni
Vampyressa bidens|ABGYD442-06|ROM 108793|Guyana.Potaro-Siparuni
Vampyressa bidens|ABECA298-06|ROM 105169|Ecuador.Napo
Vampyressa bidens|ABECA660-06|ROM F37972|Ecuador.Napo
Vampyressa bidens|ABGYD023-06|ROM 108243|Guyana.Cuyuni-Mazaruni
Vampyressa bidens|ABGYD193-06|ROM 108520|Guyana.Potaro-Siparuni
Vampyressa bidens|ABGYD213-06|ROM 108544|Guyana.Potaro-Siparuni
Vampyressa bidens|ABGYD836-06|ROM 109260|Guyana.Potaro-Siparuni
Vampyressa bidens|ABGYE422-06|ROM 111880|Guyana.Potaro-Siparuni
Vampyressa bidens|ABGYB832-06|ROM 104673|Guyana.Potaro-Siparuni
Vampyressa bidens|ABGYF066-06|ROM 113588|Guyana.Upper Takutu-Upper Essequibo
Vampyressa bidens|ABGYC264-06|ROM 107053|Guyana.Potaro-Siparuni
Vampyressa bidens|ABGYE046-06|ROM 109332|Guyana.Potaro-Siparuni
Vampyressa bidens|ABGYD190-06|ROM 108517|Guyana.Potaro-Siparuni
Vampyressa bidens|ABGYG513-06|ROM 116655|Guyana.Potaro-Siparuni
Vampyressa bidens|ABGYE428-06|ROM 111886|Guyana.Potaro-Siparuni
Vampyressa bidens|ABGYC591-06|ROM 107447|Guyana.Potaro-Siparuni
Vampyressa bidens|ABSMS415-06|ROM 117577|Suriname.Sipaliwini
Vampyressa bidens|ABGYC088-06|ROM 106592|Guyana.Upper Takutu-Upper Essequibo
Vampyressa bidens|ABGYC742-06|ROM 108143|Guyana.Cuyuni-Mazaruni
Vampyressa bidens|ABGYC807-06|ROM 108205|Guyana.Cuyuni-Mazaruni
Vampyressa bidens|ABGYE227-06|ROM 111675|Guyana.Potaro-Siparuni
Vampyressa bidens|ABRMM062-07|ROM F39001|Guyana.Potaro-Siparuni
Vampyressa bidens|ABGYB564-06|ROM 103300|Guyana.Upper Takutu-Upper Essequibo
Vampyressa bidens|BCBNT887-06|ROM 115516|Guyana.Essequibo Islands-West Demerara
Vampyressa bidens|ABGYD250-06|ROM 108585|Guyana.Potaro-Siparuni
Vampyressa bidens|ABGYG035-06|ROM 115541|Guyana.Essequibo Islands-West Demerara
Vampyressa bidens|ABGYE475-06|ROM 111947|Guyana.Potaro-Siparuni
Vampyressa bidens|ABGYE013-06|ROM 109296|Guyana.Potaro-Siparuni
Vampyressa bidens|ABGYE052-06|ROM 109338|Guyana.Potaro-Siparuni
Vampyressa bidens|ABGYC386-06|ROM 107204|Guyana.Potaro-Siparuni
Vampyressa bidens|ABGYD315-06|ROM 108654|Guyana.Potaro-Siparuni
Vampyressa bidens|ABGYD523-06|ROM 108881|Guyana.Potaro-Siparuni
Vampyressa bidens|ABGYE333-06|ROM 111783|Guyana.Potaro-Siparuni
Vampyressa bidens|ABGYA758-06|ROM 100806|Guyana.Barima-Waini
Vampyressa bidens|ABGYF083-06|ROM 113613|Guyana.Demerara-Mahaica
Vampyressa bidens|ABSMS212-06|ROM 117374|Suriname.Sipaliwini
Vampyressa bidens|ABGYB665-06|ROM 103425|Guyana.Upper Demerara-Berbice
Vampyressa bidens|ABGYD441-06|ROM 108792|Guyana.Potaro-Siparuni
Vampyressa bidens|ABGYG024-06|ROM 115517|Guyana.Essequibo Islands-West Demerara
Vampyressa bidens|ABGYB897-06|ROM 104742|Guyana.Potaro-Siparuni
Vampyressa bidens|BCBNT886-06|ROM 115515|Guyana.Essequibo Islands-West Demerara
Vampyressa bidens|ABGYE373-06|ROM 111825|Guyana.Potaro-Siparuni
Vampyressa bidens|ABGYE303-06|ROM 111753|Guyana.Potaro-Siparuni
Vampyressa bidens|ABGYE302-06|ROM 111752|Guyana.Potaro-Siparuni
Vampyressa bidens|ABGYE278-06|ROM 111727|Guyana.Potaro-Siparuni
Vampyressa bidens|ABGYE022-06|ROM 109306|Guyana.Potaro-Siparuni
Vampyressa bidens|ABGYD764-06|ROM 109184|Guyana.Potaro-Siparuni
Vampyressa bidens|ABGYD727-06|ROM 109143|Guyana.Potaro-Siparuni
Vampyressa bidens|ABGYD471-06|ROM 108822|Guyana.Potaro-Siparuni
Vampyressa bidens|ABGYD418-06|ROM 108767|Guyana.Potaro-Siparuni
Vampyressa bidens|ABGYD371-06|ROM 108715|Guyana.Potaro-Siparuni
Vampyressa bidens|ABGYD345-06|ROM 108686|Guyana.Potaro-Siparuni
Vampyressa bidens|ABGYD300-06|ROM 108639|Guyana.Potaro-Siparuni
Vampyressa bidens|ABGYD192-06|ROM 108519|Guyana.Potaro-Siparuni
Vampyressa bidens|ABGYD119-06|ROM 108417|Guyana.Potaro-Siparuni
Vampyressa bidens|ABGYC771-06|ROM 108174|Guyana.Cuyuni-Mazaruni
Vampyressa bidens|ABGYC595-06|ROM 107451|Guyana.Potaro-Siparuni
Vampyressa bidens|ABGYC499-06|ROM 107333|Guyana.Potaro-Siparuni
Vampyressa bidens|ABGYC473-06|ROM 107306|Guyana.Potaro-Siparuni
Vampyressa bidens|ABGYC359-06|ROM 107174|Guyana.Potaro-Siparuni
Vampyressa bidens|ABGYC351-06|ROM 107163|Guyana.Potaro-Siparuni
Vampyressa bidens|ABGYC342-06|ROM 107154|Guyana.Potaro-Siparuni
Vampyressa bidens|ABGYC341-06|ROM 107153|Guyana.Potaro-Siparuni
Vampyressa bidens|ABGYC183-06|ROM 106718|Guyana.Upper Takutu-Upper Essequibo
Vampyressa bidens|ABGYC106-06|ROM 106616|Guyana.Upper Takutu-Upper Essequibo
Vampyressa bidens|ABGYB914-06|ROM 104759|Guyana.Potaro-Siparuni
Vampyressa bidens|ABGYB768-06|ROM 103536|Guyana.Upper Demerara-Berbice
Vampyressa bidens|ABGYB751-06|ROM 103518|Guyana.Upper Demerara-Berbice
Vampyressa bidens|ABGYB336-06|ROM 103043|Guyana.Upper Takutu-Upper Essequibo
Vampyressa bidens|ABGYA886-06|ROM 100964|Guyana.Barima-Waini
Vampyressa bidens|ABECA895-06|ROM 106075|Ecuador.Napo
Vampyressa bidens|ABGYE374-06|ROM 111826|Guyana.Potaro-Siparuni
Vampyressa bidens|ABGYD845-06|ROM 109272|Guyana.Potaro-Siparuni
Vampyressa bidens|ABGYE383-06|ROM 111835|Guyana.Potaro-Siparuni
Vampyressa bidens|ABGYE457-06|ROM 111921|Guyana.Potaro-Siparuni
Vampyressa bidens|ABGYF195-06|ROM 113759|Guyana.Demerara-Mahaica
Vampyressa bidens|ABGYF445-06|ROM 115075|Guyana.Cuyuni-Mazaruni
Vampyressa bidens|ABGYG068-06|ROM 115590|Guyana.Essequibo Islands-West Demerara
Vampyressa bidens|ABGYG491-06|ROM 116631|Guyana.Potaro-Siparuni
Vampyressa bidens|ABGYG512-06|ROM 116654|Guyana.Potaro-Siparuni
Vampyressa bidens|ABGYG521-06|ROM 116663|Guyana.Potaro-Siparuni
Vampyressa bidens|ABGYG522-06|ROM 116664|Guyana.Potaro-Siparuni
Vampyressa bidens|ABGYG523-06|ROM 116665|Guyana.Potaro-Siparuni
Vampyressa bidens|ABGYG565-06|ROM 116719|Guyana.Potaro-Siparuni
Vampyressa bidens|ABGYG571-06|ROM 116732|Guyana.Potaro-Siparuni
Vampyressa bidens|BCBNT916-06|ROM 115587|Guyana.Essequibo Islands-West Demerara
Vampyressa bidens|ABGYA644-06|ROM 100339|Guyana.East Berbice-Corentyne
Vampyressa bidens|ABGYG578-06|ROM 116740|Guyana.Potaro-Siparuni
Vampyressa bidens|BCBNT936-06|ROM 115635|Guyana.Essequibo Islands-West Demerara
Mesophylla macconnelli|ABGYC584-06|ROM 107433|Guyana.Potaro-Siparuni
Mesophylla macconnelli|ABGYE365-06|ROM 111815|Guyana.Potaro-Siparuni
Mesophylla macconnelli|BCBNT307-06|ROM 108620|Guyana.Potaro-Siparuni
Mesophylla macconnelli|ABECA305-06|ROM 105178|Ecuador.Napo
Mesophylla macconnelli|ABECA681-06|ROM 105743|Ecuador.Napo
Mesophylla macconnelli|ABECB064-06|ROM F40543|Ecuador.Napo
Mesophylla macconnelli|ABECA844-06|ROM 106014|Ecuador.Napo
Mesophylla macconnelli|ABECA611-06|ROM 105649|Ecuador.Napo
Mesophylla macconnelli|ABECA342-06|ROM 105229|Ecuador.Napo
Mesophylla macconnelli|ABECA290-06|ROM F37474|Ecuador.Napo
Mesophylla macconnelli|ABGYA599-06|ROM 100286|Guyana.East Berbice-Corentyne
Mesophylla macconnelli|BCBN848-05|ROM 105535|Ecuador.Napo
Mesophylla macconnelli|ABECA942-06|ROM 106133|Ecuador.Napo

Mesophylla macconnelli|ABGYA599-06|ROM 100286|Guyana.East Berbice-Corentyne
Mesophylla macconnelli|BCBN848-05|ROM 105535|Ecuador.Napo
Mesophylla macconnelli|ABECA942-06|ROM 106133|Ecuador.Napo
Mesophylla macconnelli|BCBN757-05|ROM 104509|Ecuador.Napo
Mesophylla macconnelli|ABECA080-06|ROM 104076|Ecuador.Napo
Mesophylla macconnelli|ABECA481-06|ROM F37736|Ecuador.Napo
Mesophylla macconnelli|BCBN852-05|ROM 105570|Ecuador.Napo
Mesophylla macconnelli|BCBN967-05|ROM 105987|Ecuador.Napo
Mesophylla macconnelli|ABGYC280-06|ROM 107071|Guyana.Potaro-Siparuni
Mesophylla macconnelli|BCBNT002-06|ROM 106334|Ecuador.Napo
Mesophylla macconnelli|ABGYB515-06|ROM 103250|Guyana.Upper Takutu-Upper Essequibo
Mesophylla macconnelli|ABGYG833-08|ROM 119261|Guyana
Mesophylla macconnelli|ABGYD388-06|ROM 108734|Guyana.Potaro-Siparuni
Mesophylla macconnelli|ABGYA258-06|ROM 98158|Guyana.Potaro-Siparuni
Mesophylla macconnelli|ABGYG812-08|ROM 119240|Guyana
Mesophylla macconnelli|BCBNT614-06|ROM 113524|Guyana.Upper Takutu-Upper Essequibo
Mesophylla macconnelli|ABECA470-06|ROM 105493|Ecuador.Napo
Mesophylla macconnelli|ABGYG813-08|ROM 119241|Guyana
Mesophylla macconnelli|ABGYG872-08|ROM 119300|Guyana
Mesophylla macconnelli|ABECA046-06|ROM 104030|Ecuador.Napo
Mesophylla macconnelli|ABRMM061-07|ROM F38847|Guyana.Potaro-Siparuni
Mesophylla macconnelli|ABECA892-06|ROM 106071|Ecuador.Napo
Mesophylla macconnelli|ABECA893-06|ROM 106072|Ecuador.Napo
Mesophylla macconnelli|ABGYE033-06|ROM 109319|Guyana.Potaro-Siparuni
Mesophylla macconnelli|ABGYF232-06|ROM 113808|Guyana.Demerara-Mahaica
Mesophylla macconnelli|ABGYE316-06|ROM 111766|Guyana.Potaro-Siparuni
Mesophylla macconnelli|BCBN300-05|ROM 100305|Guyana.East Berbice-Corentyne
Mesophylla macconnelli|BCBNT674-06|ROM 113723|Guyana.Demerara-Mahaica
Chiroderma salvini|BCBN266-05|ROM 99703|Guatemala.El Progreso
Chiroderma villosum|ABECA802-06|ROM 105968|Ecuador.Napo
Chiroderma villosum|ABGYD416-06|ROM 108765|Guyana.Potaro-Siparuni
Chiroderma villosum|BCBN763-05|ROM 104541|Ecuador.Napo
Chiroderma villosum|ABMXA890-06|ROM FN30654|Mexico.Campeche
Chiroderma villosum|BCBN046-05|ROM 96536|Mexico.Campeche
Chiroderma villosum|BCBN384-05|ROM 101245|El Salvador.Ahuachapan
Chiroderma villosum|ABSCA114-06|ROM F38210|Panama.Darien
Chiroderma villosum|BCBN704-05|ROM 104352|Panama.Darien
Chiroderma villosum|ABGYE319-06|ROM 111769|Guyana.Potaro-Siparuni
Chiroderma villosum|ABECA658-06|ROM 105720|Ecuador.Napo
Chiroderma villosum|ABGYD723-06|ROM 109138|Guyana.Potaro-Siparuni
Chiroderma villosum|ABGYD415-06|ROM 108764|Guyana.Potaro-Siparuni
Chiroderma villosum|ABGYE320-06|ROM 111770|Guyana.Potaro-Siparuni
Chiroderma villosum|ABECA224-06|ROM 104549|Ecuador.Napo
Chiroderma villosum|ABGYD799-06|ROM 109221|Guyana.Potaro-Siparuni
Chiroderma villosum|ABGYD622-06|ROM 108998|Guyana.Potaro-Siparuni
Chiroderma villosum|ABGYC132-06|ROM 106644|Guyana.Upper Takutu-Upper Essequibo
Chiroderma villosum|ABGYB590-06|ROM 103331|Guyana.Upper Takutu-Upper Essequibo
Chiroderma villosum|ABGYB479-06|ROM 103214|Guyana.Upper Takutu-Upper Essequibo
Chiroderma villosum|ABECA657-06|ROM 105719|Ecuador.Napo
Chiroderma villosum|ABGYE051-06|ROM 109337|Guyana.Potaro-Siparuni
Chiroderma villosum|ABGYE318-06|ROM 111768|Guyana.Potaro-Siparuni
Chiroderma villosum|ABSMS213-06|ROM 117375|Suriname.Sipaliwini
Chiroderma villosum|BCBN808-05|ROM 105244|Ecuador.Napo
Chiroderma villosum|BCBN811-05|ROM 105254|Ecuador.Napo
Chiroderma villosum|ABGYE024-06|ROM 109308|Guyana.Potaro-Siparuni
Chiroderma villosum|BCBNT102-06|ROM 107112|Guyana.Potaro-Siparuni
Chiroderma villosum|BCBNT324-06|ROM 108843|Guyana.Potaro-Siparuni
Chiroderma villosum|BCBNT511-06|ROM 111628|Guyana.Potaro-Siparuni
Chiroderma villosum|ABGYD757-06|ROM 109175|Guyana.Potaro-Siparuni
Chiroderma villosum|ABGYE023-06|ROM 109307|Guyana.Potaro-Siparuni
Chiroderma villosum|ABECA659-06|ROM 105721|Ecuador.Napo
Chiroderma villosum|BCBN742-05|ROM 104448|Ecuador.Napo
Chiroderma villosum|ABSRA529-06|ROM 117119|Suriname.Nickerie
Chiroderma villosum|ABECA558-06|ROM 105587|Ecuador.Napo
Chiroderma villosum|ABGYE304-06|ROM 111754|Guyana.Potaro-Siparuni
Chiroderma villosum|ABGYE338-06|ROM 111788|Guyana.Potaro-Siparuni
Chiroderma villosum|ABECA373-06|ROM 105267|Ecuador.Napo
Chiroderma villosum|ABECA505-06|ROM F37774|Ecuador.Napo
Chiroderma villosum|ABGYG802-08|ROM 119230|Guyana
Chiroderma villosum|ABGYA441-06|ROM 98850|Guyana.Barima-Waini
Chiroderma villosum|ABGYC805-06|ROM 108203|Guyana.Cuyuni-Mazaruni
Chiroderma villosum|BCBNT101-06|ROM 107111|Guyana.Potaro-Siparuni
Chiroderma villosum|ABGYE384-06|ROM 111836|Guyana.Potaro-Siparuni
Chiroderma villosum|ABGYD843-06|ROM 109270|Guyana.Potaro-Siparuni
Chiroderma villosum|ABGYC817-06|ROM 108219|Guyana.Cuyuni-Mazaruni
Chiroderma villosum|ABECA504-06|ROM 105540|Ecuador.Napo
Chiroderma villosum|ABECA451-06|ROM 105361|Ecuador.Napo
Chiroderma villosum|ABECA225-06|ROM F37400|Ecuador.Napo
Chiroderma villosum|ABECA217-06|ROM 104540|Ecuador.Napo
Chiroderma villosum|ABGYE393-06|ROM 111845|Guyana.Potaro-Siparuni
Chiroderma villosum|BCBN956-05|ROM 105928|Ecuador.Napo
Chiroderma villosum|BCBNT134-06|ROM 107394|Guyana.Potaro-Siparuni
Chiroderma villosum|BCBNT847-06|ROM 114212|Suriname.Brokopondo
Chiroderma villosum|BCBNT855-06|ROM 114228|Suriname.Brokopondo
Chiroderma doriae|BCBNT502-06|ROM 111149|Brazil.Sao Paulo
Chiroderma doriae|BCBNT492-06|ROM 111114|Brazil.Sao Paulo
Chiroderma doriae|BCBNT500-06|ROM 111141|Brazil.Sao Paulo
Chiroderma doriae|BCBNT508-06|ROM 111163|Brazil.Sao Paulo
Chiroderma trinitatum|ABGYD223-06|ROM 108554|Guyana.Potaro-Siparuni
Chiroderma trinitatum|ABGYB720-06|ROM 103486|Guyana.Upper Demerara-Berbice
Chiroderma trinitatum|ABGYG211-06|ROM 115807|Guyana.Potaro-Siparuni
Chiroderma trinitatum|ABGYD617-06|ROM 108993|Guyana.Potaro-Siparuni
Chiroderma trinitatum|ABSRA493-06|ROM 117083|Suriname.Nickerie
Chiroderma trinitatum|BCBNT110-06|ROM 107205|Guyana.Potaro-Siparuni
Chiroderma trinitatum|ABSRA437-06|ROM 117027|Suriname.Nickerie
Chiroderma trinitatum|ABSMS393-06|ROM 117555|Suriname.Sipaliwini
Chiroderma trinitatum|ABGYE359-06|ROM 111809|Guyana.Potaro-Siparuni
Chiroderma trinitatum|ABGYE047-06|ROM 109333|Guyana.Potaro-Siparuni
Chiroderma trinitatum|ABGYD774-06|ROM 109195|Guyana.Potaro-Siparuni
Chiroderma trinitatum|ABGYD024-06|ROM 108244|Guyana.Cuyuni-Mazaruni
Chiroderma trinitatum|ABGYD251-06|ROM 108587|Guyana.Potaro-Siparuni
Chiroderma trinitatum|ABGYB736-06|ROM 103503|Guyana.Upper Demerara-Berbice
Chiroderma trinitatum|BCBNT828-06|ROM 114170|Suriname.Brokopondo

Chiroderma trinitatum|ABGYD251-06|ROM 108587|Guyana.Potaro-Siparuni
Chiroderma trinitatum|ABGYB736-06|ROM 103503|Guyana.Upper Demerara-Berbice
Chiroderma trinitatum|BCBNT828-06|ROM 114170|Suriname.Brokopondo
Chiroderma trinitatum|BCBNT856-06|ROM 114233|Suriname.Brokopondo
Chiroderma trinitatum|BCBN810-05|ROM 105253|Ecuador.Napo
Chiroderma trinitatum|ABGYG490-06|ROM 116630|Guyana.Potaro-Siparuni
Chiroderma trinitatum|BCBNT329-06|ROM 108889|Guyana.Potaro-Siparuni
Chiroderma trinitatum|BCBNT848-06|ROM 114213|Suriname.Brokopondo
Chiroderma trinitatum|ABECA343-06|ROM 105230|Ecuador.Napo
Chiroderma trinitatum|ABGYB738-06|ROM 103505|Guyana.Upper Demerara-Berbice
Chiroderma trinitatum|ABSMS214-06|ROM 117376|Suriname.Sipaliwini
Chiroderma trinitatum|BCBN790-05|ROM 105191|Ecuador.Napo
Chiroderma trinitatum|ABECB028-06|ROM F40504|Ecuador.Napo
Chiroderma trinitatum|ABGYD574-06|ROM 108950|Guyana.Potaro-Siparuni
Chiroderma trinitatum|ABECA551-06|ROM 105581|Ecuador.Napo
Chiroderma trinitatum|BCBN807-05|ROM 105243|Ecuador.Napo
Chiroderma trinitatum|ABECA656-06|ROM 105718|Ecuador.Napo
Chiroderma trinitatum|ABECB027-06|ROM 106342|Ecuador.Napo
Chiroderma trinitatum|BCBN877-05|ROM 105685|Ecuador.Napo
Chiroderma trinitatum|BCBN906-05|ROM 105766|Ecuador.Napo
Chiroderma trinitatum|BCBNT137-06|ROM 107419|Guyana.Potaro-Siparuni
Chiroderma trinitatum|ABGYD252-06|ROM 108588|Guyana.Potaro-Siparuni
Chiroderma trinitatum|ABGYG1072-08|ROM 118996|Guyana
Chiroderma trinitatum|BCBNT314-06|ROM 108714|Guyana.Potaro-Siparuni
Chiroderma trinitatum|ABGYE184-06|ROM 111627|Guyana.Potaro-Siparuni
Chiroderma trinitatum|ABGYC743-06|ROM 108144|Guyana.Cuyuni-Mazaruni
Chiroderma trinitatum|ABGYD844-06|ROM 109271|Guyana.Potaro-Siparuni
Chiroderma trinitatum|ABGYE426-06|ROM 111884|Guyana.Potaro-Siparuni
Chiroderma trinitatum|ABGYE474-06|ROM 111946|Guyana.Potaro-Siparuni
Chiroderma trinitatum|ABSRA414-06|ROM 117003|Suriname.Nickerie
Chiroderma trinitatum|ABGYD156-06|ROM 108463|Guyana.Potaro-Siparuni
Chiroderma trinitatum|BCBNT857-06|ROM 114234|Suriname.Brokopondo
Vampyrodes caraccioli|ABSCA109-06|ROM F38200|Panama.Darien
Vampyrodes caraccioli|BCBN700-05|ROM 104346|Panama.Darien
Vampyrodes caraccioli|BCBN701-05|ROM 104347|Panama.Darien
Vampyrodes caraccioli|ABECA086-06|ROM 104078|Ecuador.Napo
Vampyrodes caraccioli|ABECA344-06|ROM 105231|Ecuador.Napo
Vampyrodes caraccioli|ABECA610-06|ROM 105648|Ecuador.Napo
Vampyrodes caraccioli|ABECB039-06|ROM 106349|Ecuador.Napo
Vampyrodes caraccioli|ABECA646-06|ROM F37948|Ecuador.Napo
Vampyrodes caraccioli|ABECA941-06|ROM 106124|Ecuador.Napo
Vampyrodes caraccioli|BCBN768-05|ROM 104552|Ecuador.Napo
Vampyrodes caraccioli|ABECA067-06|ROM 104059|Ecuador.Napo
Vampyrodes caraccioli|ABECA633-06|ROM 105679|Ecuador.Napo
Vampyrodes caraccioli|BCBN805-05|ROM 105239|Ecuador.Napo
Vampyrodes caraccioli|ABECA652-06|ROM 105707|Ecuador.Napo
Vampyrodes caraccioli|ABECA644-06|ROM 105700|Ecuador.Napo
Vampyrodes caraccioli|ABECA226-06|ROM F37401|Ecuador.Napo
Vampyrodes caraccioli|ABECA351-06|ROM F37561|Ecuador.Napo
Vampyrodes caraccioli|ABECA645-06|ROM 105701|Ecuador.Napo
Vampyrodes caraccioli|ABECA228-06|ROM 104553|Ecuador.Napo
Vampyrodes caraccioli|ABECA350-06|ROM 105241|Ecuador.Napo
Vampyrodes caraccioli|ABECA841-06|ROM 106010|Ecuador.Napo
Vampyrodes caraccioli|ABECA847-06|ROM 106021|Ecuador.Napo
Vampyrodes caraccioli|ABECB037-06|ROM F40514|Ecuador.Napo
Vampyrodes caraccioli|ABECB038-06|ROM F40515|Ecuador.Napo
Vampyrodes caraccioli|BCBN764-05|ROM 104542|Ecuador.Napo
Vampyrodes caraccioli|BCBN806-05|ROM 105240|Ecuador.Napo
Vampyrodes caraccioli|ABECA183-06|ROM 104498|Ecuador.Napo
Vampyrodes caraccioli|BCBNT004-06|ROM 106348|Ecuador.Napo
Vampyrodes caraccioli|ABGYD219-06|ROM 108550|Guyana.Potaro-Siparuni
Vampyrodes caraccioli|ABGYD353-06|ROM 108694|Guyana.Potaro-Siparuni
Vampyrodes caraccioli|ABGYD354-06|ROM 108695|Guyana.Potaro-Siparuni
Vampyrodes caraccioli|BCBNT315-06|ROM 108717|Guyana.Potaro-Siparuni
Vampyrodes caraccioli|ABGYD313-06|ROM 108652|Guyana.Potaro-Siparuni
Vampyrodes caraccioli|ABGYD337-06|ROM 108677|Guyana.Potaro-Siparuni
Vampyrodes caraccioli|ABGYD346-06|ROM 108687|Guyana.Potaro-Siparuni
Vampyrodes caraccioli|ABGYD347-06|ROM 108688|Guyana.Potaro-Siparuni
Vampyrodes caraccioli|ABGYD352-06|ROM 108693|Guyana.Potaro-Siparuni
Vampyrodes caraccioli|ABGYD253-06|ROM 108589|Guyana.Potaro-Siparuni
Vampyrodes caraccioli|ABGYD311-06|ROM 108650|Guyana.Potaro-Siparuni
Vampyrodes caraccioli|ABGYD500-06|ROM 108858|Guyana.Potaro-Siparuni
Vampyrodes caraccioli|ABSMS340-06|ROM 117502|Suriname.Sipaliwini
Vampyrodes caraccioli|ABGYD312-06|ROM 108651|Guyana.Potaro-Siparuni
Vampyrodes caraccioli|ABGYD314-06|ROM 108653|Guyana.Potaro-Siparuni
Vampyrodes caraccioli|ABGYD220-06|ROM 108551|Guyana.Potaro-Siparuni
Vampyrodes caraccioli|ABGYD221-06|ROM 108552|Guyana.Potaro-Siparuni
Vampyrodes caraccioli|ABGYD222-06|ROM 108553|Guyana.Potaro-Siparuni
Vampyrodes caraccioli|ABGYD249-06|ROM 108581|Guyana.Potaro-Siparuni
Vampyrodes caraccioli|ABGYD203-06|ROM 108533|Guyana.Potaro-Siparuni
Vampyrodes caraccioli|ABGYD204-06|ROM 108534|Guyana.Potaro-Siparuni
Vampyrodes caraccioli|ABSMS343-06|ROM 117505|Suriname.Sipaliwini
Vampyrodes caraccioli|ABSMS352-06|ROM 117514|Suriname.Sipaliwini
Vampyrodes caraccioli|ABSMS370-06|ROM 117532|Suriname.Sipaliwini
Vampyrodes caraccioli|ABSRA518-06|ROM 117108|Suriname.Nickerie
Vampyrodes caraccioli|BCBNT312-06|ROM 108696|Guyana.Potaro-Siparuni
Vampyrodes caraccioli|BCBNT317-06|ROM 108756|Guyana.Potaro-Siparuni
Vampyrodes caraccioli|BCBNT318-06|ROM 108762|Guyana.Potaro-Siparuni
Vampyrodes caraccioli|BCBNT321-06|ROM 108781|Guyana.Potaro-Siparuni
Vampyrodes caraccioli|BCBNT739-06|ROM 113918|Suriname.Brokopondo
Platyrrhinus lineatus|BCBNT477-06|ROM 111084|Brazil.Sao Paulo
Platyrrhinus lineatus|BCBNT506-06|ROM 111156|Brazil.Sao Paulo
Platyrrhinus helleri|BCBN232-05|ROM 99523|Guatemala.Peten
Platyrrhinus helleri|BCBN646-05|ROM 104261|Panama
Platyrrhinus helleri|BCBNT443-06|ROM 108321|Costa Rica.Limon
Platyrrhinus helleri|BCBN657-05|ROM 104276|Panama
Platyrrhinus helleri|BCBNT432-06|ROM 108310|Costa Rica.Limon
Platyrrhinus helleri|BCBN681-05|ROM 104311|Panama.Chiriqui
Platyrrhinus helleri|BCBN386-05|ROM 101251|El Salvador.Ahuachapan
Platyrrhinus helleri|BCBN218-05|ROM 99614|Guatemala.Peten
Platyrrhinus helleri|ABSCA138-06|ROM F44040|Costa Rica.Limon
Platyrrhinus helleri|ABSCA074-06|ROM F38121|Panama
Platyrrhinus helleri|BCBNT447-06|ROM 108325|Costa Rica.Limon

[illegible]
Platyrrhinus helleri|ABSCA138-06|ROM F44040|Costa Rica.Limon
Platyrrhinus helleri|ABSCA074-06|ROM F38121|Panama
Platyrrhinus helleri|BCBNT447-06|ROM 108325|Costa Rica.Limon
Platyrrhinus helleri|ABECA356-06|ROM 105248|Ecuador.Napo
Platyrrhinus helleri|ABECB071-08|ROM 118733|Ecuador
Platyrrhinus helleri|ABECB100-08|ROM 118762|Ecuador
Platyrrhinus helleri|ABGYC081-06|ROM 106579|Guyana.Upper Takutu-Upper Essequibo
Platyrrhinus helleri|ABGYC102-06|ROM 106610|Guyana.Upper Takutu-Upper Essequibo
Platyrrhinus helleri|ABSRA470-06|ROM 117060|Suriname.Nickerie
Platyrrhinus helleri|ABECA552-06|ROM 105582|Ecuador.Napo
Platyrrhinus helleri|ABECB029-06|ROM F40505|Ecuador.Napo
Platyrrhinus helleri|BCBN723-05|ROM 104393|Ecuador.Napo
Platyrrhinus helleri|ABGYD355-06|ROM 108697|Guyana.Potaro-Siparuni
Platyrrhinus helleri|ABSMS351-06|ROM 117513|Suriname.Sipaliwini
Platyrrhinus helleri|ABSMS167-06|ROM 117329|Suriname.Sipaliwini
Platyrrhinus helleri|ABGYC070-06|ROM 106567|Guyana.Upper Takutu-Upper Essequibo
Platyrrhinus helleri|ABGYF165-06|ROM 113721|Guyana.Demerara-Mahaica
Platyrrhinus helleri|ABSMS027-06|ROM 117189|Suriname.Sipaliwini
Platyrrhinus helleri|ABSRA406-06|ROM 116995|Suriname.Nickerie
Platyrrhinus helleri|ABSRA441-06|ROM 117031|Suriname.Nickerie
Platyrrhinus helleri|ABGYD342-06|ROM 108683|Guyana.Potaro-Siparuni
Platyrrhinus helleri|BCBNT740-06|ROM 113919|Suriname.Brokopondo
Platyrrhinus helleri|ABGYE317-06|ROM 111767|Guyana.Potaro-Siparuni
Platyrrhinus helleri|ABSRA442-06|ROM 117032|Suriname.Nickerie
Platyrrhinus helleri|ABGYA076-06|ROM 97898|Guyana.Upper Takutu-Upper Essequibo
Platyrrhinus helleri|ABGYB594-06|ROM 103335|Guyana.Upper Takutu-Upper Essequibo
Platyrrhinus helleri|ABGYE308-06|ROM 111758|Guyana.Potaro-Siparuni
Platyrrhinus helleri|ABGYE485-06|ROM 111957|Guyana.Potaro-Siparuni
Platyrrhinus helleri|ABGYE582-06|ROM 112059|Guyana.Potaro-Siparuni
Platyrrhinus helleri|ABGYF166-06|ROM 113722|Guyana.Demerara-Mahaica
Platyrrhinus helleri|ABSMS323-06|ROM 117485|Suriname.Sipaliwini
Platyrrhinus helleri|ABSMS358-06|ROM 117520|Suriname.Sipaliwini
Platyrrhinus helleri|ABSRA313-06|ROM 116902|Suriname.Nickerie
Platyrrhinus helleri|ABSRA415-06|ROM 117004|Suriname.Nickerie
Platyrrhinus helleri|ABSRA458-06|ROM 117048|Suriname.Nickerie
Platyrrhinus helleri PS1|BCBNT602-06|ROM 113487|Guyana.Upper Takutu-Upper Essequibo
Platyrrhinus helleri|BCBNT766-06|ROM 113976|Suriname.Brokopondo
Platyrrhinus recifinus|BCBNT488-06|ROM 111106|Brazil.Sao Paulo
Platyrrhinus recifinus|BCBNT496-06|ROM 111133|Brazil.Sao Paulo
Platyrrhinus recifinus|BCBNT497-06|ROM 111134|Brazil.Sao Paulo
Platyrrhinus helleri|ABSMS047-06|ROM 117209|Suriname.Sipaliwini
Platyrrhinus helleri|ABSMS063-06|ROM 117225|Suriname.Sipaliwini
Platyrrhinus helleri|ABSRA459-06|ROM 117049|Suriname.Nickerie
Platyrrhinus helleri|ABSMS481-06|ROM 117643|Suriname.Sipaliwini
Platyrrhinus helleri|ABGYD531-06|ROM 108890|Guyana.Potaro-Siparuni
Platyrrhinus helleri|ABGYE581-06|ROM 112058|Guyana.Potaro-Siparuni
Platyrrhinus helleri|ABGYF006-06|ROM 113492|Guyana.Upper Takutu-Upper Essequibo
Platyrrhinus helleri|ABGYA061-06|ROM 97878|Guyana.Upper Takutu-Upper Essequibo
Platyrrhinus helleri|ABSMS486-06|ROM 117648|Suriname.Sipaliwini
Platyrrhinus helleri|ABSMS236-06|ROM 117398|Suriname.Sipaliwini
Platyrrhinus helleri|ABSMS297-06|ROM 117459|Suriname.Sipaliwini
Platyrrhinus helleri|ABSRA303-06|ROM 116892|Suriname.Nickerie
Platyrrhinus helleri|ABGYE012-06|ROM 109295|Guyana.Potaro-Siparuni
Platyrrhinus helleri|ABSMS044-06|ROM 117206|Suriname.Sipaliwini
Platyrrhinus helleri|BCBNT601-06|ROM 113465|Guyana.Upper Takutu-Upper Essequibo
Platyrrhinus helleri|ABSMS028-06|ROM 117190|Suriname.Sipaliwini
Platyrrhinus helleri|ABGYG834-08|ROM 119262|Guyana
Platyrrhinus helleri|ABSMS341-06|ROM 117503|Suriname.Sipaliwini
Platyrrhinus helleri PS2|BCBNT639-06|ROM 113584|Guyana.Upper Takutu-Upper Essequibo
Platyrrhinus helleri|ABSMS493-06|ROM 117655|Suriname.Sipaliwini
Platyrrhinus helleri|ABSMS307-06|ROM 117469|Suriname.Sipaliwini
Platyrrhinus helleri|ABSMS096-06|ROM 117258|Suriname.Sipaliwini
Platyrrhinus helleri|ABGYD167-06|ROM 108487|Guyana.Potaro-Siparuni
Platyrrhinus helleri|ABSMS342-06|ROM 117504|Suriname.Sipaliwini
Platyrrhinus helleri|ABSMS413-06|ROM 117575|Suriname.Sipaliwini
Platyrrhinus helleri|ABGYE483-06|ROM 111955|Guyana.Potaro-Siparuni
Platyrrhinus helleri|ABSRA519-06|ROM 117109|Suriname.Nickerie
Platyrrhinus helleri|BCBNT772-06|ROM 113991|Suriname.Brokopondo
Platyrrhinus helleri|ABGYE228-06|ROM 111676|Guyana.Potaro-Siparuni
Platyrrhinus helleri|ABSMS043-06|ROM 117205|Suriname.Sipaliwini
Platyrrhinus helleri PS2|BCBNT603-06|ROM 113491|Guyana.Upper Takutu-Upper Essequibo
Platyrrhinus helleri|BCBNT799-06|ROM 114070|Suriname.Brokopondo
Platyrrhinus helleri|ABECA843-06|ROM 106012|Ecuador.Napo
Platyrrhinus helleri|ABECA138-06|ROM 104436|Ecuador.Napo
Platyrrhinus helleri|ABECA355-06|ROM F37568|Ecuador.Napo
Platyrrhinus helleri|ABECA537-06|ROM F37806|Ecuador.Napo
Platyrrhinus helleri|ABECB030-06|ROM 106343|Ecuador.Napo
Platyrrhinus helleri|ABECA860-06|ROM 106036|Ecuador.Napo
Platyrrhinus helleri|ABECA735-06|ROM 105881|Ecuador.Napo
Platyrrhinus helleri|BCBN582-05|ROM 104039|Ecuador.Napo
Platyrrhinus helleri|ABECA104-06|ROM 104394|Ecuador.Napo
Platyrrhinus helleri|ABECA153-06|ROM F37296|Ecuador.Napo
Platyrrhinus helleri|ABECA395-06|ROM 105292|Ecuador.Napo
Platyrrhinus helleri|ABECA553-06|ROM 105583|Ecuador.Napo
Platyrrhinus helleri|ABECA842-06|ROM 106011|Ecuador.Napo
Platyrrhinus helleri|ABECA829-06|ROM 105998|Ecuador.Napo
Platyrrhinus helleri|ABECA828-06|ROM 105997|Ecuador.Napo
Platyrrhinus helleri|ABECA502-06|ROM 105534|Ecuador.Napo
Platyrrhinus helleri|ABECA345-06|ROM 105232|Ecuador.Napo
Platyrrhinus helleri|ABECA257-06|ROM 105127|Ecuador.Napo
Platyrrhinus helleri|ABECA159-06|ROM 104465|Ecuador.Napo
Platyrrhinus helleri|ABECA103-06|ROM 104392|Ecuador.Napo
Platyrrhinus helleri|ABECA082-06|ROM F37190|Ecuador.Napo
Platyrrhinus helleri|ABECA554-06|ROM 105584|Ecuador.Napo
Platyrrhinus helleri|ABECB017-06|ROM F40492|Ecuador.Napo
Platyrrhinus helleri|BCBN798-05|ROM 105211|Ecuador.Napo
Platyrrhinus helleri|ABECA512-06|ROM 105546|Ecuador.Napo
Platyrrhinus helleri|BCBN960-05|ROM 105937|Ecuador.Napo
Platyrrhinus helleri|ABECA357-06|ROM 105249|Ecuador.Napo
Platyrrhinus helleri|ABECA745-06|ROM 105892|Ecuador.Napo
Platyrrhinus helleri|ABSMS046-06|ROM 117208|Suriname.Sipaliwini
Platyrrhinus helleri|ABGYA186-06|ROM 98054|Guyana.Potaro-Siparuni
[illegible]

Platyrrhinus helleri|ABECA745-06|ROM 105892|Ecuador.Napo
Platyrrhinus helleri|ABSMS046-06|ROM 117208|Suriname.Sipaliwini
Platyrrhinus helleri|ABGYA186-06|ROM 98054|Guyana.Potaro-Siparuni
Platyrrhinus helleri|ABGYC772-06|ROM 108175|Guyana.Cuyuni-Mazaruni
Platyrrhinus helleri|ABGYD050-06|ROM 108273|Guyana.Cuyuni-Mazaruni
Platyrrhinus helleri|ABSMS215-06|ROM 117377|Suriname.Sipaliwini
Platyrrhinus helleri|ABSMS026-06|ROM 117188|Suriname.Sipaliwini
Platyrrhinus helleri|ABSMS121-06|ROM 117283|Suriname.Sipaliwini
Platyrrhinus helleri|ABSMS394-06|ROM 117556|Suriname.Sipaliwini
Platyrrhinus helleri|ABSMS168-06|ROM 117330|Suriname.Sipaliwini
Platyrrhinus helleri|ABSRA461-06|ROM 117051|Suriname.Nickerie
Platyrrhinus helleri PS3|BCBNT941-06|ROM 115642|Guyana.Potaro-Siparuni
Platyrrhinus helleri|BCBNT846-06|ROM 114211|Suriname.Brokopondo
Platyrrhinus helleri|ABGYB032-06|ROM 101004|Guyana.Barima-Waini
Platyrrhinus helleri|ABGYE659-06|ROM 112579|Guyana.Demerara-Mahaica
Platyrrhinus helleri|ABGYC116-06|ROM 106628|Guyana.Upper Takutu-Upper Essequibo
Platyrrhinus helleri|ABGYF159-06|ROM 113712|Guyana.Demerara-Mahaica
Platyrrhinus helleri|ABSMS086-06|ROM 117248|Suriname.Sipaliwini
Platyrrhinus helleri|ABSRA460-06|ROM 117050|Suriname.Nickerie
Platyrrhinus helleri|ABGYF143-06|ROM 113694|Guyana.Demerara-Mahaica
Platyrrhinus helleri|ABGYG505-06|ROM 116647|Guyana.Potaro-Siparuni
Platyrrhinus helleri|ABGYB099-06|ROM 101076|Guyana.Barima-Waini
Platyrrhinus helleri|ABGYB081-06|ROM 101058|Guyana.Barima-Waini
Platyrrhinus helleri|ABGYE668-06|ROM 112589|Guyana.Demerara-Mahaica
Platyrrhinus helleri|ABGYE684-06|ROM 112612|Guyana.Demerara-Mahaica
Platyrrhinus helleri|ABGYF181-06|ROM 113738|Guyana.Demerara-Mahaica
Platyrrhinus helleri|ABGYB047-06|ROM 101019|Guyana.Barima-Waini
Platyrrhinus helleri|ABSMS087-06|ROM 117249|Suriname.Sipaliwini
Platyrrhinus helleri|ABSMS045-06|ROM 117207|Suriname.Sipaliwini
Platyrrhinus helleri|ABGYB517-06|ROM 103252|Guyana.Upper Takutu-Upper Essequibo
Platyrrhinus helleri|ABSRA308-06|ROM 116897|Suriname.Nickerie
Platyrrhinus helleri|ABSMS330-06|ROM 117492|Suriname.Sipaliwini
Platyrrhinus helleri|ABSMS048-06|ROM 117210|Suriname.Sipaliwini
Platyrrhinus helleri|ABSMS029-06|ROM 117191|Suriname.Sipaliwini
Platyrrhinus helleri|ABSMS011-06|ROM 117173|Suriname.Sipaliwini
Platyrrhinus helleri|ABGYG025-06|ROM 115518|Guyana.Essequibo Islands-West Demerara
Platyrrhinus helleri|ABGYF220-06|ROM 113791|Guyana.Demerara-Mahaica
Platyrrhinus helleri|ABGYF204-06|ROM 113774|Guyana.Demerara-Mahaica
Platyrrhinus helleri|ABGYF194-06|ROM 113758|Guyana.Demerara-Mahaica
Platyrrhinus helleri|ABGYE654-06|ROM 112574|Guyana.Demerara-Mahaica
Platyrrhinus helleri|ABGYE621-06|ROM 112529|Guyana.Demerara-Mahaica
Platyrrhinus helleri|ABGYE620-06|ROM 112528|Guyana.Demerara-Mahaica
Platyrrhinus helleri|ABGYE484-06|ROM 111956|Guyana.Potaro-Siparuni
Platyrrhinus helleri|ABGYE229-06|ROM 111677|Guyana.Potaro-Siparuni
Platyrrhinus helleri|ABGYD573-06|ROM 108949|Guyana.Potaro-Siparuni
Platyrrhinus helleri|ABGYD548-06|ROM 108913|Guyana.Potaro-Siparuni
Platyrrhinus helleri|ABGYD499-06|ROM 108857|Guyana.Potaro-Siparuni
Platyrrhinus helleri|ABGYD478-06|ROM 108831|Guyana.Potaro-Siparuni
Platyrrhinus helleri|ABGYD283-06|ROM 108619|Guyana.Potaro-Siparuni
Platyrrhinus helleri|ABGYD170-06|ROM 108490|Guyana.Potaro-Siparuni
Platyrrhinus helleri|ABGYC806-06|ROM 108204|Guyana.Cuyuni-Mazaruni
Platyrrhinus helleri|ABGYC758-06|ROM 108161|Guyana.Cuyuni-Mazaruni
Platyrrhinus helleri|ABGYC247-06|ROM 107027|Guyana.Potaro-Siparuni
Platyrrhinus helleri|ABGYB100-06|ROM 101077|Guyana.Barima-Waini
Platyrrhinus helleri|ABGYB080-06|ROM 101057|Guyana.Barima-Waini
Platyrrhinus helleri|ABGYC759-06|ROM 108162|Guyana.Cuyuni-Mazaruni
Platyrrhinus helleri|ABGYB082-06|ROM 101059|Guyana.Barima-Waini
Platyrrhinus helleri|ABGYE683-06|ROM 112611|Guyana.Demerara-Mahaica
Platyrrhinus helleri|ABGYB101-06|ROM 101078|Guyana.Barima-Waini
Platyrrhinus helleri|ABSRA433-06|ROM 117023|Suriname.Nickerie
Platyrrhinus helleri|ABSRA434-06|ROM 117024|Suriname.Nickerie
Platyrrhinus helleri|ABSRA443-06|ROM 117033|Suriname.Nickerie
Platyrrhinus helleri|ABSRA490-06|ROM 117080|Suriname.Nickerie
Platyrrhinus helleri|BCBN730-05|ROM 104415|Ecuador.Napo
Platyrrhinus helleri PS3|BCBNT664-06|ROM 113673|Guyana.Demerara-Mahaica
Platyrrhinus helleri PS3|BCBNT945-06|ROM 115668|Guyana.Potaro-Siparuni
Platyrrhinus helleri|ABGYE277-06|ROM 111726|Guyana.Potaro-Siparuni
Platyrrhinus helleri|ABGYD664-06|ROM 109075|Guyana.Potaro-Siparuni
Platyrrhinus helleri|ABGYE482-06|ROM 111954|Guyana.Potaro-Siparuni
Platyrrhinus helleri|ABGYB516-06|ROM 103251|Guyana.Upper Takutu-Upper Essequibo
Platyrrhinus helleri|ABSMS030-06|ROM 117192|Suriname.Sipaliwini
Platyrrhinus helleri PS3|BCBNT868-06|ROM 115473|Guyana.Essequibo Islands-West Demerara
Platyrrhinus helleri PS3|BCBNT942-06|ROM 115643|Guyana.Potaro-Siparuni
Platyrrhinus helleri PS3|BCBNT956-06|ROM 115721|Guyana.Potaro-Siparuni
Platyrrhinus helleri PS3|BCBNT957-06|ROM 115722|Guyana.Potaro-Siparuni
Platyrrhinus brachycephalus|BCBNT189-06|ROM 107889|Venezuela.Amazonas
Platyrrhinus brachycephalus|BCBNT671-06|ROM 113713|Guyana.Demerara-Mahaica
Platyrrhinus brachycephalus|BCBNT690-06|ROM 113802|Guyana.Demerara-Mahaica
Platyrrhinus vittatus|BCBN173-05|ROM 97305|Costa Rica.Puntarenas
Platyrrhinus infuscus|ABECA627-06|ROM 105670|Ecuador.Napo
Platyrrhinus infuscus|ABECA379-06|ROM F37599|Ecuador.Napo
Platyrrhinus infuscus|BCBN569-05|ROM 104009|Ecuador.Napo
Platyrrhinus infuscus|BCBN733-05|ROM 104421|Ecuador.Napo
Platyrrhinus infuscus|BCBN983-05|ROM 106123|Ecuador.Napo
Platyrrhinus infuscus|ABECA513-06|ROM 105547|Ecuador.Napo
Platyrrhinus infuscus|ABECA982-06|ROM 106220|Ecuador.Napo
Platyrrhinus infuscus|ABECB102-08|ROM 118764|Ecuador
Platyrrhinus infuscus|BCBN884-05|ROM 105699|Ecuador.Napo
Platyrrhinus infuscus|ABECA647-06|ROM 105702|Ecuador.Napo
Platyrrhinus infuscus|BCBN963-05|ROM 105953|Ecuador.Napo
Platyrrhinus infuscus|ABECA680-06|ROM 105742|Ecuador.Napo
Platyrrhinus infuscus|ABECA124-06|ROM 104420|Ecuador.Napo
Platyrrhinus infuscus|BCBN867-05|ROM 105657|Ecuador.Napo
Platyrrhinus infuscus|BCBN556-05|ROM 103981|Ecuador.Napo
Platyrrhinus infuscus|BCBN783-05|ROM 105167|Ecuador.Napo
Platyrrhinus infuscus|ABECA068-06|ROM F37169|Ecuador.Napo
Platyrrhinus infuscus|ABECA114-06|ROM F37238|Ecuador.Napo
Platyrrhinus infuscus|ABECA354-06|ROM 105247|Ecuador.Napo
Platyrrhinus infuscus|ABECA369-06|ROM 105263|Ecuador.Napo
Platyrrhinus infuscus|BCBN760-05|ROM 104516|Ecuador.Napo
Platyrrhinus infuscus|ABECA115-06|ROM 104407|Ecuador.Napo
Platyrrhinus infuscus|ABECA632-06|ROM F37923|Ecuador.Napo
Platyrrhinus infuscus|ABECA113-06|ROM 104406|Ecuador.Napo

Platyrrhinus infuscus|ABECA115-06|ROM 104407|Ecuador.Napo
Platyrrhinus infuscus|ABECA632-06|ROM F37923|Ecuador.Napo
Platyrrhinus infuscus|ABECA113-06|ROM 104406|Ecuador.Napo
Platyrrhinus infuscus|ABECA025-06|ROM F37099|Ecuador.Napo
Platyrrhinus infuscus|ABECA307-06|ROM 105181|Ecuador.Napo
Platyrrhinus infuscus|ABECA308-06|ROM 105182|Ecuador.Napo
Platyrrhinus infuscus|ABECB101-08|ROM 118763|Ecuador
Platyrrhinus infuscus|BCBN992-05|ROM 106221|Ecuador.Napo
Platyrrhinus aurarius|ABGYF650-06|ROM 115315|Guyana.Cuyuni-Mazaruni
Platyrrhinus aurarius|BCBNC168-06|ROM 115972|Guyana.Potaro-Siparuni
Platyrrhinus aurarius|ABGYG349-06|ROM 115985|Guyana.Potaro-Siparuni
Platyrrhinus aurarius|BCBNT260-06|ROM 108220|Guyana.Cuyuni-Mazaruni
Platyrrhinus aurarius|ABGYG351-06|ROM 115987|Guyana.Potaro-Siparuni
Platyrrhinus aurarius|BCBNC174-06|ROM 116001|Guyana.Potaro-Siparuni
Platyrrhinus aurarius|BCBNC159-06|ROM 115926|Guyana.Potaro-Siparuni
Platyrrhinus aurarius|ABGYG311-06|ROM 115938|Guyana.Potaro-Siparuni
Platyrrhinus aurarius|ABGYG343-06|ROM 115979|Guyana.Potaro-Siparuni
Platyrrhinus aurarius|ABGYG347-06|ROM 115983|Guyana.Potaro-Siparuni
Platyrrhinus aurarius|ABGYG350-06|ROM 115986|Guyana.Potaro-Siparuni
Platyrrhinus aurarius|BCBNC126-06|ROM 115792|Guyana.Potaro-Siparuni
Platyrrhinus aurarius|BCBNC156-06|ROM 115920|Guyana.Potaro-Siparuni
Platyrrhinus aurarius|BCBNC172-06|ROM 115976|Guyana.Potaro-Siparuni
Platyrrhinus aurarius|BCBNT991-06|ROM 114661|Guyana.Potaro-Siparuni
Platyrrhinus aurarius|ABGYG299-06|ROM 115918|Guyana.Potaro-Siparuni
Platyrrhinus aurarius|ABGYG300-06|ROM 115919|Guyana.Potaro-Siparuni
Platyrrhinus aurarius|BCBNC085-06|ROM 115316|Guyana.Cuyuni-Mazaruni
Platyrrhinus aurarius|BCBNT978-06|ROM 114634|Guyana.Potaro-Siparuni
Platyrrhinus aurarius|ABGYG341-06|ROM 115977|Guyana.Potaro-Siparuni
Platyrrhinus aurarius|ABGYG342-06|ROM 115978|Guyana.Potaro-Siparuni
Platyrrhinus aurarius|ABGYG344-06|ROM 115980|Guyana.Potaro-Siparuni
Platyrrhinus aurarius|ABGYG262-06|ROM 115874|Guyana.Potaro-Siparuni
Platyrrhinus aurarius|ABGYG312-06|ROM 115939|Guyana.Potaro-Siparuni
Platyrrhinus aurarius|ABGYG353-06|ROM 115989|Guyana.Potaro-Siparuni
Platyrrhinus aurarius|ABGYG355-06|ROM 115991|Guyana.Potaro-Siparuni
Platyrrhinus aurarius|ABGYG348-06|ROM 115984|Guyana.Potaro-Siparuni
Platyrrhinus aurarius|ABGYG352-06|ROM 115988|Guyana.Potaro-Siparuni
Platyrrhinus aurarius|ABGYG345-06|ROM 115981|Guyana.Potaro-Siparuni
Platyrrhinus aurarius|ABGYG346-06|ROM 115982|Guyana.Potaro-Siparuni
Platyrrhinus aurarius|BCBNC160-06|ROM 115927|Guyana.Potaro-Siparuni
Platyrrhinus aurarius|BCBNC200-06|ROM 116701|Guyana.Potaro-Siparuni
Platyrrhinus aurarius|BCBNC148-06|ROM 115867|Guyana.Potaro-Siparuni
Platyrrhinus aurarius|BCBNC151-06|ROM 115873|Guyana.Potaro-Siparuni
Platyrrhinus aurarius|BCBNT990-06|ROM 114660|Guyana.Potaro-Siparuni
Platyrrhinus aurarius|BCBNC011-06|ROM 114702|Guyana.Potaro-Siparuni
Platyrrhinus aurarius|BCBNC109-06|ROM 115752|Guyana.Potaro-Siparuni
Platyrrhinus aurarius|BCBNC169-06|ROM 115973|Guyana.Potaro-Siparuni
Platyrrhinus aurarius|ABGYG356-06|ROM 115992|Guyana.Potaro-Siparuni
Platyrrhinus aurarius|BCBNC170-06|ROM 115974|Guyana.Potaro-Siparuni
Platyrrhinus aurarius|ABGYG310-06|ROM 115935|Guyana.Potaro-Siparuni
Platyrrhinus aurarius|ABGYG354-06|ROM 115990|Guyana.Potaro-Siparuni
Platyrrhinus aurarius|BCBNC161-06|ROM 115928|Guyana.Potaro-Siparuni
Platyrrhinus aurarius|BCBNC171-06|ROM 115975|Guyana.Potaro-Siparuni
Platyrrhinus aurarius|BCBNT996-06|ROM 114679|Guyana.Potaro-Siparuni
Sturnira lilium|ABSA116-06|ROM 111164|Brazil.Sao Paulo
Sturnira lilium|BCBNT466-06|ROM 111065|Brazil.Sao Paulo
Sturnira lilium|BCBNT475-06|ROM 111081|Brazil.Sao Paulo
Sturnira lilium|BCBNT458-06|ROM 111052|Brazil.Sao Paulo
Sturnira lilium|ABSA061-06|ROM 111069|Brazil.Sao Paulo
Sturnira lilium|ABSA111-06|ROM 111157|Brazil.Sao Paulo
Sturnira lilium|BCBNT465-06|ROM 111064|Brazil.Sao Paulo
Sturnira lilium|BCBNT501-06|ROM 111148|Brazil.Sao Paulo
Sturnira lilium|ABMXA744-06|ROM FN30408|Mexico.Yucatan
Sturnira lilium|ABCSA306-06|ROM 99284|Guatemala.Peten
Sturnira lilium|ABCSA496-06|ROM 99490|Guatemala.Peten
Sturnira lilium|ABCSA506-06|ROM 99512|Guatemala.Peten
Sturnira lilium|ABCSA206-06|ROM 98475|Guatemala.El Progreso
Sturnira lilium|ABCSA412-06|ROM 99432|Guatemala.Peten
Sturnira lilium|ABCSA550-06|ROM 99694|Guatemala.El Progreso
Sturnira lilium|ABCSA546-06|ROM 99690|Guatemala.El Progreso
Sturnira lilium|ABCSA291-06|ROM 99267|Guatemala.Peten
Sturnira lilium|ABCSA507-06|ROM 99513|Guatemala.Peten
Sturnira lilium|BCBN027-05|ROM 96276|Mexico.Campeche
Sturnira lilium|ABCSA549-06|ROM 99693|Guatemala.El Progreso
Sturnira lilium|BCBN408-05|ROM 101301|El Salvador.Ahuachapan
Sturnira lilium|ABCSA508-06|ROM 99514|Guatemala.Peten
Sturnira lilium|ABCSA464-06|ROM 99618|Guatemala.Peten
Sturnira lilium|ABMXC527-06|ROM 97451|Mexico.Quintana Roo
Sturnira lilium|ABCSA377-06|ROM 99391|Guatemala.Peten
Sturnira lilium|ABCSA152-06|ROM 98399|Guatemala.El Progreso
Sturnira lilium|ABCSA355-06|ROM 99338|Guatemala.Peten
Sturnira lilium|BCBN026-05|ROM 96258|Mexico.Campeche
Sturnira lilium|ABCSA462-06|ROM 99616|Guatemala.Peten
Sturnira lilium|ABCSA683-06|ROM 101225|El Salvador.Ahuachapan
Sturnira lilium|ABCSA203-06|ROM 98472|Guatemala.El Progreso
Sturnira lilium|ABCSA687-06|ROM 101237|El Salvador.Ahuachapan
Sturnira lilium|BCBN229-05|ROM 99509|Guatemala.Peten
Sturnira lilium|BCBN230-05|ROM 99510|Guatemala.Peten
Sturnira lilium|BCBN272-05|ROM 99789|Guatemala.Solola
Sturnira lilium|BCBN425-05|ROM 101340|El Salvador.Ahuachapan
Sturnira lilium|ABCSA704-06|ROM 101266|El Salvador.Ahuachapan
Sturnira lilium|ABCSA292-06|ROM 99268|Guatemala.Peten
Sturnira lilium|ABCSA310-06|ROM 99288|Guatemala.Peten
Sturnira lilium|ABCSA311-06|ROM 99289|Guatemala.Peten
Sturnira lilium|ABCSA465-06|ROM 99619|Guatemala.Peten
Sturnira lilium|BCBN480-05|ROM 97413|Mexico.Quintana Roo
Sturnira lilium|ABCSA548-06|ROM 99692|Guatemala.El Progreso
Sturnira lilium|ABCSA581-06|ROM 99787|Guatemala.Solola
Sturnira lilium|ABCSA308-06|ROM 99286|Guatemala.Peten
Sturnira lilium|ABCSA466-06|ROM 99620|Guatemala.Peten
Sturnira lilium|ABMXA749-06|ROM FN30421|Mexico.Yucatan
Sturnira lilium|ABCSA307-06|ROM 99285|Guatemala.Peten
Sturnira lilium|ABCSA262-06|ROM 99223|Guatemala.Peten
Sturnira lilium|BCBN409-05|ROM 101302|El Salvador.Ahuachapan

Sturnira lilium|ABCSA307-06|ROM 99285|Guatemala.Peten
Sturnira lilium|ABCSA262-06|ROM 99223|Guatemala.Peten
Sturnira lilium|BCBN409-05|ROM 101302|El Salvador.Ahuachapan
Sturnira lilium|ABMXC526-06|ROM 97450|Mexico.Quintana Roo
Sturnira lilium|ABMXC525-06|ROM 97449|Mexico.Quintana Roo
Sturnira lilium|ABMXC513-06|ROM 97429|Mexico.Quintana Roo
Sturnira lilium|ABMXC229-06|ROM 96263|Mexico.Campeche
Sturnira lilium|ABMXA966-06|ROM FN32530|Mexico.Quintana Roo
Sturnira lilium|ABMXA924-06|ROM FN33833|Mexico.Quintana Roo
Sturnira lilium|ABCSA684-06|ROM 101226|El Salvador.Ahuachapan
Sturnira lilium|ABCSA682-06|ROM 101224|El Salvador.Ahuachapan
Sturnira lilium|ABCSA582-06|ROM 99788|Guatemala.Solola
Sturnira lilium|ABCSA547-06|ROM 99691|Guatemala.El Progreso
Sturnira lilium|ABCSA505-06|ROM 99511|Guatemala.Peten
Sturnira lilium|ABCSA495-06|ROM 99489|Guatemala.Peten
Sturnira lilium|ABCSA356-06|ROM 99339|Guatemala.Peten
Sturnira lilium|ABCSA354-06|ROM 99337|Guatemala.Peten
Sturnira lilium|ABCSA353-06|ROM 99336|Guatemala.Peten
Sturnira lilium|ABCSA309-06|ROM 99287|Guatemala.Peten
Sturnira lilium|ABCSA205-06|ROM 98474|Guatemala.El Progreso
Sturnira lilium|ABCSA204-06|ROM 98473|Guatemala.El Progreso
Sturnira lilium|ABCSA153-06|ROM 98400|Guatemala.El Progreso
Sturnira lilium|ABCSA151-06|ROM 98398|Guatemala.El Progreso
Sturnira lilium|ABCSA150-06|ROM FN31386|Guatemala.El Progreso
Sturnira lilium|ABCSA149-06|ROM 98396|Guatemala.El Progreso
Sturnira lilium|ABMXA927-06|ROM FN33836|Mexico.Quintana Roo
Sturnira lilium|ABCSA290-06|ROM 99266|Guatemala.Peten
Sturnira lilium|ABCSA725-06|ROM 101303|El Salvador.Ahuachapan
Sturnira lilium|BCBN223-05|ROM 99487|Guatemala.Peten
Sturnira lilium|BCBN224-05|ROM 99488|Guatemala.Peten
Sturnira lilium|BCBN374-05|ROM 101223|El Salvador.Ahuachapan
Sturnira lilium|BCBN424-05|ROM 101339|El Salvador.Ahuachapan
Sturnira lilium|BCBN479-05|ROM 97412|Mexico.Quintana Roo
Sturnira lilium|BCBN481-05|ROM 97414|Mexico.Quintana Roo
Sturnira lilium|ABECA073-06|ROM 104068|Ecuador.Napo
Sturnira lilium|ABECB036-06|ROM F40512|Ecuador.Napo
Sturnira lilium|ABECB041-06|ROM F40518|Ecuador.Napo
Sturnira lilium|ABECA787-06|ROM 105951|Ecuador.Napo
Sturnira lilium|ABECA788-06|ROM F40203|Ecuador.Napo
Sturnira lilium|ABECA074-06|ROM F37179|Ecuador.Napo
Sturnira lilium|ABECB054-06|ROM F40531|Ecuador.Napo
Sturnira lilium|ABECA363-06|ROM F37578|Ecuador.Napo
Sturnira lilium|BCBN731-05|ROM 104416|Ecuador.Napo
Sturnira lilium|ABECA664-06|ROM 105727|Ecuador.Napo
Sturnira lilium|ABECA665-06|ROM 105728|Ecuador.Napo
Sturnira lilium|ABECA746-06|ROM 105894|Ecuador.Napo
Sturnira lilium|BCBN882-05|ROM 105694|Ecuador.Napo
Sturnira lilium|ABECA302-06|ROM 105174|Ecuador.Napo
Sturnira lilium|ABECA556-06|ROM 105585|Ecuador.Napo
Sturnira lilium|ABECA731-06|ROM 105875|Ecuador.Napo
Sturnira lilium|ABSMS465-06|ROM 117627|Suriname.Sipaliwini
Sturnira lilium|ABGYB225-06|ROM 102927|Guyana.Upper Takutu-Upper Essequibo
Sturnira lilium|ABGYB531-06|ROM 103266|Guyana.Upper Takutu-Upper Essequibo
Sturnira lilium|ABGYB580-06|ROM 103316|Guyana.Upper Takutu-Upper Essequibo
Sturnira lilium|ABECA075-06|ROM F37180|Ecuador.Napo
Sturnira lilium|ABECA641-06|ROM 105695|Ecuador.Napo
Sturnira lilium|ABECA907-06|ROM 106089|Ecuador.Napo
Sturnira lilium|BCBN724-05|ROM 104395|Ecuador.Napo
Sturnira lilium|ABECA353-06|ROM 105245|Ecuador.Napo
Sturnira lilium|ABECA799-06|ROM 105965|Ecuador.Napo
Sturnira lilium|ABECA909-06|ROM 106091|Ecuador.Napo
Sturnira lilium|BCBN812-05|ROM 105269|Ecuador.Napo
Sturnira lilium|ABECA005-06|ROM 103987|Ecuador.Napo
Sturnira lilium|ABECA193-06|ROM 104511|Ecuador.Napo
Sturnira lilium|BCBN885-05|ROM 105706|Ecuador.Napo
Sturnira lilium|ABGYA042-06|ROM 97833|Guyana.Upper Takutu-Upper Essequibo
Sturnira lilium|ABGYA107-06|ROM 97946|Guyana.Upper Takutu-Upper Essequibo
Sturnira lilium|ABGYB306-06|ROM 103011|Guyana.Upper Takutu-Upper Essequibo
Sturnira lilium|ABGYB374-06|ROM 103081|Guyana.Upper Takutu-Upper Essequibo
Sturnira lilium|ABGYC225-06|ROM 106770|Guyana.Upper Takutu-Upper Essequibo
Sturnira lilium|ABSMS435-06|ROM 117597|Suriname.Sipaliwini
Sturnira lilium|ABGYB149-06|ROM 101137|Guyana.Barima-Waini
Sturnira lilium|ABGYA039-06|ROM 97830|Guyana.Upper Takutu-Upper Essequibo
Sturnira lilium|ABGYA111-06|ROM 97950|Guyana.Upper Takutu-Upper Essequibo
Sturnira lilium|ABGYA452-06|ROM 98862|Guyana.Barima-Waini
Sturnira lilium|ABECB034-06|ROM 106346|Ecuador.Napo
Sturnira lilium|ABECA911-06|ROM 106093|Ecuador.Napo
Sturnira lilium|ABECB053-06|ROM F40530|Ecuador.Napo
Sturnira lilium|ABGYA303-06|ROM 98699|Guyana.Barima-Waini
Sturnira lilium|ABGYD051-06|ROM 108274|Guyana.Cuyuni-Mazaruni
Sturnira lilium|ABECA274-06|ROM 105143|Ecuador.Napo
Sturnira lilium|ABSMS480-06|ROM 117642|Suriname.Sipaliwini
Sturnira lilium|ABECB035-06|ROM 106347|Ecuador.Napo
Sturnira lilium|ABECA191-06|ROM F37356|Ecuador.Napo
Sturnira lilium|ABECA364-06|ROM 105256|Ecuador.Napo
Sturnira lilium|ABGYB488-06|ROM 103223|Guyana.Upper Takutu-Upper Essequibo
Sturnira lilium|ABGYF129-06|ROM 113676|Guyana.Demerara-Mahaica
Sturnira lilium|BCBN364-05|ROM 101121|Guyana.Barima-Waini
Sturnira lilium|ABGYA040-06|ROM 97831|Guyana.Upper Takutu-Upper Essequibo
Sturnira lilium|ABGYB573-06|ROM 103309|Guyana.Upper Takutu-Upper Essequibo
Sturnira lilium|BCBNT221-06|ROM 107936|Venezuela.Bolivar
Sturnira lilium|ABSMS430-06|ROM 117592|Suriname.Sipaliwini
Sturnira lilium|ABGYB405-06|ROM 103112|Guyana.Upper Takutu-Upper Essequibo
Sturnira lilium|ABGYB409-06|ROM 103116|Guyana.Upper Takutu-Upper Essequibo
Sturnira lilium|ABGYD019-06|ROM 108241|Guyana.Cuyuni-Mazaruni
Sturnira lilium|ABGYD020-06|ROM F43291|Guyana.Cuyuni-Mazaruni
Sturnira lilium|ABGYD036-06|ROM 108258|Guyana.Cuyuni-Mazaruni
Sturnira lilium|ABGYD284-06|ROM 108621|Guyana.Potaro-Siparuni
Sturnira lilium|ABGYF144-06|ROM 113695|Guyana.Demerara-Mahaica
Sturnira lilium|ABGYF296-06|ROM 114663|Guyana.Potaro-Siparuni
Sturnira lilium|ABGYB454-06|ROM 103169|Guyana.Upper Takutu-Upper Essequibo
Sturnira lilium|ABGYB455-06|ROM 103170|Guyana.Upper Takutu-Upper Essequibo
Sturnira lilium|ABGYB486-06|ROM 103221|Guyana.Upper Takutu-Upper Essequibo

[illegible]
Sturnira lilium|ABGYB454-06|ROM 103169|Guyana.Upper Takutu-Upper Essequibo
Sturnira lilium|ABGYB455-06|ROM 103170|Guyana.Upper Takutu-Upper Essequibo
Sturnira lilium|ABGYB486-06|ROM 103221|Guyana.Upper Takutu-Upper Essequibo
Sturnira lilium|ABGYB570-06|ROM 103306|Guyana.Upper Takutu-Upper Essequibo
Sturnira lilium|ABSMS464-06|ROM 117626|Suriname.Sipaliwini
Sturnira lilium|ABSRA500-06|ROM 117090|Suriname.Nickerie
Sturnira lilium|ABGYB571-06|ROM 103307|Guyana.Upper Takutu-Upper Essequibo
Sturnira lilium|ABGYB578-06|ROM 103314|Guyana.Upper Takutu-Upper Essequibo
Sturnira lilium|ABGYC223-06|ROM 106768|Guyana.Upper Takutu-Upper Essequibo
Sturnira lilium|ABGYC736-06|ROM 108136|Guyana.Cuyuni-Mazaruni
Sturnira lilium|BCBN373-05|ROM 101169|Guyana.Barima-Waini
Sturnira lilium|BCBNT831-06|ROM 114180|Suriname.Brokopondo
Sturnira lilium|ABSMS322-06|ROM 117484|Suriname.Sipaliwini
Sturnira lilium|ABGYB371-06|ROM 103078|Guyana.Upper Takutu-Upper Essequibo
Sturnira lilium|ABGYB241-06|ROM 102943|Guyana.Upper Takutu-Upper Essequibo
Sturnira lilium|ABGYB177-06|ROM 101170|Guyana.Barima-Waini
Sturnira lilium|ABGYB176-06|ROM 101168|Guyana.Barima-Waini
Sturnira lilium|ABGYB160-06|ROM 101149|Guyana.Barima-Waini
Sturnira lilium|ABGYB150-06|ROM 101138|Guyana.Barima-Waini
Sturnira lilium|ABGYB029-06|ROM 101001|Guyana.Barima-Waini
Sturnira lilium|ABGYA304-06|ROM 98700|Guyana.Barima-Waini
Sturnira lilium|ABECA362-06|ROM 105255|Ecuador.Napo
Sturnira lilium|ABGYB784-06|ROM 103552|Guyana.Upper Demerara-Berbice
Sturnira lilium|ABECA192-06|ROM 104510|Ecuador.Napo
Sturnira lilium|ABECB055-06|ROM 106356|Ecuador.Napo
Sturnira lilium|ABECA786-06|ROM 105950|Ecuador.Napo
Sturnira lilium|ABECA187-06|ROM 104504|Ecuador.Napo
Sturnira lilium|ABGYB450-06|ROM 103165|Guyana.Upper Takutu-Upper Essequibo
Sturnira lilium|ABGYA057-06|ROM 97864|Guyana.Upper Takutu-Upper Essequibo
Sturnira lilium|ABGYB451-06|ROM 103166|Guyana.Upper Takutu-Upper Essequibo
Sturnira lilium|ABGYB456-06|ROM 103171|Guyana.Upper Takutu-Upper Essequibo
Sturnira lilium|ABSMS408-06|ROM 117570|Suriname.Sipaliwini
Sturnira lilium|BCBNT832-06|ROM 114181|Suriname.Brokopondo
Sturnira lilium|ABECA530-06|ROM F37799|Ecuador.Napo
Sturnira lilium|ABGYB530-06|ROM 103265|Guyana.Upper Takutu-Upper Essequibo
Sturnira lilium|BCBN609-05|ROM 104204|Panama.Canal Zone
Sturnira lilium|BCBN703-05|ROM 104349|Panama.Darien
Sturnira lilium|ABGYB053-06|ROM 101025|Guyana.Barima-Waini
Sturnira lilium|ABSCA110-06|ROM F38205|Panama.Darien
Sturnira lilium|BCBN702-05|ROM 104348|Panama.Darien
Sturnira lilium|BCBN706-05|ROM 104359|Panama.Darien
Sturnira lilium|BCBN930-05|ROM 105807|Ecuador.Esmeraldas
Sturnira lilium|ABGYB487-06|ROM 103222|Guyana.Upper Takutu-Upper Essequibo
Sturnira lilium|ABGYC203-06|ROM 106742|Guyana.Upper Takutu-Upper Essequibo
Sturnira lilium|ABGYA056-06|ROM 97863|Guyana.Upper Takutu-Upper Essequibo
Sturnira lilium|ABGYB383-06|ROM 103090|Guyana.Upper Takutu-Upper Essequibo
Sturnira lilium|ABGYB404-06|ROM 103111|Guyana.Upper Takutu-Upper Essequibo
Sturnira lilium|ABGYB406-06|ROM 103113|Guyana.Upper Takutu-Upper Essequibo
Sturnira lilium|ABGYB453-06|ROM 103168|Guyana.Upper Takutu-Upper Essequibo
Sturnira lilium|ABGYB572-06|ROM 103308|Guyana.Upper Takutu-Upper Essequibo
Sturnira lilium|ABGYB577-06|ROM 103313|Guyana.Upper Takutu-Upper Essequibo
Sturnira lilium|ABSMS463-06|ROM 117625|Suriname.Sipaliwini
Sturnira lilium|ABGYB335-06|ROM 103042|Guyana.Upper Takutu-Upper Essequibo
Sturnira lilium|ABGYD472-06|ROM 108823|Guyana.Potaro-Siparuni
Sturnira lilium|ABGYB190-06|ROM 102892|Guyana.Upper Takutu-Upper Essequibo
Sturnira lilium|ABGYA075-06|ROM 97897|Guyana.Upper Takutu-Upper Essequibo
Sturnira lilium|ABGYA108-06|ROM 97947|Guyana.Upper Takutu-Upper Essequibo
Sturnira lilium|ABGYA109-06|ROM 97948|Guyana.Upper Takutu-Upper Essequibo
Sturnira lilium|ABGYB307-06|ROM 103012|Guyana.Upper Takutu-Upper Essequibo
Sturnira lilium|ABGYB370-06|ROM 103077|Guyana.Upper Takutu-Upper Essequibo
Sturnira lilium|ABGYB373-06|ROM 103080|Guyana.Upper Takutu-Upper Essequibo
Sturnira lilium|ABGYB407-06|ROM 103114|Guyana.Upper Takutu-Upper Essequibo
Sturnira lilium|ABGYB408-06|ROM 103115|Guyana.Upper Takutu-Upper Essequibo
Sturnira lilium|ABGYB575-06|ROM 103311|Guyana.Upper Takutu-Upper Essequibo
Sturnira lilium|ABGYB576-06|ROM 103312|Guyana.Upper Takutu-Upper Essequibo
Sturnira lilium|ABGYB579-06|ROM 103315|Guyana.Upper Takutu-Upper Essequibo
Sturnira lilium|ABGYC226-06|ROM 106771|Guyana.Upper Takutu-Upper Essequibo
Sturnira lilium|ABGYF249-06|ROM 113834|Guyana.Demerara-Mahaica
Sturnira lilium|BCBN368-05|ROM 101136|Guyana.Barima-Waini
Sturnira lilium|BCBN370-05|ROM 101153|Guyana.Barima-Waini
Sturnira lilium|BCBN371-05|ROM 101154|Guyana.Barima-Waini
Sturnira lilium|ABGYA074-06|ROM 97896|Guyana.Upper Takutu-Upper Essequibo
Sturnira lilium|ABGYD432-06|ROM 108783|Guyana.Potaro-Siparuni
Sturnira lilium|ABGYA041-06|ROM 97832|Guyana.Upper Takutu-Upper Essequibo
Sturnira lilium|ABGYA110-06|ROM 97949|Guyana.Upper Takutu-Upper Essequibo
Sturnira lilium|ABGYB457-06|ROM 103172|Guyana.Upper Takutu-Upper Essequibo
Sturnira lilium|ABGYB191-06|ROM 102893|Guyana.Upper Takutu-Upper Essequibo
Sturnira lilium|ABGYB305-06|ROM 103010|Guyana.Upper Takutu-Upper Essequibo
Sturnira lilium|ABGYA073-06|ROM 97895|Guyana.Upper Takutu-Upper Essequibo
Sturnira lilium|ABGYA055-06|ROM 97862|Guyana.Upper Takutu-Upper Essequibo
Sturnira lilium|ABGYB372-06|ROM 103079|Guyana.Upper Takutu-Upper Essequibo
Sturnira lilium|ABGYB379-06|ROM 103086|Guyana.Upper Takutu-Upper Essequibo
Sturnira lilium|ABGYB452-06|ROM 103167|Guyana.Upper Takutu-Upper Essequibo
Sturnira lilium|ABGYB532-06|ROM 103267|Guyana.Upper Takutu-Upper Essequibo
Sturnira lilium|ABGYB533-06|ROM 103268|Guyana.Upper Takutu-Upper Essequibo
Sturnira lilium|ABGYB534-06|ROM 103269|Guyana.Upper Takutu-Upper Essequibo
Sturnira lilium|ABGYB574-06|ROM 103310|Guyana.Upper Takutu-Upper Essequibo
Sturnira lilium|ABGYB587-06|ROM 103328|Guyana.Upper Takutu-Upper Essequibo
Sturnira lilium|ABGYC224-06|ROM 106769|Guyana.Upper Takutu-Upper Essequibo
Sturnira lilium|ABGYC236-06|ROM 106786|Guyana.Upper Takutu-Upper Essequibo
Sturnira lilium|ABSMS412-06|ROM 117574|Suriname.Sipaliwini
Sturnira lilium|ABSRA276-06|ROM 116865|Suriname.Nickerie
Sturnira lilium|BCBNT802-06|ROM 114078|Suriname.Brokopondo
Sturnira lilium|BCBNT829-06|ROM 114178|Suriname.Brokopondo
Sturnira lilium|BCBNT830-06|ROM 114179|Suriname.Brokopondo
Sturnira lilium|ABGYC801-06|ROM 108200|Guyana.Cuyuni-Mazaruni
Sturnira lilium|ABGYA423-06|ROM 98831|Guyana.Barima-Waini
Sturnira lilium|BCBNT903-06|ROM 115545|Guyana.Essequibo Islands-West Demerara
Sturnira magna|ABECA797-06|ROM 105962|Ecuador.Napo
Sturnira magna|ABECA200-06|ROM F37369|Ecuador.Napo
Sturnira magna|ABECA789-06|ROM 105952|Ecuador.Napo
Sturnira magna|ABECA539-06|ROM 105567|Ecuador.Napo
[illegible]

Sturnira magna|ABECA200-06|ROM F37[illegible]|Ecuador.Napo
Sturnira magna|ABECA789-06|ROM 105952|Ecuador.Napo
Sturnira magna|ABECA539-06|ROM 105567|Ecuador.Napo
Sturnira magna|ABECB032-06|ROM 106345|Ecuador.Napo
Sturnira magna|ABECB056-06|ROM F40533|Ecuador.Napo
Sturnira magna|ABECA773-06|ROM 105932|Ecuador.Napo
Sturnira magna|ABECA931-06|ROM 106114|Ecuador.Napo
Sturnira magna|ABECA348-06|ROM 105236|Ecuador.Napo
Sturnira magna|BCBN860-05|ROM 105622|Ecuador.Napo
Sturnira magna|ABECB076-08|ROM 118738|Ecuador
Sturnira magna|ABECA319-06|ROM 105199|Ecuador.Napo
Sturnira magna|BCBN876-05|ROM 105683|Ecuador.Napo
Sturnira magna|ABECA057-06|ROM 104043|Ecuador.Napo
Sturnira magna|BCBN565-05|ROM 104001|Ecuador.Napo
Sturnira magna|BCBN759-05|ROM 104515|Ecuador.Napo
Sturnira magna|ABECA294-06|ROM F37480|Ecuador.Napo
Sturnira magna|ABECA347-06|ROM 105235|Ecuador.Napo
Sturnira magna|BCBN803-05|ROM 105234|Ecuador.Napo
Sturnira magna|ABECA702-06|ROM 105763|Ecuador.Napo
Sturnira magna|ABECA663-06|ROM 105725|Ecuador.Napo
Sturnira magna|ABECA199-06|ROM 104521|Ecuador.Napo
Sturnira magna|ABECA651-06|ROM 105705|Ecuador.Napo
Sturnira magna|BCBN758-05|ROM 104514|Ecuador.Napo
Sturnira magna|ABECA662-06|ROM F37974|Ecuador.Napo
Sturnira magna|ABECA300-06|ROM 105171|Ecuador.Napo
Sturnira magna|ABECA205-06|ROM 104526|Ecuador.Napo
Sturnira magna|ABECA019-06|ROM F37087|Ecuador.Napo
Sturnira magna|ABECA696-06|ROM F40049|Ecuador.Napo
Sturnira magna|ABECA774-06|ROM 105933|Ecuador.Napo
Sturnira magna|ABECA793-06|ROM 105958|Ecuador.Napo
Sturnira magna|ABECB031-06|ROM 106344|Ecuador.Napo
Sturnira magna|ABECB033-06|ROM F40509|Ecuador.Napo
Sturnira magna|BCBN564-05|ROM 104000|Ecuador.Napo
Sturnira magna|BCBN761-05|ROM 104520|Ecuador.Napo
Sturnira magna|BCBN802-05|ROM 105223|Ecuador.Napo
Sturnira magna|BCBN964-05|ROM 105957|Ecuador.Napo
Sturnira ludovici|ABCSA761-06|ROM 101366|El Salvador.Santa Ana
Sturnira ludovici|ABCSA207-06|ROM 98476|Guatemala.El Progreso
Sturnira ludovici|ABCSA796-06|ROM 101402|El Salvador.Santa Ana
Sturnira ludovici|BCBN271-05|ROM 99786|Guatemala.Solola
Sturnira ludovici|BCBN273-05|ROM 99830|Guatemala.Zacapa
Sturnira ludovici|ABCSA208-06|ROM 98477|Guatemala.El Progreso
Sturnira ludovici|ABCSA815-06|ROM 101434|El Salvador.Santa Ana
Sturnira ludovici|ABCSA843-06|ROM 101473|El Salvador.Santa Ana
Sturnira ludovici|BCBN278-05|ROM 99839|Guatemala.Zacapa
Sturnira ludovici|ABCSA810-06|ROM 101421|El Salvador.Santa Ana
Sturnira ludovici|ABCSA842-06|ROM 101469|El Salvador.Santa Ana
Sturnira ludovici|BCBN454-05|ROM 101461|El Salvador.Santa Ana
Sturnira ludovici|ABCSA807-06|ROM 101418|El Salvador.Santa Ana
Sturnira ludovici|BCBN438-05|ROM 101403|El Salvador.Santa Ana
Sturnira ludovici|ABCSA808-06|ROM 101419|El Salvador.Santa Ana
Sturnira ludovici|ABCSA209-06|ROM FN31467|Guatemala.El Progreso
Sturnira ludovici|ABCSA809-06|ROM 101420|El Salvador.Santa Ana
Sturnira ludovici|BCBN270-05|ROM 99785|Guatemala.Solola
Sturnira ludovici|BCBN442-05|ROM 101417|El Salvador.Santa Ana
Sturnira ludovici|BCBN455-05|ROM 101462|El Salvador.Santa Ana
Sturnira ludovici|ABCSA811-06|ROM 101422|El Salvador.Santa Ana
Sturnira ludovici|ABCSA844-06|ROM 101474|El Salvador.Santa Ana
Sturnira ludovici|BCBN059-05|ROM 98258|Guatemala.Sacatepequez
Sturnira ludovici|BCBN458-05|ROM 101468|El Salvador.Santa Ana
Sturnira ludovici|BCBN670-05|ROM 104293|Panama.Chiriqui
Sturnira ludovici|ABSCA082-06|ROM F38146|Panama.Chiriqui
Sturnira ludovici|BCBN671-05|ROM 104294|Panama.Chiriqui
Sturnira ludovici|BCBN672-05|ROM 104295|Panama.Chiriqui
Sturnira tildae|BCBN541-05|ROM 103366|Guyana.Upper Demerara-Berbice
Sturnira tildae|BCBNT726-06|ROM 113897|Suriname.Brokopondo
Sturnira tildae|ABGYE587-06|ROM 112064|Guyana.Potaro-Siparuni
Sturnira tildae|ABGYD524-06|ROM 108882|Guyana.Potaro-Siparuni
Sturnira tildae|ABGYC414-06|ROM 107236|Guyana.Potaro-Siparuni
Sturnira tildae|ABGYB446-06|ROM 103161|Guyana.Upper Takutu-Upper Essequibo
Sturnira tildae|ABSMS392-06|ROM 117554|Suriname.Sipaliwini
Sturnira tildae|ABGYG161-06|ROM 115723|Guyana.Potaro-Siparuni
Sturnira tildae|ABSMS264-06|ROM 117426|Suriname.Sipaliwini
Sturnira tildae|BCBNT803-06|ROM 114098|Suriname.Brokopondo
Sturnira tildae|BCBNT804-06|ROM 114099|Suriname.Brokopondo
Sturnira tildae|ABGYD623-06|ROM 109000|Guyana.Potaro-Siparuni
Sturnira tildae|ABGYD842-06|ROM 109269|Guyana.Potaro-Siparuni
Sturnira tildae|ABGYF353-06|ROM 114763|Guyana.Potaro-Siparuni
Sturnira tildae|ABGYD841-06|ROM 109268|Guyana.Potaro-Siparuni
Sturnira tildae|ABGYC255-06|ROM 107041|Guyana.Potaro-Siparuni
Sturnira tildae|ABGYF351-06|ROM 114761|Guyana.Potaro-Siparuni
Sturnira tildae|ABGYB485-06|ROM 103220|Guyana.Upper Takutu-Upper Essequibo
Sturnira tildae|ABGYD624-06|ROM 109001|Guyana.Potaro-Siparuni
Sturnira tildae|ABGYD796-06|ROM 109218|Guyana.Potaro-Siparuni
Sturnira tildae|ABGYE375-06|ROM 111827|Guyana.Potaro-Siparuni
Sturnira tildae|ABGYE532-06|ROM 112005|Guyana.Potaro-Siparuni
Sturnira tildae|BCBNT930-06|ROM 115620|Guyana.Essequibo Islands-West Demerara
Sturnira tildae|ABGYE171-06|ROM 111614|Guyana.Potaro-Siparuni
Sturnira tildae|ABGYD419-06|ROM 108768|Guyana.Potaro-Siparuni
Sturnira tildae|ABGYD527-06|ROM 108885|Guyana.Potaro-Siparuni
Sturnira tildae|ABSRA305-06|ROM 116894|Suriname.Nickerie
Sturnira tildae|ABGYD788-06|ROM 109210|Guyana.Potaro-Siparuni
Sturnira tildae|ABGYF350-06|ROM 114760|Guyana.Potaro-Siparuni
Sturnira tildae|BCBNC016-06|ROM 114722|Guyana.Potaro-Siparuni
Sturnira tildae|ABGYA154-06|ROM 98005|Guyana.Potaro-Siparuni
Sturnira tildae|ABGYC388-06|ROM 107207|Guyana.Potaro-Siparuni
Sturnira tildae|ABGYE529-06|ROM 112002|Guyana.Potaro-Siparuni
Sturnira tildae|ABSMS443-06|ROM 117605|Suriname.Sipaliwini
Sturnira tildae|ABGYE561-06|ROM 112034|Guyana.Potaro-Siparuni
Sturnira tildae|ABGYE531-06|ROM 112004|Guyana.Potaro-Siparuni
Sturnira tildae|ABGYE343-06|ROM 111793|Guyana.Potaro-Siparuni
Sturnira tildae|ABGYD492-06|ROM 108849|Guyana.Potaro-Siparuni
Sturnira tildae|ABGYC719-06|ROM 108119|Guyana.Cuyuni-Mazaruni

Sturnira tildae|ABGYE343-06|ROM 111793|Guyana.Potaro-Siparuni
Sturnira tildae|ABGYD492-06|ROM 108849|Guyana.Potaro-Siparuni
Sturnira tildae|ABGYC719-06|ROM 108119|Guyana.Cuyuni-Mazaruni
Sturnira tildae|ABGYC254-06|ROM 107040|Guyana.Potaro-Siparuni
Sturnira tildae|ABGYC253-06|ROM 107039|Guyana.Potaro-Siparuni
Sturnira tildae|ABGYE510-06|ROM 111982|Guyana.Potaro-Siparuni
Sturnira tildae|ABGYB483-06|ROM 103218|Guyana.Upper Takutu-Upper Essequibo
Sturnira tildae|ABGYB448-06|ROM 103163|Guyana.Upper Takutu-Upper Essequibo
Sturnira tildae|ABGYB465-06|ROM 103199|Guyana.Upper Takutu-Upper Essequibo
Sturnira tildae|ABGYE827-06|ROM 113435|Guyana.Upper Demerara-Berbice
Sturnira tildae|BCBNC127-06|ROM 115793|Guyana.Potaro-Siparuni
Sturnira tildae|ABSRA343-06|ROM 116932|Suriname.Nickerie
Sturnira tildae|BCBN350-05|ROM 100982|Guyana.Barima-Waini
Sturnira tildae|BCBNC110-06|ROM 115753|Guyana.Potaro-Siparuni
Sturnira tildae|BCBNT030-06|ROM 106603|Guyana.Upper Takutu-Upper Essequibo
Sturnira tildae|BCBNT067-06|ROM 106767|Guyana.Upper Takutu-Upper Essequibo
Sturnira tildae|BCBNT325-06|ROM 108846|Guyana.Potaro-Siparuni
Sturnira tildae|BCBNT595-06|ROM 113437|Guyana.Upper Demerara-Berbice
Sturnira tildae|BCBNT598-06|ROM 113451|Guyana.Upper Demerara-Berbice
Sturnira tildae|BCBNT976-06|ROM 114629|Guyana.Potaro-Siparuni
Sturnira tildae|ABGYA155-06|ROM 98006|Guyana.Potaro-Siparuni
Sturnira tildae|ABGYC277-06|ROM 107068|Guyana.Potaro-Siparuni
Sturnira tildae|ABGYC436-06|ROM 107260|Guyana.Potaro-Siparuni
Sturnira tildae|ABGYD357-06|ROM 108699|Guyana.Potaro-Siparuni
Sturnira tildae|ABGYD525-06|ROM 108883|Guyana.Potaro-Siparuni
Sturnira tildae|ABGYE045-06|ROM 109331|Guyana.Potaro-Siparuni
Sturnira tildae|ABGYE062-06|ROM 109348|Guyana.Potaro-Siparuni
Sturnira tildae|BCBNT255-06|ROM 108208|Guyana.Cuyuni-Mazaruni
Sturnira tildae|BCBNT323-06|ROM 108830|Guyana.Potaro-Siparuni
Sturnira tildae|BCBNT977-06|ROM 114630|Guyana.Potaro-Siparuni
Sturnira tildae|BCBNT253-06|ROM 108195|Guyana.Cuyuni-Mazaruni
Sturnira tildae|BCBNC010-06|ROM 114701|Guyana.Potaro-Siparuni
Sturnira tildae|ABGYE159-06|ROM 111602|Guyana.Potaro-Siparuni
Sturnira tildae|ABGYF328-06|ROM 114719|Guyana.Potaro-Siparuni
Sturnira tildae|ABGYG210-06|ROM 115801|Guyana.Potaro-Siparuni
Sturnira tildae|ABGYD038-06|ROM 108260|Guyana.Cuyuni-Mazaruni
Sturnira tildae|ABGYE281-06|ROM 111730|Guyana.Potaro-Siparuni
Sturnira tildae|ABGYB449-06|ROM 103164|Guyana.Upper Takutu-Upper Essequibo
Sturnira tildae|ABGYC303-06|ROM 107113|Guyana.Potaro-Siparuni
Sturnira tildae|ABGYE309-06|ROM 111759|Guyana.Potaro-Siparuni
Sturnira tildae|ABGYE358-06|ROM 111808|Guyana.Potaro-Siparuni
Sturnira tildae|ABGYE196-06|ROM 111642|Guyana.Potaro-Siparuni
Sturnira tildae|ABGYC355-06|ROM 107169|Guyana.Potaro-Siparuni
Sturnira tildae|ABGYD018-06|ROM F43288|Guyana.Cuyuni-Mazaruni
Sturnira tildae|ABGYD037-06|ROM 108259|Guyana.Cuyuni-Mazaruni
Sturnira tildae|ABGYD321-06|ROM 108661|Guyana.Potaro-Siparuni
Sturnira tildae|ABGYE473-06|ROM 111945|Guyana.Potaro-Siparuni
Sturnira tildae|ABGYB569-06|ROM 103305|Guyana.Upper Takutu-Upper Essequibo
Sturnira tildae|BCBNC111-06|ROM 115754|Guyana.Potaro-Siparuni
Sturnira tildae|ABSRA358-06|ROM 116947|Suriname.Nickerie
Sturnira tildae|BCBNC197-06|ROM 116673|Guyana.Potaro-Siparuni
Sturnira tildae|ABGYB568-06|ROM 103304|Guyana.Upper Takutu-Upper Essequibo
Sturnira tildae|ABGYE158-06|ROM 111601|Guyana.Potaro-Siparuni
Sturnira tildae|ABGYG195-06|ROM 115772|Guyana.Potaro-Siparuni
Sturnira tildae|BCBNT596-06|ROM 113438|Guyana.Upper Demerara-Berbice
Sturnira tildae|BCBNT320-06|ROM 108770|Guyana.Potaro-Siparuni
Sturnira tildae|ABGYF330-06|ROM 114725|Guyana.Potaro-Siparuni
Sturnira tildae|BCBNC120-06|ROM 115773|Guyana.Potaro-Siparuni
Sturnira tildae|ABGYB484-06|ROM 103219|Guyana.Upper Takutu-Upper Essequibo
Sturnira tildae|ABGYC246-06|ROM 107026|Guyana.Potaro-Siparuni
Sturnira tildae|ABGYC278-06|ROM 107069|Guyana.Potaro-Siparuni
Sturnira tildae|ABGYC304-06|ROM 107114|Guyana.Potaro-Siparuni
Sturnira tildae|ABGYC756-06|ROM F43184|Guyana.Cuyuni-Mazaruni
Sturnira tildae|ABGYD439-06|ROM 108790|Guyana.Potaro-Siparuni
Sturnira tildae|ABGYE071-06|ROM 111514|Guyana.Potaro-Siparuni
Sturnira tildae|ABGYE195-06|ROM 111641|Guyana.Potaro-Siparuni
Sturnira tildae|ABGYE414-06|ROM 111869|Guyana.Potaro-Siparuni
Sturnira tildae|ABGYF352-06|ROM 114762|Guyana.Potaro-Siparuni
Sturnira tildae|ABSRA435-06|ROM 117025|Suriname.Nickerie
Sturnira tildae|BCBN349-05|ROM 100981|Guyana.Barima-Waini
Sturnira tildae|BCBN372-05|ROM 101167|Guyana.Barima-Waini
Sturnira tildae|BCBNC017-06|ROM 114723|Guyana.Potaro-Siparuni
Sturnira tildae|BCBNC079-06|ROM 115262|Guyana.Cuyuni-Mazaruni
Sturnira tildae|BCBNT264-06|ROM 108240|Guyana.Cuyuni-Mazaruni
Sturnira tildae|BCBNT326-06|ROM 108847|Guyana.Potaro-Siparuni
Sturnira tildae|BCBNT992-06|ROM 114662|Guyana.Potaro-Siparuni
Sturnira tildae|ABGYD797-06|ROM 109219|Guyana.Potaro-Siparuni
Sturnira tildae|ABGYE828-06|ROM 113439|Guyana.Upper Demerara-Berbice
Sturnira tildae|BCBNC080-06|ROM 115263|Guyana.Cuyuni-Mazaruni
Sturnira tildae|BCBNC015-06|ROM 114721|Guyana.Potaro-Siparuni
Sturnira tildae|ABGYE332-06|ROM 111782|Guyana.Potaro-Siparuni
Sturnira tildae|ABRMM086-07|ROM F39716|Guyana.Potaro-Siparuni
Sturnira tildae|ABGYB588-06|ROM 103329|Guyana.Upper Takutu-Upper Essequibo
Sturnira tildae|ABGYB447-06|ROM 103162|Guyana.Upper Takutu-Upper Essequibo
Sturnira tildae|ABGYF336-06|ROM 114739|Guyana.Potaro-Siparuni
Sturnira tildae|BCBNC018-06|ROM 114724|Guyana.Potaro-Siparuni
Sturnira tildae|BCBNT236-06|ROM 108137|Guyana.Cuyuni-Mazaruni
Sturnira tildae|BCBNT713-06|ROM 113882|Suriname.Brokopondo
Sturnira tildae|ABGYC294-06|ROM 107099|Guyana.Potaro-Siparuni
Sturnira tildae|ABGYC668-06|ROM 107488|Guyana.Potaro-Siparuni
Sturnira tildae|ABGYA149-06|ROM 97994|Guyana.Potaro-Siparuni
Sturnira tildae|ABGYF349-06|ROM 114759|Guyana.Potaro-Siparuni
Sturnira tildae|BCBN895-05|ROM 105726|Ecuador.Napo
Sturnira tildae|ABGYD795-06|ROM 109217|Guyana.Potaro-Siparuni
Sturnira tildae|BCBN905-05|ROM 105765|Ecuador.Napo
Sturnira tildae|BCBNT594-06|ROM 113436|Guyana.Upper Demerara-Berbice
Sturnira tildae|ABGYD039-06|ROM 108261|Guyana.Cuyuni-Mazaruni
Sturnira tildae|ABGYD420-06|ROM 108769|Guyana.Potaro-Siparuni
Sturnira tildae|BCBNC132-06|ROM 115800|Guyana.Potaro-Siparuni
Sturnira tildae|ABGYC375-06|ROM 107192|Guyana.Potaro-Siparuni
Sturnira tildae|ABGYC415-06|ROM 107237|Guyana.Potaro-Siparuni
Sturnira tildae|ABGYF354-06|ROM 114764|Guyana.Potaro-Siparuni
Sturnira tildae|BCBNC001-06|ROM 114687|Guyana.Potaro-Siparuni

Sturnira tildae|ABGYC415-06|ROM 107237|Guyana.Potaro-Siparuni
Sturnira tildae|ABGYF354-06|ROM 114764|Guyana.Potaro-Siparuni
Sturnira tildae|BCBNC001-06|ROM 114687|Guyana.Potaro-Siparuni
Sturnira tildae|ABGYC517-06|ROM 107355|Guyana.Potaro-Siparuni
Sturnira tildae|ABGYC416-06|ROM 107238|Guyana.Potaro-Siparuni
Sturnira tildae|ABGYB896-06|ROM 104741|Guyana.Potaro-Siparuni
Sturnira tildae|ABGYB529-06|ROM 103264|Guyana.Upper Takutu-Upper Essequibo
Sturnira tildae|ABGYE541-06|ROM 112014|Guyana.Potaro-Siparuni
Sturnira tildae|ABGYF329-06|ROM 114720|Guyana.Potaro-Siparuni
Sturnira tildae|BCBNT327-06|ROM 108848|Guyana.Potaro-Siparuni
Sturnira tildae|BCBNT966-06|ROM 114878|Guyana.Potaro-Siparuni
Sturnira tildae|ABGYC809-06|ROM F43248|Guyana.Cuyuni-Mazaruni
Sturnira tildae|ABGYC354-06|ROM 107168|Guyana.Potaro-Siparuni
Sturnira tildae|ABGYC412-06|ROM 107234|Guyana.Potaro-Siparuni
Sturnira tildae|ABGYD322-06|ROM 108662|Guyana.Potaro-Siparuni
Sturnira tildae|ABGYD438-06|ROM 108789|Guyana.Potaro-Siparuni
Sturnira tildae|ABGYD477-06|ROM 108829|Guyana.Potaro-Siparuni
Sturnira tildae|ABGYD526-06|ROM 108884|Guyana.Potaro-Siparuni
Sturnira tildae|ABGYE084-06|ROM 111527|Guyana.Potaro-Siparuni
Sturnira tildae|ABGYE839-06|ROM 113452|Guyana.Upper Demerara-Berbice
Sturnira tildae|BCBNC196-06|ROM 116671|Guyana.Potaro-Siparuni
Sturnira tildae|BCBNT060-06|ROM 106728|Guyana.Upper Takutu-Upper Essequibo
Sturnira tildae|BCBNT244-06|ROM 108159|Guyana.Cuyuni-Mazaruni
Sturnira tildae|BCBNT622-06|ROM 113538|Guyana.Upper Takutu-Upper Essequibo
Sturnira tildae|BCBNT904-06|ROM 115546|Guyana.Essequibo Islands-West Demerara
Sturnira tildae|BCBNT998-06|ROM 114686|Guyana.Potaro-Siparuni
Trachops cirrhosus|ABGYC141-06|ROM 106654|Guyana.Upper Takutu-Upper Essequibo
Trachops cirrhosus|ABGYC091-06|ROM 106597|Guyana.Upper Takutu-Upper Essequibo
Trachops cirrhosus PS2|BCBNT028-06|ROM 106595|Guyana.Upper Takutu-Upper Essequibo
Trachops cirrhosus|ABGYG745-08|ROM 119173|Guyana
Trachops cirrhosus|ABGYG1080-08|ROM 119004|Guyana
Trachops cirrhosus|ABGYA194-06|ROM 98062|Guyana.Potaro-Siparuni
Trachops cirrhosus|ABGYD336-06|ROM 108676|Guyana.Potaro-Siparuni
Trachops cirrhosus|ABGYD535-06|ROM 108894|Guyana.Potaro-Siparuni
Trachops cirrhosus PS3|BCBNT292-06|ROM 108492|Guyana.Potaro-Siparuni
Trachops cirrhosus PS3|BCBNT302-06|ROM 108538|Guyana.Potaro-Siparuni
Trachops cirrhosus|ABGYG1078-08|ROM 119002|Guyana
Trachops cirrhosus PS3|BCBNT607-06|ROM 113499|Guyana.Upper Takutu-Upper Essequibo
Trachops cirrhosus PS3|BCBNT606-06|ROM 113498|Guyana.Upper Takutu-Upper Essequibo
Trachops cirrhosus PS3|BCBNT029-06|ROM 106596|Guyana.Upper Takutu-Upper Essequibo
Trachops cirrhosus|ABGYG827-08|ROM 119255|Guyana
Trachops cirrhosus|ABGYG602-08|ROM 119030|Guyana
Trachops cirrhosus|ABGYG1067-08|ROM 118991|Guyana
Trachops cirrhosus|ABGYG1051-08|ROM 118975|Guyana
Trachops cirrhosus|ABGYG1050-08|ROM 118974|Guyana
Trachops cirrhosus|ABGYG1049-08|ROM 118973|Guyana
Trachops cirrhosus|ABGYF032-06|ROM 113533|Guyana.Upper Takutu-Upper Essequibo
Trachops cirrhosus|ABGYF009-06|ROM 113497|Guyana.Upper Takutu-Upper Essequibo
Trachops cirrhosus|ABGYF004-06|ROM 113489|Guyana.Upper Takutu-Upper Essequibo
Trachops cirrhosus|ABGYF003-06|ROM 113488|Guyana.Upper Takutu-Upper Essequibo
Trachops cirrhosus|ABGYC154-06|ROM 106675|Guyana.Upper Takutu-Upper Essequibo
Trachops cirrhosus|ABGYC153-06|ROM 106674|Guyana.Upper Takutu-Upper Essequibo
Trachops cirrhosus|ABGYC167-06|ROM 106694|Guyana.Upper Takutu-Upper Essequibo
Trachops cirrhosus PS3|BCBNT050-06|ROM 106695|Guyana.Upper Takutu-Upper Essequibo
Trachops cirrhosus PS3|BCBNT052-06|ROM 106700|Guyana.Upper Takutu-Upper Essequibo
Trachops cirrhosus|ABGYG659-08|ROM 119087|Guyana
Trachops cirrhosus|ABGYG1079-08|ROM 119003|Guyana
Trachops cirrhosus PS3|BCBNT611-06|ROM 113514|Guyana.Upper Takutu-Upper Essequibo
Trachops cirrhosus PS3|BCBNT628-06|ROM 113562|Guyana.Upper Takutu-Upper Essequibo
Trachops cirrhosus|ABECA927-06|ROM 106110|Ecuador.Napo
Trachops cirrhosus|ABECA642-06|ROM 105697|Ecuador.Napo
Trachops cirrhosus|ABECA378-06|ROM 105274|Ecuador.Napo
Trachops cirrhosus|ABECA107-06|ROM 104399|Ecuador.Napo
Trachops cirrhosus|ABECB103-08|ROM 118765|Ecuador
Trachops cirrhosus|ABECA791-06|ROM 105955|Ecuador.Napo
Trachops cirrhosus|ABECA110-06|ROM 104402|Ecuador.Napo
Trachops cirrhosus|ABECA109-06|ROM 104401|Ecuador.Napo
Trachops cirrhosus|ABECA108-06|ROM 104400|Ecuador.Napo
Trachops cirrhosus|ABECA024-06|ROM F37097|Ecuador.Napo
Trachops cirrhosus|BCBN568-05|ROM 104008|Ecuador.Napo
Trachops cirrhosus|BCBN732-05|ROM 104418|Ecuador.Napo
Trachops cirrhosus|BCBN814-05|ROM 105273|Ecuador.Napo
Trachops cirrhosus|ABECA873-06|ROM 106050|Ecuador.Napo
Trachops cirrhosus|ABECA402-06|ROM 105298|Ecuador.Napo
Trachops cirrhosus|ABECA604-06|ROM 105639|Ecuador.Napo
Trachops cirrhosus|ABECA861-06|ROM 106037|Ecuador.Napo
Trachops cirrhosus|ABECA130-06|ROM 104427|Ecuador.Napo
Trachops cirrhosus|ABECA310-06|ROM 105187|Ecuador.Napo
Trachops cirrhosus|ABECB001-06|ROM 106321|Ecuador.Napo
Trachops cirrhosus|BCBN600-05|ROM 104082|Ecuador.Napo
Trachops cirrhosus|ABECA435-06|ROM F37663|Ecuador.Napo
Trachops cirrhosus|ABECA438-06|ROM 105338|Ecuador.Napo
Trachops cirrhosus|BCBN951-05|ROM 105915|Ecuador.Napo
Trachops cirrhosus|BCBNT169-06|ROM 107843|Venezuela.Amazonas
Trachops cirrhosus|BCBNT202-06|ROM 107908|Venezuela.Bolivar
Trachops cirrhosus|BCBNT216-06|ROM 107926|Venezuela.Bolivar
Trachops cirrhosus|BCBNT220-06|ROM 107935|Venezuela.Bolivar
Trachops cirrhosus|BCBN012-05|ROM 95747|Mexico.Campeche
Trachops cirrhosus|BCBN227-05|ROM 99493|Guatemala.Peten
Trachops cirrhosus|BCBN233-05|ROM 99527|Guatemala.Peten
Trachops cirrhosus|BCBN615-05|ROM 104214|Panama.Canal Zone
Trachops cirrhosus|ABSA044-06|ROM 107945|Venezuela.Guarico
Trachops cirrhosus|BCBNT230-06|ROM 107957|Venezuela.Guarico
Trachops cirrhosus|ABSRA466-06|ROM 117056|Suriname.Nickerie
Trachops cirrhosus|BCBNT720-06|ROM 113890|Suriname.Brokopondo
Trachops cirrhosus|ABSMS487-06|ROM 117649|Suriname.Sipaliwini
Trachops cirrhosus|ABSRA275-06|ROM 116864|Suriname.Nickerie
Trachops cirrhosus|ABSRA311-06|ROM 116900|Suriname.Nickerie
Trachops cirrhosus|BCBNT719-06|ROM 113888|Suriname.Brokopondo
Trachops cirrhosus|BCBNT748-06|ROM 113931|Suriname.Brokopondo
Trachops cirrhosus|BCBNT776-06|ROM 114002|Suriname.Brokopondo
Trachops cirrhosus|ABSMS039-06|ROM 117201|Suriname.Sipaliwini
Trachops cirrhosus|ABSMS361-06|ROM 117523|Suriname.Sipaliwini

[illegible]
Trachops cirrhosus|BCBNT776-06|ROM 114002|Suriname.Brokopondo
Trachops cirrhosus|ABSMS039-06|ROM 117201|Suriname.Sipaliwini
Trachops cirrhosus|ABSMS361-06|ROM 117523|Suriname.Sipaliwini
Trachops cirrhosus|BCBNT792-06|ROM 114052|Suriname.Brokopondo
Trachops cirrhosus PS1|BCBNC004-06|ROM 114690|Guyana.Potaro-Siparuni
Trachops cirrhosus PS1|BCBNC005-06|ROM 114691|Guyana.Potaro-Siparuni
Trachops cirrhosus PS1|BCBNC028-06|ROM 114752|Guyana.Potaro-Siparuni
Trachops cirrhosus PS1|BCBNC029-06|ROM 114753|Guyana.Potaro-Siparuni
Trachops cirrhosus PS1|BCBNC044-06|ROM 114790|Guyana.Potaro-Siparuni
Trachops cirrhosus PS1|BCBNC045-06|ROM 114791|Guyana.Potaro-Siparuni
Trachops cirrhosus PS1|BCBNC047-06|ROM 115065|Guyana.Cuyuni-Mazaruni
Trachops cirrhosus PS1|BCBNC051-06|ROM 115071|Guyana.Cuyuni-Mazaruni
Trachops cirrhosus PS1|BCBNC052-06|ROM 115072|Guyana.Cuyuni-Mazaruni
Trachops cirrhosus PS1|BCBNC053-06|ROM 115073|Guyana.Cuyuni-Mazaruni
Trachops cirrhosus PS1|BCBNC152-06|ROM 115914|Guyana.Potaro-Siparuni
Trachops cirrhosus PS1|BCBNT235-06|ROM 108133|Guyana.Cuyuni-Mazaruni
Trachops cirrhosus|ABGYA144-06|ROM 97988|Guyana.Potaro-Siparuni
Trachops cirrhosus|ABGYA157-06|ROM 98008|Guyana.Potaro-Siparuni
Trachops cirrhosus|ABGYA195-06|ROM 98063|Guyana.Potaro-Siparuni
Trachops cirrhosus|ABGYB838-06|ROM 104679|Guyana.Potaro-Siparuni
Trachops cirrhosus|ABGYB877-06|ROM 104721|Guyana.Potaro-Siparuni
Trachops cirrhosus|ABGYE285-06|ROM 111734|Guyana.Potaro-Siparuni
Trachops cirrhosus|ABGYE350-06|ROM 111800|Guyana.Potaro-Siparuni
Trachops cirrhosus|ABGYE385-06|ROM 111837|Guyana.Potaro-Siparuni
Trachops cirrhosus PS1|BCBNT278-06|ROM 108448|Guyana.Potaro-Siparuni
Trachops cirrhosus|ABGYB876-06|ROM 104720|Guyana.Potaro-Siparuni
Trachops cirrhosus|ABGYB890-06|ROM 104735|Guyana.Potaro-Siparuni
Trachops cirrhosus|ABGYE398-06|ROM 111850|Guyana.Potaro-Siparuni
Trachops cirrhosus|ABGYE411-06|ROM 111864|Guyana.Potaro-Siparuni
Trachops cirrhosus PS1|BCBN138-05|ROM 98020|Guyana.Potaro-Siparuni
Trachops cirrhosus PS1|BCBNT298-06|ROM 108510|Guyana.Potaro-Siparuni
Trachops cirrhosus|ABGYG807-08|ROM 119235|Guyana
Trachops cirrhosus|ABGYG825-08|ROM 119253|Guyana
Trachops cirrhosus|ABGYG826-08|ROM 119254|Guyana
Trachops cirrhosus|ABGYG855-08|ROM 119283|Guyana
Trachops cirrhosus|ABGYG880-08|ROM 119308|Guyana
Trachops cirrhosus|ABGYG881-08|ROM 119309|Guyana
Trachops cirrhosus|ABGYG882-08|ROM 119310|Guyana
Trachops cirrhosus|ABGYG883-08|ROM 119311|Guyana
Trachops cirrhosus|ABGYG884-08|ROM 119312|Guyana
Trachops cirrhosus|ABGYG908-08|ROM 119336|Guyana
Trachops cirrhosus|ABGYG942-08|ROM 119370|Guyana
Trachops cirrhosus|ABGYG943-08|ROM 119371|Guyana
Trachops cirrhosus|ABGYC457-06|ROM 107283|Guyana.Potaro-Siparuni
Trachops cirrhosus PS1|BCBN548-05|ROM 103423|Guyana.Upper Demerara-Berbice
Trachops cirrhosus PS1|BCBN553-05|ROM 103495|Guyana.Upper Demerara-Berbice
Trachops cirrhosus PS1|BCBNC117-06|ROM 115766|Guyana.Potaro-Siparuni
Trachops cirrhosus|ABGYD558-06|ROM 108928|Guyana.Potaro-Siparuni
Trachops cirrhosus|ABGYD559-06|ROM 108929|Guyana.Potaro-Siparuni
Trachops cirrhosus PS1|BCBN326-05|ROM 100859|Guyana.Barima-Waini
Trachops cirrhosus PS1|BCBN329-05|ROM 100894|Guyana.Barima-Waini
Trachops cirrhosus PS1|BCBN351-05|ROM 100983|Guyana.Barima-Waini
Trachops cirrhosus PS1|BCBN353-05|ROM 101029|Guyana.Barima-Waini
Trachops cirrhosus PS1|BCBN539-05|ROM 103359|Guyana.Upper Demerara-Berbice
Trachops cirrhosus PS1|BCBN552-05|ROM 103474|Guyana.Upper Demerara-Berbice
Trachops cirrhosus PS1|BCBNT875-06|ROM 115494|Guyana.Essequibo Islands-West Demerara
Trachops cirrhosus PS1|BCBNT876-06|ROM 115495|Guyana.Essequibo Islands-West Demerara
Trachops cirrhosus PS1|BCBNT900-06|ROM 115535|Guyana.Essequibo Islands-West Demerara
Trachops cirrhosus PS1|BCBNT938-06|ROM 115638|Guyana.Essequibo Islands-West Demerara
Trachops cirrhosus PS1|BCBN295-05|ROM 100260|Guyana.East Berbice-Corentyne
Trachops cirrhosus PS1|BCBN320-05|ROM 100833|Guyana.Barima-Waini
Trachops cirrhosus|ABRMM072-07|ROM F39297|Guyana.Potaro-Siparuni
Trachops cirrhosus PS1|BCBN132-05|ROM 97927|Guyana.Upper Takutu-Upper Essequibo
Trachops cirrhosus|ABGYE859-06|ROM 113475|Guyana.Upper Takutu-Upper Essequibo
Trachops cirrhosus|ABGYG604-08|ROM 119032|Guyana
Trachops cirrhosus|ABGYE472-06|ROM 111944|Guyana.Potaro-Siparuni
Trachops cirrhosus|ABGYE543-06|ROM 112016|Guyana.Potaro-Siparuni
Trachops cirrhosus|BCBNC204-06|ROM 116710|Guyana.Potaro-Siparuni
Trachops cirrhosus|BCBNC210-06|ROM 116726|Guyana.Potaro-Siparuni
Trachops cirrhosus PS1|BCBNC118-06|ROM 115767|Guyana.Potaro-Siparuni
Trachops cirrhosus PS1|BCBNC191-06|ROM 116599|Guyana.Potaro-Siparuni
Trachops cirrhosus|ABGYE015-06|ROM 109299|Guyana.Potaro-Siparuni
Trachops cirrhosus|ABGYD750-06|ROM 109168|Guyana.Potaro-Siparuni
Trachops cirrhosus|ABGYC456-06|ROM 107282|Guyana.Potaro-Siparuni
Trachops cirrhosus|ABGYC268-06|ROM 107057|Guyana.Potaro-Siparuni
Trachops cirrhosus|ABGYC046-06|ROM 104823|Guyana.Potaro-Siparuni
Trachops cirrhosus|ABGYC022-06|ROM 104799|Guyana.Potaro-Siparuni
Trachops cirrhosus|ABGYB477-06|ROM 103212|Guyana.Upper Takutu-Upper Essequibo
Trachops cirrhosus|ABGYB030-06|ROM 101002|Guyana.Barima-Waini
Trachops cirrhosus|ABGYA851-06|ROM 100919|Guyana.Barima-Waini
Trachops cirrhosus|ABGYA305-06|ROM 98701|Guyana.Barima-Waini
Trachops cirrhosus|ABGYG660-08|ROM 119088|Guyana
Trachops cirrhosus PS1|BCBNT599-06|ROM 113457|Guyana.Upper Takutu-Upper Essequibo
Trachops cirrhosus PS1|BCBNT939-06|ROM 115639|Guyana.Essequibo Islands-West Demerara
Glossophaga leachii|ABCSA148-06|ROM FN31382|Guatemala.El Progreso
Glossophaga leachii|BCBN247-05|ROM 99666|Guatemala.El Progreso
Glossophaga leachii|BCBN248-05|ROM 99667|Guatemala.El Progreso
Glossophaga leachii|BCBN065-05|ROM 98392|Guatemala.El Progreso
Glossophaga leachii|BCBN066-05|ROM 98394|Guatemala.El Progreso
Glossophaga leachii|BCBN067-05|ROM 98395|Guatemala.El Progreso
Glossophaga leachii|BCBN246-05|ROM 99665|Guatemala.El Progreso
Glossophaga leachii|BCBN249-05|ROM 99668|Guatemala.El Progreso
Glossophaga leachii|BCBN420-05|ROM 101335|El Salvador.Ahuachapan
Glossophaga longirostris|ABCSA910-06|ROM 107817|Venezuela
Glossophaga longirostris|ABSA001-06|ROM 107818|Venezuela
Glossophaga longirostris|ABSA007-06|ROM 107851|Venezuela
Glossophaga longirostris|ABSA022-06|ROM 107876|Venezuela
Glossophaga longirostris|ABSA023-06|ROM 107877|Venezuela
Glossophaga longirostris|ABSA024-06|ROM 107878|Venezuela
Glossophaga longirostris|ABSA026-06|ROM 107881|Venezuela
Glossophaga longirostris|ABSA025-06|ROM 107880|Venezuela
Glossophaga longirostris|ABSA033-06|ROM 107903|Venezuela
[illegible]

Glossophaga longirostris|ABSA026-06|ROM 107881|Venezuela
Glossophaga longirostris|ABSA025-06|ROM 107880|Venezuela
Glossophaga longirostris|ABSA033-06|ROM 107903|Venezuela
Glossophaga longirostris|BCBNT156-06|ROM 107819|Venezuela.Amazonas
Glossophaga longirostris|BCBNT182-06|ROM 107879|Venezuela.Amazonas
Glossophaga longirostris|ABGYG625-08|ROM 119053|Guyana
Glossophaga longirostris|ABGYG938-08|ROM 119366|Guyana
Glossophaga longirostris|ABGYG986-08|ROM 119414|Guyana
Glossophaga longirostris|ABGYG963-08|ROM 119391|Guyana
Glossophaga longirostris|ABGYG707-08|ROM 119135|Guyana
Glossophaga longirostris|ABGYG685-08|ROM 119113|Guyana
Glossophaga longirostris|ABGYG684-08|ROM 119112|Guyana
Glossophaga longirostris|ABGYG668-08|ROM 119096|Guyana
Glossophaga longirostris|ABGYG616-08|ROM 119044|Guyana
Glossophaga longirostris|ABGYG615-08|ROM 119043|Guyana
Glossophaga longirostris|ABGYG585-08|ROM 119013|Guyana
Glossophaga longirostris|ABGYA010-06|ROM 97778|Guyana.Upper Takutu-Upper Essequibo
Glossophaga longirostris|ABGYG888-08|ROM 119316|Guyana
Glossophaga longirostris|ABGYA013-06|ROM 97784|Guyana.Upper Takutu-Upper Essequibo
Glossophaga longirostris|ABGYA008-06|ROM 97776|Guyana.Upper Takutu-Upper Essequibo
Glossophaga longirostris|ABGYA009-06|ROM 97777|Guyana.Upper Takutu-Upper Essequibo
Glossophaga longirostris|ABGYA011-06|ROM 97779|Guyana.Upper Takutu-Upper Essequibo
Glossophaga longirostris|ABGYA012-06|ROM 97780|Guyana.Upper Takutu-Upper Essequibo
Glossophaga longirostris|BCBN097-05|ROM 97781|Guyana.Upper Takutu-Upper Essequibo
Glossophaga longirostris|BCBN100-05|ROM 97785|Guyana.Upper Takutu-Upper Essequibo
Glossophaga longirostris|ABSA045-06|ROM 107946|Venezuela.Guarico
Glossophaga longirostris|BCBNT226-06|ROM 107947|Venezuela.Guarico
Glossophaga longirostris|ABSA048-06|ROM 107952|Venezuela.Guarico
Glossophaga longirostris|BCBNT217-06|ROM 107927|Venezuela.Bolivar
Glossophaga longirostris|ABSA046-06|ROM 107949|Venezuela.Guarico
Glossophaga longirostris|ABSA047-06|ROM 107951|Venezuela.Guarico
Glossophaga longirostris|BCBNT228-06|ROM 107950|Venezuela.Guarico
Glossophaga commissarisi|ABCSA722-06|ROM 101292|El Salvador.Ahuachapan
Glossophaga commissarisi|BCBN377-05|ROM 101230|El Salvador.Ahuachapan
Glossophaga commissarisi|BCBN401-05|ROM 101289|El Salvador.Ahuachapan
Glossophaga commissarisi|BCBN512-05|ROM 97626|Mexico.Chiapas
Glossophaga commissarisi|ABCSA744-06|ROM 101332|El Salvador.Ahuachapan
Glossophaga commissarisi|ABCSA746-06|ROM 101334|El Salvador.Ahuachapan
Glossophaga commissarisi|ABCSA686-06|ROM 101231|El Salvador.Ahuachapan
Glossophaga commissarisi|ABCSA701-06|ROM 101259|El Salvador.Ahuachapan
Glossophaga commissarisi|ABCSA702-06|ROM 101260|El Salvador.Ahuachapan
Glossophaga commissarisi|BCBN390-05|ROM 101261|El Salvador.Ahuachapan
Glossophaga commissarisi|ABCSA700-06|ROM 101258|El Salvador.Ahuachapan
Glossophaga commissarisi|ABCSA720-06|ROM 101290|El Salvador.Ahuachapan
Glossophaga commissarisi|ABCSA721-06|ROM 101291|El Salvador.Ahuachapan
Glossophaga commissarisi|BCBN513-05|ROM 97627|Mexico.Chiapas
Glossophaga commissarisi|BCBN514-05|ROM 97628|Mexico.Chiapas
Glossophaga commissarisi|BCBN520-05|ROM 97634|Mexico.Chiapas
Glossophaga commissarisi|BCBNT402-06|ROM 108282|Costa Rica.Limon
Glossophaga commissarisi|ABSCA075-06|ROM F38126|Panama
Glossophaga commissarisi|BCBN626-05|ROM 104231|Panama
Glossophaga commissarisi|BCBN645-05|ROM 104259|Panama
Glossophaga commissarisi|ABCSA375-06|ROM 99388|Guatemala.Peten
Glossophaga commissarisi|BCBN205-05|ROM 99424|Guatemala.Peten
Glossophaga commissarisi|BCBN220-05|ROM 99471|Guatemala.Peten
Glossophaga commissarisi|ABCSA374-06|ROM 99387|Guatemala.Peten
Glossophaga commissarisi|BCBN206-05|ROM 99425|Guatemala.Peten
Glossophaga commissarisi|BCBNT420-06|ROM 108300|Costa Rica.Limon
Glossophaga commissarisi|BCBNT448-06|ROM 108326|Costa Rica.Limon
Glossophaga commissarisi|BCBN201-05|ROM 99386|Guatemala.Peten
Glossophaga commissarisi|BCBN203-05|ROM 99422|Guatemala.Peten
Glossophaga commissarisi|BCBN204-05|ROM 99423|Guatemala.Peten
Glossophaga commissarisi|BCBN400-05|ROM 101288|El Salvador.Ahuachapan
Glossophaga commissarisi|BCBN419-05|ROM 101331|El Salvador.Ahuachapan
Glossophaga commissarisi|BCBN691-05|ROM 104331|Panama.Chiriqui
Glossophaga commissarisi|BCBNT419-06|ROM 108299|Costa Rica.Limon
Glossophaga commissarisi|BCBNT436-06|ROM 108314|Costa Rica.Limon
Glossophaga commissarisi|BCBNT449-06|ROM 108327|Costa Rica.Limon
Glossophaga soricina|ABCSA461-06|ROM 99612|Guatemala.Peten
Glossophaga soricina|ABMXC209-06|ROM 96208|Mexico.Campeche
Glossophaga soricina|BCBN213-05|ROM 99580|Guatemala.Peten
Glossophaga soricina|BCBN023-05|ROM 96202|Mexico.Campeche
Glossophaga soricina|BCBN022-05|ROM 96201|Mexico.Campeche
Glossophaga soricina|ABMXC205-06|ROM FN30019|Mexico.Campeche
Glossophaga soricina|ABCSA257-06|ROM 99216|Guatemala.Peten
Glossophaga soricina|ABCSA489-06|ROM 99473|Guatemala.Peten
Glossophaga soricina|ABMXC208-06|ROM 96207|Mexico.Campeche
Glossophaga soricina|ABMXC442-06|ROM 97386|Mexico.Quintana Roo
Glossophaga soricina|BCBN024-05|ROM 96203|Mexico.Campeche
Glossophaga soricina|ABCSA253-06|ROM 99212|Guatemala.Peten
Glossophaga soricina|BCBN021-05|ROM 96200|Mexico.Campeche
Glossophaga soricina|ABMXC211-06|ROM 96210|Mexico.Campeche
Glossophaga soricina|ABMXC207-06|ROM 96206|Mexico.Campeche
Glossophaga soricina|ABMXC524-06|ROM 97447|Mexico.Quintana Roo
Glossophaga soricina|ABCSA312-06|ROM 99290|Guatemala.Peten
Glossophaga soricina|BCBN090-05|ROM 98480|Guatemala.El Progreso
Glossophaga soricina|ABCSA481-06|ROM 99464|Guatemala.Peten
Glossophaga soricina|ABMXC206-06|ROM 96205|Mexico.Campeche
Glossophaga soricina|BCBN182-05|ROM 99238|Guatemala.Peten
Glossophaga soricina|BCBN221-05|ROM 99474|Guatemala.Peten
Glossophaga soricina|ABMXC650-06|ROM 97681|Mexico.Chiapas
Glossophaga soricina|BCBN207-05|ROM 99426|Guatemala.Peten
Glossophaga soricina|ABCSA270-06|ROM 99237|Guatemala.Peten
Glossophaga soricina|ABMXC523-06|ROM 97446|Mexico.Quintana Roo
Glossophaga soricina|BCBN214-05|ROM 99581|Guatemala.Peten
Glossophaga soricina|ABCSA255-06|ROM 99214|Guatemala.Peten
Glossophaga soricina|ABCSA254-06|ROM 99213|Guatemala.Peten
Glossophaga soricina|ABCSA210-06|ROM FN31468|Guatemala.El Progreso
Glossophaga soricina|ABCSA256-06|ROM 99215|Guatemala.Peten
Glossophaga soricina|ABCSA258-06|ROM 99217|Guatemala.Peten
Glossophaga soricina|ABCSA259-06|ROM 99218|Guatemala.Peten
Glossophaga soricina|ABCSA439-06|ROM 99582|Guatemala.Peten
Glossophaga soricina|ABCSA487-06|ROM 99470|Guatemala.Peten
[illegible]

Glossophaga soricina|ABCSA259-06|ROM 99218|Guatemala.Peten
Glossophaga soricina|ABCSA439-06|ROM 99582|Guatemala.Peten
Glossophaga soricina|ABCSA487-06|ROM 99470|Guatemala.Peten
Glossophaga soricina|ABCSA488-06|ROM 99472|Guatemala.Peten
Glossophaga soricina|ABMXC210-06|ROM 96209|Mexico.Campeche
Glossophaga soricina|ABMXC499-06|ROM 97415|Mexico.Quintana Roo
Glossophaga soricina|BCBN217-05|ROM 99613|Guatemala.Peten
Glossophaga soricina|BCBN181-05|ROM 99236|Guatemala.Peten
Glossophaga soricina|BCBN489-05|ROM 97448|Mexico.Quintana Roo
Glossophaga soricina|BCBN621-05|ROM 104221|Panama.Canal Zone
Glossophaga soricina|ABSCA044-06|ROM F38055|Panama.Canal Zone
Glossophaga soricina|BCBNT546-06|ROM 112170|Nicaragua.Rivas
Glossophaga soricina|BCBNT547-06|ROM 112171|Nicaragua.Rivas
Glossophaga soricina|ABGYF207-06|ROM 113777|Guyana.Demerara-Mahaica
Glossophaga soricina|ABGYF247-06|ROM 113829|Guyana.Demerara-Mahaica
Glossophaga soricina|ABGYD381-06|ROM 108726|Guyana.Potaro-Siparuni
Glossophaga soricina|ABGYG690-08|ROM 119118|Guyana
Glossophaga soricina|ABGYE610-06|ROM 112089|Guyana.Potaro-Siparuni
Glossophaga soricina|ABGYA046-06|ROM 97839|Guyana.Upper Takutu-Upper Essequibo
Glossophaga soricina|ABGYD341-06|ROM 108682|Guyana.Potaro-Siparuni
Glossophaga soricina|ABGYE244-06|ROM 111692|Guyana.Potaro-Siparuni
Glossophaga soricina|ABGYB697-06|ROM 103461|Guyana.Upper Demerara-Berbice
Glossophaga soricina|ABGYB756-06|ROM 103524|Guyana.Upper Demerara-Berbice
Glossophaga soricina|ABGYB760-06|ROM 103528|Guyana.Upper Demerara-Berbice
Glossophaga soricina|ABGYG630-08|ROM 119058|Guyana
Glossophaga soricina|ABGYA060-06|ROM 97867|Guyana.Upper Takutu-Upper Essequibo
Glossophaga soricina|ABGYA551-06|ROM 100224|Guyana.East Berbice-Corentyne
Glossophaga soricina|ABGYB128-06|ROM 101109|Guyana.Barima-Waini
Glossophaga soricina|ABGYB741-06|ROM 103508|Guyana.Upper Demerara-Berbice
Glossophaga soricina|ABGYC570-06|ROM 107416|Guyana.Potaro-Siparuni
Glossophaga soricina|ABGYG613-08|ROM 119041|Guyana
Glossophaga soricina|ABGYG960-08|ROM 119388|Guyana
Glossophaga soricina|BCBNT203-06|ROM 107909|Venezuela.Bolivar
Glossophaga soricina|ABGYF160-06|ROM 113714|Guyana.Demerara-Mahaica
Glossophaga soricina|ABGYF190-06|ROM 113752|Guyana.Demerara-Mahaica
Glossophaga soricina|ABGYF245-06|ROM 113827|Guyana.Demerara-Mahaica
Glossophaga soricina|BCBNT175-06|ROM 107852|Venezuela.Amazonas
Glossophaga soricina|ABGYG627-08|ROM 119055|Guyana
Glossophaga soricina|BCBNT227-06|ROM 107948|Venezuela.Guarico
Glossophaga soricina|ABGYF149-06|ROM 113702|Guyana.Demerara-Mahaica
Glossophaga soricina|ABGYG476-06|ROM 116615|Guyana.Potaro-Siparuni
Glossophaga soricina|ABGYG481-06|ROM 116621|Guyana.Potaro-Siparuni
Glossophaga soricina|ABGYA032-06|ROM 97822|Guyana.Upper Takutu-Upper Essequibo
Glossophaga soricina|ABGYG671-08|ROM 119099|Guyana
Glossophaga soricina|ABGYG672-08|ROM 119100|Guyana
Glossophaga soricina|ABGYG480-06|ROM 116620|Guyana.Potaro-Siparuni
Glossophaga soricina|ABGYD323-06|ROM 108663|Guyana.Potaro-Siparuni
Glossophaga soricina|ABGYA047-06|ROM 97840|Guyana.Upper Takutu-Upper Essequibo
Glossophaga soricina|ABGYG983-08|ROM 119411|Guyana
Glossophaga soricina|ABSMS501-06|ROM 117663|Suriname.Sipaliwini
Glossophaga soricina|ABSRA496-06|ROM 117086|Suriname.Nickerie
Glossophaga soricina|ABSMS410-06|ROM 117572|Suriname.Sipaliwini
Glossophaga soricina|BCBNT233-06|ROM 108120|Guyana.Cuyuni-Mazaruni
Glossophaga soricina|BCBN754-05|ROM 104483|Ecuador.Napo
Glossophaga soricina|BCBN981-05|ROM 106088|Ecuador.Napo
Glossophaga soricina|ABGYA302-06|ROM 98698|Guyana.Barima-Waini
Glossophaga soricina|ABGYB693-06|ROM 103457|Guyana.Upper Demerara-Berbice
Glossophaga soricina|ABGYA761-06|ROM 100810|Guyana.Barima-Waini
Glossophaga soricina|ABGYC302-06|ROM 107107|Guyana.Potaro-Siparuni
Glossophaga soricina|ABGYB205-06|ROM 102907|Guyana.Upper Takutu-Upper Essequibo
Glossophaga soricina|ABGYG935-08|ROM 119363|Guyana
Glossophaga soricina|ABGYG811-08|ROM 119239|Guyana
Glossophaga soricina|BCBNT009-06|ROM 106365|Ecuador.Napo
Glossophaga soricina|ABECB090-08|ROM 118752|Ecuador
Glossophaga soricina|ABGYC571-06|ROM 107417|Guyana.Potaro-Siparuni
Glossophaga soricina|ABGYG885-08|ROM 119313|Guyana
Glossophaga soricina|ABGYC309-06|ROM 107120|Guyana.Potaro-Siparuni
Glossophaga soricina|BCBNT268-06|ROM 108275|Guyana.Cuyuni-Mazaruni
Glossophaga soricina|ABGYC509-06|ROM 107345|Guyana.Potaro-Siparuni
Glossophaga soricina|BCBN581-05|ROM 104038|Ecuador.Napo
Glossophaga soricina|ABGYA850-06|ROM 100916|Guyana.Barima-Waini
Glossophaga soricina|ABGYE699-06|ROM 112632|Guyana.Demerara-Mahaica
Glossophaga soricina|ABGYF198-06|ROM 113768|Guyana.Demerara-Mahaica
Glossophaga soricina|ABGYC510-06|ROM 107346|Guyana.Potaro-Siparuni
Glossophaga soricina|BCBNT252-06|ROM 108193|Guyana.Cuyuni-Mazaruni
Glossophaga soricina|ABGYF241-06|ROM 113821|Guyana.Demerara-Mahaica
Glossophaga soricina|ABGYG765-08|ROM 119193|Guyana
Glossophaga soricina|ABGYB523-06|ROM 103258|Guyana.Upper Takutu-Upper Essequibo
Glossophaga soricina|ABGYE112-06|ROM 111555|Guyana.Potaro-Siparuni
Glossophaga soricina|ABGYE123-06|ROM 111566|Guyana.Potaro-Siparuni
Glossophaga soricina|ABGYG628-08|ROM 119056|Guyana
Glossophaga soricina|ABGYA241-06|ROM 98130|Guyana.Upper Demerara-Berbice
Glossophaga soricina|ABGYE774-06|ROM 113375|Guyana.Upper Demerara-Berbice
Glossophaga soricina|BCBNT717-06|ROM 113886|Suriname.Brokopondo
Glossophaga soricina|ABSMS034-06|ROM 117196|Suriname.Sipaliwini
Glossophaga soricina|BCBNT718-06|ROM 113887|Suriname.Brokopondo
Glossophaga soricina|BCBNT727-06|ROM 113898|Suriname.Brokopondo
Glossophaga soricina|ABGYG556-06|ROM 116703|Guyana.Potaro-Siparuni
Glossophaga soricina|ABGYC508-06|ROM 107344|Guyana.Potaro-Siparuni
Glossophaga soricina|ABGYG886-08|ROM 119314|Guyana
Glossophaga soricina|ABGYG887-08|ROM 119315|Guyana
Glossophaga soricina|ABGYG937-08|ROM 119365|Guyana
Glossophaga soricina|ABGYG961-08|ROM 119389|Guyana
Glossophaga soricina|ABGYC645-06|ROM 107463|Guyana.Potaro-Siparuni
Glossophaga soricina|ABGYG936-08|ROM 119364|Guyana
Glossophaga soricina|ABSMS502-06|ROM 117664|Suriname.Sipaliwini
Glossophaga soricina|ABGYB696-06|ROM 103460|Guyana.Upper Demerara-Berbice
Glossophaga soricina|ABGYA086-06|ROM 97918|Guyana.Upper Takutu-Upper Essequibo
Glossophaga soricina|ABGYG614-08|ROM 119042|Guyana
Glossophaga soricina|ABGYG497-06|ROM 116639|Guyana.Potaro-Siparuni
Glossophaga soricina|ABGYG465-06|ROM 116603|Guyana.Potaro-Siparuni
Glossophaga soricina|ABGYE832-06|ROM 113444|Guyana.Upper Demerara-Berbice
Glossophaga soricina|ABGYC572-06|ROM 107418|Guyana.Potaro-Siparuni

Glossophaga soricina|ABGYG465-06|ROM 116603|Guyana.Potaro-Siparuni
Glossophaga soricina|ABGYE832-06|ROM 113444|Guyana.Upper Demerara-Berbice
Glossophaga soricina|ABGYC572-06|ROM 107418|Guyana.Potaro-Siparuni
Glossophaga soricina|ABGYB755-06|ROM 103523|Guyana.Upper Demerara-Berbice
Glossophaga soricina|ABGYG670-08|ROM 119098|Guyana
Glossophaga soricina|ABGYG482-06|ROM 116622|Guyana.Potaro-Siparuni
Glossophaga soricina|ABGYD063-06|ROM 108359|Guyana.Potaro-Siparuni
Glossophaga soricina|ABGYA357-06|ROM 98758|Guyana.Barima-Waini
Glossophaga soricina|ABGYA240-06|ROM 98129|Guyana.Upper Demerara-Berbice
Glossophaga soricina|ABSRA436-06|ROM 117026|Suriname.Nickerie
Glossophaga soricina|ABGYG866-08|ROM 119294|Guyana
Glossophaga soricina|ABGYD549-06|ROM 108914|Guyana.Potaro-Siparuni
Glossophaga soricina|ABGYF145-06|ROM 113696|Guyana.Demerara-Mahaica
Glossophaga soricina|ABGYF246-06|ROM 113828|Guyana.Demerara-Mahaica
Glossophaga soricina|ABGYG689-08|ROM 119117|Guyana
Glossophaga soricina|BCBNT509-06|ROM 111168|Brazil.Sao Paulo
Glossophaga soricina|ABGYC130-06|ROM 106642|Guyana.Upper Takutu-Upper Essequibo
Glossophaga soricina|ABGYA516-06|ROM 100183|Guyana.Demerara-Mahaica
Glossophaga soricina|ABGYE747-06|ROM 112690|Guyana.Demerara-Mahaica
Glossophaga soricina|ABGYG545-06|ROM 116691|Guyana.Potaro-Siparuni
Glossophaga soricina|ABGYA517-06|ROM 100184|Guyana.Demerara-Mahaica
Glossophaga soricina|ABGYD433-06|ROM 108784|Guyana.Potaro-Siparuni
Glossophaga soricina|ABGYG962-08|ROM 119390|Guyana
Glossophaga soricina|ABSMS462-06|ROM 117624|Suriname.Sipaliwini
Glossophaga soricina|ABGYA511-06|ROM 100178|Guyana.Demerara-Mahaica
Glossophaga soricina|ABGYB695-06|ROM 103459|Guyana.Upper Demerara-Berbice
Glossophaga soricina|ABSMS503-06|ROM 117665|Suriname.Sipaliwini
Glossophaga soricina|ABGYE616-06|ROM 112097|Guyana.Potaro-Siparuni
Glossophaga soricina|ABGYG631-08|ROM 119059|Guyana
Glossophaga soricina|ABGYG691-08|ROM 119119|Guyana
Glossophaga soricina|ABGYG907-08|ROM 119335|Guyana
Glossophaga soricina|ABRMM068-07|ROM F39146|Guyana.Potaro-Siparuni
Glossophaga soricina|ABSMS015-06|ROM 117177|Suriname.Sipaliwini
Glossophaga soricina|ABSMS467-06|ROM 117629|Suriname.Sipaliwini
Glossophaga soricina|ABGYF244-06|ROM 113826|Guyana.Demerara-Mahaica
Glossophaga soricina|ABGYF237-06|ROM 113816|Guyana.Demerara-Mahaica
Glossophaga soricina|ABGYE775-06|ROM 113376|Guyana.Upper Demerara-Berbice
Glossophaga soricina|ABGYE615-06|ROM 112096|Guyana.Potaro-Siparuni
Glossophaga soricina|ABGYE609-06|ROM 112088|Guyana.Potaro-Siparuni
Glossophaga soricina|ABGYE568-06|ROM 112043|Guyana.Potaro-Siparuni
Glossophaga soricina|ABGYE509-06|ROM 111981|Guyana.Potaro-Siparuni
Glossophaga soricina|ABGYE191-06|ROM 111637|Guyana.Potaro-Siparuni
Glossophaga soricina|ABGYE151-06|ROM 111594|Guyana.Potaro-Siparuni
Glossophaga soricina|ABGYC439-06|ROM 107263|Guyana.Potaro-Siparuni
Glossophaga soricina|ABGYB740-06|ROM 103507|Guyana.Upper Demerara-Berbice
Glossophaga soricina|ABGYB524-06|ROM 103259|Guyana.Upper Takutu-Upper Essequibo
Glossophaga soricina|ABGYA515-06|ROM 100182|Guyana.Demerara-Mahaica
Glossophaga soricina|ABGYA085-06|ROM 97917|Guyana.Upper Takutu-Upper Essequibo
Glossophaga soricina|ABGYB694-06|ROM 103458|Guyana.Upper Demerara-Berbice
Glossophaga soricina|ABGYA513-06|ROM 100180|Guyana.Demerara-Mahaica
Glossophaga soricina|ABGYA514-06|ROM 100181|Guyana.Demerara-Mahaica
Glossophaga soricina|ABGYC695-06|ROM 108095|Guyana.Cuyuni-Mazaruni
Glossophaga soricina|ABGYA512-06|ROM 100179|Guyana.Demerara-Mahaica
Glossophaga soricina|ABGYB718-06|ROM 103484|Guyana.Upper Demerara-Berbice
Glossophaga soricina|ABGYF191-06|ROM 113755|Guyana.Demerara-Mahaica
Glossophaga soricina|ABGYG423-06|ROM 116555|Guyana.Potaro-Siparuni
Glossophaga soricina|ABGYG470-06|ROM 116608|Guyana.Potaro-Siparuni
Glossophaga soricina|BCBNT658-06|ROM 113656|Guyana.Demerara-Mahaica
Glossophaga soricina|ABGYA254-06|ROM 98154|Guyana.Upper Demerara-Berbice
Glossophaga soricina|ABGYA255-06|ROM 98155|Guyana.Upper Demerara-Berbice
Glossophaga soricina|ABGYB614-06|ROM 103365|Guyana.Upper Demerara-Berbice
Glossophaga soricina|ABGYG626-08|ROM 119054|Guyana
Glossophaga soricina|ABGYG629-08|ROM 119057|Guyana
Glossophaga soricina|BCBNT853-06|ROM 114224|Suriname.Brokopondo
Lichonycteris obscura|BCBN775-05|ROM 105140|Ecuador.Napo
Lichonycteris obscura|BCBN784-05|ROM 105173|Ecuador.Napo
Choeroniscus godmani|ABSMS239-06|ROM 117401|Suriname.Sipaliwini
Choeroniscus sp.|BCBN778-05|ROM 105150|Ecuador.Napo
Choeroniscus sp.|BCBN853-05|ROM 105574|Ecuador.Napo
Choeroniscus sp.|BCBN887-05|ROM 105710|Ecuador.Napo
Choeroniscus sp.|BCBN957-05|ROM 105931|Ecuador.Napo
Choeroniscus minor|BCBNT126-06|ROM 107343|Guyana.Potaro-Siparuni
Choeroniscus minor|BCBNT129-06|ROM 107368|Guyana.Potaro-Siparuni
Choeroniscus minor|ABGYB459-06|ROM 103193|Guyana.Upper Takutu-Upper Essequibo
Choeroniscus minor|ABGYG209-06|ROM 115798|Guyana.Potaro-Siparuni
Choeroniscus minor|BCBNT032-06|ROM 106614|Guyana.Upper Takutu-Upper Essequibo
Choeroniscus minor|BCBNT284-06|ROM 108468|Guyana.Potaro-Siparuni
Choeroniscus minor|BCBNT291-06|ROM 108486|Guyana.Potaro-Siparuni
Hylonycteris underwoodi|BCBN673-05|ROM 104298|Panama.Chiriqui
Hylonycteris underwoodi|BCBNT406-06|ROM 108286|Costa Rica.Limon
Hylonycteris underwoodi|ABSCA141-06|ROM F44058|Costa Rica.Limon
Hylonycteris underwoodi|BCBNT417-06|ROM 108297|Costa Rica.Limon
Trinycteris nicefori|ABECA176-06|ROM 104489|Ecuador.Napo
Trinycteris nicefori|ABECB093-08|ROM 118755|Ecuador
Trinycteris nicefori|BCBN752-05|ROM 104472|Ecuador.Napo
Trinycteris nicefori|ABECA315-06|ROM 105195|Ecuador.Napo
Trinycteris nicefori|ABECA694-06|ROM F40044|Ecuador.Napo
Trinycteris nicefori|BCBN870-05|ROM 105668|Ecuador.Napo
Trinycteris nicefori|BCBN902-05|ROM 105757|Ecuador.Napo
Trinycteris nicefori|BCBN974-05|ROM 106022|Ecuador.Napo
Trinycteris nicefori|ABGYA288-06|ROM 98207|Guyana.Demerara-Mahaica
Trinycteris nicefori|BCBNT452-06|ROM 108330|Costa Rica.Limon
Trinycteris nicefori|BCBNC205-06|ROM 116712|Guyana.Potaro-Siparuni
Trinycteris nicefori|BCBNC216-06|ROM 116742|Guyana.Potaro-Siparuni
Trinycteris nicefori|BCBNT385-06|ROM 109139|Guyana.Potaro-Siparuni
Trinycteris nicefori|ABGYA051-06|ROM 97853|Guyana.Upper Takutu-Upper Essequibo
Trinycteris nicefori|ABGYA053-06|ROM 97856|Guyana.Upper Takutu-Upper Essequibo
Trinycteris nicefori|ABGYB729-06|ROM 103496|Guyana.Upper Demerara-Berbice
Trinycteris nicefori|BCBNT087-06|ROM 107079|Guyana.Potaro-Siparuni
Trinycteris nicefori|BCBNT789-06|ROM 114049|Suriname.Brokopondo
Trinycteris nicefori|ABSMS479-06|ROM 117641|Suriname.Sipaliwini
Trinycteris nicefori|ABGYF132-06|ROM 113680|Guyana.Demerara-Mahaica
Trinycteris nicefori|ABGYD348-06|ROM 108689|Guyana.Potaro-Siparuni

Trinycteris nicefori|ABSMS479-06|ROM 117641|Suriname.Sipaliwini
Trinycteris nicefori|ABGYF132-06|ROM 113680|Guyana.Demerara-Mahaica
Trinycteris nicefori|ABGYD348-06|ROM 108689|Guyana.Potaro-Siparuni
Trinycteris nicefori|ABGYC606-06|ROM F38824|Guyana.Potaro-Siparuni
Trinycteris nicefori|ABGYC605-06|ROM F38823|Guyana.Potaro-Siparuni
Trinycteris nicefori|ABGYB658-06|ROM 103416|Guyana.Upper Demerara-Berbice
Trinycteris nicefori|ABGYB478-06|ROM 103213|Guyana.Upper Takutu-Upper Essequibo
Trinycteris nicefori|ABGYB238-06|ROM 102940|Guyana.Upper Takutu-Upper Essequibo
Trinycteris nicefori|ABGYA119-06|ROM 97959|Guyana.Upper Takutu-Upper Essequibo
Trinycteris nicefori|ABGYE825-06|ROM 113433|Guyana.Upper Demerara-Berbice
Trinycteris nicefori|ABRMM081-07|ROM F39690|Guyana.Potaro-Siparuni
Trinycteris nicefori|ABSRA474-06|ROM 117064|Suriname.Nickerie
Trinycteris nicefori|BCBN546-05|ROM 103415|Guyana.Upper Demerara-Berbice
Trinycteris nicefori|BCBNT151-06|ROM 107475|Guyana.Potaro-Siparuni
Trinycteris nicefori|BCBNT382-06|ROM 109100|Guyana.Potaro-Siparuni
Trinycteris nicefori|BCBNT801-06|ROM 114073|Suriname.Brokopondo
Trinycteris nicefori|BCBNT866-06|ROM 114352|Suriname.Brokopondo
Glyphonycteris sylvestris|BCBNT034-06|ROM 106626|Guyana.Upper Takutu-Upper Essequibo
Glyphonycteris sylvestris|BCBNT146-06|ROM 107445|Guyana.Potaro-Siparuni
Glyphonycteris sylvestris|BCBNT387-06|ROM 109156|Guyana.Potaro-Siparuni
Glyphonycteris sylvestris|BCBNT516-06|ROM 111823|Guyana.Potaro-Siparuni
Glyphonycteris daviesi|BCBN585-05|ROM 104042|Ecuador.Napo
Glyphonycteris daviesi|BCBN815-05|ROM 105275|Ecuador.Napo
Glyphonycteris daviesi|BCBN834-05|ROM 105495|Ecuador.Napo
Glyphonycteris daviesi|BCBN888-05|ROM 105711|Ecuador.Napo
Glyphonycteris daviesi|BCBNT581-06|ROM 112678|Guyana.Demerara-Mahaica
Glyphonycteris daviesi|BCBNC213-06|ROM 116730|Guyana.Potaro-Siparuni
Glyphonycteris daviesi|BCBNT790-06|ROM 114050|Suriname.Brokopondo
Glyphonycteris daviesi|BCBNT807-06|ROM 114114|Suriname.Brokopondo
Glyphonycteris daviesi|BCBNT946-06|ROM 115669|Guyana.Potaro-Siparuni
Lionycteris spurrelli|ABGYC720-06|ROM F39776|Guyana.Cuyuni-Mazaruni
Lionycteris spurrelli|ABGYC171-06|ROM 106702|Guyana.Upper Takutu-Upper Essequibo
Lionycteris spurrelli|BCBNT124-06|ROM 107332|Guyana.Potaro-Siparuni
Lionycteris spurrelli|BCBNT123-06|ROM 107304|Guyana.Potaro-Siparuni
Lionycteris spurrelli|ABGYC288-06|ROM 107086|Guyana.Potaro-Siparuni
Lionycteris spurrelli|ABGYG421-06|ROM 116553|Guyana.Potaro-Siparuni
Lionycteris spurrelli|ABGYE305-06|ROM 111755|Guyana.Potaro-Siparuni
Lionycteris spurrelli|ABGYC596-06|ROM 107452|Guyana.Potaro-Siparuni
Lionycteris spurrelli|ABGYG475-06|ROM 116614|Guyana.Potaro-Siparuni
Lionycteris spurrelli|ABGYG466-06|ROM 116604|Guyana.Potaro-Siparuni
Lionycteris spurrelli|ABGYG501-06|ROM 116643|Guyana.Potaro-Siparuni
Lionycteris spurrelli|ABGYG502-06|ROM 116644|Guyana.Potaro-Siparuni
Lionycteris spurrelli|BCBNT139-06|ROM 107430|Guyana.Potaro-Siparuni
Lionycteris spurrelli|ABGYG498-06|ROM 116640|Guyana.Potaro-Siparuni
Lionycteris spurrelli|BCBNT850-06|ROM 114219|Suriname.Brokopondo
Lionycteris spurrelli|ABGYC172-06|ROM 106703|Guyana.Upper Takutu-Upper Essequibo
Lionycteris spurrelli|ABGYF299-06|ROM 114668|Guyana.Potaro-Siparuni
Lionycteris spurrelli|ABGYG427-06|ROM 116562|Guyana.Potaro-Siparuni
Lionycteris spurrelli|ABGYG503-06|ROM 116645|Guyana.Potaro-Siparuni
Lionycteris spurrelli|ABGYG420-06|ROM 116552|Guyana.Potaro-Siparuni
Lionycteris spurrelli|ABGYF074-06|ROM 113601|Guyana.Upper Takutu-Upper Essequibo
Lionycteris spurrelli|ABGYG488-06|ROM 116628|Guyana.Potaro-Siparuni
Lionycteris spurrelli|ABGYF670-06|ROM 115354|Guyana.Cuyuni-Mazaruni
Lionycteris spurrelli|ABGYC696-06|ROM 108096|Guyana.Cuyuni-Mazaruni
Lionycteris spurrelli|ABGYG486-06|ROM 116626|Guyana.Potaro-Siparuni
Lionycteris spurrelli|ABGYD376-06|ROM 108721|Guyana.Potaro-Siparuni
Lionycteris spurrelli|ABGYD340-06|ROM 108681|Guyana.Potaro-Siparuni
Lionycteris spurrelli|ABGYG484-06|ROM 116624|Guyana.Potaro-Siparuni
Lionycteris spurrelli|BCBN135-05|ROM 97990|Guyana.Potaro-Siparuni
Lionycteris spurrelli|ABGYG487-06|ROM 116627|Guyana.Potaro-Siparuni
Lionycteris spurrelli|ABGYD108-06|ROM 108406|Guyana.Potaro-Siparuni
Lionycteris spurrelli|ABGYG442-06|ROM 116578|Guyana.Potaro-Siparuni
Lionycteris spurrelli|ABGYG485-06|ROM 116625|Guyana.Potaro-Siparuni
Lionycteris spurrelli|BCBNT138-06|ROM 107420|Guyana.Potaro-Siparuni
Lionycteris spurrelli|ABGYC598-06|ROM 107454|Guyana.Potaro-Siparuni
Lionycteris spurrelli|ABGYE032-06|ROM 109318|Guyana.Potaro-Siparuni
Lionycteris spurrelli|ABGYC587-06|ROM 107442|Guyana.Potaro-Siparuni
Lionycteris spurrelli|ABGYC308-06|ROM 107119|Guyana.Potaro-Siparuni
Lionycteris spurrelli|ABGYC300-06|ROM 107105|Guyana.Potaro-Siparuni
Lionycteris spurrelli|ABGYC173-06|ROM 106704|Guyana.Upper Takutu-Upper Essequibo
Lionycteris spurrelli|ABGYC204-06|ROM 106743|Guyana.Upper Takutu-Upper Essequibo
Lionycteris spurrelli|ABGYE530-06|ROM 112003|Guyana.Potaro-Siparuni
Lionycteris spurrelli|ABGYG399-06|ROM 116527|Guyana.Potaro-Siparuni
Lionycteris spurrelli|ABGYG467-06|ROM 116605|Guyana.Potaro-Siparuni
Lionycteris spurrelli|ABGYG469-06|ROM 116607|Guyana.Potaro-Siparuni
Lionycteris spurrelli|BCBNC065-06|ROM 115170|Guyana.Cuyuni-Mazaruni
Lionycteris spurrelli|BCBNT814-06|ROM 114124|Suriname.Brokopondo
Lionycteris spurrelli|ABGYC166-06|ROM 106693|Guyana.Upper Takutu-Upper Essequibo
Lionycteris spurrelli|ABGYG422-06|ROM 116554|Guyana.Potaro-Siparuni
Lionycteris spurrelli|ABGYD263-06|ROM 108599|Guyana.Potaro-Siparuni
Lionycteris spurrelli|ABGYD207-06|ROM 108537|Guyana.Potaro-Siparuni
Lionycteris spurrelli|ABGYC808-06|ROM 108206|Guyana.Cuyuni-Mazaruni
Lionycteris spurrelli|ABGYC770-06|ROM 108172|Guyana.Cuyuni-Mazaruni
Lionycteris spurrelli|ABGYC734-06|ROM 108134|Guyana.Cuyuni-Mazaruni
Lionycteris spurrelli|ABGYC713-06|ROM 108113|Guyana.Cuyuni-Mazaruni
Lionycteris spurrelli|ABGYC301-06|ROM 107106|Guyana.Potaro-Siparuni
Lionycteris spurrelli|ABGYC174-06|ROM 106705|Guyana.Upper Takutu-Upper Essequibo
Lionycteris spurrelli|BCBNT117-06|ROM 107281|Guyana.Potaro-Siparuni
Lionycteris spurrelli|ABGYC644-06|ROM 107462|Guyana.Potaro-Siparuni
Lionycteris spurrelli|ABRMM079-07|ROM F39677|Guyana.Potaro-Siparuni
Lionycteris spurrelli|BCBNT859-06|ROM 114239|Suriname.Brokopondo
Lonchophylla mordax|BCBN924-05|ROM 105798|Ecuador.Esmeraldas
Lonchophylla thomasi|BCBN982-05|ROM 106112|Ecuador.Napo
Lonchophylla thomasi|ABECA411-06|ROM 105311|Ecuador.Napo
Lonchophylla thomasi|ABECA069-06|ROM 104062|Ecuador.Napo
Lonchophylla thomasi|ABECA546-06|ROM 105575|Ecuador.Napo
Lonchophylla thomasi|BCBN595-05|ROM 104063|Ecuador.Napo
Lonchophylla thomasi|ABECA440-06|ROM 105342|Ecuador.Napo
Lonchophylla thomasi|BCBN826-05|ROM 105352|Ecuador.Napo
Lonchophylla thomasi|ABECB091-08|ROM 118753|Ecuador
Lonchophylla thomasi|BCBN577-05|ROM 104031|Ecuador.Napo
Lonchophylla thomasi|ABECA142-06|ROM 104441|Ecuador.Napo
Lonchophylla thomasi|BCBN593-05|ROM 104060|Ecuador.Napo

[illegible]
Lonchophylla thomasi|BCBN577-05|ROM 104031|Ecuador.Napo
Lonchophylla thomasi|ABECA142-06|ROM 104441|Ecuador.Napo
Lonchophylla thomasi|BCBN593-05|ROM 104060|Ecuador.Napo
Lonchophylla thomasi|BCBN594-05|ROM 104061|Ecuador.Napo
Lonchophylla thomasi|BCBN899-05|ROM 105750|Ecuador.Napo
Lonchophylla thomasi|ABECA830-06|ROM 105999|Ecuador.Napo
Lonchophylla thomasi|ABECA884-06|ROM 106063|Ecuador.Napo
Lonchophylla thomasi|ABECA318-06|ROM 105197|Ecuador.Napo
Lonchophylla thomasi|BCBN782-05|ROM 105164|Ecuador.Napo
Lonchophylla thomasi|BCBN903-05|ROM 105762|Ecuador.Napo
Lonchophylla thomasi|BCBN949-05|ROM 105903|Ecuador.Napo
Lonchophylla thomasi|ABECA471-06|ROM 105494|Ecuador.Napo
Lonchophylla thomasi|ABECA499-06|ROM 105527|Ecuador.Napo
Lonchophylla thomasi|ABECA070-06|ROM 104064|Ecuador.Napo
Lonchophylla thomasi|ABECB124-08|ROM 118786|Ecuador
Lonchophylla thomasi|BCBN832-05|ROM 105378|Ecuador.Napo
Lonchophylla thomasi|BCBN858-05|ROM 105610|Ecuador.Napo
Lonchophylla thomasi|BCBN780-05|ROM 105155|Ecuador.Napo
Lonchophylla thomasi|ABECA768-06|ROM 105923|Ecuador.Napo
Lonchophylla thomasi|BCBN953-05|ROM 105922|Ecuador.Napo
Lonchophylla thomasi|BCBN985-05|ROM 106131|Ecuador.Napo
Lonchophylla thomasi|ABECA848-06|ROM 106023|Ecuador.Napo
Lonchophylla thomasi|BCBNT200-06|ROM 107906|Venezuela.Bolivar
Lonchophylla thomasi|BCBNT019-06|ROM 106565|Guyana.Upper Takutu-Upper Essequibo
Lonchophylla thomasi|ABSRA263-06|ROM 116851|Suriname.Nickerie
Lonchophylla thomasi|ABSRA463-06|ROM 117053|Suriname.Nickerie
Lonchophylla thomasi|BCBNT243-06|ROM 108158|Guyana.Cuyuni-Mazaruni
Lonchophylla thomasi|BCBNT246-06|ROM 108173|Guyana.Cuyuni-Mazaruni
Lonchophylla thomasi|BCBNT254-06|ROM 108207|Guyana.Cuyuni-Mazaruni
Lonchophylla thomasi|BCBNC055-06|ROM 115077|Guyana.Cuyuni-Mazaruni
Lonchophylla thomasi|ABGYC256-06|ROM 107042|Guyana.Potaro-Siparuni
Lonchophylla thomasi|ABSRA274-06|ROM 116863|Suriname.Nickerie
Lonchophylla thomasi|BCBN363-05|ROM 101120|Guyana.Barima-Waini
Lonchophylla thomasi|BCBNC119-06|ROM 115771|Guyana.Potaro-Siparuni
Lonchophylla thomasi|BCBNC209-06|ROM 116725|Guyana.Potaro-Siparuni
Lonchophylla thomasi|ABSRA265-06|ROM 116853|Suriname.Nickerie
Lonchophylla thomasi|ABGYG998-08|ROM 118922|Guyana
Lonchophylla thomasi|ABGYG1062-08|ROM 118986|Guyana
Lonchophylla thomasi|BCBNT617-06|ROM 113531|Guyana.Upper Takutu-Upper Essequibo
Lonchophylla thomasi|BCBNT773-06|ROM 113992|Suriname.Brokopondo
Lonchophylla thomasi|BCBNT836-06|ROM 114187|Suriname.Brokopondo
Lonchophylla thomasi|ABGYC735-06|ROM 108135|Guyana.Cuyuni-Mazaruni
Lonchophylla thomasi|ABGYB055-06|ROM 101027|Guyana.Barima-Waini
Lonchophylla thomasi|BCBN162-05|ROM 98741|Guyana.Barima-Waini
Lonchophylla thomasi|ABGYA463-06|ROM 98873|Guyana.Barima-Waini
Lonchophylla thomasi|ABGYB011-06|ROM 100979|Guyana.Barima-Waini
Lonchophylla thomasi|BCBN347-05|ROM 100966|Guyana.Barima-Waini
Lonchophylla thomasi|BCBN348-05|ROM 100978|Guyana.Barima-Waini
Lonchophylla thomasi|BCBN281-05|ROM 100204|Guyana.East Berbice-Corentyne
Lonchophylla thomasi|ABGYC080-06|ROM 106577|Guyana.Upper Takutu-Upper Essequibo
Lonchophylla thomasi|BCBNC114-06|ROM 115761|Guyana.Potaro-Siparuni
Lonchophylla thomasi|BCBNC115-06|ROM 115762|Guyana.Potaro-Siparuni
Lonchophylla thomasi|ABGYC390-06|ROM 107209|Guyana.Potaro-Siparuni
Lonchophylla thomasi|BCBNC176-06|ROM 116493|Guyana.Potaro-Siparuni
Lonchophylla thomasi|ABGYD135-06|ROM 108438|Guyana.Potaro-Siparuni
Lonchophylla thomasi|ABSRA494-06|ROM 117084|Suriname.Nickerie
Lonchophylla thomasi|ABGYG1011-08|ROM 118935|Guyana
Lonchophylla thomasi|ABSMS396-06|ROM 117558|Suriname.Sipaliwini
Lonchophylla thomasi|ABGYC421-06|ROM 107243|Guyana.Potaro-Siparuni
Lonchophylla thomasi|BCBNT762-06|ROM 113963|Suriname.Brokopondo
Lonchophylla thomasi|ABGYA537-06|ROM 100208|Guyana.East Berbice-Corentyne
Lonchophylla thomasi|ABSMS008-06|ROM 117170|Suriname.Sipaliwini
Lonchophylla thomasi|ABSRA462-06|ROM 117052|Suriname.Nickerie
Lonchophylla thomasi|ABGYB268-06|ROM 102972|Guyana.Upper Takutu-Upper Essequibo
Lonchophylla thomasi|ABGYE245-06|ROM 111693|Guyana.Potaro-Siparuni
Lonchophylla thomasi|BCBN284-05|ROM 100223|Guyana.East Berbice-Corentyne
Lonchophylla thomasi|ABGYE306-06|ROM 111756|Guyana.Potaro-Siparuni
Lonchophylla thomasi|ABSMS299-06|ROM 117461|Suriname.Sipaliwini
Lonchophylla thomasi|ABGYE331-06|ROM 111781|Guyana.Potaro-Siparuni
Lonchophylla thomasi|ABGYB522-06|ROM 103257|Guyana.Upper Takutu-Upper Essequibo
Lonchophylla thomasi|ABGYE231-06|ROM 111679|Guyana.Potaro-Siparuni
Lonchophylla thomasi|ABGYE549-06|ROM 112022|Guyana.Potaro-Siparuni
Lonchophylla thomasi|ABGYE597-06|ROM 112076|Guyana.Potaro-Siparuni
Lonchophylla thomasi|ABGYA741-06|ROM 100447|Guyana.East Berbice-Corentyne
Lonchophylla thomasi|ABSMS420-06|ROM 117582|Suriname.Sipaliwini
Lonchophylla thomasi|ABSRA260-06|ROM 116848|Suriname.Nickerie
Lonchophylla thomasi|ABSRA261-06|ROM 116849|Suriname.Nickerie
Lonchophylla thomasi|BCBN280-05|ROM 100203|Guyana.East Berbice-Corentyne
Lonchophylla thomasi|ABSMS439-06|ROM 117601|Suriname.Sipaliwini
Lonchophylla thomasi|ABSMS395-06|ROM 117557|Suriname.Sipaliwini
Lonchophylla thomasi|ABSMS203-06|ROM 117365|Suriname.Sipaliwini
Lonchophylla thomasi|ABSMS038-06|ROM 117200|Suriname.Sipaliwini
Lonchophylla thomasi|ABSMS007-06|ROM 117169|Suriname.Sipaliwini
Lonchophylla thomasi|ABRMM080-07|ROM F39685|Guyana.Potaro-Siparuni
Lonchophylla thomasi|ABGYG999-08|ROM 118923|Guyana
Lonchophylla thomasi|ABGYG1063-08|ROM 118987|Guyana
Lonchophylla thomasi|ABGYE324-06|ROM 111774|Guyana.Potaro-Siparuni
Lonchophylla thomasi|ABGYE346-06|ROM 111796|Guyana.Potaro-Siparuni
Lonchophylla thomasi|ABGYE270-06|ROM 111719|Guyana.Potaro-Siparuni
Lonchophylla thomasi|ABGYE293-06|ROM 111742|Guyana.Potaro-Siparuni
Lonchophylla thomasi|ABGYG147-06|ROM 115707|Guyana.Potaro-Siparuni
Lonchophylla thomasi|ABSRA259-06|ROM 116847|Suriname.Nickerie
Lonchophylla thomasi|ABSRA262-06|ROM 116850|Suriname.Nickerie
Lonchophylla thomasi|ABSRA495-06|ROM 117085|Suriname.Nickerie
Lonchophylla thomasi|BCBNT605-06|ROM 113495|Guyana.Upper Takutu-Upper Essequibo
Lonchophylla thomasi|BCBNT751-06|ROM 113941|Suriname.Brokopondo
Lonchophylla thomasi|BCBNT955-06|ROM 115706|Guyana.Potaro-Siparuni
Lonchophylla thomasi|BCBNT061-06|ROM 106734|Guyana.Upper Takutu-Upper Essequibo
Lonchophylla thomasi|BCBNT716-06|ROM 113885|Suriname.Brokopondo
Lonchophylla thomasi|ABGYD752-06|ROM 109170|Guyana.Potaro-Siparuni
Lonchophylla thomasi|ABGYD592-06|ROM 109014|Guyana.Potaro-Siparuni
Lonchophylla thomasi|ABGYC471-06|ROM 107303|Guyana.Potaro-Siparuni
[illegible]

Lonchophylla thomasi|ABGYD752-06|ROM 109170|Guyana.Potaro-Siparuni
Lonchophylla thomasi|ABGYD592-06|ROM 109014|Guyana.Potaro-Siparuni
Lonchophylla thomasi|ABGYC471-06|ROM 107303|Guyana.Potaro-Siparuni
Lonchophylla thomasi|ABGYD851-06|ROM 109278|Guyana.Potaro-Siparuni
Lonchophylla thomasi|ABGYE330-06|ROM 111780|Guyana.Potaro-Siparuni
Lonchophylla thomasi|ABGYE804-06|ROM 113406|Guyana.Upper Demerara-Berbice
Lonchophylla thomasi|BCBN362-05|ROM 101110|Guyana.Barima-Waini
Lonchophylla thomasi|BCBNT644-06|ROM 113600|Guyana.Upper Takutu-Upper Essequibo
Lonchophylla thomasi|ABGYC562-06|ROM 107406|Guyana.Potaro-Siparuni
Lonchophylla thomasi|ABGYD561-06|ROM 108931|Guyana.Potaro-Siparuni
Lonchophylla thomasi|BCBNT950-06|ROM 115685|Guyana.Potaro-Siparuni
Lonchophylla thomasi|ABSRA264-06|ROM 116852|Suriname.Nickerie
Lonchophylla thomasi|ABGYA562-06|ROM 100237|Guyana.East Berbice-Corentyne
Lonchophylla thomasi|ABGYG148-06|ROM 115708|Guyana.Potaro-Siparuni
Lonchophylla thomasi|BCBN310-05|ROM 100362|Guyana.East Berbice-Corentyne
Lonchophylla thomasi|BCBNT582-06|ROM 112679|Guyana.Demerara-Mahaica
Lonchophylla thomasi|ABGYD071-06|ROM 108368|Guyana.Potaro-Siparuni
Lonchophylla thomasi|BCBNT586-06|ROM 113364|Guyana.Upper Demerara-Berbice
Lonchophylla thomasi|ABGYE548-06|ROM 112021|Guyana.Potaro-Siparuni
Lonchophylla thomasi|BCBN536-05|ROM 103352|Guyana.Upper Demerara-Berbice
Lonchophylla thomasi|BCBNT940-06|ROM 115640|Guyana.Potaro-Siparuni
Lonchophylla thomasi|BCBNT953-06|ROM 115704|Guyana.Potaro-Siparuni
Lonchophylla thomasi|BCBNC058-06|ROM 115089|Guyana.Cuyuni-Mazaruni
Lonchophylla thomasi|BCBNT023-06|ROM 106586|Guyana.Upper Takutu-Upper Essequibo
Lonchophylla thomasi|BCBNT618-06|ROM 113532|Guyana.Upper Takutu-Upper Essequibo
Lonchophylla thomasi|BCBNT627-06|ROM 113561|Guyana.Upper Takutu-Upper Essequibo
Lonchophylla thomasi|BCBNT649-06|ROM 113622|Guyana.Demerara-Mahaica
Lonchophylla thomasi|BCBNT928-06|ROM 115616|Guyana.Essequibo Islands-West Demerara
Lonchophylla thomasi|ABSMS434-06|ROM 117596|Suriname.Sipaliwini
Lonchophylla thomasi|ABSRA422-06|ROM 117011|Suriname.Nickerie
Lonchophylla thomasi|ABSMS240-06|ROM 117402|Suriname.Sipaliwini
Lonchophylla thomasi|ABSMS251-06|ROM 117413|Suriname.Sipaliwini
Lonchophylla thomasi|ABGYF059-06|ROM 113572|Guyana.Upper Takutu-Upper Essequibo
Lonchophylla thomasi|ABGYG130-06|ROM 115686|Guyana.Potaro-Siparuni
Lonchophylla thomasi|ABGYB052-06|ROM 101024|Guyana.Barima-Waini
Lonchophylla thomasi|ABGYB382-06|ROM 103089|Guyana.Upper Takutu-Upper Essequibo
Lonchophylla thomasi|ABGYB423-06|ROM 103131|Guyana.Upper Takutu-Upper Essequibo
Lonchophylla thomasi|ABGYB445-06|ROM 103159|Guyana.Upper Takutu-Upper Essequibo
Lonchophylla thomasi|ABGYC389-06|ROM 107208|Guyana.Potaro-Siparuni
Lonchophylla thomasi|ABGYC420-06|ROM 107242|Guyana.Potaro-Siparuni
Lonchophylla thomasi|ABGYD181-06|ROM 108507|Guyana.Potaro-Siparuni
Lonchophylla thomasi|ABGYD182-06|ROM 108508|Guyana.Potaro-Siparuni
Lonchophylla thomasi|ABGYD562-06|ROM 108932|Guyana.Potaro-Siparuni
Lonchophylla thomasi|ABGYE608-06|ROM 112087|Guyana.Potaro-Siparuni
Lonchophylla thomasi|BCBNT987-06|ROM 114652|Guyana.Potaro-Siparuni
Carollia castanea|ABECA146-06|ROM 104447|Ecuador.Napo
Carollia castanea|ABECA588-06|ROM 105620|Ecuador.Napo
Carollia castanea|ABECA751-06|ROM 105900|Ecuador.Napo
Carollia castanea|ABECA141-06|ROM 104439|Ecuador.Napo
Carollia castanea|ABECA317-06|ROM F37513|Ecuador.Napo
Carollia castanea|ABECA753-06|ROM 105904|Ecuador.Napo
Carollia castanea|ABECA790-06|ROM 105954|Ecuador.Napo
Carollia castanea|ABECA151-06|ROM 104456|Ecuador.Napo
Carollia castanea|ABECA447-06|ROM 105353|Ecuador.Napo
Carollia castanea|ABECA031-06|ROM F37106|Ecuador.Napo
Carollia castanea|ABECA777-06|ROM 105940|Ecuador.Napo
Carollia castanea|ABECB088-08|ROM 118750|Ecuador
Carollia castanea|ABECA750-06|ROM 105899|Ecuador.Napo
Carollia castanea|ABECA448-06|ROM 105354|Ecuador.Napo
Carollia castanea|ABECA915-06|ROM 106095|Ecuador.Napo
Carollia castanea|ABECA030-06|ROM F37105|Ecuador.Napo
Carollia castanea|ABECA152-06|ROM 104457|Ecuador.Napo
Carollia castanea|BCBN555-05|ROM 103979|Ecuador.Napo
Carollia castanea|ABECA119-06|ROM 104413|Ecuador.Napo
Carollia castanea|ABECA584-06|ROM 105616|Ecuador.Napo
Carollia castanea|ABECA916-06|ROM 106096|Ecuador.Napo
Carollia castanea|ABECA404-06|ROM 105300|Ecuador.Napo
Carollia castanea|ABECA007-06|ROM F37071|Ecuador.Napo
Carollia castanea|ABECA514-06|ROM 105548|Ecuador.Napo
Carollia castanea|BCBN557-05|ROM 103983|Ecuador.Napo
Carollia castanea|ABECA583-06|ROM 105615|Ecuador.Napo
Carollia castanea|ABECA617-06|ROM 105656|Ecuador.Napo
Carollia castanea|ABECA309-06|ROM 105184|Ecuador.Napo
Carollia castanea|ABECA749-06|ROM 105898|Ecuador.Napo
Carollia castanea|ABECA441-06|ROM 105344|Ecuador.Napo
Carollia castanea|ABECA120-06|ROM F37247|Ecuador.Napo
Carollia castanea|ABECA576-06|ROM F37849|Ecuador.Napo
Carollia castanea|ABECA585-06|ROM 105617|Ecuador.Napo
Carollia castanea|BCBN558-05|ROM 103984|Ecuador.Napo
Carollia castanea|ABSCA045-06|ROM 104224|Panama.Canal Zone
Carollia castanea|ABSCA108-06|ROM 104341|Panama.Darien
Carollia castanea|ABSCA116-06|ROM 104353|Panama.Darien
Carollia castanea|ABSCA041-06|ROM 104212|Panama.Canal Zone
Carollia castanea|ABSCA073-06|ROM 104275|Panama
Carollia castanea|BCBN709-05|ROM 104364|Panama.Darien
Carollia castanea|BCBN918-05|ROM 105792|Ecuador.Esmeraldas
Carollia castanea|BCBN937-05|ROM 105814|Ecuador.Esmeraldas
Carollia castanea|ABECA720-06|ROM 105816|Ecuador.Esmeraldas
Carollia castanea|BCBN916-05|ROM 105790|Ecuador.Esmeraldas
Carollia castanea|BCBN917-05|ROM 105791|Ecuador.Esmeraldas
Carollia castanea|BCBN938-05|ROM 105815|Ecuador.Esmeraldas
Carollia castanea|BCBN622-05|ROM 104222|Panama.Canal Zone
Carollia castanea|ABSCA115-06|ROM F38211|Panama.Darien
Carollia castanea|ABSCA042-06|ROM 104213|Panama.Canal Zone
Carollia castanea|ABSCA092-06|ROM 104316|Panama.Chiriqui
Carollia castanea|ABSCA091-06|ROM 104315|Panama.Chiriqui
Carollia castanea|BCBN679-05|ROM 104305|Panama.Chiriqui
Carollia castanea|BCBN690-05|ROM 104330|Panama.Chiriqui
Carollia castanea|ABSCA072-06|ROM F38119|Panama
Carollia castanea|BCBNT411-06|ROM 108291|Costa Rica.Limon
Carollia castanea|BCBNT424-06|ROM 108304|Costa Rica.Limon
Carollia castanea|ABSCA134-06|ROM F44030|Costa Rica.Limon
Carollia castanea|BCBNT423-06|ROM 108303|Costa Rica.Limon

Carollia castanea|BCBNT424-06|ROM 108304|Costa Rica.Limon
Carollia castanea|ABSCA134-06|ROM F44030|Costa Rica.Limon
Carollia castanea|BCBNT423-06|ROM 108303|Costa Rica.Limon
Carollia castanea|BCBNT429-06|ROM 103587|Costa Rica.Limon
Carollia subrufa|ABCSA213-06|ROM 98485|Guatemala.El Progreso
Carollia subrufa|ABCSA214-06|ROM 98486|Guatemala.El Progreso
Carollia subrufa|ABCSA736-06|ROM 101318|El Salvador.Ahuachapan
Carollia subrufa|BCBN427-05|ROM 101342|El Salvador.Ahuachapan
Carollia subrufa|ABCSA703-06|ROM 101264|El Salvador.Ahuachapan
Carollia subrufa|ABCSA750-06|ROM 101348|El Salvador.Ahuachapan
Carollia subrufa|BCBN252-05|ROM 99674|Guatemala.El Progreso
Carollia subrufa|ABCSA217-06|ROM 98489|Guatemala.El Progreso
Carollia subrufa|BCBN254-05|ROM 99676|Guatemala.El Progreso
Carollia subrufa|BCBN406-05|ROM 101299|El Salvador.Ahuachapan
Carollia subrufa|BCBN413-05|ROM 101316|El Salvador.Ahuachapan
Carollia subrufa|BCBN376-05|ROM 101229|El Salvador.Ahuachapan
Carollia subrufa|BCBN265-05|ROM 99678|Guatemala.El Progreso
Carollia subrufa|BCBN264-05|ROM 99677|Guatemala.El Progreso
Carollia subrufa|BCBN253-05|ROM 99675|Guatemala.El Progreso
Carollia subrufa|ABCSA742-06|ROM 101328|El Salvador.Ahuachapan
Carollia subrufa|ABCSA735-06|ROM 101317|El Salvador.Ahuachapan
Carollia subrufa|ABCSA218-06|ROM 98490|Guatemala.El Progreso
Carollia subrufa|ABCSA216-06|ROM 98488|Guatemala.El Progreso
Carollia subrufa|ABCSA215-06|ROM FN31476|Guatemala.El Progreso
Carollia subrufa|BCBN516-05|ROM 97630|Mexico.Chiapas
Carollia subrufa|BCBN517-05|ROM 97631|Mexico.Chiapas
Carollia subrufa|BCBN518-05|ROM 97632|Mexico.Chiapas
Carollia sowelli|ABCSA297-06|ROM 99273|Guatemala.Peten
Carollia sowelli|ABMXC570-06|ROM 97511|Mexico.Quintana Roo
Carollia sowelli|BCBN174-05|ROM 99219|Guatemala.Peten
Carollia sowelli|ABMXC572-06|ROM 97513|Mexico.Quintana Roo
Carollia sowelli|ABMXC565-06|ROM 97506|Mexico.Quintana Roo
Carollia sowelli|ABCSA260-06|ROM 99221|Guatemala.Peten
Carollia sowelli|ABCSA440-06|ROM 99585|Guatemala.Peten
Carollia sowelli|ABCSA442-06|ROM 99587|Guatemala.Peten
Carollia sowelli|BCBN175-05|ROM 99220|Guatemala.Peten
Carollia sowelli|ABCSA490-06|ROM 99476|Guatemala.Peten
Carollia sowelli|ABCSA287-06|ROM 99258|Guatemala.Peten
Carollia sowelli|ABCSA294-06|ROM 99270|Guatemala.Peten
Carollia sowelli|ABCSA261-06|ROM 99222|Guatemala.Peten
Carollia sowelli|ABCSA378-06|ROM 99392|Guatemala.Peten
Carollia sowelli|ABCSA443-06|ROM 99588|Guatemala.Peten
Carollia sowelli|ABMXC562-06|ROM 97503|Mexico.Quintana Roo
Carollia sowelli|ABMXC568-06|ROM 97509|Mexico.Quintana Roo
Carollia sowelli|ABMXC569-06|ROM 97510|Mexico.Quintana Roo
Carollia sowelli|ABMXC571-06|ROM 97512|Mexico.Quintana Roo
Carollia sowelli|ABMXC573-06|ROM 97514|Mexico.Quintana Roo
Carollia sowelli|BCBN184-05|ROM 99253|Guatemala.Peten
Carollia sowelli|BCBN185-05|ROM 99254|Guatemala.Peten
Carollia sowelli|BCBN186-05|ROM 99255|Guatemala.Peten
Carollia sowelli|BCBN500-05|ROM 97490|Mexico.Quintana Roo
Carollia sowelli|BCBN501-05|ROM 97491|Mexico.Quintana Roo
Carollia sowelli|BCBN502-05|ROM 97492|Mexico.Quintana Roo
Carollia sowelli|ABCSA492-06|ROM 99478|Guatemala.Peten
Carollia sowelli|ABCSA493-06|ROM 99479|Guatemala.Peten
Carollia sowelli|ABCSA494-06|ROM 99480|Guatemala.Peten
Carollia sowelli|ABMXC556-06|ROM 97494|Mexico.Quintana Roo
Carollia sowelli|ABMXC557-06|ROM 97495|Mexico.Quintana Roo
Carollia sowelli|ABMXC558-06|ROM 97496|Mexico.Quintana Roo
Carollia sowelli|ABMXC559-06|ROM 97497|Mexico.Quintana Roo
Carollia sowelli|ABMXC560-06|ROM 97501|Mexico.Quintana Roo
Carollia sowelli|ABMXC561-06|ROM 97502|Mexico.Quintana Roo
Carollia sowelli|ABMXC563-06|ROM 97504|Mexico.Quintana Roo
Carollia sowelli|ABMXC564-06|ROM 97505|Mexico.Quintana Roo
Carollia sowelli|ABMXC566-06|ROM 97507|Mexico.Quintana Roo
Carollia sowelli|ABMXC567-06|ROM 97508|Mexico.Quintana Roo
Carollia sowelli|ABCSA445-06|ROM 99590|Guatemala.Peten
Carollia sowelli|ABCSA482-06|ROM 99465|Guatemala.Peten
Carollia sowelli|ABCSA491-06|ROM 99477|Guatemala.Peten
Carollia sowelli|BCBN503-05|ROM 97493|Mexico.Quintana Roo
Carollia sowelli|ABCSA444-06|ROM 99589|Guatemala.Peten
Carollia sowelli|ABCSA441-06|ROM 99586|Guatemala.Peten
Carollia sowelli|ABCSA411-06|ROM 99431|Guatemala.Peten
Carollia sowelli|ABCSA410-06|ROM 99430|Guatemala.Peten
Carollia sowelli|ABCSA352-06|ROM 99335|Guatemala.Peten
Carollia sowelli|ABCSA351-06|ROM 99334|Guatemala.Peten
Carollia sowelli|ABCSA343-06|ROM 99325|Guatemala.Peten
Carollia sowelli|ABCSA299-06|ROM 99275|Guatemala.Peten
Carollia sowelli|ABCSA298-06|ROM 99274|Guatemala.Peten
Carollia sowelli|ABCSA296-06|ROM 99272|Guatemala.Peten
Carollia sowelli|ABCSA295-06|ROM 99271|Guatemala.Peten
Carollia sowelli|ABCSA286-06|ROM 99257|Guatemala.Peten
Carollia sowelli|ABCSA285-06|ROM 99256|Guatemala.Peten
Carollia sowelli|ABCSA293-06|ROM 99269|Guatemala.Peten
Carollia sowelli|ABMXC193-06|ROM 96187|Mexico.Tabasco
Carollia sowelli|BCBNC220-06|ROM 96211|Mexico.Tabasco
Carollia sowelli|ABSCA056-06|ROM 104243|Panama
Carollia sowelli|BCBNT421-06|ROM 108301|Costa Rica.Limon
Carollia sowelli|ABSCA081-06|ROM 104292|Panama.Chiriqui
Carollia sowelli|BCBN668-05|ROM 104290|Panama.Chiriqui
Carollia sowelli|BCBN683-05|ROM 104314|Panama.Chiriqui
Carollia sowelli|ABSCA133-06|ROM F44018|Costa Rica.Limon
Carollia sowelli|BCBN669-05|ROM 104291|Panama.Chiriqui
Carollia sowelli|BCBNT412-06|ROM 108292|Costa Rica.Limon
Carollia sowelli|BCBNT422-06|ROM 108302|Costa Rica.Limon
Carollia brevicauda PS2|BCBNC101-06|ROM 115350|Guyana.Cuyuni-Mazaruni
Carollia brevicauda PS2|BCBNT266-06|ROM 108248|Guyana.Cuyuni-Mazaruni
Carollia brevicauda PS2|BCBNC166-06|ROM 115961|Guyana.Potaro-Siparuni
Carollia brevicauda|ABECA396-06|ROM 105293|Ecuador.Napo
Carollia brevicauda PS2|ABGYE262-06|ROM 111711|Guyana.Potaro-Siparuni
Carollia brevicauda|ABECA919-06|ROM 106099|Ecuador.Napo
Carollia brevicauda|ABGYB083-06|ROM 101060|Guyana.Barima-Waini
Carollia brevicauda|ABGYA771-06|ROM 100821|Guyana.Barima-Waini

Carollia brevicauda|ABECA919-06|ROM 106099|Ecuador.Napo
Carollia brevicauda|ABGYB083-06|ROM 101060|Guyana.Barima-Waini
Carollia brevicauda|ABGYA771-06|ROM 100821|Guyana.Barima-Waini
Carollia brevicauda|ABGYA341-06|ROM 98740|Guyana.Barima-Waini
Carollia brevicauda PS2|ABGYC755-06|ROM 108154|Guyana.Cuyuni-Mazaruni
Carollia brevicauda PS2|BCBNT239-06|ROM 108149|Guyana.Cuyuni-Mazaruni
Carollia brevicauda|ABGYG394-06|ROM 116520|Guyana.Potaro-Siparuni
Carollia brevicauda|ABGYD466-06|ROM 108817|Guyana.Potaro-Siparuni
Carollia brevicauda|ABGYD791-06|ROM 109213|Guyana.Potaro-Siparuni
Carollia brevicauda PS2|BCBNT091-06|ROM 107088|Guyana.Potaro-Siparuni
Carollia brevicauda|ABGYD216-06|ROM 108547|Guyana.Potaro-Siparuni
Carollia brevicauda|ABGYD398-06|ROM 108744|Guyana.Potaro-Siparuni
Carollia brevicauda PS2|BCBNT642-06|ROM 113595|Guyana.Upper Takutu-Upper Essequibo
Carollia perspicillata|ABSA055-06|ROM 111053|Brazil.Sao Paulo
Carollia perspicillata|ABSA060-06|ROM 111066|Brazil.Sao Paulo
Carollia perspicillata|BCBNT467-06|ROM 111067|Brazil.Sao Paulo
Carollia perspicillata|ABSA059-06|ROM 111062|Brazil.Sao Paulo
Carollia perspicillata|ABSA076-06|ROM 111094|Brazil.Sao Paulo
Carollia perspicillata|ABSA097-06|ROM 111132|Brazil.Sao Paulo
Carollia perspicillata|BCBNT456-06|ROM 111050|Brazil.Sao Paulo
Carollia perspicillata|ABSA064-06|ROM 111078|Brazil.Sao Paulo
Carollia perspicillata|BCBNT461-06|ROM 111057|Brazil.Sao Paulo
Carollia perspicillata|ABSA078-06|ROM 111105|Brazil.Sao Paulo
Carollia perspicillata|ABSA070-06|ROM 111088|Brazil.Sao Paulo
Carollia perspicillata|BCBNT468-06|ROM 111068|Brazil.Sao Paulo
Carollia perspicillata|BCBNC135-06|ROM 115804|Guyana.Potaro-Siparuni
Carollia perspicillata|ABGYG755-08|ROM 119183|Guyana
Carollia perspicillata|BCBNT078-06|ROM 107044|Guyana.Potaro-Siparuni
Carollia perspicillata|ABGYC275-06|ROM 107066|Guyana.Potaro-Siparuni
Carollia perspicillata|ABSMS066-06|ROM 117228|Suriname.Sipaliwini
Carollia perspicillata|ABGYD177-06|ROM 108503|Guyana.Potaro-Siparuni
Carollia perspicillata|ABGYB256-06|ROM 102960|Guyana.Upper Takutu-Upper Essequibo
Carollia perspicillata|ABGYB023-06|ROM 100994|Guyana.Barima-Waini
Carollia perspicillata|ABGYA477-06|ROM 98887|Guyana.Barima-Waini
Carollia perspicillata|BCBNT685-06|ROM 113767|Guyana.Demerara-Mahaica
Carollia perspicillata|ABECA517-06|ROM 105550|Ecuador.Napo
Carollia perspicillata|ABECA775-06|ROM 105936|Ecuador.Napo
Carollia perspicillata|ABGYE698-06|ROM 112631|Guyana.Demerara-Mahaica
Carollia perspicillata|ABECA758-06|ROM 105909|Ecuador.Napo
Carollia perspicillata|ABECA910-06|ROM 106092|Ecuador.Napo
Carollia perspicillata|ABGYA569-06|ROM 100248|Guyana.East Berbice-Corentyne
Carollia perspicillata|ABGYA721-06|ROM 100426|Guyana.East Berbice-Corentyne
Carollia perspicillata|ABGYC063-06|ROM 106549|Guyana.Upper Takutu-Upper Essequibo
Carollia perspicillata|ABGYE546-06|ROM 112019|Guyana.Potaro-Siparuni
Carollia perspicillata|ABGYG014-06|ROM 115496|Guyana.Essequibo Islands-West Demerara
Carollia perspicillata|ABGYG754-08|ROM 119182|Guyana
Carollia perspicillata|ABGYG777-08|ROM 119205|Guyana
Carollia perspicillata|BCBN293-05|ROM 100257|Guyana.East Berbice-Corentyne
Carollia perspicillata|BCBN580-05|ROM 104034|Ecuador.Napo
Carollia perspicillata|ABGYE635-06|ROM 112550|Guyana.Demerara-Mahaica
Carollia perspicillata|ABGYB046-06|ROM 101018|Guyana.Barima-Waini
Carollia perspicillata|ABGYE131-06|ROM 111574|Guyana.Potaro-Siparuni
Carollia perspicillata|ABGYG589-08|ROM 119017|Guyana
Carollia perspicillata|BCBNT251-06|ROM 108190|Guyana.Cuyuni-Mazaruni
Carollia perspicillata|ABGYB831-06|ROM 104672|Guyana.Potaro-Siparuni
Carollia perspicillata|ABGYD256-06|ROM 108592|Guyana.Potaro-Siparuni
Carollia perspicillata|ABGYD834-06|ROM 109258|Guyana.Potaro-Siparuni
Carollia perspicillata|ABGYB146-06|ROM 101133|Guyana.Barima-Waini
Carollia perspicillata|ABGYC674-06|ROM 108080|Guyana.Cuyuni-Mazaruni
Carollia perspicillata|ABSMS265-06|ROM 117427|Suriname.Sipaliwini
Carollia perspicillata|ABSA027-06|ROM 107887|Venezuela
Carollia perspicillata|ABGYE614-06|ROM 112093|Guyana.Potaro-Siparuni
Carollia perspicillata|ABGYD178-06|ROM 108504|Guyana.Potaro-Siparuni
Carollia perspicillata|ABGYD390-06|ROM 108736|Guyana.Potaro-Siparuni
Carollia perspicillata|ABGYB253-06|ROM 102957|Guyana.Upper Takutu-Upper Essequibo
Carollia perspicillata|ABGYC197-06|ROM 106736|Guyana.Upper Takutu-Upper Essequibo
Carollia perspicillata|ABSMS257-06|ROM 117419|Suriname.Sipaliwini
Carollia perspicillata|BCBN854-05|ROM 105579|Ecuador.Napo
Carollia perspicillata|ABGYC563-06|ROM 107407|Guyana.Potaro-Siparuni
Carollia perspicillata|ABSMS260-06|ROM 117422|Suriname.Sipaliwini
Carollia perspicillata|ABSRA528-06|ROM 117118|Suriname.Nickerie
Carollia perspicillata|ABGYG151-06|ROM 115711|Guyana.Potaro-Siparuni
Carollia perspicillata|ABGYC205-06|ROM 106744|Guyana.Upper Takutu-Upper Essequibo
Carollia perspicillata|ABGYG408-06|ROM 116536|Guyana.Potaro-Siparuni
Carollia perspicillata|ABSMS032-06|ROM 117194|Suriname.Sipaliwini
Carollia perspicillata|ABGYB298-06|ROM 103002|Guyana.Upper Takutu-Upper Essequibo
Carollia perspicillata|BCBN777-05|ROM 105147|Ecuador.Napo
Carollia perspicillata|BCBN562-05|ROM 103997|Ecuador.Napo
Carollia perspicillata|BCBN144-05|ROM 98098|Guyana.Upper Takutu-Upper Essequibo
Carollia perspicillata|BCBN109-05|ROM 97815|Guyana.Upper Takutu-Upper Essequibo
Carollia perspicillata|ABSRA283-06|ROM 116872|Suriname.Nickerie
Carollia perspicillata|ABSRA269-06|ROM 116858|Suriname.Nickerie
Carollia perspicillata|ABSMS477-06|ROM 117639|Suriname.Sipaliwini
Carollia perspicillata|ABSMS280-06|ROM 117442|Suriname.Sipaliwini
Carollia perspicillata|ABSMS169-06|ROM 117331|Suriname.Sipaliwini
Carollia perspicillata|ABSA035-06|ROM 107928|Venezuela.Bolivar
Carollia perspicillata|ABSA018-06|ROM 107866|Venezuela
Carollia perspicillata|ABGYG989-08|ROM 119417|Guyana
Carollia perspicillata|ABGYG964-08|ROM 119392|Guyana
Carollia perspicillata|ABGYG930-08|ROM 119358|Guyana
Carollia perspicillata|ABGYG775-08|ROM 119203|Guyana
Carollia perspicillata|ABGYG737-08|ROM 119165|Guyana
Carollia perspicillata|ABGYG407-06|ROM 116535|Guyana.Potaro-Siparuni
Carollia perspicillata|ABGYG403-06|ROM 116531|Guyana.Potaro-Siparuni
Carollia perspicillata|ABGYG375-06|ROM 116498|Guyana.Potaro-Siparuni
Carollia perspicillata|ABGYG132-06|ROM 115688|Guyana.Potaro-Siparuni
Carollia perspicillata|ABGYG054-06|ROM 115566|Guyana.Essequibo Islands-West Demerara
Carollia perspicillata|ABGYE644-06|ROM 112559|Guyana.Demerara-Mahaica
Carollia perspicillata|ABGYE604-06|ROM 112083|Guyana.Potaro-Siparuni
Carollia perspicillata|ABGYE585-06|ROM 112062|Guyana.Potaro-Siparuni
Carollia perspicillata|ABGYE402-06|ROM 111854|Guyana.Potaro-Siparuni
Carollia perspicillata|ABGYE073-06|ROM 111516|Guyana.Potaro-Siparuni
Carollia perspicillata|ABGYD658-06|ROM 109067|Guyana.Potaro-Siparuni

[illegible]
Carollia perspicillata|ABGYE402-06|ROM 111854|Guyana.Potaro-Siparuni
Carollia perspicillata|ABGYE073-06|ROM 111516|Guyana.Potaro-Siparuni
Carollia perspicillata|ABGYD658-06|ROM 109067|Guyana.Potaro-Siparuni
Carollia perspicillata|ABGYD394-06|ROM 108740|Guyana.Potaro-Siparuni
Carollia perspicillata|ABGYD255-06|ROM 108591|Guyana.Potaro-Siparuni
Carollia perspicillata|ABGYD196-06|ROM 108525|Guyana.Potaro-Siparuni
Carollia perspicillata|ABGYD033-06|ROM 108255|Guyana.Cuyuni-Mazaruni
Carollia perspicillata|ABGYC790-06|ROM F43225|Guyana.Cuyuni-Mazaruni
Carollia perspicillata|ABGYB922-06|ROM 104767|Guyana.Potaro-Siparuni
Carollia perspicillata|ABGYB880-06|ROM 104724|Guyana.Potaro-Siparuni
Carollia perspicillata|ABGYB866-06|ROM 104710|Guyana.Potaro-Siparuni
Carollia perspicillata|ABGYB857-06|ROM 104701|Guyana.Potaro-Siparuni
Carollia perspicillata|ABGYB785-06|ROM 103553|Guyana.Upper Demerara-Berbice
Carollia perspicillata|ABGYB278-06|ROM 102982|Guyana.Upper Takutu-Upper Essequibo
Carollia perspicillata|ABGYB233-06|ROM 102935|Guyana.Upper Takutu-Upper Essequibo
Carollia perspicillata|ABGYB231-06|ROM 102933|Guyana.Upper Takutu-Upper Essequibo
Carollia perspicillata|ABGYB088-06|ROM 101065|Guyana.Barima-Waini
Carollia perspicillata|ABGYB040-06|ROM 101012|Guyana.Barima-Waini
Carollia perspicillata|ABGYB002-06|ROM 100969|Guyana.Barima-Waini
Carollia perspicillata|ABGYA765-06|ROM 100815|Guyana.Barima-Waini
Carollia perspicillata|ABGYA610-06|ROM 100297|Guyana.East Berbice-Corentyne
Carollia perspicillata|ABGYA546-06|ROM 100218|Guyana.East Berbice-Corentyne
Carollia perspicillata|ABGYA381-06|ROM 98785|Guyana.Barima-Waini
Carollia perspicillata|ABGYA356-06|ROM 98756|Guyana.Barima-Waini
Carollia perspicillata|ABGYA300-06|ROM 98696|Guyana.Barima-Waini
Carollia perspicillata|ABGYA225-06|ROM 98101|Guyana.Upper Takutu-Upper Essequibo
Carollia perspicillata|ABGYA152-06|ROM 97997|Guyana.Potaro-Siparuni
Carollia perspicillata|ABECA091-06|ROM F37200|Ecuador.Napo
Carollia perspicillata|ABECA755-06|ROM 105906|Ecuador.Napo
Carollia perspicillata|ABSMS166-06|ROM 117328|Suriname.Sipaliwini
Carollia perspicillata|ABGYC675-06|ROM F39729|Guyana.Cuyuni-Mazaruni
Carollia perspicillata|ABGYA827-06|ROM 100886|Guyana.Barima-Waini
Carollia perspicillata|ABECA519-06|ROM 105551|Ecuador.Napo
Carollia perspicillata|ABGYC814-06|ROM 108217|Guyana.Cuyuni-Mazaruni
Carollia perspicillata|ABGYG404-06|ROM 116532|Guyana.Potaro-Siparuni
Carollia perspicillata|ABGYE613-06|ROM 112092|Guyana.Potaro-Siparuni
Carollia perspicillata|ABGYA595-06|ROM 100282|Guyana.East Berbice-Corentyne
Carollia perspicillata|ABGYA669-06|ROM 100373|Guyana.East Berbice-Corentyne
Carollia perspicillata|ABECA874-06|ROM 106051|Ecuador.Napo
Carollia perspicillata|ABGYA824-06|ROM 100883|Guyana.Barima-Waini
Carollia perspicillata|ABGYG439-06|ROM 116574|Guyana.Potaro-Siparuni
Carollia perspicillata|ABGYB252-06|ROM 102956|Guyana.Upper Takutu-Upper Essequibo
Carollia perspicillata|ABGYD424-06|ROM 108774|Guyana.Potaro-Siparuni
Carollia perspicillata|ABGYG587-08|ROM 119015|Guyana
Carollia perspicillata|ABSRA296-06|ROM 116885|Suriname.Nickerie
Carollia perspicillata|BCBNC189-06|ROM 116577|Guyana.Potaro-Siparuni
Carollia perspicillata|BCBNT877-06|ROM 115497|Guyana.Essequibo Islands-West Demerara
Carollia perspicillata|ABGYC679-06|ROM 108081|Guyana.Cuyuni-Mazaruni
Carollia perspicillata|ABGYB217-06|ROM 102919|Guyana.Upper Takutu-Upper Essequibo
Carollia perspicillata|ABGYG001-06|ROM 115478|Guyana.Essequibo Islands-West Demerara
Carollia perspicillata|ABGYE630-06|ROM 112543|Guyana.Demerara-Mahaica
Carollia perspicillata|ABGYD128-06|ROM 108431|Guyana.Potaro-Siparuni
Carollia perspicillata|ABGYC551-06|ROM 107395|Guyana.Potaro-Siparuni
Carollia perspicillata|ABGYC332-06|ROM 107144|Guyana.Potaro-Siparuni
Carollia perspicillata|ABGYG131-06|ROM 115687|Guyana.Potaro-Siparuni
Carollia perspicillata|ABGYG055-06|ROM 115567|Guyana.Essequibo Islands-West Demerara
Carollia perspicillata|ABGYG031-06|ROM 115537|Guyana.Essequibo Islands-West Demerara
Carollia perspicillata|ABGYC526-06|ROM 107364|Guyana.Potaro-Siparuni
Carollia perspicillata|ABGYC438-06|ROM 107262|Guyana.Potaro-Siparuni
Carollia perspicillata|ABGYC328-06|ROM 107140|Guyana.Potaro-Siparuni
Carollia perspicillata|ABSMS255-06|ROM 117417|Suriname.Sipaliwini
Carollia perspicillata|ABGYG757-08|ROM 119185|Guyana
Carollia perspicillata|BCBNC106-06|ROM 115741|Guyana.Potaro-Siparuni
Carollia perspicillata|ABGYA545-06|ROM 100217|Guyana.East Berbice-Corentyne
Carollia perspicillata|ABGYE248-06|ROM 111697|Guyana.Potaro-Siparuni
Carollia perspicillata|ABSMS058-06|ROM 117220|Suriname.Sipaliwini
Carollia perspicillata|ABSRA450-06|ROM 117040|Suriname.Nickerie
Carollia perspicillata|ABGYB280-06|ROM 102984|Guyana.Upper Takutu-Upper Essequibo
Carollia perspicillata|ABGYG059-06|ROM 115574|Guyana.Essequibo Islands-West Demerara
Carollia perspicillata|ABGYD738-06|ROM 109155|Guyana.Potaro-Siparuni
Carollia perspicillata|ABGYD790-06|ROM 109212|Guyana.Potaro-Siparuni
Carollia perspicillata|ABSMS431-06|ROM 117593|Suriname.Sipaliwini
Carollia perspicillata|BCBNT912-06|ROM 115577|Guyana.Essequibo Islands-West Demerara
Carollia perspicillata|ABGYB017-06|ROM 100988|Guyana.Barima-Waini
Carollia perspicillata|ABGYD062-06|ROM 108358|Guyana.Potaro-Siparuni
Carollia perspicillata|ABGYE634-06|ROM 112549|Guyana.Demerara-Mahaica
Carollia perspicillata|ABGYC289-06|ROM 107087|Guyana.Potaro-Siparuni
Carollia perspicillata|ABGYC477-06|ROM 107310|Guyana.Potaro-Siparuni
Carollia perspicillata|ABGYD613-06|ROM 108983|Guyana.Potaro-Siparuni
Carollia perspicillata|ABGYE039-06|ROM 109325|Guyana.Potaro-Siparuni
Carollia perspicillata|ABGYA480-06|ROM 98890|Guyana.Barima-Waini
Carollia perspicillata|ABGYA385-06|ROM 98789|Guyana.Barima-Waini
Carollia perspicillata|ABGYC682-06|ROM 108084|Guyana.Cuyuni-Mazaruni
Carollia perspicillata|ABGYG815-08|ROM 119243|Guyana
Carollia perspicillata|ABECA145-06|ROM 104446|Ecuador.Napo
Carollia perspicillata|ABSRA282-06|ROM 116871|Suriname.Nickerie
Carollia perspicillata|ABGYC518-06|ROM 107356|Guyana.Potaro-Siparuni
Carollia perspicillata|ABSRA449-06|ROM 117039|Suriname.Nickerie
Carollia perspicillata|ABGYC209-06|ROM 106749|Guyana.Upper Takutu-Upper Essequibo
Carollia perspicillata|ABGYE109-06|ROM 111552|Guyana.Potaro-Siparuni
Carollia perspicillata|ABSA093-06|ROM 111125|Brazil.Sao Paulo
Carollia perspicillata|ABSRA297-06|ROM 116886|Suriname.Nickerie
Carollia perspicillata|BCBN105-05|ROM 97795|Guyana.Upper Takutu-Upper Essequibo
Carollia perspicillata|ABGYE629-06|ROM 112542|Guyana.Demerara-Mahaica
Carollia perspicillata|ABGYA083-06|ROM 97906|Guyana.Upper Takutu-Upper Essequibo
Carollia perspicillata|ABGYE796-06|ROM 113397|Guyana.Upper Demerara-Berbice
Carollia perspicillata|ABGYE823-06|ROM 113429|Guyana.Upper Demerara-Berbice
Carollia perspicillata|ABGYD362-06|ROM 108705|Guyana.Potaro-Siparuni
Carollia perspicillata|ABGYE639-06|ROM 112554|Guyana.Demerara-Mahaica
Carollia perspicillata|ABGYA719-06|ROM 100424|Guyana.East Berbice-Corentyne
Carollia perspicillata|ABGYD102-06|ROM 108400|Guyana.Potaro-Siparuni
Carollia perspicillata|BCBNC182-06|ROM 116538|Guyana.Potaro-Siparuni
[illegible]

Carollia perspicillata|ABGYA719-06|ROM 100424|Guyana.East Berbice-Corentyne
Carollia perspicillata|ABGYD102-06|ROM 108400|Guyana.Potaro-Siparuni
Carollia perspicillata|BCBNC182-06|ROM 116538|Guyana.Potaro-Siparuni
Carollia perspicillata|ABGYA753-06|ROM 100459|Guyana.East Berbice-Corentyne
Carollia perspicillata|BCBNT077-06|ROM 107033|Guyana.Potaro-Siparuni
Carollia perspicillata|ABGYC649-06|ROM 107467|Guyana.Potaro-Siparuni
Carollia perspicillata|ABSMS022-06|ROM 117184|Suriname.Sipaliwini
Carollia perspicillata|ABGYA555-06|ROM 100230|Guyana.East Berbice-Corentyne
Carollia perspicillata|ABSMS135-06|ROM 117297|Suriname.Sipaliwini
Carollia perspicillata|ABGYG779-08|ROM 119207|Guyana
Carollia perspicillata|ABGYG042-06|ROM 115552|Guyana.Essequibo Islands-West Demerara
Carollia perspicillata|ABGYA553-06|ROM 100226|Guyana.East Berbice-Corentyne
Carollia perspicillata|ABGYB044-06|ROM 101016|Guyana.Barima-Waini
Carollia perspicillata|ABSMS476-06|ROM 117638|Suriname.Sipaliwini
Carollia perspicillata|ABGYA808-06|ROM 100867|Guyana.Barima-Waini
Carollia perspicillata|ABGYB859-06|ROM 104703|Guyana.Potaro-Siparuni
Carollia perspicillata|ABGYG154-06|ROM 115714|Guyana.Potaro-Siparuni
Carollia perspicillata|ABSA068-06|ROM 111086|Brazil.Sao Paulo
Carollia perspicillata|BCBN153-05|ROM 98182|Guyana.Demerara-Mahaica
Carollia perspicillata|ABGYA524-06|ROM 100191|Guyana.East Berbice-Corentyne
Carollia perspicillata|ABGYE407-06|ROM 111860|Guyana.Potaro-Siparuni
Carollia perspicillata|BCBNT223-06|ROM 107938|Venezuela.Bolivar
Carollia perspicillata|ABGYE408-06|ROM 111861|Guyana.Potaro-Siparuni
Carollia perspicillata|ABGYE761-06|ROM 113360|Guyana.Upper Demerara-Berbice
Carollia perspicillata|ABECA032-06|ROM 104013|Ecuador.Napo
Carollia perspicillata|ABGYA445-06|ROM 98854|Guyana.Barima-Waini
Carollia perspicillata|ABGYC791-06|ROM F43226|Guyana.Cuyuni-Mazaruni
Carollia perspicillata|ABGYG409-06|ROM 116537|Guyana.Potaro-Siparuni
Carollia perspicillata|ABGYA568-06|ROM 100247|Guyana.East Berbice-Corentyne
Carollia perspicillata|ABSRA285-06|ROM 116874|Suriname.Nickerie
Carollia perspicillata|ABGYB092-06|ROM 101069|Guyana.Barima-Waini
Carollia perspicillata|ABSA083-06|ROM 111115|Brazil.Sao Paulo
Carollia perspicillata|ABSA077-06|ROM 111096|Brazil.Sao Paulo
Carollia perspicillata|ABSA095-06|ROM 111127|Brazil.Sao Paulo
Carollia perspicillata|ABGYA169-06|ROM 98028|Guyana.Potaro-Siparuni
Carollia perspicillata|ABGYA171-06|ROM 98030|Guyana.Potaro-Siparuni
Carollia perspicillata|ABGYA364-06|ROM 98765|Guyana.Barima-Waini
Carollia perspicillata|ABGYA596-06|ROM 100283|Guyana.East Berbice-Corentyne
Carollia perspicillata|ABGYA781-06|ROM 100831|Guyana.Barima-Waini
Carollia perspicillata|ABGYA849-06|ROM 100915|Guyana.Barima-Waini
Carollia perspicillata|ABGYB145-06|ROM 101132|Guyana.Barima-Waini
Carollia perspicillata|ABGYC347-06|ROM 107159|Guyana.Potaro-Siparuni
Carollia perspicillata|ABGYC722-06|ROM 108122|Guyana.Cuyuni-Mazaruni
Carollia perspicillata|ABGYF101-06|ROM 113640|Guyana.Demerara-Mahaica
Carollia perspicillata|ABGYG1081-08|ROM 119005|Guyana
Carollia perspicillata|BCBNC202-06|ROM 116705|Guyana.Potaro-Siparuni
Carollia perspicillata|ABGYE840-06|ROM 113453|Guyana.Upper Takutu-Upper Essequibo
Carollia perspicillata|ABGYC746-06|ROM F43169|Guyana.Cuyuni-Mazaruni
Carollia perspicillata|ABGYB119-06|ROM 101100|Guyana.Barima-Waini
Carollia perspicillata|ABGYB120-06|ROM 101101|Guyana.Barima-Waini
Carollia perspicillata|ABGYD199-06|ROM 108528|Guyana.Potaro-Siparuni
Carollia perspicillata|BCBN145-05|ROM 98099|Guyana.Upper Takutu-Upper Essequibo
Carollia perspicillata|BCBNT662-06|ROM 113662|Guyana.Demerara-Mahaica
Carollia perspicillata|ABGYA529-06|ROM 100196|Guyana.East Berbice-Corentyne
Carollia perspicillata|ABGYD435-06|ROM 108786|Guyana.Potaro-Siparuni
Carollia perspicillata|BCBNT906-06|ROM 115564|Guyana.Essequibo Islands-West Demerara
Carollia perspicillata|BCBN142-05|ROM 98096|Guyana.Upper Takutu-Upper Essequibo
Carollia perspicillata|ABGYC038-06|ROM 104815|Guyana.Potaro-Siparuni
Carollia perspicillata|ABGYC648-06|ROM 107466|Guyana.Potaro-Siparuni
Carollia perspicillata|ABGYE121-06|ROM 111564|Guyana.Potaro-Siparuni
Carollia perspicillata|BCBN126-05|ROM 97883|Guyana.Upper Takutu-Upper Essequibo
Carollia perspicillata|BCBN327-05|ROM 100866|Guyana.Barima-Waini
Carollia perspicillata|ABGYD104-06|ROM 108402|Guyana.Potaro-Siparuni
Carollia perspicillata|ABGYA244-06|ROM 98133|Guyana.Upper Demerara-Berbice
Carollia perspicillata|ABGYA250-06|ROM 98139|Guyana.Upper Demerara-Berbice
Carollia perspicillata|ABGYA520-06|ROM 100187|Guyana.East Berbice-Corentyne
Carollia perspicillata|ABGYE449-06|ROM 111912|Guyana.Potaro-Siparuni
Carollia perspicillata|ABGYE691-06|ROM 112621|Guyana.Demerara-Mahaica
Carollia perspicillata|ABSMS409-06|ROM 117571|Suriname.Sipaliwini
Carollia perspicillata|ABGYA375-06|ROM 98779|Guyana.Barima-Waini
Carollia perspicillata|ABGYB215-06|ROM 102917|Guyana.Upper Takutu-Upper Essequibo
Carollia perspicillata|ABSA056-06|ROM 111054|Brazil.Sao Paulo
Carollia perspicillata|ABSA082-06|ROM 111113|Brazil.Sao Paulo
Carollia perspicillata|ABGYC478-06|ROM 107311|Guyana.Potaro-Siparuni
Carollia perspicillata|BCBN150-05|ROM 98179|Guyana.Demerara-Mahaica
Carollia perspicillata|ABGYB507-06|ROM 103242|Guyana.Upper Takutu-Upper Essequibo
Carollia perspicillata|ABGYA789-06|ROM 100842|Guyana.Barima-Waini
Carollia perspicillata|ABGYA366-06|ROM 98767|Guyana.Barima-Waini
Carollia perspicillata|ABGYA778-06|ROM 100828|Guyana.Barima-Waini
Carollia perspicillata|ABGYE638-06|ROM 112553|Guyana.Demerara-Mahaica
Carollia perspicillata|ABSMS259-06|ROM 117421|Suriname.Sipaliwini
Carollia perspicillata|ABGYB036-06|ROM 101008|Guyana.Barima-Waini
Carollia perspicillata|ABGYB147-06|ROM 101134|Guyana.Barima-Waini
Carollia perspicillata|ABGYB208-06|ROM 102910|Guyana.Upper Takutu-Upper Essequibo
Carollia perspicillata|ABGYB279-06|ROM 102983|Guyana.Upper Takutu-Upper Essequibo
Carollia perspicillata|ABGYB708-06|ROM 103472|Guyana.Upper Demerara-Berbice
Carollia perspicillata|ABGYB732-06|ROM 103499|Guyana.Upper Demerara-Berbice
Carollia perspicillata|ABGYB858-06|ROM 104702|Guyana.Potaro-Siparuni
Carollia perspicillata|ABGYB900-06|ROM 104745|Guyana.Potaro-Siparuni
Carollia perspicillata|ABGYB903-06|ROM 104748|Guyana.Potaro-Siparuni
Carollia perspicillata|ABGYC006-06|ROM 104783|Guyana.Potaro-Siparuni
Carollia perspicillata|ABGYC019-06|ROM 104796|Guyana.Potaro-Siparuni
Carollia perspicillata|ABGYC037-06|ROM 104814|Guyana.Potaro-Siparuni
Carollia perspicillata|ABGYC327-06|ROM 107139|Guyana.Potaro-Siparuni
Carollia perspicillata|ABGYC455-06|ROM 107280|Guyana.Potaro-Siparuni
Carollia perspicillata|ABGYC475-06|ROM 107308|Guyana.Potaro-Siparuni
Carollia perspicillata|ABGYC672-06|ROM 108078|Guyana.Cuyuni-Mazaruni
Carollia perspicillata|ABGYC683-06|ROM 108085|Guyana.Cuyuni-Mazaruni
Carollia perspicillata|ABGYD074-06|ROM 108371|Guyana.Potaro-Siparuni
Carollia perspicillata|ABGYD197-06|ROM 108526|Guyana.Potaro-Siparuni
Carollia perspicillata|ABGYD325-06|ROM 108665|Guyana.Potaro-Siparuni
Carollia perspicillata|ABGYE336-06|ROM 111786|Guyana.Potaro-Siparuni
Carollia perspicillata|ABGYE403-06|ROM 111855|Guyana.Potaro-Siparuni
[illegible]

Carollia perspicillata|ABGYD325-06|ROM 108665|Guyana.Potaro-Siparuni
Carollia perspicillata|ABGYE336-06|ROM 111786|Guyana.Potaro-Siparuni
Carollia perspicillata|ABGYE403-06|ROM 111855|Guyana.Potaro-Siparuni
Carollia perspicillata|ABGYE448-06|ROM 111911|Guyana.Potaro-Siparuni
Carollia perspicillata|ABGYE481-06|ROM 111953|Guyana.Potaro-Siparuni
Carollia perspicillata|ABGYE487-06|ROM 111959|Guyana.Potaro-Siparuni
Carollia perspicillata|ABGYE849-06|ROM 113464|Guyana.Upper Takutu-Upper Essequibo
Carollia perspicillata|ABGYF097-06|ROM 113631|Guyana.Demerara-Mahaica
Carollia perspicillata|ABGYF098-06|ROM 113632|Guyana.Demerara-Mahaica
Carollia perspicillata|ABGYG016-06|ROM 115501|Guyana.Essequibo Islands-West Demerara
Carollia perspicillata|ABGYG590-08|ROM 119018|Guyana
Carollia perspicillata|ABGYG642-08|ROM 119070|Guyana
Carollia perspicillata|ABGYG679-08|ROM 119107|Guyana
Carollia perspicillata|ABGYG692-08|ROM 119120|Guyana
Carollia perspicillata|ABGYG878-08|ROM 119306|Guyana
Carollia perspicillata|ABGYG991-08|ROM 119419|Guyana
Carollia perspicillata|ABSMS144-06|ROM 117306|Suriname.Sipaliwini
Carollia perspicillata|ABSMS216-06|ROM 117378|Suriname.Sipaliwini
Carollia perspicillata|ABSMS444-06|ROM 117606|Suriname.Sipaliwini
Carollia perspicillata|ABGYC148-06|ROM 106664|Guyana.Upper Takutu-Upper Essequibo
Carollia perspicillata|ABGYC273-06|ROM 107062|Guyana.Potaro-Siparuni
Carollia perspicillata|ABSRA294-06|ROM 116883|Suriname.Nickerie
Carollia perspicillata|ABSRA527-06|ROM 117117|Suriname.Nickerie
Carollia perspicillata|ABGYA666-06|ROM 100370|Guyana.East Berbice-Corentyne
Carollia perspicillata|ABGYA848-06|ROM 100914|Guyana.Barima-Waini
Carollia perspicillata|ABGYB005-06|ROM 100972|Guyana.Barima-Waini
Carollia perspicillata|ABGYB034-06|ROM 101006|Guyana.Barima-Waini
Carollia perspicillata|BCBN289-05|ROM 100245|Guyana.East Berbice-Corentyne
Carollia perspicillata|BCBNC020-06|ROM 114728|Guyana.Potaro-Siparuni
Carollia perspicillata|BCBNT075-06|ROM 107031|Guyana.Potaro-Siparuni
Carollia perspicillata|BCBNT204-06|ROM 107910|Venezuela.Bolivar
Carollia perspicillata|BCBNT652-06|ROM 113633|Guyana.Demerara-Mahaica
Carollia perspicillata|BCBNT753-06|ROM 113945|Suriname.Brokopondo
Carollia perspicillata|ABGYA665-06|ROM 100369|Guyana.East Berbice-Corentyne
Carollia perspicillata|ABGYA662-06|ROM 100361|Guyana.East Berbice-Corentyne
Carollia perspicillata|ABGYA421-06|ROM 98829|Guyana.Barima-Waini
Carollia perspicillata|ABGYA228-06|ROM 98104|Guyana.Upper Takutu-Upper Essequibo
Carollia perspicillata|ABGYA133-06|ROM 97975|Guyana.Potaro-Siparuni
Carollia perspicillata|ABGYA132-06|ROM 97974|Guyana.Potaro-Siparuni
Carollia perspicillata|ABGYA014-06|ROM 97796|Guyana.Upper Takutu-Upper Essequibo
Carollia perspicillata|ABECA299-06|ROM 105170|Ecuador.Napo
Carollia perspicillata|ABECA289-06|ROM 105160|Ecuador.Napo
Carollia perspicillata|ABGYA168-06|ROM 98027|Guyana.Potaro-Siparuni
Carollia perspicillata|ABGYG122-06|ROM 115676|Guyana.Potaro-Siparuni
Carollia perspicillata|ABSA006-06|ROM 107850|Venezuela
Carollia perspicillata|ABGYA003-06|ROM 97771|Guyana.Upper Takutu-Upper Essequibo
Carollia perspicillata|ABGYE786-06|ROM 113386|Guyana.Upper Demerara-Berbice
Carollia perspicillata|ABGYB901-06|ROM 104746|Guyana.Potaro-Siparuni
Carollia perspicillata|ABGYG674-08|ROM 119102|Guyana
Carollia perspicillata|ABGYB554-06|ROM 103289|Guyana.Upper Takutu-Upper Essequibo
Carollia perspicillata|ABGYE642-06|ROM 112557|Guyana.Demerara-Mahaica
Carollia perspicillata|ABSMS382-06|ROM 117544|Suriname.Sipaliwini
Carollia perspicillata|ABGYA779-06|ROM 100829|Guyana.Barima-Waini
Carollia perspicillata|ABGYB207-06|ROM 102909|Guyana.Upper Takutu-Upper Essequibo
Carollia perspicillata|ABGYF094-06|ROM 113628|Guyana.Demerara-Mahaica
Carollia perspicillata|ABGYD179-06|ROM 108505|Guyana.Potaro-Siparuni
Carollia perspicillata|ABGYE624-06|ROM 112537|Guyana.Demerara-Mahaica
Carollia perspicillata|ABGYC237-06|ROM 106787|Guyana.Upper Takutu-Upper Essequibo
Carollia perspicillata|ABGYA606-06|ROM 100293|Guyana.East Berbice-Corentyne
Carollia perspicillata|ABGYA527-06|ROM 100194|Guyana.East Berbice-Corentyne
Carollia perspicillata|ABGYA128-06|ROM 97970|Guyana.Upper Takutu-Upper Essequibo
Carollia perspicillata|ABGYA743-06|ROM 100449|Guyana.East Berbice-Corentyne
Carollia perspicillata|ABGYB206-06|ROM 102908|Guyana.Upper Takutu-Upper Essequibo
Carollia perspicillata|ABGYC680-06|ROM 108082|Guyana.Cuyuni-Mazaruni
Carollia perspicillata|ABGYC744-06|ROM 108146|Guyana.Cuyuni-Mazaruni
Carollia perspicillata|ABGYC789-06|ROM 108191|Guyana.Cuyuni-Mazaruni
Carollia perspicillata|ABGYC274-06|ROM 107063|Guyana.Potaro-Siparuni
Carollia perspicillata|ABGYD833-06|ROM 109257|Guyana.Potaro-Siparuni
Carollia perspicillata|ABGYA780-06|ROM 100830|Guyana.Barima-Waini
Carollia perspicillata|ABGYA367-06|ROM 98768|Guyana.Barima-Waini
Carollia perspicillata|ABGYA829-06|ROM 100888|Guyana.Barima-Waini
Carollia perspicillata|ABSMS009-06|ROM 117171|Suriname.Sipaliwini
Carollia perspicillata|BCBNT076-06|ROM 107032|Guyana.Potaro-Siparuni
Carollia perspicillata|BCBNT878-06|ROM 115499|Guyana.Essequibo Islands-West Demerara
Carollia perspicillata|BCBNT915-06|ROM 115586|Guyana.Essequibo Islands-West Demerara
Carollia perspicillata|ABGYB525-06|ROM 103260|Guyana.Upper Takutu-Upper Essequibo
Carollia perspicillata|ABGYA828-06|ROM 100887|Guyana.Barima-Waini
Carollia perspicillata|ABGYE865-06|ROM 113481|Guyana.Upper Takutu-Upper Essequibo
Carollia perspicillata|BCBN933-05|ROM 105810|Ecuador.Esmeraldas
Carollia perspicillata|ABECA410-06|ROM 105310|Ecuador.Napo
Carollia perspicillata|ABSRA448-06|ROM 117038|Suriname.Nickerie
Carollia perspicillata|ABECA098-06|ROM 104385|Ecuador.Napo
Carollia perspicillata|ABSA038-06|ROM 107931|Venezuela.Bolivar
Carollia perspicillata|ABGYB421-06|ROM 103129|Guyana.Upper Takutu-Upper Essequibo
Carollia perspicillata|ABGYA249-06|ROM 98138|Guyana.Upper Demerara-Berbice
Carollia perspicillata|ABGYB067-06|ROM 101043|Guyana.Barima-Waini
Carollia perspicillata|ABGYE480-06|ROM 111952|Guyana.Potaro-Siparuni
Carollia perspicillata|ABGYG440-06|ROM 116575|Guyana.Potaro-Siparuni
Carollia perspicillata|ABGYG494-06|ROM 116635|Guyana.Potaro-Siparuni
Carollia perspicillata|ABGYD696-06|ROM 109109|Guyana.Potaro-Siparuni
Carollia perspicillata|BCBN695-05|ROM 104338|Panama.Darien
Carollia perspicillata|ABECA550-06|ROM 105580|Ecuador.Napo
Carollia perspicillata|ABGYE489-06|ROM 111961|Guyana.Potaro-Siparuni
Carollia perspicillata|ABGYA769-06|ROM 100819|Guyana.Barima-Waini
Carollia perspicillata|ABGYA777-06|ROM 100827|Guyana.Barima-Waini
Carollia perspicillata|ABGYG911-08|ROM 119339|Guyana
Carollia perspicillata|ABGYG1040-08|ROM 118964|Guyana
Carollia perspicillata|BCBN932-05|ROM 105809|Ecuador.Esmeraldas
Carollia perspicillata|ABSRA288-06|ROM 116877|Suriname.Nickerie
Carollia perspicillata|ABSRA291-06|ROM 116880|Suriname.Nickerie
Carollia perspicillata|BCBNT632-06|ROM 113574|Guyana.Upper Takutu-Upper Essequibo
Carollia perspicillata|ABGYF106-06|ROM 113645|Guyana.Demerara-Mahaica
Carollia perspicillata|ABSA029-06|ROM 107896|Venezuela

Carollia perspicillata|BCBNT632-06|ROM 113574|Guyana.Upper Takutu-Upper Essequibo
Carollia perspicillata|ABGYF106-06|ROM 113645|Guyana.Demerara-Mahaica
Carollia perspicillata|ABSA029-06|ROM 107896|Venezuela
Carollia perspicillata|ABGYE626-06|ROM 112539|Guyana.Demerara-Mahaica
Carollia perspicillata|ABGYE829-06|ROM 113440|Guyana.Upper Demerara-Berbice
Carollia perspicillata|BCBNT704-06|ROM 113873|Suriname.Brokopondo
Carollia perspicillata|ABGYD463-06|ROM 108814|Guyana.Potaro-Siparuni
Carollia perspicillata|ABGYD335-06|ROM 108675|Guyana.Potaro-Siparuni
Carollia perspicillata|ABGYD324-06|ROM 108664|Guyana.Potaro-Siparuni
Carollia perspicillata|ABGYD174-06|ROM 108495|Guyana.Potaro-Siparuni
Carollia perspicillata|ABGYC156-06|ROM 106678|Guyana.Upper Takutu-Upper Essequibo
Carollia perspicillata|ABGYB666-06|ROM 103428|Guyana.Upper Demerara-Berbice
Carollia perspicillata|ABGYB108-06|ROM 101086|Guyana.Barima-Waini
Carollia perspicillata|ABGYA129-06|ROM 97971|Guyana.Upper Takutu-Upper Essequibo
Carollia perspicillata|ABECA679-06|ROM F37995|Ecuador.Napo
Carollia perspicillata|ABECA196-06|ROM 104517|Ecuador.Napo
Carollia perspicillata|ABGYB022-06|ROM 100993|Guyana.Barima-Waini
Carollia perspicillata|ABSCA031-06|ROM 104197|Panama.Canal Zone
Carollia perspicillata|BCBN538-05|ROM 103357|Guyana.Upper Demerara-Berbice
Carollia perspicillata|BCBNT880-06|ROM 115502|Guyana.Essequibo Islands-West Demerara
Carollia perspicillata|ABECA101-06|ROM 104390|Ecuador.Napo
Carollia perspicillata|ABGYA313-06|ROM 98709|Guyana.Barima-Waini
Carollia perspicillata|ABGYB879-06|ROM 104723|Guyana.Potaro-Siparuni
Carollia perspicillata|ABGYC269-06|ROM 107058|Guyana.Potaro-Siparuni
Carollia perspicillata|ABGYG875-08|ROM 119303|Guyana
Carollia perspicillata|ABGYG990-08|ROM 119418|Guyana
Carollia perspicillata|ABGYC681-06|ROM 108083|Guyana.Cuyuni-Mazaruni
Carollia perspicillata|ABGYA519-06|ROM 100186|Guyana.East Berbice-Corentyne
Carollia perspicillata|ABGYA772-06|ROM 100822|Guyana.Barima-Waini
Carollia perspicillata|ABGYB090-06|ROM 101067|Guyana.Barima-Waini
Carollia perspicillata|ABGYB098-06|ROM 101075|Guyana.Barima-Waini
Carollia perspicillata|BCBN325-05|ROM 100856|Guyana.Barima-Waini
Carollia perspicillata|ABGYC686-06|ROM 108088|Guyana.Cuyuni-Mazaruni
Carollia perspicillata|ABGYA335-06|ROM 98734|Guyana.Barima-Waini
Carollia perspicillata|ABGYA668-06|ROM 100372|Guyana.East Berbice-Corentyne
Carollia perspicillata|BCBN154-05|ROM 98183|Guyana.Demerara-Mahaica
Carollia perspicillata|BCBNT654-06|ROM 113635|Guyana.Demerara-Mahaica
Carollia perspicillata|BCBN129-05|ROM 97886|Guyana.Upper Takutu-Upper Essequibo
Carollia perspicillata|ABGYF100-06|ROM 113639|Guyana.Demerara-Mahaica
Carollia perspicillata|ABGYB016-06|ROM 100987|Guyana.Barima-Waini
Carollia perspicillata|ABGYA809-06|ROM 100868|Guyana.Barima-Waini
Carollia perspicillata|ABGYB745-06|ROM 103512|Guyana.Upper Demerara-Berbice
Carollia perspicillata|ABGYF105-06|ROM 113644|Guyana.Demerara-Mahaica
Carollia perspicillata|BCBN550-05|ROM 103427|Guyana.Upper Demerara-Berbice
Carollia perspicillata|ABGYA136-06|ROM 97980|Guyana.Potaro-Siparuni
Carollia perspicillata|ABGYC305-06|ROM 107116|Guyana.Potaro-Siparuni
Carollia perspicillata|ABGYD396-06|ROM 108742|Guyana.Potaro-Siparuni
Carollia perspicillata|ABGYB819-06|ROM 104660|Guyana.Potaro-Siparuni
Carollia perspicillata|ABGYA799-06|ROM 100852|Guyana.Barima-Waini
Carollia perspicillata|ABGYA153-06|ROM 97998|Guyana.Potaro-Siparuni
Carollia perspicillata|ABGYA265-06|ROM 98165|Guyana.Potaro-Siparuni
Carollia perspicillata|ABGYG041-06|ROM 115551|Guyana.Essequibo Islands-West Demerara
Carollia perspicillata|BCBN146-05|ROM 98100|Guyana.Upper Takutu-Upper Essequibo
Carollia perspicillata|BCBN935-05|ROM 105812|Ecuador.Esmeraldas
Carollia perspicillata|BCBN689-05|ROM 104329|Panama.Chiriqui
Carollia perspicillata|BCBNT440-06|ROM 108318|Costa Rica.Limon
Carollia perspicillata|ABGYE643-06|ROM 112558|Guyana.Demerara-Mahaica
Carollia perspicillata|ABGYB113-06|ROM 101091|Guyana.Barima-Waini
Carollia perspicillata|ABGYB057-06|ROM 101031|Guyana.Barima-Waini
Carollia perspicillata|ABGYB667-06|ROM 103430|Guyana.Upper Demerara-Berbice
Carollia perspicillata|ABGYG818-08|ROM 119246|Guyana
Carollia perspicillata|BCBN101-05|ROM 97791|Guyana.Upper Takutu-Upper Essequibo
Carollia perspicillata|ABGYA084-06|ROM 97907|Guyana.Upper Takutu-Upper Essequibo
Carollia perspicillata|ABGYB701-06|ROM 103465|Guyana.Upper Demerara-Berbice
Carollia perspicillata|ABGYA384-06|ROM 98788|Guyana.Barima-Waini
Carollia perspicillata|ABGYA798-06|ROM 100851|Guyana.Barima-Waini
Carollia perspicillata|BCBN547-05|ROM 103419|Guyana.Upper Demerara-Berbice
Carollia perspicillata|BCBN506-05|ROM 97500|Mexico.Quintana Roo
Carollia perspicillata|BCBN505-05|ROM 97499|Mexico.Quintana Roo
Carollia perspicillata|BCBN507-05|ROM 97517|Mexico.Quintana Roo
Carollia perspicillata|BCBN522-05|ROM 97725|Mexico.Campeche
Carollia perspicillata|ABMXA946-06|ROM MDE6004|Mexico.Quintana Roo
Carollia perspicillata|ABGYB091-06|ROM 101068|Guyana.Barima-Waini
Carollia perspicillata|ABGYB218-06|ROM 102920|Guyana.Upper Takutu-Upper Essequibo
Carollia perspicillata|ABGYA134-06|ROM 97976|Guyana.Potaro-Siparuni
Carollia perspicillata|ABSMS223-06|ROM 117385|Suriname.Sipaliwini
Carollia perspicillata|ABGYB112-06|ROM 101090|Guyana.Barima-Waini
Carollia perspicillata|ABGYC671-06|ROM 108077|Guyana.Cuyuni-Mazaruni
Carollia perspicillata|ABSA032-06|ROM 107899|Venezuela
Carollia perspicillata|ABSA039-06|ROM 107932|Venezuela.Bolivar
Carollia perspicillata|ABGYG1008-08|ROM 118932|Guyana
Carollia perspicillata|ABGYE830-06|ROM 113441|Guyana.Upper Demerara-Berbice
Carollia perspicillata|ABGYC688-06|ROM F39742|Guyana.Cuyuni-Mazaruni
Carollia perspicillata|ABGYB923-06|ROM 104768|Guyana.Potaro-Siparuni
Carollia perspicillata|ABGYA475-06|ROM 98885|Guyana.Barima-Waini
Carollia perspicillata|ABGYA796-06|ROM 100849|Guyana.Barima-Waini
Carollia perspicillata|ABGYA365-06|ROM 98766|Guyana.Barima-Waini
Carollia perspicillata|ABGYC270-06|ROM 107059|Guyana.Potaro-Siparuni
Carollia perspicillata|ABGYD026-06|ROM 108247|Guyana.Cuyuni-Mazaruni
Carollia perspicillata|ABGYG455-06|ROM 116592|Guyana.Potaro-Siparuni
Carollia perspicillata|ABSMS331-06|ROM 117493|Suriname.Sipaliwini
Carollia perspicillata|BCBN104-05|ROM 97794|Guyana.Upper Takutu-Upper Essequibo
Carollia perspicillata|ABGYA033-06|ROM 97823|Guyana.Upper Takutu-Upper Essequibo
Carollia perspicillata|ABGYE263-06|ROM 111712|Guyana.Potaro-Siparuni
Carollia perspicillata|ABGYE628-06|ROM 112541|Guyana.Demerara-Mahaica
Carollia perspicillata|ABGYF104-06|ROM 113643|Guyana.Demerara-Mahaica
Carollia perspicillata|ABGYD505-06|ROM 108863|Guyana.Potaro-Siparuni
Carollia perspicillata|ABGYD703-06|ROM 109117|Guyana.Potaro-Siparuni
Carollia perspicillata|ABGYA279-06|ROM 98184|Guyana.Demerara-Mahaica
Carollia perspicillata|ABGYB214-06|ROM 102916|Guyana.Upper Takutu-Upper Essequibo
Carollia perspicillata|ABGYG673-08|ROM 119101|Guyana
Carollia perspicillata|ABGYG156-06|ROM 115716|Guyana.Potaro-Siparuni
Carollia perspicillata|BCBN102-05|ROM 97792|Guyana.Upper Takutu-Upper Essequibo

Carollia perspicillata|ABGYB2[illegible]-06|ROM 1029[illegible]|Guyana.Upper Takutu-Upper Essequibo
Carollia perspicillata|ABGYG673-08|ROM 119101|Guyana
Carollia perspicillata|ABGYG156-06|ROM 115716|Guyana.Potaro-Siparuni
Carollia perspicillata|BCBN102-05|ROM 97792|Guyana.Upper Takutu-Upper Essequibo
Carollia perspicillata|ABGYG931-08|ROM 119359|Guyana
Carollia perspicillata|ABGYF007-06|ROM 113493|Guyana.Upper Takutu-Upper Essequibo
Carollia perspicillata|ABGYE841-06|ROM 113454|Guyana.Upper Takutu-Upper Essequibo
Carollia perspicillata|ABGYE826-06|ROM 113434|Guyana.Upper Demerara-Berbice
Carollia perspicillata|ABGYE813-06|ROM 113417|Guyana.Upper Demerara-Berbice
Carollia perspicillata|ABGYE760-06|ROM 113359|Guyana.Upper Demerara-Berbice
Carollia perspicillata|ABGYE636-06|ROM 112551|Guyana.Demerara-Mahaica
Carollia perspicillata|ABGYD461-06|ROM 108812|Guyana.Potaro-Siparuni
Carollia perspicillata|ABGYD434-06|ROM 108785|Guyana.Potaro-Siparuni
Carollia perspicillata|ABGYD218-06|ROM 108549|Guyana.Potaro-Siparuni
Carollia perspicillata|ABGYB786-06|ROM 103554|Guyana.Upper Demerara-Berbice
Carollia perspicillata|ABGYA450-06|ROM 98860|Guyana.Barima-Waini
Carollia perspicillata|ABGYA394-06|ROM 98799|Guyana.Barima-Waini
Carollia perspicillata|ABGYA350-06|ROM 98750|Guyana.Barima-Waini
Carollia perspicillata|ABGYA248-06|ROM 98137|Guyana.Upper Demerara-Berbice
Carollia perspicillata|ABGYA246-06|ROM 98135|Guyana.Upper Demerara-Berbice
Carollia perspicillata|ABGYA243-06|ROM 98132|Guyana.Upper Demerara-Berbice
Carollia perspicillata|ABGYA242-06|ROM 98131|Guyana.Upper Demerara-Berbice
Carollia perspicillata|ABGYA130-06|ROM 97972|Guyana.Upper Takutu-Upper Essequibo
Carollia perspicillata|ABGYA004-06|ROM 97772|Guyana.Upper Takutu-Upper Essequibo
Carollia perspicillata|ABGYA280-06|ROM 98185|Guyana.Demerara-Mahaica
Carollia perspicillata|ABGYB118-06|ROM 101099|Guyana.Barima-Waini
Carollia perspicillata|ABGYB018-06|ROM 100989|Guyana.Barima-Waini
Carollia perspicillata|ABGYC060-06|ROM 106546|Guyana.Upper Takutu-Upper Essequibo
Carollia perspicillata|ABGYG776-08|ROM 119204|Guyana
Carollia perspicillata|ABGYB235-06|ROM 102937|Guyana.Upper Takutu-Upper Essequibo
Carollia perspicillata|BCBNT242-06|ROM 108157|Guyana.Cuyuni-Mazaruni
Carollia perspicillata|ABGYB045-06|ROM 101017|Guyana.Barima-Waini
Carollia perspicillata|ABGYB015-06|ROM 100986|Guyana.Barima-Waini
Carollia perspicillata|ABGYD030-06|ROM 108252|Guyana.Cuyuni-Mazaruni
Carollia perspicillata|ABGYA270-06|ROM 98170|Guyana.Potaro-Siparuni
Carollia perspicillata|ABGYA791-06|ROM 100844|Guyana.Barima-Waini
Carollia perspicillata|ABGYB013-06|ROM 100984|Guyana.Barima-Waini
Carollia perspicillata|ABGYB114-06|ROM 101092|Guyana.Barima-Waini
Carollia perspicillata|ABGYC007-06|ROM 104784|Guyana.Potaro-Siparuni
Carollia perspicillata|ABGYC320-06|ROM 107132|Guyana.Potaro-Siparuni
Carollia perspicillata|ABGYC329-06|ROM 107141|Guyana.Potaro-Siparuni
Carollia perspicillata|ABGYE690-06|ROM 112620|Guyana.Demerara-Mahaica
Carollia perspicillata|ABGYA526-06|ROM 100193|Guyana.East Berbice-Corentyne
Carollia perspicillata|ABSRA295-06|ROM 116884|Suriname.Nickerie
Carollia perspicillata|BCBN152-05|ROM 98181|Guyana.Demerara-Mahaica
Carollia perspicillata|ABGYG819-08|ROM 119247|Guyana
Carollia perspicillata|ABGYC059-06|ROM 106545|Guyana.Upper Takutu-Upper Essequibo
Carollia perspicillata|ABGYA653-06|ROM 100352|Guyana.East Berbice-Corentyne
Carollia perspicillata|ABGYB526-06|ROM 103261|Guyana.Upper Takutu-Upper Essequibo
Carollia perspicillata|ABGYC213-06|ROM 106754|Guyana.Upper Takutu-Upper Essequibo
Carollia perspicillata|ABGYC319-06|ROM 107131|Guyana.Potaro-Siparuni
Carollia perspicillata|ABGYC519-06|ROM 107357|Guyana.Potaro-Siparuni
Carollia perspicillata|ABGYE601-06|ROM 112080|Guyana.Potaro-Siparuni
Carollia perspicillata|ABGYF279-06|ROM 114626|Guyana.Potaro-Siparuni
Carollia perspicillata|ABGYG929-08|ROM 119357|Guyana
Carollia perspicillata|ABSCA039-06|ROM 104209|Panama.Canal Zone
Carollia perspicillata|ABGYB757-06|ROM 103525|Guyana.Upper Demerara-Berbice
Carollia perspicillata|BCBN603-05|ROM 104196|Panama.Canal Zone
Carollia perspicillata|BCBN504-05|ROM 97498|Mexico.Quintana Roo
Carollia perspicillata|BCBN936-05|ROM 105813|Ecuador.Esmeraldas
Carollia perspicillata|ABSCA140-06|ROM F44046|Costa Rica.Limon
Carollia perspicillata|ABSCA107-06|ROM 104340|Panama.Darien
Carollia perspicillata|ABSCA106-06|ROM 104339|Panama.Darien
Carollia perspicillata|ABSCA090-06|ROM 104313|Panama.Chiriqui
Carollia perspicillata|ABSCA055-06|ROM 104240|Panama
Carollia perspicillata|ABMXC575-06|ROM 97516|Mexico.Quintana Roo
Carollia perspicillata|ABGYG401-06|ROM 116529|Guyana.Potaro-Siparuni
Carollia perspicillata|ABGYC331-06|ROM 107143|Guyana.Potaro-Siparuni
Carollia perspicillata|ABGYB111-06|ROM 101089|Guyana.Barima-Waini
Carollia perspicillata|ABGYB419-06|ROM 103127|Guyana.Upper Takutu-Upper Essequibo
Carollia perspicillata|ABSCA032-06|ROM 104198|Panama.Canal Zone
Carollia perspicillata|ABSRA290-06|ROM 116879|Suriname.Nickerie
Carollia perspicillata|ABGYB282-06|ROM 102986|Guyana.Upper Takutu-Upper Essequibo
Carollia perspicillata|ABGYA795-06|ROM 100848|Guyana.Barima-Waini
Carollia perspicillata|ABGYA266-06|ROM 98166|Guyana.Potaro-Siparuni
Carollia perspicillata|ABSCA038-06|ROM F38040|Panama.Canal Zone
Carollia perspicillata|ABGYG873-08|ROM 119301|Guyana
Carollia perspicillata|ABGYA826-06|ROM 100885|Guyana.Barima-Waini
Carollia perspicillata|ABGYE253-06|ROM 111702|Guyana.Potaro-Siparuni
Carollia perspicillata|ABSMS049-06|ROM 117211|Suriname.Sipaliwini
Carollia perspicillata|BCBNT013-06|ROM 106559|Guyana.Upper Takutu-Upper Essequibo
Carollia perspicillata|BCBNT413-06|ROM 108293|Costa Rica.Limon
Carollia perspicillata|BCBNT549-06|ROM 112173|Nicaragua.Rivas
Carollia perspicillata|ABSA017-06|ROM 107865|Venezuela
Carollia perspicillata|BCBNT425-06|ROM 108305|Costa Rica.Limon
Carollia perspicillata|ABGYC330-06|ROM 107142|Guyana.Potaro-Siparuni
Carollia perspicillata|ABGYG432-06|ROM 116567|Guyana.Potaro-Siparuni
Carollia perspicillata|ABGYA770-06|ROM 100820|Guyana.Barima-Waini
Carollia perspicillata|ABGYA830-06|ROM 100889|Guyana.Barima-Waini
Carollia perspicillata|ABGYC003-06|ROM 104780|Guyana.Potaro-Siparuni
Carollia perspicillata|ABGYC146-06|ROM 106662|Guyana.Upper Takutu-Upper Essequibo
Carollia perspicillata|ABGYC357-06|ROM 107171|Guyana.Potaro-Siparuni
Carollia perspicillata|ABRMM075-07|ROM F39657|Guyana.Potaro-Siparuni
Carollia perspicillata|ABGYA334-06|ROM 98733|Guyana.Barima-Waini
Carollia perspicillata|ABGYA449-06|ROM 98859|Guyana.Barima-Waini
Carollia perspicillata|ABGYA500-06|ROM 98911|Guyana.Barima-Waini
Carollia perspicillata|ABGYD632-06|ROM 109030|Guyana.Potaro-Siparuni
Carollia perspicillata|ABGYC552-06|ROM 107396|Guyana.Potaro-Siparuni
Carollia perspicillata|ABGYB219-06|ROM 102921|Guyana.Upper Takutu-Upper Essequibo
Carollia perspicillata|ABGYA131-06|ROM 97973|Guyana.Potaro-Siparuni
Carollia perspicillata|ABGYD849-06|ROM 109276|Guyana.Potaro-Siparuni
Carollia perspicillata|ABGYE329-06|ROM 111779|Guyana.Potaro-Siparuni
Carollia perspicillata|BCBN324-05|ROM 100855|Guyana.Barima-Waini
Carollia perspicillata|ABECA757-06|ROM 105908|Ecuador.Napo

Carollia perspicillata|ABGYD849-06|ROM 109276|Guyana.Potaro-Siparuni
Carollia perspicillata|ABGYE329-06|ROM 111779|Guyana.Potaro-Siparuni
Carollia perspicillata|BCBN324-05|ROM 100855|Guyana.Barima-Waini
Carollia perspicillata|ABECA757-06|ROM 105908|Ecuador.Napo
Carollia perspicillata|ABGYB213-06|ROM 102915|Guyana.Upper Takutu-Upper Essequibo
Carollia perspicillata|ABGYC476-06|ROM 107309|Guyana.Potaro-Siparuni
Carollia perspicillata|ABGYG374-06|ROM 116497|Guyana.Potaro-Siparuni
Carollia perspicillata|ABGYE627-06|ROM 112540|Guyana.Demerara-Mahaica
Carollia perspicillata|ABGYA611-06|ROM 100298|Guyana.East Berbice-Corentyne
Carollia perspicillata|ABGYA552-06|ROM 100225|Guyana.East Berbice-Corentyne
Carollia perspicillata|ABGYA720-06|ROM 100425|Guyana.East Berbice-Corentyne
Carollia perspicillata|ABGYA861-06|ROM 100932|Guyana.Barima-Waini
Carollia perspicillata|ABGYB093-06|ROM 101070|Guyana.Barima-Waini
Carollia perspicillata|ABGYD576-06|ROM 108955|Guyana.Potaro-Siparuni
Carollia perspicillata|ABGYE603-06|ROM 112082|Guyana.Potaro-Siparuni
Carollia perspicillata|ABGYE631-06|ROM 112544|Guyana.Demerara-Mahaica
Carollia perspicillata|ABGYF156-06|ROM 113709|Guyana.Demerara-Mahaica
Carollia perspicillata|ABGYG592-08|ROM 119020|Guyana
Carollia perspicillata|ABSMS389-06|ROM 117551|Suriname.Sipaliwini
Carollia perspicillata|ABSMS505-06|ROM 117667|Suriname.Sipaliwini
Carollia perspicillata|BCBNC107-06|ROM 115742|Guyana.Potaro-Siparuni
Carollia perspicillata|BCBN537-05|ROM 103353|Guyana.Upper Demerara-Berbice
Carollia perspicillata|ABGYG778-08|ROM 119206|Guyana
Carollia perspicillata|ABSMS320-06|ROM 117482|Suriname.Sipaliwini
Carollia perspicillata|ABGYC061-06|ROM 106547|Guyana.Upper Takutu-Upper Essequibo
Carollia perspicillata|ABGYC234-06|ROM 106784|Guyana.Upper Takutu-Upper Essequibo
Carollia perspicillata|ABGYG817-08|ROM 119245|Guyana
Carollia perspicillata|ABGYB212-06|ROM 102914|Guyana.Upper Takutu-Upper Essequibo
Carollia perspicillata|ABGYB285-06|ROM 102989|Guyana.Upper Takutu-Upper Essequibo
Carollia perspicillata|ABSMS059-06|ROM 117221|Suriname.Sipaliwini
Carollia perspicillata|ABGYA066-06|ROM 97888|Guyana.Upper Takutu-Upper Essequibo
Carollia perspicillata|ABGYD075-06|ROM 108372|Guyana.Potaro-Siparuni
Carollia perspicillata|ABGYE776-06|ROM 113377|Guyana.Upper Demerara-Berbice
Carollia perspicillata|ABGYC036-06|ROM 104813|Guyana.Potaro-Siparuni
Carollia perspicillata|ABGYG405-06|ROM 116533|Guyana.Potaro-Siparuni
Carollia perspicillata|ABSRA292-06|ROM 116881|Suriname.Nickerie
Carollia perspicillata|ABGYG756-08|ROM 119184|Guyana
Carollia perspicillata|ABGYA267-06|ROM 98167|Guyana.Potaro-Siparuni
Carollia perspicillata|ABGYC346-06|ROM 107158|Guyana.Potaro-Siparuni
Carollia perspicillata|ABSA031-06|ROM 107898|Venezuela
Carollia perspicillata|BCBN741-05|ROM 104445|Ecuador.Napo
Carollia perspicillata|ABGYA652-06|ROM 100351|Guyana.East Berbice-Corentyne
Carollia perspicillata|ABGYE641-06|ROM 112556|Guyana.Demerara-Mahaica
Carollia perspicillata|ABSMS145-06|ROM 117307|Suriname.Sipaliwini
Carollia perspicillata|BCBNT474-06|ROM 111075|Brazil.Sao Paulo
Carollia perspicillata|ABGYB037-06|ROM 101009|Guyana.Barima-Waini
Carollia perspicillata|ABGYD395-06|ROM 108741|Guyana.Potaro-Siparuni
Carollia perspicillata|ABGYB881-06|ROM 104725|Guyana.Potaro-Siparuni
Carollia perspicillata|ABGYB420-06|ROM 103128|Guyana.Upper Takutu-Upper Essequibo
Carollia perspicillata|ABGYD029-06|ROM 108251|Guyana.Cuyuni-Mazaruni
Carollia perspicillata|ABGYA151-06|ROM 97996|Guyana.Potaro-Siparuni
Carollia perspicillata|BCBNT166-06|ROM 107840|Venezuela.Amazonas
Carollia perspicillata|ABGYA065-06|ROM 97887|Guyana.Upper Takutu-Upper Essequibo
Carollia perspicillata|ABGYG056-06|ROM 115568|Guyana.Essequibo Islands-West Demerara
Carollia perspicillata|ABGYD241-06|ROM 108573|Guyana.Potaro-Siparuni
Carollia perspicillata|ABGYA271-06|ROM 98171|Guyana.Potaro-Siparuni
Carollia perspicillata|ABGYA768-06|ROM 100818|Guyana.Barima-Waini
Carollia perspicillata|ABGYB255-06|ROM 102959|Guyana.Upper Takutu-Upper Essequibo
Carollia perspicillata|ABGYE337-06|ROM 111787|Guyana.Potaro-Siparuni
Carollia perspicillata|ABGYC345-06|ROM 107157|Guyana.Potaro-Siparuni
Carollia perspicillata|ABGYG753-08|ROM 119181|Guyana
Carollia perspicillata|ABSMS143-06|ROM 117305|Suriname.Sipaliwini
Carollia perspicillata|BCBNT074-06|ROM 107030|Guyana.Potaro-Siparuni
Carollia perspicillata|ABGYA034-06|ROM 97824|Guyana.Upper Takutu-Upper Essequibo
Carollia perspicillata|ABGYA790-06|ROM 100843|Guyana.Barima-Waini
Carollia perspicillata|ABGYF071-06|ROM 113597|Guyana.Upper Takutu-Upper Essequibo
Carollia perspicillata|ABGYA718-06|ROM 100423|Guyana.East Berbice-Corentyne
Carollia perspicillata|ABGYB004-06|ROM 100971|Guyana.Barima-Waini
Carollia perspicillata|ABGYD612-06|ROM 108982|Guyana.Potaro-Siparuni
Carollia perspicillata|ABGYC040-06|ROM 104817|Guyana.Potaro-Siparuni
Carollia perspicillata|ABGYA273-06|ROM 98173|Guyana.Potaro-Siparuni
Carollia perspicillata|ABGYE249-06|ROM 111698|Guyana.Potaro-Siparuni
Carollia perspicillata|ABGYE323-06|ROM 111773|Guyana.Potaro-Siparuni
Carollia perspicillata|ABSRA476-06|ROM 117066|Suriname.Nickerie
Carollia perspicillata|BCBNT653-06|ROM 113634|Guyana.Demerara-Mahaica
Carollia perspicillata|ABGYA227-06|ROM 98103|Guyana.Upper Takutu-Upper Essequibo
Carollia perspicillata|ABGYD596-06|ROM 109019|Guyana.Potaro-Siparuni
Carollia perspicillata|ABGYG152-06|ROM 115712|Guyana.Potaro-Siparuni
Carollia perspicillata|ABGYA597-06|ROM 100284|Guyana.East Berbice-Corentyne
Carollia perspicillata|ABGYA387-06|ROM 98791|Guyana.Barima-Waini
Carollia perspicillata|ABGYA314-06|ROM 98710|Guyana.Barima-Waini
Carollia perspicillata|ABGYA776-06|ROM 100826|Guyana.Barima-Waini
Carollia perspicillata|ABGYD059-06|ROM 108355|Guyana.Potaro-Siparuni
Carollia perspicillata|ABSMS525-06|ROM 117687|Suriname.Sipaliwini
Carollia perspicillata|BCBNT656-06|ROM 113637|Guyana.Demerara-Mahaica
Carollia perspicillata|ABGYG816-08|ROM 119244|Guyana
Carollia perspicillata|ABGYA862-06|ROM 100933|Guyana.Barima-Waini
Carollia perspicillata|ABGYG759-08|ROM 119187|Guyana
Carollia perspicillata|ABGYC008-06|ROM 104785|Guyana.Potaro-Siparuni
Carollia perspicillata|ABSRA503-06|ROM 117093|Suriname.Nickerie
Carollia perspicillata|ABGYG644-08|ROM 119072|Guyana
Carollia perspicillata|ABGYG123-06|ROM 115677|Guyana.Potaro-Siparuni
Carollia perspicillata|ABGYD061-06|ROM 108357|Guyana.Potaro-Siparuni
Carollia perspicillata|ABGYC149-06|ROM 106665|Guyana.Upper Takutu-Upper Essequibo
Carollia perspicillata|ABGYA229-06|ROM 98105|Guyana.Upper Takutu-Upper Essequibo
Carollia perspicillata|ABGYA301-06|ROM 98697|Guyana.Barima-Waini
Carollia perspicillata|ABGYB066-06|ROM 101042|Guyana.Barima-Waini
Carollia perspicillata|ABSMS023-06|ROM 117185|Suriname.Sipaliwini
Carollia perspicillata|BCBNT609-06|ROM 113512|Guyana.Upper Takutu-Upper Essequibo
Carollia perspicillata|BCBNT625-06|ROM 113551|Guyana.Upper Takutu-Upper Essequibo
Carollia perspicillata|ABGYD367-06|ROM 108710|Guyana.Potaro-Siparuni
Carollia perspicillata|ABGYA015-06|ROM 97797|Guyana.Upper Takutu-Upper Essequibo
Carollia perspicillata|ABGYA019-06|ROM 97801|Guyana.Upper Takutu-Upper Essequibo
[illegible]

Carollia perspicillata|ABGYD367-06|ROM 108710|Guyana.Potaro-Siparuni
Carollia perspicillata|ABGYA015-06|ROM 97797|Guyana.Upper Takutu-Upper Essequibo
Carollia perspicillata|ABGYA019-06|ROM 97801|Guyana.Upper Takutu-Upper Essequibo
Carollia perspicillata|ABGYD547-06|ROM 108912|Guyana.Potaro-Siparuni
Carollia perspicillata|ABGYB121-06|ROM 101102|Guyana.Barima-Waini
Carollia perspicillata|ABGYD839-06|ROM 109265|Guyana.Potaro-Siparuni
Carollia perspicillata|ABSRA451-06|ROM 117041|Suriname.Nickerie
Carollia perspicillata|ABSRA452-06|ROM 117042|Suriname.Nickerie
Carollia perspicillata|ABGYA774-06|ROM 100824|Guyana.Barima-Waini
Carollia perspicillata|ABGYB902-06|ROM 104747|Guyana.Potaro-Siparuni
Carollia perspicillata|BCBN290-05|ROM 100246|Guyana.East Berbice-Corentyne
Carollia perspicillata|ABGYG758-08|ROM 119186|Guyana
Carollia perspicillata|BCBNT011-06|ROM 106557|Guyana.Upper Takutu-Upper Essequibo
Carollia perspicillata|BCBN107-05|ROM 97813|Guyana.Upper Takutu-Upper Essequibo
Carollia perspicillata|ABSRA377-06|ROM 116966|Suriname.Nickerie
Carollia perspicillata|ABGYD105-06|ROM 108403|Guyana.Potaro-Siparuni
Carollia perspicillata|ABGYC792-06|ROM F43227|Guyana.Cuyuni-Mazaruni
Carollia perspicillata|ABGYA717-06|ROM 100422|Guyana.East Berbice-Corentyne
Carollia perspicillata|ABGYG643-08|ROM 119071|Guyana
Carollia perspicillata|ABSRA267-06|ROM 116856|Suriname.Nickerie
Carollia perspicillata|ABGYF058-06|ROM 113571|Guyana.Upper Takutu-Upper Essequibo
Carollia perspicillata|ABGYA654-06|ROM 100353|Guyana.East Berbice-Corentyne
Carollia perspicillata|ABGYF027-06|ROM 113522|Guyana.Upper Takutu-Upper Essequibo
Carollia perspicillata|BCBNC123-06|ROM 115778|Guyana.Potaro-Siparuni
Carollia perspicillata|ABGYC196-06|ROM 106735|Guyana.Upper Takutu-Upper Essequibo
Carollia perspicillata|ABGYG392-06|ROM 116518|Guyana.Potaro-Siparuni
Carollia perspicillata|ABGYA016-06|ROM 97798|Guyana.Upper Takutu-Upper Essequibo
Carollia perspicillata|ABGYG677-08|ROM 119105|Guyana
Carollia perspicillata|ABSMS504-06|ROM 117666|Suriname.Sipaliwini
Carollia perspicillata|ABGYA269-06|ROM 98169|Guyana.Potaro-Siparuni
Carollia perspicillata|ABGYA609-06|ROM 100296|Guyana.East Berbice-Corentyne
Carollia perspicillata|ABGYA226-06|ROM 98102|Guyana.Upper Takutu-Upper Essequibo
Carollia perspicillata|ABGYA172-06|ROM 98031|Guyana.Potaro-Siparuni
Carollia perspicillata|ABGYA170-06|ROM 98029|Guyana.Potaro-Siparuni
Carollia perspicillata|ABGYA150-06|ROM 97995|Guyana.Potaro-Siparuni
Carollia perspicillata|ABGYA029-06|ROM 97819|Guyana.Upper Takutu-Upper Essequibo
Carollia perspicillata|ABGYA028-06|ROM 97818|Guyana.Upper Takutu-Upper Essequibo
Carollia perspicillata|ABGYA018-06|ROM 97800|Guyana.Upper Takutu-Upper Essequibo
Carollia perspicillata|ABECA759-06|ROM 105910|Ecuador.Napo
Carollia perspicillata|ABSA036-06|ROM 107929|Venezuela.Bolivar
Carollia perspicillata|ABGYB744-06|ROM 103511|Guyana.Upper Demerara-Berbice
Carollia perspicillata|ABGYB210-06|ROM 102912|Guyana.Upper Takutu-Upper Essequibo
Carollia perspicillata|ABGYA251-06|ROM 98140|Guyana.Upper Demerara-Berbice
Carollia perspicillata|ABECA325-06|ROM F37525|Ecuador.Napo
Carollia perspicillata|ABECA284-06|ROM 105154|Ecuador.Napo
Carollia perspicillata|ABGYD704-06|ROM 109118|Guyana.Potaro-Siparuni
Carollia perspicillata|ABGYB882-06|ROM 104726|Guyana.Potaro-Siparuni
Carollia perspicillata|ABGYC747-06|ROM F43170|Guyana.Cuyuni-Mazaruni
Carollia perspicillata|ABGYC793-06|ROM 108192|Guyana.Cuyuni-Mazaruni
Carollia perspicillata|ABGYA311-06|ROM 98707|Guyana.Barima-Waini
Carollia perspicillata|ABGYA767-06|ROM 100817|Guyana.Barima-Waini
Carollia perspicillata|ABGYB068-06|ROM 101044|Guyana.Barima-Waini
Carollia perspicillata|ABGYB107-06|ROM 101085|Guyana.Barima-Waini
Carollia perspicillata|ABGYC721-06|ROM 108121|Guyana.Cuyuni-Mazaruni
Carollia perspicillata|ABGYB220-06|ROM 102922|Guyana.Upper Takutu-Upper Essequibo
Carollia perspicillata|ABGYB211-06|ROM 102913|Guyana.Upper Takutu-Upper Essequibo
Carollia perspicillata|ABGYC381-06|ROM 107198|Guyana.Potaro-Siparuni
Carollia perspicillata|ABGYC673-06|ROM 108079|Guyana.Cuyuni-Mazaruni
Carollia perspicillata|ABGYC689-06|ROM F39743|Guyana.Cuyuni-Mazaruni
Carollia perspicillata|ABGYG030-06|ROM 115536|Guyana.Essequibo Islands-West Demerara
Carollia perspicillata|ABGYG393-06|ROM 116519|Guyana.Potaro-Siparuni
Carollia perspicillata|ABGYF155-06|ROM 113708|Guyana.Demerara-Mahaica
Carollia perspicillata|ABSRA284-06|ROM 116873|Suriname.Nickerie
Carollia perspicillata|ABECA049-06|ROM F37134|Ecuador.Napo
Carollia perspicillata|ABGYA528-06|ROM 100195|Guyana.East Berbice-Corentyne
Carollia perspicillata|ABGYA534-06|ROM 100201|Guyana.East Berbice-Corentyne
Carollia perspicillata|ABGYA523-06|ROM 100190|Guyana.East Berbice-Corentyne
Carollia perspicillata|ABGYD476-06|ROM 108827|Guyana.Potaro-Siparuni
Carollia perspicillata|ABGYE640-06|ROM 112555|Guyana.Demerara-Mahaica
Carollia perspicillata|ABGYG876-08|ROM 119304|Guyana
Carollia perspicillata|ABGYB109-06|ROM 101087|Guyana.Barima-Waini
Carollia perspicillata|ABGYA017-06|ROM 97799|Guyana.Upper Takutu-Upper Essequibo
Carollia perspicillata|ABSMS461-06|ROM 117623|Suriname.Sipaliwini
Carollia perspicillata|ABECA194-06|ROM 104512|Ecuador.Napo
Carollia perspicillata|ABGYA173-06|ROM 98032|Guyana.Potaro-Siparuni
Carollia perspicillata|ABGYE328-06|ROM 111778|Guyana.Potaro-Siparuni
Carollia perspicillata|ABGYF103-06|ROM 113642|Guyana.Demerara-Mahaica
Carollia perspicillata|ABSA037-06|ROM 107930|Venezuela.Bolivar
Carollia perspicillata|ABGYF044-06|ROM 113552|Guyana.Upper Takutu-Upper Essequibo
Carollia perspicillata|ABGYG933-08|ROM 119361|Guyana
Carollia perspicillata|ABGYD073-06|ROM 108370|Guyana.Potaro-Siparuni
Carollia perspicillata|ABGYB003-06|ROM 100970|Guyana.Barima-Waini
Carollia perspicillata|ABGYA383-06|ROM 98787|Guyana.Barima-Waini
Carollia perspicillata|ABGYA744-06|ROM 100450|Guyana.East Berbice-Corentyne
Carollia perspicillata|ABSRA293-06|ROM 116882|Suriname.Nickerie
Carollia perspicillata|ABGYA794-06|ROM 100847|Guyana.Barima-Waini
Carollia perspicillata|BCBNT016-06|ROM 106562|Guyana.Upper Takutu-Upper Essequibo
Carollia perspicillata|ABGYA358-06|ROM 98759|Guyana.Barima-Waini
Carollia perspicillata|ABGYA359-06|ROM 98760|Guyana.Barima-Waini
Carollia perspicillata|ABGYD060-06|ROM 108356|Guyana.Potaro-Siparuni
Carollia perspicillata|ABGYD464-06|ROM 108815|Guyana.Potaro-Siparuni
Carollia perspicillata|ABGYE491-06|ROM 111963|Guyana.Potaro-Siparuni
Carollia perspicillata|ABGYC437-06|ROM 107261|Guyana.Potaro-Siparuni
Carollia perspicillata|ABGYG591-08|ROM 119019|Guyana
Carollia perspicillata|ABGYG675-08|ROM 119103|Guyana
Carollia perspicillata|ABGYG676-08|ROM 119104|Guyana
Carollia perspicillata|ABGYE492-06|ROM 111964|Guyana.Potaro-Siparuni
Carollia perspicillata|ABGYB878-06|ROM 104722|Guyana.Potaro-Siparuni
Carollia perspicillata|ABGYA247-06|ROM 98136|Guyana.Upper Demerara-Berbice
Carollia perspicillata|ABGYG932-08|ROM 119360|Guyana
Carollia perspicillata|ABGYA810-06|ROM 100869|Guyana.Barima-Waini
Carollia perspicillata|ABGYC348-06|ROM 107160|Guyana.Potaro-Siparuni
Carollia perspicillata|ABGYA742-06|ROM 100448|Guyana.East Berbice-Corentyne

Carollia perspicillata|ABGYA810-06|ROM 100869|Guyana.Barima-Waini
Carollia perspicillata|ABGYC348-06|ROM 107160|Guyana.Potaro-Siparuni
Carollia perspicillata|ABGYA742-06|ROM 100448|Guyana.East Berbice-Corentyne
Carollia perspicillata|ABGYA792-06|ROM 100845|Guyana.Barima-Waini
Carollia perspicillata|ABGYB115-06|ROM 101093|Guyana.Barima-Waini
Carollia perspicillata|ABGYB281-06|ROM 102985|Guyana.Upper Takutu-Upper Essequibo
Carollia perspicillata|ABGYE122-06|ROM 111565|Guyana.Potaro-Siparuni
Carollia perspicillata|ABGYA422-06|ROM 98830|Guyana.Barima-Waini
Carollia perspicillata|ABGYA479-06|ROM 98889|Guyana.Barima-Waini
Carollia perspicillata|ABGYA396-06|ROM 98802|Guyana.Barima-Waini
Carollia perspicillata|ABGYF099-06|ROM 113638|Guyana.Demerara-Mahaica
Carollia perspicillata|ABSRA445-06|ROM 117035|Suriname.Nickerie
Carollia perspicillata|BCBNT218-06|ROM 107933|Venezuela.Bolivar
Carollia perspicillata|ABGYA245-06|ROM 98134|Guyana.Upper Demerara-Berbice
Carollia perspicillata|ABGYA272-06|ROM 98172|Guyana.Potaro-Siparuni
Carollia perspicillata|ABGYA274-06|ROM 98174|Guyana.Potaro-Siparuni
Carollia perspicillata|ABGYA281-06|ROM 98186|Guyana.Demerara-Mahaica
Carollia perspicillata|ABGYA282-06|ROM 98187|Guyana.Demerara-Mahaica
Carollia perspicillata|ABGYA283-06|ROM 98188|Guyana.Demerara-Mahaica
Carollia perspicillata|ABGYA308-06|ROM 98704|Guyana.Barima-Waini
Carollia perspicillata|ABGYA309-06|ROM 98705|Guyana.Barima-Waini
Carollia perspicillata|ABGYA343-06|ROM 98743|Guyana.Barima-Waini
Carollia perspicillata|ABGYA374-06|ROM 98778|Guyana.Barima-Waini
Carollia perspicillata|ABGYA395-06|ROM 98801|Guyana.Barima-Waini
Carollia perspicillata|ABGYA451-06|ROM 98861|Guyana.Barima-Waini
Carollia perspicillata|ABGYA478-06|ROM 98888|Guyana.Barima-Waini
Carollia perspicillata|ABGYA489-06|ROM 98899|Guyana.Barima-Waini
Carollia perspicillata|ABGYA490-06|ROM 98900|Guyana.Barima-Waini
Carollia perspicillata|ABGYA505-06|ROM 98916|Guyana.Barima-Waini
Carollia perspicillata|ABGYA506-06|ROM 98917|Guyana.Barima-Waini
Carollia perspicillata|ABGYA518-06|ROM 100185|Guyana.East Berbice-Corentyne
Carollia perspicillata|ABGYA525-06|ROM 100192|Guyana.East Berbice-Corentyne
Carollia perspicillata|ABGYA530-06|ROM 100197|Guyana.East Berbice-Corentyne
Carollia perspicillata|ABGYA531-06|ROM 100198|Guyana.East Berbice-Corentyne
Carollia perspicillata|ABGYA532-06|ROM 100199|Guyana.East Berbice-Corentyne
Carollia perspicillata|ABGYA547-06|ROM 100219|Guyana.East Berbice-Corentyne
Carollia perspicillata|ABGYA570-06|ROM 100249|Guyana.East Berbice-Corentyne
Carollia perspicillata|ABGYA607-06|ROM 100294|Guyana.East Berbice-Corentyne
Carollia perspicillata|ABGYA608-06|ROM 100295|Guyana.East Berbice-Corentyne
Carollia perspicillata|ABGYA612-06|ROM 100299|Guyana.East Berbice-Corentyne
Carollia perspicillata|ABGYA797-06|ROM 100850|Guyana.Barima-Waini
Carollia perspicillata|ABGYA825-06|ROM 100884|Guyana.Barima-Waini
Carollia perspicillata|ABGYB006-06|ROM 100973|Guyana.Barima-Waini
Carollia perspicillata|ABGYB014-06|ROM 100985|Guyana.Barima-Waini
Carollia perspicillata|ABGYB020-06|ROM 100991|Guyana.Barima-Waini
Carollia perspicillata|ABGYB042-06|ROM 101014|Guyana.Barima-Waini
Carollia perspicillata|ABGYB089-06|ROM 101066|Guyana.Barima-Waini
Carollia perspicillata|ABGYB094-06|ROM 101071|Guyana.Barima-Waini
Carollia perspicillata|ABGYB110-06|ROM 101088|Guyana.Barima-Waini
Carollia perspicillata|ABGYB209-06|ROM 102911|Guyana.Upper Takutu-Upper Essequibo
Carollia perspicillata|ABGYB232-06|ROM 102934|Guyana.Upper Takutu-Upper Essequibo
Carollia perspicillata|ABGYB234-06|ROM 102936|Guyana.Upper Takutu-Upper Essequibo
Carollia perspicillata|ABGYB284-06|ROM 102988|Guyana.Upper Takutu-Upper Essequibo
Carollia perspicillata|ABGYB333-06|ROM 103040|Guyana.Upper Takutu-Upper Essequibo
Carollia perspicillata|ABGYB527-06|ROM 103262|Guyana.Upper Takutu-Upper Essequibo
Carollia perspicillata|ABGYB731-06|ROM 103498|Guyana.Upper Demerara-Berbice
Carollia perspicillata|ABGYB787-06|ROM 103555|Guyana.Upper Demerara-Berbice
Carollia perspicillata|ABGYB904-06|ROM 104749|Guyana.Potaro-Siparuni
Carollia perspicillata|ABGYC005-06|ROM 104782|Guyana.Potaro-Siparuni
Carollia perspicillata|ABGYC039-06|ROM 104816|Guyana.Potaro-Siparuni
Carollia perspicillata|ABGYC062-06|ROM 106548|Guyana.Upper Takutu-Upper Essequibo
Carollia perspicillata|ABGYC155-06|ROM 106677|Guyana.Upper Takutu-Upper Essequibo
Carollia perspicillata|ABGYC271-06|ROM 107060|Guyana.Potaro-Siparuni
Carollia perspicillata|ABGYC272-06|ROM 107061|Guyana.Potaro-Siparuni
Carollia perspicillata|ABGYC276-06|ROM 107067|Guyana.Potaro-Siparuni
Carollia perspicillata|ABGYC306-06|ROM 107117|Guyana.Potaro-Siparuni
Carollia perspicillata|ABGYC525-06|ROM 107363|Guyana.Potaro-Siparuni
Carollia perspicillata|ABGYC676-06|ROM F39730|Guyana.Cuyuni-Mazaruni
Carollia perspicillata|ABGYC678-06|ROM F39732|Guyana.Cuyuni-Mazaruni
Carollia perspicillata|ABGYC684-06|ROM 108086|Guyana.Cuyuni-Mazaruni
Carollia perspicillata|ABGYC685-06|ROM 108087|Guyana.Cuyuni-Mazaruni
Carollia perspicillata|ABGYC687-06|ROM F39741|Guyana.Cuyuni-Mazaruni
Carollia perspicillata|ABGYC690-06|ROM 108089|Guyana.Cuyuni-Mazaruni
Carollia perspicillata|ABGYC723-06|ROM 108123|Guyana.Cuyuni-Mazaruni
Carollia perspicillata|ABGYC815-06|ROM 108218|Guyana.Cuyuni-Mazaruni
Carollia perspicillata|ABGYD031-06|ROM 108253|Guyana.Cuyuni-Mazaruni
Carollia perspicillata|ABGYD032-06|ROM 108254|Guyana.Cuyuni-Mazaruni
Carollia perspicillata|ABGYD034-06|ROM 108256|Guyana.Cuyuni-Mazaruni
Carollia perspicillata|ABGYD035-06|ROM 108257|Guyana.Cuyuni-Mazaruni
Carollia perspicillata|ABGYD103-06|ROM 108401|Guyana.Potaro-Siparuni
Carollia perspicillata|ABGYD127-06|ROM 108430|Guyana.Potaro-Siparuni
Carollia perspicillata|ABGYD157-06|ROM 108469|Guyana.Potaro-Siparuni
Carollia perspicillata|ABGYD158-06|ROM 108470|Guyana.Potaro-Siparuni
Carollia perspicillata|ABGYD180-06|ROM 108506|Guyana.Potaro-Siparuni
Carollia perspicillata|ABGYD198-06|ROM 108527|Guyana.Potaro-Siparuni
Carollia perspicillata|ABGYD240-06|ROM 108572|Guyana.Potaro-Siparuni
Carollia perspicillata|ABGYD391-06|ROM 108737|Guyana.Potaro-Siparuni
Carollia perspicillata|ABGYD392-06|ROM 108738|Guyana.Potaro-Siparuni
Carollia perspicillata|ABGYD425-06|ROM 108775|Guyana.Potaro-Siparuni
Carollia perspicillata|ABGYD460-06|ROM 108811|Guyana.Potaro-Siparuni
Carollia perspicillata|ABGYD462-06|ROM 108813|Guyana.Potaro-Siparuni
Carollia perspicillata|ABGYD465-06|ROM 108816|Guyana.Potaro-Siparuni
Carollia perspicillata|ABGYD504-06|ROM 108862|Guyana.Potaro-Siparuni
Carollia perspicillata|ABGYD556-06|ROM 108926|Guyana.Potaro-Siparuni
Carollia perspicillata|ABGYD575-06|ROM 108954|Guyana.Potaro-Siparuni
Carollia perspicillata|ABGYD714-06|ROM 109129|Guyana.Potaro-Siparuni
Carollia perspicillata|ABGYD715-06|ROM 109130|Guyana.Potaro-Siparuni
Carollia perspicillata|ABGYD848-06|ROM 109275|Guyana.Potaro-Siparuni
Carollia perspicillata|ABGYE061-06|ROM 109347|Guyana.Potaro-Siparuni
Carollia perspicillata|ABGYE111-06|ROM 111554|Guyana.Potaro-Siparuni
Carollia perspicillata|ABGYE130-06|ROM 111573|Guyana.Potaro-Siparuni
Carollia perspicillata|ABGYE409-06|ROM 111862|Guyana.Potaro-Siparuni
Carollia perspicillata|ABGYE413-06|ROM 111868|Guyana.Potaro-Siparuni

[illegible]
Carollia perspicillata|ABGYE130-06|ROM 111573|Guyana.Potaro-Siparuni
Carollia perspicillata|ABGYE409-06|ROM 111862|Guyana.Potaro-Siparuni
Carollia perspicillata|ABGYE413-06|ROM 111868|Guyana.Potaro-Siparuni
Carollia perspicillata|ABGYE450-06|ROM 111913|Guyana.Potaro-Siparuni
Carollia perspicillata|ABGYE637-06|ROM 112552|Guyana.Demerara-Mahaica
Carollia perspicillata|ABGYE837-06|ROM 113449|Guyana.Upper Demerara-Berbice
Carollia perspicillata|ABGYE857-06|ROM 113473|Guyana.Upper Takutu-Upper Essequibo
Carollia perspicillata|ABGYF070-06|ROM 113594|Guyana.Upper Takutu-Upper Essequibo
Carollia perspicillata|ABGYF096-06|ROM 113630|Guyana.Demerara-Mahaica
Carollia perspicillata|ABGYF102-06|ROM 113641|Guyana.Demerara-Mahaica
Carollia perspicillata|ABGYG015-06|ROM 115498|Guyana.Essequibo Islands-West Demerara
Carollia perspicillata|ABGYG017-06|ROM 115503|Guyana.Essequibo Islands-West Demerara
Carollia perspicillata|ABGYG039-06|ROM 115549|Guyana.Essequibo Islands-West Demerara
Carollia perspicillata|ABGYG040-06|ROM 115550|Guyana.Essequibo Islands-West Demerara
Carollia perspicillata|ABGYG053-06|ROM 115565|Guyana.Essequibo Islands-West Demerara
Carollia perspicillata|ABGYG058-06|ROM 115573|Guyana.Essequibo Islands-West Demerara
Carollia perspicillata|ABGYG155-06|ROM 115715|Guyana.Potaro-Siparuni
Carollia perspicillata|ABGYG586-08|ROM 119014|Guyana
Carollia perspicillata|ABGYG678-08|ROM 119106|Guyana
Carollia perspicillata|ABGYG680-08|ROM 119108|Guyana
Carollia perspicillata|ABGYG718-08|ROM 119146|Guyana
Carollia perspicillata|ABGYG874-08|ROM 119302|Guyana
Carollia perspicillata|ABGYG877-08|ROM 119305|Guyana
Carollia perspicillata|ABGYG879-08|ROM 119307|Guyana
Carollia perspicillata|ABGYG912-08|ROM 119340|Guyana
Carollia perspicillata|ABMXC574-06|ROM 97515|Mexico.Quintana Roo
Carollia perspicillata|ABSA005-06|ROM 107849|Venezuela
Carollia perspicillata|ABSA030-06|ROM 107897|Venezuela
Carollia perspicillata|ABSA042-06|ROM 107941|Venezuela.Bolivar
Carollia perspicillata|ABSMS021-06|ROM 117183|Suriname.Sipaliwini
Carollia perspicillata|ABSMS024-06|ROM 117186|Suriname.Sipaliwini
Carollia perspicillata|ABSMS241-06|ROM 117403|Suriname.Sipaliwini
Carollia perspicillata|ABSMS256-06|ROM 117418|Suriname.Sipaliwini
Carollia perspicillata|ABSMS298-06|ROM 117460|Suriname.Sipaliwini
Carollia perspicillata|ABSMS500-06|ROM 117662|Suriname.Sipaliwini
Carollia perspicillata|ABSRA268-06|ROM 116857|Suriname.Nickerie
Carollia perspicillata|ABSRA287-06|ROM 116876|Suriname.Nickerie
Carollia perspicillata|BCBN103-05|ROM 97793|Guyana.Upper Takutu-Upper Essequibo
Carollia perspicillata|BCBN108-05|ROM 97814|Guyana.Upper Takutu-Upper Essequibo
Carollia perspicillata|BCBN110-05|ROM 97816|Guyana.Upper Takutu-Upper Essequibo
Carollia perspicillata|BCBN111-05|ROM 97817|Guyana.Upper Takutu-Upper Essequibo
Carollia perspicillata|BCBN127-05|ROM 97884|Guyana.Upper Takutu-Upper Essequibo
Carollia perspicillata|BCBN128-05|ROM 97885|Guyana.Upper Takutu-Upper Essequibo
Carollia perspicillata|BCBN151-05|ROM 98180|Guyana.Demerara-Mahaica
Carollia perspicillata|BCBN288-05|ROM 100244|Guyana.East Berbice-Corentyne
Carollia perspicillata|BCBN292-05|ROM 100256|Guyana.East Berbice-Corentyne
Carollia perspicillata|BCBN323-05|ROM 100854|Guyana.Barima-Waini
Carollia perspicillata|BCBNT241-06|ROM 108156|Guyana.Cuyuni-Mazaruni
Carollia perspicillata|BCBNT528-06|ROM 111910|Guyana.Potaro-Siparuni
Carollia perspicillata|BCBNT633-06|ROM 113575|Guyana.Upper Takutu-Upper Essequibo
Carollia perspicillata|BCBNT634-06|ROM 113576|Guyana.Upper Takutu-Upper Essequibo
Carollia perspicillata|BCBNT660-06|ROM 113658|Guyana.Demerara-Mahaica
Carollia perspicillata|BCBNT698-06|ROM 113830|Guyana.Demerara-Mahaica
Carollia perspicillata|BCBNT705-06|ROM 113874|Suriname.Brokopondo
Carollia perspicillata|BCBNT706-06|ROM 113875|Suriname.Brokopondo
Carollia perspicillata|BCBNT905-06|ROM 115563|Guyana.Essequibo Islands-West Demerara
Carollia perspicillata|BCBNT911-06|ROM 115576|Guyana.Essequibo Islands-West Demerara
Carollia perspicillata|BCBNT914-06|ROM 115580|Guyana.Essequibo Islands-West Demerara
Carollia perspicillata|BCBNT973-06|ROM 114622|Guyana.Potaro-Siparuni
Carollia perspicillata|ABGYA081-06|ROM 97904|Guyana.Upper Takutu-Upper Essequibo
Carollia perspicillata|ABGYA082-06|ROM 97905|Guyana.Upper Takutu-Upper Essequibo
Carollia perspicillata|BCBNC188-06|ROM 116561|Guyana.Potaro-Siparuni
Carollia perspicillata|ABGYA613-06|ROM 100300|Guyana.East Berbice-Corentyne
Carollia perspicillata|ABGYC004-06|ROM 104781|Guyana.Potaro-Siparuni
Carollia perspicillata|ABGYG112-06|ROM 115661|Guyana.Potaro-Siparuni
Carollia perspicillata|ABGYE602-06|ROM 112081|Guyana.Potaro-Siparuni
Carollia perspicillata|ABGYA773-06|ROM 100823|Guyana.Barima-Waini
Carollia perspicillata|ABGYC754-06|ROM 108153|Guyana.Cuyuni-Mazaruni
Carollia perspicillata|ABGYC732-06|ROM 108130|Guyana.Cuyuni-Mazaruni
Carollia perspicillata|ABGYE479-06|ROM 111951|Guyana.Potaro-Siparuni
Carollia perspicillata|ABGYE645-06|ROM 112560|Guyana.Demerara-Mahaica
Carollia perspicillata|ABGYG588-08|ROM 119016|Guyana
Carollia perspicillata|ABGYF095-06|ROM 113629|Guyana.Demerara-Mahaica
Carollia perspicillata|ABGYF088-06|ROM 113619|Guyana.Demerara-Mahaica
Carollia perspicillata|ABGYE625-06|ROM 112538|Guyana.Demerara-Mahaica
Carollia perspicillata|ABGYD389-06|ROM 108735|Guyana.Potaro-Siparuni
Carollia perspicillata|ABGYC222-06|ROM 106766|Guyana.Upper Takutu-Upper Essequibo
Carollia perspicillata|ABGYB918-06|ROM 104763|Guyana.Potaro-Siparuni
Carollia perspicillata|ABGYB618-06|ROM 103371|Guyana.Upper Demerara-Berbice
Carollia perspicillata|ABGYA554-06|ROM 100229|Guyana.East Berbice-Corentyne
Carollia perspicillata|ABGYC147-06|ROM 106663|Guyana.Upper Takutu-Upper Essequibo
Carollia perspicillata|ABGYE764-06|ROM 113363|Guyana.Upper Demerara-Berbice
Carollia perspicillata|ABGYG913-08|ROM 119341|Guyana
Carollia perspicillata|ABGYG934-08|ROM 119362|Guyana
Carollia perspicillata|ABSRA286-06|ROM 116875|Suriname.Nickerie
Carollia perspicillata|BCBNT659-06|ROM 113657|Guyana.Demerara-Mahaica
Carollia perspicillata|ABGYB283-06|ROM 102987|Guyana.Upper Takutu-Upper Essequibo
Carollia perspicillata|ABSA040-06|ROM 107939|Venezuela.Bolivar
Carollia perspicillata|ABSMS418-06|ROM 117580|Suriname.Sipaliwini
Carollia perspicillata|BCBNC183-06|ROM 116539|Guyana.Potaro-Siparuni
Carollia perspicillata|ABGYE008-06|ROM 109291|Guyana.Potaro-Siparuni
Carollia perspicillata|ABGYE354-06|ROM 111804|Guyana.Potaro-Siparuni
Carollia perspicillata|ABSMS469-06|ROM 117631|Suriname.Sipaliwini
Carollia perspicillata|ABGYG433-06|ROM 116568|Guyana.Potaro-Siparuni
Carollia perspicillata|ABGYA522-06|ROM 100189|Guyana.East Berbice-Corentyne
Carollia perspicillata|BCBN543-05|ROM 103373|Guyana.Upper Demerara-Berbice
Carollia perspicillata|BCBN322-05|ROM 100853|Guyana.Barima-Waini
Carollia perspicillata|BCBN143-05|ROM 98097|Guyana.Upper Takutu-Upper Essequibo
Carollia perspicillata|ABSRA289-06|ROM 116878|Suriname.Nickerie
Carollia perspicillata|ABGYF285-06|ROM 114640|Guyana.Potaro-Siparuni
Carollia perspicillata|ABGYE534-06|ROM 112007|Guyana.Potaro-Siparuni
Carollia perspicillata|ABGYE490-06|ROM 111962|Guyana.Potaro-Siparuni
[illegible]

Carollia perspicillata|ABGYF285-06|ROM 114640|Guyana.Potaro-Siparuni
Carollia perspicillata|ABGYE534-06|ROM 112007|Guyana.Potaro-Siparuni
Carollia perspicillata|ABGYE490-06|ROM 111962|Guyana.Potaro-Siparuni
Carollia perspicillata|ABGYE299-06|ROM 111749|Guyana.Potaro-Siparuni
Carollia perspicillata|ABGYE129-06|ROM 111572|Guyana.Potaro-Siparuni
Carollia perspicillata|ABGYE007-06|ROM 109290|Guyana.Potaro-Siparuni
Carollia perspicillata|ABGYD302-06|ROM 108641|Guyana.Potaro-Siparuni
Carollia perspicillata|ABGYC677-06|ROM F39731|Guyana.Cuyuni-Mazaruni
Carollia perspicillata|ABGYC553-06|ROM 107397|Guyana.Potaro-Siparuni
Carollia perspicillata|ABGYB709-06|ROM 103473|Guyana.Upper Demerara-Berbice
Carollia perspicillata|ABGYB528-06|ROM 103263|Guyana.Upper Takutu-Upper Essequibo
Carollia perspicillata|ABGYB254-06|ROM 102958|Guyana.Upper Takutu-Upper Essequibo
Carollia perspicillata|ABGYB216-06|ROM 102918|Guyana.Upper Takutu-Upper Essequibo
Carollia perspicillata|ABGYA667-06|ROM 100371|Guyana.East Berbice-Corentyne
Carollia perspicillata|ABGYA533-06|ROM 100200|Guyana.East Berbice-Corentyne
Carollia perspicillata|ABGYA521-06|ROM 100188|Guyana.East Berbice-Corentyne
Carollia perspicillata|ABGYA337-06|ROM 98736|Guyana.Barima-Waini
Carollia perspicillata|ABGYA327-06|ROM 98725|Guyana.Barima-Waini
Carollia perspicillata|ABGYA268-06|ROM 98168|Guyana.Potaro-Siparuni
Carollia perspicillata|ABGYA035-06|ROM 97825|Guyana.Upper Takutu-Upper Essequibo
Carollia perspicillata|ABGYA503-06|ROM 98914|Guyana.Barima-Waini
Carollia perspicillata|ABGYA355-06|ROM 98755|Guyana.Barima-Waini
Carollia perspicillata|ABGYA793-06|ROM 100846|Guyana.Barima-Waini
Carollia perspicillata|ABGYF008-06|ROM 113496|Guyana.Upper Takutu-Upper Essequibo
Carollia perspicillata|ABGYE075-06|ROM 111518|Guyana.Potaro-Siparuni
Carollia perspicillata|BCBNT015-06|ROM 106561|Guyana.Upper Takutu-Upper Essequibo
Carollia perspicillata|BCBNT879-06|ROM 115500|Guyana.Essequibo Islands-West Demerara
Carollia perspicillata|BCBNT910-06|ROM 115575|Guyana.Essequibo Islands-West Demerara
Carollia perspicillata|BCBNT979-06|ROM 114635|Guyana.Potaro-Siparuni
Carollia brevicauda|ABSCA063-06|ROM 104256|Panama
Carollia brevicauda|BCBN656-05|ROM 104273|Panama
Carollia brevicauda|ABSCA057-06|ROM 104244|Panama
Carollia brevicauda|ABSCA071-06|ROM 104274|Panama
Carollia brevicauda|BCBN710-05|ROM 104365|Panama.Darien
Carollia brevicauda|BCBN915-05|ROM 105789|Ecuador.Esmeraldas
Carollia brevicauda|BCBN919-05|ROM 105793|Ecuador.Esmeraldas
Carollia brevicauda|ABCMA561-06|ROM 103977|Ecuador.Napo
Carollia brevicauda|ABECA016-06|ROM F37082|Ecuador.Napo
Carollia brevicauda|ABECA886-06|ROM 106065|Ecuador.Napo
Carollia brevicauda|ABECA503-06|ROM 105536|Ecuador.Napo
Carollia brevicauda|ABECA898-06|ROM 106078|Ecuador.Napo
Carollia brevicauda|ABECA247-06|ROM F37423|Ecuador.Napo
Carollia brevicauda|ABECA695-06|ROM 105756|Ecuador.Napo
Carollia brevicauda|ABECA725-06|ROM 105866|Ecuador.Napo
Carollia brevicauda|ABECA727-06|ROM 105868|Ecuador.Napo
Carollia brevicauda|ABECA756-06|ROM 105907|Ecuador.Napo
Carollia brevicauda|ABECB002-06|ROM F40474|Ecuador.Napo
Carollia brevicauda|ABECB059-06|ROM F40536|Ecuador.Napo
Carollia brevicauda|ABECB087-08|ROM 118749|Ecuador
Carollia brevicauda|ABECA918-06|ROM 106098|Ecuador.Napo
Carollia brevicauda|ABECA001-06|ROM 103978|Ecuador.Napo
Carollia brevicauda|ABECA135-06|ROM F37271|Ecuador.Napo
Carollia brevicauda|ABECA249-06|ROM 105118|Ecuador.Napo
Carollia brevicauda|BCBN859-05|ROM 105611|Ecuador.Napo
Carollia brevicauda|BCBN939-05|ROM 105865|Ecuador.Napo
Carollia brevicauda|ABECA726-06|ROM 105867|Ecuador.Napo
Carollia brevicauda|ABECA375-06|ROM 105270|Ecuador.Napo
Carollia brevicauda|ABECA589-06|ROM 105621|Ecuador.Napo
Carollia brevicauda|ABECA678-06|ROM F37994|Ecuador.Napo
Carollia brevicauda|ABECA015-06|ROM F37081|Ecuador.Napo
Carollia brevicauda|ABECA472-06|ROM 105496|Ecuador.Napo
Carollia brevicauda|ABECA747-06|ROM 105895|Ecuador.Napo
Carollia brevicauda|ABECA248-06|ROM 105117|Ecuador.Napo
Carollia brevicauda|ABECA518-06|ROM F37787|Ecuador.Napo
Carollia brevicauda|ABECA897-06|ROM 106077|Ecuador.Napo
Carollia brevicauda|ABECA125-06|ROM F37257|Ecuador.Napo
Carollia brevicauda|ABECA515-06|ROM 105549|Ecuador.Napo
Carollia brevicauda|ABECA549-06|ROM 105578|Ecuador.Napo
Carollia brevicauda|ABECA008-06|ROM F37072|Ecuador.Napo
Carollia brevicauda|BCBN962-05|ROM 105941|Ecuador.Napo
Carollia brevicauda|ABECA516-06|ROM F37785|Ecuador.Napo
Carollia brevicauda|ABECA754-06|ROM 105905|Ecuador.Napo
Carollia brevicauda|ABECA126-06|ROM 104423|Ecuador.Napo
Carollia brevicauda|ABECA246-06|ROM 105116|Ecuador.Napo
Carollia brevicauda|ABECA262-06|ROM 105130|Ecuador.Napo
Carollia brevicauda|ABECA264-06|ROM 105132|Ecuador.Napo
Carollia brevicauda|BCBN831-05|ROM 105369|Ecuador.Napo
Carollia brevicauda|ABECA017-06|ROM 103996|Ecuador.Napo
Carollia brevicauda|BCBNT007-06|ROM 106362|Ecuador.Napo
Carollia brevicauda|BCBN909-05|ROM 105781|Ecuador.Esmeraldas
Carollia brevicauda|BCBN934-05|ROM 105811|Ecuador.Esmeraldas
Carollia brevicauda|ABSRA375-06|ROM 116964|Suriname.Nickerie
Carollia brevicauda|ABSRA421-06|ROM 117010|Suriname.Nickerie
Carollia brevicauda|ABSMS261-06|ROM 117423|Suriname.Sipaliwini
Carollia brevicauda|ABSMS376-06|ROM 117538|Suriname.Sipaliwini
Carollia brevicauda|ABSRA281-06|ROM 116870|Suriname.Nickerie
Carollia brevicauda|ABSRA464-06|ROM 117054|Suriname.Nickerie
Carollia brevicauda|ABSMS242-06|ROM 117404|Suriname.Sipaliwini
Carollia brevicauda|ABGYD397-06|ROM 108743|Guyana.Potaro-Siparuni
Carollia brevicauda|ABGYF283-06|ROM 114636|Guyana.Potaro-Siparuni
Carollia brevicauda|ABSMS243-06|ROM 117405|Suriname.Sipaliwini
Carollia brevicauda|ABSMS092-06|ROM 117254|Suriname.Sipaliwini
Carollia brevicauda|ABSRA266-06|ROM 116854|Suriname.Nickerie
Carollia brevicauda|ABSMS050-06|ROM 117212|Suriname.Sipaliwini
Carollia brevicauda|ABSRA332-06|ROM 116921|Suriname.Nickerie
Carollia brevicauda|ABGYD173-06|ROM 108494|Guyana.Potaro-Siparuni
Carollia brevicauda|ABSMS095-06|ROM 117257|Suriname.Sipaliwini
Carollia brevicauda|ABSRA351-06|ROM 116940|Suriname.Nickerie
Carollia brevicauda|ABSMS020-06|ROM 117182|Suriname.Sipaliwini
Carollia brevicauda|BCBNT774-06|ROM 113993|Suriname.Brokopondo
Carollia brevicauda|BCBN554-05|ROM 103521|Guyana.Upper Demerara-Berbice
Carollia brevicauda|ABGYG402-06|ROM 116530|Guyana.Potaro-Siparuni
Carollia brevicauda|ABGYF089-06|ROM 113620|Guyana.Demerara-Mahaica

Carollia brevicauda|BCBN554-05|ROM 103521|Guyana.Upper Demerara-Berbice
Carollia brevicauda|ABGYG402-06|ROM 116530|Guyana.Potaro-Siparuni
Carollia brevicauda|ABGYF089-06|ROM 113620|Guyana.Demerara-Mahaica
Carollia brevicauda|ABSRA306-06|ROM 116895|Suriname.Nickerie
Carollia brevicauda PS1|BCBNT518-06|ROM 111856|Guyana.Potaro-Siparuni
Carollia brevicauda PS1|BCBNC006-06|ROM 114692|Guyana.Potaro-Siparuni
Carollia brevicauda PS1|BCBNC040-06|ROM 114773|Guyana.Potaro-Siparuni
Carollia brevicauda|ABSRA475-06|ROM 117065|Suriname.Nickerie
Carollia brevicauda|ABSMS475-06|ROM 117637|Suriname.Sipaliwini
Carollia brevicauda|BCBNT743-06|ROM 113922|Suriname.Brokopondo
Carollia brevicauda|BCBNC083-06|ROM 115302|Guyana.Cuyuni-Mazaruni
Carollia brevicauda|BCBNC084-06|ROM 115303|Guyana.Cuyuni-Mazaruni
Carollia brevicauda|BCBNT749-06|ROM 113932|Suriname.Brokopondo
Carollia brevicauda|BCBNT781-06|ROM 114011|Suriname.Brokopondo
Carollia brevicauda|BCBNT780-06|ROM 114010|Suriname.Brokopondo
Carollia brevicauda|ABGYB381-06|ROM 103088|Guyana.Upper Takutu-Upper Essequibo
Carollia brevicauda PS1|BCBNC150-06|ROM 115872|Guyana.Potaro-Siparuni
Carollia brevicauda|ABGYD258-06|ROM 108594|Guyana.Potaro-Siparuni
Carollia brevicauda|ABGYE298-06|ROM 111748|Guyana.Potaro-Siparuni
Carollia brevicauda|ABGYE297-06|ROM 111747|Guyana.Potaro-Siparuni
Carollia brevicauda|ABGYD217-06|ROM 108548|Guyana.Potaro-Siparuni
Carollia brevicauda|ABGYD261-06|ROM 108597|Guyana.Potaro-Siparuni
Carollia brevicauda|ABGYD301-06|ROM 108640|Guyana.Potaro-Siparuni
Carollia brevicauda|ABGYD506-06|ROM 108864|Guyana.Potaro-Siparuni
Carollia brevicauda|ABGYF305-06|ROM 114674|Guyana.Potaro-Siparuni
Carollia brevicauda|BCBNC193-06|ROM 116616|Guyana.Potaro-Siparuni
Carollia brevicauda PS1|BCBNT968-06|ROM 115366|Guyana.Cuyuni-Mazaruni
Carollia brevicauda|BCBN542-05|ROM 103372|Guyana.Upper Demerara-Berbice
Carollia brevicauda|ABGYB058-06|ROM 101032|Guyana.Barima-Waini
Carollia brevicauda|ABGYD243-06|ROM 108575|Guyana.Potaro-Siparuni
Carollia brevicauda|ABGYD334-06|ROM 108674|Guyana.Potaro-Siparuni
Carollia brevicauda PS1|BCBNC021-06|ROM 114729|Guyana.Potaro-Siparuni
Carollia brevicauda PS1|BCBNT610-06|ROM 113513|Guyana.Upper Takutu-Upper Essequibo
Carollia brevicauda PS1|BCBN106-05|ROM 97812|Guyana.Upper Takutu-Upper Essequibo
Carollia brevicauda|ABSMS468-06|ROM 117630|Suriname.Sipaliwini
Carollia brevicauda|ABSMS419-06|ROM 117581|Suriname.Sipaliwini
Carollia brevicauda|ABSMS350-06|ROM 117512|Suriname.Sipaliwini
Carollia brevicauda|ABSMS006-06|ROM 117168|Suriname.Sipaliwini
Carollia brevicauda|ABGYG831-08|ROM 119259|Guyana
Carollia brevicauda|ABGYG1071-08|ROM 118995|Guyana
Carollia brevicauda|ABGYF309-06|ROM 114681|Guyana.Potaro-Siparuni
Carollia brevicauda|ABGYF286-06|ROM 114641|Guyana.Potaro-Siparuni
Carollia brevicauda|ABGYF277-06|ROM 114624|Guyana.Potaro-Siparuni
Carollia brevicauda|ABGYD259-06|ROM 108595|Guyana.Potaro-Siparuni
Carollia brevicauda|ABSMS170-06|ROM 117332|Suriname.Sipaliwini
Carollia brevicauda|ABSMS306-06|ROM 117468|Suriname.Sipaliwini
Carollia brevicauda|ABSRA453-06|ROM 117043|Suriname.Nickerie
Carollia brevicauda|ABSMS451-06|ROM 117613|Suriname.Sipaliwini
Carollia brevicauda|ABSRA316-06|ROM 116905|Suriname.Nickerie
Carollia brevicauda|ABSMS440-06|ROM 117602|Suriname.Sipaliwini
Carollia brevicauda|ABGYG791-08|ROM 119219|Guyana
Carollia brevicauda|ABSMS357-06|ROM 117519|Suriname.Sipaliwini
Carollia brevicauda|ABSMS067-06|ROM 117229|Suriname.Sipaliwini
Carollia brevicauda|ABGYG325-06|ROM 115953|Guyana.Potaro-Siparuni
Carollia brevicauda PS1|BCBNT635-06|ROM 113579|Guyana.Upper Takutu-Upper Essequibo
Carollia brevicauda PS1|BCBNT969-06|ROM 114616|Guyana.Potaro-Siparuni
Carollia brevicauda|ABSRA485-06|ROM 117075|Suriname.Nickerie
Carollia brevicauda|ABGYD257-06|ROM 108593|Guyana.Potaro-Siparuni
Carollia brevicauda|ABGYA344-06|ROM 98744|Guyana.Barima-Waini
Carollia brevicauda|ABSMS478-06|ROM 117640|Suriname.Sipaliwini
Carollia brevicauda|ABGYG331-06|ROM 115960|Guyana.Potaro-Siparuni
Carollia brevicauda|ABSMS033-06|ROM 117195|Suriname.Sipaliwini
Carollia brevicauda|ABGYG752-08|ROM 119180|Guyana
Carollia brevicauda|ABSRA315-06|ROM 116904|Suriname.Nickerie
Carollia brevicauda PS1|BCBNC042-06|ROM 114775|Guyana.Potaro-Siparuni
Carollia brevicauda PS1|BCBNC125-06|ROM 115780|Guyana.Potaro-Siparuni
Carollia brevicauda|BCBNT980-06|ROM 114638|Guyana.Potaro-Siparuni
Carollia brevicauda|ABGYB410-06|ROM 103117|Guyana.Upper Takutu-Upper Essequibo
Carollia brevicauda|ABGYA481-06|ROM 98891|Guyana.Barima-Waini
Carollia brevicauda|ABGYC787-06|ROM 108187|Guyana.Cuyuni-Mazaruni
Carollia brevicauda|ABGYF278-06|ROM 114625|Guyana.Potaro-Siparuni
Carollia brevicauda|ABGYF294-06|ROM 114657|Guyana.Potaro-Siparuni
Carollia brevicauda|ABGYB136-06|ROM 101118|Guyana.Barima-Waini
Carollia brevicauda|ABGYA766-06|ROM 100816|Guyana.Barima-Waini
Carollia brevicauda|ABGYA502-06|ROM 98913|Guyana.Barima-Waini
Carollia brevicauda|ABGYD260-06|ROM 108596|Guyana.Potaro-Siparuni
Carollia brevicauda|ABGYD366-06|ROM 108709|Guyana.Potaro-Siparuni
Carollia brevicauda|ABGYD436-06|ROM 108787|Guyana.Potaro-Siparuni
Carollia brevicauda|ABGYE254-06|ROM 111703|Guyana.Potaro-Siparuni
Carollia brevicauda|BCBNC025-06|ROM 114737|Guyana.Potaro-Siparuni
Carollia brevicauda|BCBNC195-06|ROM 116637|Guyana.Potaro-Siparuni
Carollia brevicauda|BCBNC199-06|ROM 116690|Guyana.Potaro-Siparuni
Carollia brevicauda|BCBNT240-06|ROM 108155|Guyana.Cuyuni-Mazaruni
Carollia brevicauda|BCBNT981-06|ROM 114639|Guyana.Potaro-Siparuni
Carollia brevicauda|ABGYF276-06|ROM 114623|Guyana.Potaro-Siparuni
Carollia brevicauda|ABGYB087-06|ROM 101064|Guyana.Barima-Waini
Carollia brevicauda|ABGYA448-06|ROM 98858|Guyana.Barima-Waini
Carollia brevicauda|BCBNC100-06|ROM 115349|Guyana.Cuyuni-Mazaruni
Carollia brevicauda|ABGYE719-06|ROM 112654|Guyana.Demerara-Mahaica
Carollia brevicauda|ABGYE741-06|ROM 112684|Guyana.Demerara-Mahaica
Carollia brevicauda PS1|BCBNT661-06|ROM 113661|Guyana.Demerara-Mahaica
Carollia brevicauda PS1|BCBNT082-06|ROM 107064|Guyana.Potaro-Siparuni
Carollia brevicauda PS1|BCBNT983-06|ROM 114644|Guyana.Potaro-Siparuni
Carollia brevicauda PS1|BCBNT982-06|ROM 114643|Guyana.Potaro-Siparuni
Carollia brevicauda PS1|BCBNC164-06|ROM 115952|Guyana.Potaro-Siparuni
Carollia brevicauda|ABGYD437-06|ROM 108788|Guyana.Potaro-Siparuni
Carollia brevicauda|ABGYD333-06|ROM 108673|Guyana.Potaro-Siparuni
Carollia brevicauda PS1|BCBNT984-06|ROM 114645|Guyana.Potaro-Siparuni
Carollia brevicauda|ABGYD349-06|ROM 108690|Guyana.Potaro-Siparuni
Carollia brevicauda|ABGYB126-06|ROM 101107|Guyana.Barima-Waini
Carollia brevicauda PS1|BCBN341-05|ROM 100937|Guyana.Barima-Waini
Carollia brevicauda PS1|BCBNT238-06|ROM 108147|Guyana.Cuyuni-Mazaruni
Carollia brevicauda|ABGYB124-06|ROM 101105|Guyana.Barima-Waini

Carollia brevicauda PS1|BCBN341-05|ROM 100937|Guyana.Barima-Waini
Carollia brevicauda PS1|BCBNT238-06|ROM 108147|Guyana.Cuyuni-Mazaruni
Carollia brevicauda|ABGYB124-06|ROM 101105|Guyana.Barima-Waini
Carollia brevicauda|ABGYA764-06|ROM 100814|Guyana.Barima-Waini
Carollia brevicauda|ABGYA310-06|ROM 98706|Guyana.Barima-Waini
Carollia brevicauda|ABGYB086-06|ROM 101063|Guyana.Barima-Waini
Carollia brevicauda|ABGYE357-06|ROM 111807|Guyana.Potaro-Siparuni
Carollia brevicauda|ABGYA360-06|ROM 98761|Guyana.Barima-Waini
Carollia brevicauda|ABGYD467-06|ROM 108818|Guyana.Potaro-Siparuni
Carollia brevicauda|ABGYE247-06|ROM 111696|Guyana.Potaro-Siparuni
Carollia brevicauda|BCBNC124-06|ROM 115779|Guyana.Potaro-Siparuni
Carollia brevicauda|BCBNT974-06|ROM 114627|Guyana.Potaro-Siparuni
Carollia brevicauda|ABGYA376-06|ROM 98780|Guyana.Barima-Waini
Carollia brevicauda PS1|BCBNC190-06|ROM 116584|Guyana.Potaro-Siparuni
Carollia brevicauda|ABGYE677-06|ROM 112604|Guyana.Demerara-Mahaica
Carollia brevicauda PS1|BCBNT673-06|ROM 113717|Guyana.Demerara-Mahaica
Carollia brevicauda|ABGYA788-06|ROM 100841|Guyana.Barima-Waini
Carollia brevicauda|ABGYA326-06|ROM 98724|Guyana.Barima-Waini
Carollia brevicauda|ABGYA345-06|ROM 98745|Guyana.Barima-Waini
Carollia brevicauda|ABGYB043-06|ROM 101015|Guyana.Barima-Waini
Carollia brevicauda|ABGYB085-06|ROM 101062|Guyana.Barima-Waini
Carollia brevicauda|ABGYB084-06|ROM 101061|Guyana.Barima-Waini
Carollia brevicauda PS1|BCBNT259-06|ROM 108216|Guyana.Cuyuni-Mazaruni
Carollia brevicauda|ABGYD172-06|ROM 108493|Guyana.Potaro-Siparuni
Carollia brevicauda|ABGYD215-06|ROM 108546|Guyana.Potaro-Siparuni
Carollia brevicauda|ABGYD242-06|ROM 108574|Guyana.Potaro-Siparuni
Carollia brevicauda|ABGYD332-06|ROM 108672|Guyana.Potaro-Siparuni
Carollia brevicauda|ABGYF284-06|ROM 114637|Guyana.Potaro-Siparuni
Carollia brevicauda|ABGYF287-06|ROM 114642|Guyana.Potaro-Siparuni
Carollia brevicauda|ABGYF293-06|ROM 114656|Guyana.Potaro-Siparuni
Carollia brevicauda|ABGYF303-06|ROM 114672|Guyana.Potaro-Siparuni
Carollia brevicauda|ABGYB123-06|ROM 101104|Guyana.Barima-Waini
Carollia brevicauda|ABGYB125-06|ROM 101106|Guyana.Barima-Waini
Carollia brevicauda|ABGYB127-06|ROM 101108|Guyana.Barima-Waini
Carollia brevicauda|ABGYB137-06|ROM 101119|Guyana.Barima-Waini
Carollia brevicauda|ABGYD847-06|ROM 109274|Guyana.Potaro-Siparuni
Carollia brevicauda|ABGYE014-06|ROM 109297|Guyana.Potaro-Siparuni
Carollia brevicauda|ABGYF304-06|ROM 114673|Guyana.Potaro-Siparuni
Carollia brevicauda|ABGYG562-06|ROM 116714|Guyana.Potaro-Siparuni
Carollia brevicauda PS1|BCBN330-05|ROM 100895|Guyana.Barima-Waini
Carollia brevicauda PS1|BCBN334-05|ROM 100913|Guyana.Barima-Waini
Carollia brevicauda PS1|BCBNC041-06|ROM 114774|Guyana.Potaro-Siparuni
Carollia brevicauda PS1|BCBNC113-06|ROM 115760|Guyana.Potaro-Siparuni
Carollia brevicauda|ABGYB001-06|ROM 100968|Guyana.Barima-Waini
Carollia brevicauda|ABGYB021-06|ROM 100992|Guyana.Barima-Waini
Carollia brevicauda PS1|BCBNC136-06|ROM 115805|Guyana.Potaro-Siparuni
Carollia brevicauda PS1|BCBNC140-06|ROM 115811|Guyana.Potaro-Siparuni
Carollia brevicauda PS1|BCBNC141-06|ROM 115812|Guyana.Potaro-Siparuni
Carollia brevicauda PS1|BCBNC163-06|ROM 115937|Guyana.Potaro-Siparuni
Carollia brevicauda PS1|BCBNC173-06|ROM 116000|Guyana.Potaro-Siparuni
Carollia brevicauda PS1|BCBNC184-06|ROM 116540|Guyana.Potaro-Siparuni
Carollia brevicauda PS1|BCBNT083-06|ROM 107065|Guyana.Potaro-Siparuni
Carollia brevicauda PS1|BCBNT965-06|ROM 114876|Guyana.Potaro-Siparuni
Carollia brevicauda|ABGYB041-06|ROM 101013|Guyana.Barima-Waini
Carollia brevicauda|ABGYB122-06|ROM 101103|Guyana.Barima-Waini
Carollia brevicauda|ABGYB035-06|ROM 101007|Guyana.Barima-Waini
Carollia brevicauda|ABGYB039-06|ROM 101011|Guyana.Barima-Waini
Carollia brevicauda|ABGYA865-06|ROM 100938|Guyana.Barima-Waini
Carollia brevicauda|ABGYA874-06|ROM 100950|Guyana.Barima-Waini
Carollia brevicauda|ABGYA807-06|ROM 100865|Guyana.Barima-Waini
Carollia brevicauda|ABGYA775-06|ROM 100825|Guyana.Barima-Waini
Carollia brevicauda|ABGYA504-06|ROM 98915|Guyana.Barima-Waini
Carollia brevicauda|ABGYA501-06|ROM 98912|Guyana.Barima-Waini
Carollia brevicauda|ABGYA476-06|ROM 98886|Guyana.Barima-Waini
Carollia brevicauda|ABGYA388-06|ROM 98792|Guyana.Barima-Waini
Carollia brevicauda|ABGYA386-06|ROM 98790|Guyana.Barima-Waini
Carollia brevicauda|ABGYA312-06|ROM 98708|Guyana.Barima-Waini
Carollia brevicauda|ABGYA336-06|ROM 98735|Guyana.Barima-Waini
Carollia brevicauda|ABGYB038-06|ROM 101010|Guyana.Barima-Waini
Carollia brevicauda PS1|BCBN331-05|ROM 100896|Guyana.Barima-Waini
Carollia brevicauda PS1|BCBN333-05|ROM 100912|Guyana.Barima-Waini
Carollia brevicauda|ABGYG569-06|ROM 116724|Guyana.Potaro-Siparuni
Carollia brevicauda PS1|BCBN551-05|ROM 103429|Guyana.Upper Demerara-Berbice
Carollia brevicauda|ABSMS010-06|ROM 117172|Suriname.Sipaliwini
Carollia brevicauda PS1|BCBNC165-06|ROM 115959|Guyana.Potaro-Siparuni
Carollia brevicauda PS1|BCBNT249-06|ROM 108186|Guyana.Cuyuni-Mazaruni
Carollia brevicauda PS1|BCBNT988-06|ROM 114654|Guyana.Potaro-Siparuni
Lonchophylla robusta|BCBN651-05|ROM 104268|Panama
Lonchophylla chocoana|BCBN912-05|ROM 105786|Ecuador.Esmeraldas
Phylloderma stenops|BCBN624-05|ROM 104225|Panama.Canal Zone
Phylloderma stenops PS2|BCBN172-05|ROM 98903|Guyana.Barima-Waini
Phylloderma stenops|ABGYA230-06|ROM 98106|Guyana.Upper Takutu-Upper Essequibo
Phylloderma stenops|ABGYC152-06|ROM 106673|Guyana.Upper Takutu-Upper Essequibo
Phylloderma stenops|ABGYD112-06|ROM 108410|Guyana.Potaro-Siparuni
Phylloderma stenops|ABGYD200-06|ROM 108529|Guyana.Potaro-Siparuni
Phylloderma stenops|ABGYE695-06|ROM 112628|Guyana.Demerara-Mahaica
Phylloderma stenops|ABGYG561-06|ROM 116713|Guyana.Potaro-Siparuni
Phylloderma stenops|ABGYG909-08|ROM 119337|Guyana
Phylloderma stenops PS2|BCBN770-05|ROM 104694|Guyana.Potaro-Siparuni
Phylloderma stenops PS2|BCBNT035-06|ROM 106627|Guyana.Upper Takutu-Upper Essequibo
Phylloderma stenops|ABGYE036-06|ROM 109322|Guyana.Potaro-Siparuni
Phylloderma stenops|ABGYG125-06|ROM 115679|Guyana.Potaro-Siparuni
Phylloderma stenops|ABGYC642-06|ROM F39660|Guyana.Potaro-Siparuni
Phylloderma stenops|ABGYD565-06|ROM 108937|Guyana.Potaro-Siparuni
Phylloderma stenops PS2|BCBNT109-06|ROM 107203|Guyana.Potaro-Siparuni
Phylloderma stenops|ABECA693-06|ROM F40043|Ecuador.Napo
Phylloderma stenops|BCBN789-05|ROM 105186|Ecuador.Napo
Phylloderma stenops|BCBN898-05|ROM 105749|Ecuador.Napo
Phylloderma stenops|ABSMS349-06|ROM 117511|Suriname.Sipaliwini
Phylloderma stenops|ABSRA473-06|ROM 117063|Suriname.Nickerie
Phylloderma stenops|ABSMS016-06|ROM 117178|Suriname.Sipaliwini
Phylloderma stenops|ABGYC051-06|ROM 104828|Guyana.Potaro-Siparuni
Phylloderma stenops|ABGYE086-06|ROM 111529|Guyana.Potaro-Siparuni

Phylloderma stenops|ABSMS016-06|ROM 117178|Suriname.Sipaliwini
Phylloderma stenops|ABGYC051-06|ROM 104828|Guyana.Potaro-Siparuni
Phylloderma stenops|ABGYE086-06|ROM 111529|Guyana.Potaro-Siparuni
Phylloderma stenops PS1|BCBNT510-06|ROM 111506|Guyana.Potaro-Siparuni
Rhinophylla fischerae|ABECA285-06|ROM 105156|Ecuador.Napo
Rhinophylla fischerae|ABECA047-06|ROM F37132|Ecuador.Napo
Rhinophylla fischerae|ABECA097-06|ROM 104382|Ecuador.Napo
Rhinophylla fischerae|ABECA521-06|ROM F37790|Ecuador.Napo
Rhinophylla fischerae|ABECA409-06|ROM 105309|Ecuador.Napo
Rhinophylla fischerae|ABECA690-06|ROM 105752|Ecuador.Napo
Rhinophylla fischerae|ABECA547-06|ROM 105576|Ecuador.Napo
Rhinophylla fischerae|ABECB085-08|ROM 118747|Ecuador
Rhinophylla fischerae|BCBN573-05|ROM 104025|Ecuador.Napo
Rhinophylla fischerae|BCBN948-05|ROM 105902|Ecuador.Napo
Rhinophylla fischerae|ABECA881-06|ROM 106060|Ecuador.Napo
Rhinophylla fischerae|ABECA127-06|ROM F37260|Ecuador.Napo
Rhinophylla fischerae|ABECA096-06|ROM 104381|Ecuador.Napo
Rhinophylla fischerae|ABECA400-06|ROM 105296|Ecuador.Napo
Rhinophylla fischerae|ABECA824-06|ROM F40246|Ecuador.Napo
Rhinophylla fischerae|ABECA475-06|ROM 105498|Ecuador.Napo
Rhinophylla fischerae|BCBN560-05|ROM 103992|Ecuador.Napo
Rhinophylla fischerae|ABECA880-06|ROM F40314|Ecuador.Napo
Rhinophylla fischerae|ABECA314-06|ROM 105194|Ecuador.Napo
Rhinophylla fischerae|BCBN714-05|ROM 104375|Ecuador.Napo
Rhinophylla fischerae|ABECA882-06|ROM 106061|Ecuador.Napo
Rhinophylla fischerae|BCBN715-05|ROM 104376|Ecuador.Napo
Rhinophylla fischerae|ABECA048-06|ROM F37133|Ecuador.Napo
Rhinophylla fischerae|ABECA050-06|ROM F37136|Ecuador.Napo
Rhinophylla fischerae|ABECA629-06|ROM 105675|Ecuador.Napo
Rhinophylla fischerae|BCBN856-05|ROM 105606|Ecuador.Napo
Rhinophylla fischerae|ABECA399-06|ROM F37621|Ecuador.Napo
Rhinophylla fischerae|ABECA548-06|ROM 105577|Ecuador.Napo
Rhinophylla fischerae|ABECA883-06|ROM 106062|Ecuador.Napo
Rhinophylla fischerae|ABECB086-08|ROM 118748|Ecuador
Rhinophylla fischerae|ABECA128-06|ROM 104425|Ecuador.Napo
Rhinophylla fischerae|ABECA730-06|ROM 105874|Ecuador.Napo
Rhinophylla fischerae|ABECA304-06|ROM 105177|Ecuador.Napo
Rhinophylla fischerae|ABECA852-06|ROM 106027|Ecuador.Napo
Rhinophylla fischerae|BCBN561-05|ROM 103993|Ecuador.Napo
Rhinophylla fischerae|BCBN797-05|ROM 105210|Ecuador.Napo
Rhinophylla fischerae|BCBN942-05|ROM 105873|Ecuador.Napo
Rhinophylla fischerae|ABECA482-06|ROM 105506|Ecuador.Napo
Rhinophylla fischerae|BCBN959-05|ROM 105935|Ecuador.Napo
Lonchorhina orinocensis|BCBNT171-06|ROM 107845|Venezuela.Amazonas
Lonchorhina orinocensis|BCBNT152-06|ROM 107813|Venezuela.Amazonas
Lonchorhina orinocensis|BCBNT153-06|ROM 107814|Venezuela.Amazonas
Lonchorhina orinocensis|BCBNT154-06|ROM 107815|Venezuela.Amazonas
Lonchorhina orinocensis|BCBNT167-06|ROM 107841|Venezuela.Amazonas
Lonchorhina orinocensis|BCBNT170-06|ROM 107844|Venezuela.Amazonas
Lonchorhina orinocensis|BCBNT180-06|ROM 107868|Venezuela.Amazonas
Lonchorhina orinocensis|BCBNT187-06|ROM 107886|Venezuela.Amazonas
Lonchorhina orinocensis|BCBNT205-06|ROM 107911|Venezuela.Bolivar
Lonchorhina orinocensis|BCBNT206-06|ROM 107912|Venezuela.Bolivar
Macrophyllum macrophyllum|ABSCA033-06|ROM F38027|Panama.Canal Zone
Macrophyllum macrophyllum|BCBN604-05|ROM 104199|Panama.Canal Zone
Macrophyllum macrophyllum|BCBN605-05|ROM 104200|Panama.Canal Zone
Macrophyllum macrophyllum|ABECA100-06|ROM F37220|Ecuador.Napo
Macrophyllum macrophyllum|BCBN578-05|ROM 104032|Ecuador.Napo
Macrophyllum macrophyllum|BCBN579-05|ROM 104033|Ecuador.Napo
Macrophyllum macrophyllum|BCBN721-05|ROM 104388|Ecuador.Napo
Macrophyllum macrophyllum|BCBN722-05|ROM 104389|Ecuador.Napo
Macrophyllum macrophyllum|ABGYC129-06|ROM 106641|Guyana.Upper Takutu-Upper Essequibo
Macrophyllum macrophyllum|BCBNT017-06|ROM 106563|Guyana.Upper Takutu-Upper Essequibo
Macrophyllum macrophyllum|BCBNT018-06|ROM 106564|Guyana.Upper Takutu-Upper Essequibo
Macrophyllum macrophyllum|BCBNT066-06|ROM 106765|Guyana.Upper Takutu-Upper Essequibo
Macrophyllum macrophyllum|ABGYB867-06|ROM 104711|Guyana.Potaro-Siparuni
Macrophyllum macrophyllum|ABGYE589-06|ROM 112068|Guyana.Potaro-Siparuni
Macrophyllum macrophyllum|ABGYG158-06|ROM 115718|Guyana.Potaro-Siparuni
Macrophyllum macrophyllum|ABSMS217-06|ROM 117379|Suriname.Sipaliwini
Macrophyllum macrophyllum|BCBN771-05|ROM 104698|Guyana.Potaro-Siparuni
Macrophyllum macrophyllum|BCBNT513-06|ROM 111632|Guyana.Potaro-Siparuni
Anoura caudifer|BCBN907-05|ROM 105779|Ecuador.Esmeraldas
Anoura caudifer|BCBN913-05|ROM 105787|Ecuador.Esmeraldas
Anoura caudifer|BCBN920-05|ROM 105794|Ecuador.Esmeraldas
Anoura latidens|ABGYF655-06|ROM 115323|Guyana.Cuyuni-Mazaruni
Anoura latidens|BCBNC102-06|ROM 115351|Guyana.Cuyuni-Mazaruni
Anoura latidens|BCBNC158-06|ROM 115925|Guyana.Potaro-Siparuni
Anoura latidens|BCBNC087-06|ROM 115318|Guyana.Cuyuni-Mazaruni
Anoura latidens|BCBNC086-06|ROM 115317|Guyana.Cuyuni-Mazaruni
Anoura latidens|BCBNT232-06|ROM 108111|Guyana.Cuyuni-Mazaruni
Anoura geoffroyi|BCBN274-05|ROM 99831|Guatemala.Zacapa
Anoura geoffroyi|BCBN275-05|ROM 99832|Guatemala.Zacapa
Anoura geoffroyi|BCBN456-05|ROM 101463|El Salvador.Santa Ana
Anoura geoffroyi|BCBN457-05|ROM 101464|El Salvador.Santa Ana
Anoura geoffroyi|BCBN465-05|ROM 101527|El Salvador.Santa Ana
Anoura geoffroyi|BCBN674-05|ROM 104299|Panama.Chiriqui
Anoura geoffroyi|ABGYG395-06|ROM 116522|Guyana.Potaro-Siparuni
Anoura geoffroyi|ABGYG464-06|ROM 116602|Guyana.Potaro-Siparuni
Anoura geoffroyi|ABGYG504-06|ROM 116646|Guyana.Potaro-Siparuni
Anoura geoffroyi|ABGYG525-06|ROM 116667|Guyana.Potaro-Siparuni
Anoura geoffroyi|ABGYG527-06|ROM 116669|Guyana.Potaro-Siparuni
Anoura geoffroyi|ABGYG528-06|ROM 116670|Guyana.Potaro-Siparuni
Anoura geoffroyi|ABGYG529-06|ROM 116672|Guyana.Potaro-Siparuni
Anoura geoffroyi|ABGYG547-06|ROM 116693|Guyana.Potaro-Siparuni
Anoura geoffroyi|ABGYF656-06|ROM 115324|Guyana.Cuyuni-Mazaruni
Anoura geoffroyi|BCBNC035-06|ROM 114766|Guyana.Potaro-Siparuni
Anoura geoffroyi|ABGYF669-06|ROM 115353|Guyana.Cuyuni-Mazaruni
Anoura geoffroyi|BCBNC066-06|ROM 115171|Guyana.Cuyuni-Mazaruni
Anoura geoffroyi|BCBNC099-06|ROM 115348|Guyana.Cuyuni-Mazaruni
Anoura geoffroyi|ABSMS321-06|ROM 117483|Suriname.Sipaliwini
Anoura geoffroyi|ABGYG447-06|ROM 116583|Guyana.Potaro-Siparuni
Anoura geoffroyi|ABGYG499-06|ROM 116641|Guyana.Potaro-Siparuni
[illegible]

Anoura geoffroyi|ABSMS321-06|ROM 117483|Suriname.Sipaliwini
Anoura geoffroyi|ABGYG447-06|ROM 116583|Guyana.Potaro-Siparuni
Anoura geoffroyi|ABGYG499-06|ROM 116641|Guyana.Potaro-Siparuni
Anoura geoffroyi|ABGYG546-06|ROM 116692|Guyana.Potaro-Siparuni
Anoura geoffroyi|BCBNC203-06|ROM 116708|Guyana.Potaro-Siparuni
Anoura geoffroyi|BCBNC208-06|ROM 116723|Guyana.Potaro-Siparuni
Anoura geoffroyi|BCBNC091-06|ROM 115329|Guyana.Cuyuni-Mazaruni
Anoura geoffroyi|BCBNT038-06|ROM 106660|Guyana.Upper Takutu-Upper Essequibo
Anoura geoffroyi|BCBNC082-06|ROM 115301|Guyana.Cuyuni-Mazaruni
Anoura geoffroyi|BCBNT054-06|ROM 106706|Guyana.Upper Takutu-Upper Essequibo
Anoura geoffroyi|BCBNT055-06|ROM 106707|Guyana.Upper Takutu-Upper Essequibo
Anoura geoffroyi|BCBNT057-06|ROM 106717|Guyana.Upper Takutu-Upper Essequibo
Anoura geoffroyi|BCBNC089-06|ROM 115327|Guyana.Cuyuni-Mazaruni
Anoura geoffroyi|ABGYG526-06|ROM 116668|Guyana.Potaro-Siparuni
Anoura geoffroyi|ABGYG524-06|ROM 116666|Guyana.Potaro-Siparuni
Anoura geoffroyi|ABGYG445-06|ROM 116581|Guyana.Potaro-Siparuni
Anoura geoffroyi|ABGYF601-06|ROM 115260|Guyana.Cuyuni-Mazaruni
Anoura geoffroyi|ABGYG563-06|ROM 116716|Guyana.Potaro-Siparuni
Anoura geoffroyi|BCBNC090-06|ROM 115328|Guyana.Cuyuni-Mazaruni
Anoura geoffroyi|BCBNT176-06|ROM 107853|Venezuela.Amazonas
Anoura geoffroyi|ABGYF612-06|ROM 115274|Guyana.Cuyuni-Mazaruni
Anoura geoffroyi|BCBNC104-06|ROM 115361|Guyana.Cuyuni-Mazaruni
Anoura geoffroyi|ABGYF651-06|ROM 115319|Guyana.Cuyuni-Mazaruni
Anoura geoffroyi|BCBNC175-06|ROM 116492|Guyana.Potaro-Siparuni
Anoura geoffroyi|ABGYF599-06|ROM 115258|Guyana.Cuyuni-Mazaruni
Anoura geoffroyi|ABGYG477-06|ROM 116617|Guyana.Potaro-Siparuni
Anoura geoffroyi|BCBNC206-06|ROM 116715|Guyana.Potaro-Siparuni
Anoura geoffroyi|BCBNT849-06|ROM 114218|Suriname.Brokopondo
Anoura geoffroyi|ABGYF356-06|ROM 114772|Guyana.Potaro-Siparuni
Anoura geoffroyi|BCBNC024-06|ROM 114736|Guyana.Potaro-Siparuni
Anoura geoffroyi|BCBNC215-06|ROM 116734|Guyana.Potaro-Siparuni
Anoura geoffroyi|BCBNC217-06|ROM 116746|Guyana.Potaro-Siparuni
Anoura geoffroyi|BCBNC023-06|ROM 114735|Guyana.Potaro-Siparuni
Anoura geoffroyi|ABSMS369-06|ROM 117531|Suriname.Sipaliwini
Anoura geoffroyi|ABGYF653-06|ROM 115321|Guyana.Cuyuni-Mazaruni
Anoura geoffroyi|ABGYF654-06|ROM 115322|Guyana.Cuyuni-Mazaruni
Anoura geoffroyi|ABGYG520-06|ROM 116662|Guyana.Potaro-Siparuni
Anoura geoffroyi|BCBNC077-06|ROM 115256|Guyana.Cuyuni-Mazaruni
Anoura geoffroyi|ABGYF602-06|ROM 115261|Guyana.Cuyuni-Mazaruni
Anoura geoffroyi|ABGYF652-06|ROM 115320|Guyana.Cuyuni-Mazaruni
Anoura geoffroyi|BCBNC103-06|ROM 115352|Guyana.Cuyuni-Mazaruni
Anoura geoffroyi|BCBNT322-06|ROM 108828|Guyana.Potaro-Siparuni
Anoura geoffroyi|ABSMS281-06|ROM 117443|Suriname.Sipaliwini
Anoura geoffroyi|BCBNC092-06|ROM 115330|Guyana.Cuyuni-Mazaruni
Anoura geoffroyi|BCBNT854-06|ROM 114225|Suriname.Brokopondo
Anoura geoffroyi|BCBNC036-06|ROM 114767|Guyana.Potaro-Siparuni
Anoura geoffroyi|ABGYG558-06|ROM 116707|Guyana.Potaro-Siparuni
Anoura geoffroyi|BCBNT519-06|ROM 111865|Guyana.Potaro-Siparuni
Anoura geoffroyi|ABGYG428-06|ROM 116563|Guyana.Potaro-Siparuni
Anoura geoffroyi|ABGYF600-06|ROM 115259|Guyana.Cuyuni-Mazaruni
Anoura geoffroyi|ABGYF611-06|ROM 115273|Guyana.Cuyuni-Mazaruni
Anoura geoffroyi|ABGYG483-06|ROM 116623|Guyana.Potaro-Siparuni
Anoura geoffroyi|BCBNC034-06|ROM 114765|Guyana.Potaro-Siparuni
Anoura geoffroyi|BCBNC057-06|ROM 115088|Guyana.Cuyuni-Mazaruni
Anoura geoffroyi|BCBNC073-06|ROM 115236|Guyana.Cuyuni-Mazaruni
Anoura geoffroyi|BCBNC088-06|ROM 115326|Guyana.Cuyuni-Mazaruni
Anoura geoffroyi|BCBNC093-06|ROM 115331|Guyana.Cuyuni-Mazaruni
Anoura geoffroyi|BCBNT964-06|ROM 114874|Guyana.Potaro-Siparuni
Anoura cultrata|BCBN921-05|ROM 105795|Ecuador.Esmeraldas
Anoura caudifer|BCBNC037-06|ROM 114768|Guyana.Potaro-Siparuni
Anoura caudifer|BCBN572-05|ROM 104024|Ecuador.Napo
Anoura caudifer|BCBN781-05|ROM 105163|Ecuador.Napo
Anoura caudifer|ABECA929-06|ROM 106113|Ecuador.Napo
Anoura caudifer|ABECA150-06|ROM 104454|Ecuador.Napo
Anoura caudifer|ABECB049-06|ROM 106353|Ecuador.Napo
Anoura caudifer|BCBN773-05|ROM 105122|Ecuador.Napo
Anoura caudifer|BCBN835-05|ROM 105499|Ecuador.Napo
Anoura caudifer|ABECA476-06|ROM F37728|Ecuador.Napo
Anoura caudifer|ABECB115-08|ROM 118777|Ecuador
Anoura caudifer|BCBN945-05|ROM 105890|Ecuador.Napo
Anoura caudifer|BCBN958-05|ROM 105934|Ecuador.Napo
Anoura caudifer|BCBNT813-06|ROM 114123|Suriname.Brokopondo
Anoura caudifer|BCBNT026-06|ROM 106589|Guyana.Upper Takutu-Upper Essequibo
Anoura caudifer|BCBNT025-06|ROM 106588|Guyana.Upper Takutu-Upper Essequibo
Anoura caudifer|BCBNT024-06|ROM 106587|Guyana.Upper Takutu-Upper Essequibo
Anoura caudifer|BCBNT027-06|ROM 106590|Guyana.Upper Takutu-Upper Essequibo
Anoura caudifer|ABGYF289-06|ROM 114649|Guyana.Potaro-Siparuni
Anoura caudifer|ABGYF378-06|ROM 114801|Guyana.Potaro-Siparuni
Anoura caudifer|ABGYF335-06|ROM 114734|Guyana.Potaro-Siparuni
Anoura caudifer|ABGYF300-06|ROM 114669|Guyana.Potaro-Siparuni
Anoura caudifer|BCBNC039-06|ROM 114770|Guyana.Potaro-Siparuni
Anoura caudifer|BCBNC038-06|ROM 114769|Guyana.Potaro-Siparuni
Anoura caudifer|BCBNC031-06|ROM 114756|Guyana.Potaro-Siparuni
Anoura caudifer|BCBNC030-06|ROM 114755|Guyana.Potaro-Siparuni
Anoura caudifer|ABGYF357-06|ROM 114776|Guyana.Potaro-Siparuni
Anoura caudifer|ABGYF355-06|ROM 114771|Guyana.Potaro-Siparuni
Anoura caudifer|ABGYF348-06|ROM 114754|Guyana.Potaro-Siparuni
Anoura caudifer|ABGYF291-06|ROM 114651|Guyana.Potaro-Siparuni
Anoura caudifer|ABGYF290-06|ROM 114650|Guyana.Potaro-Siparuni
Anoura caudifer|ABGYF274-06|ROM 114618|Guyana.Potaro-Siparuni
Anoura caudifer|BCBNC146-06|ROM 115865|Guyana.Potaro-Siparuni
Anoura caudifer|BCBNT761-06|ROM 113962|Suriname.Brokopondo
Anoura caudifer|BCBNC145-06|ROM 115864|Guyana.Potaro-Siparuni
Anoura caudifer|BCBNC167-06|ROM 115962|Guyana.Potaro-Siparuni
Anoura caudifer|BCBNC094-06|ROM 115343|Guyana.Cuyuni-Mazaruni
Anoura caudifer|BCBNC105-06|ROM 115362|Guyana.Cuyuni-Mazaruni
Anoura caudifer|BCBNC098-06|ROM 115347|Guyana.Cuyuni-Mazaruni
Anoura caudifer|BCBNC097-06|ROM 115346|Guyana.Cuyuni-Mazaruni
Anoura caudifer|BCBNC096-06|ROM 115345|Guyana.Cuyuni-Mazaruni
Anoura caudifer|BCBNC081-06|ROM 115272|Guyana.Cuyuni-Mazaruni
Anoura caudifer|BCBNC076-06|ROM 115255|Guyana.Cuyuni-Mazaruni
Anoura caudifer|ABGYF668-06|ROM 115342|Guyana.Cuyuni-Mazaruni
Anoura caudifer|ABGYF667-06|ROM 115341|Guyana.Cuyuni-Mazaruni
[illegible]

Anoura caudifer|BCBNC076-06|ROM 115255|Guyana.Cuyuni-Mazaruni
Anoura caudifer|ABGYF668-06|ROM 115342|Guyana.Cuyuni-Mazaruni
Anoura caudifer|ABGYF667-06|ROM 115341|Guyana.Cuyuni-Mazaruni
Anoura caudifer|ABGYF658-06|ROM 115332|Guyana.Cuyuni-Mazaruni
Anoura caudifer|ABGYF637-06|ROM 115299|Guyana.Cuyuni-Mazaruni
Anoura caudifer|ABGYF636-06|ROM 115298|Guyana.Cuyuni-Mazaruni
Anoura caudifer|ABGYF635-06|ROM 115297|Guyana.Cuyuni-Mazaruni
Anoura caudifer|BCBNC095-06|ROM 115344|Guyana.Cuyuni-Mazaruni
Anoura caudifer|BCBNC070-06|ROM 115210|Guyana.Cuyuni-Mazaruni
Anoura caudifer|ABGYF638-06|ROM 115300|Guyana.Cuyuni-Mazaruni
Anoura caudifer|BCBNT967-06|ROM 115363|Guyana.Cuyuni-Mazaruni
Thyroptera lavali|BCBN574-05|ROM 104026|Ecuador.Napo
Thyroptera lavali|BCBN799-05|ROM 105215|Ecuador.Napo
Thyroptera lavali|BCBN869-05|ROM 105667|Ecuador.Napo
Thyroptera tricolor|ABSCA139-06|ROM F44044|Costa Rica.Limon
Thyroptera tricolor|BCBNT433-06|ROM 108311|Costa Rica.Limon
Thyroptera tricolor|BCBNT434-06|ROM 108312|Costa Rica.Limon
Thyroptera tricolor|BCBNT435-06|ROM 108313|Costa Rica.Limon
Thyroptera tricolor|ABECB156-08|ROM 118834|Ecuador
Thyroptera tricolor|ABECB158-08|ROM 118836|Ecuador
Thyroptera tricolor|BCBN753-05|ROM 104477|Ecuador.Napo
Thyroptera tricolor|BCBN988-05|ROM 106160|Ecuador.Napo
Thyroptera tricolor|BCBN857-05|ROM 105609|Ecuador.Napo
Thyroptera tricolor|BCBN993-05|ROM 106318|Ecuador.Napo
Thyroptera tricolor|ABSMS406-06|ROM 117568|Suriname.Sipaliwini
Thyroptera tricolor|ABSMS407-06|ROM 117569|Suriname.Sipaliwini
Thyroptera tricolor|ABSMS454-06|ROM 117616|Suriname.Sipaliwini
Thyroptera tricolor|ABSMS474-06|ROM 117636|Suriname.Sipaliwini
Thyroptera tricolor|ABGYA284-06|ROM 98199|Guyana.Demerara-Mahaica
Thyroptera tricolor|ABGYA285-06|ROM 98200|Guyana.Demerara-Mahaica
Thyroptera tricolor|ABGYA286-06|ROM 98201|Guyana.Demerara-Mahaica
Thyroptera tricolor|ABGYA287-06|ROM 98203|Guyana.Demerara-Mahaica
Thyroptera tricolor|ABGYB142-06|ROM 101129|Guyana.Barima-Waini
Thyroptera tricolor|BCBN358-05|ROM 101079|Guyana.Barima-Waini
Thyroptera tricolor|BCBNT300-06|ROM 108524|Guyana.Potaro-Siparuni
Thyroptera tricolor|BCBNT757-06|ROM 113952|Suriname.Brokopondo
Thyroptera tricolor|BCBNT758-06|ROM 113953|Suriname.Brokopondo
Thyroptera tricolor|BCBNT745-06|ROM 113925|Suriname.Brokopondo
Thyroptera tricolor|BCBNT756-06|ROM 113951|Suriname.Brokopondo
Thyroptera tricolor|BCBNT759-06|ROM 113956|Suriname.Brokopondo
Diphylla ecaudata|BCBN008-05|ROM 95706|Mexico.Campeche
Diphylla ecaudata|BCBN013-05|ROM 95776|Mexico.Campeche
Diphylla ecaudata|BCBN813-05|ROM 105272|Ecuador.Napo
Furipterus horrens|ABSMS460-06|ROM 117622|Suriname.Sipaliwini
Furipterus horrens|ABSMS421-06|ROM 117583|Suriname.Sipaliwini
Furipterus horrens|BCBN279-05|ROM 100202|Guyana.East Berbice-Corentyne
Furipterus horrens|BCBN592-05|ROM 104058|Ecuador.Napo
Noctilio leporinus|BCBNC218-06|ROM 97682|Mexico.Chiapas
Noctilio leporinus|ABMXC659-07|ROM FN33163|Mexico.Chiapas
Noctilio leporinus|BCBNC219-06|ROM 97683|Mexico.Chiapas
Noctilio leporinus|BCBN163-05|ROM 98757|Guyana.Barima-Waini
Noctilio leporinus|ABSCA037-06|ROM F38038|Panama.Canal Zone
Noctilio leporinus|BCBN611-05|ROM 104207|Panama.Canal Zone
Noctilio leporinus|ABGYC211-06|ROM 106752|Guyana.Upper Takutu-Upper Essequibo
Noctilio leporinus|ABSRA508-06|ROM 117098|Suriname.Nickerie
Noctilio leporinus|ABGYC099-06|ROM 106606|Guyana.Upper Takutu-Upper Essequibo
Noctilio leporinus|BCBNT037-06|ROM 106658|Guyana.Upper Takutu-Upper Essequibo
Noctilio leporinus|ABGYC201-06|ROM 106740|Guyana.Upper Takutu-Upper Essequibo
Noctilio leporinus|ABGYF211-06|ROM 113782|Guyana.Demerara-Mahaica
Noctilio leporinus|ABGYE709-06|ROM 112644|Guyana.Demerara-Mahaica
Noctilio leporinus|ABGYF215-06|ROM 113786|Guyana.Demerara-Mahaica
Noctilio leporinus|ABGYC104-06|ROM 106612|Guyana.Upper Takutu-Upper Essequibo
Noctilio leporinus|ABGYC228-06|ROM 106774|Guyana.Upper Takutu-Upper Essequibo
Noctilio leporinus|ABGYE755-06|ROM 112701|Guyana.Demerara-Mahaica
Noctilio leporinus|ABGYF214-06|ROM 113785|Guyana.Demerara-Mahaica
Noctilio leporinus|ABGYC066-06|ROM 106553|Guyana.Upper Takutu-Upper Essequibo
Noctilio leporinus|ABGYE056-06|ROM 109342|Guyana.Potaro-Siparuni
Noctilio leporinus|ABGYE708-06|ROM 112643|Guyana.Demerara-Mahaica
Noctilio leporinus|ABGYE834-06|ROM 113446|Guyana.Upper Demerara-Berbice
Noctilio leporinus|ABGYE835-06|ROM 113447|Guyana.Upper Demerara-Berbice
Noctilio leporinus|ABGYF128-06|ROM 113675|Guyana.Demerara-Mahaica
Noctilio leporinus|ABGYF216-06|ROM 113787|Guyana.Demerara-Mahaica
Noctilio leporinus|ABGYF252-06|ROM 113837|Guyana.Demerara-Mahaica
Noctilio leporinus|ABGYF212-06|ROM 113783|Guyana.Demerara-Mahaica
Noctilio leporinus|ABGYE767-06|ROM 113367|Guyana.Upper Demerara-Berbice
Noctilio leporinus|BCBNT042-06|ROM 106671|Guyana.Upper Takutu-Upper Essequibo
Noctilio leporinus|BCBNT059-06|ROM 106727|Guyana.Upper Takutu-Upper Essequibo
Noctilio leporinus|ABGYF213-06|ROM 113784|Guyana.Demerara-Mahaica
Noctilio leporinus|BCBNT047-06|ROM 106689|Guyana.Upper Takutu-Upper Essequibo
Noctilio leporinus|BCBNT069-06|ROM 106775|Guyana.Upper Takutu-Upper Essequibo
Noctilio albiventris PS1|ABGYE560-06|ROM 112033|Guyana.Potaro-Siparuni
Noctilio albiventris PS1|ABGYD637-06|ROM 109040|Guyana.Potaro-Siparuni
Noctilio albiventris PS1|BCBNT115-06|ROM 107254|Guyana.Potaro-Siparuni
Noctilio albiventris PS1|BCBNT368-06|ROM 109032|Guyana.Potaro-Siparuni
Noctilio albiventris PS1|BCBNT369-06|ROM 109033|Guyana.Potaro-Siparuni
Noctilio albiventris PS1|ABGYC429-06|ROM 107251|Guyana.Potaro-Siparuni
Noctilio albiventris PS1|ABGYD636-06|ROM 109039|Guyana.Potaro-Siparuni
Noctilio albiventris PS1|ABGYE556-06|ROM 112029|Guyana.Potaro-Siparuni
Noctilio albiventris PS1|ABGYG100-06|ROM 115650|Guyana.Potaro-Siparuni
Noctilio albiventris PS1|ABGYG133-06|ROM 115691|Guyana.Potaro-Siparuni
Noctilio albiventris PS1|ABGYG134-06|ROM 115692|Guyana.Potaro-Siparuni
Noctilio albiventris PS1|ABGYA231-06|ROM 98108|Guyana.Upper Takutu-Upper Essequibo
Noctilio albiventris PS1|ABGYD634-06|ROM 109037|Guyana.Potaro-Siparuni
Noctilio albiventris PS1|ABGYE523-06|ROM 111995|Guyana.Potaro-Siparuni
Noctilio albiventris PS1|ABGYE555-06|ROM 112028|Guyana.Potaro-Siparuni
Noctilio albiventris PS1|ABGYG101-06|ROM 115651|Guyana.Potaro-Siparuni
Noctilio albiventris PS1|ABGYG102-06|ROM 115652|Guyana.Potaro-Siparuni
Noctilio albiventris PS1|BCBNT371-06|ROM 109035|Guyana.Potaro-Siparuni
Noctilio albiventris PS2|ABGYG162-06|ROM 115729|Guyana.Potaro-Siparuni
Noctilio albiventris PS2|ABGYG666-08|ROM 119094|Guyana
Noctilio albiventris PS2|ABGYG667-08|ROM 119095|Guyana
Noctilio albiventris PS2|BCBNT114-06|ROM 107253|Guyana.Potaro-Siparuni
Noctilio albiventris PS2|ABGYG688-08|ROM 119116|Guyana

Noctilio albiventris PS2|ABGYG667-08|ROM 119095|Guyana
Noctilio albiventris PS2|BCBNT114-06|ROM 107253|Guyana.Potaro-Siparuni
Noctilio albiventris PS2|ABGYG688-08|ROM 119116|Guyana
Noctilio albiventris PS2|ABGYG716-08|ROM 119144|Guyana
Noctilio albiventris PS2|ABGYG597-08|ROM 119025|Guyana
Noctilio albiventris PS2|ABGYG959-08|ROM 119387|Guyana
Noctilio albiventris PS2|BCBN133-05|ROM 97928|Guyana.Upper Takutu-Upper Essequibo
Noctilio albiventris PS2|ABGYG686-08|ROM 119114|Guyana
Noctilio albiventris PS2|ABGYG600-08|ROM 119028|Guyana
Noctilio albiventris PS2|ABGYG598-08|ROM 119026|Guyana
Noctilio albiventris PS2|ABGYE559-06|ROM 112032|Guyana.Potaro-Siparuni
Noctilio albiventris PS2|ABGYE558-06|ROM 112031|Guyana.Potaro-Siparuni
Noctilio albiventris PS2|ABGYE557-06|ROM 112030|Guyana.Potaro-Siparuni
Noctilio albiventris PS2|ABGYE511-06|ROM 111983|Guyana.Potaro-Siparuni
Noctilio albiventris PS2|ABGYC430-06|ROM 107252|Guyana.Potaro-Siparuni
Noctilio albiventris PS2|ABGYE512-06|ROM 111984|Guyana.Potaro-Siparuni
Noctilio albiventris PS2|ABGYG637-08|ROM 119065|Guyana
Noctilio albiventris PS2|ABGYG638-08|ROM 119066|Guyana
Noctilio albiventris PS2|ABGYG639-08|ROM 119067|Guyana
Noctilio albiventris PS2|ABGYG687-08|ROM 119115|Guyana
Noctilio albiventris PS2|ABGYG717-08|ROM 119145|Guyana
Noctilio albiventris PS2|BCBN147-05|ROM 98107|Guyana.Upper Takutu-Upper Essequibo
Noctilio albiventris PS2|ABGYD635-06|ROM 109038|Guyana.Potaro-Siparuni
Noctilio albiventris PS2|ABGYE522-06|ROM 111994|Guyana.Potaro-Siparuni
Noctilio albiventris PS2|ABGYG121-06|ROM 115675|Guyana.Potaro-Siparuni
Noctilio albiventris PS2|BCBNT370-06|ROM 109034|Guyana.Potaro-Siparuni
Noctilio albiventris PS2|BCBNT372-06|ROM 109036|Guyana.Potaro-Siparuni
Noctilio albiventris PS2|BCBNT686-06|ROM 113781|Guyana.Demerara-Mahaica
Diaemus youngi|BCBNT010-06|ROM 106551|Guyana.Upper Takutu-Upper Essequibo
Diaemus youngi|BCBNC137-06|ROM 115806|Guyana.Potaro-Siparuni
Diaemus youngi|ABSRA374-06|ROM 116963|Suriname.Nickerie
Diaemus youngi|BCBNT683-06|ROM 113765|Guyana.Demerara-Mahaica
Desmodus rotundus|ABMXC218-06|ROM 96220|Mexico.Tabasco
Desmodus rotundus|ABMXC654-06|ROM 96221|Mexico.Tabasco
Desmodus rotundus|BCBN025-05|ROM 96219|Mexico.Tabasco
Desmodus rotundus|ABMXC536-06|ROM 97462|Mexico.Quintana Roo
Desmodus rotundus|BCBN492-05|ROM 97463|Mexico.Quintana Roo
Desmodus rotundus|BCBN461-05|ROM 101472|El Salvador.Santa Ana
Desmodus rotundus|BCBN460-05|ROM 101471|El Salvador.Santa Ana
Desmodus rotundus|BCBN092-05|ROM 98484|Guatemala.El Progreso
Desmodus rotundus|BCBN219-05|ROM 99615|Guatemala.Peten
Desmodus rotundus|BCBN491-05|ROM 97461|Mexico.Quintana Roo
Desmodus rotundus|BCBN493-05|ROM 97464|Mexico.Quintana Roo
Desmodus rotundus|BCBNT415-06|ROM 108295|Costa Rica.Limon
Desmodus rotundus|BCBNT437-06|ROM 108315|Costa Rica.Limon
Desmodus rotundus|BCBNT453-06|ROM 108331|Costa Rica.Limon
Desmodus rotundus|BCBNT454-06|ROM 108332|Costa Rica.Limon
Desmodus rotundus|ABSCA068-06|ROM F38108|Panama
Desmodus rotundus|BCBN649-05|ROM 104266|Panama
Desmodus rotundus|ABECA374-06|ROM 105268|Ecuador.Napo
Desmodus rotundus|ABECA377-06|ROM 105271|Ecuador.Napo
Desmodus rotundus|ABECA465-06|ROM 105377|Ecuador.Napo
Desmodus rotundus|ABECA581-06|ROM 105613|Ecuador.Napo
Desmodus rotundus|BCBN883-05|ROM 105696|Ecuador.Napo
Desmodus rotundus|ABECA112-06|ROM 104405|Ecuador.Napo
Desmodus rotundus|ABECA649-06|ROM 105703|Ecuador.Napo
Desmodus rotundus|ABECA648-06|ROM F37950|Ecuador.Napo
Desmodus rotundus|ABECA111-06|ROM 104404|Ecuador.Napo
Desmodus rotundus|BCBN726-05|ROM 104403|Ecuador.Napo
Desmodus rotundus|ABECA650-06|ROM 105704|Ecuador.Napo
Desmodus rotundus|BCBN809-05|ROM 105246|Ecuador.Napo
Desmodus rotundus|BCBN966-05|ROM 105972|Ecuador.Napo
Desmodus rotundus|ABSA057-06|ROM 111060|Brazil.Sao Paulo
Desmodus rotundus|ABSA058-06|ROM 111061|Brazil.Sao Paulo
Desmodus rotundus|ABSA065-06|ROM 111079|Brazil.Sao Paulo
Desmodus rotundus|ABSA074-06|ROM 111092|Brazil.Sao Paulo
Desmodus rotundus|BCBNT457-06|ROM 111051|Brazil.Sao Paulo
Desmodus rotundus|BCBNT462-06|ROM 111058|Brazil.Sao Paulo
Desmodus rotundus|BCBNT472-06|ROM 111073|Brazil.Sao Paulo
Desmodus rotundus|BCBNT473-06|ROM 111074|Brazil.Sao Paulo
Desmodus rotundus|ABGYB079-06|ROM 101056|Guyana.Barima-Waini
Desmodus rotundus|BCBNT164-06|ROM 107838|Venezuela.Amazonas
Desmodus rotundus|BCBNT165-06|ROM 107839|Venezuela.Amazonas
Desmodus rotundus|BCBNT667-06|ROM 113682|Guyana.Demerara-Mahaica
Desmodus rotundus|ABGYC238-06|ROM 106791|Guyana.Upper Takutu-Upper Essequibo
Desmodus rotundus|ABGYG849-08|ROM 119277|Guyana
Desmodus rotundus|ABGYG903-08|ROM 119331|Guyana
Desmodus rotundus|ABGYD028-06|ROM 108250|Guyana.Cuyuni-Mazaruni
Desmodus rotundus|ABGYA713-06|ROM 100418|Guyana.East Berbice-Corentyne
Desmodus rotundus|ABGYE181-06|ROM 111624|Guyana.Potaro-Siparuni
Desmodus rotundus|ABGYA166-06|ROM 98025|Guyana.Potaro-Siparuni
Desmodus rotundus|ABGYB604-06|ROM 103350|Guyana.Upper Demerara-Berbice
Desmodus rotundus|ABGYB605-06|ROM 103351|Guyana.Upper Demerara-Berbice
Desmodus rotundus|ABGYB700-06|ROM 103464|Guyana.Upper Demerara-Berbice
Desmodus rotundus|ABGYB743-06|ROM 103510|Guyana.Upper Demerara-Berbice
Desmodus rotundus|ABGYB856-06|ROM 104700|Guyana.Potaro-Siparuni
Desmodus rotundus|ABGYB926-06|ROM 104771|Guyana.Potaro-Siparuni
Desmodus rotundus|ABGYC585-06|ROM 107440|Guyana.Potaro-Siparuni
Desmodus rotundus|ABGYE261-06|ROM 111710|Guyana.Potaro-Siparuni
Desmodus rotundus|ABGYF133-06|ROM 113684|Guyana.Demerara-Mahaica
Desmodus rotundus|ABGYB713-06|ROM 103478|Guyana.Upper Demerara-Berbice
Desmodus rotundus|ABGYB742-06|ROM 103509|Guyana.Upper Demerara-Berbice
Desmodus rotundus|ABGYF270-06|ROM 114877|Guyana.Potaro-Siparuni
Desmodus rotundus|BCBNT556-06|ROM 112547|Guyana.Demerara-Mahaica
Desmodus rotundus|BCBNT668-06|ROM 113683|Guyana.Demerara-Mahaica
Desmodus rotundus|BCBNT672-06|ROM 113715|Guyana.Demerara-Mahaica
Desmodus rotundus|BCBNT692-06|ROM 113809|Guyana.Demerara-Mahaica
Desmodus rotundus|BCBNT882-06|ROM 115510|Guyana.Essequibo Islands-West Demerara
Desmodus rotundus|ABGYG607-08|ROM 119035|Guyana
Desmodus rotundus|ABGYG608-08|ROM 119036|Guyana
Desmodus rotundus|ABGYG653-08|ROM 119081|Guyana
Desmodus rotundus|ABGYG654-08|ROM 119082|Guyana
Desmodus rotundus|ABGYG656-08|ROM 119084|Guyana

[illegible]
Desmodus rotundus|ABGYG653-08|ROM 119081|Guyana
Desmodus rotundus|ABGYG654-08|ROM 119082|Guyana
Desmodus rotundus|ABGYG656-08|ROM 119084|Guyana
Desmodus rotundus|ABGYG657-08|ROM 119085|Guyana
Desmodus rotundus|ABGYG658-08|ROM 119086|Guyana
Desmodus rotundus|ABGYG694-08|ROM 119122|Guyana
Desmodus rotundus|ABGYG695-08|ROM 119123|Guyana
Desmodus rotundus|ABGYG696-08|ROM 119124|Guyana
Desmodus rotundus|ABGYG697-08|ROM 119125|Guyana
Desmodus rotundus|ABGYG856-08|ROM 119284|Guyana
Desmodus rotundus|ABGYG904-08|ROM 119332|Guyana
Desmodus rotundus|ABGYG941-08|ROM 119369|Guyana
Desmodus rotundus|ABGYG636-08|ROM 119064|Guyana
Desmodus rotundus|ABGYG652-08|ROM 119080|Guyana
Desmodus rotundus|ABGYG634-08|ROM 119062|Guyana
Desmodus rotundus|ABGYG635-08|ROM 119063|Guyana
Desmodus rotundus|ABGYG632-08|ROM 119060|Guyana
Desmodus rotundus|ABGYG633-08|ROM 119061|Guyana
Desmodus rotundus|ABGYG605-08|ROM 119033|Guyana
Desmodus rotundus|ABGYG606-08|ROM 119034|Guyana
Desmodus rotundus|ABGYG444-06|ROM 116580|Guyana.Potaro-Siparuni
Desmodus rotundus|ABGYG560-06|ROM 116711|Guyana.Potaro-Siparuni
Desmodus rotundus|ABGYG434-06|ROM 116569|Guyana.Potaro-Siparuni
Desmodus rotundus|ABGYG1034-08|ROM 118958|Guyana
Desmodus rotundus|ABGYG1006-08|ROM 118930|Guyana
Desmodus rotundus|ABGYF017-06|ROM 113508|Guyana.Upper Takutu-Upper Essequibo
Desmodus rotundus|ABGYC241-06|ROM 106794|Guyana.Upper Takutu-Upper Essequibo
Desmodus rotundus|ABGYC239-06|ROM 106792|Guyana.Upper Takutu-Upper Essequibo
Desmodus rotundus|ABGYC181-06|ROM 106715|Guyana.Upper Takutu-Upper Essequibo
Desmodus rotundus|ABGYA235-06|ROM 98113|Guyana.Upper Takutu-Upper Essequibo
Desmodus rotundus|ABGYA234-06|ROM 98112|Guyana.Upper Takutu-Upper Essequibo
Desmodus rotundus|ABGYA045-06|ROM 97836|Guyana.Upper Takutu-Upper Essequibo
Desmodus rotundus|ABSMS136-06|ROM 117298|Suriname.Sipaliwini
Desmodus rotundus|ABGYA707-06|ROM 100412|Guyana.East Berbice-Corentyne
Desmodus rotundus|ABGYE846-06|ROM 113460|Guyana.Upper Takutu-Upper Essequibo
Desmodus rotundus|ABGYG382-06|ROM 116505|Guyana.Potaro-Siparuni
Desmodus rotundus|ABSRA347-06|ROM 116936|Suriname.Nickerie
Desmodus rotundus|ABSRA413-06|ROM 117002|Suriname.Nickerie
Desmodus rotundus|BCBNT883-06|ROM 115511|Guyana.Essequibo Islands-West Demerara
Vampyrum spectrum|BCBN940-05|ROM 105870|Ecuador.Napo
Vampyrum spectrum|BCBN941-05|ROM 105871|Ecuador.Napo
Vampyrum spectrum|ABSMS201-06|ROM 117363|Suriname.Sipaliwini
Vampyrum spectrum|BCBNC027-06|ROM 114740|Guyana.Potaro-Siparuni
Vampyrum spectrum|BCBNT127-06|ROM 107352|Guyana.Potaro-Siparuni
Chrotopterus auritus|BCBNT464-06|ROM 111063|Brazil.Sao Paulo
Chrotopterus auritus|BCBNT469-06|ROM 111070|Brazil.Sao Paulo
Chrotopterus auritus|BCBNT478-06|ROM 111085|Brazil.Sao Paulo
Chrotopterus auritus|ABMXA928-06|ROM FN33837|Mexico.Quintana Roo
Chrotopterus auritus|BCBN028-05|ROM 96319|Mexico.Campeche
Chrotopterus auritus|BCBN029-05|ROM 96377|Mexico.Campeche
Chrotopterus auritus|BCBN225-05|ROM 99491|Guatemala.Peten
Chrotopterus auritus|BCBN599-05|ROM 104074|Ecuador.Napo
Chrotopterus auritus|BCBN946-05|ROM 105893|Ecuador.Napo
Chrotopterus auritus|ABGYE816-06|ROM 113420|Guyana.Upper Demerara-Berbice
Chrotopterus auritus|ABGYE539-06|ROM 112012|Guyana.Potaro-Siparuni
Chrotopterus auritus|ABGYE540-06|ROM 112013|Guyana.Potaro-Siparuni
Chrotopterus auritus|ABGYE758-06|ROM 113357|Guyana.Upper Demerara-Berbice
Chrotopterus auritus|BCBN540-05|ROM 103361|Guyana.Upper Demerara-Berbice
Chrotopterus auritus|ABGYE168-06|ROM 111611|Guyana.Potaro-Siparuni
Chrotopterus auritus|ABGYD754-06|ROM 109172|Guyana.Potaro-Siparuni
Chrotopterus auritus|ABGYD064-06|ROM 108360|Guyana.Potaro-Siparuni
Chrotopterus auritus|ABGYC641-06|ROM F39658|Guyana.Potaro-Siparuni
Chrotopterus auritus|ABGYC582-06|ROM 107431|Guyana.Potaro-Siparuni
Chrotopterus auritus|ABGYB610-06|ROM 103360|Guyana.Upper Demerara-Berbice
Chrotopterus auritus|ABGYA147-06|ROM 97992|Guyana.Potaro-Siparuni
Chrotopterus auritus|ABGYA706-06|ROM 100411|Guyana.East Berbice-Corentyne
Chrotopterus auritus|ABGYE169-06|ROM 111612|Guyana.Potaro-Siparuni
Chrotopterus auritus|BCBNT591-06|ROM 113421|Guyana.Upper Demerara-Berbice
Chrotopterus auritus|ABGYD753-06|ROM 109171|Guyana.Potaro-Siparuni
Chrotopterus auritus|ABGYG1017-08|ROM 118941|Guyana
Chrotopterus auritus|ABGYE119-06|ROM 111562|Guyana.Potaro-Siparuni
Chrotopterus auritus|ABGYF010-06|ROM 113500|Guyana.Upper Takutu-Upper Essequibo
Chrotopterus auritus|ABGYF023-06|ROM 113518|Guyana.Upper Takutu-Upper Essequibo
Chrotopterus auritus|ABGYC516-06|ROM 107353|Guyana.Potaro-Siparuni
Chrotopterus auritus|ABGYE295-06|ROM 111745|Guyana.Potaro-Siparuni
Chrotopterus auritus|ABGYE507-06|ROM 111979|Guyana.Potaro-Siparuni
Chrotopterus auritus|ABGYF269-06|ROM 114875|Guyana.Potaro-Siparuni
Chrotopterus auritus|ABRMM065-07|ROM F39079|Guyana.Potaro-Siparuni
Chrotopterus auritus|BCBNT587-06|ROM 113369|Guyana.Upper Demerara-Berbice
Chrotopterus auritus|BCBNT763-06|ROM 113966|Suriname.Brokopondo
Chrotopterus auritus|ABGYD106-06|ROM 108404|Guyana.Potaro-Siparuni
Chrotopterus auritus|ABGYD557-06|ROM 108927|Guyana.Potaro-Siparuni
Chrotopterus auritus|ABGYD749-06|ROM 109167|Guyana.Potaro-Siparuni
Chrotopterus auritus|ABGYE225-06|ROM 111673|Guyana.Potaro-Siparuni
Chrotopterus auritus|ABGYC768-06|ROM 108170|Guyana.Cuyuni-Mazaruni
Chrotopterus auritus|ABGYC612-06|ROM F39078|Guyana.Potaro-Siparuni
Chrotopterus auritus|ABGYC593-06|ROM 107449|Guyana.Potaro-Siparuni
Chrotopterus auritus|ABGYC385-06|ROM 107202|Guyana.Potaro-Siparuni
Chrotopterus auritus|ABGYC041-06|ROM 104818|Guyana.Potaro-Siparuni
Chrotopterus auritus|ABGYC016-06|ROM 104793|Guyana.Potaro-Siparuni
Chrotopterus auritus|ABGYB474-06|ROM 103209|Guyana.Upper Takutu-Upper Essequibo
Chrotopterus auritus|ABGYA162-06|ROM 98021|Guyana.Potaro-Siparuni
Chrotopterus auritus|ABGYD183-06|ROM 108509|Guyana.Potaro-Siparuni
Chrotopterus auritus|BCBNT806-06|ROM 114103|Suriname.Brokopondo
Chrotopterus auritus|BCBNT592-06|ROM 113422|Guyana.Upper Demerara-Berbice
Chrotopterus auritus|ABGYB690-06|ROM 103454|Guyana.Upper Demerara-Berbice
Chrotopterus auritus|ABGYB706-06|ROM 103470|Guyana.Upper Demerara-Berbice
Chrotopterus auritus|ABGYB712-06|ROM 103477|Guyana.Upper Demerara-Berbice
Chrotopterus auritus|BCBNT597-06|ROM 113443|Guyana.Upper Demerara-Berbice
Chrotopterus auritus|ABGYB659-06|ROM 103417|Guyana.Upper Demerara-Berbice
Chrotopterus auritus|ABGYE355-06|ROM 111805|Guyana.Potaro-Siparuni
Chrotopterus auritus|ABGYF024-06|ROM 113519|Guyana.Upper Takutu-Upper Essequibo
Chrotopterus auritus|ABGYF030-06|ROM 113527|Guyana.Upper Takutu-Upper Essequibo

[illegible]
Chrotopterus auritus|ABGYE355-06|ROM 111805|Guyana.Potaro-Siparuni
Chrotopterus auritus|ABGYF024-06|ROM 113519|Guyana.Upper Takutu-Upper Essequibo
Chrotopterus auritus|ABGYF030-06|ROM 113527|Guyana.Upper Takutu-Upper Essequibo
Chrotopterus auritus|ABGYF075-06|ROM 113602|Guyana.Upper Takutu-Upper Essequibo
Chrotopterus auritus|ABGYF076-06|ROM 113603|Guyana.Upper Takutu-Upper Essequibo
Chrotopterus auritus|ABGYG1016-08|ROM 118940|Guyana
Chrotopterus auritus|ABSMS090-06|ROM 117252|Suriname.Sipaliwini
Chrotopterus auritus|BCBNT922-06|ROM 115602|Guyana.Essequibo Islands-West Demerara
Pteronotus parnellii|ABCSA212-06|ROM 98483|Guatemala.El Progreso
Pteronotus parnellii|BCBN452-05|ROM 101459|El Salvador.Santa Ana
Pteronotus parnellii|ABCSA459-06|ROM 99610|Guatemala.Peten
Pteronotus parnellii|ABCSA511-06|ROM 99522|Guatemala.Peten
Pteronotus parnellii|ABCSA757-06|ROM 101360|El Salvador.Santa Ana
Pteronotus parnellii|ABCSA795-06|ROM 101400|El Salvador.Santa Ana
Pteronotus parnellii|BCBN180-05|ROM 99235|Guatemala.Peten
Pteronotus parnellii|BCBN202-05|ROM 99389|Guatemala.Peten
Pteronotus parnellii|BCBN463-05|ROM 101476|El Salvador.Santa Ana
Pteronotus parnellii|ABCSA759-06|ROM 101362|El Salvador.Santa Ana
Pteronotus parnellii|BCBN444-05|ROM 101424|El Salvador.Santa Ana
Pteronotus parnellii|ABCSA498-06|ROM 99496|Guatemala.Peten
Pteronotus parnellii|ABCSA758-06|ROM 101361|El Salvador.Santa Ana
Pteronotus parnellii|BCBN010-05|ROM 95741|Mexico.Campeche
Pteronotus parnellii|ABCSA376-06|ROM 99390|Guatemala.Peten
Pteronotus parnellii|ABCSA460-06|ROM 99611|Guatemala.Peten
Pteronotus parnellii|ABCSA497-06|ROM 99495|Guatemala.Peten
Pteronotus parnellii|BCBN222-05|ROM 99475|Guatemala.Peten
Pteronotus parnellii|BCBN228-05|ROM 99494|Guatemala.Peten
Pteronotus parnellii|BCBN437-05|ROM 101401|El Salvador.Santa Ana
Pteronotus parnellii|BCBN179-05|ROM 99234|Guatemala.Peten
Pteronotus parnellii|BCBN445-05|ROM 101425|El Salvador.Santa Ana
Pteronotus parnellii|BCBN446-05|ROM 101426|El Salvador.Santa Ana
Pteronotus parnellii|ABCSA760-06|ROM 101363|El Salvador.Santa Ana
Pteronotus parnellii|BCBN447-05|ROM 101427|El Salvador.Santa Ana
Pteronotus parnellii|BCBN453-05|ROM 101460|El Salvador.Santa Ana
Pteronotus parnellii|BCBN459-05|ROM 101470|El Salvador.Santa Ana
Pteronotus parnellii|ABSCA117-06|ROM 104355|Panama.Darien
Pteronotus parnellii|ABSCA040-06|ROM F38044|Panama.Canal Zone
Pteronotus parnellii|ABSCA066-06|ROM 104263|Panama
Pteronotus parnellii|ABSCA067-06|ROM 104264|Panama
Pteronotus parnellii|ABSCA085-06|ROM 104304|Panama.Chiriqui
Pteronotus parnellii|ABSCA125-06|ROM 104369|Panama.Darien
Pteronotus parnellii|BCBN610-05|ROM 104205|Panama.Canal Zone
Pteronotus parnellii|BCBN625-05|ROM 104227|Panama.Canal Zone
Pteronotus parnellii|BCBN647-05|ROM 104262|Panama
Pteronotus parnellii|BCBN678-05|ROM 104303|Panama.Chiriqui
Pteronotus parnellii|BCBN688-05|ROM 104328|Panama.Chiriqui
Pteronotus parnellii|ABSA008-06|ROM 107854|Venezuela
Pteronotus parnellii|ABSA009-06|ROM 107855|Venezuela
Pteronotus parnellii|ABSA013-06|ROM 107861|Venezuela
Pteronotus parnellii|ABSA010-06|ROM 107856|Venezuela
Pteronotus parnellii|BCBNT178-06|ROM 107858|Venezuela.Amazonas
Pteronotus parnellii|ABGYC800-06|ROM 108199|Guyana.Cuyuni-Mazaruni
Pteronotus parnellii|ABSA014-06|ROM 107862|Venezuela
Pteronotus parnellii|ABGYD017-06|ROM F43287|Guyana.Cuyuni-Mazaruni
Pteronotus parnellii|ABGYD012-06|ROM 108235|Guyana.Cuyuni-Mazaruni
Pteronotus parnellii|ABGYC811-06|ROM 108211|Guyana.Cuyuni-Mazaruni
Pteronotus parnellii|ABGYC810-06|ROM 108210|Guyana.Cuyuni-Mazaruni
Pteronotus parnellii|ABGYC701-06|ROM 108101|Guyana.Cuyuni-Mazaruni
Pteronotus parnellii|ABGYB070-06|ROM 101046|Guyana.Barima-Waini
Pteronotus parnellii|ABGYA872-06|ROM 100948|Guyana.Barima-Waini
Pteronotus parnellii|ABGYA812-06|ROM 100871|Guyana.Barima-Waini
Pteronotus parnellii|ABGYA801-06|ROM 100858|Guyana.Barima-Waini
Pteronotus parnellii|ABSA012-06|ROM 107860|Venezuela
Pteronotus parnellii|ABSA015-06|ROM 107863|Venezuela
Pteronotus parnellii|BCBNT214-06|ROM 107924|Venezuela.Bolivar
Pteronotus parnellii|BCBNT177-06|ROM 107857|Venezuela.Amazonas
Pteronotus parnellii|ABSA011-06|ROM 107859|Venezuela
Pteronotus parnellii|BCBNT219-06|ROM 107934|Venezuela.Bolivar
Pteronotus parnellii|BCBNT225-06|ROM 107943|Venezuela.Bolivar
Pteronotus parnellii|BCBNT340-06|ROM 108934|Guyana.Potaro-Siparuni
Pteronotus parnellii|ABSMS446-06|ROM 117608|Suriname.Sipaliwini
Pteronotus parnellii|ABGYA680-06|ROM 100385|Guyana.East Berbice-Corentyne
Pteronotus parnellii|ABGYA689-06|ROM 100394|Guyana.East Berbice-Corentyne
Pteronotus parnellii|ABGYE538-06|ROM 112011|Guyana.Potaro-Siparuni
Pteronotus parnellii|ABGYE563-06|ROM 112037|Guyana.Potaro-Siparuni
Pteronotus parnellii|ABGYE784-06|ROM 113384|Guyana.Upper Demerara-Berbice
Pteronotus parnellii|ABGYG010-06|ROM 115489|Guyana.Essequibo Islands-West Demerara
Pteronotus parnellii|ABSMS205-06|ROM 117367|Suriname.Sipaliwini
Pteronotus parnellii|ABSRA354-06|ROM 116943|Suriname.Nickerie
Pteronotus parnellii|ABGYC394-06|ROM 107213|Guyana.Potaro-Siparuni
Pteronotus parnellii|ABGYC409-06|ROM 107231|Guyana.Potaro-Siparuni
Pteronotus parnellii|ABGYG185-06|ROM 115750|Guyana.Potaro-Siparuni
Pteronotus parnellii|ABGYB727-06|ROM 103493|Guyana.Upper Demerara-Berbice
Pteronotus parnellii|ABGYB480-06|ROM 103215|Guyana.Upper Takutu-Upper Essequibo
Pteronotus parnellii|ABGYA694-06|ROM 100399|Guyana.East Berbice-Corentyne
Pteronotus parnellii|ABGYA724-06|ROM 100429|Guyana.East Berbice-Corentyne
Pteronotus parnellii|ABGYA725-06|ROM 100430|Guyana.East Berbice-Corentyne
Pteronotus parnellii|ABGYF210-06|ROM 113780|Guyana.Demerara-Mahaica
Pteronotus parnellii|ABGYE794-06|ROM 113395|Guyana.Upper Demerara-Berbice
Pteronotus parnellii|ABGYE792-06|ROM 113393|Guyana.Upper Demerara-Berbice
Pteronotus parnellii|ABGYE723-06|ROM 112658|Guyana.Demerara-Mahaica
Pteronotus parnellii|ABGYB194-06|ROM 102896|Guyana.Upper Takutu-Upper Essequibo
Pteronotus parnellii|ABGYA685-06|ROM 100390|Guyana.East Berbice-Corentyne
Pteronotus parnellii|ABGYA686-06|ROM 100391|Guyana.East Berbice-Corentyne
Pteronotus parnellii|ABGYA690-06|ROM 100395|Guyana.East Berbice-Corentyne
Pteronotus parnellii|ABGYA691-06|ROM 100396|Guyana.East Berbice-Corentyne
Pteronotus parnellii|ABGYA692-06|ROM 100397|Guyana.East Berbice-Corentyne
Pteronotus parnellii|ABGYA693-06|ROM 100398|Guyana.East Berbice-Corentyne
Pteronotus parnellii|ABGYA695-06|ROM 100400|Guyana.East Berbice-Corentyne
Pteronotus parnellii|ABGYA696-06|ROM 100401|Guyana.East Berbice-Corentyne
Pteronotus parnellii|ABGYA712-06|ROM 100417|Guyana.East Berbice-Corentyne
Pteronotus parnellii|ABGYA726-06|ROM 100431|Guyana.East Berbice-Corentyne
[illegible]

Pteronotus parnellii|ABGYA696-06|ROM 100401|Guyana.East Berbice-Corentyne
Pteronotus parnellii|ABGYA712-06|ROM 100417|Guyana.East Berbice-Corentyne
Pteronotus parnellii|ABGYA726-06|ROM 100431|Guyana.East Berbice-Corentyne
Pteronotus parnellii|ABGYB192-06|ROM 102894|Guyana.Upper Takutu-Upper Essequibo
Pteronotus parnellii|ABGYB193-06|ROM 102895|Guyana.Upper Takutu-Upper Essequibo
Pteronotus parnellii|ABGYB227-06|ROM 102929|Guyana.Upper Takutu-Upper Essequibo
Pteronotus parnellii|ABGYB286-06|ROM 102990|Guyana.Upper Takutu-Upper Essequibo
Pteronotus parnellii|ABGYB287-06|ROM 102991|Guyana.Upper Takutu-Upper Essequibo
Pteronotus parnellii|ABGYB301-06|ROM 103006|Guyana.Upper Takutu-Upper Essequibo
Pteronotus parnellii|ABGYB376-06|ROM 103083|Guyana.Upper Takutu-Upper Essequibo
Pteronotus parnellii|ABGYB377-06|ROM 103084|Guyana.Upper Takutu-Upper Essequibo
Pteronotus parnellii|ABGYB418-06|ROM 103126|Guyana.Upper Takutu-Upper Essequibo
Pteronotus parnellii|ABGYB620-06|ROM 103375|Guyana.Upper Demerara-Berbice
Pteronotus parnellii|ABGYB661-06|ROM 103420|Guyana.Upper Demerara-Berbice
Pteronotus parnellii|ABGYB702-06|ROM 103466|Guyana.Upper Demerara-Berbice
Pteronotus parnellii|ABGYB925-06|ROM 104770|Guyana.Potaro-Siparuni
Pteronotus parnellii|ABGYC002-06|ROM 104779|Guyana.Potaro-Siparuni
Pteronotus parnellii|ABGYA754-06|ROM 100460|Guyana.East Berbice-Corentyne
Pteronotus parnellii|ABGYA756-06|ROM 100462|Guyana.East Berbice-Corentyne
Pteronotus parnellii|ABGYC021-06|ROM 104798|Guyana.Potaro-Siparuni
Pteronotus parnellii|ABGYC078-06|ROM 106575|Guyana.Upper Takutu-Upper Essequibo
Pteronotus parnellii|ABGYC151-06|ROM 106667|Guyana.Upper Takutu-Upper Essequibo
Pteronotus parnellii|ABGYC202-06|ROM 106741|Guyana.Upper Takutu-Upper Essequibo
Pteronotus parnellii|ABGYC260-06|ROM 107046|Guyana.Potaro-Siparuni
Pteronotus parnellii|ABGYC384-06|ROM 107201|Guyana.Potaro-Siparuni
Pteronotus parnellii|ABGYC392-06|ROM 107211|Guyana.Potaro-Siparuni
Pteronotus parnellii|ABGYC449-06|ROM 107274|Guyana.Potaro-Siparuni
Pteronotus parnellii|ABGYD603-06|ROM 108968|Guyana.Potaro-Siparuni
Pteronotus parnellii|ABGYD627-06|ROM 109024|Guyana.Potaro-Siparuni
Pteronotus parnellii|ABGYE153-06|ROM 111596|Guyana.Potaro-Siparuni
Pteronotus parnellii|ABGYE564-06|ROM 112038|Guyana.Potaro-Siparuni
Pteronotus parnellii|ABGYE689-06|ROM 112619|Guyana.Demerara-Mahaica
Pteronotus parnellii|ABGYE726-06|ROM 112666|Guyana.Demerara-Mahaica
Pteronotus parnellii|ABGYE727-06|ROM 112667|Guyana.Demerara-Mahaica
Pteronotus parnellii|ABGYE728-06|ROM 112668|Guyana.Demerara-Mahaica
Pteronotus parnellii|ABGYE765-06|ROM 113365|Guyana.Upper Demerara-Berbice
Pteronotus parnellii|ABGYE793-06|ROM 113394|Guyana.Upper Demerara-Berbice
Pteronotus parnellii|ABGYE795-06|ROM 113396|Guyana.Upper Demerara-Berbice
Pteronotus parnellii|ABGYF061-06|ROM 113578|Guyana.Upper Takutu-Upper Essequibo
Pteronotus parnellii|ABGYF182-06|ROM 113739|Guyana.Demerara-Mahaica
Pteronotus parnellii|ABGYF242-06|ROM 113823|Guyana.Demerara-Mahaica
Pteronotus parnellii|ABGYF243-06|ROM 113824|Guyana.Demerara-Mahaica
Pteronotus parnellii|ABGYG002-06|ROM 115481|Guyana.Essequibo Islands-West Demerara
Pteronotus parnellii|ABGYG003-06|ROM 115482|Guyana.Essequibo Islands-West Demerara
Pteronotus parnellii|ABGYG004-06|ROM 115483|Guyana.Essequibo Islands-West Demerara
Pteronotus parnellii|ABGYA687-06|ROM 100392|Guyana.East Berbice-Corentyne
Pteronotus parnellii|ABGYA688-06|ROM 100393|Guyana.East Berbice-Corentyne
Pteronotus parnellii|ABGYA683-06|ROM 100388|Guyana.East Berbice-Corentyne
Pteronotus parnellii|ABGYA684-06|ROM 100389|Guyana.East Berbice-Corentyne
Pteronotus parnellii|ABGYA678-06|ROM 100383|Guyana.East Berbice-Corentyne
Pteronotus parnellii|ABGYA679-06|ROM 100384|Guyana.East Berbice-Corentyne
Pteronotus parnellii|ABGYA593-06|ROM 100280|Guyana.East Berbice-Corentyne
Pteronotus parnellii|ABGYA594-06|ROM 100281|Guyana.East Berbice-Corentyne
Pteronotus parnellii|ABGYA564-06|ROM 100240|Guyana.East Berbice-Corentyne
Pteronotus parnellii|ABGYA572-06|ROM 100251|Guyana.East Berbice-Corentyne
Pteronotus parnellii|ABGYA563-06|ROM 100239|Guyana.East Berbice-Corentyne
Pteronotus parnellii|ABGYA238-06|ROM 98127|Guyana.Upper Demerara-Berbice
Pteronotus parnellii|ABGYA232-06|ROM 98110|Guyana.Upper Takutu-Upper Essequibo
Pteronotus parnellii|ABGYA127-06|ROM 97967|Guyana.Upper Takutu-Upper Essequibo
Pteronotus parnellii|ABGYA126-06|ROM 97966|Guyana.Upper Takutu-Upper Essequibo
Pteronotus parnellii|ABGYA125-06|ROM 97965|Guyana.Upper Takutu-Upper Essequibo
Pteronotus parnellii|ABGYA124-06|ROM 97964|Guyana.Upper Takutu-Upper Essequibo
Pteronotus parnellii|ABGYA123-06|ROM 97963|Guyana.Upper Takutu-Upper Essequibo
Pteronotus parnellii|ABGYA118-06|ROM 97957|Guyana.Upper Takutu-Upper Essequibo
Pteronotus parnellii|ABGYA233-06|ROM 98111|Guyana.Upper Takutu-Upper Essequibo
Pteronotus parnellii|ABGYB228-06|ROM 102930|Guyana.Upper Takutu-Upper Essequibo
Pteronotus parnellii|ABGYA755-06|ROM 100461|Guyana.East Berbice-Corentyne
Pteronotus parnellii|ABGYB378-06|ROM 103085|Guyana.Upper Takutu-Upper Essequibo
Pteronotus parnellii|ABGYC512-06|ROM 107348|Guyana.Potaro-Siparuni
Pteronotus parnellii|ABGYE009-06|ROM 109292|Guyana.Potaro-Siparuni
Pteronotus parnellii|ABGYF092-06|ROM 113626|Guyana.Demerara-Mahaica
Pteronotus parnellii|ABGYA723-06|ROM 100428|Guyana.East Berbice-Corentyne
Pteronotus parnellii|ABGYF233-06|ROM 113811|Guyana.Demerara-Mahaica
Pteronotus parnellii|ABGYG009-06|ROM 115488|Guyana.Essequibo Islands-West Demerara
Pteronotus parnellii|ABGYG011-06|ROM 115490|Guyana.Essequibo Islands-West Demerara
Pteronotus parnellii|ABGYG013-06|ROM 115493|Guyana.Essequibo Islands-West Demerara
Pteronotus parnellii|ABGYA591-06|ROM 100278|Guyana.East Berbice-Corentyne
Pteronotus parnellii|ABGYA592-06|ROM 100279|Guyana.East Berbice-Corentyne
Pteronotus parnellii|ABGYG027-06|ROM 115532|Guyana.Essequibo Islands-West Demerara
Pteronotus parnellii|ABGYG028-06|ROM 115533|Guyana.Essequibo Islands-West Demerara
Pteronotus parnellii|ABGYG005-06|ROM 115484|Guyana.Essequibo Islands-West Demerara
Pteronotus parnellii|ABGYG006-06|ROM 115485|Guyana.Essequibo Islands-West Demerara
Pteronotus parnellii|ABGYG007-06|ROM 115486|Guyana.Essequibo Islands-West Demerara
Pteronotus parnellii|ABGYG008-06|ROM 115487|Guyana.Essequibo Islands-West Demerara
Pteronotus parnellii|ABGYG049-06|ROM 115559|Guyana.Essequibo Islands-West Demerara
Pteronotus parnellii|ABGYG050-06|ROM 115560|Guyana.Essequibo Islands-West Demerara
Pteronotus parnellii|ABGYG051-06|ROM 115561|Guyana.Essequibo Islands-West Demerara
Pteronotus parnellii|ABGYG060-06|ROM 115578|Guyana.Essequibo Islands-West Demerara
Pteronotus parnellii|ABGYG064-06|ROM 115584|Guyana.Essequibo Islands-West Demerara
Pteronotus parnellii|ABGYG065-06|ROM 115585|Guyana.Essequibo Islands-West Demerara
Pteronotus parnellii|ABGYG071-06|ROM 115596|Guyana.Essequibo Islands-West Demerara
Pteronotus parnellii|ABGYG096-06|ROM 115646|Guyana.Potaro-Siparuni
Pteronotus parnellii|ABGYG103-06|ROM 115653|Guyana.Potaro-Siparuni
Pteronotus parnellii|ABGYG1070-08|ROM 118994|Guyana
Pteronotus parnellii|ABGYG139-06|ROM 115696|Guyana.Potaro-Siparuni
Pteronotus parnellii|ABGYG143-06|ROM 115700|Guyana.Potaro-Siparuni
Pteronotus parnellii|ABGYG766-08|ROM 119194|Guyana
Pteronotus parnellii|ABGYG808-08|ROM 119236|Guyana
Pteronotus parnellii|ABSMS051-06|ROM 117213|Suriname.Sipaliwini
Pteronotus parnellii|ABSMS052-06|ROM 117214|Suriname.Sipaliwini
Pteronotus parnellii|ABSMS064-06|ROM 117226|Suriname.Sipaliwini
Pteronotus parnellii|ABSMS065-06|ROM 117227|Suriname.Sipaliwini
Pteronotus parnellii|ABSMS093-06|ROM 117255|Suriname.Sipaliwini

Pteronotus parnellii|ABSMS064-06|ROM 117226|Suriname.Sipaliwini
Pteronotus parnellii|ABSMS065-06|ROM 117227|Suriname.Sipaliwini
Pteronotus parnellii|ABSMS093-06|ROM 117255|Suriname.Sipaliwini
Pteronotus parnellii|ABSMS120-06|ROM 117282|Suriname.Sipaliwini
Pteronotus parnellii|ABSMS126-06|ROM 117288|Suriname.Sipaliwini
Pteronotus parnellii|ABSMS141-06|ROM 117303|Suriname.Sipaliwini
Pteronotus parnellii|ABSMS176-06|ROM 117338|Suriname.Sipaliwini
Pteronotus parnellii|ABSMS267-06|ROM 117429|Suriname.Sipaliwini
Pteronotus parnellii|ABSMS429-06|ROM 117591|Suriname.Sipaliwini
Pteronotus parnellii|ABSMS492-06|ROM 117654|Suriname.Sipaliwini
Pteronotus parnellii|ABSRA331-06|ROM 116920|Suriname.Nickerie
Pteronotus parnellii|ABSRA423-06|ROM 117012|Suriname.Nickerie
Pteronotus parnellii|ABGYA615-06|ROM 100303|Guyana.East Berbice-Corentyne
Pteronotus parnellii|ABGYA616-06|ROM 100304|Guyana.East Berbice-Corentyne
Pteronotus parnellii|ABGYA681-06|ROM 100386|Guyana.East Berbice-Corentyne
Pteronotus parnellii|ABGYA682-06|ROM 100387|Guyana.East Berbice-Corentyne
Pteronotus parnellii|BCBNT336-06|ROM 108916|Guyana.Potaro-Siparuni
Pteronotus parnellii|BCBNT767-06|ROM 113978|Suriname.Brokopondo
Pteronotus parnellii|ABGYB728-06|ROM 103494|Guyana.Upper Demerara-Berbice
Pteronotus parnellii|ABGYC520-06|ROM 107358|Guyana.Potaro-Siparuni
Pteronotus parnellii|ABGYE037-06|ROM 109323|Guyana.Potaro-Siparuni
Pteronotus parnellii|ABGYE098-06|ROM 111541|Guyana.Potaro-Siparuni
Pteronotus parnellii|ABGYG119-06|ROM 115671|Guyana.Potaro-Siparuni
Pteronotus parnellii|ABGYG384-06|ROM 116507|Guyana.Potaro-Siparuni
Pteronotus parnellii|ABGYG459-06|ROM 116596|Guyana.Potaro-Siparuni
Pteronotus parnellii|ABGYB892-06|ROM 104737|Guyana.Potaro-Siparuni
Pteronotus parnellii|ABGYC279-06|ROM 107070|Guyana.Potaro-Siparuni
Pteronotus parnellii|ABGYC506-06|ROM 107341|Guyana.Potaro-Siparuni
Pteronotus parnellii|ABGYE469-06|ROM 111937|Guyana.Potaro-Siparuni
Pteronotus parnellii|ABGYE565-06|ROM 112039|Guyana.Potaro-Siparuni
Pteronotus parnellii|ABGYE766-06|ROM 113366|Guyana.Upper Demerara-Berbice
Pteronotus parnellii|ABGYF042-06|ROM 113549|Guyana.Upper Takutu-Upper Essequibo
Pteronotus parnellii|ABGYF052-06|ROM 113563|Guyana.Upper Takutu-Upper Essequibo
Pteronotus parnellii|ABGYF053-06|ROM 113564|Guyana.Upper Takutu-Upper Essequibo
Pteronotus parnellii|ABGYF054-06|ROM 113565|Guyana.Upper Takutu-Upper Essequibo
Pteronotus parnellii|ABGYG386-06|ROM 116509|Guyana.Potaro-Siparuni
Pteronotus parnellii|ABSMS414-06|ROM 117576|Suriname.Sipaliwini
Pteronotus parnellii|ABGYC042-06|ROM 104819|Guyana.Potaro-Siparuni
Pteronotus parnellii|ABGYE126-06|ROM 111569|Guyana.Potaro-Siparuni
Pteronotus parnellii|ABGYE038-06|ROM 109324|Guyana.Potaro-Siparuni
Pteronotus parnellii|ABGYG457-06|ROM 116594|Guyana.Potaro-Siparuni
Pteronotus parnellii|ABGYG458-06|ROM 116595|Guyana.Potaro-Siparuni
Pteronotus parnellii|ABGYG418-06|ROM 116550|Guyana.Potaro-Siparuni
Pteronotus parnellii|ABGYG419-06|ROM 116551|Guyana.Potaro-Siparuni
Pteronotus parnellii|ABGYC336-06|ROM 107148|Guyana.Potaro-Siparuni
Pteronotus parnellii|ABGYC350-06|ROM 107162|Guyana.Potaro-Siparuni
Pteronotus parnellii|ABGYC252-06|ROM 107038|Guyana.Potaro-Siparuni
Pteronotus parnellii|ABGYC259-06|ROM 107045|Guyana.Potaro-Siparuni
Pteronotus parnellii|ABGYC400-06|ROM 107219|Guyana.Potaro-Siparuni
Pteronotus parnellii|ABGYC451-06|ROM 107276|Guyana.Potaro-Siparuni
Pteronotus parnellii|ABGYC522-06|ROM 107360|Guyana.Potaro-Siparuni
Pteronotus parnellii|ABGYD245-06|ROM 108577|Guyana.Potaro-Siparuni
Pteronotus parnellii|ABGYE091-06|ROM 111534|Guyana.Potaro-Siparuni
Pteronotus parnellii|ABGYE583-06|ROM 112060|Guyana.Potaro-Siparuni
Pteronotus parnellii|ABGYE847-06|ROM 113461|Guyana.Upper Takutu-Upper Essequibo
Pteronotus parnellii|ABGYG099-06|ROM 115649|Guyana.Potaro-Siparuni
Pteronotus parnellii|ABGYG135-06|ROM 115693|Guyana.Potaro-Siparuni
Pteronotus parnellii|ABGYG417-06|ROM 116549|Guyana.Potaro-Siparuni
Pteronotus parnellii|ABGYG492-06|ROM 116633|Guyana.Potaro-Siparuni
Pteronotus parnellii|ABSRA350-06|ROM 116939|Suriname.Nickerie
Pteronotus parnellii|ABGYB619-06|ROM 103374|Guyana.Upper Demerara-Berbice
Pteronotus parnellii|ABGYC068-06|ROM 106555|Guyana.Upper Takutu-Upper Essequibo
Pteronotus parnellii|BCBNT342-06|ROM 108938|Guyana.Potaro-Siparuni
Pteronotus parnellii|BCBNT775-06|ROM 113998|Suriname.Brokopondo
Pteronotus parnellii|ABGYB662-06|ROM 103421|Guyana.Upper Demerara-Berbice
Pteronotus parnellii|ABGYB834-06|ROM 104675|Guyana.Potaro-Siparuni
Pteronotus parnellii|ABGYE584-06|ROM 112061|Guyana.Potaro-Siparuni
Pteronotus parnellii|ABGYG1032-08|ROM 118956|Guyana
Pteronotus parnellii|ABGYG118-06|ROM 115670|Guyana.Potaro-Siparuni
Pteronotus parnellii|ABGYG385-06|ROM 116508|Guyana.Potaro-Siparuni
Pteronotus parnellii|ABGYG456-06|ROM 116593|Guyana.Potaro-Siparuni
Pteronotus parnellii|ABSMS014-06|ROM 117176|Suriname.Sipaliwini
Pteronotus parnellii|ABSMS383-06|ROM 117545|Suriname.Sipaliwini
Pteronotus parnellii|BCBNT788-06|ROM 114045|Suriname.Brokopondo
Pteronotus parnellii|ABSMS105-06|ROM 117267|Suriname.Sipaliwini
Pteronotus parnellii|ABGYF029-06|ROM 113526|Guyana.Upper Takutu-Upper Essequibo
Pteronotus parnellii|ABGYF043-06|ROM 113550|Guyana.Upper Takutu-Upper Essequibo
Pteronotus parnellii|ABGYG1055-08|ROM 118979|Guyana
Pteronotus parnellii|ABSRA270-06|ROM 116859|Suriname.Nickerie
Pteronotus parnellii|BCBNT794-06|ROM 114060|Suriname.Brokopondo
Pteronotus parnellii|ABGYG461-06|ROM 116598|Guyana.Potaro-Siparuni
Pteronotus parnellii|ABGYG142-06|ROM 115699|Guyana.Potaro-Siparuni
Pteronotus parnellii|ABGYG140-06|ROM 115697|Guyana.Potaro-Siparuni
Pteronotus parnellii|ABGYE029-06|ROM 109314|Guyana.Potaro-Siparuni
Pteronotus parnellii|BCBNT348-06|ROM 108953|Guyana.Potaro-Siparuni
Pteronotus parnellii|ABGYG168-06|ROM 115735|Guyana.Potaro-Siparuni
Pteronotus parnellii|ABGYE216-06|ROM 111664|Guyana.Potaro-Siparuni
Pteronotus parnellii|ABGYA722-06|ROM 100427|Guyana.East Berbice-Corentyne
Pteronotus parnellii|ABGYC391-06|ROM 107210|Guyana.Potaro-Siparuni
Pteronotus parnellii|ABGYE768-06|ROM 113368|Guyana.Upper Demerara-Berbice
Pteronotus parnellii|ABGYG098-06|ROM 115648|Guyana.Potaro-Siparuni
Pteronotus parnellii|ABGYF034-06|ROM 113539|Guyana.Upper Takutu-Upper Essequibo
Pteronotus parnellii|ABGYF041-06|ROM 113548|Guyana.Upper Takutu-Upper Essequibo
Pteronotus parnellii|ABGYF275-06|ROM 114619|Guyana.Potaro-Siparuni
Pteronotus parnellii|ABGYG097-06|ROM 115647|Guyana.Potaro-Siparuni
Pteronotus parnellii|ABGYE757-06|ROM 113356|Guyana.Upper Demerara-Berbice
Pteronotus parnellii|ABGYE805-06|ROM 113407|Guyana.Upper Demerara-Berbice
Pteronotus parnellii|ABGYE806-06|ROM 113408|Guyana.Upper Demerara-Berbice
Pteronotus parnellii|ABGYF028-06|ROM 113525|Guyana.Upper Takutu-Upper Essequibo
Pteronotus parnellii|ABGYE396-06|ROM 111848|Guyana.Potaro-Siparuni
Pteronotus parnellii|ABGYE446-06|ROM 111908|Guyana.Potaro-Siparuni
Pteronotus parnellii|ABGYE470-06|ROM 111939|Guyana.Potaro-Siparuni
Pteronotus parnellii|ABGYE545-06|ROM 112018|Guyana.Potaro-Siparuni

Pteronotus parnellii|ABGYE446-06|ROM 111908|Guyana.Potaro-Siparuni
Pteronotus parnellii|ABGYE470-06|ROM 111939|Guyana.Potaro-Siparuni
Pteronotus parnellii|ABGYE545-06|ROM 112018|Guyana.Potaro-Siparuni
Pteronotus parnellii|ABGYC229-06|ROM 106776|Guyana.Upper Takutu-Upper Essequibo
Pteronotus parnellii|ABGYC250-06|ROM 107036|Guyana.Potaro-Siparuni
Pteronotus parnellii|ABGYC251-06|ROM 107037|Guyana.Potaro-Siparuni
Pteronotus parnellii|ABGYC307-06|ROM 107118|Guyana.Potaro-Siparuni
Pteronotus parnellii|ABGYC349-06|ROM 107161|Guyana.Potaro-Siparuni
Pteronotus parnellii|ABGYC373-06|ROM 107189|Guyana.Potaro-Siparuni
Pteronotus parnellii|ABGYC393-06|ROM 107212|Guyana.Potaro-Siparuni
Pteronotus parnellii|ABGYC399-06|ROM 107218|Guyana.Potaro-Siparuni
Pteronotus parnellii|ABGYC410-06|ROM 107232|Guyana.Potaro-Siparuni
Pteronotus parnellii|ABGYC411-06|ROM 107233|Guyana.Potaro-Siparuni
Pteronotus parnellii|ABGYC448-06|ROM 107273|Guyana.Potaro-Siparuni
Pteronotus parnellii|ABGYC450-06|ROM 107275|Guyana.Potaro-Siparuni
Pteronotus parnellii|ABGYC521-06|ROM 107359|Guyana.Potaro-Siparuni
Pteronotus parnellii|ABGYC523-06|ROM 107361|Guyana.Potaro-Siparuni
Pteronotus parnellii|ABGYG1009-08|ROM 118933|Guyana
Pteronotus parnellii|ABGYG1020-08|ROM 118944|Guyana
Pteronotus parnellii|ABGYG104-06|ROM 115654|Guyana.Potaro-Siparuni
Pteronotus parnellii|ABGYG105-06|ROM 115645|Guyana.Potaro-Siparuni
Pteronotus parnellii|ABGYG141-06|ROM 115698|Guyana.Potaro-Siparuni
Pteronotus parnellii|ABGYG167-06|ROM 115734|Guyana.Potaro-Siparuni
Pteronotus parnellii|ABGYG184-06|ROM 115749|Guyana.Potaro-Siparuni
Pteronotus parnellii|ABGYG370-06|ROM 116491|Guyana.Potaro-Siparuni
Pteronotus parnellii|ABGYG396-06|ROM 116524|Guyana.Potaro-Siparuni
Pteronotus parnellii|ABGYG446-06|ROM 116582|Guyana.Potaro-Siparuni
Pteronotus parnellii|ABGYG448-06|ROM 116585|Guyana.Potaro-Siparuni
Pteronotus parnellii|ABGYG495-06|ROM 116636|Guyana.Potaro-Siparuni
Pteronotus parnellii|ABGYG511-06|ROM 116653|Guyana.Potaro-Siparuni
Pteronotus parnellii|ABRMM063-07|ROM F39025|Guyana.Potaro-Siparuni
Pteronotus parnellii|ABGYG508-06|ROM 116650|Guyana.Potaro-Siparuni
Pteronotus parnellii|ABGYG509-06|ROM 116651|Guyana.Potaro-Siparuni
Pteronotus parnellii|ABSMS025-06|ROM 117187|Suriname.Sipaliwini
Pteronotus parnellii|ABSMS036-06|ROM 117198|Suriname.Sipaliwini
Pteronotus parnellii|ABSMS127-06|ROM 117289|Suriname.Sipaliwini
Pteronotus parnellii|ABSMS235-06|ROM 117397|Suriname.Sipaliwini
Pteronotus parnellii|ABSMS405-06|ROM 117567|Suriname.Sipaliwini
Pteronotus parnellii|ABSMS519-06|ROM 117681|Suriname.Sipaliwini
Pteronotus parnellii|ABSRA438-06|ROM 117028|Suriname.Nickerie
Pteronotus parnellii|BCBNT335-06|ROM 108915|Guyana.Potaro-Siparuni
Pteronotus parnellii|ABGYE394-06|ROM 111846|Guyana.Potaro-Siparuni
Pteronotus parnellii|ABGYE395-06|ROM 111847|Guyana.Potaro-Siparuni
Pteronotus parnellii|ABGYE349-06|ROM 111799|Guyana.Potaro-Siparuni
Pteronotus parnellii|ABGYE364-06|ROM 111814|Guyana.Potaro-Siparuni
Pteronotus parnellii|ABGYD602-06|ROM 108967|Guyana.Potaro-Siparuni
Pteronotus parnellii|ABGYE113-06|ROM 111556|Guyana.Potaro-Siparuni
Pteronotus parnellii|ABGYD541-06|ROM 108901|Guyana.Potaro-Siparuni
Pteronotus parnellii|ABGYD593-06|ROM 109015|Guyana.Potaro-Siparuni
Pteronotus parnellii|ABGYC144-06|ROM 106659|Guyana.Upper Takutu-Upper Essequibo
Pteronotus parnellii|ABGYC086-06|ROM 106585|Guyana.Upper Takutu-Upper Essequibo
Pteronotus parnellii|ABGYB862-06|ROM 104706|Guyana.Potaro-Siparuni
Pteronotus parnellii|ABGYB861-06|ROM 104705|Guyana.Potaro-Siparuni
Pteronotus parnellii|ABGYB860-06|ROM 104704|Guyana.Potaro-Siparuni
Pteronotus parnellii|ABGYB707-06|ROM 103471|Guyana.Upper Demerara-Berbice
Pteronotus parnellii|ABGYB663-06|ROM 103422|Guyana.Upper Demerara-Berbice
Pteronotus parnellii|ABGYB621-06|ROM 103376|Guyana.Upper Demerara-Berbice
Pteronotus parnellii|ABGYB269-06|ROM 102973|Guyana.Upper Takutu-Upper Essequibo
Pteronotus parnellii|ABGYA239-06|ROM 98128|Guyana.Upper Demerara-Berbice
Pteronotus parnellii|ABGYF020-06|ROM 113511|Guyana.Upper Takutu-Upper Essequibo
Pteronotus parnellii|ABGYE246-06|ROM 111694|Guyana.Potaro-Siparuni
Pteronotus parnellii|ABGYF060-06|ROM 113577|Guyana.Upper Takutu-Upper Essequibo
Pteronotus parnellii|ABGYG144-06|ROM 115701|Guyana.Potaro-Siparuni
Pteronotus parnellii|BCBNT824-06|ROM 114151|Suriname.Brokopondo
Pteronotus personatus|BCBN079-05|ROM 98438|Guatemala.Alta Verapaz
Pteronotus personatus|ABCSA180-06|ROM FN31428|Guatemala.Alta Verapaz
Pteronotus personatus|BCBN080-05|ROM 98440|Guatemala.Alta Verapaz
Pteronotus personatus|BCBN081-05|ROM 98441|Guatemala.Alta Verapaz
Pteronotus personatus|ABGYA106-06|ROM 97944|Guyana.Upper Takutu-Upper Essequibo
Pteronotus personatus|ABGYA105-06|ROM 97943|Guyana.Upper Takutu-Upper Essequibo
Pteronotus personatus|ABSMS290-06|ROM 117452|Suriname.Sipaliwini
Pteronotus personatus|ABGYG046-06|ROM 115556|Guyana.Essequibo Islands-West Demerara
Pteronotus personatus|ABSMS083-06|ROM 117245|Suriname.Sipaliwini
Pteronotus personatus|BCBNT097-06|ROM 107098|Guyana.Potaro-Siparuni
Pteronotus personatus|ABSMS327-06|ROM 117489|Suriname.Sipaliwini
Pteronotus personatus|ABSMS175-06|ROM 117337|Suriname.Sipaliwini
Pteronotus personatus|ABSMS129-06|ROM 117291|Suriname.Sipaliwini
Pteronotus personatus|ABSMS106-06|ROM 117268|Suriname.Sipaliwini
Pteronotus personatus|ABGYG047-06|ROM 115557|Guyana.Essequibo Islands-West Demerara
Pteronotus personatus|ABSMS197-06|ROM 117359|Suriname.Sipaliwini
Pteronotus personatus|ABSMS199-06|ROM 117361|Suriname.Sipaliwini
Pteronotus personatus|ABSMS204-06|ROM 117366|Suriname.Sipaliwini
Pteronotus personatus|ABSMS035-06|ROM 117197|Suriname.Sipaliwini
Pteronotus personatus|ABSMS085-06|ROM 117247|Suriname.Sipaliwini
Pteronotus personatus|ABSMS148-06|ROM 117310|Suriname.Sipaliwini
Pteronotus personatus|ABSMS247-06|ROM 117409|Suriname.Sipaliwini
Pteronotus personatus|ABSMS296-06|ROM 117458|Suriname.Sipaliwini
Pteronotus personatus|ABSMS328-06|ROM 117490|Suriname.Sipaliwini
Pteronotus personatus|ABSMS377-06|ROM 117539|Suriname.Sipaliwini
Pteronotus personatus|ABSMS455-06|ROM 117617|Suriname.Sipaliwini
Pteronotus personatus|ABGYC422-06|ROM 107244|Guyana.Potaro-Siparuni
Pteronotus personatus|ABGYE824-06|ROM 113432|Guyana.Upper Demerara-Berbice
Pteronotus personatus|BCBNT399-06|ROM 109298|Guyana.Potaro-Siparuni
Pteronotus personatus|ABSMS075-06|ROM 117237|Suriname.Sipaliwini
Pteronotus personatus|ABSRA525-06|ROM 117115|Suriname.Nickerie
Pteronotus personatus|ABSRA477-06|ROM 117067|Suriname.Nickerie
Pteronotus personatus|ABSMS436-06|ROM 117598|Suriname.Sipaliwini
Pteronotus personatus|ABSMS198-06|ROM 117360|Suriname.Sipaliwini
Pteronotus personatus|ABSMS149-06|ROM 117311|Suriname.Sipaliwini
Pteronotus personatus|ABGYG045-06|ROM 115555|Guyana.Essequibo Islands-West Demerara
Pteronotus personatus|ABGYA237-06|ROM 98126|Guyana.Upper Demerara-Berbice
Pteronotus personatus|ABGYC468-06|ROM 107300|Guyana.Potaro-Siparuni
Pteronotus personatus|ABGYG012-06|ROM 115492|Guyana.Essequibo Islands-West Demerara

[illegible]
Pteronotus personatus|ABGYA237-06|ROM 98126|Guyana.Upper Demerara-Berbice
Pteronotus personatus|ABGYC468-06|ROM 107300|Guyana.Potaro-Siparuni
Pteronotus personatus|ABGYG012-06|ROM 115492|Guyana.Essequibo Islands-West Demerara
Pteronotus personatus|ABGYG026-06|ROM 115531|Guyana.Essequibo Islands-West Demerara
Pteronotus personatus|ABSMS084-06|ROM 117246|Suriname.Sipaliwini
Pteronotus personatus|ABSMS130-06|ROM 117292|Suriname.Sipaliwini
Pteronotus personatus|ABSMS150-06|ROM 117312|Suriname.Sipaliwini
Pteronotus personatus|ABSMS151-06|ROM 117313|Suriname.Sipaliwini
Pteronotus personatus|ABSRA409-06|ROM 116998|Suriname.Nickerie
Pteronotus personatus|ABSRA417-06|ROM 117006|Suriname.Nickerie
Pteronotus personatus|BCBNT874-06|ROM 115491|Guyana.Essequibo Islands-West Demerara
Pteronotus personatus|BCBNT920-06|ROM 115597|Guyana.Essequibo Islands-West Demerara
Mormoops megalophylla|ABCSA181-06|ROM FN31433|Guatemala.Alta Verapaz
Mormoops megalophylla|BCBN083-05|ROM 98443|Guatemala.Alta Verapaz
Mormoops megalophylla|BCBN084-05|ROM 98445|Guatemala.Alta Verapaz
Mormoops megalophylla|BCBN085-05|ROM 98446|Guatemala.Alta Verapaz
Mormoops megalophylla|BCBN086-05|ROM 98447|Guatemala.Alta Verapaz
Pteronotus davyi|BCBN070-05|ROM 98424|Guatemala.Alta Verapaz
Pteronotus davyi|ABCSA179-06|ROM FN31426|Guatemala.Alta Verapaz
Pteronotus davyi|BCBN011-05|ROM 95742|Mexico.Campeche
Pteronotus davyi|BCBN068-05|ROM 98422|Guatemala.Alta Verapaz
Pteronotus davyi|BCBN069-05|ROM 98423|Guatemala.Alta Verapaz
Pteronotus davyi|BCBN078-05|ROM 98436|Guatemala.Alta Verapaz
Pteronotus davyi|BCBN194-05|ROM 99291|Guatemala.Peten
Pteronotus davyi|BCBN388-05|ROM 101253|El Salvador.Ahuachapan
Pteronotus davyi|BCBN411-05|ROM 101305|El Salvador.Ahuachapan
Pteronotus davyi|BCBN423-05|ROM 101338|El Salvador.Ahuachapan
Pteronotus gymnonotus|ABSMS491-06|ROM 117653|Suriname.Sipaliwini
Pteronotus gymnonotus|BCBNT215-06|ROM 107925|Venezuela.Bolivar
Pteronotus gymnonotus|BCBNT393-06|ROM 109253|Guyana.Potaro-Siparuni
Pteronotus gymnonotus|BCBNT113-06|ROM 107229|Guyana.Potaro-Siparuni
Pteronotus gymnonotus|BCBNT760-06|ROM 113957|Suriname.Brokopondo
Pteronotus gymnonotus|ABSMS200-06|ROM 117362|Suriname.Sipaliwini
Pteronotus gymnonotus|BCBN648-05|ROM 104265|Panama
Pteronotus gymnonotus|ABSMS131-06|ROM 117293|Suriname.Sipaliwini
Pteronotus gymnonotus|ABSMS147-06|ROM 117309|Suriname.Sipaliwini
Pteronotus gymnonotus|BCBN654-05|ROM 104271|Panama
Pteronotus gymnonotus|BCBNT934-06|ROM 115628|Guyana.Essequibo Islands-West Demerara
Micronycteris minuta|ABECB094-08|ROM 118756|Ecuador
Micronycteris minuta|BCBN844-05|ROM 105529|Ecuador.Napo
Micronycteris minuta|BCBN596-05|ROM 104067|Ecuador.Napo
Micronycteris minuta|BCBN821-05|ROM 105336|Ecuador.Napo
Micronycteris minuta|BCBN822-05|ROM 105339|Ecuador.Napo
Micronycteris minuta|BCBN994-05|ROM 106320|Ecuador.Napo
Micronycteris minuta|ABGYG661-08|ROM 119089|Guyana
Micronycteris minuta|ABGYB337-06|ROM 103044|Guyana.Upper Takutu-Upper Essequibo
Micronycteris minuta|ABGYE410-06|ROM 111863|Guyana.Potaro-Siparuni
Micronycteris minuta|ABGYC245-06|ROM 107025|Guyana.Potaro-Siparuni
Micronycteris minuta|ABGYB822-06|ROM 104663|Guyana.Potaro-Siparuni
Micronycteris minuta|ABGYB825-06|ROM 104666|Guyana.Potaro-Siparuni
Micronycteris minuta|ABGYB905-06|ROM 104750|Guyana.Potaro-Siparuni
Micronycteris minuta|ABGYD698-06|ROM 109111|Guyana.Potaro-Siparuni
Micronycteris minuta|ABGYG810-08|ROM 119238|Guyana
Micronycteris minuta|BCBN134-05|ROM 97958|Guyana.Upper Takutu-Upper Essequibo
Micronycteris minuta|BCBN769-05|ROM 104681|Guyana.Potaro-Siparuni
Micronycteris minuta|ABGYC567-06|ROM 107412|Guyana.Potaro-Siparuni
Micronycteris minuta|ABGYC635-06|ROM F39540|Guyana.Potaro-Siparuni
Micronycteris minuta|ABGYD563-06|ROM 108933|Guyana.Potaro-Siparuni
Micronycteris minuta|BCBN311-05|ROM 100364|Guyana.East Berbice-Corentyne
Micronycteris minuta|BCBNT512-06|ROM 111631|Guyana.Potaro-Siparuni
Micronycteris minuta|BCBNT561-06|ROM 112573|Guyana.Demerara-Mahaica
Lampronycteris brachyotis|BCBN047-05|ROM 98223|Mexico.Quintana Roo
Lampronycteris brachyotis|BCBN048-05|ROM 98224|Mexico.Quintana Roo
Lampronycteris brachyotis|BCBNT392-06|ROM 109224|Guyana.Potaro-Siparuni
Micronycteris hirsuta|BCBN743-05|ROM 104451|Ecuador.Napo
Micronycteris hirsuta|BCBN644-05|ROM 104258|Panama
Micronycteris hirsuta|ABSRA526-06|ROM 117116|Suriname.Nickerie
Micronycteris hirsuta|BCBNT515-06|ROM 111743|Guyana.Potaro-Siparuni
Micronycteris hirsuta|BCBNT349-06|ROM 108958|Guyana.Potaro-Siparuni
Micronycteris hirsuta|ABGYG1039-08|ROM 118963|Guyana
Micronycteris hirsuta|ABSMS196-06|ROM 117358|Suriname.Sipaliwini
Micronycteris hirsuta|BCBN355-05|ROM 101033|Guyana.Barima-Waini
Micronycteris hirsuta|BCBNT765-06|ROM 113969|Suriname.Brokopondo
Micronycteris brosseti|ABGYC616-06|ROM F39138|Guyana.Potaro-Siparuni
Micronycteris brosseti|ABGYG038-06|ROM 115548|Guyana.Essequibo Islands-West Demerara
Micronycteris brosseti|ABGYG037-06|ROM 115547|Guyana.Essequibo Islands-West Demerara
Micronycteris brosseti|BCBNT149-06|ROM 111469|Guyana.Potaro-Siparuni
Micronycteris megalotis|BCBNT106-06|ROM 107172|Guyana.Potaro-Siparuni
Micronycteris megalotis|ABGYB466-06|ROM 103200|Guyana.Upper Takutu-Upper Essequibo
Micronycteris megalotis|ABSMS195-06|ROM 117357|Suriname.Sipaliwini
Micronycteris megalotis|ABSMS282-06|ROM 117444|Suriname.Sipaliwini
Micronycteris megalotis|ABGYA259-06|ROM 98159|Guyana.Potaro-Siparuni
Micronycteris megalotis|ABGYB299-06|ROM 103004|Guyana.Upper Takutu-Upper Essequibo
Micronycteris megalotis|ABGYD072-06|ROM 108369|Guyana.Potaro-Siparuni
Micronycteris megalotis|BCBNT033-06|ROM 106615|Guyana.Upper Takutu-Upper Essequibo
Micronycteris megalotis|BCBNT070-06|ROM 106788|Guyana.Upper Takutu-Upper Essequibo
Micronycteris megalotis|ABRMM066-07|ROM F39082|Guyana.Potaro-Siparuni
Micronycteris megalotis|BCBN139-05|ROM 98038|Guyana.Potaro-Siparuni
Micronycteris megalotis|BCBNT128-06|ROM 107354|Guyana.Potaro-Siparuni
Micronycteris megalotis|BCBN886-05|ROM 105709|Ecuador.Napo
Micronycteris megalotis|BCBN986-05|ROM 106132|Ecuador.Napo
Micronycteris megalotis|BCBN563-05|ROM 103999|Ecuador.Napo
Micronycteris megalotis|ABECB120-08|ROM 118782|Ecuador
Micronycteris megalotis|BCBN739-05|ROM 104440|Ecuador.Napo
Micronycteris megalotis|BCBN987-05|ROM 106148|Ecuador.Napo
Micronycteris megalotis|ABMXA925-06|ROM FN33834|Mexico.Quintana Roo
Micronycteris megalotis|BCBN466-05|ROM 97379|Mexico.Quintana Roo
Micronycteris megalotis|BCBN490-05|ROM 97452|Mexico.Quintana Roo
Micronycteris megalotis|BCBN511-05|ROM 97625|Mexico.Chiapas
Micronycteris megalotis|BCBN914-05|ROM 105788|Ecuador.Esmeraldas
Micronycteris megalotis|BCBNC071-06|ROM 115234|Guyana.Cuyuni-Mazaruni
Micronycteris megalotis|BCBN602-05|ROM 104195|Panama.Canal Zone
[illegible]

Micronycteris megalotis|BCBN914-05|ROM 105788|Ecuador.Esmeraldas
Micronycteris megalotis|BCBNC071-06|ROM 115234|Guyana.Cuyuni-Mazaruni
Micronycteris megalotis|BCBN602-05|ROM 104195|Panama.Canal Zone
Micronycteris megalotis|BCBN660-05|ROM 104279|Panama
Micronycteris megalotis|BCBNT155-06|ROM 107816|Venezuela.Amazonas
Micronycteris megalotis|BCBNT207-06|ROM 107913|Venezuela.Bolivar
Micronycteris microtis|BCBNT482-06|ROM 111099|Brazil.Sao Paulo
Micronycteris microtis|BCBNT493-06|ROM 111129|Brazil.Sao Paulo
Micronycteris megalotis|ABGYE207-06|ROM 111654|Guyana.Potaro-Siparuni
Micronycteris megalotis|BCBNT588-06|ROM 113398|Guyana.Upper Demerara-Berbice
Micronycteris megalotis|BCBNT777-06|ROM 114004|Suriname.Brokopondo
Micronycteris megalotis|BCBNC157-06|ROM 115924|Guyana.Potaro-Siparuni
Micronycteris megalotis|BCBNC144-06|ROM 115863|Guyana.Potaro-Siparuni
Micronycteris megalotis|ABGYG261-06|ROM 115871|Guyana.Potaro-Siparuni
Micronycteris megalotis|BCBNC162-06|ROM 115936|Guyana.Potaro-Siparuni
Micronycteris megalotis|ABGYG400-06|ROM 116528|Guyana.Potaro-Siparuni
Micronycteris megalotis|BCBNC214-06|ROM 116731|Guyana.Potaro-Siparuni
Micronycteris megalotis|BCBNT663-06|ROM 113663|Guyana.Demerara-Mahaica
Micronycteris megalotis|BCBNT234-06|ROM 108131|Guyana.Cuyuni-Mazaruni
Micronycteris megalotis|BCBN336-05|ROM 100918|Guyana.Barima-Waini
Micronycteris megalotis|BCBNT231-06|ROM 108094|Guyana.Cuyuni-Mazaruni
Micronycteris megalotis|ABGYG979-08|ROM 119407|Guyana
Micronycteris megalotis|ABGYD456-06|ROM 108807|Guyana.Potaro-Siparuni
Micronycteris megalotis|ABGYD457-06|ROM 108808|Guyana.Potaro-Siparuni
Micronycteris megalotis|ABGYA058-06|ROM 97865|Guyana.Upper Takutu-Upper Essequibo
Micronycteris megalotis|ABGYD399-06|ROM 108745|Guyana.Potaro-Siparuni
Micronycteris megalotis|ABGYD600-06|ROM 108964|Guyana.Potaro-Siparuni
Micronycteris megalotis|ABGYD604-06|ROM 108970|Guyana.Potaro-Siparuni
Micronycteris megalotis|ABGYC284-06|ROM 107078|Guyana.Potaro-Siparuni
Micronycteris megalotis|ABGYE027-06|ROM 109312|Guyana.Potaro-Siparuni
Micronycteris megalotis|BCBNT517-06|ROM 111824|Guyana.Potaro-Siparuni
Micronycteris megalotis|BCBNC116-06|ROM 115764|Guyana.Potaro-Siparuni
Micronycteris megalotis|BCBNT972-06|ROM 114621|Guyana.Potaro-Siparuni
Mimon crenulatum|BCBN931-05|ROM 105808|Ecuador.Esmeraldas
Mimon crenulatum|BCBN900-05|ROM 105754|Ecuador.Napo
Mimon crenulatum|BCBN786-05|ROM 105179|Ecuador.Napo
Mimon crenulatum|ABECA691-06|ROM F40041|Ecuador.Napo
Mimon crenulatum|BCBN901-05|ROM 105755|Ecuador.Napo
Mimon crenulatum|ABECA692-06|ROM 105753|Ecuador.Napo
Mimon crenulatum|BCBN851-05|ROM 105539|Ecuador.Napo
Mimon crenulatum|ABECA822-06|ROM 105992|Ecuador.Napo
Mimon crenulatum|BCBN968-05|ROM 105991|Ecuador.Napo
Mimon crenulatum|BCBNT157-06|ROM 107820|Venezuela.Amazonas
Mimon crenulatum|ABGYG871-08|ROM 119299|Guyana
Mimon crenulatum|ABGYG905-08|ROM 119333|Guyana
Mimon crenulatum|ABGYG906-08|ROM 119334|Guyana
Mimon crenulatum|ABGYG940-08|ROM 119368|Guyana
Mimon crenulatum|ABGYE351-06|ROM 111801|Guyana.Potaro-Siparuni
Mimon crenulatum|ABGYC064-06|ROM 106550|Guyana.Upper Takutu-Upper Essequibo
Mimon crenulatum|ABSMS179-06|ROM 117341|Suriname.Sipaliwini
Mimon crenulatum|ABGYE090-06|ROM 111533|Guyana.Potaro-Siparuni
Mimon crenulatum|ABGYB069-06|ROM 101045|Guyana.Barima-Waini
Mimon crenulatum|ABGYC733-06|ROM 108132|Guyana.Cuyuni-Mazaruni
Mimon crenulatum|ABGYC769-06|ROM 108171|Guyana.Cuyuni-Mazaruni
Mimon crenulatum|ABGYC816-06|ROM F43259|Guyana.Cuyuni-Mazaruni
Mimon crenulatum|ABGYB766-06|ROM 103534|Guyana.Upper Demerara-Berbice
Mimon crenulatum|ABGYC614-06|ROM F39108|Guyana.Potaro-Siparuni
Mimon crenulatum|ABGYG157-06|ROM 115717|Guyana.Potaro-Siparuni
Mimon crenulatum|ABSRA465-06|ROM 117055|Suriname.Nickerie
Mimon crenulatum|ABGYE506-06|ROM 111978|Guyana.Potaro-Siparuni
Mimon crenulatum|ABSMS091-06|ROM 117253|Suriname.Sipaliwini
Mimon crenulatum|ABGYB637-06|ROM 103393|Guyana.Upper Demerara-Berbice
Mimon crenulatum|ABGYE447-06|ROM 111909|Guyana.Potaro-Siparuni
Mimon crenulatum|ABSMS268-06|ROM 117430|Suriname.Sipaliwini
Mimon crenulatum|ABSMS209-06|ROM 117371|Suriname.Sipaliwini
Mimon crenulatum|ABSMS094-06|ROM 117256|Suriname.Sipaliwini
Mimon crenulatum|ABGYE815-06|ROM 113419|Guyana.Upper Demerara-Berbice
Mimon crenulatum|ABGYE814-06|ROM 113418|Guyana.Upper Demerara-Berbice
Mimon crenulatum|ABGYE089-06|ROM 111532|Guyana.Potaro-Siparuni
Mimon crenulatum|ABGYE104-06|ROM 111547|Guyana.Potaro-Siparuni
Mimon crenulatum|ABGYB239-06|ROM 102941|Guyana.Upper Takutu-Upper Essequibo
Mimon crenulatum|ABGYD607-06|ROM 108973|Guyana.Potaro-Siparuni
Mimon crenulatum|ABGYE107-06|ROM 111550|Guyana.Potaro-Siparuni
Mimon crenulatum|ABSMS300-06|ROM 117462|Suriname.Sipaliwini
Mimon crenulatum|BCBNT844-06|ROM 114205|Suriname.Brokopondo
Mimon crenulatum|BCBNT826-06|ROM 114165|Suriname.Brokopondo
Mimon crenulatum|BCBNT839-06|ROM 114194|Suriname.Brokopondo
Mimon crenulatum|BCBNT861-06|ROM 114241|Suriname.Brokopondo
Mimon crenulatum|BCBNT862-06|ROM 114242|Suriname.Brokopondo
Mimon crenulatum|ABGYC547-06|ROM 107386|Guyana.Potaro-Siparuni
Mimon crenulatum|ABGYE028-06|ROM 109313|Guyana.Potaro-Siparuni
Mimon crenulatum|ABGYE773-06|ROM 113374|Guyana.Upper Demerara-Berbice
Mimon crenulatum|BCBNT567-06|ROM 112600|Guyana.Demerara-Mahaica
Mimon crenulatum|ABGYF046-06|ROM 113554|Guyana.Upper Takutu-Upper Essequibo
Mimon crenulatum|ABGYG496-06|ROM 116638|Guyana.Potaro-Siparuni
Mimon crenulatum|ABGYE064-06|ROM 111507|Guyana.Potaro-Siparuni
Mimon crenulatum|ABGYE223-06|ROM 111671|Guyana.Potaro-Siparuni
Mimon crenulatum|ABGYC559-06|ROM 107403|Guyana.Potaro-Siparuni
Mimon crenulatum|ABGYC617-06|ROM F39170|Guyana.Potaro-Siparuni
Mimon crenulatum|ABGYC640-06|ROM F39651|Guyana.Potaro-Siparuni
Mimon crenulatum|ABGYE598-06|ROM 112077|Guyana.Potaro-Siparuni
Mimon crenulatum|ABGYE759-06|ROM 113358|Guyana.Upper Demerara-Berbice
Mimon crenulatum|ABGYD771-06|ROM 109191|Guyana.Potaro-Siparuni
Mimon crenulatum|ABGYC395-06|ROM 107214|Guyana.Potaro-Siparuni
Mimon crenulatum|ABGYD589-06|ROM 109009|Guyana.Potaro-Siparuni
Mimon crenulatum|ABGYD599-06|ROM 108963|Guyana.Potaro-Siparuni
Mimon crenulatum|ABGYE224-06|ROM 111672|Guyana.Potaro-Siparuni
Mimon crenulatum|ABGYF282-06|ROM 114633|Guyana.Potaro-Siparuni
Mimon crenulatum|ABGYF281-06|ROM 114632|Guyana.Potaro-Siparuni
Mimon crenulatum|ABGYF292-06|ROM 114653|Guyana.Potaro-Siparuni
Mimon crenulatum|ABGYG191-06|ROM 115763|Guyana.Potaro-Siparuni
Mimon crenulatum|ABGYE623-06|ROM 112534|Guyana.Demerara-Mahaica
Mimon crenulatum|ABGYE740-06|ROM 112683|Guyana.Demerara-Mahaica

Mimon crenulatum|ABGYG191-06|ROM 115763|Guyana.Potaro-Siparuni
Mimon crenulatum|ABGYE623-06|ROM 112534|Guyana.Demerara-Mahaica
Mimon crenulatum|ABGYE740-06|ROM 112683|Guyana.Demerara-Mahaica
Mimon crenulatum|BCBNT553-06|ROM 112535|Guyana.Demerara-Mahaica
Mimon crenulatum|BCBNT576-06|ROM 112660|Guyana.Demerara-Mahaica
Mimon crenulatum|BCBNT666-06|ROM 113681|Guyana.Demerara-Mahaica
Mimon crenulatum|BCBNT678-06|ROM 113751|Guyana.Demerara-Mahaica
Mimon crenulatum|BCBNT918-06|ROM 115592|Guyana.Essequibo Islands-West Demerara
Mimon crenulatum|BCBNT921-06|ROM 115601|Guyana.Essequibo Islands-West Demerara
Phyllostomus discolor|ABGYG709-08|ROM 119137|Guyana
Phyllostomus discolor|ABGYD656-06|ROM 109064|Guyana.Potaro-Siparuni
Phyllostomus discolor|ABGYD667-06|ROM 109078|Guyana.Potaro-Siparuni
Phyllostomus discolor|BCBNT864-06|ROM 114334|Suriname.Brokopondo
Phyllostomus discolor|BCBN096-05|ROM 98495|Guatemala.El Progreso
Phyllostomus discolor|BCBN093-05|ROM 98492|Guatemala.El Progreso
Phyllostomus discolor|BCBN094-05|ROM 98493|Guatemala.El Progreso
Phyllostomus discolor|BCBN095-05|ROM 98494|Guatemala.El Progreso
Phyllostomus discolor|BCBN410-05|ROM 101304|El Salvador.Ahuachapan
Phyllostomus discolor|BCBN429-05|ROM 101347|El Salvador.Ahuachapan
Phyllostomus discolor|ABGYA043-06|ROM 97834|Guyana.Upper Takutu-Upper Essequibo
Phyllostomus discolor|ABGYF256-06|ROM 113844|Guyana.Demerara-Mahaica
Phyllostomus discolor|BCBNT701-06|ROM 113838|Guyana.Demerara-Mahaica
Phyllostomus discolor|ABGYC142-06|ROM 106656|Guyana.Upper Takutu-Upper Essequibo
Phyllostomus discolor|ABGYD735-06|ROM 109151|Guyana.Potaro-Siparuni
Phyllostomus discolor|ABGYE578-06|ROM 112055|Guyana.Potaro-Siparuni
Phyllostomus discolor|ABGYB919-06|ROM 104764|Guyana.Potaro-Siparuni
Phyllostomus discolor|ABGYD672-06|ROM 109083|Guyana.Potaro-Siparuni
Phyllostomus discolor|ABSRA408-06|ROM 116997|Suriname.Nickerie
Phyllostomus discolor|ABGYD671-06|ROM 109082|Guyana.Potaro-Siparuni
Phyllostomus discolor|ABGYD677-06|ROM 109088|Guyana.Potaro-Siparuni
Phyllostomus discolor|ABGYC258-06|ROM 107043|Guyana.Potaro-Siparuni
Phyllostomus discolor|ABGYE569-06|ROM 112044|Guyana.Potaro-Siparuni
Phyllostomus discolor|ABGYE476-06|ROM 111948|Guyana.Potaro-Siparuni
Phyllostomus discolor|ABGYD655-06|ROM 109063|Guyana.Potaro-Siparuni
Phyllostomus discolor|ABGYC474-06|ROM 107307|Guyana.Potaro-Siparuni
Phyllostomus discolor|ABGYD136-06|ROM 108439|Guyana.Potaro-Siparuni
Phyllostomus discolor|ABGYD490-06|ROM 108844|Guyana.Potaro-Siparuni
Phyllostomus discolor|BCBNT194-06|ROM 107895|Venezuela.Amazonas
Phyllostomus discolor|BCBNT702-06|ROM 113839|Guyana.Demerara-Mahaica
Phyllostomus discolor|ABGYD675-06|ROM 109086|Guyana.Potaro-Siparuni
Phyllostomus discolor|ABGYD669-06|ROM 109080|Guyana.Potaro-Siparuni
Phyllostomus discolor|ABGYF258-06|ROM 113846|Guyana.Demerara-Mahaica
Phyllostomus discolor|ABGYD721-06|ROM 109136|Guyana.Potaro-Siparuni
Phyllostomus discolor|ABGYD708-06|ROM 109122|Guyana.Potaro-Siparuni
Phyllostomus discolor|ABGYD707-06|ROM 109121|Guyana.Potaro-Siparuni
Phyllostomus discolor|ABGYD706-06|ROM 109120|Guyana.Potaro-Siparuni
Phyllostomus discolor|ABGYD686-06|ROM 109098|Guyana.Potaro-Siparuni
Phyllostomus discolor|ABGYD685-06|ROM 109097|Guyana.Potaro-Siparuni
Phyllostomus discolor|ABGYD678-06|ROM 109089|Guyana.Potaro-Siparuni
Phyllostomus discolor|ABGYD676-06|ROM 109087|Guyana.Potaro-Siparuni
Phyllostomus discolor|ABGYD674-06|ROM 109085|Guyana.Potaro-Siparuni
Phyllostomus discolor|ABGYD673-06|ROM 109084|Guyana.Potaro-Siparuni
Phyllostomus discolor|ABGYD670-06|ROM 109081|Guyana.Potaro-Siparuni
Phyllostomus discolor|ABGYD668-06|ROM 109079|Guyana.Potaro-Siparuni
Phyllostomus discolor|ABGYD666-06|ROM 109077|Guyana.Potaro-Siparuni
Phyllostomus discolor|ABGYD501-06|ROM 108859|Guyana.Potaro-Siparuni
Phyllostomus discolor|ABGYA044-06|ROM 97835|Guyana.Upper Takutu-Upper Essequibo
Phyllostomus discolor|ABGYD201-06|ROM 108530|Guyana.Potaro-Siparuni
Phyllostomus discolor|ABGYD734-06|ROM 109150|Guyana.Potaro-Siparuni
Phyllostomus discolor|ABGYD744-06|ROM 109162|Guyana.Potaro-Siparuni
Phyllostomus discolor|ABGYD762-06|ROM 109182|Guyana.Potaro-Siparuni
Phyllostomus discolor|ABGYE003-06|ROM 109284|Guyana.Potaro-Siparuni
Phyllostomus discolor|ABGYF254-06|ROM 113842|Guyana.Demerara-Mahaica
Phyllostomus discolor|ABGYF257-06|ROM 113845|Guyana.Demerara-Mahaica
Phyllostomus discolor|BCBNT584-06|ROM 112692|Guyana.Demerara-Mahaica
Phyllostomus discolor|BCBNT585-06|ROM 112693|Guyana.Demerara-Mahaica
Phyllostomus discolor|BCBNT703-06|ROM 113840|Guyana.Demerara-Mahaica
Phyllostomus discolor|BCBNT809-06|ROM 114116|Suriname.Brokopondo
Phyllostomus discolor|ABGYD621-06|ROM 108997|Guyana.Potaro-Siparuni
Phyllostomus discolor|ABGYG892-08|ROM 119320|Guyana
Phyllostomus discolor|BCBNT816-06|ROM 114127|Suriname.Brokopondo
Phyllostomus discolor|ABGYE570-06|ROM 112045|Guyana.Potaro-Siparuni
Phyllostomus discolor|ABGYC266-06|ROM 107055|Guyana.Potaro-Siparuni
Phyllostomus discolor|ABGYE011-06|ROM 109294|Guyana.Potaro-Siparuni
Phyllostomus discolor|ABGYE756-06|ROM 112702|Guyana.Demerara-Mahaica
Phyllostomus discolor|ABGYF255-06|ROM 113843|Guyana.Demerara-Mahaica
Phyllostomus discolor|ABGYG1029-08|ROM 118953|Guyana
Phyllostomus discolor|ABGYD805-06|ROM 109228|Guyana.Potaro-Siparuni
Phyllostomus discolor|ABGYF253-06|ROM 113841|Guyana.Demerara-Mahaica
Phyllostomus discolor|BCBNT665-06|ROM 113677|Guyana.Demerara-Mahaica
Phyllostomus discolor|BCBNT865-06|ROM 114344|Suriname.Brokopondo
Phyllostomus discolor|ABGYD605-06|ROM 108971|Guyana.Potaro-Siparuni
Phyllostomus discolor|ABGYG710-08|ROM 119138|Guyana
Phyllostomus discolor|BCBNT867-06|ROM 114353|Suriname.Brokopondo
Phyllostomus latifolius|ABGYG431-06|ROM 116566|Guyana.Potaro-Siparuni
Phyllostomus latifolius|ABGYG450-06|ROM 116587|Guyana.Potaro-Siparuni
Phyllostomus latifolius|ABGYG462-06|ROM 116600|Guyana.Potaro-Siparuni
Phyllostomus latifolius|BCBNT796-06|ROM 114063|Suriname.Brokopondo
Phyllostomus latifolius|BCBNT797-06|ROM 114064|Suriname.Brokopondo
Phyllostomus latifolius|BCBNT817-06|ROM 114129|Suriname.Brokopondo
Phyllostomus latifolius|BCBNT818-06|ROM 114130|Suriname.Brokopondo
Phyllostomus hastatus|ABSCA111-06|ROM F38206|Panama.Darien
Phyllostomus hastatus|BCBN707-05|ROM 104360|Panama.Darien
Phyllostomus hastatus|BCBN708-05|ROM 104361|Panama.Darien
Phyllostomus hastatus|BCBN872-05|ROM 105673|Ecuador.Napo
Phyllostomus hastatus|ABECA182-06|ROM F37342|Ecuador.Napo
Phyllostomus hastatus|ABECA454-06|ROM 105364|Ecuador.Napo
Phyllostomus hastatus|ABECA406-06|ROM 105306|Ecuador.Napo
Phyllostomus hastatus|ABGYE085-06|ROM 111528|Guyana.Potaro-Siparuni
Phyllostomus hastatus|ABGYE388-06|ROM 111840|Guyana.Potaro-Siparuni
Phyllostomus hastatus|ABECA122-06|ROM 104417|Ecuador.Napo
Phyllostomus hastatus|ABECA944-06|ROM 106135|Ecuador.Napo
Phyllostomus hastatus|BCBN873-05|ROM 105674|Ecuador.Napo

Phyllostomus hastatus|ABECA122-06|ROM 104417|Ecuador.Napo
Phyllostomus hastatus|ABECA944-06|ROM 106135|Ecuador.Napo
Phyllostomus hastatus|BCBN873-05|ROM 105674|Ecuador.Napo
Phyllostomus hastatus|BCBNT184-06|ROM 107883|Venezuela.Amazonas
Phyllostomus hastatus|ABECA524-06|ROM 105554|Ecuador.Napo
Phyllostomus hastatus|ABECA297-06|ROM 105168|Ecuador.Napo
Phyllostomus hastatus|BCBNT186-06|ROM 107885|Venezuela.Amazonas
Phyllostomus hastatus|ABGYB288-06|ROM 102992|Guyana.Upper Takutu-Upper Essequibo
Phyllostomus hastatus|ABGYE356-06|ROM 111806|Guyana.Potaro-Siparuni
Phyllostomus hastatus|ABGYF068-06|ROM 113592|Guyana.Upper Takutu-Upper Essequibo
Phyllostomus hastatus|BCBNT197-06|ROM 107902|Venezuela.Amazonas
Phyllostomus hastatus|ABGYA617-06|ROM 100307|Guyana.East Berbice-Corentyne
Phyllostomus hastatus|ABGYB560-06|ROM 103296|Guyana.Upper Takutu-Upper Essequibo
Phyllostomus hastatus|ABECA458-06|ROM 105368|Ecuador.Napo
Phyllostomus hastatus|ABECA449-06|ROM 105358|Ecuador.Napo
Phyllostomus hastatus|BCBNT185-06|ROM 107884|Venezuela.Amazonas
Phyllostomus hastatus|ABGYE525-06|ROM 111998|Guyana.Potaro-Siparuni
Phyllostomus hastatus|ABSMS301-06|ROM 117463|Suriname.Sipaliwini
Phyllostomus hastatus|ABGYE021-06|ROM 109305|Guyana.Potaro-Siparuni
Phyllostomus hastatus|ABGYC408-06|ROM 107230|Guyana.Potaro-Siparuni
Phyllostomus hastatus|ABECA407-06|ROM 105307|Ecuador.Napo
Phyllostomus hastatus|ABECA945-06|ROM 106136|Ecuador.Napo
Phyllostomus hastatus|ABGYB469-06|ROM 103203|Guyana.Upper Takutu-Upper Essequibo
Phyllostomus hastatus|BCBNT381-06|ROM 109090|Guyana.Potaro-Siparuni
Phyllostomus hastatus|BCBNT707-06|ROM 113876|Suriname.Brokopondo
Phyllostomus hastatus|BCBNT800-06|ROM 114071|Suriname.Brokopondo
Phyllostomus hastatus|ABECA160-06|ROM 104471|Ecuador.Napo
Phyllostomus hastatus|ABECA078-06|ROM 104073|Ecuador.Napo
Phyllostomus hastatus|ABGYD202-06|ROM 108531|Guyana.Potaro-Siparuni
Phyllostomus hastatus|BCBNT183-06|ROM 107882|Venezuela.Amazonas
Phyllostomus hastatus|ABECA201-06|ROM 104522|Ecuador.Napo
Phyllostomus hastatus|ABECB084-08|ROM 118746|Ecuador
Phyllostomus hastatus|BCBN874-05|ROM 105678|Ecuador.Napo
Phyllostomus hastatus|ABECA197-06|ROM 104518|Ecuador.Napo
Phyllostomus hastatus|ABGYD684-06|ROM 109096|Guyana.Potaro-Siparuni
Phyllostomus hastatus|ABGYB561-06|ROM 103297|Guyana.Upper Takutu-Upper Essequibo
Phyllostomus hastatus|ABGYF227-06|ROM 113800|Guyana.Demerara-Mahaica
Phyllostomus hastatus|BCBN756-05|ROM 104497|Ecuador.Napo
Phyllostomus hastatus|BCBNT810-06|ROM 114117|Suriname.Brokopondo
Phyllostomus hastatus|BCBN283-05|ROM 100216|Guyana.East Berbice-Corentyne
Phyllostomus hastatus|ABGYE063-06|ROM 111505|Guyana.Potaro-Siparuni
Phyllostomus hastatus|ABSMS142-06|ROM 117304|Suriname.Sipaliwini
Phyllostomus hastatus|ABGYF219-06|ROM 113790|Guyana.Demerara-Mahaica
Phyllostomus hastatus|ABGYF218-06|ROM 113789|Guyana.Demerara-Mahaica
Phyllostomus hastatus|BCBNT390-06|ROM 109194|Guyana.Potaro-Siparuni
Phyllostomus hastatus|BCBNT833-06|ROM 114182|Suriname.Brokopondo
Phyllostomus elongatus|ABGYE576-06|ROM 112053|Guyana.Potaro-Siparuni
Phyllostomus elongatus|ABECA522-06|ROM F37791|Ecuador.Napo
Phyllostomus elongatus|ABGYF045-06|ROM 113553|Guyana.Upper Takutu-Upper Essequibo
Phyllostomus elongatus|ABECA523-06|ROM 105553|Ecuador.Napo
Phyllostomus elongatus|BCBN308-05|ROM 100349|Guyana.East Berbice-Corentyne
Phyllostomus elongatus|BCBN788-05|ROM 105185|Ecuador.Napo
Phyllostomus elongatus|ABECA445-06|ROM 105349|Ecuador.Napo
Phyllostomus elongatus|ABECA687-06|ROM 105748|Ecuador.Napo
Phyllostomus elongatus|ABGYA114-06|ROM 97953|Guyana.Upper Takutu-Upper Essequibo
Phyllostomus elongatus|ABGYA080-06|ROM 97903|Guyana.Upper Takutu-Upper Essequibo
Phyllostomus elongatus|ABGYB698-06|ROM 103462|Guyana.Upper Demerara-Berbice
Phyllostomus elongatus|ABGYD107-06|ROM 108405|Guyana.Potaro-Siparuni
Phyllostomus elongatus|ABGYD810-06|ROM 109233|Guyana.Potaro-Siparuni
Phyllostomus elongatus|ABGYE471-06|ROM 111940|Guyana.Potaro-Siparuni
Phyllostomus elongatus|ABGYG1053-08|ROM 118977|Guyana
Phyllostomus elongatus|ABGYE399-06|ROM 111851|Guyana.Potaro-Siparuni
Phyllostomus elongatus|ABECA403-06|ROM F37626|Ecuador.Napo
Phyllostomus elongatus|ABGYD840-06|ROM 109266|Guyana.Potaro-Siparuni
Phyllostomus elongatus|ABGYF022-06|ROM 113517|Guyana.Upper Takutu-Upper Essequibo
Phyllostomus elongatus|ABECA408-06|ROM 105308|Ecuador.Napo
Phyllostomus elongatus|ABECA761-06|ROM 105912|Ecuador.Napo
Phyllostomus elongatus|ABECB089-08|ROM 118751|Ecuador
Phyllostomus elongatus|ABGYB612-06|ROM 103363|Guyana.Upper Demerara-Berbice
Phyllostomus elongatus|ABGYA708-06|ROM 100413|Guyana.East Berbice-Corentyne
Phyllostomus elongatus|ABGYC265-06|ROM 107054|Guyana.Potaro-Siparuni
Phyllostomus elongatus|ABGYC580-06|ROM 107428|Guyana.Potaro-Siparuni
Phyllostomus elongatus|ABGYD379-06|ROM 108724|Guyana.Potaro-Siparuni
Phyllostomus elongatus|ABECA885-06|ROM 106064|Ecuador.Napo
Phyllostomus elongatus|ABECA056-06|ROM F37146|Ecuador.Napo
Phyllostomus elongatus|ABECA144-06|ROM 104444|Ecuador.Napo
Phyllostomus elongatus|BCBN740-05|ROM 104443|Ecuador.Napo
Phyllostomus elongatus|ABGYA156-06|ROM 98007|Guyana.Potaro-Siparuni
Phyllostomus elongatus|ABECB066-06|ROM 106364|Ecuador.Napo
Phyllostomus elongatus|ABGYG193-06|ROM 115769|Guyana.Potaro-Siparuni
Phyllostomus elongatus|BCBN305-05|ROM 100335|Guyana.East Berbice-Corentyne
Phyllostomus elongatus|BCBN309-05|ROM 100350|Guyana.East Berbice-Corentyne
Phyllostomus elongatus|ABECA955-06|ROM 106146|Ecuador.Napo
Phyllostomus elongatus|ABGYE424-06|ROM 111882|Guyana.Potaro-Siparuni
Phyllostomus elongatus|ABGYA261-06|ROM 98161|Guyana.Potaro-Siparuni
Phyllostomus elongatus|ABGYB839-06|ROM 104680|Guyana.Potaro-Siparuni
Phyllostomus elongatus|ABGYD786-06|ROM 109208|Guyana.Potaro-Siparuni
Phyllostomus elongatus|ABGYE353-06|ROM 111803|Guyana.Potaro-Siparuni
Phyllostomus elongatus|ABGYE577-06|ROM 112054|Guyana.Potaro-Siparuni
Phyllostomus elongatus|ABSRA425-06|ROM 117014|Suriname.Nickerie
Phyllostomus elongatus|ABGYE034-06|ROM 109320|Guyana.Potaro-Siparuni
Phyllostomus elongatus|ABGYG108-06|ROM 115657|Guyana.Potaro-Siparuni
Phyllostomus elongatus|ABECA380-06|ROM 105276|Ecuador.Napo
Phyllostomus elongatus|BCBNT168-06|ROM 107842|Venezuela.Amazonas
Phyllostomus elongatus|BCBN736-05|ROM 104428|Ecuador.Napo
Phyllostomus elongatus|BCBN584-05|ROM 104041|Ecuador.Napo
Phyllostomus elongatus|BCBN287-05|ROM 100238|Guyana.East Berbice-Corentyne
Phyllostomus elongatus|ABGYG860-08|ROM 119288|Guyana
Phyllostomus elongatus|ABGYG859-08|ROM 119287|Guyana
Phyllostomus elongatus|ABGYG809-08|ROM 119237|Guyana
Phyllostomus elongatus|ABGYG109-06|ROM 115658|Guyana.Potaro-Siparuni
Phyllostomus elongatus|ABGYG1052-08|ROM 118976|Guyana
Phyllostomus elongatus|ABGYF843-06|ROM 113456|Guyana.Upper Takutu-Upper Essequibo

Phyllostomus elongatus|ABGY[illegible]|ROM [illegible]|Guyana
Phyllostomus elongatus|ABGYG109-06|ROM 115658|Guyana.Potaro-Siparuni
Phyllostomus elongatus|ABGYG1052-08|ROM 118976|Guyana
Phyllostomus elongatus|ABGYE843-06|ROM 113456|Guyana.Upper Takutu-Upper Essequibo
Phyllostomus elongatus|ABGYE769-06|ROM 113370|Guyana.Upper Demerara-Berbice
Phyllostomus elongatus|ABGYE542-06|ROM 112015|Guyana.Potaro-Siparuni
Phyllostomus elongatus|ABGYE499-06|ROM 111971|Guyana.Potaro-Siparuni
Phyllostomus elongatus|ABGYE445-06|ROM 111907|Guyana.Potaro-Siparuni
Phyllostomus elongatus|ABGYE345-06|ROM 111795|Guyana.Potaro-Siparuni
Phyllostomus elongatus|ABGYE335-06|ROM 111785|Guyana.Potaro-Siparuni
Phyllostomus elongatus|ABGYE287-06|ROM 111736|Guyana.Potaro-Siparuni
Phyllostomus elongatus|ABGYE286-06|ROM 111735|Guyana.Potaro-Siparuni
Phyllostomus elongatus|ABGYD804-06|ROM 109227|Guyana.Potaro-Siparuni
Phyllostomus elongatus|ABGYD577-06|ROM 108956|Guyana.Potaro-Siparuni
Phyllostomus elongatus|ABGYD546-06|ROM 108911|Guyana.Potaro-Siparuni
Phyllostomus elongatus|ABGYC101-06|ROM 106609|Guyana.Upper Takutu-Upper Essequibo
Phyllostomus elongatus|ABGYC079-06|ROM 106576|Guyana.Upper Takutu-Upper Essequibo
Phyllostomus elongatus|ABGYB777-06|ROM 103545|Guyana.Upper Demerara-Berbice
Phyllostomus elongatus|ABGYB775-06|ROM 103543|Guyana.Upper Demerara-Berbice
Phyllostomus elongatus|ABGYA664-06|ROM 100365|Guyana.East Berbice-Corentyne
Phyllostomus elongatus|ABGYA198-06|ROM 98066|Guyana.Potaro-Siparuni
Phyllostomus elongatus|ABGYA115-06|ROM 97954|Guyana.Upper Takutu-Upper Essequibo
Phyllostomus elongatus|ABGYA113-06|ROM 97952|Guyana.Upper Takutu-Upper Essequibo
Phyllostomus elongatus|ABECB075-08|ROM 118737|Ecuador
Phyllostomus elongatus|ABECA798-06|ROM 105963|Ecuador.Napo
Phyllostomus elongatus|ABECA688-06|ROM F40038|Ecuador.Napo
Phyllostomus elongatus|ABECA582-06|ROM 105614|Ecuador.Napo
Phyllostomus elongatus|ABECA276-06|ROM 105144|Ecuador.Napo
Phyllostomus elongatus|ABECA168-06|ROM 104481|Ecuador.Napo
Phyllostomus elongatus|ABECA167-06|ROM F37321|Ecuador.Napo
Phyllostomus elongatus|ABECA156-06|ROM 104462|Ecuador.Napo
Phyllostomus elongatus|ABECA084-06|ROM F37192|Ecuador.Napo
Phyllostomus elongatus|ABECA043-06|ROM F37125|Ecuador.Napo
Phyllostomus elongatus|ABECA020-06|ROM 104002|Ecuador.Napo
Phyllostomus elongatus|ABECA003-06|ROM 103982|Ecuador.Napo
Phyllostomus elongatus|ABECA296-06|ROM 105166|Ecuador.Napo
Phyllostomus elongatus|ABGYE189-06|ROM 111635|Guyana.Potaro-Siparuni
Phyllostomus elongatus|ABGYA759-06|ROM 100807|Guyana.Barima-Waini
Phyllostomus elongatus|ABGYA760-06|ROM 100808|Guyana.Barima-Waini
Phyllostomus elongatus|ABGYB868-06|ROM 104712|Guyana.Potaro-Siparuni
Phyllostomus elongatus|ABGYB660-06|ROM 103418|Guyana.Upper Demerara-Berbice
Phyllostomus elongatus|ABGYC321-06|ROM 107133|Guyana.Potaro-Siparuni
Phyllostomus elongatus|ABGYD380-06|ROM 108725|Guyana.Potaro-Siparuni
Phyllostomus elongatus|ABGYG192-06|ROM 115768|Guyana.Potaro-Siparuni
Phyllostomus elongatus|ABSMS310-06|ROM 117472|Suriname.Sipaliwini
Phyllostomus elongatus|ABECA287-06|ROM 105158|Ecuador.Napo
Phyllostomus elongatus|BCBN868-05|ROM 105658|Ecuador.Napo
Phyllostomus elongatus|BCBNT229-06|ROM 107956|Venezuela.Guarico
Phyllostomus elongatus|BCBNT843-06|ROM 114204|Suriname.Brokopondo
Phyllostomus elongatus|BCBNT863-06|ROM 114248|Suriname.Brokopondo
Phyllostomus elongatus|BCBNT873-06|ROM 115480|Guyana.Essequibo Islands-West Demerara
Phyllostomus elongatus|ABSMS101-06|ROM 117263|Suriname.Sipaliwini
Phyllostomus elongatus|ABGYE566-06|ROM 112040|Guyana.Potaro-Siparuni
Phyllostomus elongatus|ABGYC018-06|ROM 104795|Guyana.Potaro-Siparuni
Phyllostomus elongatus|ABGYD503-06|ROM 108861|Guyana.Potaro-Siparuni
Phyllostomus elongatus|ABGYE106-06|ROM 111549|Guyana.Potaro-Siparuni
Phyllostomus elongatus|ABSMS113-06|ROM 117275|Suriname.Sipaliwini
Phyllostomus elongatus|ABGYE537-06|ROM 112010|Guyana.Potaro-Siparuni
Phyllostomus elongatus|BCBNT834-06|ROM 114184|Suriname.Brokopondo
Phyllostomus elongatus|ABSMS178-06|ROM 117340|Suriname.Sipaliwini
Phyllostomus elongatus|ABGYG194-06|ROM 115770|Guyana.Potaro-Siparuni
Phyllostomus elongatus|ABGYF005-06|ROM 113490|Guyana.Upper Takutu-Upper Essequibo
Phyllostomus elongatus|ABGYG1010-08|ROM 118934|Guyana
Phyllostomus elongatus|ABGYE831-06|ROM 113442|Guyana.Upper Demerara-Berbice
Phyllostomus elongatus|ABGYE371-06|ROM 111821|Guyana.Potaro-Siparuni
Phyllostomus elongatus|ABGYE208-06|ROM 111655|Guyana.Potaro-Siparuni
Phyllostomus elongatus|ABGYE102-06|ROM 111545|Guyana.Potaro-Siparuni
Phyllostomus elongatus|ABGYE076-06|ROM 111519|Guyana.Potaro-Siparuni
Phyllostomus elongatus|ABGYD566-06|ROM 108942|Guyana.Potaro-Siparuni
Phyllostomus elongatus|ABGYD502-06|ROM 108860|Guyana.Potaro-Siparuni
Phyllostomus elongatus|ABGYB776-06|ROM 103544|Guyana.Upper Demerara-Berbice
Phyllostomus elongatus|ABGYB640-06|ROM 103396|Guyana.Upper Demerara-Berbice
Phyllostomus elongatus|ABGYB056-06|ROM 101028|Guyana.Barima-Waini
Phyllostomus elongatus|ABGYA260-06|ROM 98160|Guyana.Potaro-Siparuni
Phyllostomus elongatus|ABGYA196-06|ROM 98064|Guyana.Potaro-Siparuni
Phyllostomus elongatus|ABGYB638-06|ROM 103394|Guyana.Upper Demerara-Berbice
Phyllostomus elongatus|ABGYD560-06|ROM 108930|Guyana.Potaro-Siparuni
Phyllostomus elongatus|ABGYE501-06|ROM 111973|Guyana.Potaro-Siparuni
Phyllostomus elongatus|ABGYE526-06|ROM 111999|Guyana.Potaro-Siparuni
Phyllostomus elongatus|ABGYE600-06|ROM 112079|Guyana.Potaro-Siparuni
Phyllostomus elongatus|ABGYG186-06|ROM 115751|Guyana.Potaro-Siparuni
Phyllostomus elongatus|ABSMS160-06|ROM 117322|Suriname.Sipaliwini
Phyllostomus elongatus|ABSMS453-06|ROM 117615|Suriname.Sipaliwini
Phyllostomus elongatus|BCBNT712-06|ROM 113881|Suriname.Brokopondo
Phyllostomus elongatus|ABGYE500-06|ROM 111972|Guyana.Potaro-Siparuni
Phyllostomus elongatus|ABGYF062-06|ROM 113580|Guyana.Upper Takutu-Upper Essequibo
Phyllostomus elongatus|ABGYA782-06|ROM 100834|Guyana.Barima-Waini
Phyllostomus elongatus|ABGYB699-06|ROM 103463|Guyana.Upper Demerara-Berbice
Phyllostomus elongatus|ABSMS161-06|ROM 117323|Suriname.Sipaliwini
Phyllostomus elongatus|BCBN291-05|ROM 100252|Guyana.East Berbice-Corentyne
Phyllostomus elongatus|ABGYG645-08|ROM 119073|Guyana
Phyllostomus elongatus|ABGYG992-08|ROM 119420|Guyana
Phyllostomus elongatus|ABGYG124-06|ROM 115678|Guyana.Potaro-Siparuni
Phyllostomus elongatus|ABGYG594-08|ROM 119022|Guyana
Phyllostomus elongatus|ABGYG1048-08|ROM 118972|Guyana
Phyllostomus elongatus|ABGYG110-06|ROM 115659|Guyana.Potaro-Siparuni
Phyllostomus elongatus|ABGYG1027-08|ROM 118951|Guyana
Phyllostomus elongatus|ABGYG1028-08|ROM 118952|Guyana
Phyllostomus elongatus|ABGYE836-06|ROM 113448|Guyana.Upper Demerara-Berbice
Phyllostomus elongatus|ABGYG1007-08|ROM 118931|Guyana
Phyllostomus elongatus|ABGYE770-06|ROM 113371|Guyana.Upper Demerara-Berbice
Phyllostomus elongatus|ABGYE807-06|ROM 113410|Guyana.Upper Demerara-Berbice
Phyllostomus elongatus|ABGYE222-06|ROM 111670|Guyana.Potaro-Siparuni
Phyllostomus elongatus|ABGYE[illegible]|ROM [illegible]|Guyana.Potaro-Siparuni

Phyllostomus elongatus|ABGYE770-06|ROM [illegible]|Guyana.Upper Demerara-Berbice
Phyllostomus elongatus|ABGYE807-06|ROM 113410|Guyana.Upper Demerara-Berbice
Phyllostomus elongatus|ABGYE222-06|ROM 111670|Guyana.Potaro-Siparuni
Phyllostomus elongatus|ABGYE334-06|ROM 111784|Guyana.Potaro-Siparuni
Phyllostomus elongatus|ABGYE160-06|ROM 111603|Guyana.Potaro-Siparuni
Phyllostomus elongatus|ABGYE188-06|ROM 111634|Guyana.Potaro-Siparuni
Phyllostomus elongatus|BCBNT750-06|ROM 113940|Suriname.Brokopondo
Phyllostomus elongatus|ABGYE147-06|ROM 111590|Guyana.Potaro-Siparuni
Phyllostomus elongatus|ABGYE120-06|ROM 111563|Guyana.Potaro-Siparuni
Phyllostomus elongatus|ABGYE035-06|ROM 109321|Guyana.Potaro-Siparuni
Phyllostomus elongatus|ABGYD606-06|ROM 108972|Guyana.Potaro-Siparuni
Phyllostomus elongatus|ABGYC283-06|ROM 107077|Guyana.Potaro-Siparuni
Phyllostomus elongatus|ABGYC195-06|ROM 106733|Guyana.Upper Takutu-Upper Essequibo
Phyllostomus elongatus|ABGYC096-06|ROM 106602|Guyana.Upper Takutu-Upper Essequibo
Phyllostomus elongatus|ABGYC047-06|ROM 104824|Guyana.Potaro-Siparuni
Phyllostomus elongatus|ABGYB639-06|ROM 103395|Guyana.Upper Demerara-Berbice
Phyllostomus elongatus|ABGYB562-06|ROM 103298|Guyana.Upper Takutu-Upper Essequibo
Phyllostomus elongatus|ABGYB470-06|ROM 103204|Guyana.Upper Takutu-Upper Essequibo
Phyllostomus elongatus|ABGYB240-06|ROM 102942|Guyana.Upper Takutu-Upper Essequibo
Phyllostomus elongatus|ABGYB138-06|ROM 101122|Guyana.Barima-Waini
Phyllostomus elongatus|ABGYA716-06|ROM 100421|Guyana.East Berbice-Corentyne
Phyllostomus elongatus|ABGYA197-06|ROM 98065|Guyana.Potaro-Siparuni
Phyllostomus elongatus|ABGYG146-06|ROM 115703|Guyana.Potaro-Siparuni
Phyllostomus elongatus|ABGYB188-06|ROM 102890|Guyana.Upper Takutu-Upper Essequibo
Phyllostomus elongatus|ABSMS411-06|ROM 117573|Suriname.Sipaliwini
Phyllostomus elongatus|BCBNT924-06|ROM 115605|Guyana.Essequibo Islands-West Demerara
Lonchorhina aurita|ABMXA921-06|ROM FN33830|Mexico.Quintana Roo
Lonchorhina aurita|BCBN051-05|ROM 98227|Mexico.Quintana Roo
Lonchorhina inusitata|BCBNT043-06|ROM 106672|Guyana.Upper Takutu-Upper Essequibo
Lonchorhina inusitata|BCBNC186-06|ROM 116556|Guyana.Potaro-Siparuni
Lonchorhina inusitata|ABSRA300-06|ROM 116889|Suriname.Nickerie
Lonchorhina inusitata|BCBN141-05|ROM 98053|Guyana.Potaro-Siparuni
Lonchorhina inusitata|BCBNT048-06|ROM 106691|Guyana.Upper Takutu-Upper Essequibo
Mimon cozumelae|BCBN045-05|ROM 96534|Mexico.Yucatan
Mimon cozumelae|ABMXA923-06|ROM FN33832|Mexico.Quintana Roo
Mimon cozumelae|ABMXA783-06|ROM FN30472|Mexico.Yucatan
Mimon cozumelae|ABMXA784-06|ROM FN30473|Mexico.Yucatan
Mimon cozumelae|ABMXA785-06|ROM FN30474|Mexico.Yucatan
Mimon cozumelae|BCBN006-05|ROM 95685|Mexico.Campeche
Mimon cozumelae|BCBN226-05|ROM 99492|Guatemala.Peten
Mimon bennettii|ABGYG1056-08|ROM 118980|Guyana
Mimon bennettii|BCBNC130-06|ROM 115797|Guyana.Potaro-Siparuni
Mimon bennettii|BCBNT301-06|ROM 108532|Guyana.Potaro-Siparuni
Lophostoma schulzi|ABGYG1086-08|ROM 119010|Guyana
Lophostoma schulzi|ABSMS266-06|ROM 117428|Suriname.Sipaliwini
Lophostoma schulzi|BCBNC009-06|ROM 114700|Guyana.Potaro-Siparuni
Lophostoma schulzi|BCBN772-05|ROM 104727|Guyana.Potaro-Siparuni
Lophostoma schulzi|BCBNC153-06|ROM 115915|Guyana.Potaro-Siparuni
Lophostoma schulzi|BCBNT808-06|ROM 114115|Suriname.Brokopondo
Lophostoma schulzi|BCBNT838-06|ROM 114191|Suriname.Brokopondo
Lophostoma evotis|BCBN005-05|ROM 95626|Mexico.Campeche
Lophostoma evotis|BCBN007-05|ROM 95705|Mexico.Campeche
Lophostoma evotis|BCBN183-05|ROM 99239|Guatemala.Peten
Lophostoma silvicolum|ABECA845-06|ROM 106017|Ecuador.Napo
Lophostoma silvicolum|BCBN748-05|ROM 104466|Ecuador.Napo
Lophostoma silvicolum|ABSCA050-06|ROM F38068|Panama
Lophostoma silvicolum|BCBN627-05|ROM 104232|Panama
Lophostoma silvicolum|BCBN643-05|ROM 104257|Panama
Lophostoma silvicolum|BCBN792-05|ROM 105193|Ecuador.Napo
Lophostoma silvicolum|ABECA123-06|ROM 104419|Ecuador.Napo
Lophostoma silvicolum|ABECA177-06|ROM F37333|Ecuador.Napo
Lophostoma silvicolum|BCBN755-05|ROM 104490|Ecuador.Napo
Lophostoma silvicolum|ABECA628-06|ROM 105671|Ecuador.Napo
Lophostoma silvicolum|ABECB078-08|ROM 118740|Ecuador
Lophostoma silvicolum|ABECA301-06|ROM 105172|Ecuador.Napo
Lophostoma silvicolum|ABECA943-06|ROM 106134|Ecuador.Napo
Lophostoma silvicolum|ABGYE387-06|ROM 111839|Guyana.Potaro-Siparuni
Lophostoma silvicolum|ABGYG870-08|ROM 119298|Guyana
Lophostoma silvicolum|ABECA729-06|ROM 105872|Ecuador.Napo
Lophostoma silvicolum|ABECB164-08|ROM 118842|Ecuador
Lophostoma silvicolum|ABECA106-06|ROM 104398|Ecuador.Napo
Lophostoma silvicolum|ABECA762-06|ROM 105913|Ecuador.Napo
Lophostoma silvicolum|ABECA825-06|ROM 105993|Ecuador.Napo
Lophostoma silvicolum|ABECA490-06|ROM 105512|Ecuador.Napo
Lophostoma silvicolum|BCBN589-05|ROM 104049|Ecuador.Napo
Lophostoma silvicolum|ABECA129-06|ROM 104426|Ecuador.Napo
Lophostoma silvicolum|ABECA540-06|ROM F37809|Ecuador.Napo
Lophostoma silvicolum|ABECA557-06|ROM 105586|Ecuador.Napo
Lophostoma silvicolum|ABECA436-06|ROM 105335|Ecuador.Napo
Lophostoma silvicolum|ABECA376-06|ROM F37594|Ecuador.Napo
Lophostoma silvicolum|ABECA326-06|ROM 105208|Ecuador.Napo
Lophostoma silvicolum|ABECA105-06|ROM 104397|Ecuador.Napo
Lophostoma silvicolum|BCBN800-05|ROM 105217|Ecuador.Napo
Lophostoma silvicolum|ABGYB692-06|ROM 103456|Guyana.Upper Demerara-Berbice
Lophostoma silvicolum|ABGYE209-06|ROM 111656|Guyana.Potaro-Siparuni
Lophostoma silvicolum|ABGYA576-06|ROM 100259|Guyana.East Berbice-Corentyne
Lophostoma silvicolum|ABGYC048-06|ROM 104825|Guyana.Potaro-Siparuni
Lophostoma silvicolum|ABGYC212-06|ROM 106753|Guyana.Upper Takutu-Upper Essequibo
Lophostoma silvicolum|ABGYD782-06|ROM 109203|Guyana.Potaro-Siparuni
Lophostoma silvicolum|ABGYE072-06|ROM 111515|Guyana.Potaro-Siparuni
Lophostoma silvicolum|ABGYE575-06|ROM 112052|Guyana.Potaro-Siparuni
Lophostoma silvicolum|ABGYF026-06|ROM 113521|Guyana.Upper Takutu-Upper Essequibo
Lophostoma silvicolum|ABGYC292-06|ROM 107091|Guyana.Potaro-Siparuni
Lophostoma silvicolum|ABGYF115-06|ROM 113654|Guyana.Demerara-Mahaica
Lophostoma silvicolum|ABGYC603-06|ROM 107460|Guyana.Potaro-Siparuni
Lophostoma silvicolum|ABGYD800-06|ROM 109222|Guyana.Potaro-Siparuni
Lophostoma silvicolum|ABGYA091-06|ROM 97925|Guyana.Upper Takutu-Upper Essequibo
Lophostoma silvicolum|ABGYA117-06|ROM 97956|Guyana.Upper Takutu-Upper Essequibo
Lophostoma silvicolum|ABGYA192-06|ROM 98060|Guyana.Potaro-Siparuni
Lophostoma silvicolum|ABGYB476-06|ROM 103211|Guyana.Upper Takutu-Upper Essequibo
Lophostoma silvicolum|ABGYD620-06|ROM 108996|Guyana.Potaro-Siparuni
Lophostoma silvicolum|ABGYF021-06|ROM 113515|Guyana.Upper Takutu-Upper Essequibo
Lophostoma silvicolum|ABGYE352-06|ROM 111802|Guyana.Potaro-Siparuni

Lophostoma silvicolum|ABGYD620-06|ROM 108996|Guyana.Potaro-Siparuni
Lophostoma silvicolum|ABGYF021-06|ROM 113515|Guyana.Upper Takutu-Upper Essequibo
Lophostoma silvicolum|ABGYE352-06|ROM 111802|Guyana.Potaro-Siparuni
Lophostoma silvicolum|ABGYG1054-08|ROM 118978|Guyana
Lophostoma silvicolum|ABGYA090-06|ROM 97924|Guyana.Upper Takutu-Upper Essequibo
Lophostoma silvicolum|ABGYA714-06|ROM 100419|Guyana.East Berbice-Corentyne
Lophostoma silvicolum|ABGYC498-06|ROM 107331|Guyana.Potaro-Siparuni
Lophostoma silvicolum|ABGYC581-06|ROM 107429|Guyana.Potaro-Siparuni
Lophostoma silvicolum|ABGYE344-06|ROM 111794|Guyana.Potaro-Siparuni
Lophostoma silvicolum|ABGYE593-06|ROM 112072|Guyana.Potaro-Siparuni
Lophostoma silvicolum|ABGYE739-06|ROM 112682|Guyana.Demerara-Mahaica
Lophostoma silvicolum|ABGYF025-06|ROM 113520|Guyana.Upper Takutu-Upper Essequibo
Lophostoma silvicolum|ABGYF047-06|ROM 113555|Guyana.Upper Takutu-Upper Essequibo
Lophostoma silvicolum|ABGYG1038-08|ROM 118962|Guyana
Lophostoma silvicolum|ABGYG868-08|ROM 119296|Guyana
Lophostoma silvicolum|ABGYC044-06|ROM 104821|Guyana.Potaro-Siparuni
Lophostoma silvicolum|ABGYF073-06|ROM 113599|Guyana.Upper Takutu-Upper Essequibo
Lophostoma silvicolum|ABGYE128-06|ROM 111571|Guyana.Potaro-Siparuni
Lophostoma silvicolum|ABGYA811-06|ROM 100870|Guyana.Barima-Waini
Lophostoma silvicolum|ABGYD683-06|ROM 109095|Guyana.Potaro-Siparuni
Lophostoma silvicolum|ABGYE087-06|ROM 111530|Guyana.Potaro-Siparuni
Lophostoma silvicolum|ABGYE725-06|ROM 112661|Guyana.Demerara-Mahaica
Lophostoma silvicolum|ABGYF118-06|ROM 113664|Guyana.Demerara-Mahaica
Lophostoma silvicolum|BCBNT805-06|ROM 114102|Suriname.Brokopondo
Lophostoma silvicolum|ABGYG869-08|ROM 119297|Guyana
Lophostoma silvicolum|ABSRA467-06|ROM 117057|Suriname.Nickerie
Lophostoma silvicolum|ABGYB753-06|ROM 103520|Guyana.Upper Demerara-Berbice
Lophostoma silvicolum|ABGYB655-06|ROM 103412|Guyana.Upper Demerara-Berbice
Lophostoma silvicolum|ABGYG170-06|ROM 115737|Guyana.Potaro-Siparuni
Lophostoma silvicolum|ABGYA193-06|ROM 98061|Guyana.Potaro-Siparuni
Lophostoma silvicolum|ABGYB300-06|ROM 103005|Guyana.Upper Takutu-Upper Essequibo
Lophostoma silvicolum|ABGYB913-06|ROM 104758|Guyana.Potaro-Siparuni
Lophostoma silvicolum|ABGYE554-06|ROM 112027|Guyana.Potaro-Siparuni
Lophostoma silvicolum|ABRMM084-07|ROM F39710|Guyana.Potaro-Siparuni
Lophostoma silvicolum|BCBNT764-06|ROM 113967|Suriname.Brokopondo
Lophostoma silvicolum|BCBNT670-06|ROM 113699|Guyana.Demerara-Mahaica
Lophostoma silvicolum|BCBNT669-06|ROM 113698|Guyana.Demerara-Mahaica
Lophostoma silvicolum|BCBNT554-06|ROM 112536|Guyana.Demerara-Mahaica
Lophostoma silvicolum|BCBNC211-06|ROM 116727|Guyana.Potaro-Siparuni
Lophostoma silvicolum|ABSRA509-06|ROM 117099|Suriname.Nickerie
Lophostoma silvicolum|ABSRA333-06|ROM 116922|Suriname.Nickerie
Lophostoma silvicolum|ABSMS017-06|ROM 117179|Suriname.Sipaliwini
Lophostoma silvicolum|ABGYG997-08|ROM 118921|Guyana
Lophostoma silvicolum|ABGYG939-08|ROM 119367|Guyana
Lophostoma silvicolum|ABGYG169-06|ROM 115736|Guyana.Potaro-Siparuni
Lophostoma silvicolum|ABGYG111-06|ROM 115660|Guyana.Potaro-Siparuni
Lophostoma silvicolum|ABGYG1069-08|ROM 118993|Guyana
Lophostoma silvicolum|ABGYG1068-08|ROM 118992|Guyana
Lophostoma silvicolum|ABGYG1037-08|ROM 118961|Guyana
Lophostoma silvicolum|ABGYG088-06|ROM 115627|Guyana.Essequibo Islands-West Demerara
Lophostoma silvicolum|ABGYG075-06|ROM 115603|Guyana.Essequibo Islands-West Demerara
Lophostoma silvicolum|ABGYF146-06|ROM 113697|Guyana.Demerara-Mahaica
Lophostoma silvicolum|ABGYF072-06|ROM 113598|Guyana.Upper Takutu-Upper Essequibo
Lophostoma silvicolum|ABGYF069-06|ROM 113593|Guyana.Upper Takutu-Upper Essequibo
Lophostoma silvicolum|ABGYE845-06|ROM 113459|Guyana.Upper Takutu-Upper Essequibo
Lophostoma silvicolum|ABGYE652-06|ROM 112569|Guyana.Demerara-Mahaica
Lophostoma silvicolum|ABGYE651-06|ROM 112568|Guyana.Demerara-Mahaica
Lophostoma silvicolum|ABGYE574-06|ROM 112051|Guyana.Potaro-Siparuni
Lophostoma silvicolum|ABGYE544-06|ROM 112017|Guyana.Potaro-Siparuni
Lophostoma silvicolum|ABGYE464-06|ROM 111931|Guyana.Potaro-Siparuni
Lophostoma silvicolum|ABGYE463-06|ROM 111930|Guyana.Potaro-Siparuni
Lophostoma silvicolum|ABGYE462-06|ROM 111929|Guyana.Potaro-Siparuni
Lophostoma silvicolum|ABGYE401-06|ROM 111853|Guyana.Potaro-Siparuni
Lophostoma silvicolum|ABGYE386-06|ROM 111838|Guyana.Potaro-Siparuni
Lophostoma silvicolum|ABGYE294-06|ROM 111744|Guyana.Potaro-Siparuni
Lophostoma silvicolum|ABGYE170-06|ROM 111613|Guyana.Potaro-Siparuni
Lophostoma silvicolum|ABGYE148-06|ROM 111591|Guyana.Potaro-Siparuni
Lophostoma silvicolum|ABGYE127-06|ROM 111570|Guyana.Potaro-Siparuni
Lophostoma silvicolum|ABGYE103-06|ROM 111546|Guyana.Potaro-Siparuni
Lophostoma silvicolum|ABGYE088-06|ROM 111531|Guyana.Potaro-Siparuni
Lophostoma silvicolum|ABGYE077-06|ROM 111520|Guyana.Potaro-Siparuni
Lophostoma silvicolum|ABGYE016-06|ROM 109300|Guyana.Potaro-Siparuni
Lophostoma silvicolum|ABGYD400-06|ROM 108746|Guyana.Potaro-Siparuni
Lophostoma silvicolum|ABGYD080-06|ROM 108377|Guyana.Potaro-Siparuni
Lophostoma silvicolum|ABGYC794-06|ROM 108194|Guyana.Cuyuni-Mazaruni
Lophostoma silvicolum|ABGYC515-06|ROM 107351|Guyana.Potaro-Siparuni
Lophostoma silvicolum|ABGYC343-06|ROM 107155|Guyana.Potaro-Siparuni
Lophostoma silvicolum|ABGYC267-06|ROM 107056|Guyana.Potaro-Siparuni
Lophostoma silvicolum|ABGYC210-06|ROM 106750|Guyana.Upper Takutu-Upper Essequibo
Lophostoma silvicolum|ABGYC182-06|ROM 106716|Guyana.Upper Takutu-Upper Essequibo
Lophostoma silvicolum|ABGYC090-06|ROM 106594|Guyana.Upper Takutu-Upper Essequibo
Lophostoma silvicolum|ABGYC043-06|ROM 104820|Guyana.Potaro-Siparuni
Lophostoma silvicolum|ABGYC023-06|ROM 104800|Guyana.Potaro-Siparuni
Lophostoma silvicolum|ABGYC017-06|ROM 104794|Guyana.Potaro-Siparuni
Lophostoma silvicolum|ABGYB927-06|ROM 104772|Guyana.Potaro-Siparuni
Lophostoma silvicolum|ABGYB869-06|ROM 104713|Guyana.Potaro-Siparuni
Lophostoma silvicolum|ABGYB770-06|ROM 103538|Guyana.Upper Demerara-Berbice
Lophostoma silvicolum|ABGYB691-06|ROM 103455|Guyana.Upper Demerara-Berbice
Lophostoma silvicolum|ABGYB656-06|ROM 103413|Guyana.Upper Demerara-Berbice
Lophostoma silvicolum|ABGYB654-06|ROM 103411|Guyana.Upper Demerara-Berbice
Lophostoma silvicolum|ABGYB464-06|ROM 103198|Guyana.Upper Takutu-Upper Essequibo
Lophostoma silvicolum|ABGYA873-06|ROM 100949|Guyana.Barima-Waini
Lophostoma silvicolum|ABGYA800-06|ROM 100857|Guyana.Barima-Waini
Lophostoma silvicolum|ABGYA317-06|ROM 98714|Guyana.Barima-Waini
Lophostoma silvicolum|ABGYA092-06|ROM 97926|Guyana.Upper Takutu-Upper Essequibo
Lophostoma silvicolum|ABGYA116-06|ROM 97955|Guyana.Upper Takutu-Upper Essequibo
Lophostoma silvicolum|ABGYD582-06|ROM 108962|Guyana.Potaro-Siparuni
Lophostoma silvicolum|ABSMS040-06|ROM 117202|Suriname.Sipaliwini
Lophostoma silvicolum|ABSMS162-06|ROM 117324|Suriname.Sipaliwini
Lophostoma silvicolum|ABGYE844-06|ROM 113458|Guyana.Upper Takutu-Upper Essequibo
Lophostoma silvicolum|BCBNT815-06|ROM 114126|Suriname.Brokopondo
Lophostoma silvicolum|BCBNT835-06|ROM 114185|Suriname.Brokopondo
Lophostoma silvicolum|BCBNT872-06|ROM 115479|Guyana.Essequibo Islands-West Demerara

Lophostoma silvicolum|BCBNT815-06|ROM 114126|Suriname.Brokopondo
Lophostoma silvicolum|BCBNT835-06|ROM 114185|Suriname.Brokopondo
Lophostoma silvicolum|BCBNT872-06|ROM 115479|Guyana.Essequibo Islands-West Demerara
Lophostoma silvicolum|ABGYG087-06|ROM 115626|Guyana.Essequibo Islands-West Demerara
Lophostoma silvicolum|BCBNT923-06|ROM 115604|Guyana.Essequibo Islands-West Demerara
Lophostoma brasiliense|BCBN652-05|ROM 104269|Panama
Lophostoma brasiliense|BCBN335-05|ROM 100917|Guyana.Barima-Waini
Lophostoma brasiliense|ABGYF161-06|ROM 113716|Guyana.Demerara-Mahaica
Lophostoma brasiliense|ABGYF127-06|ROM 113674|Guyana.Demerara-Mahaica
Lophostoma brasiliense|ABGYG719-08|ROM 119147|Guyana
Lophostoma brasiliense|BCBNT133-06|ROM 107393|Guyana.Potaro-Siparuni
Lophostoma brasiliense|ABGYG662-08|ROM 119090|Guyana
Lophostoma brasiliense|BCBNT679-06|ROM 113753|Guyana.Demerara-Mahaica
Lophostoma brasiliense|BCBNT680-06|ROM 113754|Guyana.Demerara-Mahaica
Lophostoma brasiliense|BCBNT031-06|ROM 106608|Guyana.Upper Takutu-Upper Essequibo
Lophostoma brasiliense|ABGYE697-06|ROM 112630|Guyana.Demerara-Mahaica
Lophostoma brasiliense|BCBNT568-06|ROM 112601|Guyana.Demerara-Mahaica
Lophostoma brasiliense|BCBNT684-06|ROM 113766|Guyana.Demerara-Mahaica
Lophostoma brasiliense|BCBNT563-06|ROM 112590|Guyana.Demerara-Mahaica
Lophostoma brasiliense|BCBNT696-06|ROM 113822|Guyana.Demerara-Mahaica
Lophostoma carrikeri|BCBNT787-06|ROM 114041|Suriname.Brokopondo
Lophostoma carrikeri|ABGYC637-06|ROM F39589|Guyana.Potaro-Siparuni
Lophostoma carrikeri|ABGYC623-06|ROM F39318|Guyana.Potaro-Siparuni
Lophostoma carrikeri|ABRMM067-07|ROM F39144|Guyana.Potaro-Siparuni
Lophostoma carrikeri|ABSRA416-06|ROM 117005|Suriname.Nickerie
Lophostoma carrikeri|BCBNT108-06|ROM 107190|Guyana.Potaro-Siparuni
Lophostoma carrikeri|BCBNT148-06|ROM 107461|Guyana.Potaro-Siparuni
Lophostoma carrikeri|ABGYC643-06|ROM F39663|Guyana.Potaro-Siparuni
Lophostoma carrikeri|BCBNT131-06|ROM 107391|Guyana.Potaro-Siparuni
Lophostoma carrikeri|BCBNT132-06|ROM 107392|Guyana.Potaro-Siparuni
Lophostoma carrikeri|BCBNT994-06|ROM 114667|Guyana.Potaro-Siparuni
Rhinophylla alethina|BCBN925-05|ROM 105800|Ecuador.Esmeraldas
Rhinophylla alethina|BCBN908-05|ROM 105780|Ecuador.Esmeraldas
Rhinophylla alethina|BCBN926-05|ROM 105801|Ecuador.Esmeraldas
Rhinophylla pumilio|BCBNT021-06|ROM 106578|Guyana.Upper Takutu-Upper Essequibo
Rhinophylla pumilio|BCBNT064-06|ROM 106759|Guyana.Upper Takutu-Upper Essequibo
Rhinophylla pumilio|ABECA094-06|ROM 104380|Ecuador.Napo
Rhinophylla pumilio|ABECA009-06|ROM 103989|Ecuador.Napo
Rhinophylla pumilio|ABECA026-06|ROM 104011|Ecuador.Napo
Rhinophylla pumilio|ABECA027-06|ROM 104012|Ecuador.Napo
Rhinophylla pumilio|ABECA473-06|ROM 105497|Ecuador.Napo
Rhinophylla pumilio|ABECA697-06|ROM F40050|Ecuador.Napo
Rhinophylla pumilio|ABECB072-08|ROM 118734|Ecuador
Rhinophylla pumilio|BCBN716-05|ROM 104377|Ecuador.Napo
Rhinophylla pumilio|BCBN717-05|ROM 104378|Ecuador.Napo
Rhinophylla pumilio|ABECA263-06|ROM 105131|Ecuador.Napo
Rhinophylla pumilio|ABECA474-06|ROM F37725|Ecuador.Napo
Rhinophylla pumilio|ABECA288-06|ROM 105159|Ecuador.Napo
Rhinophylla pumilio|ABECA733-06|ROM 105879|Ecuador.Napo
Rhinophylla pumilio|ABECA752-06|ROM 105901|Ecuador.Napo
Rhinophylla pumilio|ABECA066-06|ROM F37167|Ecuador.Napo
Rhinophylla pumilio|ABECA520-06|ROM 105552|Ecuador.Napo
Rhinophylla pumilio|ABECA914-06|ROM F40351|Ecuador.Napo
Rhinophylla pumilio|ABECA174-06|ROM F37329|Ecuador.Napo
Rhinophylla pumilio|ABECA251-06|ROM F37427|Ecuador.Napo
Rhinophylla pumilio|ABECA913-06|ROM 106094|Ecuador.Napo
Rhinophylla pumilio|ABECA095-06|ROM F37210|Ecuador.Napo
Rhinophylla pumilio|ABECA272-06|ROM 105141|Ecuador.Napo
Rhinophylla pumilio|ABECA093-06|ROM 104379|Ecuador.Napo
Rhinophylla pumilio|ABECA320-06|ROM 105200|Ecuador.Napo
Rhinophylla pumilio|ABECA737-06|ROM 105883|Ecuador.Napo
Rhinophylla pumilio|ABECA958-06|ROM F40407|Ecuador.Napo
Rhinophylla pumilio|BCBN735-05|ROM 104424|Ecuador.Napo
Rhinophylla pumilio|ABECA538-06|ROM 105566|Ecuador.Napo
Rhinophylla pumilio|ABECA252-06|ROM 105120|Ecuador.Napo
Rhinophylla pumilio|ABECA250-06|ROM 105119|Ecuador.Napo
Rhinophylla pumilio|ABECA148-06|ROM 104450|Ecuador.Napo
Rhinophylla pumilio|ABECA029-06|ROM F37104|Ecuador.Napo
Rhinophylla pumilio|ABECA028-06|ROM F37103|Ecuador.Napo
Rhinophylla pumilio|ABECA012-06|ROM 103991|Ecuador.Napo
Rhinophylla pumilio|ABECA577-06|ROM 105607|Ecuador.Napo
Rhinophylla pumilio|ABECA578-06|ROM 105608|Ecuador.Napo
Rhinophylla pumilio|ABECA698-06|ROM 105758|Ecuador.Napo
Rhinophylla pumilio|ABECA738-06|ROM 105884|Ecuador.Napo
Rhinophylla pumilio|ABECA766-06|ROM 105920|Ecuador.Napo
Rhinophylla pumilio|ABECA767-06|ROM 105921|Ecuador.Napo
Rhinophylla pumilio|ABECA912-06|ROM F40349|Ecuador.Napo
Rhinophylla pumilio|BCBN779-05|ROM 105153|Ecuador.Napo
Rhinophylla pumilio|BCBN980-05|ROM 106079|Ecuador.Napo
Rhinophylla pumilio|ABGYB249-06|ROM 102953|Guyana.Upper Takutu-Upper Essequibo
Rhinophylla pumilio|ABGYB482-06|ROM 103217|Guyana.Upper Takutu-Upper Essequibo
Rhinophylla pumilio|ABGYB512-06|ROM 103247|Guyana.Upper Takutu-Upper Essequibo
Rhinophylla pumilio|BCBNT631-06|ROM 113573|Guyana.Upper Takutu-Upper Essequibo
Rhinophylla pumilio|ABGYE425-06|ROM 111883|Guyana.Potaro-Siparuni
Rhinophylla pumilio|ABSMS122-06|ROM 117284|Suriname.Sipaliwini
Rhinophylla pumilio|ABGYC419-06|ROM 107241|Guyana.Potaro-Siparuni
Rhinophylla pumilio|ABGYC318-06|ROM 107130|Guyana.Potaro-Siparuni
Rhinophylla pumilio|ABGYA257-06|ROM 98157|Guyana.Potaro-Siparuni
Rhinophylla pumilio|ABGYC558-06|ROM 107402|Guyana.Potaro-Siparuni
Rhinophylla pumilio|ABGYD057-06|ROM 108353|Guyana.Potaro-Siparuni
Rhinophylla pumilio|ABGYD176-06|ROM 108502|Guyana.Potaro-Siparuni
Rhinophylla pumilio|ABGYD214-06|ROM 108545|Guyana.Potaro-Siparuni
Rhinophylla pumilio|ABGYB826-06|ROM 104667|Guyana.Potaro-Siparuni
Rhinophylla pumilio|ABGYE415-06|ROM 111870|Guyana.Potaro-Siparuni
Rhinophylla pumilio|ABGYE405-06|ROM 111858|Guyana.Potaro-Siparuni
Rhinophylla pumilio|ABGYE296-06|ROM 111746|Guyana.Potaro-Siparuni
Rhinophylla pumilio|ABGYE251-06|ROM 111700|Guyana.Potaro-Siparuni
Rhinophylla pumilio|ABGYE243-06|ROM 111691|Guyana.Potaro-Siparuni
Rhinophylla pumilio|ABGYD697-06|ROM 109110|Guyana.Potaro-Siparuni
Rhinophylla pumilio|ABGYD237-06|ROM 108569|Guyana.Potaro-Siparuni
Rhinophylla pumilio|ABGYC662-06|ROM 107482|Guyana.Potaro-Siparuni
Rhinophylla pumilio|ABGYC588-06|ROM 107443|Guyana.Potaro-Siparuni
Rhinophylla pumilio|ABGYC382-06|ROM 107199|Guyana.Potaro-Siparuni

[illegible]
Rhinophylla pumilio|ABGYC662-06|ROM 107482|Guyana.Potaro-Siparuni
Rhinophylla pumilio|ABGYC588-06|ROM 107443|Guyana.Potaro-Siparuni
Rhinophylla pumilio|ABGYC382-06|ROM 107199|Guyana.Potaro-Siparuni
Rhinophylla pumilio|ABGYB865-06|ROM 104709|Guyana.Potaro-Siparuni
Rhinophylla pumilio|ABGYB764-06|ROM 103532|Guyana.Upper Demerara-Berbice
Rhinophylla pumilio|ABGYB440-06|ROM 103154|Guyana.Upper Takutu-Upper Essequibo
Rhinophylla pumilio|ABGYB422-06|ROM 103130|Guyana.Upper Takutu-Upper Essequibo
Rhinophylla pumilio|ABGYB265-06|ROM 102969|Guyana.Upper Takutu-Upper Essequibo
Rhinophylla pumilio|ABGYA256-06|ROM 98156|Guyana.Potaro-Siparuni
Rhinophylla pumilio|ABGYE292-06|ROM 111741|Guyana.Potaro-Siparuni
Rhinophylla pumilio|ABGYE416-06|ROM 111871|Guyana.Potaro-Siparuni
Rhinophylla pumilio|ABGYE436-06|ROM 111896|Guyana.Potaro-Siparuni
Rhinophylla pumilio|ABGYE451-06|ROM 111914|Guyana.Potaro-Siparuni
Rhinophylla pumilio|ABGYE478-06|ROM 111950|Guyana.Potaro-Siparuni
Rhinophylla pumilio|ABGYE502-06|ROM 111974|Guyana.Potaro-Siparuni
Rhinophylla pumilio|ABGYD129-06|ROM 108432|Guyana.Potaro-Siparuni
Rhinophylla pumilio|ABGYC590-06|ROM 107446|Guyana.Potaro-Siparuni
Rhinophylla pumilio|ABGYE605-06|ROM 112084|Guyana.Potaro-Siparuni
Rhinophylla pumilio|ABGYF085-06|ROM 113616|Guyana.Demerara-Mahaica
Rhinophylla pumilio|ABGYG093-06|ROM 115634|Guyana.Essequibo Islands-West Demerara
Rhinophylla pumilio|BCBNT643-06|ROM 113596|Guyana.Upper Takutu-Upper Essequibo
Rhinophylla pumilio|ABGYF178-06|ROM 113735|Guyana.Demerara-Mahaica
Rhinophylla pumilio|ABGYD421-06|ROM 108771|Guyana.Potaro-Siparuni
Rhinophylla pumilio|ABGYE226-06|ROM 111674|Guyana.Potaro-Siparuni
Rhinophylla pumilio|ABGYE434-06|ROM 111894|Guyana.Potaro-Siparuni
Rhinophylla pumilio|ABGYF192-06|ROM 113756|Guyana.Demerara-Mahaica
Rhinophylla pumilio|ABGYB848-06|ROM 104690|Guyana.Potaro-Siparuni
Rhinophylla pumilio|ABGYB911-06|ROM 104756|Guyana.Potaro-Siparuni
Rhinophylla pumilio|ABGYC248-06|ROM 107029|Guyana.Potaro-Siparuni
Rhinophylla pumilio|ABGYC362-06|ROM 107178|Guyana.Potaro-Siparuni
Rhinophylla pumilio|ABGYD076-06|ROM 108373|Guyana.Potaro-Siparuni
Rhinophylla pumilio|ABGYD099-06|ROM 108397|Guyana.Potaro-Siparuni
Rhinophylla pumilio|ABGYD238-06|ROM 108570|Guyana.Potaro-Siparuni
Rhinophylla pumilio|ABGYE190-06|ROM 111636|Guyana.Potaro-Siparuni
Rhinophylla pumilio|ABGYE206-06|ROM 111653|Guyana.Potaro-Siparuni
Rhinophylla pumilio|ABGYE742-06|ROM 112685|Guyana.Demerara-Mahaica
Rhinophylla pumilio|ABSMS252-06|ROM 117414|Suriname.Sipaliwini
Rhinophylla pumilio|BCBN156-05|ROM 98204|Guyana.Demerara-Mahaica
Rhinophylla pumilio|BCBN157-05|ROM 98205|Guyana.Demerara-Mahaica
Rhinophylla pumilio|BCBN159-05|ROM 98713|Guyana.Barima-Waini
Rhinophylla pumilio|BCBN171-05|ROM 98857|Guyana.Barima-Waini
Rhinophylla pumilio|BCBNT044-06|ROM 106676|Guyana.Upper Takutu-Upper Essequibo
Rhinophylla pumilio|BCBNT564-06|ROM 112594|Guyana.Demerara-Mahaica
Rhinophylla pumilio|BCBNT784-06|ROM 114030|Suriname.Brokopondo
Rhinophylla pumilio|BCBNT871-06|ROM 115476|Guyana.Essequibo Islands-West Demerara
Rhinophylla pumilio|BCBNT786-06|ROM 114035|Suriname.Brokopondo
Rhinophylla pumilio|ABGYE242-06|ROM 111690|Guyana.Potaro-Siparuni
Rhinophylla pumilio|ABGYC334-06|ROM 107146|Guyana.Potaro-Siparuni
Rhinophylla pumilio|ABGYB912-06|ROM 104757|Guyana.Potaro-Siparuni
Rhinophylla pumilio|ABGYD130-06|ROM 108433|Guyana.Potaro-Siparuni
Rhinophylla pumilio|ABGYD175-06|ROM 108496|Guyana.Potaro-Siparuni
Rhinophylla pumilio|BCBNT296-06|ROM 108500|Guyana.Potaro-Siparuni
Rhinophylla pumilio|ABGYC052-06|ROM 104829|Guyana.Potaro-Siparuni
Rhinophylla pumilio|ABGYB820-06|ROM 104661|Guyana.Potaro-Siparuni
Rhinophylla pumilio|ABGYC335-06|ROM 107147|Guyana.Potaro-Siparuni
Rhinophylla pumilio|BCBNT071-06|ROM 106789|Guyana.Upper Takutu-Upper Essequibo
Rhinophylla pumilio|BCBNT293-06|ROM 108497|Guyana.Potaro-Siparuni
Rhinophylla pumilio|BCBNT053-06|ROM 106701|Guyana.Upper Takutu-Upper Essequibo
Rhinophylla pumilio|ABGYE503-06|ROM 111975|Guyana.Potaro-Siparuni
Rhinophylla pumilio|ABGYE437-06|ROM 111897|Guyana.Potaro-Siparuni
Rhinophylla pumilio|ABGYE280-06|ROM 111729|Guyana.Potaro-Siparuni
Rhinophylla pumilio|ABGYE279-06|ROM 111728|Guyana.Potaro-Siparuni
Rhinophylla pumilio|ABGYE265-06|ROM 111714|Guyana.Potaro-Siparuni
Rhinophylla pumilio|ABGYE150-06|ROM 111593|Guyana.Potaro-Siparuni
Rhinophylla pumilio|ABGYD422-06|ROM 108772|Guyana.Potaro-Siparuni
Rhinophylla pumilio|ABGYD239-06|ROM 108571|Guyana.Potaro-Siparuni
Rhinophylla pumilio|ABGYD097-06|ROM 108395|Guyana.Potaro-Siparuni
Rhinophylla pumilio|ABGYD091-06|ROM 108388|Guyana.Potaro-Siparuni
Rhinophylla pumilio|ABGYC564-06|ROM 107409|Guyana.Potaro-Siparuni
Rhinophylla pumilio|ABGYC344-06|ROM 107156|Guyana.Potaro-Siparuni
Rhinophylla pumilio|ABGYC333-06|ROM 107145|Guyana.Potaro-Siparuni
Rhinophylla pumilio|ABGYB519-06|ROM 103254|Guyana.Upper Takutu-Upper Essequibo
Rhinophylla pumilio|BCBNT621-06|ROM 113537|Guyana.Upper Takutu-Upper Essequibo
Rhinophylla pumilio|BCBNT845-06|ROM 114209|Suriname.Brokopondo
Rhinophylla pumilio|BCBN352-05|ROM 100997|Guyana.Barima-Waini
Rhinophylla pumilio|BCBNT557-06|ROM 112566|Guyana.Demerara-Mahaica
Rhinophylla pumilio|ABGYE327-06|ROM 111777|Guyana.Potaro-Siparuni
Rhinophylla pumilio|ABGYF087-06|ROM 113618|Guyana.Demerara-Mahaica
Rhinophylla pumilio|ABGYB012-06|ROM 100980|Guyana.Barima-Waini
Rhinophylla pumilio|BCBNT256-06|ROM 108209|Guyana.Cuyuni-Mazaruni
Rhinophylla pumilio|ABGYF193-06|ROM 113757|Guyana.Demerara-Mahaica
Rhinophylla pumilio|ABGYE622-06|ROM 112533|Guyana.Demerara-Mahaica
Rhinophylla pumilio|ABGYE632-06|ROM 112545|Guyana.Demerara-Mahaica
Rhinophylla pumilio|ABGYE722-06|ROM 112657|Guyana.Demerara-Mahaica
Rhinophylla pumilio|ABGYF150-06|ROM 113703|Guyana.Demerara-Mahaica
Rhinophylla pumilio|ABGYF205-06|ROM 113775|Guyana.Demerara-Mahaica
Rhinophylla pumilio|ABGYG018-06|ROM 115504|Guyana.Essequibo Islands-West Demerara
Rhinophylla pumilio|ABGYG020-06|ROM 115506|Guyana.Essequibo Islands-West Demerara
Rhinophylla pumilio|ABGYG021-06|ROM 115507|Guyana.Essequibo Islands-West Demerara
Rhinophylla pumilio|ABGYG086-06|ROM 115625|Guyana.Essequibo Islands-West Demerara
Rhinophylla pumilio|BCBNT570-06|ROM 112613|Guyana.Demerara-Mahaica
Rhinophylla pumilio|BCBNT917-06|ROM 115591|Guyana.Essequibo Islands-West Demerara
Rhinophylla pumilio|ABGYD131-06|ROM 108434|Guyana.Potaro-Siparuni
Rhinophylla pumilio|ABGYG412-06|ROM 116544|Guyana.Potaro-Siparuni
Rhinophylla pumilio|ABGYG022-06|ROM 115508|Guyana.Essequibo Islands-West Demerara
Rhinophylla pumilio|ABSRA352-06|ROM 116941|Suriname.Nickerie
Rhinophylla pumilio|ABGYC813-06|ROM 108215|Guyana.Cuyuni-Mazaruni
Rhinophylla pumilio|ABGYC767-06|ROM F43196|Guyana.Cuyuni-Mazaruni
Rhinophylla pumilio|ABGYC753-06|ROM F43176|Guyana.Cuyuni-Mazaruni
Rhinophylla pumilio|ABGYC748-06|ROM F43171|Guyana.Cuyuni-Mazaruni
Rhinophylla pumilio|BCBNC064-06|ROM 115169|Guyana.Cuyuni-Mazaruni
Rhinophylla pumilio|BCBNT263-06|ROM 108234|Guyana.Cuyuni-Mazaruni
[illegible]

Rhinophylla pumilio|ABGYC748-06|ROM [illegible]|Guyana.Cuyuni-Mazaruni
Rhinophylla pumilio|BCBNC064-06|ROM 115169|Guyana.Cuyuni-Mazaruni
Rhinophylla pumilio|BCBNT263-06|ROM 108234|Guyana.Cuyuni-Mazaruni
Rhinophylla pumilio|BCBNT265-06|ROM 108246|Guyana.Cuyuni-Mazaruni
Rhinophylla pumilio|ABGYD100-06|ROM 108398|Guyana.Potaro-Siparuni
Rhinophylla pumilio|ABGYE404-06|ROM 111857|Guyana.Potaro-Siparuni
Rhinophylla pumilio|ABGYB461-06|ROM 103195|Guyana.Upper Takutu-Upper Essequibo
Rhinophylla pumilio|ABGYB762-06|ROM 103530|Guyana.Upper Demerara-Berbice
Rhinophylla pumilio|BCBNT820-06|ROM 114135|Suriname.Brokopondo
Rhinophylla pumilio|ABGYF179-06|ROM 113736|Guyana.Demerara-Mahaica
Rhinophylla pumilio|ABGYF116-06|ROM 113659|Guyana.Demerara-Mahaica
Rhinophylla pumilio|ABGYF676-06|ROM 115477|Guyana.Essequibo Islands-West Demerara
Rhinophylla pumilio|ABGYG032-06|ROM 115538|Guyana.Essequibo Islands-West Demerara
Rhinophylla pumilio|BCBNT294-06|ROM 108498|Guyana.Potaro-Siparuni
Rhinophylla pumilio|BCBN545-05|ROM 103400|Guyana.Upper Demerara-Berbice
Rhinophylla pumilio|BCBN314-05|ROM 100368|Guyana.East Berbice-Corentyne
Rhinophylla pumilio|BCBN313-05|ROM 100367|Guyana.East Berbice-Corentyne
Rhinophylla pumilio|BCBN312-05|ROM 100366|Guyana.East Berbice-Corentyne
Rhinophylla pumilio|BCBN298-05|ROM 100277|Guyana.East Berbice-Corentyne
Rhinophylla pumilio|BCBN297-05|ROM 100276|Guyana.East Berbice-Corentyne
Rhinophylla pumilio|BCBN158-05|ROM 98206|Guyana.Demerara-Mahaica
Rhinophylla pumilio|ABSRA355-06|ROM 116944|Suriname.Nickerie
Rhinophylla pumilio|ABGYG150-06|ROM 115710|Guyana.Potaro-Siparuni
Rhinophylla pumilio|ABGYG1033-08|ROM 118957|Guyana
Rhinophylla pumilio|ABGYG083-06|ROM 115618|Guyana.Essequibo Islands-West Demerara
Rhinophylla pumilio|ABGYG077-06|ROM 115610|Guyana.Essequibo Islands-West Demerara
Rhinophylla pumilio|ABGYG019-06|ROM 115505|Guyana.Essequibo Islands-West Demerara
Rhinophylla pumilio|ABGYF117-06|ROM 113660|Guyana.Demerara-Mahaica
Rhinophylla pumilio|ABGYF086-06|ROM 113617|Guyana.Demerara-Mahaica
Rhinophylla pumilio|ABGYF084-06|ROM 113615|Guyana.Demerara-Mahaica
Rhinophylla pumilio|ABGYE732-06|ROM 112672|Guyana.Demerara-Mahaica
Rhinophylla pumilio|ABGYE650-06|ROM 112565|Guyana.Demerara-Mahaica
Rhinophylla pumilio|ABGYE649-06|ROM 112564|Guyana.Demerara-Mahaica
Rhinophylla pumilio|ABGYE646-06|ROM 112561|Guyana.Demerara-Mahaica
Rhinophylla pumilio|ABGYE619-06|ROM 112527|Guyana.Demerara-Mahaica
Rhinophylla pumilio|ABGYE606-06|ROM 112085|Guyana.Potaro-Siparuni
Rhinophylla pumilio|ABGYE533-06|ROM 112006|Guyana.Potaro-Siparuni
Rhinophylla pumilio|ABGYE435-06|ROM 111895|Guyana.Potaro-Siparuni
Rhinophylla pumilio|ABGYE291-06|ROM 111740|Guyana.Potaro-Siparuni
Rhinophylla pumilio|ABGYE264-06|ROM 111713|Guyana.Potaro-Siparuni
Rhinophylla pumilio|ABGYE252-06|ROM 111701|Guyana.Potaro-Siparuni
Rhinophylla pumilio|ABGYE241-06|ROM 111689|Guyana.Potaro-Siparuni
Rhinophylla pumilio|ABGYD384-06|ROM 108729|Guyana.Potaro-Siparuni
Rhinophylla pumilio|ABGYD383-06|ROM 108728|Guyana.Potaro-Siparuni
Rhinophylla pumilio|ABGYD101-06|ROM 108399|Guyana.Potaro-Siparuni
Rhinophylla pumilio|ABGYD058-06|ROM 108354|Guyana.Potaro-Siparuni
Rhinophylla pumilio|ABGYC663-06|ROM 107483|Guyana.Potaro-Siparuni
Rhinophylla pumilio|ABGYC661-06|ROM 107481|Guyana.Potaro-Siparuni
Rhinophylla pumilio|ABGYC576-06|ROM 107424|Guyana.Potaro-Siparuni
Rhinophylla pumilio|ABGYC543-06|ROM 107382|Guyana.Potaro-Siparuni
Rhinophylla pumilio|ABGYC317-06|ROM 107129|Guyana.Potaro-Siparuni
Rhinophylla pumilio|ABGYC316-06|ROM 107128|Guyana.Potaro-Siparuni
Rhinophylla pumilio|ABGYB910-06|ROM 104755|Guyana.Potaro-Siparuni
Rhinophylla pumilio|ABGYB835-06|ROM 104676|Guyana.Potaro-Siparuni
Rhinophylla pumilio|ABGYB828-06|ROM 104669|Guyana.Potaro-Siparuni
Rhinophylla pumilio|ABGYB827-06|ROM 104668|Guyana.Potaro-Siparuni
Rhinophylla pumilio|ABGYB824-06|ROM 104665|Guyana.Potaro-Siparuni
Rhinophylla pumilio|ABGYB730-06|ROM 103497|Guyana.Upper Demerara-Berbice
Rhinophylla pumilio|ABGYB443-06|ROM 103157|Guyana.Upper Takutu-Upper Essequibo
Rhinophylla pumilio|ABGYB264-06|ROM 102968|Guyana.Upper Takutu-Upper Essequibo
Rhinophylla pumilio|ABGYC353-06|ROM 107165|Guyana.Potaro-Siparuni
Rhinophylla pumilio|ABGYC418-06|ROM 107240|Guyana.Potaro-Siparuni
Rhinophylla pumilio|ABGYF206-06|ROM 113776|Guyana.Demerara-Mahaica
Rhinophylla pumilio|ABGYC579-06|ROM 107427|Guyana.Potaro-Siparuni
Rhinophylla pumilio|ABGYC650-06|ROM 107468|Guyana.Potaro-Siparuni
Rhinophylla pumilio|ABGYE647-06|ROM 112562|Guyana.Demerara-Mahaica
Rhinophylla pumilio|ABGYE648-06|ROM 112563|Guyana.Demerara-Mahaica
Rhinophylla pumilio|BCBNT295-06|ROM 108499|Guyana.Potaro-Siparuni
Rhinophylla pumilio|BCBNT297-06|ROM 108501|Guyana.Potaro-Siparuni
Rhinophylla pumilio|BCBNT558-06|ROM 112567|Guyana.Demerara-Mahaica
Rhinophylla pumilio|BCBNT589-06|ROM 113409|Guyana.Upper Demerara-Berbice
Rhinophylla pumilio|BCBNT590-06|ROM 113416|Guyana.Upper Demerara-Berbice
Rhinophylla pumilio|BCBNT593-06|ROM 113430|Guyana.Upper Demerara-Berbice
Rhinophylla pumilio|BCBNT608-06|ROM 113507|Guyana.Upper Takutu-Upper Essequibo
Rhinophylla pumilio|BCBNT744-06|ROM 113923|Suriname.Brokopondo
Rhinophylla pumilio|BCBNT881-06|ROM 115509|Guyana.Essequibo Islands-West Demerara
Rhinophylla pumilio|ABGYE711-06|ROM 112646|Guyana.Demerara-Mahaica
Rhinophylla pumilio|BCBNT574-06|ROM 112633|Guyana.Demerara-Mahaica
Rhinophylla pumilio|BCBNT927-06|ROM 115609|Guyana.Essequibo Islands-West Demerara
Rhinophylla pumilio|ABGYF288-06|ROM 114648|Guyana.Potaro-Siparuni
Rhinophylla pumilio|ABGYF295-06|ROM 114658|Guyana.Potaro-Siparuni
Rhinophylla pumilio|ABGYA158-06|ROM 98014|Guyana.Potaro-Siparuni
Rhinophylla pumilio|ABGYC751-06|ROM 108152|Guyana.Cuyuni-Mazaruni
Rhinophylla pumilio|ABGYF308-06|ROM 114677|Guyana.Potaro-Siparuni
Rhinophylla pumilio|ABGYF310-06|ROM 114682|Guyana.Potaro-Siparuni
Rhinophylla pumilio|ABGYF311-06|ROM 114683|Guyana.Potaro-Siparuni
Rhinophylla pumilio|ABGYF318-06|ROM 114708|Guyana.Potaro-Siparuni
Rhinophylla pumilio|ABGYF492-06|ROM 115131|Guyana.Cuyuni-Mazaruni
Rhinophylla pumilio|ABGYG411-06|ROM 116543|Guyana.Potaro-Siparuni
Rhinophylla pumilio|ABGYG415-06|ROM 116547|Guyana.Potaro-Siparuni
Rhinophylla pumilio|BCBNC061-06|ROM 115135|Guyana.Cuyuni-Mazaruni
Rhinophylla pumilio|ABGYG416-06|ROM 116548|Guyana.Potaro-Siparuni
Rhinophylla pumilio|ABGYG410-06|ROM 116542|Guyana.Potaro-Siparuni
Rhinophylla pumilio|BCBNC048-06|ROM 115066|Guyana.Cuyuni-Mazaruni
Rhinophylla pumilio|BCBNC067-06|ROM 115172|Guyana.Cuyuni-Mazaruni
Rhinophylla pumilio|ABGYF312-06|ROM 114694|Guyana.Potaro-Siparuni
Rhinophylla pumilio|BCBNC179-06|ROM 116513|Guyana.Potaro-Siparuni
Rhinophylla pumilio|BCBNC180-06|ROM 116521|Guyana.Potaro-Siparuni
Rhinophylla pumilio|BCBNC046-06|ROM 114802|Guyana.Potaro-Siparuni
Rhinophylla pumilio|BCBNC054-06|ROM 115076|Guyana.Cuyuni-Mazaruni
Rhinophylla pumilio|ABGYF475-06|ROM 115112|Guyana.Cuyuni-Mazaruni
Rhinophylla pumilio|ABGYF331-06|ROM 114726|Guyana.Potaro-Siparuni
Rhinophylla pumilio|ABGYF316-06|ROM 114706|Guyana.Potaro-Siparuni
[illegible]

Rhinophylla pumilio|ABGYF475-06|ROM 115112|Guyana.Cuyuni-Mazaruni
Rhinophylla pumilio|ABGYF331-06|ROM 114726|Guyana.Potaro-Siparuni
Rhinophylla pumilio|ABGYF316-06|ROM 114706|Guyana.Potaro-Siparuni
Rhinophylla pumilio|ABGYF306-06|ROM 114675|Guyana.Potaro-Siparuni
Rhinophylla pumilio|ABGYE053-06|ROM 109339|Guyana.Potaro-Siparuni
Rhinophylla pumilio|ABGYC750-06|ROM 108151|Guyana.Cuyuni-Mazaruni
Rhinophylla pumilio|ABGYC714-06|ROM 108114|Guyana.Cuyuni-Mazaruni
Rhinophylla pumilio|ABGYB829-06|ROM 104670|Guyana.Potaro-Siparuni
Rhinophylla pumilio|ABGYG212-06|ROM 115810|Guyana.Potaro-Siparuni
Rhinophylla pumilio|ABGYG372-06|ROM 116495|Guyana.Potaro-Siparuni
Rhinophylla pumilio|ABGYG383-06|ROM 116506|Guyana.Potaro-Siparuni
Rhinophylla pumilio|ABGYG414-06|ROM 116546|Guyana.Potaro-Siparuni
Rhinophylla pumilio|BCBNC185-06|ROM 116541|Guyana.Potaro-Siparuni
Rhinophylla pumilio|ABGYG391-06|ROM 116517|Guyana.Potaro-Siparuni
Rhinophylla pumilio|BCBNC138-06|ROM 115808|Guyana.Potaro-Siparuni
Rhinophylla pumilio|ABGYG198-06|ROM 115782|Guyana.Potaro-Siparuni
Rhinophylla pumilio|ABGYC752-06|ROM F43175|Guyana.Cuyuni-Mazaruni
Rhinophylla pumilio|ABGYA316-06|ROM 98712|Guyana.Barima-Waini
Rhinophylla pumilio|BCBNT257-06|ROM 108213|Guyana.Cuyuni-Mazaruni
Rhinophylla pumilio|BCBNC139-06|ROM 115809|Guyana.Potaro-Siparuni
Rhinophylla pumilio|ABGYC784-06|ROM 108185|Guyana.Cuyuni-Mazaruni
Rhinophylla pumilio|ABGYD850-06|ROM 109277|Guyana.Potaro-Siparuni
Rhinophylla pumilio|ABGYC783-06|ROM 108184|Guyana.Cuyuni-Mazaruni
Rhinophylla pumilio|ABGYC724-06|ROM 108124|Guyana.Cuyuni-Mazaruni
Rhinophylla pumilio|ABGYC715-06|ROM 108115|Guyana.Cuyuni-Mazaruni
Rhinophylla pumilio|ABGYC709-06|ROM 108108|Guyana.Cuyuni-Mazaruni
Rhinophylla pumilio|ABGYC697-06|ROM 108097|Guyana.Cuyuni-Mazaruni
Rhinophylla pumilio|ABGYC548-06|ROM 107387|Guyana.Potaro-Siparuni
Rhinophylla pumilio|ABGYC310-06|ROM 107121|Guyana.Potaro-Siparuni
Rhinophylla pumilio|ABGYB891-06|ROM 104736|Guyana.Potaro-Siparuni
Rhinophylla pumilio|ABGYB026-06|ROM 100998|Guyana.Barima-Waini
Rhinophylla pumilio|ABGYA380-06|ROM 98784|Guyana.Barima-Waini
Rhinophylla pumilio|ABGYA315-06|ROM 98711|Guyana.Barima-Waini
Rhinophylla pumilio|ABGYA159-06|ROM 98015|Guyana.Potaro-Siparuni
Rhinophylla pumilio|ABGYD098-06|ROM 108396|Guyana.Potaro-Siparuni
Rhinophylla pumilio|ABGYE025-06|ROM 109309|Guyana.Potaro-Siparuni
Rhinophylla pumilio|ABGYC749-06|ROM 108150|Guyana.Cuyuni-Mazaruni
Rhinophylla pumilio|ABGYF455-06|ROM 115091|Guyana.Cuyuni-Mazaruni
Rhinophylla pumilio|ABGYG187-06|ROM 115756|Guyana.Potaro-Siparuni
Rhinophylla pumilio|ABGYG389-06|ROM 116515|Guyana.Potaro-Siparuni
Rhinophylla pumilio|ABGYC785-06|ROM F43217|Guyana.Cuyuni-Mazaruni
Rhinophylla pumilio|ABGYC786-06|ROM F43218|Guyana.Cuyuni-Mazaruni
Rhinophylla pumilio|ABGYD025-06|ROM 108245|Guyana.Cuyuni-Mazaruni
Rhinophylla pumilio|ABGYD426-06|ROM 108776|Guyana.Potaro-Siparuni
Rhinophylla pumilio|ABGYD475-06|ROM 108826|Guyana.Potaro-Siparuni
Rhinophylla pumilio|ABGYF298-06|ROM 114666|Guyana.Potaro-Siparuni
Rhinophylla pumilio|ABGYF307-06|ROM 114676|Guyana.Potaro-Siparuni
Rhinophylla pumilio|ABGYF315-06|ROM 114705|Guyana.Potaro-Siparuni
Rhinophylla pumilio|ABGYF474-06|ROM 115111|Guyana.Cuyuni-Mazaruni
Rhinophylla pumilio|ABGYF484-06|ROM 115122|Guyana.Cuyuni-Mazaruni
Rhinophylla pumilio|ABGYF491-06|ROM 115130|Guyana.Cuyuni-Mazaruni
Rhinophylla pumilio|ABGYF493-06|ROM 115132|Guyana.Cuyuni-Mazaruni
Rhinophylla pumilio|ABGYF494-06|ROM 115133|Guyana.Cuyuni-Mazaruni
Rhinophylla pumilio|ABGYG084-06|ROM 115619|Guyana.Essequibo Islands-West Demerara
Rhinophylla pumilio|ABGYG177-06|ROM 115739|Guyana.Potaro-Siparuni
Rhinophylla pumilio|ABGYG188-06|ROM 115757|Guyana.Potaro-Siparuni
Rhinophylla pumilio|ABGYG189-06|ROM 115758|Guyana.Potaro-Siparuni
Rhinophylla pumilio|ABGYG190-06|ROM 115759|Guyana.Potaro-Siparuni
Rhinophylla pumilio|ABGYG371-06|ROM 116494|Guyana.Potaro-Siparuni
Rhinophylla pumilio|ABGYG373-06|ROM 116496|Guyana.Potaro-Siparuni
Rhinophylla pumilio|ABGYG387-06|ROM 116510|Guyana.Potaro-Siparuni
Rhinophylla pumilio|ABGYG413-06|ROM 116545|Guyana.Potaro-Siparuni
Rhinophylla pumilio|ABGYG435-06|ROM 116570|Guyana.Potaro-Siparuni
Rhinophylla pumilio|BCBN161-05|ROM 98732|Guyana.Barima-Waini
Rhinophylla pumilio|BCBN549-05|ROM 103426|Guyana.Upper Demerara-Berbice
Rhinophylla pumilio|BCBNC014-06|ROM 114709|Guyana.Potaro-Siparuni
Rhinophylla pumilio|BCBNC019-06|ROM 114727|Guyana.Potaro-Siparuni
Rhinophylla pumilio|BCBNC026-06|ROM 114738|Guyana.Potaro-Siparuni
Rhinophylla pumilio|BCBNC049-06|ROM 115067|Guyana.Cuyuni-Mazaruni
Rhinophylla pumilio|BCBNC050-06|ROM 115068|Guyana.Cuyuni-Mazaruni
Rhinophylla pumilio|BCBNC059-06|ROM 115090|Guyana.Cuyuni-Mazaruni
Rhinophylla pumilio|BCBNC062-06|ROM 115136|Guyana.Cuyuni-Mazaruni
Rhinophylla pumilio|BCBNC068-06|ROM 115173|Guyana.Cuyuni-Mazaruni
Rhinophylla pumilio|BCBNC069-06|ROM 115196|Guyana.Cuyuni-Mazaruni
Rhinophylla pumilio|BCBNC128-06|ROM 115794|Guyana.Potaro-Siparuni
Rhinophylla pumilio|BCBNC129-06|ROM 115795|Guyana.Potaro-Siparuni
Rhinophylla pumilio|BCBNC134-06|ROM 115803|Guyana.Potaro-Siparuni
Rhinophylla pumilio|BCBNC177-06|ROM 116511|Guyana.Potaro-Siparuni
Rhinophylla pumilio|BCBNC178-06|ROM 116512|Guyana.Potaro-Siparuni
Rhinophylla pumilio|BCBNT258-06|ROM 108214|Guyana.Cuyuni-Mazaruni
Rhinophylla pumilio|BCBNT929-06|ROM 115617|Guyana.Essequibo Islands-West Demerara
Rhinophylla pumilio|BCBNT963-06|ROM 114873|Guyana.Potaro-Siparuni
Rhinophylla pumilio|BCBNT970-06|ROM 114617|Guyana.Potaro-Siparuni
Rhinophylla pumilio|BCBNT975-06|ROM 114628|Guyana.Potaro-Siparuni
Rhinophylla pumilio|BCBNT985-06|ROM 114646|Guyana.Potaro-Siparuni
Rhinophylla pumilio|BCBNT986-06|ROM 114647|Guyana.Potaro-Siparuni
Rhinophylla pumilio|ABGYB444-06|ROM 103158|Guyana.Upper Takutu-Upper Essequibo
Rhinophylla pumilio|ABGYD077-06|ROM 108374|Guyana.Potaro-Siparuni
Rhinophylla pumilio|ABGYD331-06|ROM 108671|Guyana.Potaro-Siparuni
Rhinophylla pumilio|ABGYE250-06|ROM 111699|Guyana.Potaro-Siparuni
Rhinophylla pumilio|ABGYG197-06|ROM 115781|Guyana.Potaro-Siparuni
Rhinophylla pumilio|BCBNC007-06|ROM 114693|Guyana.Potaro-Siparuni
Rhinophylla pumilio|BCBNC013-06|ROM 114704|Guyana.Potaro-Siparuni
Rhinophylla pumilio|ABGYD262-06|ROM 108598|Guyana.Potaro-Siparuni
Rhinophylla pumilio|ABGYF317-06|ROM 114707|Guyana.Potaro-Siparuni
Rhinophylla pumilio|ABGYC454-06|ROM 107279|Guyana.Potaro-Siparuni
Rhinophylla pumilio|ABGYC577-06|ROM 107425|Guyana.Potaro-Siparuni
Rhinophylla pumilio|ABGYF495-06|ROM 115134|Guyana.Cuyuni-Mazaruni
Rhinophylla pumilio|ABGYG178-06|ROM 115740|Guyana.Potaro-Siparuni
Rhinophylla pumilio|ABGYG390-06|ROM 116516|Guyana.Potaro-Siparuni
Rhinophylla pumilio|BCBNT989-06|ROM 114659|Guyana.Potaro-Siparuni
