# Supplementary figures and images for: Neotropical Bats: Estimating Species Diversity with DNA Barcodes
Source: PLoS One. 2011 Jul 26;6(7):e22648. doi: 10.1371/journal.pone.0022648 (PMC3144236; doi:10.1371/journal.pone.0022648)

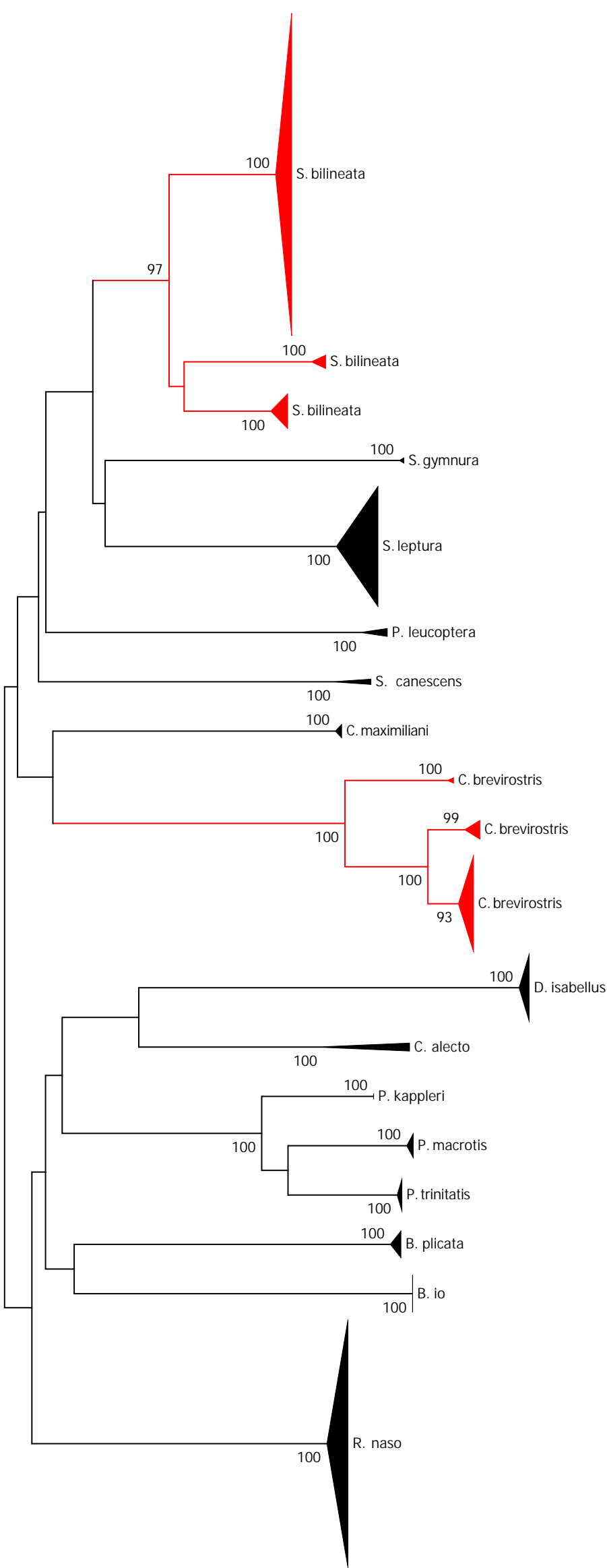

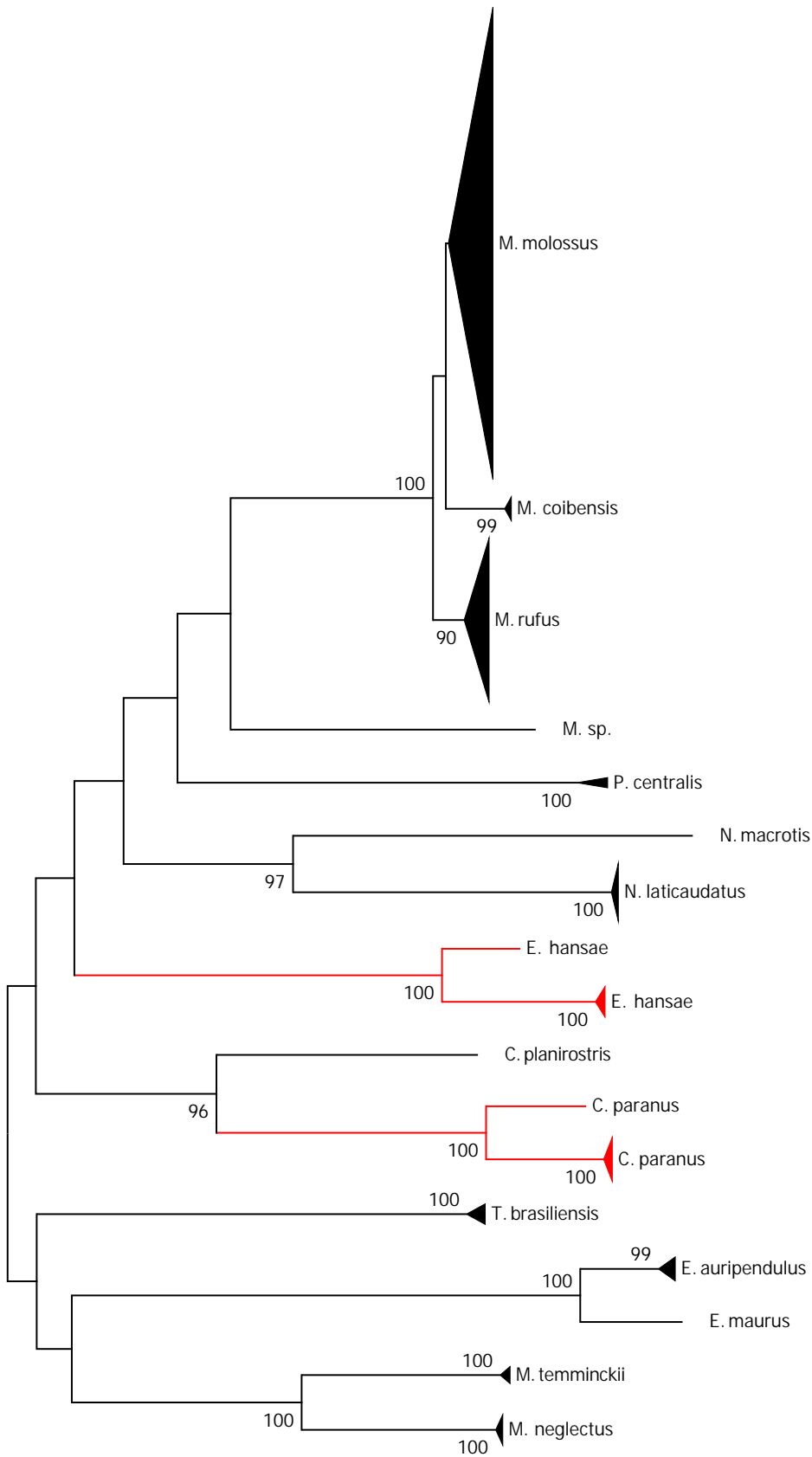

0.02

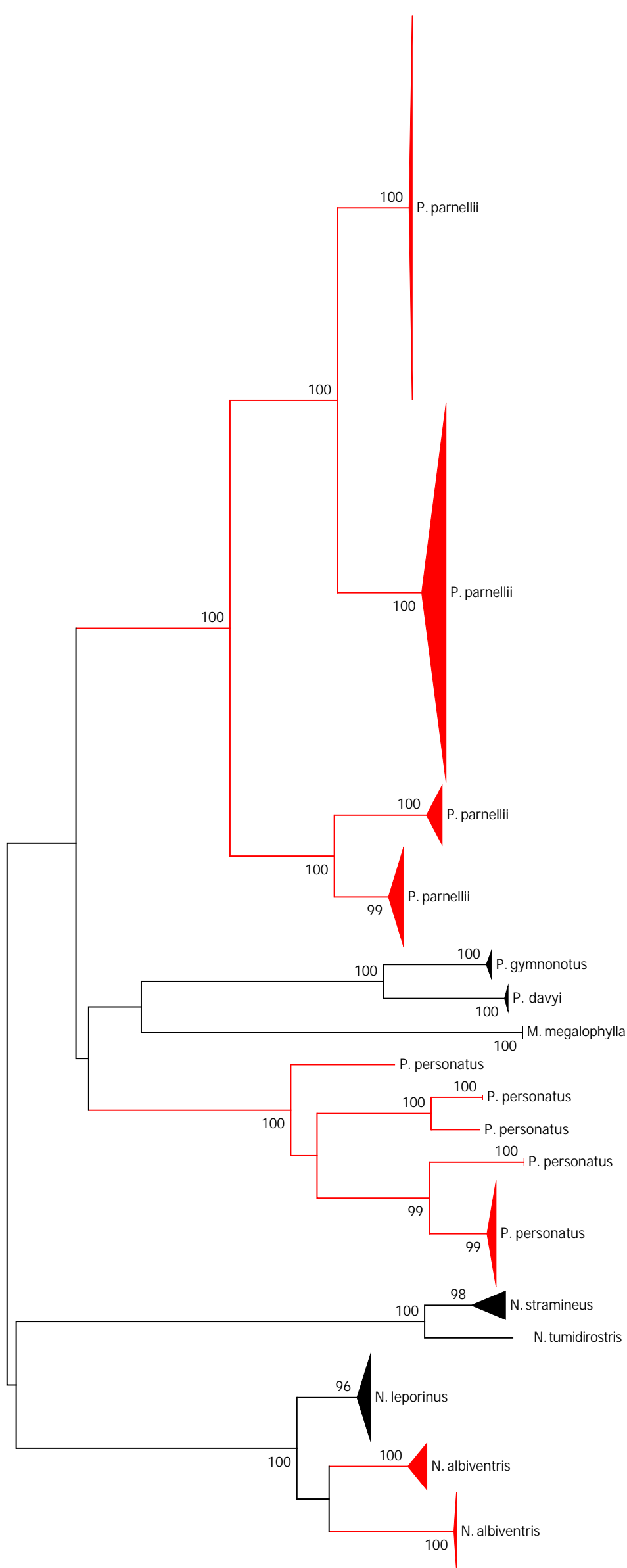

0.02

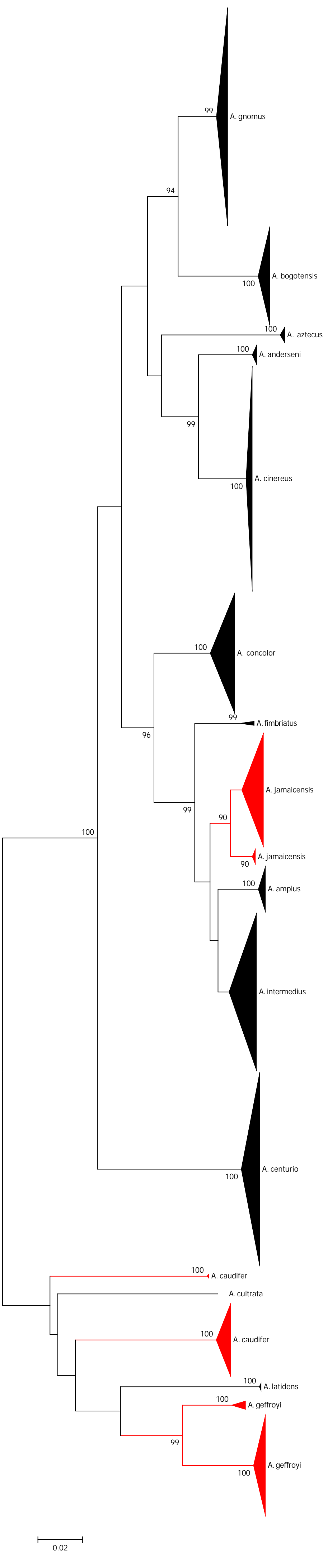

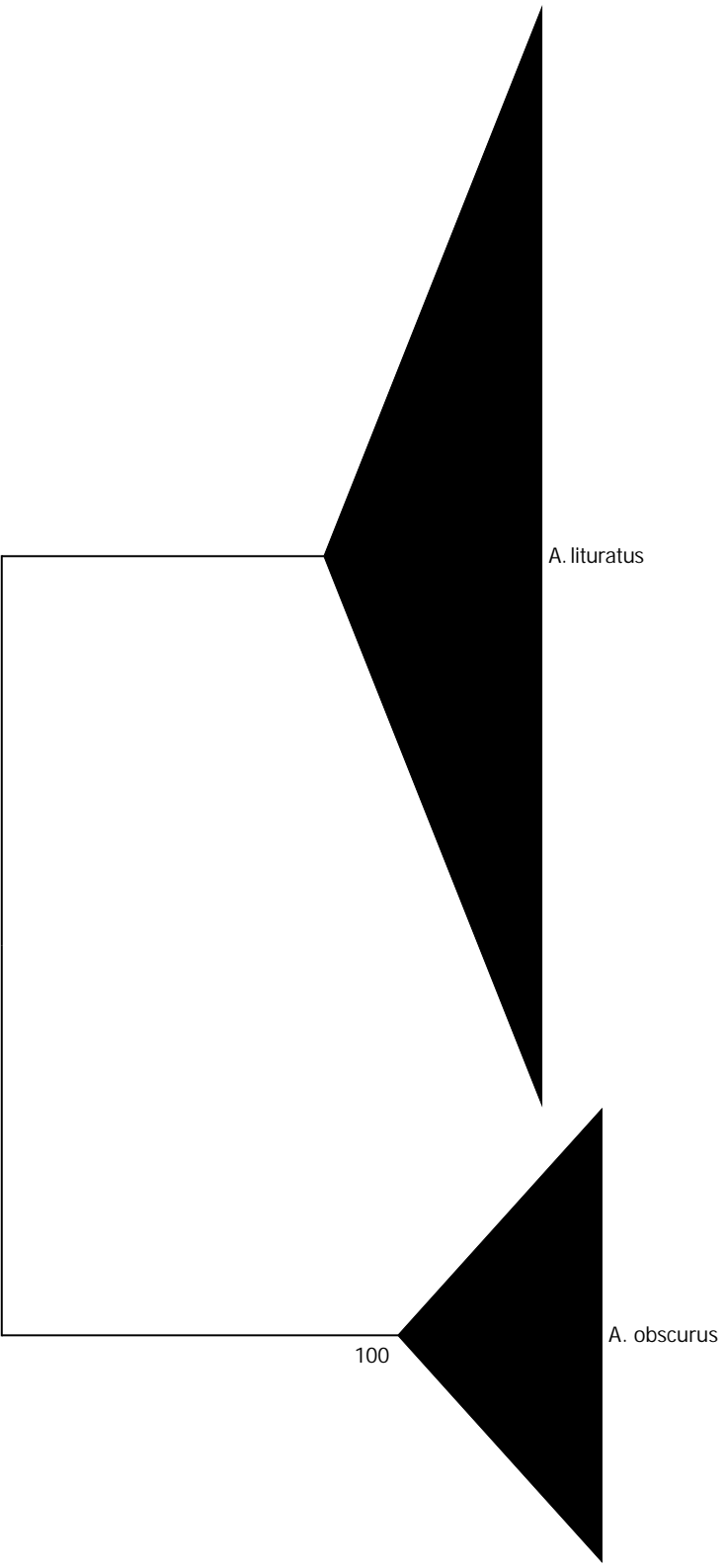

0.005

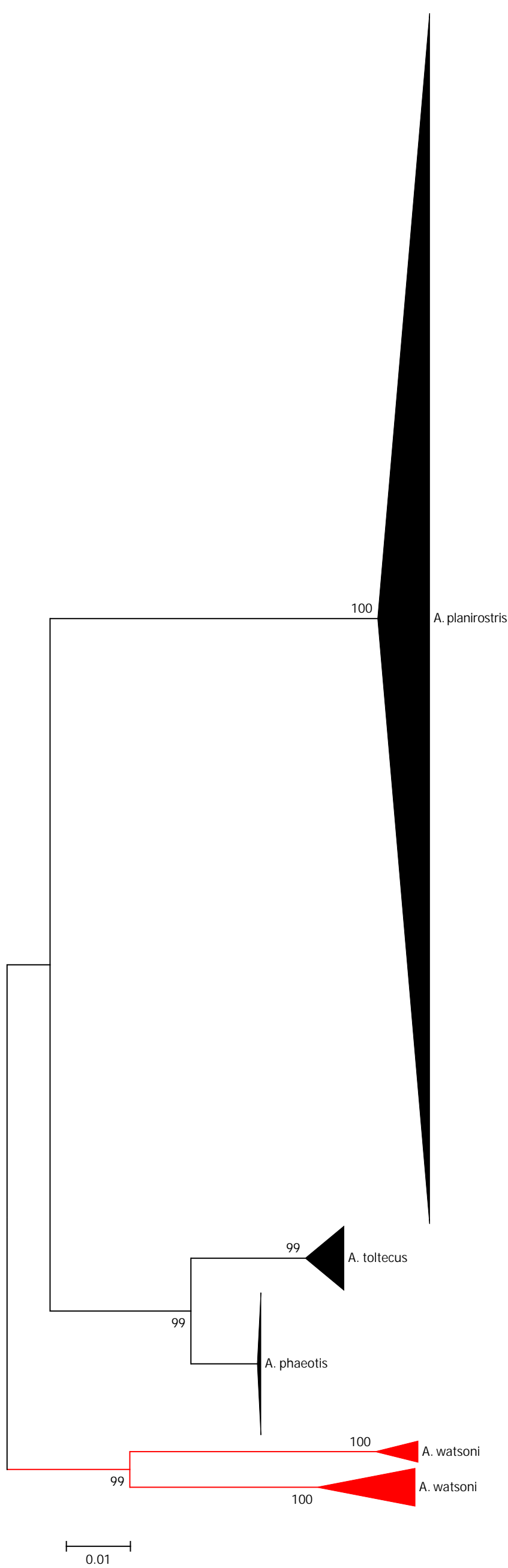

0.01

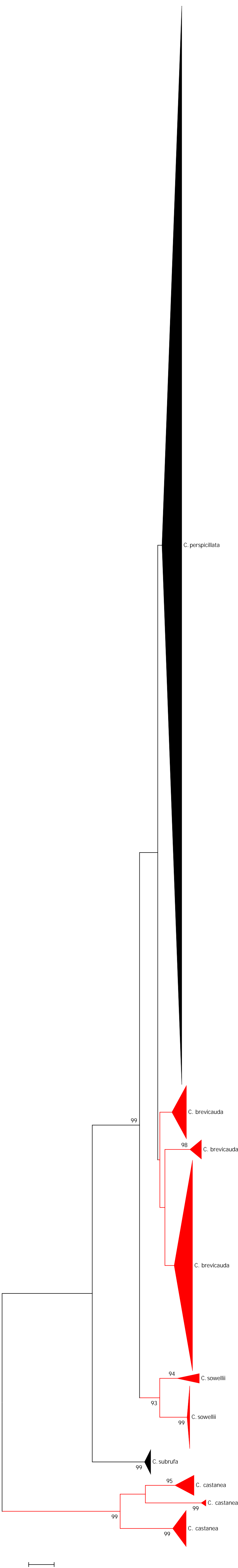



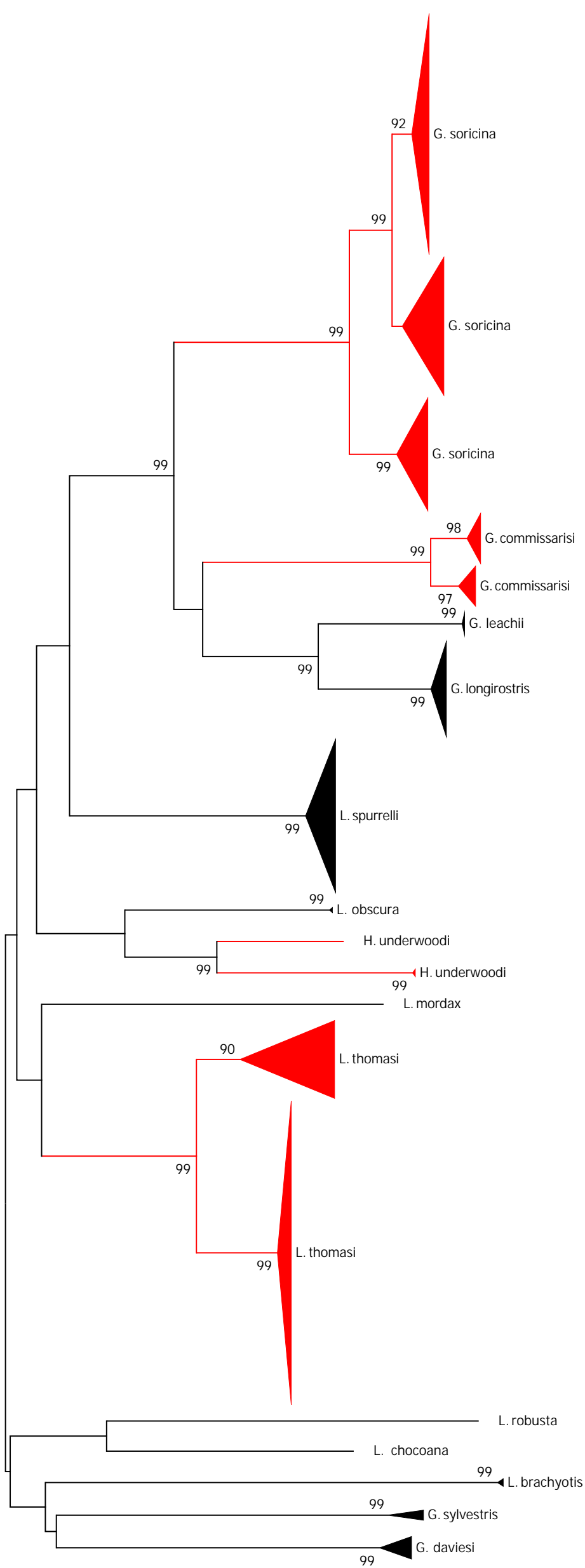

0.02

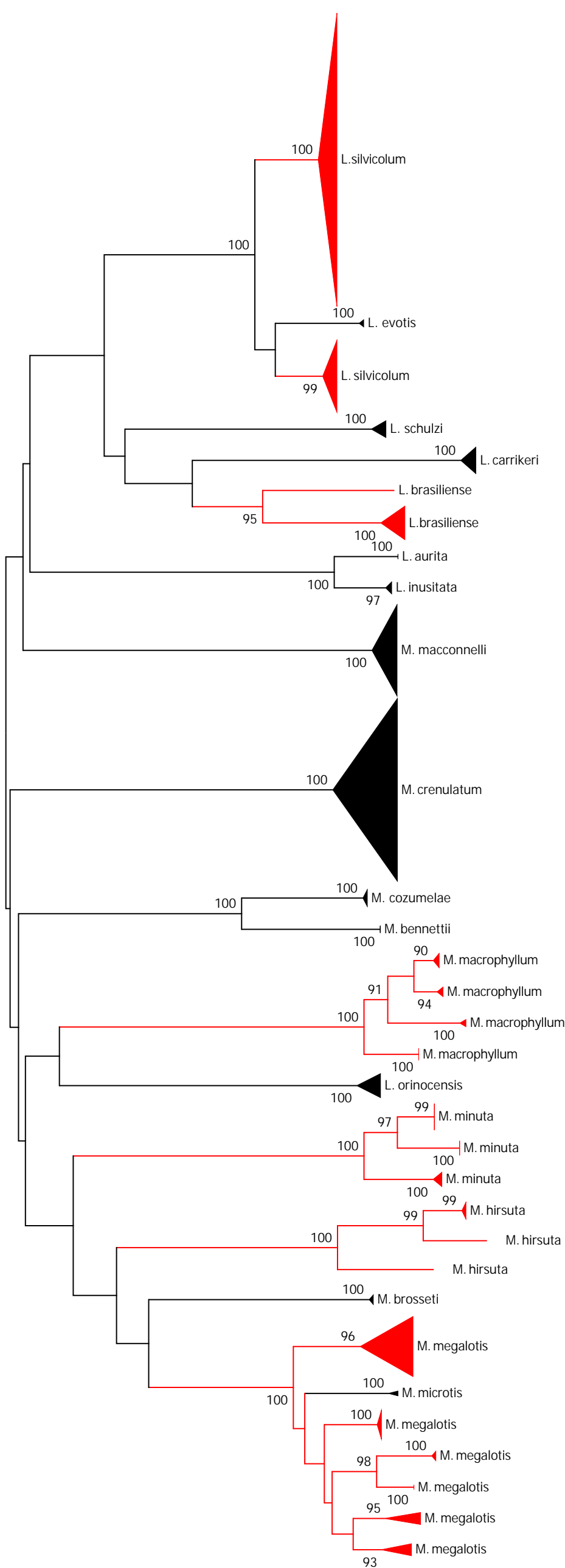

0.02

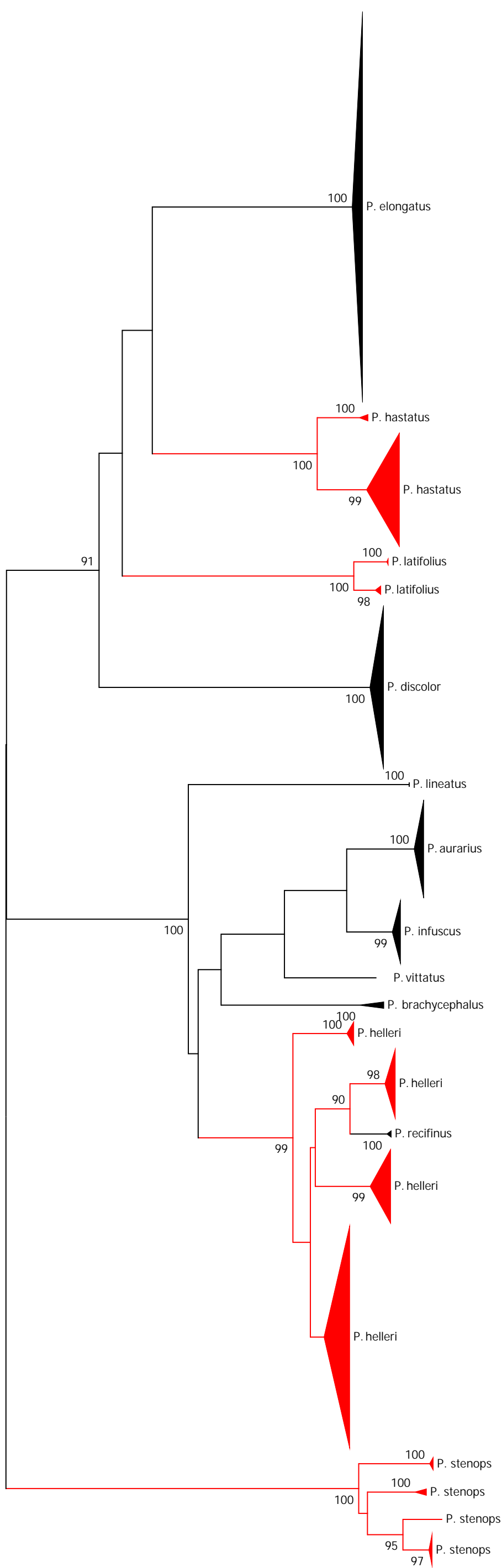

0.02

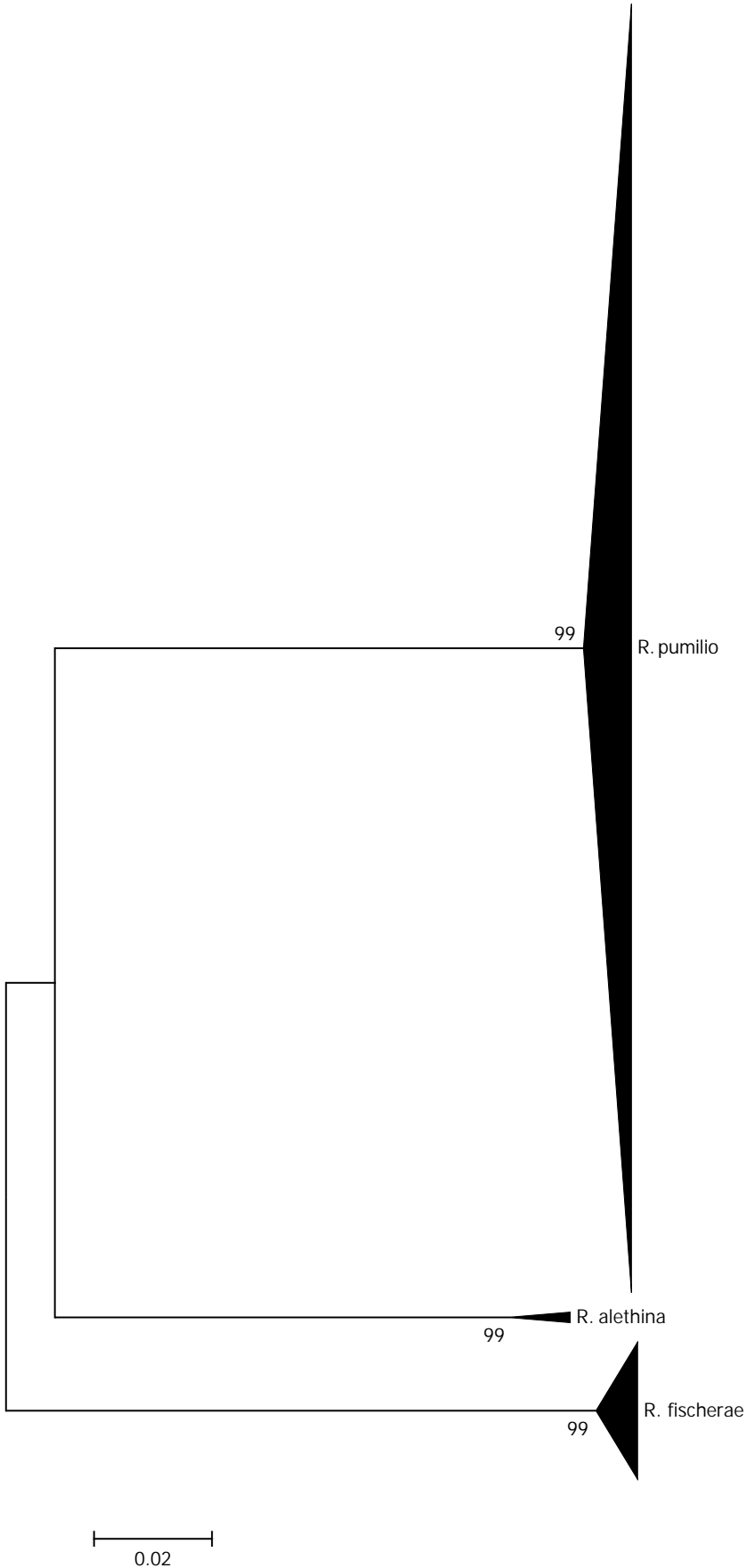

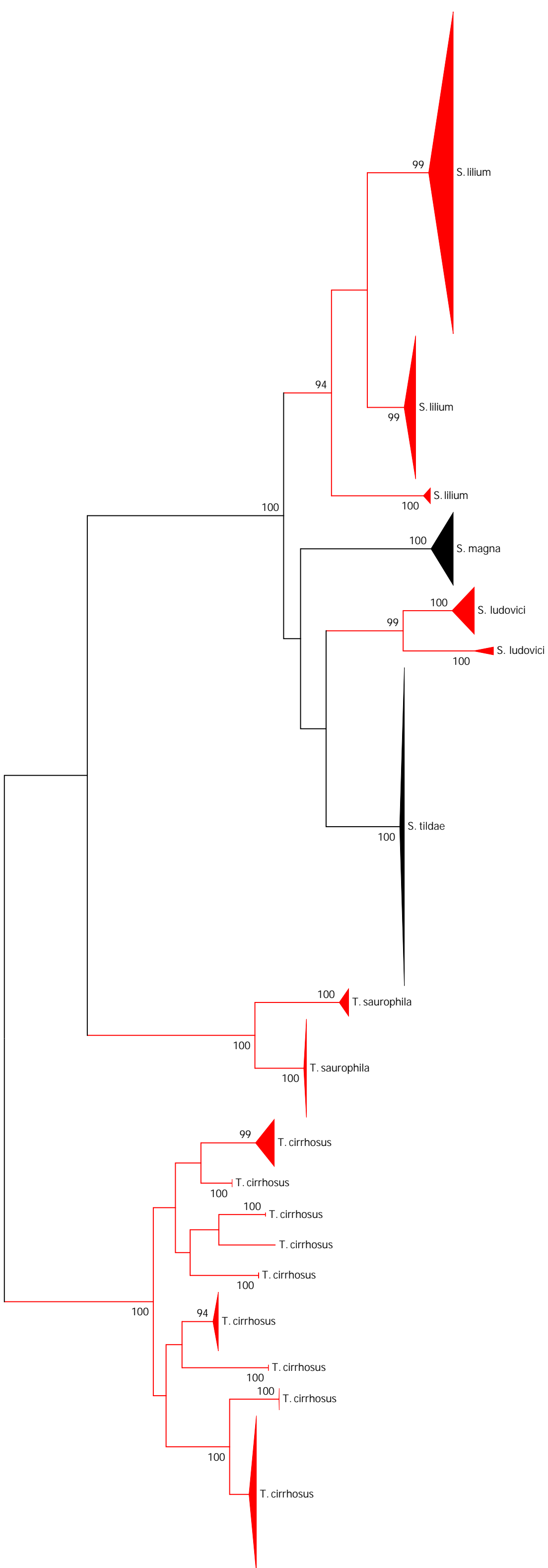

0.02

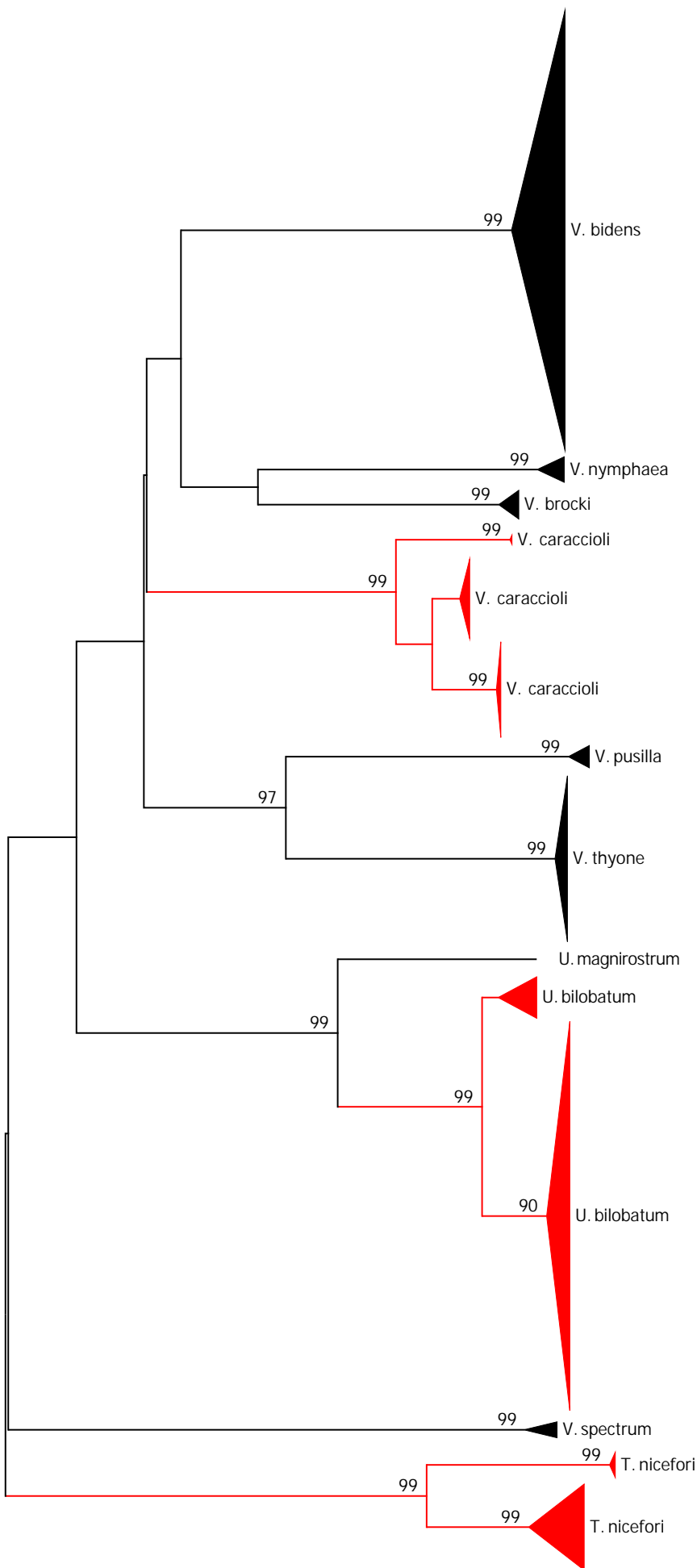

0.02

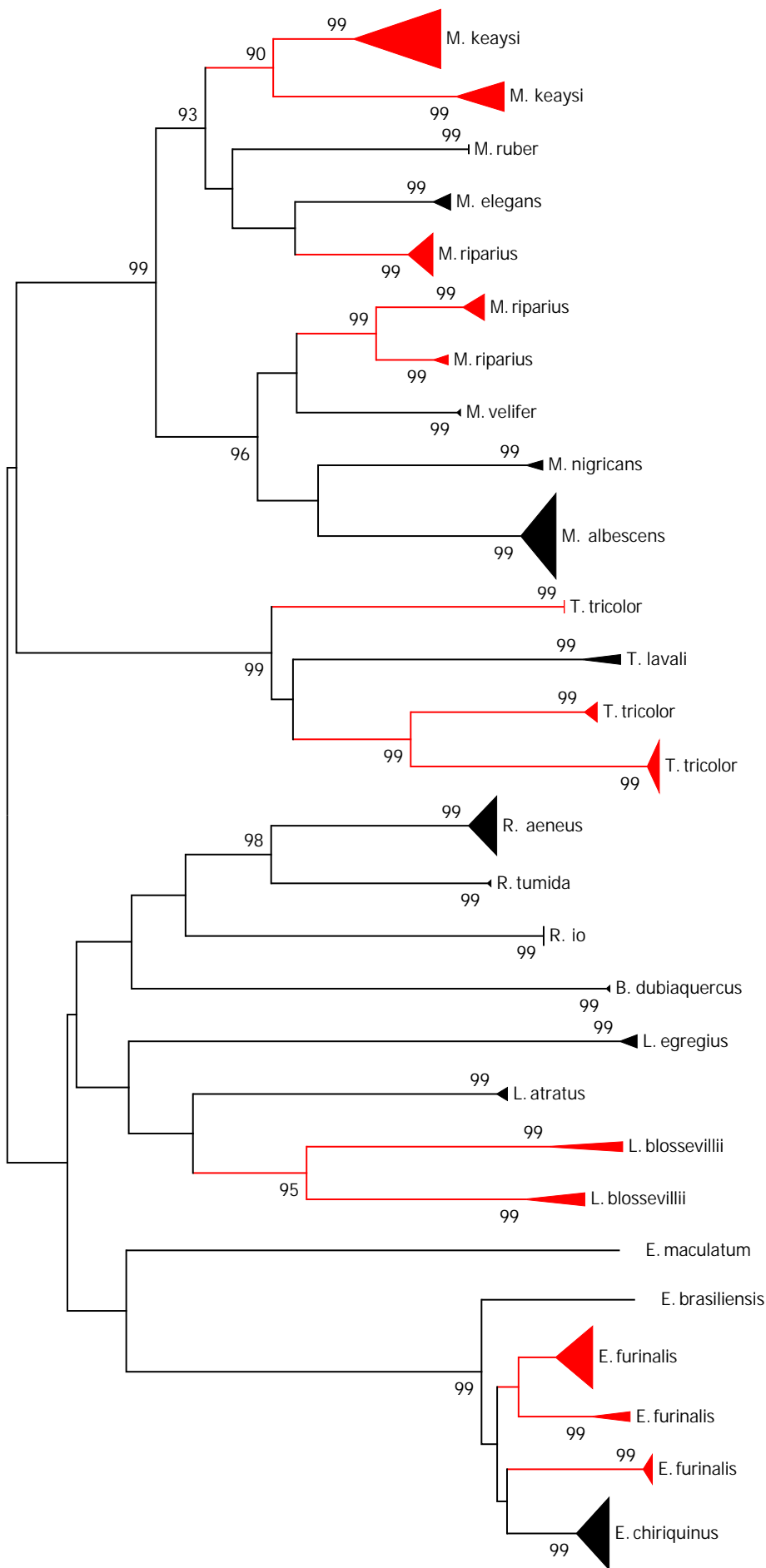

0.02

Supplement: Figure S2 — Neighbour-joining trees of COI sequence divergence (K2P) in surveyed species simplified to show current species designations and cases of deeply divergent intraspecific lineages (coloured red) in need of further systematic study. For clarity, trees were generated on subsets of the total dataset. All branch supports represent boostrap values (1000 replications). (PDF) [file pone.0022648.s002.pdf]
